# Proceedings of Reanimation 2022, the French Intensive Care Society International Congress

**DOI:** 10.1186/s13613-022-01016-6

**Published:** 2022-06-22

## Acknowledgements

### This abstract report was edited and corrected by the members of the Congress Committee of the French Intensive Care Society

Laetitia BODET-CONTENTIN^1^, Emmanuel CANET^2^, Guillaume CARTEAUX^3^, Jean-Pierre FRAT^4^, Guillaume GERI^5^, Olfa HAMZAOUI^6^, Julie HELMS^7^, Mathieu JOZWIAK^8^, Saad NSEIR^9^, Mehdi OUALHA^10^, Frédéric PÈNE^11^, Nicolas WEISS^12^

^1^CHU de Tours, Tours, France; ^2^CHU de Nantes, Nantes, France; ^3^Hôpital Henri Mondor, Assistance-Publique des Hôpitaux de Paris (AP-HP), Créteil, France; ^4^CHU de Poitiers, Poitiers, France; ^5^Clinique Ambroise Paré, Neuilly s/Seine, France; ^6^Hôpital Antoine Béclère, AP-HP Université Paris-Saclay, Clamart, France; ^7^CHU de Strasbourg, Strasbourg,France; ^8^CHU de Nice, Nice, France; ^9^CHU de Lille, Lille, France; ^10^Hôpital Necker-Enfants Malades, AP-HP, Paris, France; ^11^Hôpital Cochin, AP-HP. Centre, Université Paris Cité, Paris, France; ^12^Sorbonne Université, Groupe Hospitalier AP-HP.Sorbonne Université, Hôpital Pitié-Salpêtrière, Paris, France

## Oral communications

### CO-01 Platelet transfusion efficacy in intensive care unit: a prospective multicenter observational

#### REIZINE Florian^1^, LE MAREC Sarah^2^, LE MEUR Anthony^3^, CONSIGNY Maëlys^2^, BERTEAU Florian^1^, GESLAIN Marie^2^, LE NIGER Catherine^2^, HUNTZINGER Julien^5^, SEGUIN Philippe^1^, THIBERT Jean-Baptiste^6^, REIGNIER Jean^3^, EGRETEAU Pierre-Yves^4^, TADIÉ Jean-Marc^1^, HUET Olivier^1^, ASFAR Pierre^7^, EHRMANN Stephan^8^, AUBRON Cécile^2^

##### ^1^CHU de Rennes, Rennes, France; ^2^CHU de Brest, Brest, France; ^3^CHU de Nantes, Nantes, France; ^4^CH de Morlaix, Morlaix, France; ^5^CH de Vannes, Vannes, France; ^6^Etablissement français du sang Bretagne, Rennes, France; ^7^CHU d’Angers, Angers, France; ^8^CHU de Tours, Tours, France

###### Correspondence: Florian REIZINE (florian.reizine@gmail.com)

*Annals of Intensive Care* 2022, **12(1):**CO-01

**Rationale:** Up to 15% of critically ill patients receive platelets in intensive care units (ICU) (1). Both preventive and therapeutic platelet transfusions (PT) are not fully supported by high levels of evidence and the benefits of platelet transfusion remain subject to debates in some settings. This study aims to describe the efficacy of PT in ICU, and its impact on patients’ outcomes.

**Patients and methods/Materials and methods:** From June 2018 to November 2019, we conducted a prospective multicenter observational study recruiting patients that received at least one PT in one of the 9 participating ICUs. Inefficacy of preventive PT was defined as a Corrected Count Increment (CCI, that adjusts for the transfused platelet dose and the body weight) < 7 at 18 to 24 h after PT. Factors associated with transfusion inefficacy were assessed by performing an univariate analysis and in a mixed effect model.

**Results:** Of the 310 included patients, 119 patients (38.4%) received curative PT while 191 patients (61.6%) were treated preventively. Of the 975 transfusion episodes, 765 were given in prevention of bleeding because of low platelet count and 210 in treatment of active bleeding. PT efficacy according to the CCI was assessed in 679 preventive transfusion. Inefficacy criteria were met in 297 episodes (43.7%). Demographic and baseline characteristics associated with preventive PT inefficacy in the univariate analysis were younger age (57.7 years [Interquartile range (IQR) 44.5–66.6] versus 62.5 [53.1–69.5]; p = 0.01); immunosuppression (69.9% versus 51.8%; p = 0.025) and lower haemoglobin (8.5 g/dL [7.4–10] versus 9.7 g/dL [7.8–11.5]; p = 0.0028). Among clinical features, PT inefficacy was associated with higher heart pulse (106 [92–120] versus 99 [86–114]; p < 0.0001) and higher temperature prior to PT (37.2 [36.5–37.9] versus 37 [36.4–37.6]; p = 0.016), both possible surrogate of sepsis. Interestingly, ABO compatibility did not affect PT efficacy. The mixed effect model identified haemoglobin (Estimate (E): 1.83 [Confidence Interval 95% (CI) 0.56–3.11]; p = 0.0051), heart pulse before transfusion (Estimate: − 0.17 [− 0.3 to − 0.03]; p = 0.016), curative anticoagulation (E: 14.1 [4.36; 23.77]; p = 0.008), chronic kidney injury (E: 20.12 [0.86; 39.37]; p = 0.008) and mean age of platelet transfused (E: − 3.21 [− 5.61; − 0.81]; p = 0.009) being independently associated with the CCI.

**Conclusion:** Almost half of preventive PT in ICU do not meet efficacy criteria based on the CCI. Further research is warranted to investigate whether changes in the identified independent risk factors for PT inefficacy improve patients’ outcomes.

**Reference 1:** Arnold DM, Crowther MA, Cook RJ, et al. (2006) Utilization of platelet transfusions in the intensive care unit: indications, transfusion triggers, and platelet count responses. Transfusion 46:1286–1291. https://doi.org/10.1111/j.1537-2995.2006.00892.x

Compliance with ethics regulations: Yes in clinical research.

### CO-02 Platelet transfusion in ICU: are we applying guidelines?

#### LE MAREC Sarah^1^, REIZINE Florian^2^, LE MEUR Anthony^3^, CONSIGNY Maëlys^1^, BODENES Laetitia^1^, BERTEAU Florian^4^, GESLAIN Marie^1^, LE NIGER Catherine^1^, HUNTZINGER Julien^5^, SEGUIN Philippe^2^, THIBERT Jean-Baptiste^6^, REIGNIER Jean^3^, EGRETEAU Pierre-Yves^4^, TADIÉ Jean-Marc^2^, HUET Olivier^1^, ASFAR Pierre^7^, EHRMANN Stephan^8^, AUBRON Cécile^1^

##### ^1^CHU de Brest, Brest, France; ^2^CHU de Rennes, Rennes, France; ^3^CHU de Nantes, Nantes, France; ^4^CH de Morlaix, Morlaix, France; ^5^CH de Vannes, Vannes, France; ^6^Etablissement français du sang Bretagne, Rennes, France; ^7^CHU d’Angers, Angers, France; ^8^CHU de Tours, Tours, France

###### Correspondence: Florian REIZINE (florian.reizine@gmail.com)

*Annals of Intensive Care* 2022, **12(1):**CO-02

**Rationale:** National and international guidelines for platelet transfusion in ICU are mostly based on studies conducted in non-critically ill patients or on low level of evidence leading to the potential risk of non-conformity to those recommendations. We study the conformity to both, French guidelines performed by High Health Authority (HHA) and American guidelines by the American Association of Blood Banks (AABB).

**Patients and methods/Materials and methods:** This is a prospective multicenter observational study conducted in 9 French ICU over a 18-month period. Indications and conformity of platelet transfusion (PT) to national and international guidelines were analyzed by two independent investigators and by a third one in case of disagreement between those 2 investigators. Parameters associated with PT conformity and the impact of non-conformity to 28-day mortality were identified.

**Results:** Of the 975 platelet transfusion episodes given to 310 patients, 210 (21.5%) were transfused to treat major bleeding, 407 (41.7%) to prevent bleeding because of low platelet count, 192 (19.8%) before an invasive procedure or surgery and 166 (17%) in treatments for minor bleeding. Among them, 80% (IC 95% 77–82) were conform to the French HHA guidelines, and 62% (IC 95% 59–65) to the AABB. The factors independently associated with non-conformity to the French guidelines were preventive PT in patients with low platelet count (OR 5.28; IC 95% 3.66–7.63, p < 0.0001), and ongoing curative anticoagulation (OR 2.21; IC 95% 1.30–3.77, p = 0.0034). There was no difference in mortality between patients receiving conform and non-conform PT.

**Conclusion:** Around 20% and 40% of PT were non-conform to the national and the international guidelines, respectively. Independent risk factors for non-conformity were preventive PT for low platelet count, and ongoing curative anticoagulation. Our results highlight the need for further research to better define PT indications in ICU.

Compliance with ethics regulations: Yes in clinical research.

### CO-03 Prognostic factors of renal function in critically ill patients with hemolytic and uremic syndrome: a 6-year retrospective multicenter study

#### QUELVEN Quentin^1^, COIRIER Valentin^1^, DELAMAIRE Flora^1^, MARIOTTE Eric^2^, FILLATRE Pierre^4^, DELBOVE Agathe^3^, GUILLOT Pauline^1^, GACOUIN Arnaud^1^, TADIÉ Jean-Marc^1^, CAMUS Christophe^1^, MAAMAR Adel^1^

##### ^1^CHU Rennes, Pontchaillou, Rennes, France; ^2^Hopital Saint Louis, Paris, France; ^3^CH Bretagne Atlantique, Vannes, France; ^4^CH Yves Le Foll, Saint Brieuc, France

###### Correspondence: Quentin QUELVEN (quentinquelven@hotmail.fr)

*Annals of Intensive Care* 2022, **12(1):**CO-03

**Rationale:** Hemolytic and uremic syndrome (HUS) is a field of heterogeneous pathologies whose diagnostic and therapeutic management remain difficult. We aimed to describe clinical and biological features of critically ill patients admitted to intensive care unit (ICU) for HUS and assess the impact of common therapeutics on renal function.

**Patients and methods/Materials and methods:** We performed a 6-year retrospective multicenter study from 2014 to 2019 of patients admitted for a proven HUS in four French ICU. HUS was defined by the presence of anemia with arguments in favor of a hemolytic origin and non-autoimmune origin, associated with thrombocytopenia, or with histologic evidence of thrombotic microangiopathy. Clinical features, biological features and therapeutics initiated during the ICU stay were retrospectively collected. Glomerular filtration rate (GFR) was assessed by the Chronic Kidney Disease Epidemiology Collaboration at day-30, 90 and 180. We also assessed 30-day mortality. A repeated-measures analysis of variance was employed to assess the changes in GFR with the use of plasma exchange, Eculizumab and extra renal replacement therapy (RRT) during the ICU stay.

**Results:** We included 103 patients (median age 54 [32–65], males 48 (47%)). Twenty-seven (26%) had STEC-HUS, 13 (13%) aHUS and 59 (57%) secondary HUS. Among these patients, 71 (69%) received plasma exchange, 18 (18%) received eculizumab and 46 (45%) received renal replacement therapy (RRT) during their ICU stay. Ninety-two (89%) survived in ICU and 78 (86%) were alive at day 30. At day-180, 13 (12.6%) still required RRT. Renal function at six months was not significantly different between the patients who received plasma exchange (69 [41–91] versus 43 [23–50] mL/mn/1.73 m^2^, p = 0.17), Eculizumab (55 [50–73] versus 69 [31–91] mL/min/1.73 m^2^, p = 0.57) or RRT (49 [31–69] versus 69 [36–91] mL/mn/1.73 m^2^, p = 0.40). Moreover, ICU and hospital lengths of stay were significantly longer for patients who received plasma exchange and they had a longer duration of RRT. Finally, multivariate analysis shows that RRT in ICU is a prognosis factor independently associated with RRT at day-180.

**Conclusion:** Plasma exchange, eculizumab and RRT were not associated with an improvement of day-180 renal function in our multicenter study. Further studies are still needed to define the best therapeutic management of these patients.

**Reference 1:** Pène F, Vigneau C, Auburtin M, Moreau D, Zahar J-R, Coste J, et al. Outcome of severe adult thrombotic microangiopathies in the intensive care unit. Intensive Care Med. janv 2005;31(1):71-8

**Compliance with ethics regulations:** Yes in clinical research.
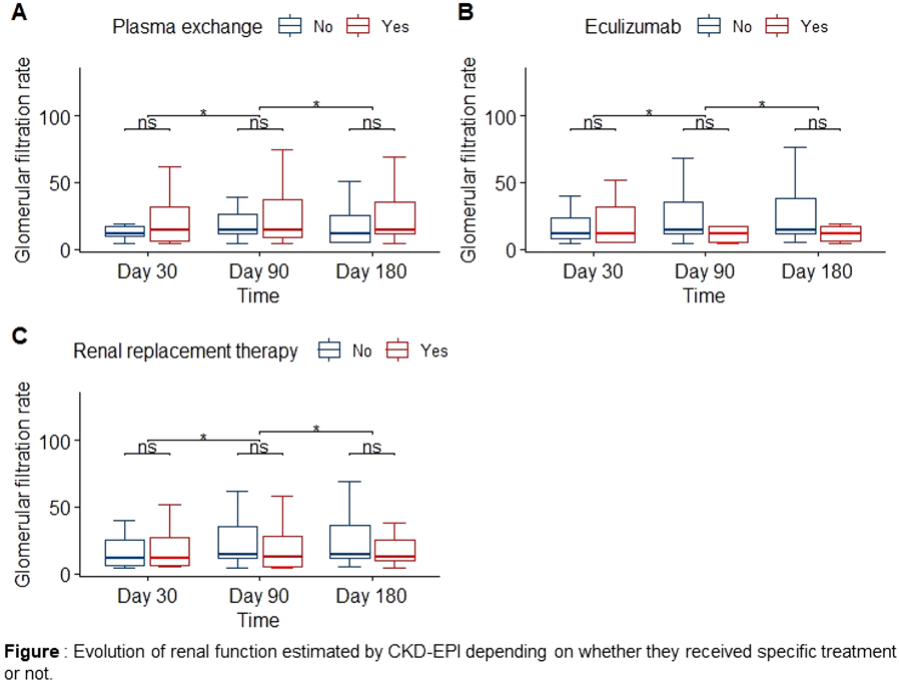


### CO-04 Epidemiology, clinical presentation, evolution and prognostic factors for survival of patients with eosinophilia in intensive care: retrospective national multicenter cohort study "REO"

#### GAILLET Antoine^7^, BAY Pierre^2^, PÉJU Edwige^8^, FAGUER Steven^4^, ASMA Mabrouki^10^, AZOULAY Elie^10^, PÈNE Frédéric^8^, TANDJAOUI-LAMBIOTTE Yacine^11^, COHEN Yves^11^, GERI Guillaume^12^, VIEILLARD-BARON Antoine^12^, PINETON DE CHAMBRUN Marc^2^, BENCHABANE Nacim^5^, LARCHER Romaric^5^, GRANGE Steven^9^, QUENOT Jean-Pierre^14^, AIT OUFELLA Hafid^13^, TIMSIT Jean-Francois^3^, MEKONTSO DESSAP Armand^7^, KAHN Jean-Emmanuel^12^, DARGENT Auguste^6^, HERAULT Antoine^9^, GROH Matthieu^1^

##### ^1^Foch, Suresnes, France; ^2^Pitié Salpêtrière, Paris, France; ^3^Bichat, Paris, France; ^4^Rangueil, Toulouse, France; ^5^Lapeyronie, Montpellier, France; ^6^Edouard Herriot, Lyon, France; ^7^Henri Mondor, Créteil, France; ^8^Cochin, Paris, France; ^9^CHU Rouen, Rouen, France; ^10^Saint Louis, Paris, France; ^11^Avicenne, Bobigny, France; ^12^Ambroise Paré, Boulogne-Billancourt, France; ^13^Saint Antoine, Paris, France; ^14^François Mitterrand, Dijon, France

###### Correspondence: Antoine GAILLET (gaillet.antoine75@gmail.com)

*Annals of Intensive Care* 2022, **12(1):**CO-04

**Rationale:** The objectives of the “REO” study are to describe the epidemiological characteristics of situations associated with eosinophilia in intensive care units (ICU) and to identify predictors of mortality in this situation.

**Patients and methods/Materials and methods:** Retrospective, national, multicenter cohort (14 centers), including adult patients (≥ 18 years old) who presented, from 01/01/2013 to 12/31/2018, eosinophilia ≥ 1000/mm^3^ on two samples (not necessarily consecutive) performed from the day before admission to the end of the stay in ICU.

**Results:** 620 patients were included (i.e. approximately 1.2% of all stays over the period and participating centers): 248 (40%) with early eosinophilia (occurring within the first 24 h of admission, ICU-Eo1 group) and 348 (56%) with delayed eosinophilia (> 24 h after admission, ICU-Eo2 group), 24 patients with no reported time to onset of eosinophilia. In the ICU-Eo1 group, eosinophilia was mostly non-idiopathic (with a high incidence of onco-haematological pathologies: 26% vs. 4% in the ICU-Eo2 group, p < 0.001), most often symptomatic (59% vs 25% for ICU-Eo2, p < 0.001), especially with respiratory involvement. Eosinophilia in patients in the ICU-Eo2 group was most often idiopathic or related to drug hypersensitivity (85% vs. 47% in patients in the ICU-Eo1 group, p < 0.001), and visceral skin involvement was at first plan (72% of visceral damage in ICU-Eo2 vs. 32% in the ICU-Eo1 group). Nevertheless, in 14% of ICU-Eo2 patients, an “unexpected” etiology (particularly parasitic, onco-haematological) was identified, especially when the patient had a prior history of eosinophilic disease, was admitted for acute respiratory distress or had an eosinophilia peak > 5000/mm^3^ during ICU stay. The risk factors for mortality at day 60 highlighted in multivariate analysis for the ICU-Eo1 group were age > 65 years (HR 1.78 95% CI [1.02; 3.13] p = 0.044), current immunosuppressant therapy at ICU admission (HR 2.32 95% CI [1.18; 4.54] p = 0.014), SOFA score > 8 (HR 2.26 95% CI [1.17; 4.39] p = 0.015), admission for acute respiratory distress (HR 2.43 IC95% [1.06; 5.60] p = 0.037) or for shock (HR 3.08 95% CI [1.15; 8.29] p = 0.026) and finally that the current eosinophilia is of onco-haematological origin (HR 4.41 [2.47; 7.90] p < 0.001). About the ICU-Eo2 group, only the use of invasive ventilation during the stay was associated with poor prognosis (HR 5.54 [1.32; 23.35] p = 0.019).

**Conclusion:** In ICU, two distinct populations of eosinophilia seem to be defined according to the time of onset of this one. When eosinophilia is present on admission, the onco-haematological origin particularly affects the prognosis.

**Compliance with ethics regulations:** Yes in clinical research.

### CO-05 Etoposide-containing regimens for the treatment of critically ill patients with hematological malignancy-related hemophagocytic lymphohistiocytosis

#### LE STANG Valentine^1^, VIGNERON Clara^2^, DECROOCQ Justine^2^, PÉJU Edwige^2^, BURRONI Barbara^2^, CHAPUIS Nicolas^2^, CHARPENTIER Julien^2^, PÈNE Frédéric^2^

##### ^1^La Pitié Salpétrière, Paris, France; ^2^Hôpital Cochin, Paris, France

###### Correspondence: Valentine LE STANG (vavaw_100@hotmail.fr)

*Annals of Intensive Care* 2022, **12(1):**CO-05

**Rationale:** Hemophagocytic lymphohistiocytosis (HLH) related to hematological malignancies is a rare condition likely responsible for life-threatening multiple organ failure. Etoposide is often used as a first-line treatment in this setting but its impact on the course of HLH-defining criteria and organ failures has not been investigated.

**Patients and methods/Materials and methods:** We conducted an 8-year (2013–2020) single-center retrospective study in a medical ICU of a tertiary-care center, including adult patients with severe hematological malignancy-related HLH treated with etoposide.

**Results:** Twenty-four patients were analyzed, including 17 with lymphoma. Median H-score was 253.0 [226.3–287.0] and 16 patients fulfilled at least five HLH-2004 diagnostic criteria. All patients received first-line etoposide treatment, combined with corticosteroids in 21 patients, followed by combination chemotherapy in 16 patients after a median of 4 days [1.5–7.0]. Following etoposide initiation, body temperature decreased from 38.9 °C [38.0–39.5] to 37.6 °C [36.8–39.0] within 24 h (p = 0.01) (Fig. 1) and ferritin levels decreased by 59.8 ± 28.9% within 7 days. Lactate level decreased from 3.0 mmol/L [1.5–4.3] to 2.1 mmol/L [1.6–3.0] (p = 0.02) within 24 h from etoposide (Fig. 1). Patients exhibited early and transient deterioration in non-platelet SOFA score (npSOFA) that significantly increased from 5 [1.3–10] at etoposide initiation to 9.5 [2–15] at day 1, and thereafter improved to npSOFA value of 7 [1–11] at day 2 (Fig. 1). In-ICU, in-hospital and 1-year mortality were 45.8%, 54.2% and 66.7%, respectively.

**Conclusion:** Etoposide as single treatment or as part of sequential combination regimen was able to rapidly dampen some dynamic HLH-defining variables and to eventually improve organ failures.

**Compliance with ethics regulations:** Yes in clinical research.

### CO-06 Clinical significance of thrombocytopenia in patients with septic shock

#### PÉJU Edwige^1^, FOUQUÉ Gaëlle^1^, CHARPENTIER Julien^1^, VIGNERON Clara^1^, JOZWIAK Mathieu^2^, MIRA Jean-Paul ^1^, CARIOU Alain^1^, JAMME Matthieu^3^, PÈNE Frédéric^1^

##### ^1^APHP/Hôpital Cochin, Paris, France; ^2^CHU de Nice/Hôpital l'Archet, Nice, France; ^3^Hôpital CHI Poissy/Saint-Germain-en-Laye, Paris, France

###### Correspondence: Edwige PÉJU (edwigepeju@gmail.com)

*Annals of Intensive Care* 2022, **12(1):**CO-06

**Rationale:** Thrombocytopenia is a common disorder in critically ill patients and is known to be associated with poor prognosis. Whether thrombocytopenia accounts for a bystander of severity or may drive specific complications is unclear. With respect to the various immune and procoagulant functions of platelets, the aim of this study is to address the impact of thrombocytopenia on the development of ICU-acquired infections, bleeding and ischemic events in high-risk patients with septic shock.

**Patients and methods/Materials and methods:** This was a single-center retrospective study conducted in a medical ICU over a 12-year period (2008–2019). Patients admitted for septic shock (Sepsis-2 definition) were included if still alive in the ICU after 48 h, to retain patients at risk of further ICU-acquired complications. Morning platelet counts were collected daily. Groups of patients were defined according to 7-day platelet count trajectories estimated using latent class mixed model. Outcomes were ICU mortality and ICU-acquired complications including infections, severe bleeding events (WHO grade 3–4) and thrombotic events.

**Results:** The cohort comprised 1024 48-h survivors. Among them, 39% were immunocompromised, including one third with hematological malignancies. Upon admission, 153 patients (15%) had profound thrombocytopenia with platelet count below 50 G/L. The in-ICU mortality rate was 27% (n = 281). ICU-acquired infections, severe bleeding events and thrombotic events occurred in 28%, 13% and 12% of patients, respectively. The latent class model identified five subgroups with consistent 7-day platelet count trajectories. The subgroup of patients with initial low and sustained thrombocytopenia (n = 224) was associated with high prevalence (39%) of hematologic malignancies and displayed increased mortality when compared to patients with alternative platelet trajectories (49% vs. 13%, 21%, 30%, 24%, p < 0.001). In multivariate analysis, initial low and sustained thrombocytopenia (cause-specific hazard (CSH) = 2.96 [95% CI 2.16–4.06], p < 0.001) and initial normal with fast decrease in platelet count (CSH = 2.21 [1.19–4.12], p = 0.01) were independently associated with mortality. Sustained profound thrombocytopenia was independently associated with increased risk of bleeding (CSH = 1.57 [1.02–2.65], p = 0.004). Platelet count trajectories were not significantly associated with the development of ICU-acquired infections and thrombotic events.

**Conclusion:** Patterns of thrombocytopenia are associated with outcomes in septic shock. Sustained profound thrombocytopenia and fast relative thrombocytopenia were both independent determinants of ICU mortality. Sustained profound thrombocytopenia was associated with an increased risk of severe bleeding. Platelet count trajectories did not impact on the risk of ICU-acquired infections or thrombotic events.

**Compliance with ethics regulations:** Yes in clinical research.

### CO-07 Reliability of peak inspiratory pressure to estimate plateau pressure in infants with severe virus airway infection

#### ROTAVA BURATTI Cecília^1,2,3^, JOUVET Philippe^3^, ANDREOLIO Cinara^1,2^, BRUNO Francisco^1,2^, ANDRADE Lívia^2^, MARCON Mônica^1^, NAVARRO Nádia^1,2^, PIVA Jefferson^1,2^

##### ^1^Hospital de Clínicas de Porto Alegre, Porto Alegre, Bresil; ^2^Postgraduate program in child and adolescent health, Universidade Federal do Rio Grande do Sul, Porto Alegre, Brazil; ^3^Sainte Justine Research Center, Montreal, Canada

###### Correspondence: Rotava Buratti CECÍLIA (ceciliaburatti@gmail.com)

*Annals of Intensive Care* 2022, **12(1):**CO-07

**Rationale:** Peak inspiratory pressure (PIP) measured in decelerating-flow mode (PIPdc), i.e. pressure modes, is close to plateau pressure (Pplat) in children with pediatric acute respiratory distress syndrome^1^. Since one third of children admitted to the PICU are less than 1 year old and that bronchiolitis is the leading cause of hospitalization in this age group^2^, our aim was to evaluate the reliability of PIPdc to estimate Pplat in volume control (VC) in infants with severe respiratory virus infection (SRVI), which is characterized by increased airway resistance.

**Patients and methods/Materials and methods:** A retrospective observational study was developed including infants diagnosed as SRVI, mechanically ventilated, and with respiratory mechanics measurements taken between 2017 and 2021. Measurements were taken during inspiratory and expiratory pauses after switching ventilatory mode from pressure control to volume control in patients without respiratory effort. Statistical analysis included paired t-test, Pearson correlation (r) and coefficient of determination (R^2^).

**Results:** Thirty-seven patients were included with a median age of 3 months (2–5), a *Pediatric Index of Mortality-2* of 0.2% (0.2–0.6) and a mean oxygen saturation index of 4.3 ± 1. Inspiratory (RIaw) and expiratory airway resistance (REaw) were 136 ± 43 and 168 ± 66 cmH_2_O/L/s, respectively, and static compliance: 0.75 ± 0.30 ml/kg/cmH_2_O. PIPdc was lower than PIP in VC, but still higher than measured Pplat (33 ± 3, 36 ± 5 and 26 ± 5 cmH_2_O respectively, p = 0.01 between PIP and Pplat). PIPdc overestimates Pplat with a moderate correlation (r = 0.57, p < 0.001 and R^2^ = 0.33, p < 0.001) and a mean difference of 7.3 ± 4 cmH_2_O **(Figure).**

**Conclusion:** A large difference between PIP and Pplat can be observed in infants with a significant increase in airway resistance. In the absence of Pplat measurement in a static condition, any estimation of Pplat from PIP needs to consider the resistive component of respiratory system.

**Reference 1:** Patel B et al.: Agreement Between Peak Inspiratory Pressure in Decelerating-Flow Ventilation and Plateau Pressure in Square-Flow Ventilation in Pediatric Acute Respiratory Distress Syndrome. Pediatr Crit Care Med 2022.

**Reference 2:** Fujiogi M et al.: Trends in bronchiolitis hospitalizations in the United States: 2000–2016. Pediatrics 2019.

**Compliance with ethics regulations:** Yes in clinical research.
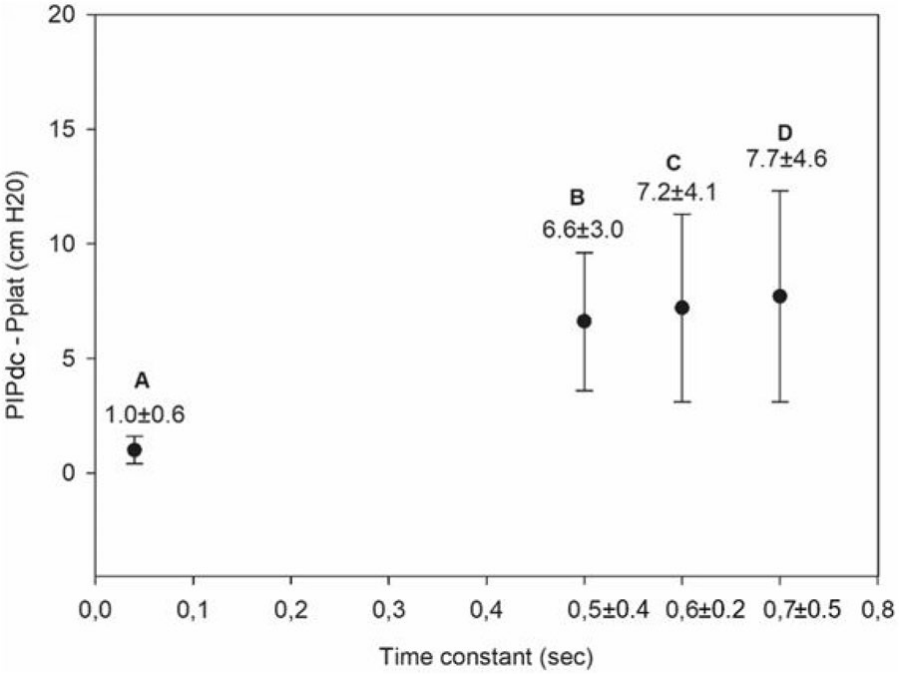


*Difference between positive inspiratory pressure in decelerating-flow modes (PIPdc) and plateau pressure (Pplat) in volume control according to time constant, in mechanically ventilated children.*

**Figure Legend:** Difference between positive inspiratory pressure in decelerating-flow modes (PIPdc) and plateau pressure (Pplat) in volume control according to time constant, in mechanically ventilated children. **Panel A:** cohort of Patel et al.^1^, patients with pediatric acute respiratory distress syndrome n = 52, median age 8.6 yo. And from our sample it has been shown **Panel B, C and D. Panel B:** patients with severe respiratory virus infection (SRVI) n = 9, median age 4 mo, RIaw 50–100 cmH_2_O/L/s; **Panel C:** patients with SRVI n = 13, median age 3 mo, RIaw 100–150 cmH_2_O/L/s; **Panel D:** patients with SRVI n = 15, median age 3 mo, RIaw 150–200 cmH_2_O/L/s. RIaw: inspiratory airway resistance; REaw: expiratory airway resistance.

### CO-08 Predictive factors of high flow nasal canula (HFNC) short term failure in severe acute bronchiolitis

#### ZINI Justine^1^, VAUGIER Isabelle^1^, BERGOUNIOUX Jean^1^

##### ^1^CHU Raymond Poincaré, Garches, France

###### Correspondence: Justine ZINI (justine.zini@gmail.com)

*Annals of Intensive Care* 2022, **12(1):**CO-08

**Rationale:** High flow oxygen nasal cannula therapy (HFNC) is a non-invasive respiratory support increasingly used to treat bronchiolitis induced respiratory failure. HFNC utilisation spread in pediatric emergencies department because of its affordable material and ease of use. However, regular monitoring and clinical assessment of the HFNC treated child by emergency physicians and nurses is needed. Its use has been shown to avoid transfers to intensive care that is a limited resource in epidemic period. However, at the early time of its implementation, it is difficult and yet critical for the security of the patient to predict its efficiency in treating the bronchiolitis induced respiratory failure.

**Objective:** The aim of this study was to identify factors predicting early failure of HFNC once it has been implemented in a pediatric emergency department to treat bronchiolitis induced respiratory failure.

**Patients and methods/Materials and methods:** This is a retrospective multicenter study in 4 Ile-de-France region hospitals (France), comparing biometric, anamnestic, clinical, biological and radiological patient date before and until 12 h HFNC treatment. Patients included were aged 0 to 12 months, admitted to the pediatric emergency department and diagnosed with acute bronchiolitis with initiation of high-flow oxygen therapy within 12 h of arrival in the pediatric emergency department.

**Results:** 160 patients were included with 52 HFNC failures. Abnormal consciousness and apneas during the use of high-flow oxygen therapy were a predictor of early high-flow oxygen therapy failure (p = 0.0001). We showed a correlation of lower the pH, higher PCO_2_ and bicarbonate levels with early HFNC failure (p < 0.001). Similarly, lack of improvement in pH (p = 0.03), PCO_2_ (p = 0.017), and bicarbonate levels (p = 0.0005) shortly HFNC implementation are predictors of high-flow oxygen therapy failure. Chest X-ray atelectasis was a predictive factor for failure of OHD (p = 0.003). Wheezing heard before and during HFNC was associated with success (p = 0.01).

**Conclusion:** Our results showed that blood gases before and after HFNC implementation were a major predictive element for success or failure in the very first hours of HFNC treatment but that blood gases bicarbonate blood levels were also strongly correlated to HFNC success. Several clinical criteria were also associated with success such as the presence of wheezing sound, atelectasia, hypoxemia before HFNC. We believe that those results may help pediatric emergency physicians to anticipate HFNC success or failure in infant with acute bronchiolitis presenting to the pediatric emergency department.

**Reference 1:** The New England Journal of Medicine 2018 “A Randomized Trial of High-Flow Oxygen Therapy in Infants with Bronchiolitis.” Donna Franklin.

**Reference 2:** Eur Respir J 2020 “A randomised trial of high-flow nasal cannula in infants with moderate bronchiolitis” Philippe Durand.

**Compliance with ethics regulations:** Yes in clinical research.
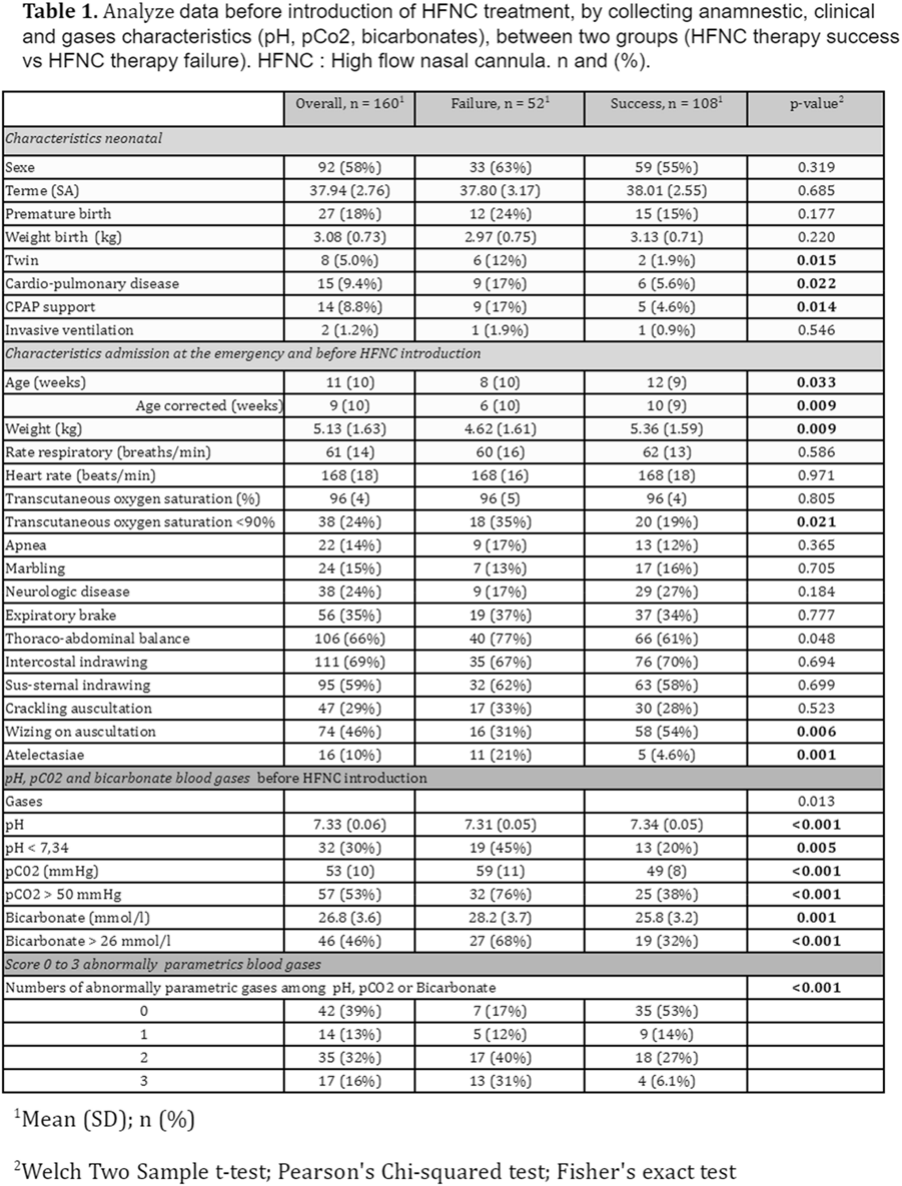


*Table 1. Analyze data before introduction of HFNC treatment, by collecting anamnestic, clinical and blood gas characteristics (pH, pCO*_*2*_ *, bicarbonates), between two groups (HFNC therapy success vs HFNC therapy failure). HFNC: High flow nasal cannula. n and (%)*

### CO-09 Mechanical ventilator settings during extracorporeal membrane oxygenation for pediatric acute respiratory distress syndrome: An European observational study

#### RAMBAUD Jerome^1^, BROMAN Lars^2^, VISCONTI Federico^6^, LEGER Pierre Louis^1^, BUTRAGUEÑO LAISECA Laura ^5^, PILOQUER Jean Eudes^4^, DINARDO Matteo^3^

##### ^1^Armand-Trousseau, Paris, France; ^2^Karolinska Institute, Stockholm, Suede; ^3^Ospedale Pediatrico Bambino Gesù, Rome, Italie; ^4^Nantes universitary hospital, Nantes, France; ^5^Hospital General Universitario Gregorio Marañón, Madrid, Espagne; ^6^University Hospital of Padova, Padoue, Italie

###### Correspondence: Jerome RAMBAUD (jerome.rambaud@aphp.fr)

*Annals of Intensive Care* 2022, **12(1):**CO-09

**Rationale:** The main objective of the study was to first describe which settings are used for mechanical ventilation during ECMO for pediatric acute respiratory distress syndrome. The secondary objective of this study is to identify risk factors associated with poor prognosis or uncomplete recovery.

**Patients and methods/Materials and methods:** A retrospective observational study in five European centers. All children aged 1 month—18 years supported by ECMO for refractory P-ARDS from January 2009 to December 2019 were included. Collected data were pre-ECMO clinical score invasive ventilation parameters before ECMO and at day 1, 3, 7 and 14 of assistance (positive end-expiratory pressure, mean pressure, plateau pressure, driving pressure), adjunctive therapies during ECMO (prone positioning, steroids, tracheostomy, bronchoscopy and cross-sectional imaging). Finally, we gathered outcomes parameters (survival rate, length of ECMO and invasive ventilation).

**Results:** We included 256 patients. Median oxygenation index and oxygenation saturation index were 37 and 24.7, respectively. Half of the P-ARDS cases were viral pneumonia. Before ECMO implantation, prone positioning and neuromuscular blockers were used in 39% and 61% of the patients. Veno-venous ECMO was offered in 62%, and dual-lumen cannula was used in one quarter of VV ECMO cases. Preferential ventilator mode during ECMO was barometric setting during the whole study period. Positive end-expiratory pressure (PEEP) and mean airway pressure were significantly different between centers. Prone positioning (16%), recruitment maneuvers (2%), cross sectional imaging (8%), and tracheostomy (3%) during ECMO remained limited. Higher PEEP and higher mean airway pressure during the whole study period were associated with higher mortality.

**Conclusion:** Ventilatory setting during ECMO for acute respiratory distress syndrome are highly dependent on center. Associations between high PEEP and mean airway pressure with lower survival rate raise the question to define the best way to manage patients under ECMO for ARDS.

Compliance with ethics regulations: Yes in clinical research.

### CO-10 Pediatric microcirculation monitoring during ECMO weaning

#### SUC Violette^1^, RAMBAUD Jerome^1^, STARCK Julie^1^, LEGER Pierre-Louis^1^

##### ^1^Hôpital Armand Trousseau, Paris, France

###### Correspondence: Violette SUC (violette.suc@gmail.com)

*Annals of Intensive Care* 2022, **12(1):**CO-10

**Rationale:** ECMO is an extracorporeal respiratory and circulatory support used in children for severe respiratory failure or refractory shock states. The usual monitoring of patient under ECMO makes it possible to understand the macrocirculatory system. Perfusion and tissue oxygenation are represented by microcirculation. It has been shown to be a powerful prognostic factor for survival in children in septic shock (reference 1). The duration of ECMO depends on the remission of the organs. Early and successful weaning from ECMO reduces morbidity and mortality of the technique. The aim of the study was to determine the relevance of the sublingual microcirculation to predict the risk of “difficult weaning” of ECMO in children, expressed by the occurrence of severe hemodynamic failure during the clamping of ECMO.

**Patients and methods/Materials and methods:** A single-center prospective study on patients hospitalized in pediatric intensive care at Trousseau Hospital between March 2017 and December 2020. A video analysis in sidestream dark field was performed for all patients at the weaning time as well as an echocardiography and a blood gas. Patients were classified into two groups: “easy weaning” or “difficult weaning” depending on the outcome after ECMO clamping. Difficult weaning group was defined as patients with hemodynamic failure and need of restart ECMO within 24 h of the weaning, or patients who presented one of the following criteria: hemodynamic failure requiring introduction or increase of vasopressorss, cardiac dysfunction requiring introduction or increase of inotropic drugs, pulmonary arterial hypertension requiring vasodilator treatment, ventilation increase (Pmax > 28–30 cmH_2_0), refractory hypoxemia (SpO_2_ < 94% with FiO_2_ > 75%) or hypercapnic acidosis (pH < 7.30).

**Results:** 30 patients were included in the study (13 newborns, median age at admission 29 days [1–770]) including 18 (60%) in veino-arterial ECMO. 19 patients were classified in the “easy weaning” group and 11 in the “difficult weaning” group. The small vessel Microvascular Flow Index at weaning was high in both groups (median 2.3 [1.8-2.4] vs 2.3 [2.3-2.6], p = 0.24) without a significant difference (Table 1). There were no differences in macro-circulatory indices, nor by ECMO type or age. The microcirculation parameters were not a predictive factor of mortality.

**Conclusion:** Our study is the first pediatric study to present microcirculation data at the ECMO weaning time. The microcirculation parameters at the weaning time in ECMO don’t seem to be predictive of success or failure of weaning. Post-clamping analysis may be relevant.

**Reference 1:** Top APC, Ince C, de Meij N, van Dijk M, Tibboel D. Persistent low microcirculatory vessel density in nonsurvivors of sepsis in pediatric intensive care. Crit Care Med. 2011.

**Compliance with ethics regulations:** Yes in clinical research
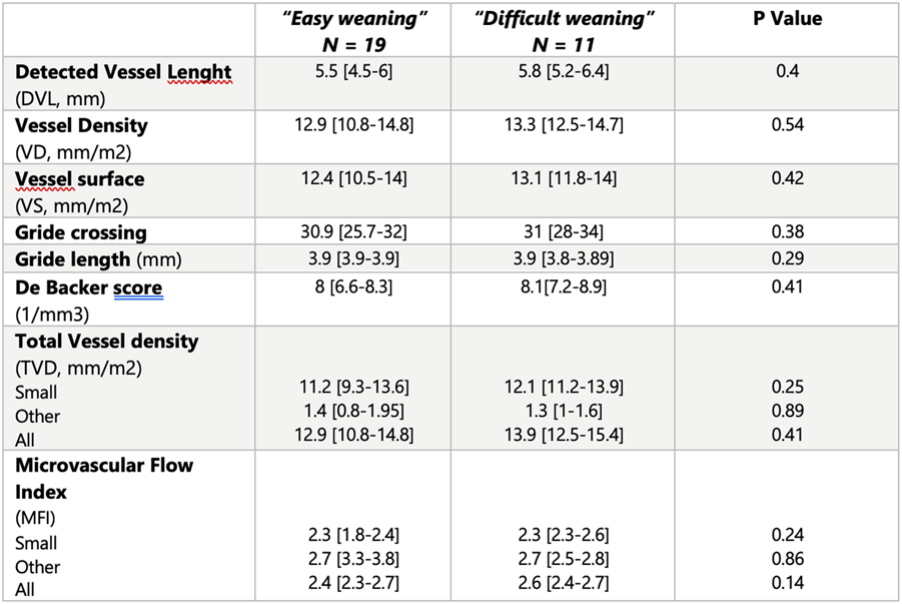


*Microcirculatory parameters after one hour of weaning test*

### CO-11 Beta-lactam exposure of children on ECMO: are conventional dosing appropriate?

#### MARSAUX Alice^1^, LÉGER Pierre-Louis^2^, BILLE Emmanuelle^3^, RENOLLEAU Sylvain^3^, TRELUYER Jean-Marc^4^, LORROT Mathie^2^, GRIMAUD Marion^3^, TOUBIANA Julie^3^, BERANGER Agathe^3^, OUALHA Mehdi^3^

##### ^1^APHP, Paris, France; ^2^Hopital Trousseau, APHP, Paris, France; ^3^Hopital Necker, APHP, Paris, France; ^4^Hopital Cochin, APHP, Paris, France

###### Correspondence: Alice MARSAUX (alice.marsaux@gmail.com)

*Annals of Intensive Care* 2022, **12(1):**CO-11

**Rationale:** Children under Extracorporeal Membrane Oxygenation (ECMO) are at high risk of complications, particularly infectious ones, which may worsen their prognosis. However, recommendations for the use of antibiotics, particularly beta-lactams dosing which are frequently administered, are extrapolated from adult data and are not adapted to the pharmacokinetic and pharmacodynamic (PK/PD) changes observed in children on ECMO. Therefore, there is a risk of inadequate exposure using the standard dosing. The objective of this study is to describe, for children undergoing ECMO, the exposure to beta-lactams at currently used dosing; and to identify factors associated with inadequate exposure.

**Patients and methods/Materials and methods:** This was an observational, retrospective, bicentric study conducted in 2 pediatric intensive care units, on 57 children undergoing veno-arterial or veno-venous ECMO. Plasma concentrations of beta-lactams used were measured and interpreted according to EUCAST, then to the minimum inhibitory concentration (MIC) found and according to the following PK/PD targets: serum concentration 4 times above the MIC throughout the dosing interval (100% fT > 4 × MIC).

**Results:** We included 57 patients (21 (36.8%) died during our study), who received 11 types of beta-lactams (including 30 patients receiving piperacillin/ tazobactam) resulting in 226 concentrations analyzed. A total of 32 infections were documented. Based on the PK/PD target of 100% fT > 4 × MIC, for the highest MICs, 58.8% of concentrations were insufficient, mainly in young children (p = 0.035) with a low bodyweight (p = 0.013), or in case of hypoalbuminemia (p = 0.011), reflecting the increase in volume of distribution, and also in case of increased renal clearance (p 0.032). The risk of supra-therapeutic concentrations was 10.5% and was mainly associated with poor elimination (presence of renal impairment (p < 0.01)).

**Conclusion:** There is a non-negligible risk of under-exposure in children receiving conventional dosing of beta-lactam and ECMO support (mainly related to renal function and volume of distribution variations). Therapeutic drug monitoring combined with the application of specific PK/PD models would reduce this risk and ultimately improve the prognosis of these patients.

**Compliance with ethics regulations:** Yes in clinical research
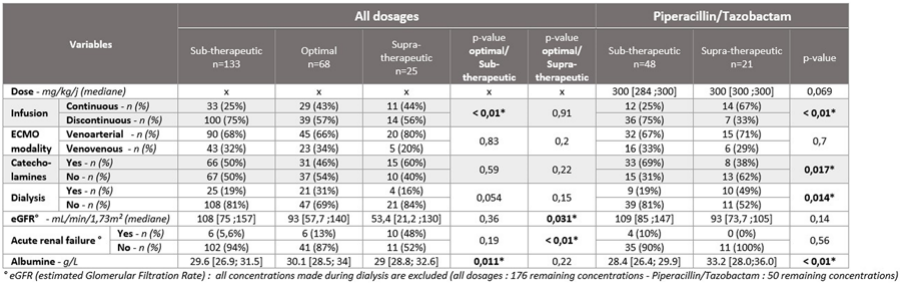


*Association between clinical variables and the result of concentrations for all dosages and Piperacillin/Tazobactam (for an exposure assumption of serum concentration 4 times above the MIC throughout the dosing interval (100% fT > 4 × MIC)*

### CO-12 Anti-infective prescription practices in children undergoing renal replacement therapy: a multicenter survey

#### THY Michael^1,3^, NAUDIN Jérôme^4^, GENUINI Mathieu^4^, LETEURTRE Stéphane^5^, OUALHA Mehdi^2,3^

##### ^1^Assistance Publique-Hôpitaux de Paris, Infectious and tropical diseases department, Bichat University Hospital, Université de Paris, Paris, France; ^2^Assistance Publique-Hôpitaux de Paris, Paediatric Intensive Care Unit, Necker-Enfants Malades University Hospital, Université de Paris, Paris, France; ^3^EA 7323 - Pharmacology and Therapeutic Evaluation in Children and Pregnant Women, Université de Paris, France; ^4^Assistance Publique-Hôpitaux de Paris, Paediatric Intensive Care Unit, Robert-Debré University Hospital, Université de Paris, Paris, France; ^5^Pediatric Intensive Care Unit, CHU Lille, Lille, France

###### Correspondence: Michael THY (michael245thy@gmail.com)

*Annals of Intensive Care* 2022, **12(1):**CO-12

**Rationale:** The need of renal replacement therapy (RRT) in septic children may occur and add variability leading to unpredictable anti-infective concentrations with risks of treatment failure, toxicity and emergence of multidrug resistant bacteria. We aim to better understand anti-infective prescription practices in children undergoing RRT.

**Patients and methods/Materials and methods:** An online survey was sent via email to physicians working in pediatric intensive care units (PICU) from the Groupe Francophone de Réanimation et d’Urgences Pédiatiques (GFRUP). The survey form assessed the characteristics of the PICU, practices of RRT, anti-infective prescription and therapeutic drug monitoring. We excluded plasma exchanges and adsorption techniques from the survey. When several respondents from the same center answered, we selected the most complete form.

**Results:** From 10/04/2021 to 10/05/2021, 47 physicians answered the survey corresponding to 26 different centers including 21 French centers, corresponding to 88% of response rate for French PICU > 4 beds. The median [IQR] number of ICU beds was 12 [8-16] and intermediate care beds was 5 [2-8]. Every PICU used continuous RRT with mainly Prismaflex^®^ machine. Adaptation of anti-infective prescriptions to the presence of RRT were declared in 23 (89%) PICU according to the molecular weight in 6 (26%), to the molecule protein binding in 6 (26%), to the lipo/hydrophilic nature of the molecule in 4 (17%), to the elimination routes in 15 (65%). The anti-infective were adapted to the residual diuresis in 9 (41%) PICU, to the RRT flow in 6 (26%) and to the type of RRT used in 15 (65%). Most of the PICU declared no available guidelines (n = 20, 80%) but used VIDAL^®^ in 12 (46%) or GPR© in 4 (15%). We noticed great variability even between the physicians from the same department. Adaptation of the anti-infective doses from the respondents are displayed on Fig. 1. Most of the centers (n = 20, 77%) used therapeutic drug monitoring under RRT and was systematically done for betalactams in 18 (69%) PICU, for aminoglycosides in 22 (92%), for glycopeptides in 21 (84%), for linezolid in 13 (54%), for antivirals in 16 (64%), for azoles 15 (60%), for candins in 12 (48%). Obstacles for monitoring were mainly (n = 11, 42%) the delay for the results and the absence of on-site laboratory (n = 8, 31%). Anti-infective stewardship was available with a specialist (pharmacologist or infectiologist) in 11 (42%) PICU.

**Conclusion:** Our survey reported great variability of anti-infective prescription practices in children undergoing RRT pointing out the need for specific guidelines.

**Compliance with ethics regulations:** Yes in clinical research.
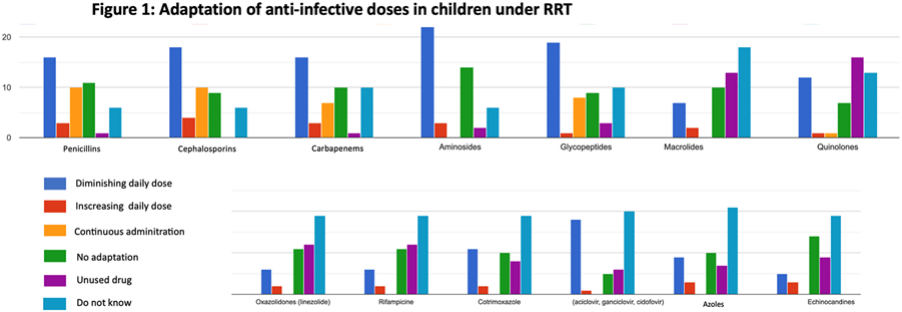


*Figure 1: Adaptation of anti-infective doses in children under RRT*

### CO-13 High-flow nasal oxygen alone or alternating with noninvasive ventilation in critically ill immunocompromised patients with acute respiratory failure. A randomised controlled trial

#### COUDROY Rémi^1^, FRAT Jean-Pierre^1^, EHRMANN Stephan^2^, PÈNE Frédéric^3^, DECAVÈLE Maxens^4^, TERZI Nicolas^5^, PRAT Gwenael^6^, GARRET Charlotte^7^, CONTOU Damien^8^, GACOUIN Arnaud^9^, BOURENNE Jérémy^10^, GIRAULT Christophe^11^, VINSONNEAU Christophe^12^, DELLAMONICA Jean^13^, LABRO Guylaine^14^, JOCHMANS Sébastien^15^, HERBLAND Alexandre^16^, QUENOT Jean-Pierre^17^, DEVAQUET Jérôme^18^, BENZEKRI Dalila^28^, VIVIER Emmanuel^19^, NSEIR Saad^20^, COLIN Gwenahel^21^, THEVENIN Didier^22^, GRASSELLI Giacomo^23^, BOUGON David^24^, ASSEFI Mona^4^, GUÉRIN Claude^25^, LHERM Thierry^26^, KOUATCHET Achille^27^, RAGOT Stéphanie^1^, THILLE Arnaud W.^1^

##### ^1^CHU de Poitiers, Poitiers, France; ^2^CHU de Tours, Tours, France; ^3^Hôpital Cochin, Paris, France; ^4^Pitié-Salpêtrière, Paris, France; ^5^CHU Grenoble Alpes, Grenoble, France; ^6^CHU de Brest, Brest, France; ^7^CHU de Nantes, Nantes, France; ^8^Centre Hospitalier Victor Dupouy, Argenteuil, France; ^9^CHU de Rennes, Rennes, France; ^10^CHU La Timone 2, Marseille, France; ^11^CHU de Rouen, Rouen, France; ^12^Centre hospitalier de Béthune, Beuvry, France; ^13^CHU de Nice, Nice, France; ^14^CHU de Besançon, Besançon, France; ^15^Centre hospitalier Sud-Ile-de France, Melun, France; ^16^Centre hospitalier Saint Louis, La Rochelle, France; ^17^CHU Dijon-Bourgogne, Dijon, France; ^18^Hôpital Foch, Suresnes, France; ^19^Hôpital Saint Joseph Saint Luc, Lyon, France; ^20^CHU de Lille, Lille, France; ^21^Centre Hospitalier Départemental de Vendée, La Roche Sur Yon, France; ^22^CH de Lens, Lens, France; ^23^Fondazione IRCCS Ca' Granda Ospedale Maggiore Policlinico, Milan, Italie; ^24^Centre Hospitalier Annecy Genevois, Annecy, France; ^25^Hôpital de La Croix-Rousse, Lyon, France; ^26^Hôpital de Chartres, Chartres, France; ^27^CHU d'Angers, Angers, France; ^28^CHR d'Orléans, Orléans, France

###### Correspondence: Rémi COUDROY (remi.coudroy@chu-poitiers.fr)

*Annals of Intensive Care* 2022, **12(1):**CO-13

**Rationale:** Whereas noninvasive ventilation is recommended for immunocompromised patients with acute respiratory failure in the intensive care unit (ICU), it may have deleterious effects in most severe patients. High-flow nasal oxygen alone may be an alternative to reduce mortality. We aimed to determine whether high-flow nasal oxygen alone could reduce the rate of mortality at day 28 compared to its alternation with noninvasive ventilation in this setting.

**Patients and methods/Materials and methods:** The FLORALI-IM is a multicenter, open-label randomised clinical trial was conducted in 30 ICUs (29 in France and one in Italy). Adult immunocompromised patients with acute respiratory failure defined as respiratory rate ≥ 25/min and a partial pressure of oxygen to inspired fraction of oxygen ≤ 300 mmHg were randomly assigned to high-flow nasal oxygen alone (n = 154) or noninvasive ventilation alternating with high-flow nasal oxygen (n = 145). The primary outcome was mortality at day 28. The trial is registered with ClinicalTrials.gov, number NCT02978300.

**Results:** Between January 2017 to March 2019, 299 patients were included in the intention-to-treat analysis. Mortality rate at day 28 was 36% (56/154) with high-flow nasal oxygen alone and 35% (51/145) with noninvasive ventilation alternating with high-flow nasal oxygen (absolute difference, 1·2% [95% CI, − 9·6 to 11·9%], p = 0·83). Intubation rate at day 28 was 51% (78/154) with high-flow nasal oxygen alone and 46% (67/145) with noninvasive ventilation alternating with high-flow nasal oxygen (difference, 4·4% [95% CI, − 6·8 to 15·5%], p = 0·44). None of the other prespecified secondary outcomes were different between groups except for greater decreased discomfort after initiation of high-flow nasal oxygen than with noninvasive ventilation (− 4 mm on visual analogic scale [95 %CI, − 18 to 4] vs. 0 mm [95% CI, − 16 to 17], p = 0·04).

**Conclusion:** In critically ill immunocompromised patients with acute respiratory failure, the mortality rate did not differ between high-flow nasal oxygen alone and noninvasive ventilation alternating with high-flow nasal oxygen.

**Compliance with ethics regulations:** Yes in clinical research.

### CO-14 Lung histopathology of Covid-19 patients with acute respiratory distress syndrome: a French multicenter study

#### MORIN Jean^1^, SAGAN Christine^1^, PLANTEFEVRE Gaetan^2^, CHELHA Riad^3^, ASFAR Pierre^4^, SIRODOT Michel^5^, SIMON Georges^6^, FERRE Alexis^7^, KAMEL Toufik^8^, PENE Frédéric^9^, DECAVELE Maxens^10^, BADIE Julio^11^, BOYER Alexandre^12^, SOUWEINE Bertrand^13^, HRAIECH Sami^14^, DELBOVE Agathe^15^, FOUCAULT Camille^16^, AUCHABIE Johann^17^, CHANAREILLE Paul-Marie^18^, ARGAUD Laurent^19^, RICHARD Jean-Christophe^20^, BEURET Pascal^21^, GARÇON Pierre^22^, HAYON Jan^23^, BONNY Vincent^24^, LEGAY François^25^, GERI Guillaume^26^, REIGNIER Jean^1^, CANET Emmanuel^1^

##### ^1^CHU de Nantes, Nantes, France; ^2^Centre Hospitalier Victor Dupouy, Argenteuil, France; ^3^Hôpital Privé Claude Galien, Quincy-Sous-Sénart, France; ^4^CHU Angers, Angers, France; ^5^Centre Hospitalier Annecy Genevois, Annecy, France; ^6^Centre Hospitalier de Troyes, Troyes, France; ^7^Hôpital de Versailles, Le Chesnay, France; ^8^CHR Orléans, Orléans, France; ^9^CHU Cochin, Paris, France; ^10^CHU Pitié Salpêtrière, Paris, France; ^11^Hôpital Nord Franche-Comté, Belfort, France; ^12^CHU de Bordeaux, Bordeaux, France; ^13^CHU Clermont-Ferrand, Clermont-Ferrand, France; ^14^APHM CHU Nord, Marseille, France; ^15^CHBA Vannes-Auray, Vannes, France; ^16^CH Cahors - Hôpital Jean Rougier, Cahors, France; ^17^CH Cholet, Cholet, France; ^18^GHPP Montélimar, Montélimar, France; ^19^CHU Edouard Herriot, Lyon, France; ^20^CHU Hôpital Lyon Sud, Lyon, France; ^21^CH de Roanne, Roanne, France; ^22^GHEF site de Marne-La-Vallée, Jossigny, France; ^23^CHI Poissy-Saint-Germain-en-Laye, Poissy, France; ^24^CHU Saint-Antoine, Paris, France; ^25^CH de Saint-Brieuc, Saint-Brieuc, France; ^26^CHU Ambroise Paré, Boulogne, France

###### Correspondence: Emmanuel CANET (emmanuel.canet@chu-nantes.fr)

*Annals of Intensive Care* 2022, **12(1):**CO-14

**Rationale:** COVID-19 is a viral pneumonia which may deteriorate into acute respiratory distress syndrome (ARDS). Analysis of the pathological features in the lung tissues of patients who have died from COVID-19-ARDS might help to understand the disease pathogenesis.

**Patients and methods/Materials and methods:** We conducted a multicenter study which involved 26 French ICUs. All Covid-19 patients who died from COVID-19-ARDS between February 1st 2020 and April 30th 2021 were eligible. The lung biopsies were performed by the intensivists immediately after the death following anatomical landmarks.

**Results:** Overall, 171 patients were included in the study. We report the results of the first 90 patients analyzed. Age was 70 [65–76] years-old, 62 (69%) patients were men, and 18 (20%) patients had a pre-existing pulmonary disease. Time from the onset of mechanical ventilation (MV) to death was 18 [10–31] days. The durations of moderate and severe ARDS were 6 [2–12] and 8 [3–15] days, respectively. The main causes of death were refractory shock and hypoxemia (42.2%), refractory hypoxemia (31.1%), and refractory shock (8.9%). Of the 90 biopsies performed, 85 (94%) had lung tissue available for pathological analysis. The most common primary lesions of the alveoli were: type-2 pneumocyte hyperplasia (84.7%), fibrin deposition (62.3%), plugs of fibroblastic tissue (36.4%), hyaline membranes (29.4%), fibrin balls (15.3%). The primary lesions identified in the septa were: proliferation of fibroblastic tissue (82.4%), lymphocytic infiltrate (20%), oedema (15.3%), and collagen deposition (8.2%). Microthrombi were identified in 7% of biopsies. Final pathological diagnosei were: late phase of proliferative diffuse alveolar damage (DAD) (38.8%), early phase of proliferative DAD (34.1%), unclassified interstitial pneumonia (29.4%), acute fibrinous and organizing pneumonia (AFOP) (12.9%), acute exsudative DAD (11.8%), and organizing pneumonia (OP) (7.1%). Early deaths (before day-10 of MV) were characterized by a high rate of acute exsudative DAD and early phase of proliferative DAD and the absence of collagen deposition. In contrast, late phase of proliferative DAD was the most common lesion encountered in late deaths (after day-30 of MV) with extensive proliferation of fibroblastic tissue. AFOP and OP were diagnosed in patients who died before day-30 of MV.

**Conclusion:** The predominant pattern of lung lesions in patients who died from Covid-19-ARDS is DAD. Fibroblastic tissue proliferation, AFOP, and OP were commonly reported while collagen fibrosis was rare. Our results support further evaluation of steroids in non-resolving ARDS Covid-19 given the significant proportion of potential steroid-sensitive patterns.

**Compliance with ethics regulations:** Yes in clinical research.

### CO-15 Management and outcomes of pregnant woman with severe pneumonia related to SARS-CoV-2 infection admitted in intensive care unit

#### PÉJU Edwige^1^, BELICARD Félicie^1^, STEIN Silva^30^, HRAIECH Sami^24^, TADIÉ Jean-Marc^7^, MULLER Grégoire^13^, THILLE Arnaud^11^, GOURY Antoine^18^, GRIMALDI David^26^, JUNG Boris^23^, PITON Gael^17^, PIAGNERELLI Michael^27^, GIBOT Sébastien^14^, THIERY Guillaume^20^, LASCARROU Jean-Baptiste^8^, BOUHEMAD Belaid^16^, ARGAUD Laurent^22^, TERZI Nicolas^31^, TAMION Fabienne^5^, EHRMANN Stephan^10^, VIGNON Philippe^19^, BELONCLE François^9^, PUGIN Jérrôme^25^, AUBRON Cécile^6^, BERTRAND Pierre-Marie^28^, GUITTON Christophe^12^, KAIDOMAR Michel^29^, MAIZEL Julien^3^, MEZIANI Ferhat^15^, SOUWEINE Bertrand^21^, JOURDAIN Merce^4^, JOZWIAK Mathieu^2^

##### ^1^Hôpital Cochin - APHP, Paris, France; ^2^CHU de Nice, Nice, France; ^3^CHU de Amiens, Amiens, France; ^4^CHU de Lille, Lille, France; ^5^CHU de Rouen, Rouen, France; ^6^CHU de Brest, Brest, France; ^7^CHU de Rennes, Rennes, France; ^8^CHU de Nantes, Nantes, France; ^9^CHU de Angers, Angers, France; ^10^CHU de Tours, Tours, France; ^11^CHU de Poitiers, Poitiers, France; ^12^Hôpital du Mans, Le Mans, France; ^13^CHR d'Orléans, Orléans, France; ^14^CHRU de Nancy, Nancy, France; ^15^CHRU de Strasbourg, Strasbourg, France; ^16^CHU de Dijon, Dijon, France; ^17^CHU de Besançon, Besançon, France; ^18^CHU de Reims, Reims, France; ^19^CHU de Limoges, Limoges, France; ^20^CHU de Saint Etienne, Saint Etienne, France; ^21^CHU de Clermond-Ferrand, Clermond-Ferrand, France; ^22^CHU de Lyon, Lyon, France; ^23^CHU de Montpellier, Montpellier, France; ^24^CHU de Marseille, Marseille, France; ^25^CHU de Genève, Genève, Suisse; ^26^Hôpital Erasme, Bruxelles, Belgique; ^27^CHU de Charleroi, Charleroi, Belgique; ^28^Hôpital de Cannes, Cannes, France; ^29^CHI de Fréjus, Fréjus, France; ^30^CHU de Toulouse, Toulouse, France; ^31^CHU de Grenoble, Grenoble, France

###### Correspondence: Edwige PÉJU (edwigepeju@gmail.com)

*Annals of Intensive Care* 2022, **12(1):**CO-15

**Rationale:** Pregnancy is a risk factor of developing a severe form of SARS-CoV-2 infection. So far, the management and outcomes of pregnant women with severe SARS-CoV-2 infection requiring intensive care unit (ICU) admission remain to be investigated.

**Patients and methods/Materials and methods:** This multicentric, retrospective and observational study was conducted in 31 ICUs in France, Belgium and Switzerland between March 2020 and December 2021. All patients admitted in ICU for severe pneumonia related to SARS-CoV-2 infection during pregnancy or just after fetal extraction were included. Maternal ventilatory and obstetrical management in ICU as well as maternal and fetal outcomes were collected and analyzed. Risk factors of intubation were also assessed.

**Results:** Overall, 165 patients were included with a median age of 34 [30–37] years old and a median gestation length of 29 [26–33] amenorrhea weeks. Among them, 61 (37%) had obesity, 10 (6%) had diabetes mellitus and 53 (32%) had pregnancy-related complications. The median time between the onset of symptoms and ICU admission was 8 [6–10] days. Corticosteroids and Tocilizumab were administered in 141 (85%) and 23 (14%) patients respectively, CT-Scan was performed in 120 (73%) patients and pulmonary embolism was found in 8 (5%) patients. Regarding ventilatory management, 107 (65%) patients were treated with high-flow nasal cannula oxygenation therapy, 40 (20%) with non-invasive ventilation, 74 (45%) required to be intubated, 20% of them prior to ICU admission, and 12 (7%) required veno-venous extracorporeal membrane oxygenation. Awake prone positioning was performed in 7 (4%) patients prior to any fetal extraction and in 2 (1%) after fetal extraction. In multivariate analysis, obesity (OR = 3.22 [1.11–9.88], p = 0.03), term (OR = 1.14 [1.05–1.27], p = 0.005), extent of CT-Scan abnormalities > 50% (OR = 3.37 [1.17–10.13], p = 0.03) and non-invasive ventilation use (OR = 3.70 [1.20–12.24], p = 0.03) were associated with intubation. Fetal extraction was performed prior to ICU admission in 19 (12%) patients and was required during ICU stay in 58 (35%) patients, always by cesarean section: 45 (78%) for maternal respiratory worsening and 7 (12%) for fetal suffering. The maternal ICU mortality rate was 1%. Main maternal complications were infection, thrombosis and hemorrhage (18%, 7%, 7%, respectively). The fetal mortality rate was 5% with 7 (4%) stillborns, 69 (40%) prematures including 55 (32%) requiring ICU admission and 103 (60%) born at term.

**Conclusion:** Despite low maternal and fetal mortality rates, half of pregnant women admitted in ICU for severe pneumonia related to SARS-CoV-2 infection required to be intubated and required fetal extraction. Risk factors of intubation were obesity, term, extent of CT-Scan abnormalities > 50% and non-invasive ventilation use. Inclusions are still in progress.

**Compliance with ethics regulations:** Yes in clinical research.

### CO-16 Impact of obesity on survival in COVID-19 ARDS patients receiving ECMO: results from an ambispective observational cohort

#### DAVIET Florence^1,3^, GUILLOUX Philippe^2^, HRAIECH Sami^1,3^, TONON David^2^, VELLY Lionel^2^, BOURENNE Jeremy^4^, PORTO Alizee^5^, GRAGUEB CHATTI Ines^1^, BOBOT Mickaël^1^, BAUMSTARCK Karine^6^, PAPAZIAN Laurent^1,3^, COLLART Frederic^5^, FOREL Jean-Marie^1,3^, GUERVILLY Christophe^1,3^

##### ^1^Assistance Publique Hôpitaux de Marseille - Hôpital Nord- Medecine Intensive reanimation, Marseille, France; ^2^Assistance Publique Hôpitaux de Marseille- Hôpital de la Timone -Département d’Anesthésie-réanimation, Marseille, France; ^3^Aix-Marseille Université, Faculté de Médecine Centre d’Études et de Recherches sur les Services de Santé et qualité de vie EA 3279, Marseille, France; ^4^Assistance Publique Hôpitaux de Marseille- Hôpital de la Timone -Réanimation des Urgences, Marseille, France; ^5^Assistance Publique Hôpitaux de Marseille- Hôpital de la Timone -Département de chirurgie cardiaque, Marseille, France; ^6^Faculté de Médecine, Centre d'Etudes et de Recherches sur les Services de Santé et Qualité de Vie EA 3279, Marseille, France

###### Correspondence: Florence DAVIET (florence.daviet@ap-hm.fr)

*Annals of Intensive Care* 2022, **12(1):**CO-16

**Rationale:** Since March 2020, healthcare systems were importantly affected by Severe Acute Respiratory Syndrome Coronavirus 2 (SARS-CoV-2) outbreak, with some patients presenting severe Acute Respiratory Distress Syndrome (ARDS), requiring extracorporeal membrane oxygenation (ECMO).

**Patients and methods/Materials and methods:** We designed an ambispective observational cohort study including all consecutive adult patients admitted to 5 different ICUs from a university hospital. The main objective was to identify the risk factors of severe COVID-19 ARDS patients supported by ECMO associated with 90-day survival.

**Results:** Between March 1st and November 30th 2020, 76 patients with severe COVID-19 ARDS were supported by ECMO. Median (interquartile range IQR) duration of mechanical ventilation (MV) prior to ECMO was of 6 (3–10) days. At ECMO initiation, patients had a median PaO_2_/FiO_2_ ratio of 71 mmHg (IQR 62–81), median PaCO_2_ of 58 mmHg (IQR 51–66) and a median arterial pH of 7.33 (IQR 7.25–7.38). Forty-five patients (59%) were weaned from ECMO. Twenty-eight day, 60-day and 90-day survival rates were respectively 92, 62 and 51%. Factors associated with 90-day survival in univariate analysis were younger age, higher BMI and lower Charlson score. Of note, time from ICU admission to intubation (2 days (0–5) in survivors versus 4 days (1–7) in non survivors, p = 0.02) and time to ECMO cannulation (9 days (4–11) in survivors versus 11 (8–14) in non survivors, p = 0.02) were shorter in 90-day survivors. In multivariate logistic regression analysis, with 2 models, one with the RESP score and one with the PRESERVE score, we found that higher BMI was associated with higher 90-day survival (Odds ratio (OR): 0.669 (0.506–0.886), p = 0.005) and 0.757 (0.632–0.907) respectively). Younger age was also associated with 90-day survival in one model (OR: 1.14 (1.007–1.292), p = 0.039). Kaplan–Meier cumulated survival curves according to the presence of obesity were significantly different (Fig. 1, p = 0.006), the survival was also different when comparing the different obesity grades (p = 0.003). Obese patients were ventilated with higher PEEP than non-obese patients (14 (10–15) vs. 10 (8–12) cm H_2_O; p < 0.001) with comparable Pplat, (29 (25–31) vs. 28 (25–30) cm H_2_O, respectively (p = 0.923)). There was a trend to higher compliance of respiratory system in obese patients as compared with non-obese patients, respectively 26.2 (21–39.1) and 23 (15.5–27.3) mL/cm H_2_O (p = 0.07).

**Conclusion:** In this ambispective observational cohort of COVID-19 severe ARDS supported by ECMO, obesity was an independent factor associated with improved survival at 90-day.

**Compliance with ethics regulations:** Yes in clinical research.
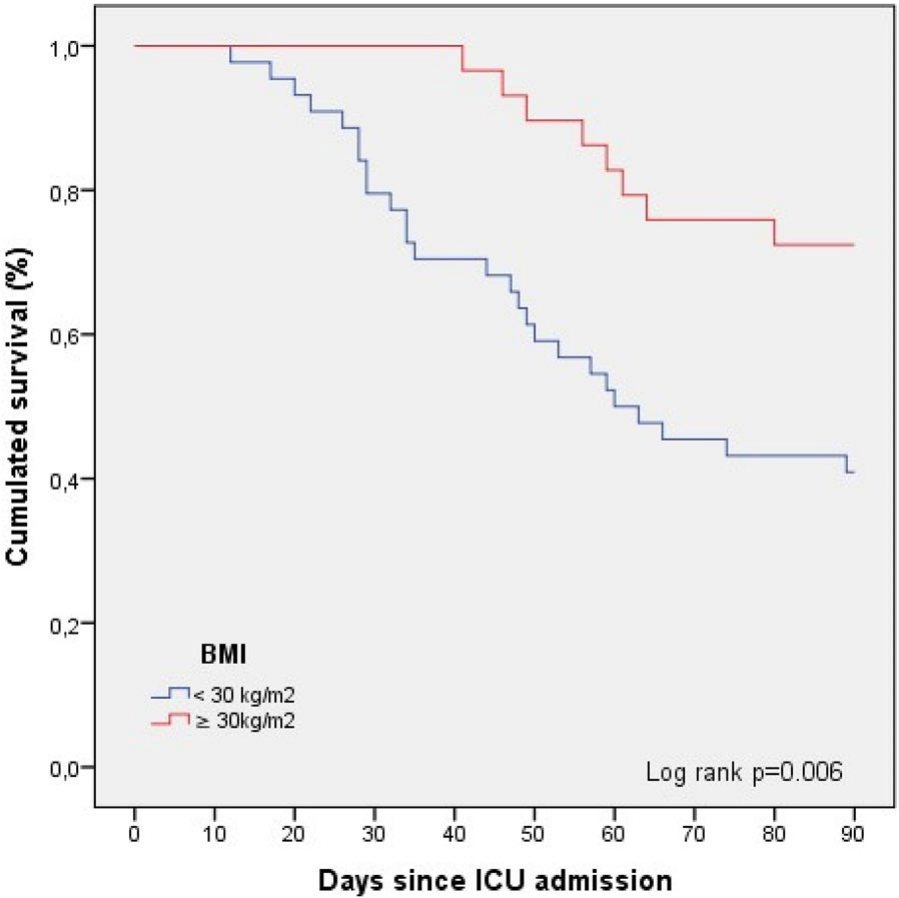


*Kaplan–Meier cumulated survival curves at day 90 since ICU admission in obese (red curve) and non-obese patients (blue curve). Obesity was defined as a BMI > 30 kg/m* ^*2*^ *.*

### CO-17 Management of acute exacerbations of chronic obstructive pulmonary disease in the intensive care unit: the Outcomerea database, 1997–2018

#### GALERNEAU Louis-Marie^1,2^, BAILLY Sébastien^1,2^, TERZI Nicolas^1,2^, RUCKLY Stéphane^3^, GARROUSTE-ORGEAS Maité^4^, COHEN Yves^5^, HONG TUAN HA Vivien^6^, GAINNIER Marc^7^, SIAMI Shidasp^8^, DUPUIS Claire^9^, DARMON Michael^10^, AZOULAY Elie^10^, FOREL Jean-Marie^11^, RIGAULT Guillaume^1^, ADRIE Christophe^12^, GOLDGRAN-TOLEDANO Dany^13^, BRUNEEL Fabrice^14^, DE MONTMOLLIN Etienne^15^, ARGAUD Laurent^16^, REIGNIER Jean^17^, PEPIN Jean-Louis^1,2^, TIMSIT Jean-François^15^

##### ^1^CHU Grenoble Alpes, La Tronche, France; ^2^Université Grenoble Alpes, INSERM U1300 HP2, La Tronche, France; ^3^Département de biostatistiques, Outcomerea, Paris, France; ^4^Hopital Franco-Britanique, Levallois-Perret, France; ^5^AP-HP, Hôpital Avicenne, Paris, France; ^6^Grand Hôpital de l'Est Francilien, Meaux, France; ^7^Hôpital de ta Timone, Marseille, France; ^8^Centre Hospitalier Sud Essonne, Etampes, France; ^9^CHU Gabriel-Montpied, Clermont-Ferrand, France; ^10^AP-HP, Hôpital Saint-Louis, Paris, France; ^11^Hôpital Universitaire Marseille Nord, Marseille, France; ^12^Hôpital Delafontaine Hôpital, Saint-Denis, France; ^13^Groupe Hospitalier Intercommunal Le Raincy Montfermeil, Montfermeil, France; ^14^Centre Hospitalier de Versailles, Le Chesnay, France; ^15^AP-HP, Hôpital Bichat, Paris, France; ^16^Hospices civils de Lyon, Hôpital Edouard Herriot, Lyon, France; ^17^CHU de Nantes, Nantes, France

###### Correspondence: Louis-Marie GALERNEAU (lmgalerneau@chu-grenoble.fr)

*Annals of Intensive Care* 2022, **12(1):**CO-17

**Rationale:** The natural history of COPD is punctuated of acute exacerbations of COPD (AECOPDs) leading to hospitalization in intensive care units (ICU) for the most severe cases. The daily practice of corticosteroids/antibiotics prescriptions and the respective proportions of invasive and non-invasive ventilation (NIV) have evolved over time in the ICU. These contextual changes might have implications regarding AECOPDs management and ICU outcomes such as length of stay, intubation rates and mortality. The aim of the current study was to assess over 22 years the evolution of AECOPDs changes in the management practices and the impact on main outcomes including length of stay and mortality in ICU and in post-ICU stay.

**Patients and methods/Materials and methods:** 1,816 patients admitted for AECOPDs were included prospectively between 1997 and 2018 in the Outcomerea French database from 32 ICUs. Evaluation of time trends were performed with analysis of the evolution of these variables over time out using a mixed model. Time series analyses were performed to assess the relationship between mortality in ICU and use of corticosteroids, antibiotics and invasive mechanical ventilation, using a dynamic regression model.

**Results:** We observed limited changes in anthropometrics of AECOPD patients admitted to ICUs except a slight increase in body mass index (+ 0.32%/year, p = .01) and severity was greater at ICU admission (+ 0.6% of SAPS II score/year, p < .01). There was overtime a significant reduction in the prescription of corticosteroids (− 4.7%/year, p < .01) and antibiotics (− 5.8%/year, p < .01), without these changes being correlated with the evolution of mortality in ICU in a time series analysis. The proportion of patients treated with invasive mechanical ventilation (IMV) also gradually declined (− 3.7%/year, p = .01) with this time a significant correlation between variations of use of IMV and variations of deaths in ICU in the time series analysis (p < .01). Rate of NIV failures decreased over time (− 6.2%/year, p < .01) with a diffusion of NIV for facilitating IMV weaning (+ 8.1%/year, p < .01). There was a significant shortening of length of stay in ICU (− 3.2%/year, p < .01) and total duration of hospital stays (− 2.6%/year, p < .01). We observed an improvement of the prognosis (− 4.1%/year of deaths in ICU, p = 0.03; − 4.2%/year of deaths in hospital post-ICU stay, p = 0.02 and − 4.98%/year of 90-day mortality, p = 0.02).

**Conclusion:** Lengths of stay decreased with a better prognosis of AECOPD in ICU. The management of ventilatory support has improved. And if prescriptions for corticosteroids and antibiotics have been reduced, prescription strategies for these therapeutics in ICU have to be precised.

**Compliance with ethics regulations:** Yes in clinical research.

### CO-18 WeanIng according to new definition: burden and long-term outcomes of WIND 2 and 3 survivors (WIND BLOWS) study

#### BEDUNEAU Gaëtan^1^, GILLIBERT André^1^, DEMOULE Alexandre^4^, RICHARD Jean Christophe^2^, MERCAT Alain^2^, BROCHARD Laurent^5^, PHAM Tài^3^

##### ^1^CHU Rouen, Rouen, France; ^2^CHU Angers, Angers, France; ^3^CHU Tenon, Paris, France; ^4^CHU La Pitié, Paris, France; ^5^Hôp Universitaire Montréal, Montréal, Canada

###### Correspondence: Gaëtan BEDUNEAU (gaetan.beduneau@chu-rouen.fr)

*Annals of Intensive Care* 2022, **12(1):**CO-18

**Rationale:** In order to analyze hospital burden and long-term outcomes of prolonged and difficult to wean patients we performed a follow-up of patients included in the WIND study.

**Patients and methods/Materials and methods:** Among the 2709 patients included in the WIND study (ref 1), 508 were classified either in group 2 (difficult weaning, n = 273) or 3 (prolonged weaning, n = 235), of whom 459 were included in France. The 339 patients of French centers alive at the end of the princeps study constituted the eligible population of our study. Each center investigator collected vital status, intercurrent hospitalization, life at home, need for oxygen therapy or home ventilation up to 8 years after the study participation.

**Results:** Data were obtained for 284 patients: 94 (33%) females, mean (standard deviation) age of 64 (14). The mean (SD) SAPS-II score at ICU admission was 50 (17) and 164 (58%) belonged to WIND group 2. We observed mortality rates of 27% at one year, 50% at 5 years and 60% at 8 years. Between 1 and 8 years, the standardized mortality ratio (SMR) compared to the general French population of the same age, sex and period distribution was estimated at 3.20 (95% CI 2.69 to 3.65). Overall mortality was higher for group 3 patients than group 2 patients (66% vs 55%, p = 0.06, Fig. 1). At 1, 5 and 8 years, respectively 88%, 89% and 90% of alive patients had no oxygen or ventilation support and 89%, 93% and 92% lived at home. The estimated cumulative risks of having at least one rehospitalization among survivors at 1, 5 and 8 years were respectively 51%, 71% and 76%.

**Conclusion:** Although the risk of death and readmission is high, especially in the first year following discharge, survivors at 8 years mostly live at home without oxygen or home ventilation.

**Reference 1:** AJRCCM Vol195, 772–783.

**Compliance with ethics regulations:** Yes in clinical research.
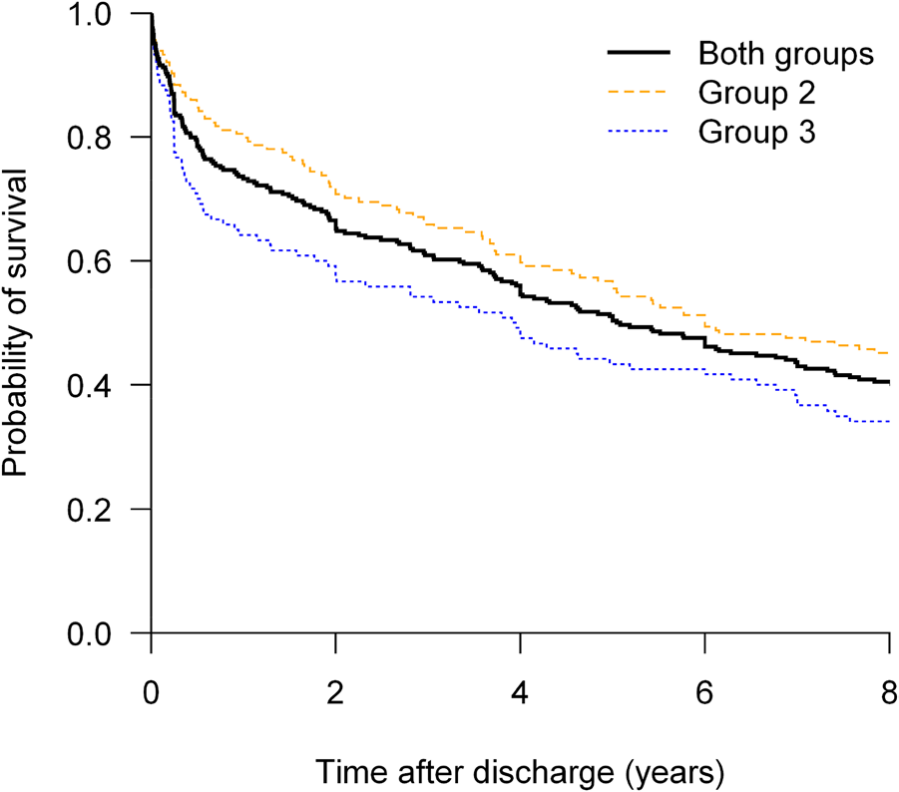


*Probability of survival*

### CO-19 HIV-infected patients hospitalized in intensive care from 1997 to 2020: analysis of the OUTCOMEREA multicenter cohort

#### GAILLET Antoine^1^, ELIE Azoulay^2^, ETIENNE Demontmollin^3^, GARROUSTE Maite^4^, COHEN Yves^5^, DUPUIS Claire^6^, SCHWEBEL Carole^7^, REIGNIER Jean^8^, SHIDASP Siami^9^, ARGAUD Laurent^10^, ADRIE Christophe^14^, MOURVILLIER Bruno^12^, RUCKLY Stephane^7^, FOREL Jean-Marie^13^, TIMSIT Jean-Francois^3^

##### ^1^Henri Mondor, Créteil, France; ^2^Saint Louis, Paris, France; ^3^Bichat, Paris, France; ^4^Saint Joseph, Paris, France; ^5^Avicenne, Bobigny, France; ^6^Gabriel Montpied, Clermont Ferrand, France; ^7^Grenoble Alpes, Grenoble, France; ^8^CHU Nantes, Nantes, France; ^9^Sud Essone, Etampes, France; ^10^Edouard Herriot, Lyon, France; ^11^Delafontaine, Saint Denis, France; ^12^CHU Reims, Reims, France; ^13^Hopital Nord, Marseille, France; ^14^Cochin, Paris, France

###### Correspondence: Antoine GAILLET (gaillet.antoine75@gmail.com)

*Annals of Intensive Care* 2022, **12(1):**CO-19

**Rationale:** Despite antiviral therapy (ARV), HIV infection is still responsible for 800,000 deaths per year. In parallel with the good virological control and the aging of this population, multiple comorbidities (HIV associated non-AIDS (HANA) conditions) have developed in this population. The objectives of the “VIHREA” study were to describe the phenotypic evolution of HIV patients admitted to intensive care unit (ICU) from 1997 to 2020 and then to identify risk factors for day-60 (D60) death.

**Patients and methods/Materials and methods:** Prospective, multicenter cohort (23 centers, OutcomeRea™), including HIV adult patients (≥ 18 years old) hospitalized in ICU from 01/01/1997 to 12/31/2020, with trend analysis through 3 periods dichotomized according to the integrase inhibitors appearance (2007) and WHO recommendation for systematic treatment of all HIV patients (2016). A survival model was used to study prognostic factors at D60.

**Results:** Of the 24,298 stays registered, 630 (2.6%) concerned first stays for a HIV patient. Throughout the 3 periods, the mean age increased (51.6 years in period 3 versus 44.2 years in period 1, p < 0.001), as did the level of comorbidity of the patients (diabetes, renal and respiratory history, solid neoplasia). The proportion of HIV discovered on ICU admission decreased significantly (9.8% versus 28.4%, p < 0.001) while the median duration of HIV disease (18 years versus 5 years, p < 0.001) as well as the percentage of patients admitted on ARV (72% versus 47.6%, p < 0.001) increased. The main reasons for admission distribution remained stable over time (acute respiratory distress > shock > coma). Moreover, while we observed a significant drop in the rate of active opportunistic infections on admission (24% versus 36.2%, p = 0.007), pneumocystosis (9.2%) and tuberculosis (6.9%) being the most frequent, the rate of active hemopathy classifying AIDS increased non-significantly (p = 0.154) but was associated with a significant increase in the administration of chemotherapy in ICU (12.7% versus 1.4%, p < 0.001), non-Hodgkin’s lymphoma being the majority (14.3%). Admissions for HANA (11.2%) or non-HIV (50.4%) reasons were stable over time, as were SOFA score on admission (p = 0.668), and the proportions of mechanically ventilated (49.4% versus 48.4%, p = 0.707) and dialysed patients (20.2% versus 14.9%, p = 0.128). In multivariate analysis, predictors of D60 mortality were advanced age, chronic liver disease, prior chemotherapy, SOFA score > 4 at admission, hospitalization time before admission to intensive care > 24 h, AIDS status, but not the period of admission.

**Conclusion:** The phenotype of HIV patients admitted to intensive care has evolved over time (HIV better controlled but more comorbidity associated). Mortality risk factors remain stable over time, including AIDS status.

**Compliance with ethics regulations:** Yes in clinical research.
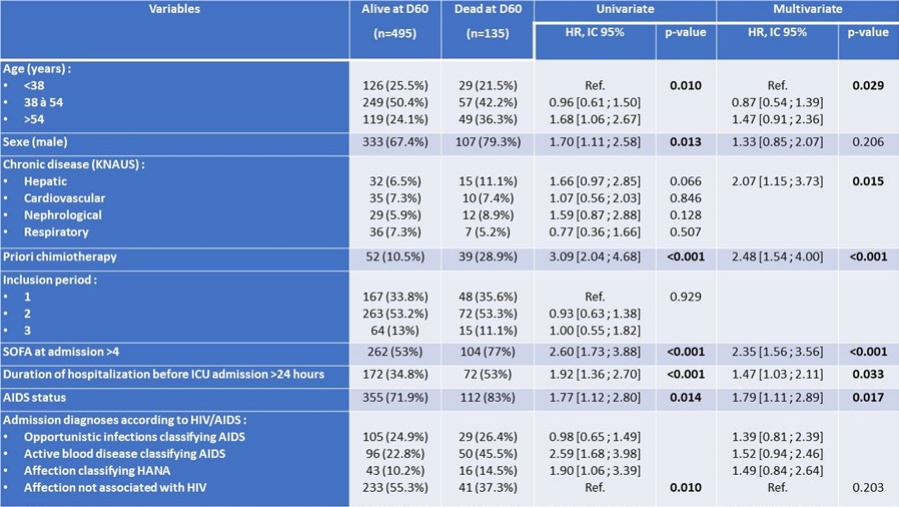


*Predictors of death at D60 after ICU admission in the VIHREA cohort (Cox regression)*

### CO-20 Necrotizing soft tissue infections in neutropenic patients requiring ICU admission: a French multicenter retrospective cohort study

#### ARRESTIER Romain^1,2,3^, CHABA Anis^4^, MABROUKI Asma^5^, SACCHERI Clément^6^, CANET Emmanuel^7^, PINETON DE CHAMBRUN Marc^8^, STOCLIN Anabelle^9^, PICARD Muriel^10^, WALLET Florent^11^, PERIER François^12^, TURPIN Matthieu^13^, ARGAUD Laurent^14^, DECAVELE Maxens^15^, ISSA Nahema^16^, CADOZ Cyril^17^, KLOUCHE Kada^18^, COHEN Johana^19^, MOKART Djamel^20^, URBINA Tomas^21^, HUA Camille^22^, CHOSIDOW Olivier^22^, MEKONTSO-DESSAP Armand^1,2,3^, AZOULAY Elie^5^, DE PROST Nicolas^1,2,3^

##### ^1^Service de Médecine Intensive et Réanimation, APHP Hôpital Henri Mondor, Créteil, France; ^2^Groupe de Recherche Clinique CARMAS, faculté de Santé de Créteil, UPEC, Créteil, France; ^3^INSERM, IMRB, Université Paris Est Créteil, Créteil, France; ^4^Service de médecine intensive et réanimation, APHP Hôpital Cochin, Paris, France; ^5^Service de médecine intensive et réanimation, APHP Hôpital Saint-Louis, Paris, France; ^6^Service de médecine intensive et réanimation, Hôpital Archet 1, Nice, France; ^7^Service de médecine intensive et réanimation, Centre Hospitalier de Nantes, Nantes, France; ^8^Service de médecine intensive et réanimation, APHP Hôpital La Pitié Salpétrière, Paris, France; ^9^Service de médecine intensive et réanimation, Institut Gustave Roussy, Villejuif, France; ^10^Service de Réanmation Polyvalente, Centre Hospitalier Universitaire de Toulouse, Oncopôle, Toulous, France; ^11^Service d'anesthésie, médecine intensive, réanimation, Hospices Civils de Lyon, Lyon, France; ^12^Réanimation Médico-Chirurgicale, Hôpital André Mignot, Versailles, France; ^13^Service de médecine intensive et réanimation, APHP, Hôpital Tenon, Paris, France; ^14^Service de médecine intensive et réanimation, Hôpital Edouard Herriot, Lyon, France; ^15^Service de Pneumologie, Médecine Intensive et Réanimation, APHP Hôpital La Pitié Salpétrière, Paris, France; ^16^Service de médecine intensive et réanimation, Hôpital Saint-André, CHU Bordeaux, Bordeaux, France; ^17^Réanimation Polyvalente, CHR Metz-Thionville, Metz, France; ^18^Service de médecine intensive et réanimation, CHU Montpellier, Montpellier, France; ^19^Service de médecine intensive et réanimation, Groupe Hospitalier Intercommunal Le Raincy Montfermeil, Montfermeil, France; ^20^Unité Traitement Soins Intensifs, Institut Paoli .Calmettes, Marseille, France; ^21^Service de médecine intensive et réanimation, APHP Hôpital Saint-Antoine, Paris, France; ^22^Service de Dermatologie, APHP Hôpital Henri Mondor, Créteil, France

###### Correspondence: Romain ARRESTIER (romain.arrestier@aphp.fr)

*Annals of Intensive Care* 2022, **12(1):**CO-20

**Rationale:** Necrotizing soft tissue infections (NSTIs) are rare life-threatening bacterial infections characterized by an extensive necrosis of skin and subcutaneous tissues. Immunocompromised patients have an increased risk of dying. Among them, neutropenic patients could have a particularly high risk of poor outcome but data are scarce. Our objectives are to describe the characteristics and the mortality of neutropenic patients with NSTI requiring intensive care unit (ICU) admission.

**Patients and methods/Materials and methods:** We conducted a retrospective multicenter cohort study in 18 French ICUs. Patients admitted in participating ICUs between 2011 and 2021 with either surgically confirmed NSTI or clinically suspected diagnosis of NSTI and neutropenia < 1.5 G/L at diagnosis were included. Characteristics and hospital mortality of patients with neutropenic NSTI were compared to a historical cohort of ICU patients with non-neutropenic NSTI. The factors associated with hospital mortality were identified using Cox regression.

**Results:** 83 neutropenic patients were included and compared to 129 historical non-neutropenic patients. Neutropenia was related to haematological malignancy in 64 (77.1%) patients and to solid cancer in 10 (12%) patients. Median time from neutropenia onset to NSTI was 4 [1-14] days. Other patients’ characteristics are described in Table 1. As compared to non-neutropenic patients, neutropenic patients were younger (65 [56–75] vs 58 [46.5–64] years, p < 0.001), had less frequent lower limb (70.3% vs 42.2%, p < 0.01) and more abdomino-perineal NSTIs (25% vs 42.2%, p = 0.014). Neutropenic patients presented more frequent bacteraemia (58.5% vs 26.4%, p < 0.001). The most frequent bacteria isolated in neutropenic patients were *Enterobacteriaceae* (42.2%) and *Pseudomonas aeruginosa* (30.1%). In contrast, non-neutropenic patients were more frequently infected by *Streptococcus* species than neutropenic patients (68.2% (n = 88) vs 2.4% (n = 2), p < 0.001). Time from NSTI diagnosis to first surgery was shorter in non-neutropenic patients than in others (0 [0–0] days vs 1 [1–2] days, p < 0.001). 28-day and in-hospital mortality rates were significantly higher in neutropenic than in non-neutropenic patients (48.2% vs 25.6%, p = 0.001, and 54.2% vs 35.7%, p = 0.012). In multivariate analysis, factors associated with hospital mortality for neutropenic patients were age (adjusted Hazard Ratio (aHR) = 1.04 (1.02–1.06), p = 0.001) and SOFA score (aHR = 1.16 (1.08–1.25, p < 0.001), while treatment with G-CSF (aHR = 0.34 (0.17–0.66), p = 0.002) was protective.

**Conclusion:** NSTI in neutropenic patients presented different characteristics and higher mortality than in non-neutropenic patients requiring ICU. Treatment with G-CSF could be associated with better outcomes, although this finding should be interpreted with caution due to the presence of potential unadjusted confounding factors.

**Reference 1:** Urbina, T. et al. Early identification of patients at high risk of group A streptococcus-associated necrotizing skin and soft tissue infections: a retrospective cohort study. Crit Care 23, 417 (2019).

**Compliance with ethics regulations:** Yes in clinical research
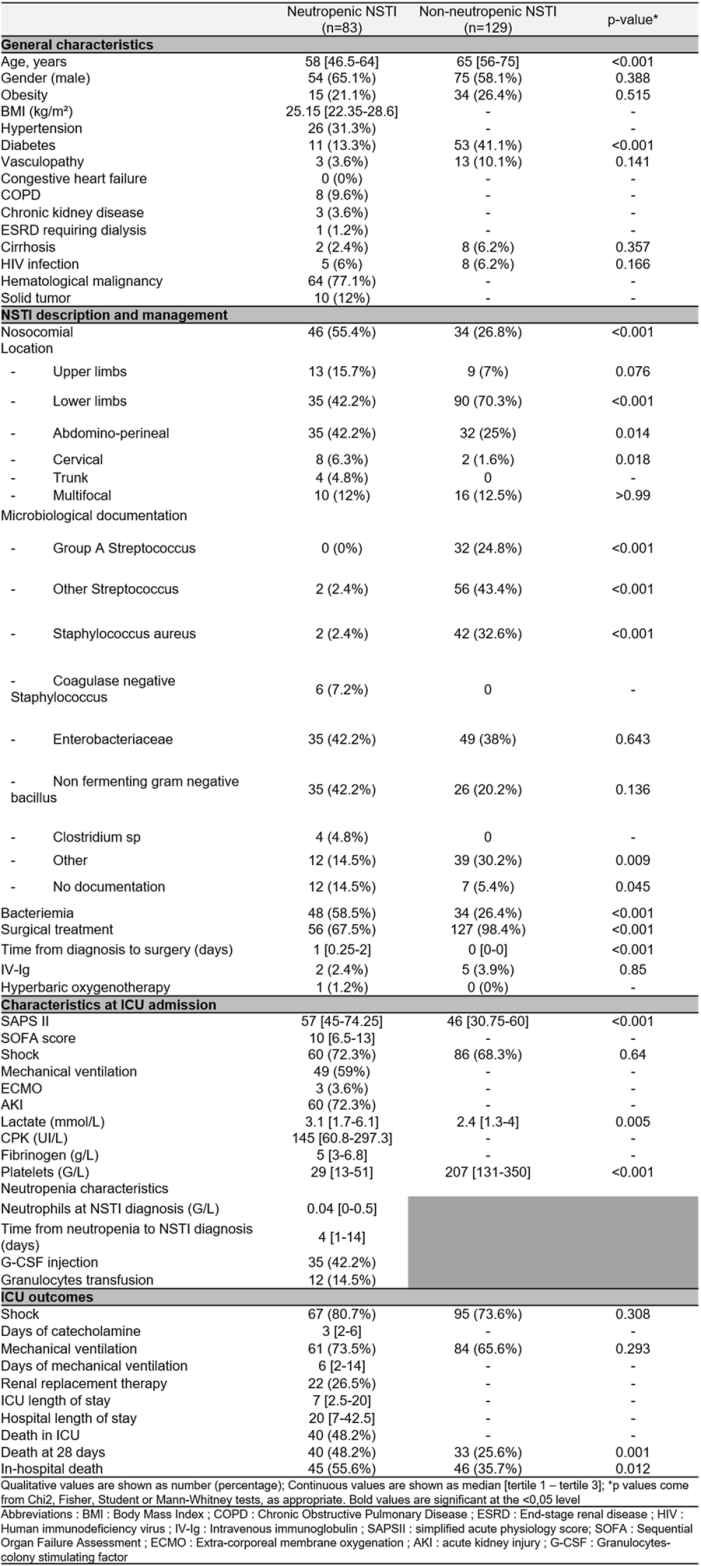


*Characteristics and ICU outcomes of neutropenic and non-neutropenic patients*

### CO-21 Critically ill patients with infective endocarditis, neurological failure and indication for cardiac surgery: a multicenter propensity-adjusted study

#### GROS Alexandre^1^, GUETTARD Yves Olivier^1^, SEGUY Benjamin^1^, PILLOIS Xavier^1^, PREVEL Renaud^1^, ORIEUX Arthur^1^, PREAU Sebastien^2^, LAVIE-BADIE Yoan^3^, COUPEZ Elisabeth^7^, COUDROY Remi^4^, MAREST Delphine^5^, MARTINS Raphael^6^, HIKARU Fukutomi^1^, GRUSON Didier^1^, TOURDIAS Thomas^1^, BOYER Alexandre^1^

##### ^1^CHU Bordeaux, Bordeaux, France; ^2^CHU Lille, Lille, France; ^3^CHU Toulouse, Toulouse, France; ^4^CHU Poitiers, Poitiers, France; ^5^CHU Nantes, Nantes, France; ^6^CHU Rennes, Rennes, France; ^7^CHU Clermont-Ferrand, Clermont-Ferrand, France

###### Correspondence: Alexandre BOYER (alexandre.boyer@chu-bordeaux.fr)

*Annals of Intensive Care* 2022, **12(1):**CO-21

**Rationale:** The benefit-risk balance and/or optimal timing of surgery for infective endocarditis (IE) with neurological events (ischemic or haemorrhagic stroke) remain debated, especially in critically ill patients. Our hypothesis is that these patients should still benefit from surgery. The main objective of this study was to compare their neurological functional outcome whether they received surgery or not.

**Patients and methods/Materials and methods:** We conducted a retrospective (2010–2017), French multi-center study of critically ill patients with acute IE. They were included if they met the following criteria: (i) left-sided IE according to the modified Duke criteria, (ii) with cerebral complications documented by cerebral imaging before cardiac surgery and (iii) with Sequential Organ Failure Assessment score (SOFA) ≥ 3; one of these justifying admission to ICU. Exclusion criteria were isolated right endocarditis, in-hospital acquired endocarditis and patients who developed cerebral complications only after cardiac surgery. All patients were explored with brain imaging before surgery. The primary analysis consisted in a propensity score adjusted logistic regression of surgery as an independent variable of the primary outcome, i.e. modified Rankin score (mRS) at 6 months. The 1-year mortality rate was the main secondary outcome.

**Results:** 192 patients were included whom 67 patients had medical treatment (35%) and 125 underwent cardiac surgery (65%). Delayed surgery according to theoretical recommended timing was decided in 62/125 (50%). Ischemic stroke was the most prevalent neurological complications (74.5%) followed by hemorrhagic lesion (15.6%). In the propensity score-adjusted logistic regression, the odds ratio (OR) for favorable 6-month functional outcome was 0.08 (95% CI 0.03–0.13) in favor of surgery. One-year mortality was strongly reduced with surgery in the fixed-effect propensity adjusted cox model (Hazard Ratio 0.23; 95% CI 0.14–0.36; p < 0.001). These effects remained whether the patients received delayed surgery or not and whether they were deeply comatose or not (Glasgow score > 10).

**Conclusion:** In this study of critically ill patients with simultaneous infectious endocarditis, indication for surgery and cerebral events, a better propensity-adjusted functional outcome was associated with surgery compared with medical treatment, whatever the deepness of coma. A delayed surgery should be considered in severe acute regurgitations. In the absence of randomized study precluded for ethical concerns, an individualized strategy remains highly suggested.

**Compliance with ethics regulations:** Yes in clinical research.

### CO-22 Predicting central venous catheter-tip colonization in the ICU: development and validation of a score in several randomized trials

#### IACHKINE Jeanne^1^, BUETTI Niccolò^2^, BRIANT Anaïs^1^, MIMOZ Olivier^3^, MÉGARBANE Bruno^5^, MIRA Jean-Paul^6^, RUCKLY Stéphane^8^, SOUWEINE Bertrand^4^, DU CHEYRON Damien^1^, TIMSIT Jean-François^7^, PARIENTI Jean-Jacques^1^

##### ^1^CHU Caen, Caen, France; ^2^Hôpitaux universitaires de Genève, Genève, Suisse; ^3^CHU de Poitiers, Poitiers, France; ^4^CHU de Clermont-Ferrand, Clermont-Ferrand, France; ^5^Hôpital Lariboisière AP-HP, Paris, France; ^6^Hôpital Cochin AP-HP, Paris, France; ^7^Hôpital Bichat AP-HP, Paris, France; ^8^Université de Paris, Paris, France

###### Correspondence: Jeanne IACHKINE (jeanne.iachkine@gmail.com)

*Annals of Intensive Care* 2022, **12(1):**CO-22

**Rationale:** Catheter-related bloodstream infection (CRBSI) requires urgent removal of the central venous catheter (CVC). However, the majority of CVCs removed because of a suspicion of CRBSI proves sterile. We aimed to develop and validate a score predictive of catheter-tip colonization with pathogens other than Coagulase-negative Staphylococcus (CoNS).

**Patients and methods/Materials and methods:** We conducted a retrospective study based on databases from two multicenter randomized controlled trials investigating the effect of the catheter insertion site on intravascular complications (training cohort). Catheter colonization was defined as growth of > = 10^3^ colony-forming units per milliliter from the catheter tip culture yielding to pathogens other than skin contaminants according to Brun-Buisson quantitative technique. Potential factors associated with catheter colonization were identified using a generalized linear model with binomial distribution to account for several CVCs per patients, in univariate and multivariate analyses. Internal validation of these risk factors was performed using bootstrap with 500 replications. Then, a score was computed from the adjusted Odds Ratio coefficients. Finally, external validation was performed in three other independent RCTs investigating the effect of different prevention strategies on the incidence of catheter-related infections (validation cohort). Discrimination was assessed by the Area Under the Curve (c-index).

**Results:** Among 3,681 CVCs and dialysis catheters included in the training cohort, 357 (9.7%) were colonized with microorganisms other than CoNS. Age, obesity, diabetes, site of insertion (jugular and femoral versus sub-clavian), type of catheter (dialysis versus CVC), catheterization duration, fever and local inflammation at removal were independently associated with colonization in multivariate analysis. Diabetes, site of insertion, type of catheter, catheterization duration, fever and local inflammation at removal were robust after internal validation and were computed in the score. Area under the ROC curve for the score was 0.70, 95% CI [0.68–0.73] in the training cohort (Hosmer and Lemeshow goodness of fit test: p = 0.94). The validating cohort included 6,299 dialysis catheters and CVCs, of which 262 (4.2%) were colonized. AUC for the score was 0.65, 95% CI [0.62-0.69] in the validation cohort (Hosmer and Lemeshow goodness of fit test: p = 0.18) (see Fig. 1). Among 1340 catheters removed for suspicion of CRBSI infection in the training and validating cohorts, 1168 (87.2%) were sterile.

**Conclusion:** This score had a moderate ability to discriminate central venous catheter colonization. Further research is needed to assess the clinical utility of this score for managing CVCs in the ICU, in particular to help clinicians’ decision facing a catheter-related infection suspicion.

**Compliance with ethics regulations:** Yes in clinical research.
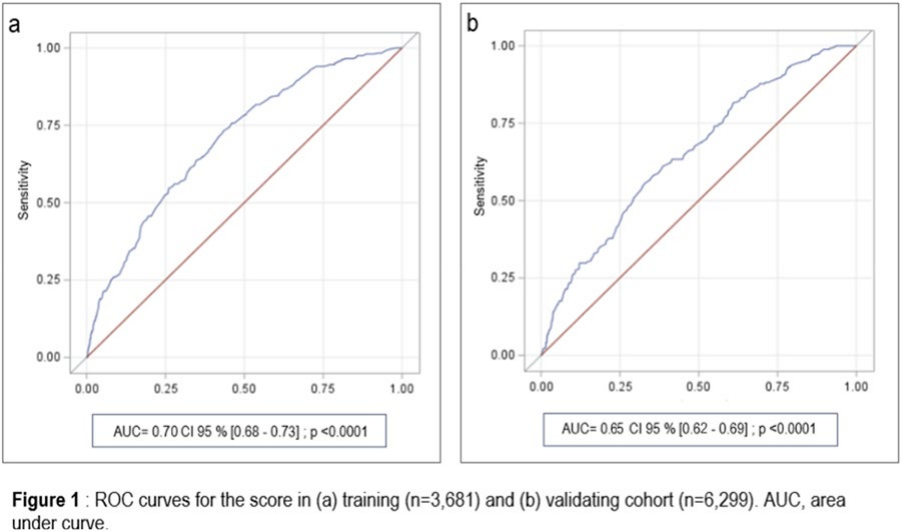


*Roc curves for the scores in (a) training (b) cohorts. AUC, area under the curve.*

### CO-23 Efficacy of carbapenem vs carbapenem-sparing therapy as empiric antimicrobial therapy in patients with extended-spectrum Beta-lactamase-producing Enterobacteriales urinary septic shock: a propensity-weighted multicenter cohort study (SCRUTIN study)

#### CARIOU Erwann^1^, PREVEL Renaud^1^, SILVA Stein^2^, FAGUER Stanislas^2^, SEGUIN Thierry^2^, NSEIR Saadalla^3^, CANET Emmanuel^4^, DESCLAUX Arnaud^1^, SOUWEINE Bertrand^6^, KLOUCHE Kada^5^, GUISSET Olivier^1^, PILLOT Jerome^7^, PICARD Walter^8^, SAGHI Tahar^9^, DELOBEL Pierre^2^, GRUSON Didier^1^, BOYER Alexandre^1^

##### ^1^CHU de Bordeaux, Bordeaux, France; ^2^CHU de Toulouse, Toulouse, France; ^3^CHU de Lille, Lille, France; ^4^CHU de Nantes, Nantes, France; ^5^CHU de Montpellier, Montpellier, France; ^6^CHU de Clermont Ferrand, Clermont Ferrand, France; ^7^Ch de Bayonne, Bayonne, France; ^8^CH de Pau, Pau, France; ^9^Polyclinique Bordeaux Nord Aquitaine, Bordeaux, France

###### Correspondence: Erwann CARIOU (erwann.ca@gmail.com)

*Annals of Intensive Care* 2022, **12(1):**CO-23

**Rationale:** Extended-spectrum beta-lactamase-producing Enterobacteriales (ESBL-E) is a serious health threat with $1.2 billion estimated attributable US healthcare costs in 2017. Their dissemination has led to a major increase in the use of carbapenems, last-resort antibiotics. The objective of this study was to assess the efficacy of carbapenem-sparing regimen as empiric therapy in extended-spectrum β-lactamase-producing Enterobacteriales (ESBL-E) urinary septic shock.

**Patients and methods/Materials and methods:** This retrospective propensity-weighted multicenter observational study conducted in 11 ICUs compared the outcomes of ESBL-E urinary septic shock patients treated with carbapenem or carbapenem-sparing regimen as empiric treatment (piperacillin–tazobactam (PTZ) + aminoglycosides or 3rd generation cephalosporin (3GC) + aminoglycosides). The primary outcome was Day-30 mortality. Secondary outcomes included in-ICU and Day-90 mortality rates, severity of illness, septic shock resolution, ICU and in-hospital length of stay and *Clostridium difficile* infection.

**Results:** Among 156 patients included in the study, 69 received a carbapenem and 87 received non-carbapenem antibiotics as empiric treatment. Baseline clinical characteristics were similar between the 2 groups. Patients who received an empiric carbapenem-sparing therapy had similar Day-30 mortality (OR = 0.462 [0.173; 1.775] p = 0,143), illness severity, resolution of septic shock and ESBL-E infection recurrence rates than patients who received carbapenem. The rate of secondary infection with *C. difficile* was comparable.

**Conclusion:** In ESBL-E urinary septic shock, empiric treatment with carbapenem-sparing regimen was not associated with higher mortality, compared to a carbapenem regimen.

**Reference 1:** Harris PNA, Tambyah PA, Lye DC, et al. Effect of piperacillin-tazobactam vs meropenem on 30-day mortality for patients with *E coli* or *Klebsiella pneumoniae* bloodstream infection and ceftriaxone resistance: a randomized clinical trial. JAMA 2018; 320:984–9.

**Reference 2:** Karaiskos I, Giamarellou H. Carbapenem-sparing strategies for ESBL producers: when and how. Antibiot Basel Switz 2020; 9:E61.

**Compliance with ethics regulations:** Yes in clinical research
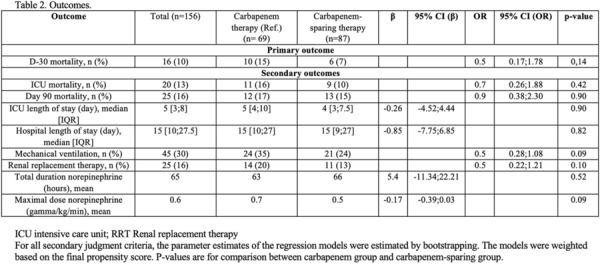


*Outcomes.*

### CO-24 Intravenous Interleukin-7 to restore absolute lymphocyte counts in patients with sepsis

#### DAIX Thomas^1^, JEANNET Robin^1^, MATHONNET Armelle^2^, MIRA Jean-Paul^3^, DEQUIN Pierre-François^4^, MORRE Michel^5^, WALTON Andrew^6^, HOTCHKISS Richard^6^, FRANÇOIS Bruno^1^

##### ^1^CHU Dupuytren, Limoges, France; ^2^CHR Orléans, Orléans, France; ^3^Hôpital Cochin, AP-HP, Paris, France; ^4^CHRU de Tours, Tours, France; ^5^Revimmune, Inc., Bethesda, Md, Etats-Unis; ^6^Washington University School of Medicine, St Louis, Mo, Etats-Unis

###### Correspondence: Thomas DAIX (thomas.daix@chu-limoges.fr)

*Annals of Intensive Care* 2022, **12(1):**CO-24

**Rationale:** Sepsis leads to deep apoptosis-induced depletion of T-cells resulting in increased rate of secondary infection and late morbidity (1). Interleukin-7 (IL-7) is a pluripotent cytokine essential for lymphocyte proliferation and survival. By intra-muscular (IM) or subcutaneous route, IL-7 improved immunity by increasing T-cell count and reversed the marked loss of immune effector cells in lymphopenic patients with septic shock (2). Because IL-7 by IM route led to injection site reactions due to local lymphocytic infiltration, we designed a phase II study evaluating efficacy and safety of a potentially better tolerated route (intravenous).

**Patients and methods/Materials and methods:** Phase II prospective, randomized, double blind, placebo-controlled trial in 8 ICUs in France and 2 in the United States. Immunocompetent patients with vasopressor-dependent sepsis, two successive absolute lymphocyte counts (ALC) < 900 cells/mm^3^ and acute respiratory failure and/or acute kidney injury at 48 h after ICU admission were eligible. Forty patients were planned to be randomized 3:1 to receive IV administration of recombinant IL-7 (CYT107) at 10 µg/kg or placebo twice a week for 3 weeks. Primary objective was to determine if IV IL-7 induces an increased ALC at day 29 or hospital discharge. Secondary objective was to assess the safety and tolerability of CYT107.

**Results:** Between June 2019 and March 2020 21 patients (63.8 ± 15.8 y.o.; SOFA score = 8.8 ± 4.4, APACHE II = 17.5 ± 5.6) received CYT107 (n = 15) or placebo (n = 6). ALC was similar in both group at baseline (0.73 ± 0.38 G/L vs 0.70 ± 0.57 G/L). CYT107 tended to increase the ALC at day 29 or hospital discharge (1.71 ± 1.2 G/L in treatment groups vs 1.18 ± 0.79 G/L in control group; p = 0.17) (Figure). Patients in the treatment group had more organ support-free days (22.13 vs 7.7 days; p = 0.01) and slightly less secondary infections (26.7% vs 33%). Three patients developed fever, tachypnea and evidence of hypercytokinemia associated with IL-7 administration, which necessitated discontinuing therapy. Following the review of these events by the Data Safety Monitoring Board, a pharmacokinetic control was carried out and evidenced a higher concentration of CYT107 than expected (Cmax at 41,423 ± 8,548 ng/ml and an AUC at 273,772 ± 107,786 pg/ml⋅h) at time of adverse event.

**Conclusion:** While IV injection of IL-7 showed ability to reverse sepsis-related lymphopenia, safety issues related to this route led to favor IM or subcutaneous route for the next phase III trial.

**Reference 1:** Drewry AM, Samra N, Skrupky LP, Fuller BM, Compton SM, Hotchkiss RS. Persistent lymphopenia after diagnosis of sepsis predicts mortality. Shock. 2014;42(5):383–391.

**Reference 2:** Francois B, Jeannet R, Daix T, et al. Interleukin-7 restores lymphocytes in septic shock: the IRIS-7 randomized clinical trial. JCI Insight. 2018;3(5):e98960.

**Compliance with ethics regulations:** Yes in clinical research.
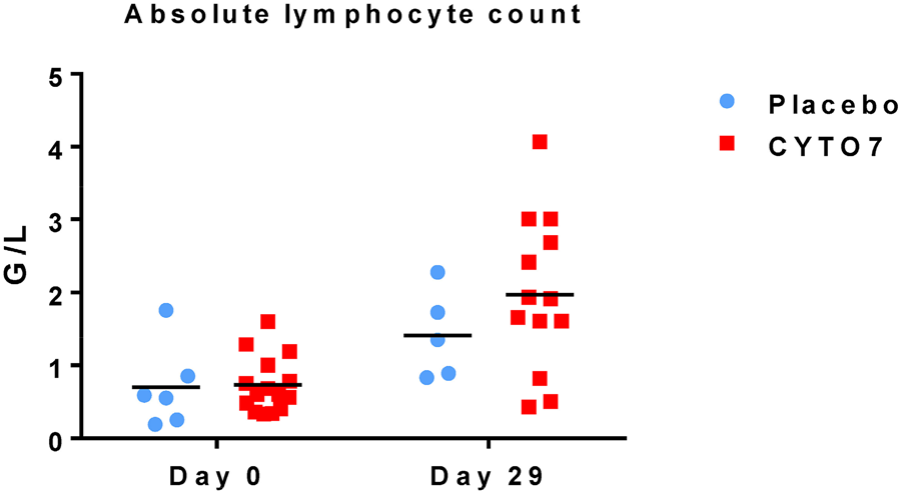


*Absolute Lymphocyte Count (ALC) at day 0 and day 29 (or hospital discharge). Blue dots represent placebo-treated patients and red squares CYT107-treated patients.*

### CO-25 Intravenous immunoglobulins in patients with COVID-19-associated moderate-to-severe acute respiratory distress syndrome (ICAR): multicentre, double-blind, placebo-controlled, phase 3 trial

#### MAZERAUD Aurélien^1^, JAMME Matthieu^2^, LUCAS Bruno^3^, SYLLA Khaoussou^1^, SHARSHAR Tarek^1^

##### ^1^GHU Paris Psychiatrie et Neurosciences, Paris, France; ^2^CH Poissy Saint Germain en Laye, Poissy, France; ^3^Institut Cochin, INSERM, Paris, France

###### Correspondence: Aurélien MAZERAUD (aurelien.mazeraud@gmail.com)

*Annals of Intensive Care* 2022, **12(1):**CO-25

**Rationale:** Acute respiratory distress syndrome (ARDS) is a major complication of COVID-19 and is associated with high mortality and morbidity. We aimed to assess whether intravenous immunoglobulins (IVIG) could improve outcomes by reducing inflammation-mediated lung injury.

**Patients and methods/Materials and methods:** In this multicentre, double-blind, placebo-controlled trial, done at 43 centers in France, we randomly assigned patients (1:1) receiving invasive mechanical ventilation for up to 72 h with PCR confirmed COVID-19 and associated moderate-to-severe ARDS to receive either IVIG (2 g/kg over 4 days) or placebo. Random assignment was done with a web-based system and was stratified according to the participating center and the duration of invasive mechanical ventilation before inclusion in the trial (< 12 h, 12–24 h, and > 24–72 h), and treatment was administered within the first 96 h of invasive mechanical ventilation. To minimize the risk of adverse events, the IVIG administration was divided into four perfusions of 0·5 g/kg each administered over at least 8 h. Patients in the placebo group received an equivalent volume of sodium chloride 0·9% (10 mL/kg) over the same period. The primary outcome was the number of ventilation-free days by day 28, assessed according to the intention-to-treat principle. This trial was registered on ClinicalTrials.gov, NCT04350580.

**Results:** Between April 3, and October 20, 2020, 146 patients (43 [29%] women) were eligible for inclusion and randomly assigned: 69 (47%) patients to the IVIG group and 77 (53%) to the placebo group. The intention-to-treat analysis showed no statistical difference in the median number of ventilation-free days at day 28 between the IVIG group (0·0 [IQR 0·0–8·0]) and the placebo group (0·0 [0·0–6·0]; difference estimate 0·0 [0·0–0·0]; p = 0·21). Serious adverse events were more frequent in the IVIG group (78 events in 22 [32%] patients) than in the placebo group (47 events in 15 [20%] patients; p = 0·089).

**Conclusion:** In patients with COVID-19 who received invasive mechanical ventilation for moderate-to-severe ARDS, IVIG did not improve clinical outcomes at day 28 and tended to be associated with an increased frequency of serious adverse events, although not significant. The effect of IVIGs on earlier disease stages of COVID-19 should be assessed in future trials.

**Compliance with ethics regulations:** Yes in clinical research.

### CO-26 Unsupervised clustering in critically ill COVID-19 patients: corticosteroid therapy may not fit for all

#### ZERBIB Yoann^1^, CARPENTIER Mathieu^1^, RICHECOEUR Jack^2^, TAMION Fabienne^3^, CUGNART Cécile^3^, BONEF Olivier^4^, VANDERBECKEN Carla^1^, MICHAUD Audrey^1^, KONTAR Loay^1^, SLAMA Michel^1^, BRAULT Clément^1^, MAIZEL Julien^1^

##### ^1^CHU Amiens PICARDIE, Amiens, France; ^2^CH Simone Veil, Beauvais, France; ^3^CHU Rouen, Rouen, France; ^4^CH Saint Quentin, Saint Quentin, France

###### Correspondence: Yoann ZERBIB (zerbib.yoann@chu-amiens.fr)

*Annals of Intensive Care* 2022, **12(1):**CO-26

**Rationale:** Since the beginning of the COVID-19 pandemic, corticosteroid therapy has become a standard treatment for critically ill patients. The aim of this study was to identity COVID-19 clusters and investigate therapeutic response among clusters.

**Patients and methods/Materials and methods:** We performed a multicenter observational retrospective study between March 2020 and December 2021. A hierarchical clustering on principal components (HCPC) was used to establish COVID-19 profiles based on standard laboratory tests. Demographic data, ICU management, mortality and therapeutic response were compared between clusters.

**Results:** During the period, 329 patients were included in the analysis. HCPC could identified 2 clusters. Patients of Cluster 2 were older (69 [61, 74] vs 64 [55, 71], p = 0.001) and had more comorbidities (3.50 [2, 5] vs 2 [1, 4], p = < 0.001). These patients had a more severe organ failure. Requirement of invasive mechanical ventilation (69.9% (n = 58) vs 56.5% (n = 139); p = 0.033) and renal replacement therapy (39.8% (n = 33) vs 14,2% (n = 31), p < 0.001) were more frequent. In line with this, in-ICU mortality rate was 44.6% (n = 37) while patients of Cluster 1 died in 27,2% of cases (n = 67) (p = 0,004). Corticosteroid therapy was associated with better outcome among patients of Cluster 1 (Relative Risk = 0.66, 95% CI [0,45–1,001], p = 0.06 and Log Rank test = 0,035) while it was not associated with better survival among patients of Cluster 2 (Relative Risk = 1.51, 95% CI [0,94–2,2], p = 0.12 and Log Rank test = 0,12)

**Conclusion:** Based on HCPC, we could identify 2 distinct profiles with different severity and different outcome. More importantly, corticosteroid therapy was associated with a better outcome only in the less severe patients. This approach might help clinician to undertake personalized ICU management.

**Compliance with ethics regulations:** Yes in clinical research.

### CO-27 Relationship between corticosteroid adjuvant therapy and incidence of ventilator-associated lower respiratory tract infections: a planned ancillary analysis of the coVAPid cohort

#### SAURA Ouriel^1^, ROUZÉ Anahita^1^, POVOA Pedro^3^, MARTIN-LOECHES Ignacio^2^, MAKRIS Demosthenes^4^, ARTIGAS Antonio^5^, LABREUCHE Julien^1^, NSEIR Saad^1^

##### ^1^CHU Lille, Lille, France; ^2^St James Hospital, Dublin, Irlande; ^3^Hospital de Sao Francisco Xavier, Lisbone, Portugal; ^4^University Hospital of Larissa, Larissa, Grece; ^5^Corporacion Sanitaria Universitaria Parc Tauli, CIBER Enferme dades Respiratorias, Sabadell, Espagne

###### Correspondence: Ouriel SAURA (ouriel.saura@gmail.com)

*Annals of Intensive Care* 2022, **12(1):**CO-27

**Rationale:** Ventilator-associated lower respiratory tract infection (VA-LRTI) is a common and severe complication in patients admitted to the intensive care unit (ICU) for SARS-CoV-2 pneumonia. Corticosteroids are widely used to treat these patients although the impact of this adjuvant therapy on the incidence of VA-LRTI in this population is still unclear. We sought to determine the relationship between corticosteroid adjuvant therapy and the incidence of VA-LRTI in a large cohort of COVID-19 critically ill patients.

**Patients and methods/Materials and methods:** This is a planned ancillary analysis of the retrospective multicenter coVAPid study. Adult patients invasively ventilated for more than 48 h for a SARS-CoV-2 pneumonia during the first epidemic surge were consecutively included. VA-LRTI diagnosis required strict definition with clinical, radiological and microbiological documentation. We assessed the association between VA-LRTI and corticosteroid adjuvant therapy using univariate and multivariate cause-specific Cox’s proportional hazard models with adjustment on prespecified confounders and with time stratification.

**Results:** 545 patients were included, 191 (35%) received corticosteroids. The incidence of VA-LRTI was not significantly different in the corticosteroid-treated versus non-treated patients (p = 0.068 for the likelihood ratio in adjusted model). We found a significant time varying effect of corticosteroid on the incidence of VA-LRTI (p = 0.018), with a non-significant increased risk of developing VA-LRTI after the 14th day following intubation (adjusted cHR 1.38 (95% CI 0.99 to 1.92)).

**Conclusion:** We found no significant association between corticosteroid adjuvant therapy and the incidence of VA-LRTI in a cohort of COVID-19 critically ill patients. We demonstrated a significant time-varying effect of corticosteroids on the risk of developing VA-LRTI all along the 28-day follow-up.

**Compliance with ethics regulations:** Yes in clinical research.

### CO-28 Impact of corticosteroid therapy on the incidence of ventilator-associated pneumonia in patients with COVID-19: retrospective multicenter observational study

#### FORT Romain^1^, VACHERON Charles Herve^1^, BOHE Julien^1^, RICHARD Jean Christophe^2^, RIMMELE Thomas^2^, LUKASZEWICZ Anne Claire^2^, ARGAUD Laurent^2^, DAILLER Frederic^2^, AUBRUN Frederic^2^, FELLAHI Jean Luc ^2^, ALLAOUCHICHE Bernard^1^, BESTION Audrey^2^, HODILLE Elisabeth^2^, LEVRARD Melanie^1^, GERBAUD COULAS Chloe^1^, JOFFREDO Emilie^1^, THIOLLIERE Fabrice^1^, VASSAL Olivia^1^, DE SEISSAN DE MARIGNAN Donatien^1^, JAY Lucille^1^, PIRIOU Vincent^1^, FRIGGERI Arnaud^1^, WALLET Florent^1^

##### ^1^CHLS - HCL, Pierre-Bénite, France; ^2^HCL, Lyon, Gambie

###### Correspondence: Romain FORT (romain.fort@chu-lyon.fr)

*Annals of Intensive Care* 2022, **12(1):**CO-28

**Rationale:** Several epidemic reports indicated a high incidence of ventilator-associated pneumonia (VAP) in patients with severe SARS-CoV2 pneumonia (1). Considering the common use of dexamethasone (DXM) since the 2nd wave, we conducted a large retrospective multicenter study to evaluate the influence of DXM exposure on the incidence of VAP.

**Patients and methods/Materials and methods:** 2088 patients with a diagnosis of COVID-19 admitted to the intensive care unit (ICU) between 27/02/20 and 30/05/21 were included. Among them 1080 (42.2%) had mechanical ventilation (MV) > 48 h, and thus separated into 2 groups according to exposure or not to DXM (protocol defined in the RECOVERY trial): DXM+ (n = 751) and DXM− (n = 329). The diagnosis of VAP was based on clinical and microbiological arguments.

**Results:** The 2 groups were similar. Majority of patients were on MV within the first 24 h after admission; however, with a longer median time from admission to intubation in the DXM+ group (1 [0–3] vs 0 [0–1]; p < 0.001). Patients exposed to DXM had also a longer duration of intubation: 15 days [8–28] vs 13 days [4–25], p = 0.003. The univariate analysis found a higher proportion of VAP in the DXM+ group (58.7%) compared with the DXM− group (51%) (p = 0.023). The overall incidence rate of VAP episodes was essentially the same in the 2 groups: 50.4‰ ventilator days [46.9–54.2] in the DXM+ group vs 49‰ [43.4–55.1] in the other group. Considering competing factors such as extubation and death, the cumulative incidence of the first episode of VAP was higher in the DXM+ group (p = 0.006) with respectively at 7 days: 37.1% [33.7–40.6] vs. 26.4% [21.7–31.2]; at 14 days: 52.7% [49.2–56.2] vs. 43.5% [38.2–48.7]; and at 28 days: 57.8% [54.3–61.2] vs. 49.9% [44.6–55.1] (Figure). But in multivariate analysis, DXM was not identified as an independent risk factor for VAP: HR = 1.08 [0.89–1.30]; p = 0.444. The microbiological distribution was approximately the same in both groups, most often with polymicrobial documentation represented essentially by gram-negative bacteria (GNB) with an overrepresentation of enterobacteria (45%) and non-fermenting GNB (20%). There was also no difference in terms of mortality.

**Discussion:** The incidence of VAP in our series is close to that found in Rouzé et al. study (2) of 1576 patients with the same diagnostic criteria for VAP.

**Conclusion:** In this large multicenter cohort of patients treated for severe SARS-CoV2 infection, there is no association between the incidence of VAP and the use of DXM.

**Reference 1:** Yang X, Yu Y, Xu J, et al.: Clinical course and outcomes of critically ill patients with SARS-CoV-2 pneumonia in Wuhan, China: a single-centered, retrospective, observational study. The Lancet Respiratory Medicine 2020; 8:475–481.

**Reference 2:** Rouzé A, Martin-Loeches I, et al.: Relationship between SARS-CoV-2 infection and the incidence of ventilator-associated lower respiratory tract infections: a European multicenter cohort study. Intensive Care Medicine 2021; 47:188–198.

**Compliance with ethics regulations:** Yes in clinical research.
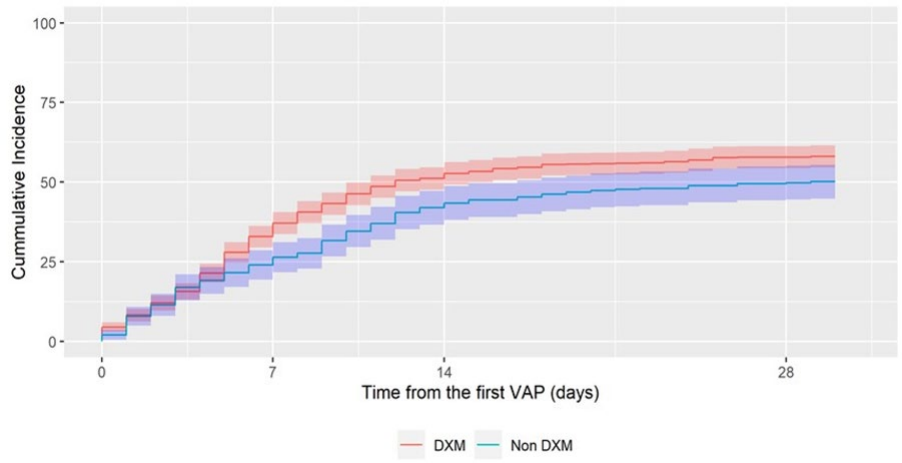


*Cumulative incidence curve of the first episode of VAP in patients exposed and not exposed to DXM*

### CO-29 Citrulline administration in COVID-19 associated ARDS patients: a randomized controlled trial

#### REIZINE Florian^1^, LESOUHAITIER Mathieu^1^, GREGOIRE Murielle^1^, DULONG Joelle^1^, LAUNEY Yoann^1^, LEBOUBIER Thomas^1^, BENDAVID Claude^1^, HAMON Catherine^1^, LE PABIC Estelle^1^, GACOUIN Arnaud^1^, MAAMAR Adel^1^, PAINVIN Benoit^1^, ROUSSEL Mikael^1^, PRONIER Charlotte^1^, LE TULZO Yves^1^, LAVIOLLE Bruno^1^, COGNE Michel^1^, TARTE Karin^1^, TADIÉ Jean-Marc^1^

##### ^1^CHU de Rennes, Rennes, France

###### Correspondence: Florian REIZINE (florian.reizine@gmail.com)

*Annals of Intensive Care* 2022, **12(1):**CO-29

**Rationale:** COVID-19 induces a sustained immunosuppression responsible for secondary infections acquisition and late mortality. We recently demonstrated that COVID-19 was responsible for T-cells dysfunction through arginine depletion. Several studies have found that supplementation with citrulline, which is converted in arginine through the activity of argininosuccinate synthetase and argininosuccinate lyase, was more efficient than arginine to increase plasma level of arginine. In the present study, we aimed to assess the effect of citrulline supplementation in COVID-19-associated Acute Respiratory Distress Syndrome (ARDS) patients as an adjuvant therapy with the goal to relieve immunosuppression and help virus clearance.

**Patients and methods/Materials and methods:** We performed a prospective randomized controlled trial. Patients with RT-PCR confirmed COVID-19 associated ARDS were randomized to receive enteral citrulline supplementation 7 g/day for 7 days or placebo. The primary endpoint was SOFA score on day 7. Secondary outcomes included SOFA score on day 14, Intensive Care Unit (ICU) length of stay (LOS), Day-28 mortality and SARS-CoV2 PCR positivity in tracheal aspirates at day 14 and plasma arginine concentration at day 7.

**Results:** A total of 32 patients were included (10 women, median age 66 years). SOFA score at day 7 were not statistically different between the two groups (4 (IQR: 3–6) versus 3.5 (3–4); p = 0.8) nor was the SOFA score at day 14 despite a trend towards a diminished SOFA score in the citrulline group (3(2.5–6.5) versus 2 (1–5); p = 0.068). Interestingly, a lower proportion of SARS-COV2 PCR positive respiratory samples was observed at day 14 in citrulline patients (30.8% versus 75%; p = 0.027). ICU LOS did not differ between the two groups (18.5 (13–28) versus 15.5 (10.5–25.5); p = 0.32. Arginine concentrations in patients treated by citrulline were higher at day 7 (51.9 (44.5–70.4) versus 77.7 (56.1–102.6), p = 0.026). Finally, Day-28, mortality was 0/16 in the citrulline group and 2/16 in the placebo group, log-rank test p = 0.2.

**Conclusion:** In this proof-of-concept study, citrulline supplementation among COVID-19 ARDS patients did not improve day-7 SOFA score. The higher viral clearance promoted by such treatment deserves further investigations.

**Compliance with ethics regulations:** Yes in clinical research.

### CO-30 Decontamination regimen in COVID-19 ICU patients

#### MASSART Nicolas^1^, REIZINE Florian^2^, FILLATRE Pierre^1^, SEGUIN Philippe^2^, LACOMBE Béatrice^4^, FREROU Aurélien^5^, EGRETEAU Pierre-Yves^6^, HOURMANT Baptiste^7^, KERGOAT Pierre^8^, LORBER Julien^9^, SOUCHARD Jerome^2^, CANET Emmanuel^10^, RIEUL Guillaume^3^, FEDUN Yannick^3^, DELBOVE Agathe^3^, CAMUS Christophe^2^

##### ^1^CH Saint-Brieuc, Saint-Brieuc, France; ^2^CHU de Rennes, Rennes, France; ^3^CH de Vannes, Vannes, France; ^4^CH Bretagne SUD, Lorient, France; ^5^Centre Hospitalier de Saint-Malo, Saint-Malo, France; ^6^Centre Hospitalier de Morlaix, Morlaix, France; ^7^CHU de Brest, Brest, France; ^8^CH de QUIMPER, Quimper, France; ^9^CH de Saint-Nazaire, Saint-Nazaire, France; ^10^CHU de Nantes, Nantes, France

###### Correspondence: Nicola MASSART (nicolasmassart@hotmail.fr)

*Annals of Intensive Care* 2022, **12(1):**CO-30

**Rationale:** Critically ill patients admitted with SARS-COV 2 infectious disease (COVID-19) are at high risk of ventilator-associated infection (VAP) and ICU acquired bloodstream infection (BSI) with implications for outcomes. Among strategies that aimed to prevent both such acquired infections (AI), selective decontamination regimen has been poorly studied in COVID-19 setting.

**Patients and methods/Materials and methods:** We performed an ancillary analysis of the COCOREVAP study which is a multicenter retrospective observational study in 15 ICUs in western France. All adults admitted with COVID-19 from February 1st, 2020 until December 31th 2021 who required mechanical ventilation were eligible. In addition to standard care, 3 ICUs used a multiple-site decontamination regimen (MSD), a variant of selective digestive decontamination, which consists of the administration of topical antibiotics including an aminoglycoside (tobramycin or gentamicin), polymyxin and amphotericin B, four times daily in the oropharynx and the gastric tube, chlorhexidine body washing and a 5-day nasal mupirocin course in patients who had an expected intubation duration of 24 h or more. AI and death risk factors were estimated using logistic regression.

**Results:** During study period, 614 of 1158 COVID-19 patients admitted in our ICUs were intubated for at least 48 h. Due to missing data regarding AIs in 153 patients, 461 patients were finally included. Baseline characteristics in both groups were similar at the exception of a lower age (62 years [55–71] vs 68 [61–73] p = 0.002), a lower body mass index (27.46 [24.39–31.40] vs 28.76 [25.38–32.16] p = 0.026) and a lower proportion of patients admitted during fall 2020 (31.5% vs 48.1% p < 0.001) in the MSD group. Compared with the standard-care group, AI were less frequent in the MSD group with incidence rates of 30.6 per 1000 patients-days and 16.1 per 1000 patients-days (Incidence Rate Ratio = 0.53 95% CI [0.37–0.75] p < 0.001) respectively. In multivariate analysis, MSD administration was associated with a lower risk of AI (OR = 0.36, 95% CI [0.21–0.63]; p < 0.001). Regarding AI site, MSD remained independently associated with a significant lower risk of VAP (OR = 0.35, 95% CI [0.20–0.60]; p < 0.001) but not of BSI (OR = 0.54, 95% CI [0.57–1.31], p = 0.17). Hospital mortality was lower among patients receiving MSD (30.1% vs 16.9% p = 0.017) (Figure), with an independent protective effect (OR = 0.49, 95% CI [0.24–0.99]; p = 0.049).

**Conclusion:** MSD was associated with a lower incidence of AI among ventilated COVID-19 patients. These promising results deserve confirmation by randomized controlled trials.

**Compliance with ethics regulations:** Yes in clinical research.
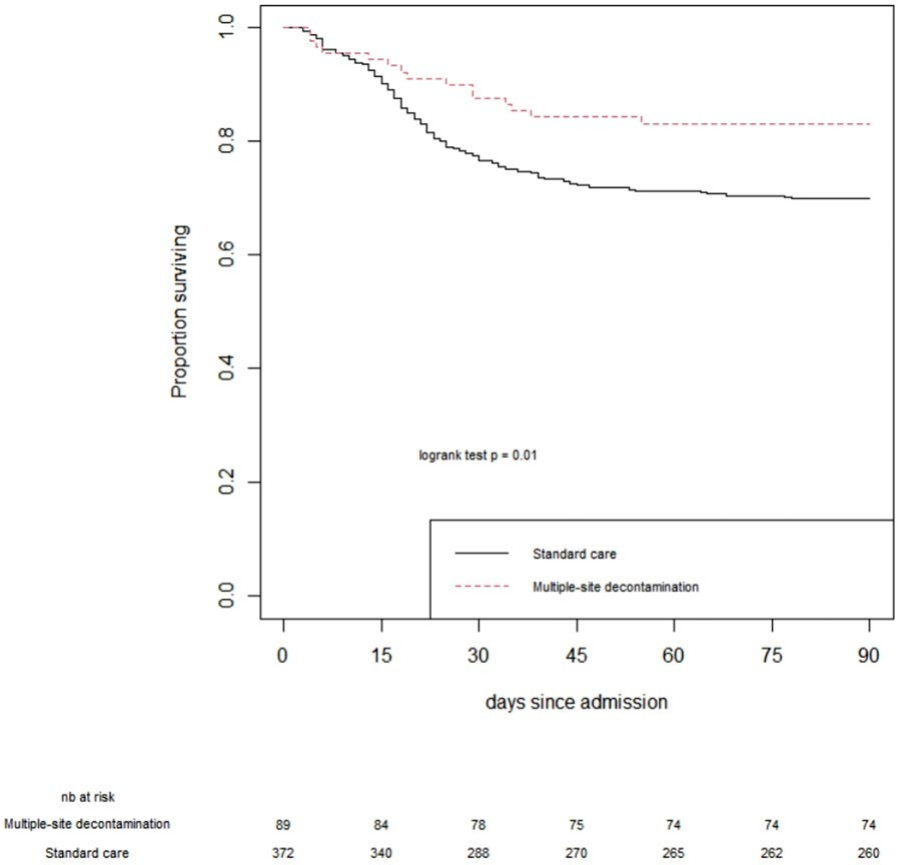


*Figure. Survival curves*

### CO-31 The association of induced hypothermia at 33 °C and controlled normothermia at 37 °C with outcome in patients with shock on ICU admission after cardiac arrest in non-shockable rhythm: a post-hoc analysis of the HYPERION trial

#### ZIRIAT Ines^1^, LE THUAUT Aurélie^1^, LASCARROU Jean-Baptiste^1^

##### ^1^CHU de Nantes, Nantes, France

**Correspondence:** Ines ZIRIAT (ziriat.inesnaila@gmail.com)

*Annals of Intensive Care* 2022, **12(1):**CO-31

**Rationale:** Last consensus of ERC-ESICM on temperature management indicates there was insufficient evidence to recommend for or against temperature control at 32–36 °C or early cooling after cardiac arrest. Post-resuscitation shock defined as need for vasopressors after return of spontaneous circulation is associated with high mortality and brain damage. A post-hoc analysis of TTM2 trial indicated a safety signal about higher mortality when 33 °C was chosen as target during TTM. We perform a post-hoc analysis of HYPERION trial to explore interaction between post-resuscitation shock status and temperature targeted after cardiac arrest.

**Patients and methods/Materials and methods:** Post-analysis of HYPERION trial which include patients with ROSC after cardiac arrest in non-shockable rhythm. Patients were divided according to presence or absence of post-resuscitation shock after cardiac arrest. Impact of induced hypothermia (33 °C) or controlled normothermia (37 °C) on favorable functional outcome (defined as Cerebral Performance Category (CPC) 1 or 2 at day 90) was evaluated.

**Results:** We included, 581 patients: 339 with post-resuscitation shock and 242 without. Of patient with post-resuscitation shock, 159 received induced hypothermia and 180 controlled normothermia. On day 90, 14 of the 159 patients in the hypothermia group had a CPC score of 1 or 2, as compared with 10 of the 180 patients in the normothermia group, with no significant difference (8.81% vs 5.56%, p = 0.24) (Table 3). Patients in the hypothermia group and the normothermia groups did not differ in terms of mortality on day 90 (83% vs 86%, p = 0.43, Table 4, HR 0.92, 95% CI 0.73-1.16, p = 0.46, Fig. 2).

**Conclusion:** Presence of a post-resuscitation shock at ICU admission after cardiac arrest in non-shockable rhythm is a major determinant of day-90 functional outcome. There is no interaction between post-resuscitation shock presence and benefits of induced hypothermia provided for patients with cardiac arrest in non-shockable rhythm as compared to controlled normothermia.

**Compliance with ethics regulations:** Yes in clinical research.

### CO-32 Organ donation after out-of-hospital cardiac arrest: a population-based study

#### RENAUDIER Marie^1,2^, BINOIS Yannick^1,2^, DUMAS Florence^1,2^, LAMHAUT Lionel^2,3^, BEGANTON Frankie^2^, JOST Daniel^2,4^, CHARPENTIER Julien^1^, LESIEUR Olivier^5^, MARIJON Eloi^2,6^, JOUVEN Xavier^2,6^, CARIOU Alain^1,2^, BOUGOUIN Wulfran^2,7^

##### ^1^Hôpital Cochin, Paris, France; ^2^Paris Sudden Death Expertise Center, Paris, France; ^3^Hôpital Necker Enfants-Malades, Paris, France; ^4^Brigade des Sapeurs Pompiers de Paris, Paris, France; ^5^Hôpital Saint-Louis, La Rochelle, France; ^6^Hôpital Européen Georges Pompidou, Paris, France; ^7^Hôpital Privé Jacques Cartier, Massy, France

###### Correspondence: Marie RENAUDIER (Mrenaudier@aol.com)

*Annals of Intensive Care* 2022, **12(1):**CO-32

**Rationale:** Organ shortage is a major public health issue, and patients who die after out-of-hospital cardiac arrest (OHCA) could be a valuable source of organs. Our objective was to identify factors associated with organ donation after brain death complicating OHCA, in unselected patients entered into a comprehensive real-life registry covering a well-defined geographic area.

**Patients and methods/Materials and methods:** We prospectively analyzed consecutive adults with OHCA who were successfully resuscitated but died in intensive care units in the Paris region in 2011–2018. The primary outcome was organ donation after brain death. Independent risk factors were identified using logistic regression analysis. A donation-likelihood score was established. One-year outcomes of transplants and transplant recipients were assessed using Cox and log-rank tests.

**Results:** Of the 3061 included patients, 136 (4.4%) became organ donors after brain death, i.e. 28% of the patients with brain death. Patients characteristics are described in the Table. An interaction between admission pH and post-resuscitation shock was identified. By multivariate analysis, in patients with post-resuscitation shock, predictors of organ donation were neurological cause of OHCA (odds ratio [OR], 14.5 [7.6–27.4], P < 0.001), higher pH (OR/0.1 increase, 1.3 [1.1–1.6], P < 0.001); older age predicted absence of donation (OR/10-year increase, 0.7 [0.6–0.8], P < 0.001). In patients without post-resuscitation shock, the only predictor was neurological cause of OHCA (OR, 6.9 [3.0–15.9], P < 0.001); higher pH (OR/0.1 increase, 0.8 [0.7–1.0], P = 0.04) and OHCA at home (OR, 0.4 [0.2–0.7], P = 0.006) predicted absence of donation. Organ donation occurred in 3% of patients with a prediction score < 0 and 34% of those with a score > 0. One-year outcomes of kidney transplants and their recipients did not differ according to Utstein characteristics of the donor.

**Conclusion:** Organ donation should be considered in every patient with OHCA due to a neurological cause, independently from presence of post-resuscitation shock and from Utstein characteristics.

**Compliance with ethics regulations:** Yes in clinical research.
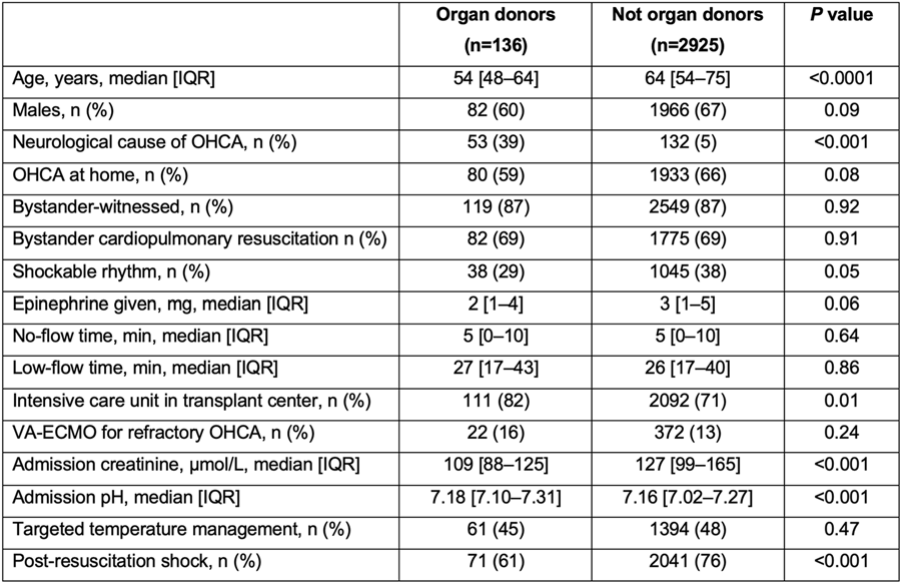


*Table: Utstein characteristics of patients with out-of-hospital cardiac arrest*

### CO-33 Factors associated with circulatory death after cardiac arrest: a population-based, clustering analysis

#### BINOIS Yannick^1,7^, RENAUDIER Marie^1,7^, DUMAS Florence^3,7,8^, YOUSSFI Younès^8,9^, BEGANTON Frankie^7,8^, JOST Daniel^5,7,8^, LAMHAUT Lionel^6,7,8^, MARIJON Eloi^4,7,8^, JOUVEN Xavier^4,7,8^, CARIOU Alain^1,7,8^, BOUGOUIN Wulfran^2,7^

##### ^1^Hôpital Cochin, ICU department, Paris, France; ^2^Hôpital Jacques Cartier, ICU department, Massy, France; ^3^Hôpital Cochin, Emergency department, Paris, France; ^4^Hôpital Georges Pompidou, Cardiology department, Paris, France; ^5^BSPP Paris, Paris, France; ^6^Hôpital Necker Enfants malades, ICU department, Paris, France; ^7^Université de Paris, INSERM U970 - PARCC, Paris, France; ^8^Paris Sudden Death Expertise Center, Paris, France; ^9^Center for Research in Economics and Statistics, Palaiseau, France

###### Correspondence: Yannick BINOIS (yannick.binois@gmail.com)

*Annals of Intensive Care* 2022, **12(1):**CO-33

**Rationale:** Out-of-hospital cardiac arrest (OHCA) is a common cause of death, with a very low survival rate. Early circulatory failure is the most common reason for death within the first 48 h after resuscitation. This study including intensive care unit (ICU) patients with OHCA was designed to identify and characterize clusters based on clinical and laboratory features and to determine the frequency of death from refractory post-resuscitation shock (RPRS) in each cluster.

**Patients and methods/Materials and methods:** We retrospectively identified adults who were admitted alive to ICUs after OHCA in 2011–2018 and recorded in a prospectively established registry for the Paris area (France). We identified patient clusters by performing an unsupervised hierarchical cluster analysis (without mode of death among the variables) based on Utstein clinical and laboratory variables. For each cluster, we used the Fine-and-Gray approach to estimate the hazard ratio (HRs) for RPRS (defined as post-resuscitation shock refractory to aggressive critical care). Inclusion was at ICU admission. The time-to-event analysis was censored on the date of death in the ICU or date of ICU discharge alive.

**Results:** Of the 4445 included patients, 1468 (33%) were discharged alive from the ICU and 2977 (67%) died in the ICU. We identified four clusters: initial shockable rhythm with short low-flow time (cluster 1), initial non-shockable rhythm with usual absence of ST-segment elevation (cluster 2), initial non-shockable rhythm with long no-flow time (cluster 3), and long low-flow time with high epinephrine dose (cluster 4). RPRS was significantly associated with this last cluster (HR, 5.51; 95% confidence interval, 4.51–6.74).

**Conclusion:** We identified patient clusters based on Utstein criteria, and one cluster was strongly associated with RPRS. This result may help to make decisions about using specific treatments after OHCA.

**Compliance with ethics regulations:** Yes in clinical research.
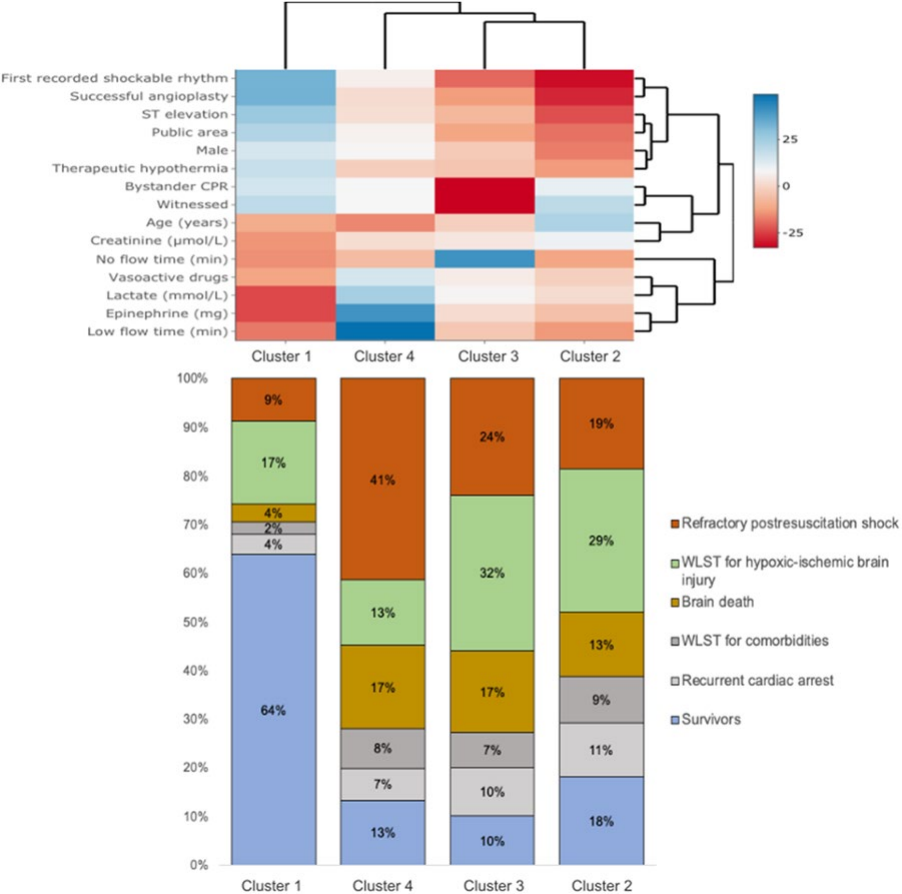


*Heatmap* + *PCA*

### CO-34 Effect of moderate hypothermia vs normothermia on 30-day mortality in patients with cardiogenic shock receiving venoarterial extracorporeal membrane oxygenation: a randomized clinical trial

#### LEVY Bruno^1^, GIRERD Nicolas^1^, OUATTARA Alexandre^2^, COMBES Alain^3^

##### ^1^CHU Nancy, Vandoeuvre Les Nancy, France; ^2^CHU Bordeaux, Department Of Anaesthesia And Critical Care, Magellan Medico-Surgical Centre, France; ^3^APHP, Paris La Pitie Salpetriere, Mir, France

###### Correspondence: Bruno LEVY (blevy@sfr.fr)

*Annals of Intensive Care* 2022, **12(1):**CO-34

**Rationale:** The optimal approach to the use of venoarterial extracorporeal membrane oxygenation (VA-ECMO) during cardiogenic shock is uncertain.

**Patients and methods/Materials and methods:** To determine whether early use of moderate hypothermia (33–34 °C) compared to strict normothermia (36–37 °C) improves mortality in patients with cardiogenic shock receiving VA-ECMO. Multicenter, unblinded, parallel-group, randomized clinical trial in the intensive care units (ICU) of 20 French Cardiac Shock Care Centers Patients were eligible if they were endotracheally intubated and had been receiving VA-ECMO for cardiogenic shock for < 6 h. Of 786 eligible patients, 374 were randomized. The primary outcome was mortality at 30 days. There were 21 secondary outcomes including mortality at days 7, 60, and 180; a composite outcome of death, cardiac transplant, stroke or escalation to left ventricular assist device (LVAD) at days 30, 60 and 180, ventilatory- and kidney replacement therapy-free days between inclusion and days 30, 60 and 180. Adverse events included rates of severe bleeding, sepsis and number of packed red blood cells transfused during VA-ECMO.

**Results:** 334 patients completed the trial (mean age, 57 [SD 12] years. At 30 days, 71 of the 168 patients (42%) in the moderate hypothermia group and 84 of the 166 patients (51%) in the normothermia group had died (adjusted odds-ratio = 0.71; 95% confidence interval 0.45–1.13, p = 0.15; risk difference -8.3% (− 16.3 to − 0.3%)). The odds-ratio of the composite outcome of death, cardiac transplant, escalation to LVAD and stroke in the hypothermia group, as compared with the control group, was 0.61 (95% CI 0.39–0.96, p = 0.03) at day 30 (risk difference − 11.5% (− 23.2 to 0.2%)). Of the 27 secondary outcomes, 26 were inconclusive. The incidence of severe or moderate bleeding was 34% in the hypothermia group and 36% in the normothermia group. The incidence of infections was 52% in both groups.

**Conclusion:** In patients with refractory cardiogenic shock treated with VA-ECMO, early application of moderate hypothermia for 24 h did not significantly increase survival compared with normothermia. However, because the confidence interval was wide and included a potentially clinical important effect size, these findings should be considered inconclusive.

**Reference 1:** Levy B et al. JAMA. 2022 Feb 1;327(5):442-453. https://doi.org/10.1001/jama.2021.24776.

**Compliance with ethics regulations:** Yes in clinical research.
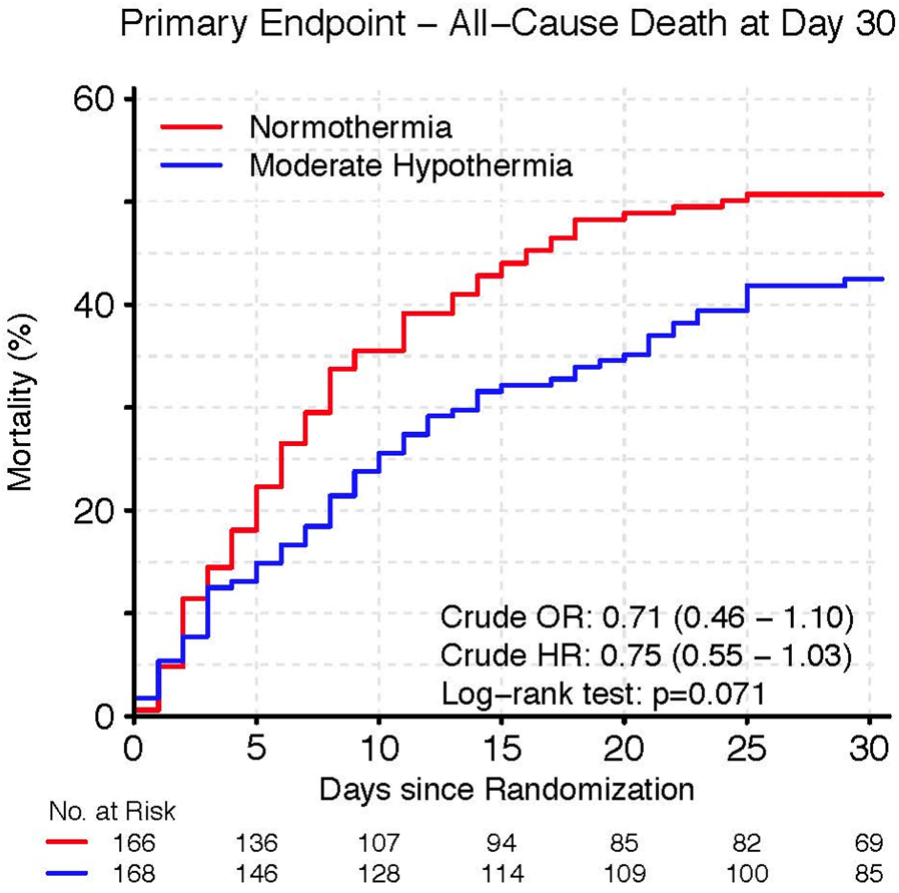


*Kaplan Meier survival estimates during the first 30 days of VA-ECMO patients treated with moderate hypothermia or normothermia. Median (IQR) of observation time was 8 (28, 30) days.*

### CO-35 Effects of mean arterial pressure target on mottling and arterial lactate normalization in patients with septic shock

#### FAGE Nicolas^1,2^, DEMISELLE Julien^3,4^, SEEGERS Valérie^5^, MERDJI Hamid^3,4^, GRELON Fabien^6^, MEGARBANE Bruno^7^, ANGUEL Nadia^8^, MIRA Jean-Paul^9^, DEQUIN Pierre-François^10^, GERGAUD Soizic^11^, WEISS Nicolas^12^, LEGAY François^13^, LE TULZO Yves^14^, CONRAD Marie^15^, COUDROY Rémi^16^, GONZALEZ Frédéric^17^, GUITTON Christophe^18^, TAMION Fabienne^19^, TONNELIER Jean-Marie^20^, BEDOS Jean Pierre^21^, VAN DER LINDEN Thierry^22^, VIEILLARD-BARON Antoine^23,24^, MARIOTTE Eric^25^, PRADEL Gaël^26^, LESIEUR Olivier^27^, RICARD Jean-Damien^28^, HERVE Fabien^29^, DU CHEYRON Damien^30^, GUERIN Claude^31^, MERCAT Alain^1^, TEBOUL Jean Louis^8^, RADERMACHER Peter^32^, ASFAR Pierre^1^

##### ^1^Department of Medical Intensive Care, University Hospital of Angers, Angers, France; ^2^MITOVASC Laboratory UMR INSERM (French National Institute of Health and Medical Research), 1083 – CNRS 6015, University of Angers, Angers, France; ^3^Department of Intensive Care (Service de Médecine Intensive – Réanimation), Nouvel Hôpital Civil, University Hospital of Strasbourg, Strasbourg, France; ^4^INSERM (French National Institute of Health and Medical Research), UMR 1260, Regenerative Nanomedicine (RNM), FMTS (Fédération de Médecine Translationnelle de Strasbourg), University of Strasbourg, Strasbourg, France; ^5^Service de Biométrie, Institut de Cancérologie de l'Ouest, Centre Paul Papin, Angers, France; ^6^Medical and Surgical Intensive Care Unit, Le Mans Hospital, Le Mans, France; ^7^Department of Medical and Toxicological Critical Care, Lariboisière Hospital, Paris University, INSERM UMRS-1144, Paris, France; ^8^Department of Medical Intensive Care, Bicêtre University Hospital, AP-HP, Paris-Saclay University, Le Kremlin Bicêtre, France; ^9^Department of Medical Intensive Care, Cochin University Hospital, Paris, France; ^10^Department of Medical Intensive Care, Tours University Hospital, Tours, France; ^11^Department of Surgical Intensive Care, University Hospital of Angers, Angers, France; ^12^Department of Medical Intensive Care, Georges Pompidou European Hospital, Assistance Publique – Hôpitaux de Paris, University of Paris, Paris, France; ^13^Medical and Surgical Intensive Care Unit, Saint Brieuc Hospital, Saint Brieuc, France; ^14^Department of Infectious Diseases and Medical Intensive Care, Rennes University Hospital, Rennes, France; ^15^Department of Medical Intensive Care, Nancy University Hospital, Nancy, France; ^16^Department of Medical Intensive Care, Université de Poitiers, CHU Poitiers, Poitiers, France; ^17^Department of Medical and Surgical Intensive Care, Avicenne Teaching Hospital, Bobigny, France; ^18^Department of Medical Intensive Care, Nantes University Hospital, Nantes, France; ^19^Department of Medical Intensive Care, Rouen University Hospital, Rouen, France; ^20^Department of Medical Intensive Care, Brest University Hospital, Brest, France; ^21^Intensive Care Unit, Versailles Hospital, Le Chesnay, France; ^22^Department of Intensive Care, Saint Philibert hospital, Catholic University of Lille, Lille, France; ^23^Department of Medical Intensive Care, University Hospital of Ambroise Paré, Boulogne Billancourt, France; ^24^Inserm U1018, Center for Research in Epidemiology and Population Health (CESP), Paris Saclay University, Villejuif, France; ^25^Department of Intensive Care, Saint Louis Hospital, Paris, France; ^26^Department of Intensive Care, Avignon Hospital, Avignon, France; ^27^Department of Medical and Surgical Intensive Care, La Rochelle Saint Louis Hospital, La Rochelle, France; ^28^Université de Paris, AP-HP, Hôpital Louis Mourier, DMU ESPRIT, Médecine Intensive Réanimation, Colombes, France; ^29^Department of Medical and Surgical Intensive Care, Quimper Hospital, Quimper, France; ^30^Department of Medical Intensive Care, Caen University Hospital, Caen, France; ^31^Department of Medical Intensive Care, Edouard Herriot Hospital, Lyon, France; ^32^Institut für Anästhesiologische Pathophysiologie und Verfahrensentwicklung, Universitätsklinikum, Helmholtzstrasse 8-1, Ulm, Allemagne

###### Correspondence: Nicolas FAGE (fage.nicolas@gmail.com)

*Annals of Intensive Care* 2022, **12(1):**CO-35

**Rationale:** In patients with septic shock, the impact of mean arterial pressure (MAP) target on the course of mottling remains uncertain. We investigated whether a low-MAP (between 65 and 70 mm Hg) or a high-MAP target (between 80 and 85 mm Hg) would affect the course of mottling and arterial lactate in patients with septic shock.

**Patients and methods/Materials and methods:** For this post hoc analysis of the SESPSISPAM trial (1), we included patients with at least one available data regarding mottling. Data that concerned mottling were considered until the discontinuation of catecholamine or for a maximum of 5 days under vasopressors. The presence or absence of mottling was recorded every 2 h from 2 h after inclusion to the catecholamine weaning. We compared time course of mottling and arterial lactate between the two MAP target groups. We then evaluated the patient’s outcome according to the presence or absence of mottling.

**Results:** We included 747 patients in this analysis: 374 were assigned to the low-MAP target group and 373 to the high-MAP target group. After adjustment for MAP target and confounding factors, the presence of mottling ≥ 6 h during the first 24 h was significantly associated with a higher risk of death at day 90 [Hazard Ratio (HR) 2.28 (1.74–3.00), p < 0.0001]. As compared with low MAP target, a high MAP target did not alter mottling course (Fig. 1) and arterial lactate normalization. Our results were similar when considering only patients who reached the criteria of the SEPSIS-3 definition of septic shock. In addition, when compared to arterial lactate at inclusion, mottling duration appeared to be a better microcirculatory marker of mortality risk. Indeed, patients without mottling or mottling during less than 6 h and with arterial lactate ≥ 2 mmol/l has a lower mortality [HR 1.56 (1.10–2.20)] than patients with mottling during more than 6 h and with arterial lactate < 2 mmol/L [HR of 2.77 (1.80–4.30) (p = 0.005)].

**Conclusion:** In this large-scale study, we showed that a MAP target between 80 and 85 mm Hg, achieved through increased vasopressor doses, did not alter the course of mottling nor arterial lactate normalization. In patients with septic shock, the presence of mottling ≥ 6 h was associated with higher mortality at day 28 and day 90. In addition, compared to arterial lactate at inclusion, mottling duration appears to be a stronger marker of mortality risk.

**Reference 1:** Asfar P, Meziani F, Hamel J-F et al. High versus low blood-pressure target in patients with septic shock. N Engl J Med. 24 avr 2014;370(17):1583–93.

**Compliance with ethics regulations:** Yes in clinical research.
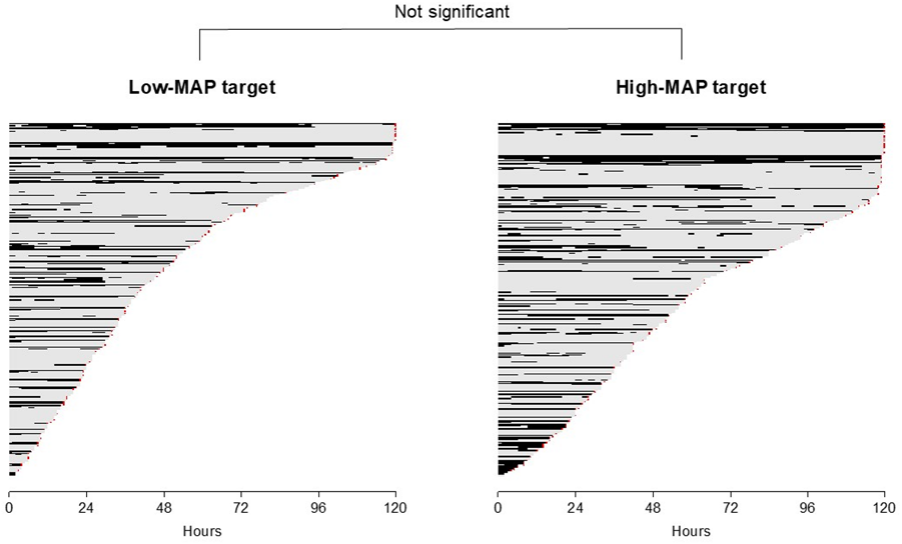


*Course of mottling in patients with septic shock according to the mean arterial pressure target. Horizontal line represents a patient follow-up. Solid line corresponds to a period with mottling; hatched line corresponds to period without mottling*

### CO-36 Right ventricular failure is strongly associated with mortality in patients with moderate-to-severe COVID-19-related ARDS

#### EVRARD Bruno^1^, GOUDELIN Marine^1^, GIRAUDEAU Bruno^2^, BRIAT Charlotte^1^, FEDOU Anne-Laure^1^, DAIX Thomas^1^, VAIDIE Julien^1^, SANSON Amandine^1^, FRANCOIS Bruno^1^, VIGNON Philippe^1^

##### ^1^CHU Limoges, Limoges, France; ^2^CHU de Tours, Tours, France

###### Correspondence: Bruno EVRARD (bruno.evrard@chu-limoges.fr)

*Annals of Intensive Care* 2022, **12(1):**CO-36

**Rationale:** The incidence and prognostic value of new-onset right ventricular failure (RVF) in patients hospitalized in intensive care unit (ICU) for moderate-to-severe acute respiratory distress syndrome (ARDS) related to COVID-19 has been scarcely studied. Accordingly, the objective was to evaluate the association between the development of RVF during ICU stay and 90-day mortality in patients admitted for COVID-19 ARDS. Secondary objective was to evaluate the association between the new-onset RVF and variations of respiratory parameters.

**Patients and methods/Materials and methods:** In this single-center prospective observational study, ICU patients with COVID-19 moderate-to-severe ARDS were serially assessed using echocardiography. RVF was defined as the association of dilated RV (RV/left ventricular end-diastolic area > 0.6) and elevated central venous pressure (≥ 8 mmHg). Multivariate Cox model analysis accounting for new-onset RVF as time-dependent variable was used to identify parameters associated with mortality. In the subset of patients without RVF at baseline who subsequently developed RVF during the ICU stay, we compared respiratory parameters between the day of the RVF diagnosis and the day of the preceding CCE assessment using a Wilcoxon signed-rank test.

**Results:** Overall, 401 echocardiographic assessments were performed in 140 patients, 43 of whom died at day 90 (31%). RVF was identified within 72 h of ICU admission in 35 patients and secondarily in 40 patients. RVF was independently associated with 90-day mortality (adjusted hazard ratio: 8.17; 95% confidence intervals: 3.15–21.2; p < 0.001). Other independent risk factors were age (HR per 10 years: 1.84; 95% CI: 1.20–2.81; p = 0.005) and ischemic cardiomyopathy (HR: 2.26; 95% CI: 1.19–4.31; p = 0.013). Worsening of PaO_2_/FiO_2_ (median [quartiles]: 70 mmHg [57–126] vs. 96 mmHg [69–159]: p = 0.005), PaCO_2_ (60 mmHg [46–65] vs. 51 mmHg [35–61] p = 0.036), ventilatory ratio (2.73 [2.29–3.57] vs. 2.25 [1.51–2.92]: p = 0.004), and driving pressure (14 cm H_2_O [13–19] vs. 13 cm H_2_O [10–14]: p = 0.005) were associated with new-onset RVF.

**Conclusion:** The development of RVF during ICU stay was independently associated with a markedly higher risk of 90-days mortality and appeared associated with a worsening of respiratory parameters.

**Compliance with ethics regulations:** Yes in clinical research.

### CO-37 Sedation-analgesia in pediatric ICU: PEDIASLEEP study

#### ALLARY Chloé^1^, RONCIN César^1^, MICHEL Fabrice^1^

##### ^1^La Timone - Assistance Publique des Hôpitaux de Marseille, Marseille, France

###### Correspondence: Chloé ALLARY (chloe.allary@gmail.com)

*Annals of Intensive Care* 2022, **12(1):**CO-37

**Rationale:** Sedation in pediatric intensive care units (PICU) is balancing between endangerment patient and the risk of tachyphylaxis, withdrawal syndrome and delirium (1). The main objective was to describe sedation-analgesia procedures in France. The secondary objectives was to specify the use of drugs without marketing authorization, and to observe the drugs associations.

**Patients and methods/Materials and methods:** We conducted a multicentric, descriptive and prospective study in France, between May and July 2021. PICU were joined by the PICURE network or by the chief of department. The referring doctors answered a first online survey describing their unit. They answered a second online survey about each sedated patient on the days of the study. The included patients were hospitalized in PICU; aged between 38 weeks of amenorrhea (WA) and 18 years old; received drugs for sedation or withdrawal syndrome. Excluded patients received painkillers against pure nociceptive pain, or palliative or terminal sedation.

**Results:** Thirty PICU were included, between 8 and 34 beds. Fourteen centers (47%) used protocols. Nurses were autonomous to set dose of sedation according to protocol in 13 centers. Four hundred and two questionnaires were filed. Twelve were excluded: 5 because of age < 38 WA, 7 because they received pure analgesia. Patients ages were: neonates 19%, infant 39%, 2-6 years old 17%, 6–10 years old 5%, 10–14 years old 8%, and 14–17 years old 11%. Forty eight percent patients were in postoperative care, including 30% post cardiotomy; 74% had invasive ventilation, 4% extra corporeal membrane oxygenation, 5% hemodialysis. Sedation was scored in 291 (74%) patients mainly by Comfort Behavior Scale (55%). Pain was scored in 314 (80%) patients, mainly using the Comfort Behavior Scale (50%). Withdrawal was scored in 214 patients (55%), mainly using the Withdrawal Assessment Tool 1. Sixteen per cent patients received no opioids; 42% received Morphine, and 36% Sufentanil. Non-opioid antalgics were: Acetaminophen (78%), NSAIDs, Nefopam, Nalbuphine. While 22% patients didn’t received hypnotic, 57% received Midazolam, 34% alpha agonist, 18% Ketamine, 8,5% Propofol. Use of Pentothal, Levomepromazine and Sevoflurane was uncommon. Only 9% of patients received neuromuscular blocking agents, mainly Cisatracurium and Atracurium. Non-drug treatment was used in 77% cases: mainly parental presence, circadian rhythm observance, music therapy and cocooning.

**Conclusion:** The most frequent sedation includes an association between an opioid and a benzodiazepine. Non-drug cares are widely used. Though recommended, scores are not often used.

**Reference 1:** Harris et al. Clinical recommendations for pain, sedation, withdrawal and delirium assessment in critically ill infants and children: an ESPNIC position statement for healthcare professionals, Intensive Care Med 2016 Jun;42(6):972–86.

**Compliance with ethics regulations:** Yes in clinical research.

### CO-38 Retrospective study on prolonged sedation effects with inhaled agents in PICU

#### BERGER Léo^1^, MIATELO Jordi^2^, LÉGER Pierre-Louis^1^

##### ^1^Hôpital Armand-Trousseau, Paris, France; ^2^Hôpital Bicêtre, Le Kremlin-Bicêtre, France

###### Correspondence: Léo BERGER (leo.berger@aphp.fr)

*Annals of Intensive Care* 2022, **12(1):**CO-38

**Rationale:** In ICU, sedation-analgesia is a major therapeutic element to provide adequate comfort to the patient and to permit a good synchronization with the respirator. Currently, it is common to associate benzodiazepine with opioid. However, after prolonged sedation, effects of the sedative become exhausted, requiring an increase in doses, leading to an increased incidence of withdrawal syndrome, and delaying extubation. Since the beginning of the XXI century, there has been a growing interest in the use of halogenated gases in ICU, because of their hypnotic effects. Furthermore, there is little data on their use during prolonged sedation. Therefore, their efficacy and tolerance should be assessed. They could reduce the dosage of benzodiazepines and opioids, thereby reducing the incidence of withdrawal syndrome.

**Patients and methods/Materials and methods:** This is a retrospective, multicentric cohort study. 50 children (median age 2.2 years, [0.8–7.2]) admitted to PICU between January 2018 and December 2020 were included. All of them received multimodal prolonged sedation (> 72 h) for mechanical ventilation. They all benefited from volatile sedation (Isoflurane of Sevoflurane) for 24 h at least. The primary endpoint was a decrease superior to 20% in benzodiazepine dosages during the first 24 h after halogenated introduction. A 20% decrease in other hypnotics, NMBA, and opioids were also explored. The proportion of adverse events and withdrawal syndrome were collected.

**Results:** A statistically significant reduction in benzodiazepines dosages (μg/kg/h) was reported (118 [62.5; 200] vs 80.0 [32.5; 120], p < 0,01). The same results were found for other hypnotics (Ketamine: 2.00 [1.00; 2.00] vs 1.50 [1.00; 2.00], p = 0.036, mg/kg/h; Clonidine: 0.55 [0.35; 1.27] vs 0.20 [0.12; 0.43], p = 0.036, μg/kg/h). For opioids (μg/kg/h), only Sufentanil (0.80 [0.50; 1.00] VS 0.70 [0.35; 1.00]; p = 0.022) and Fentanyl (6.00 [4.00; 8.00] VS 5.75 [3.25; 6.00], p = 0.012) decreased significantly. No major adverse effects were reported. 26% of patients developed withdrawal syndrome.

**Conclusion:** Halogenated gases seem to be an interesting therapeutic to reduce dosages of different hypnotics and opioids used during multimodal prolonged sedations. Inhalation of halogenated gas via the ACD seems to be sure and easy to use. That appears to be a simple method for maintaining long-term sedation in PICU. Our results suggested IA sedation using the ACD to be an effective and safe alternative to the usual intravenous Propofol- or Midazolam-based regimen. Sevoflurane provided sedation quality comparable to Propofol and Midazolam, but with decreased wake-up and extubation times.

**Compliance with ethics regulations:** Yes in clinical research.

### CO-39 3D and Thermography video research infrastructure for multi-modal image acquisition in the Pediatric Intensive Care Unit

#### SHCHERBAKOVA Monisha^1^, BOIVIN Vincent^2^, TIASSOU Edem^1^, WILLGENSS Priscilla^2^, VAIL Mariane^3^, SAUTHIER Michael^1^, JOUVET Philippe^1^, NOUMEIR Rita^2^

##### ^1^CHU Sainte Justine, Montreal, Canada; ^2^Ecole de technologie supérieure, Montreal, Canada; ^3^HEC Montreal, Montreal, Canada

###### Correspondence: Monisha SHCHERBAKOVA (monisha.shcherbakova.hsj@ssss.gouv.qc.ca)

*Annals of Intensive Care* 2022, **12(1):**CO-39

**Rationale:** Computer vision has promising potential for the diagnosis of vital distress in critically ill patients including neurological, respiratory and hemodynamic distress. For instance, measurement of respiratory rate using 3D videos in spontaneous breathing patients and correlation between low cardiac output and thermal distribution using infrared (IR) images were reported in several studies. The aim of our research was to setup a multi-modal video infrastructure to create a clinical video research database and pave the way to real-time vital distress video monitoring within a pediatric intensive care unit (PICU).

**Patients and methods/Materials and methods:** The specifications of the infrastructure were developed by an interdisciplinary team including clinicians, computer scientists, and hospital information technology personnel. These specifications took into account the requirements of the research ethics board: (1) a multimodal video system (MMVS) that is able to capture color (RGB) videos for neurological distress analysis, 3D videos for respiratory distress analysis and thermal videos for hemodynamic analysis; (2) a MMVS in each room on the ceiling above the patient’s bed; (3) security systems to only register videos of patients who have consented to the study; (4) automatic data storage on the hospital private servers; (5) synchronization of the videos with clinical data collected simultaneously; (6) data classification in a research database.

**Results:** The MMVS developed included a hardware and a software component (Fig. 1). The hardware component consisted of a NVIDIA Jetson Xavier NX mini-computer that has a built-in GPU (NVIDIA Corp, USA) connected to a Kinect Azure RGB-3D camera (Microsoft, USA) and a FLIR Lepton 3.5 thermal sensor (Teledyne FLIR, USA). Both cameras are pointed towards the patient and are placed in a 3D printed support. The software component included a web user interface for performing the acquisitions in the hospital intranet in a secure manner. The video research database currently includes more than 200 acquisitions and is being used in various studies to assess vital distress signs including facial expressions to estimate sedation level and consciousness using RGB videos, the refinement of tidal volume and respiratory rate measurements in patients using 3D videos, and estimation of the thermal gradient across various parts of the body using IR.

**Conclusion:** A multi-modal RGB 3D and thermography video infrastructure was successfully set up and a video research database of critically ill children was constructed and enabled algorithm development to assess various vital distress. This setup could be replicated in other PICUs.

**Compliance with ethics regulations:** Yes in clinical research.

### CO-40 Volume per kg per transfusion of the first red blood cell (RBC) transfusion given to participants of the ABC-PICU randomized controlled trial, for the ABC-PICU Investigators, CCCTG, PALISI Network, BloodNet Network, and GFRUP

#### GALLAND Anne^1^, DU PONT-THIDODEAU Geneviève^1^, SPINELLA Philipp^2^, LETEURTRE Stéphane^3^, DUCRUET Thierry^4^, LACROIX Jacques^1^, TUCCI Marisa^1^

##### ^1^Division of Pediatric Critical Care Medicine, Department of Pediatrics, CHU Sainte-Justine, Université de Montréal, Montreal, Canada; ^2^Department of Surgery and Department of Critical Care Medicine, University of Pittsburgh, Pittsburgh, Etats-Unis; ^3^Réanimation et Surveillance Continue Pédiatriques, Hôpital Jeanne de Flandre, CHRU Lille, Lille, France; ^4^Unité de recherches cliniques appliquées, Research Centre, CHU Sainte-Justine, Montreal, Canada

###### Correspondence: Jacques LACROIX (jlacroix052@gmail.com)

*Annals of Intensive Care* 2022, **12(1):**CO-40

**Rationale:** New *et al*^1^ completed a national cohort study in the United Kingdom (UK); all hospitals were invited to participate. Hospitalized children—not only those in pediatric intensive care units (PICU)—< 18 years old who received an RBC transfusion during a 3-month period in 2009 were eligible for inclusion. The median volume was 15.0 (IQR: 11.8–19.2) mL/kg per transfusion, with peaks of prescription volume at 10, 15 and 20 mL/kg. Almost no specific attention has been paid to the volume of RBC transfusion administered in PICU. We determined the volume/kg/transfusion of the first RBC transfusion given to participants in the Age of Blood in Children in PICU (ABC-PICU) trial.^2^ The hypothesis was that we would find the same variability in the volume/kg administered to PICU patients than reported by New in hospitalized children.

**Patients and methods/Materials and methods:** Patients enrolled in ABC-PICU were allocated to receive either fresh (stored <7 days) or standard-issue RBC (delivery of oldest compatible unit available in the blood bank). The volume of RBC unit per transfusion was not controlled in ABC-PICU. Cardiac patients were randomized before surgery; other patients were randomized in PICU. In the former, data on RBC transfusion were collected in the operating room and PICU while data in the latter were collected only in PICU.

**Results:** 1538 patients across 50 centers (US, Canada, France, Italy, Israel) were randomized between February 2014 and August 2018; 1474, including 1263 non-cardiac patients, received at least one RBC transfusion. Figure 1 reports the number of participants to ABC-PICU per range of volume of the first RBC transfusion administered after randomization. Among the 1474 participants, 105 (7.1%) received > 30 mL/kg/transfusion; most (82/105 = 78.1%) were cardiac patients who were first transfused while priming a cardio-pulmonary bypass. Among 1263 non-cardiac participants, 736 (58.3%) received between 9 and 16.99 mL/kg/transfusion; a significant proportion received < 9 or > 17 mL/kg/transfusion (286 or 22.6% and 241 or 19.1%, respectively).

**Conclusion:** There is an important variability in the volume/kg/transfusion given to participants in ABC-PICU. Data on bleeding status and on-site policy limiting volume per transfusion (e.g., not more than one unit per transfusion) were not collected in ABC-PICU, which limits the interpretation of the results reported in this abstract. Future studies taking into account patients’ bleeding status and site-specific policy on volume per transfusion must investigate what are the causes and the clinical impact of the variability that we observed in ABC-PICU participants.

**Reference 1:** New HV, Grant-Casey J, Lowe D, Kelleher A, Hennem S, Stanworth SJ. Red blood cell transfusion practice in children: current status and areas for improvement? A study of the use of red blood cell transfusions in children and infants. Transfusion 2014;54.

**Reference 2:** Spinella PC, Tucci M, Fergusson DA, Lacroix J, Hébert PC, Leteurtre S, et al. The age of transfused blood in critically ill children. JAMA 2019;322:2179–90.

**Compliance with ethics regulations:** Yes in clinical research.
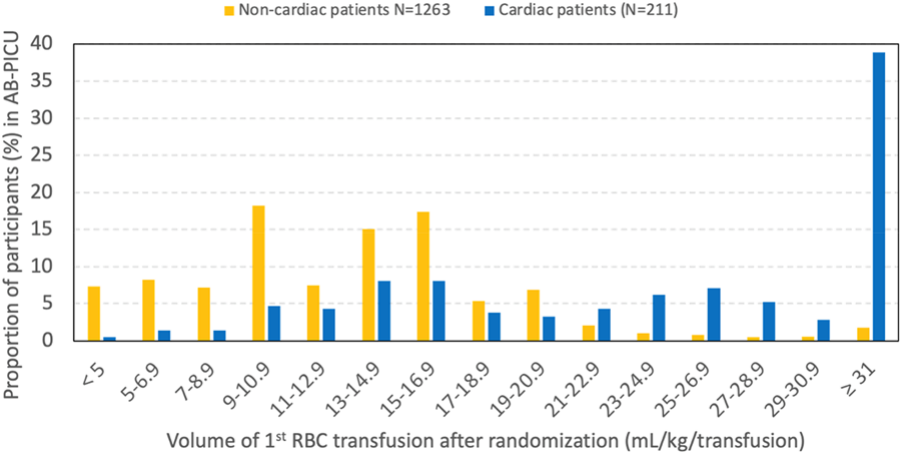


*Volume of first red cell transfusion given after randomization to 1263 non-cardiac and 211 cardiac participants to the ABC-PICU trial.*

### CO-41 In situ simulation training for parental presence during critical situations in PICU: an observational study

#### BORDESSOULE Alice^1^, FELICE-CIVITILLO Cristina^1^, GRAZIOLI Serge^1^, BARCOS Francisca^1^, HADDAD Kevin^1^, RIMENSBERGER Peter^1^, POLITO Angelo^1^

##### ^1^HUG, Geneva, Suisse

###### Correspondence: Alice BORDESSOULE (alice.bordessoule@hcuge.ch)

*Annals of Intensive Care* 2022, **12(1):**CO-41

**Rationale:** Family presence during invasive procedures or cardiopulmonary resuscitation (CPR) is a part of the family-centered approach in pediatric intensive care units (PICUs). We established a simulation program aiming at providing communication tools to healthcare professionals. The goal of this study was to evaluate the impact of this program on the stress of PICU professionals and its acceptance.

**Patients and methods/Materials and methods:** An observational study of a simulation program, with questionnaire, was used to measure pre- and post-simulation stress and the degree of satisfaction of the participants. Setting: PICU of Geneva Children’s Hospital, Switzerland. Forty simulations with four different simulation scenarios and various types of parental behavior, as imitated by professional actors, were completed during a 1-year period. Primary outcomes were the difference in perceived stress level before and after the simulation and the degree of satisfaction of healthcare professionals (nursing assistants, nurses, physicians). The impact of previous experience with family members during critical situations or CPR was evaluated by variation in perceived stress level.

**Results:** Overall, 201 questionnaires were analyzed. Perceived stress associated with parental presence decreased from a pre-simulation value of 6 (IQR, 4–7) to 4 (IQR, 2–5) post-simulation on a scale of 1–10. However, in 25.7% of cases, the individually perceived post-simulation stress level was higher than the pre-simulation one. Satisfaction of the participants was high with a median of 10 (IQR, 9–10) out of 10 (Fig. 1).

**Discussion:** Our study describes an in situ pediatric simulation program of critical situations in the presence of family members played by professional actors. The simulation program was explicitly aiming at the development of communication skills. The “in situ” setting and the possibility to debrief in the presence of a psychologist represent two innovative features of our study. The simulation generally, but not always, showed a reduction in healthcare professionals perceived stress caused by family presence during a critical situation in the PICU. To meet the demand of participants experiencing a high level of stress after the simulation, a pre-simulation video on potential benefits and pitfalls of family presence during critical situations as well as on ad-hoc communication tools is now available to all future participants.

**Conclusion:** A simulation program helps reduce PICU team emotional stress associated with the presence of family members during critical situations or CPR, and is welcomed by PICU team members.

**Compliance with ethics regulations:** Yes in clinical research.
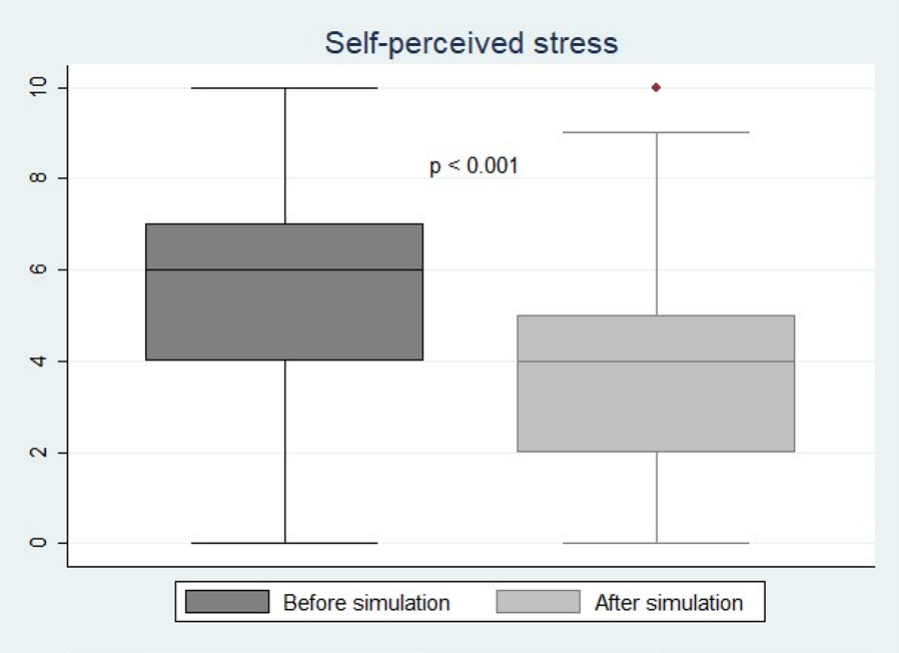


*Figure 1. Box plot showing overall pediatric intensive care team stress related to family member presence during cardiopulmonary resuscitation or other major interventions in the pediatric intensive care unit, before and after the simulation.*

### CO-42 Beta-lactam exposure and safety in intermittent or continuous infusion in critically ill children

#### DEBRAY Agathe^3^, CALLOT Delphine^2^, HIRT Deborah^2^, BILLE Emmanuelle^1^, RENOLLEAU Sylvain^1^, CHOUCHANA Laurent^2^, TRELUYER Jean-Marc^1,2^, OUALHA Mehdi^1^, BERANGER Agathe^1^

##### ^1^Necker enfants malades, Paris, France; ^2^cochin, Paris, France; ^3^trousseau, Paris, France

###### Correspondence: Agathe BERANGER (agathe.beranger@gmail.com)

*Annals of Intensive Care* 2022, **12(1):**CO-42

**Rationale:** To assess the pharmacokinetic (PK) efficacy and clinical toxicity for three beta-lactams: cefotaxime, piperacillin/tazobactam and meropenem, depending on two administration modalities: continuous or intermittent infusions in critically ill children.

**Patients and methods/Materials and methods:** This single center observational prospective study was conducted in a pediatric intensive care unit. All hospitalized children who had one measured plasma concentration of the investigated antibiotics were included. Plasma antibiotic concentrations were interpreted by a pharmacologist, using a Bayesian approach based on previously published population pharmacokinetic models. Exposure was considered optimal, low, or high according to the PK target 100% fT > 4 × MIC and a trough concentration below the toxic concentration (50 mg.L^−1^ for cefotaxime, 150 mg.L^−1^ for piperacillin and 44 mg.L^−1^ for meropenem).

**Results:** Between May 2019 and January 2020, 80 patients were included and received 106 antibiotic courses: 74 (70%) were administered in intermittent infusion (II) and 32 (30%) in continuous infusion (CI). Compared to II, CI provided more optimal PK exposure (OR 1.2, 95% CI 1.01–1.5, p = 0.04), less underexposure (OR 0.7, 95% CI 0.6–0.84, p < 0.001) and more overexposure (OR 1.2, 95% CI 1.03–1.3, p = 0.01) (Fig. 1). Five adverse events have been reported during the study period, although none have been attributed to beta-lactam treatment.

**Conclusion:** Continuous infusion provided a higher probability to attain an optimal PK target compared to intermittent infusion, but also a higher risk for overexposure. Regular therapeutic drug monitoring is recommended in critically ill children receiving beta-lactams, regardless of the administration modality.

**Compliance with ethics regulations:** Yes in clinical research.
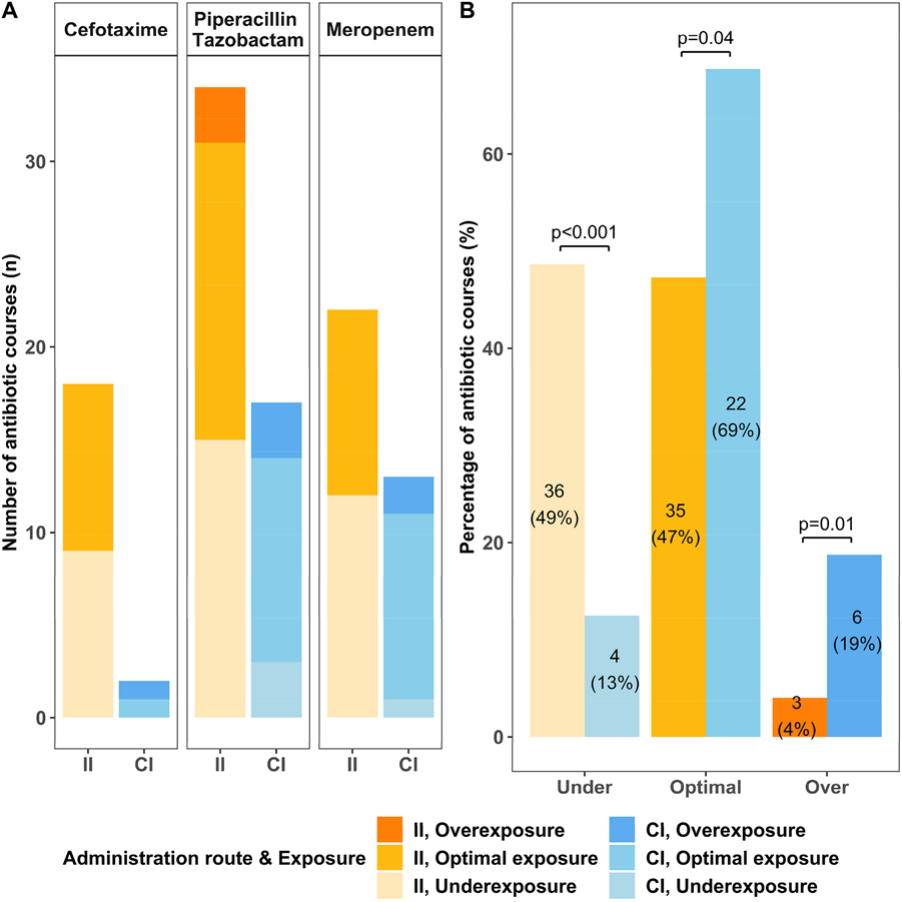


*Description of the antibiotic exposure (underexposure, optimal exposure or overexposure) depending on the drug (A) or for the whole cohort (B), with respect to the administration route, intermittent infusion (II) or continuous infusion (CI).*

### CO-43 Incidence and risk factors of weaning-induced cardiac dysfunction: results from a multicenter, observational study

#### SHI Rui^1^, AYED Soufia^1^, TEBOUL Jean-Louis^1^, ANGUEL Nadia^1^, OSMAN David^1^, PHAM Tài^1^, MORETTO Francesca^1^, LAI Christopher^1^, BEUZELIN Marie^2^, PERSICHINI Romain^3^, LEGOUGE Marie^4^, DE VITA Nello^5^, LEVY Bruno^6^, BEURTON Alexandra^7^, MANGAL Kishore^8^, HULLIN Thomas^9^, LABBE Vincent^10^, GUILLOT Max^11^, HARROIS Anatole^12^, MONNET Xavier^1^

##### ^1^Service de médecine intensive-réanimation, Université Paris-Saclay, AP-HP, Hôpital de Bicêtre, DMU CORREVE, Inserm UMR S_999, FHU SEPSIS, Groupe de recherche clinique CARMAS, Le Kremlin-Bicêtre, France; ^2^Service de réanimation polyvalente, Centre hospitalier de Dieppe, Dieppe, France; ^3^Service de réanimation médicale, Hôpital Félix Guyon, Saint-Denis-De-La-Réunion, France; ^4^Service de d’anesthésie-réanimation adultes, Centre hospitalier d'Orléans, Orléans, France; ^5^Università del Piemonte Orientale, Dipartimento di Medicina Traslazionale, Novara, Italie; ^6^Service de réanimation médicale, Hôpital de Nancy-Brabois, Vandoeuvre-Lès-Nancy, France; ^7^AP-HP, Groupe Hospitalier Universitaire APHP-Sorbonne Université, site Pitié-Salpêtrière, Service de Pneumologie, Médecine intensive Réanimation (Département R3S), Paris, France; ^8^Department of Medicine and Critical Care, Eternal Hospital, Eternal Heart Care Centre & Research Institute, Jaipur, Inde; ^9^Service de réanimation polyvalente, Hôpital Sud-Essone, Etampes, France; ^10^Service de réanimation, AP-HP, Groupe Hospitalier Universitaire APHP-Sorbonne Université, site Tenon, Paris, France; ^11^Service de réanimation médicale, Hôpital de Strasbourg, Strasbourg, France; ^12^Service d'anesthésie-réanimation, Université Paris-Saclay, AP-HP, Hôpital de Bicêtre, Le Kremlin-Bicêtre, France

###### Correspondence: Rui SHI (rui.shi@u-psud.fr)

*Annals of Intensive Care* 2022, **12(1):**CO-43

**Rationale:** Weaning-induced pulmonary edema (WiPO) is one of the main reasons for weaning failure. Nevertheless, the reported incidence of WiPO is variable mainly in monocentric studies with small sample size. We thus aimed to evaluate the incidence and risk factors of WiPO in a large mixed population of critically ill patients.

**Patients and methods/Materials and methods:** Adult critically ill patients receiving invasive ventilation were included once the attending physicians decided to perform a spontaneous breathing trial (SBT). Patients with tracheostomy were excluded. The duration and modalities of the SBT (T-tube/pressure support or others) were decided by attending physicians. The consensual diagnosis of WiPO was made a posteriori by five experts based on the patient characteristics, hemodynamic and echocardiographic variables, and biochemical results.

**Results:** From July 2019 to February 2021, 634 SBT performed in 500 patients (65 (55–74) y.o., 64% male) from twelve intensive care units, were prospectively included. The main indication for intubation was acute respiratory failure in 202 (40%) patients, neurological failure in 100 (20%) patients, interventional procedure in 74 (15%) patients, shock in 55 (11%) patients, resuscitated cardiac arrest in 10 (2%) patients, a mixture of previous reasons in 53 (11%) patients and miscellaneous reasons in 6 (1%) patients. SBT failed at 217 (34%) occurrences observed in 157 (31%) patients. WiPO was identified in 79 (36%) failed SBTs in 58 (37%) patients. We assessed WiPO according to the main indication for intubation, the prevalence of WiPO was 73% in patients intubated for acute respiratory failure/shock/sepsis, 13% in patients intubated for neurological failure, 9% in patients intubated after resuscitated cardiac arrest, and 5% in those intubated for an interventional procedure. Among 217 failed SBTs, WiPO occurred in 54 (25%) of SBTs performed with a T tube, 16 (7%) with pressure support ventilation, and 9 (4%) with other modalities. Compared to patients without WiPO (n = 98), patients with at least one WiPO (n = 58) had a higher prevalence of chronic obstructive pulmonary disease (COPD) (27% vs. 7%, respectively; p = 0.001), and previous cardiopathy (dilated and/or hypertrophic and/or valvular disease, 49% vs. 23%, respectively; p = 0.002). A logistic regression analysis found that, COPD (odds ratio (OR): 5.1, [95% confidence interval: 1.9–14.0]), and previous cardiopathy (OR: 2.9 [1.4–6.2]) were independent risk factors for developing WiPO.

**Conclusion:** In a large mixed population of critically ill patients, WiPO accounts for 36% of SBT failure. COPD and a previous cardiopathy were independent risk factors for developing WiPO.

**Compliance with ethics regulations:** Yes in clinical research.

### CO-44 Plasma exchange are not associated with better outcome in MDA5 rapidly-progressive interstitial lung disease

#### BAY Pierre^1^, PINETON DE CHAMBRUN Marc^2^, ROTHSTEIN Vincent^3^, MAHEVAS Matthieu^1^, DE PROST Nicolas^1^, ROUX Antoine^4^, ZUBER Benjamin^4^, ISRAEL BIET Dominique^5^, HERVIER Baptiste^6^, TAZI Abdellatif^6^, MOUTHON Luc^7^, MEKINIAN Arsene^9^, DELIGNY Christophe^15^, BORIE Raphael^8^, MEURICE Jean Claude^10^, PRIOU Pascaline^10^, MEYER Alain^12^, SAVALE Laurent^14^, DE SAINT MARTIN Luc^13^, BRILLIET Pierre-Yves^3^, KHAFAGY Philippe^3^, BENVENISTE Olivier^2^, NUNES Hilario^3^, ALLENBACH Yves^2^, UZUNHAN Yurdagul^3^

##### ^1^CHU Henri Mondor, Créteil, France; ^2^CHU Pitié Salpétrière, France, France; ^3^CHU Avicenne, Bobigny, France; ^4^Hopital Foch, Suresnes, France; ^5^Hopital Européen Georges Pompidou, Paris, France; ^6^CHU Saint Louis, Paris, France; ^7^CHU Cochin, Paris, France; ^8^CHU Bichat, Paris, France; ^9^CHU Saint Antoine, Paris, France; ^10^CHU Poitiers, Poitiers, France; ^11^CHU Angers, Angers, France; ^12^CHU Strasbourg, Strasbourg, France; ^13^CHU Brest, Brest, France; ^14^CHU Bicetre, Le Kremlin Bicetre, France; ^15^CHU Martinique, Fort De France, France

**Correspondence:** Pierre BAY (pierrebay53@yahoo.fr)

*Annals of Intensive Care* 2022, **12(1):**CO-44

**Rationale:** Rapidly progressive interstitial lung disease (RP-ILD) is a frequent manifestation of anti-MDA5 dermatomyositis (DM) and is associated with a high mortality rate. The appropriate treatment regimen of anti-MDA5 RP-ILD is uncertain. A potential pathogenic role of MDA5 antibodies motivated plasma exchange (PLEX) but whether the effectiveness of this procedure is unknown. The aim of the study was to evaluate the outcome of patients undergoing PLEX for anti-MDA5 RP-ILD.

**Patients and methods/Materials and methods:** This French multicenter retrospective study was conducted from 2012 to 2021 in 18 hospitals and included all patients with anti-MDA5 RP-ILD. The primary endpoint was one-year mortality.

**Results:** 51 patients with anti-MDA5 RP-ILD (female 67%; mean age at disease onset: 51 ± 11.6 years) were included. 32 patients (62.7%) required mechanical ventilation. 25 patients (49%) received PLEX. Baseline characteristics were not different between PLEX+ and PLEX− patients. PLEX+ patients received more immunosuppressants and required more often mechanical ventilation (19 PLEX+ vs 13 PLEX−, p 0.05). One-year morality rate for PLEX+ and PLEX− patients was 64% and 42.6%, respectively. The Kaplan–Meier estimated probabilities of one-year survival were similar for PLEX+ vs. PLEX− patients (Fig. 1).

**Conclusion:** Anti-MDA5 RP-ILD is associated with a high 1-year mortality rate. The use of PLEX was not associated with a favorable outcome. Further studies are needed to evaluate their efficacy.

**Compliance with ethics regulations:** Yes in clinical research.
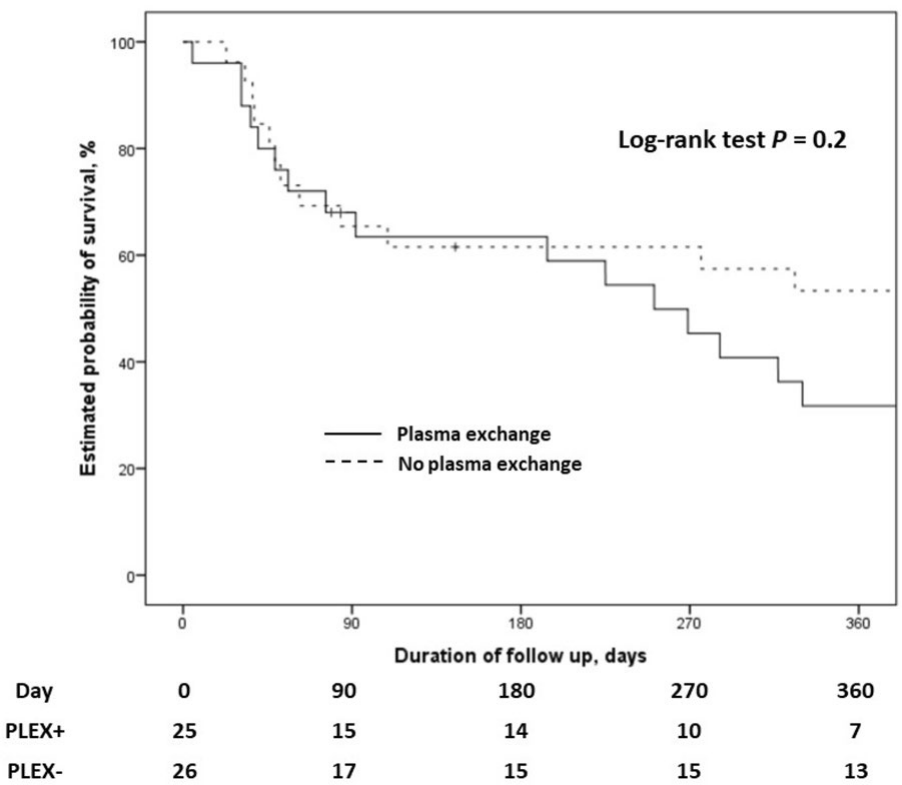


*Kaplan–Meier Curves for the 1-year mortality status according to plasma exchange status.*

### CO-45 Spontaneous pneumomediastinum in critically ill COVID-19 patients

#### ELABBADI Alexandre^1^, TOMAS Urbina^2^, ENORA Berti^3^, CONTOU Damien ^4^, QUINTANA Soulier^2^, CARTEAU Guillaume^3^, VOIROT Guillaume^1^, FARTOUKH Muriel^1^, GIBELIN Aude^1^

##### ^1^Hôpital Tenon, APHP, Paris, France; ^2^Hôpital Saint-Antoine, APHP, Paris, France; ^3^Hôpital Henri Mondor, Créteil, France; ^4^Centre Hospitalier Victor Dupouy, Argenteuil, France

###### Correspondence: Alexandre ELABBADI (alexandre.elabbadi@aphp.fr)

*Annals of Intensive Care* 2022, **12(1):**CO-45

**Rationale:** Spontaneous pneumomediastinum (SP), defined by the presence of air within the mediastinum without traumatic lesion, has been described during ARDS, even in the era of protective ventilation. It has been also described in case series of COVID-19 with severe pneumonia in the absence of use of invasive mechanical ventilation. We aimed at describing the prevalence of spontaneous pneumomediastinum during severe COVID-19 pneumonia, and at investigating its prognostic impact

**Patients and methods/Materials and methods:** We conducted a retrospective multicenter observational study in 4 French intensive care units (ICUs) between August 2020 and April 2021. All patients with laboratory-confirmed COVID-19 with severe pneumonia were included. Spontaneous pneumomediastinum was diagnosed either on chest X-ray or chest CT-scan. The primary endpoint was to estimate the prevalence of SP during COVID-19 with severe pneumonia. Secondary endpoints were to investigate the prognostic impact of SP, using a composite criterion named “complicated course” including mechanical ventilation or death at day-28.

**Results:** During the study period, 672 patients with COVID-19 with severe pneumonia were included. Thirty-two patients (5%) developed a SP after 9 days [4–-13] of COVID-19 onset. A pneumothorax was associated in 10 patients (31%). Two-third of patients (n = 21; 66%) developed a SP after 6 days [4–12] of ICU admission, while they did not receive invasive mechanical ventilation (IMV). Finally, half of these patients (n = 11/21) required IMV after 1 day [0–3] of SP diagnosis. Although the proportion of patients requiring IMV was similar, the time to tracheal intubation was longer in the patients with SP (6 days vs. 2 days; p < 0.01), with a significantly higher first-line use of high-flow nasal oxygen therapy or non-invasive ventilation, and more awake prone positioning sessions. Although there was no difference in “complicated course”, the length of stay in the ICU was significantly higher in the SP group (18 vs 9 days, p = 0.01).

**Conclusion:** Prevalence of SP was not uncommon, affecting nearly 5% of all patients admitted to the ICU. Nearly two third of SP occur before or in the absence of IMV. This suggests that, barotrauma secondary to invasive mechanical ventilation, does not appear to be preponderant in the occurrence of pneumomediastinum in COVID-19. Mechanism is potentially carried-out by patient self-inflected lung injury and hyperinflation secondary to a prolonged respiratory failure, as underlined by a longer delay before invasive mechanical ventilation. Presence of a pneumomediastinum should alert the clinician in a spontaneously breathing patient to its tolerance and the need to use a more protective ventilation.

**Compliance with ethics regulations:** Yes in clinical research.

### CO-46 Attributable mortality and population attributable fraction of death of ventilator-associated pneumonia among pandemic non-COVID-19 patients, and pandemic COVID-19 patients

#### VACHERON Charles-Hervé^1^, LEPAPE Alain^1^, SAVEY Anne^1^, MACHUT Anais^1^, TIMSIT Jean-Francois^2^, COURNO Gaelle^4^, VANHEMS Philippe^5^, LANDEL Verena^5^, LAVIGNE Thierry^6^, BAILLY Sebastien^7^, BETTEGA Francois^7^, COMPAROT Sylvie^3^, REA-REZO Study Group^1^, MAUCORT-BOULCH Delphine^5^, FRIGGERI Arnaud^5^

##### ^1^CHU lyon sud, Lyon, France; ^2^APHP, Paris, France; ^3^CH Avignon, Avignon, France; ^4^CH Toulon, Toulon, France; ^5^CHU Lyon, Lyon, France; ^6^CHU Strasbourg, Strasbourg, France; ^7^CHU Grenoble, Grenoble, France

###### Correspondence: Charles-Hervé VACHERON (charles-herve.vacheron@chu-lyon.fr)

*Annals of Intensive Care* 2022, **12(1):**CO-46

**Rationale:** Patients with a SARS-CoV-2 infection are at higher risk of Ventilator-Associated Pneumonia (VAP) and may have an increased Attributable Mortality (AM) and Population Attributable Fraction (PAF) of mortality related to VAP.

**Patients and methods/Materials and methods:** Using the REA-REZO surveillance network, 3 groups of adult medical ICU patients were computed: control group (patients admitted between 2016 and 2019), pandemic COVID-19 group (PandeCOV+), and pandemic non-COVID-19 group (PandeCOV−) admitted during 2020. The primary outcome was the estimation of AM and PAF related to VAP in these patients. Using multi-state modeling with causal inference, the outcomes related to VAP were also evaluated.

**Results:** A total of 64816 patients were included in the control group, 7442 in the PandeCOV−, and 1687 in the PandeCOV+ . The incidence of VAP was 14.2 (95% CI [13.9;14.6]), 18.3 (95% CI [17.3;19.4]), and 31.9 (95% CI [29.8;34.2]) VAP per 1000 ventilation-day, in each group, respectively. AM at 90 days was 3.15% (95% CI [2.04;3.43]), 2.91% (95% CI [− 0.21;5.02]), and 8.13% (95% CI [3.54–12.24]), and PAF of mortality at 90 days was 1.22% (95% CI [0.83;1.63]), 1.42% (95% CI [− 0.11–2.61]), and 9.17% (95% CI [3.54;12.24]) for the control, PandeCOV−, and PandeCOV+ .groups, respectively. Except for the higher risk of developing a VAP, the PandeCOV− group shared similar VAP characteristics with the control group. PandeCOV+ patients were at lower risk of death without VAP (HR 0.62, 95% CI [0.52;0.74]) and tended to have a higher risk of death after acquiring VAP (HR 1.07, 95% CI [0.86;1.33]) compared to the control group.

**Conclusion:** Patients admitted in ICUs during the pandemic, even without a diagnosis of COVID-19, were at higher risk of VAP. VAP attributable mortality was higher for COVID-19 patients, with more than 9% of the overall mortality related to VAP.

**Compliance with ethics regulations:** Yes in clinical research.

### CO-47 FX06 to rescue acute respiratory distress syndrome during Covid-19 pneumonia. A randomized clinical trial

#### GUERIN Emmanuelle^1^, FRANCHINEAU Guillaume^2^, LE GUENNEC Loic^1^, FRAPARD Thomas^1^, LEFEVRE Lucie^1^, LUYT Charles-Edouard^1^, COMBES Alain^1^, HAYON Jan^2^, ASFAR Pierre^3^, BRECHOT Nicolas^1^

##### ^1^CHU Pitié-Salpêtrière, Paris, France; ^2^CHI de POISSY, Saint-Germain-En-Laye, France; ^3^CHU d'ANGERS, Angers, France

###### Correspondence: Nicolas BRECHOT (nicolas.brechot@aphp.fr)

*Annals of Intensive Care* 2022, **12(1):**CO-47

**Rationale:** Vascular leakage is a major feature of SARS-CoV-2 induced acute respiratory distress syndrome (ARDS). Its levels are associated with mortality, which remains as high as 50%. FX06, a drug under development containing fibrin-derived peptide beta15–42, stabilizes cell-cell interactions, thereby reducing vascular leak and mortality in several animal models of ARDS. It was successfully used as a rescue therapy in a patient exhibiting a severe ARDS following EBOLA virus infection. The aim of this study was to evaluate the efficacy of FX06 in reducing vascular leakage during SARS-CoV-2 induced ARDS.

**Patients and methods/Materials and methods:** We conducted a double-blinded placebo-controlled multicenter trial. Patients receiving invasive mechanical ventilation for less than 5 days for a SARS-CoV-2 induced ARDS were randomized to receive intravenous FX06, 400 mg per day during 5 days, or its placebo, on the top of usual care. The primary endpoint was the reduction of pulmonary vascular leakage from day 1 to day 7, evaluated by transpulmonary thermodilution-derived extra-vascular lung water index (EVLWi). All analyses were conducted on an intent-to-treat basis.

**Results:** After one consent withdrawal, 49 patients were enrolled and randomized, 25 in the FX06 group and 24 in the placebo group. Patients were very severe, with a median SAPS-II score of 57 [IQR 39; 66], a median PaO_2_:FiO_2_ ratio of 104 [69; 165], and a median static pulmonary compliance of 28 ml/cm of water [19; 35]. One third of them were equipped with veno-venous ECMO. Although EVLWi was elevated at baseline (15.6 ml/kg [13.5; 18.5]), the primary endpoint of its reduction from day 1 to day 7 was comparable between groups (− 1.9 ml/kg [− 3.3; − 0.5] in the FX06 group vs. − 0.8 ml/kg [− 5.5; − 1.1] in the placebo group, estimated effect − 0.8 [− 3.1; 2.4], p = 0.51). Cardiac index, pulmonary vascular permeability index, and fluid balance were also comparable between groups. PaO_2_:FiO_2_ ratio remained low and comparable between groups. Duration of mechanical ventilation and survival were also not affected by FX06 infusion, with 21 (84%) patients surviving at day 30 in the FX06 group and 17 (71%) in the placebo group (p = 0.27). Adverse events rates were comparable between groups, although patients receiving the drug experienced more ventilator-associated pneumonia (16/25 vs. 6/24, p = 0.009).

**Conclusion:** In this trial FX06 failed to reduce the level of SARS-CoV-2 induced pulmonary vascular leakage. Further studies are needed to evaluate its efficacy at earlier time points of the disease or using other dosing regimens.

**Compliance with ethics regulations:** Yes in clinical research.

### CO-48 Quality of life and long-term assessment of survivors after extracorporeal membrane oxygenation for severe acute respiratory distress syndrome due to COVID 19

#### CHOMMELOUX Juliette^1^, VALENTIN Simon^2^, ADDA Mélanie^3^, PINETON DE CHAMBRUN Marc^1^, MOYON Quentin^1^, MATHIAN Alexis^1^, CAPELLIER Gilles^4^, GUERVILLY Christophe^3^, LEVY Bruno^2^, JACQUET Pierre^5^, SONNEVILLE Romain^5^, VOIRIOT Guillaume^6^, DEMOULE Alexandre^1^, BOUSSOUAR Samia^1^, BRECHOT Nicolas^1^, LEBRETON Guillaume^1^, BARHOUM Pétra^1^, LEFÈVRE Lucie^1^, HEKIMIAN Guillaume^1^, LUYT Charles-Edouard^1^, COMBES Alain^1^, WINISZEWSKI Hadrien^4^, SCHMIDT Matthieu^1^

##### ^1^Hôpital de la Pitié Salpêtrière, Paris, France; ^2^Centre Hospitalier Régional Universitaire de Nancy, Nancy, France; ^3^Hôpital Nord, Marseille, France; ^4^Centre Hospitalier Régional Universitaire de Besançon, Besançon, France; ^5^Hôpital Bichat, Paris, France; ^6^Hôpital Tenon, Paris, France

###### Correspondence: Juliette CHOMMELOUX (juliette.chommeloux@gmail.com)

*Annals of Intensive Care* 2022, **12(1):**CO-48

**Rationale:** Mortality of COVID- related acute respiratory distress syndrome (ARDS) treated with ECMO did not differ from other ARDS in the first wave of the pandemic. However, ECMO duration and hospital length of stay were much longer. We aimed to assess the long-term quality of life (QoL) and mortality of patients COVID 19 who received ECMO.

**Patients and methods/Materials and methods:** Survivors after ECMO-treated ARDS from March to June 2020 in 7 French Intensive Care Unit (ICU) were followed up at 6 and 12 months after ECMO onset. Pulmonary (CT scan, pulmonary function test, St George score), physical (MRC and examination), and psychological were assessed. QoL was compared to the age-matched French population and patients with non-COVID ARDS treated with ECMO.

**Results:** Eighty out of 132 patients with ECMO were discharged alive during that period. Eighteen patients were lost to follow-up and were not included in our study. The 62 studied survivors were predominantly male, median age 47 [40–55] years, and have very few comorbidities except obesity (BMI 32 [28–36] kg/m2). ECMO and mechanical ventilation duration were 18 [11–25] and 36 [27–62] days, respectively. Besides, their ICU and hospital length of stay were 43 [33–62] and 85 [29–112] days. At 1 year, only one patient was still in the hospital. Pulmonary function tests were good at 6 months except for a persistent impairment of the DLCO. However, 75% of patients had fibrotic-like patterns on the CT scan at that time. QoL, assessed by the SF-36, was impaired compared with the French age-matched population, but also with non-COVID ARDS treated with ECMO. Noticeably, QoL did not improve at 1 year of follow-up. At 1 year, 44% and 42% of the patients had signs of anxiety and depression, respectively, whereas 42% of survivors had symptoms of post-traumatic stress disorder. Lastly, only 38% of these patients returned to their initial work at 1 year.

**Conclusion:** Contrary to non-COVID ARDS, the long-term QoL of COVID patients treated with ECMO was still severely impaired at one year. Besides, a very large proportion of survivors still complained about anxiety, depression, and post-traumatic stress symptoms. These results emphasize the importance to integrate these young patients into customized, patient-centered, rehabilitation programs after ICU discharge.

**Compliance with ethics regulations:** Yes in clinical research.
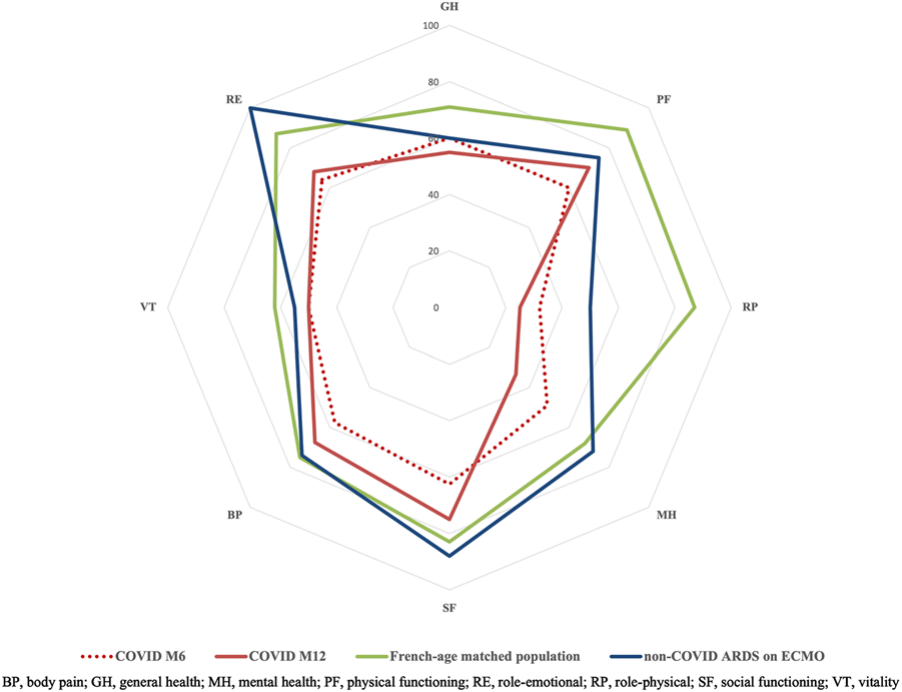


*Compared quality of life (SF-36) 6 and 12 months after ECMO for severe ARDS due to COVID 19 with non-COVID ARDS on ECMO and French-age matched population*

### CO-49 Late venovenous extracorporeal membrane oxygenation in patients with acute respiratory distress syndrome due to SARS-CoV-2

#### NATIVEL Mathilde^1^, PUECH Bérénice^1^, VALANCE Dorothée^1^, CALLY Radj^1^, DANGERS Laurence^1^, JABOT Julien^1^, BRAUNBERGER Eric^1^, NIGOLEAN Alexandru^1^, ALLYN Jérôme^1^, ALLOU Nicolas^1^, VIDAL Charles^1^

##### ^1^CHU Nord de La réunion, Saint-Denis, Reunion

###### Correspondence: Mathilde NATIVEL (mathildennativel@gmail.com)

*Annals of Intensive Care* 2022, **12(1):**CO-49

**Rationale:** Invasive mechanical ventilation (IMV) beyond 10 days is a relative contraindication to venovenous extracorporeal membrane oxygenation (VV-ECMO). Few studies have evaluated the prognosis of late VV-ECMO support in patients with acute respiratory distress syndrome (ARDS) due to SARS-CoV-2. The aim of our study was to evaluate the characteristics and prognosis of patients supported with late VV-ECMO for refractory ARDS due to SARS-CoV-2.

**Patients and methods/Materials and methods:** It was a multicenter, retrospective, observational study that evaluated all patients assisted with VV-ECMO between July 2020 and December 2021 for refractory ARDS related to SARS-CoV-2. We compared the characteristics of patients supported with late VV-ECMO (beyond 10 days of IMV) with those supported with early VV-ECMO (< 10 days).

**Results:** Over the study period, 467 patients were hospitalized in 4 intensive care units (ICU) for SARS-CoV-2 pneumonia. Among them 54 patients with a median age of 51 (42;57) years had required VV-ECMO support (12%). Of the 54 patients, 11 had late VV-ECMO (20%) with a median implantation time of 11 [11–14] days after the beginning of IMV (Table 1). Late implanted patients were less obese (p = 0.02), they tended to have less diabetes mellitus (p = 0.09) and to have a lower simplified acute physiology score (SAPS) II (p = 0.12). At the time of VV-ECMO implantation, significant differences were observed with higher lactatemia in the early implanted group (1.8 mmol/L vs. 1.2 mmol/L, p = 0.02) and higher capnia in the late VV-ECMO patients (67 mmHg vs. 53 mmHg, p < 0.01). Vasopressor support by norepinephrine was comparable between the two groups (p = 0.19). The in-ICU mortality was 56% without significant difference between the two groups (p = 1), even after adjustment for Respiratory Extracorporeal Membrane Oxygenation Survival Prediction (RESP) score (p = 0.64) or SAPS II (p = 0.99).

**Conclusion:** Late VV-ECMO implantation in patients with refractory ARDS related to SARS-CoV-2 does not appear to be associated with an excess risk of mortality, provided that patients were selected on their clinical characteristics at ICU admission (past medical history, SAPS II) and their severity at the time of implantation (associated organ failures). These results are consistent with other studies concerning SARS-CoV-2 associated ARDS [1,2]. The delay of IMV beyond 10 days should not be a contraindication to the implantation of VV-ECMO in patients with SARS-CoV-2 associated ARDS.

**Reference 1:** Olivier et al. Crit Care (2021) Prolonged time from intubation to cannulation in VV-ECMO for COVID-19: does it really matter? 25:385 https://doi.org/10.1186/s13054-021-03800-5.

**Reference 2:** Hermann et al. Annals of Intensive Care (2022) Duration of invasive mechanical ventilation prior to extracorporeal membrane oxygenation… 12:6 https://doi.org/10.1186/s13613-022-00980-3.

**Compliance with ethics regulations:** Yes in clinical research.
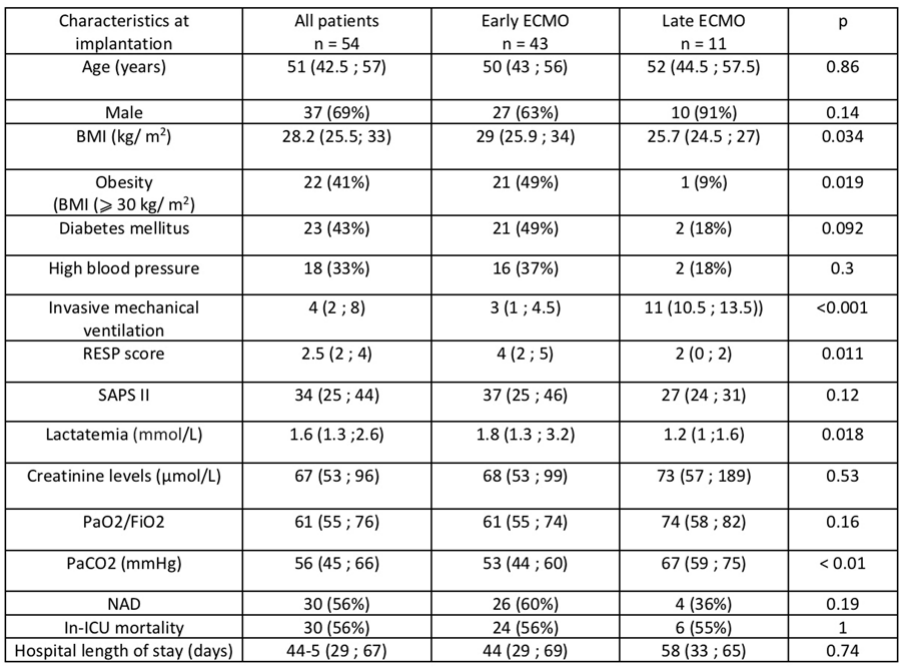


*Table 1: Selected characteristics at implantation of the whole population and univariate analyses between early (*< *10 days of mechanical ventilation) and late (beyond 10 days) VV-ECMO. Results are expressed as median ([Q25; 75] or number (%).*

### CO-50 DD ECMO: Prevalence and evolution of diaphragmatic dysfunction in patients with acute respiratory distress syndrome under veno-venous ECMO

#### GAUTIER Melchior^1^, JOUSSELIN Vincent^1^, DEMOULE Alexandre^1^, COMBES Alain^1^, SCHMIDT Matthieu^1^, DRES Martin^1^

##### ^1^Pitié Salpetrière, Paris, France

###### Correspondence: Melchior GAUTIER (melchiorgautier25@gmail.com)

*Annals of Intensive Care* 2022, **12(1):**CO-50

**Rationale:** ECMO is currently part of care in severe acute respiratory distress syndrome (ARDS). Its use is frequently associated with prolonged deep sedation and neuromuscular blockades, that may lead to diaphragm dysfunction. This latter is associated with delayed mechanical ventilation weaning and poor outcomes. Our study aims to assess the prevalence of diaphragmatic dysfunction in a large population of severe ARDS on veno-venous ECMO. We hypothesize that diaphragmatic dysfunction is frequent, severe, and associated with prolonged mechanical ventilation and poor outcomes in that severe population.

**Patients and methods/Materials and methods:** We conducted a prospective, observational study in two medical intensive care units. All patients with SARS-CoV-2 related ARDS requiring veno-venous ECMO were included from February 1 to September 31, 2021. Diaphragmatic function was daily assessed by measuring diaphragm pressure generation in response to phrenic nerve stimulation (Ptr,stim) from ECMO initiation until ECMO weaning.

**Results:** Sixty-three patients were included with a median age of 53 (42–59) years old and after a median of 4 days (2–6) of mechanical ventilation. Diaphragmatic dysfunction upon inclusion (at day 1 of ECMO) was present in 38 patients (60%). Patients with diaphragmatic dysfunction at day 1 were older (55 years [43–60] vs. 48 years [40–55], p = 0.042) and had a higher LUS (Lung Ultrasound score as a surrogate of lung aeration loss) (26 vs. 24, p = 0.037). Diaphragmatic function did not significantly change over the study period (Fig. 1). 24 patients had a successful ECMO weaning, sixteen patients (67%) had diaphragmatic dysfunction the day of ECMO weaning. Besides, these patients had longer mechanical ventilation duration when compared to those without diaphragmatic dysfunction (62.9 vs 41.7 days).

**Conclusion:** Diaphragmatic dysfunction is frequent in ARDS patients undergoing ECMO for SARS-CoV-2 infection. When present at ECMO day-1, diaphragmatic dysfunction did not seem to evolve over time. However, it was associated with a longer duration of mechanical ventilation in patients successfully weaned from ECMO.

**Compliance with ethics regulations:** Yes in clinical research.
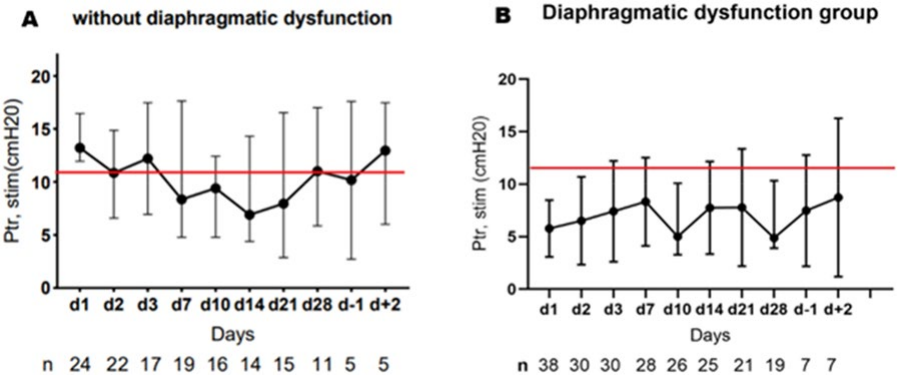


*Figure 1: Time course of diaphragmatic function from D1 to ECMO weaning. Values are presented as medians with their standard deviations. The red line corresponds to the threshold of 11 cmH*_*2*_ *O below which diaphragmatic dysfunction is defined.*

### CO-51 Impaired pharmacokinetics of amiodarone under veno-venous extracorporeal membrane oxygenation: from bench to bedside

#### LESCROART Mickael^1^, PRESSIAT Claire^2^, PEQUIGNOT Benjamin^1^, HÉBERT Jean-Louis^3^, ALSAGHEER Nassib^1^, SCALA BERTOLA Julien^1^, LEVY Bruno^1^

##### ^1^CHRU Nancy, Vandoeuvre, France; ^2^CHRU Henri Mondor, Créteil, France; ^3^Hopital Pitié Salpétrière, Institut de cardiologie, Paris, France

###### Correspondence: Mickael LESCROART (dr.lescroart@gmail.com)

*Annals of Intensive Care* 2022, **12(1):**CO-51

**Rationale:** Adjusting drug therapy under Veno-Venous Extra-Corporeal-Membrane-Oxygenation (VV-ECMO) is challenging. Albeit impaired pharmacokinetics (PK) under VV-ECMO have been reported for sedative drugs and antibiotics, data about amiodarone are lacking. We assessed the PK of amiodarone under VV-ECMO in vitro and in vivo using a porcine model of cardiac arrest ongoing CPR previously injured with ARDS rescued by VV-ECMO.

**Patients and methods/Materials and methods:** In vitro: closed loop ECMO were used for studying amiodarone adsorption over a 120 min period versus a recipient control. Amiodarone bolus (100 and 300 mg) were studied. In vivo: ARDS was induced in 10 pigs. Animals were randomly assigned to control or VV-ECMO groups. Amiodarone 300 mg was injected once CPR started and twelve blood samples were drawn over a 12 min period. Pharmacokinetic analysis was performed with non-linear mixed effects modelling.

**Results:** In vitro study revealed a significant decrease in amiodarone concentrations after 10 min and a loss of 99.6% of amiodarone concentrations after 120 min. In vivo pharmacokinetics revealed a significant decrease of Cmax, with 123.5 mg/L (109.5–150.0) versus 61.7 mg/L (55.3–80.3) in the control vs ECMO groups (p = 0.02), respectively without delaying time to peak concentration with 90 s (60–90) versus 90 s (75–90), respectively (p = 1). VV-ECMO significantly modified central distribution volume and amiodarone clearance. Monte-Carlo simulations predicted that amiodarone 600 mg bolus under VV-ECMO could achieve the AUC observed in the control group.

**Conclusion:** This is the first study reporting pharmacokinetics of amiodarone under VV-ECMO in an animal model. We found significant alterations of drug delivery. Higher amiodarone doses might be considered for efficient pharmacokinetics under VV-ECMO.

**Compliance with ethics regulations:** Yes in animal testing.
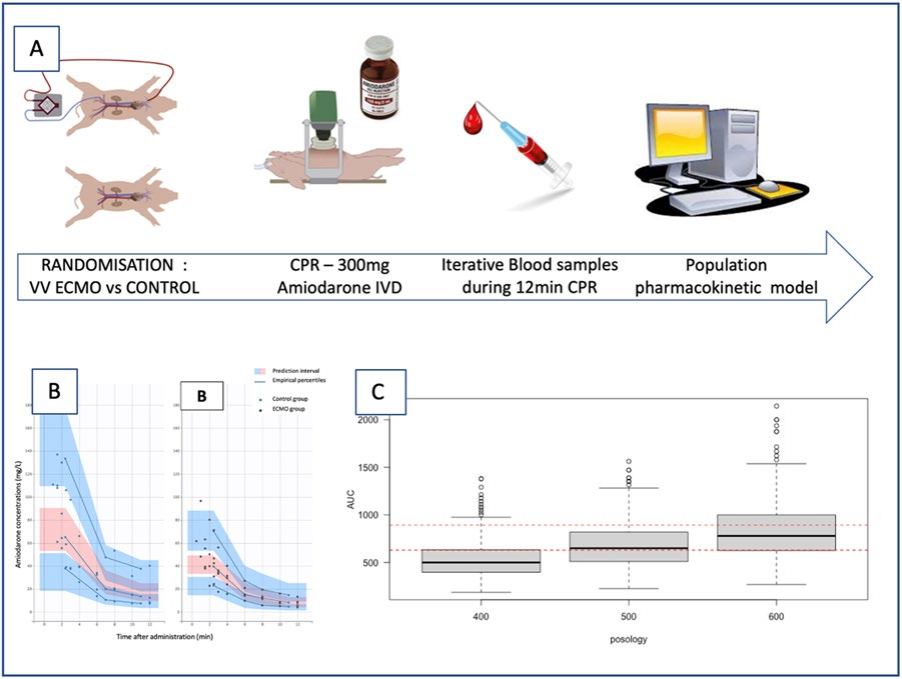


*Panel A: experimental protocol. Panel B: Visual Predictive Check for amiodarone model. Left panel: control group. right panel: ECMO group. Panel C: Monte Carlo simulations to reach amiodarone AUC Q1–Q3 in the control group.*

### CO-52 Increasing sweep gas flow through the membrane lung reduces dyspnea and respiratory drive in veno-arterial ECMO patients– The DysCO 2 study

#### BUREAU Côme^1^, SCHMIDT Matthieu^1^, NIERAT Marie-Cécile^1^, DANGERS Laurence^1^, CLERC Sébastien^1^, JOUSSELLIN Vincent^1^, MAYAUX Julien^1^, MORAWIEC Elise^1^, COMBES Alain^1^, SIMILOWSKI Thomas^1^, DEMOULE Alexandre^1^

##### ^1^Hôpital Universitaire Pitié-Salpêtrière, Paris, France

###### Correspondence: Côme BUREAU (come.bureau@aphp.fr)

*Annals of Intensive Care* 2022, **12(1):**CO-52

**Rationale:** Patients with severe heart failure may benefit from veno-arterial extracorporeal membrane oxygenation vaECMO, which preserves systemic blood flow. In addition, the vaECMO oxygenation membrane ensures blood oxygenation and CO_2_ removal. In clinical practice, vaECMO patients may exhibit dyspnea despite adequate blood flow and the absence of blood gas abnormalities. Our objective was to evaluate, in vaECMO patients exhibiting significant dyspnea, the impact of an increase in sweep gas flow through the vaECMO membrane on dyspnea.

**Patients and methods/Materials and methods:** Patients with (1) vaECMO for cardiogenic shock and (2) a dyspnea ≥ 40 mm on a visual analog dyspnea scale (Dyspnea-VAS) from zero to 100 mm were included. Four conditions were studied: on inclusion and after three sweep gas flow increments of two liters per minute each. Dyspnea was assessed with the Dyspnea-VAS, the A1 score of the Multidimensional Dyspnea Profile and the Intensive Care Respiratory Distress Operating Scale (IC-RDOS). The respiratory drive was concomitantly assessed by the measure of the electromyographic activity of the Alea Nasi and parasternal muscles.

**Results:** We included 21 non-mechanically ventilated patients. Median (interquartile range) age was 40 years (30–55), 62% male and duration of ECMO was 3 days (2–4). Dyspnea-VAS was 50 (45–60) mm. Weinberg radiological pulmonary oedema score was 3 (0–5). Gas flow at inclusion was 1 L/min (0.5–2). Table 1 shows respiratory rate, PaCO_2_, Dyspnea-VAS, A1 score and IC-RDOS across the four conditions. PaCO_2_ decreased in response to the 2-L-per-minute increase in sweep, but it ceased to decrease after 6 L. Dyspnea did not decrease immediately but was significantly lower after 6 L of increased sweep regardless of the assessment score. The electromyographic activity of Alea nasi and parasternal muscles decreased significantly after sweep gas flow increment. There was a significant inverse correlation between the Dyspnea-VAS and the sweep gas flow (Rho = − 0.68, p < 0.0001) but not between Dyspnea-VAS and PaCO_2_ (Rho = 0.136, p = 0.236).

**Conclusion:** In critically ill patients with vaECMO, incrementation of sweep gas flow through the oxygenation membrane decreases dyspnea. It might be mediated by a decrease in respiratory drive, as suggests the concomitant decrease in respiratory rate and electromyographic activity of respiratory muscles.

**Compliance with ethics regulations:** Yes in clinical research.
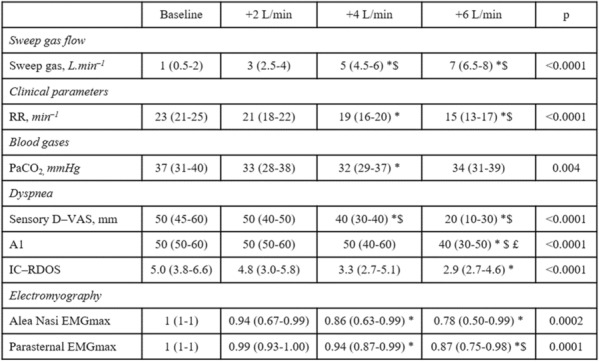


*p* < *0.05 compared to the Baseline condition, $ p* < *0.05 compared to the *+ *2 condition, £ p* < *0.05 compared to the *+ *4 condition*

### CO-53 Amniotic fluid embolism rescued by venoarterial extracorporeal membrane oxygenation

#### AISSI JAMES Sarah ^1^, KLEIN Thomas^2^, LEBRETON Guillaume ^1^, NIZARD Jacky^1^, CHOMMELOUX Juliette^1^, BRECHOT Nicolas^1^, PINETON DE CHAMBRUN Marc^1^, HEKIMIAN Guillaume^1^, LUYT Charles-Edouard^1^, LEVY Bruno^2^, KIMMOUN Antoine^2^, COMBES Alain^1^, SCHMIDT Matthieu^1^

##### ^1^Hôpital Pitié Salpêtrière, Paris, France; ^2^CHRU, Nancy, France

**Correspondence:** Sarah AISSI JAMES (sarah.aissi@live.fr)

*Annals of Intensive Care* 2022, **12(1):**CO-53

**Rationale:** Amniotic fluid embolism (AFE) is a rare but often catastrophic complication of pregnancy. The cardiopulmonary dysfunction associated with AFE being typically self-limited, venoarterial extracorporeal membrane oxygenation (VA-ECMO) support has been reported in the most severe forms. Data available on AFE rescued by VA-ECMO were mainly described in single-case reports and concern can be raised about the bleeding risks with ECMO in that context. The objectives of this retrospective study were to report outcomes of ECMO-treated AFE; to describe their critical care management and in-ICU complications; and to report long-term maternal health-related quality of life (HRQOL).

**Patients and methods/Materials and methods:** This study included patients with AFE, according to Clark diagnostic criteria (1), rescued by VA-ECMO and hospitalized in two ECMO centers between August 2008 and February 2021. Clinical characteristics, peri-delivery resuscitative procedures and critical care management are detailed. Main outcome variables included survival to ICU discharge, days under ECMO therapy, ECMO-associated complications, time on mechanical ventilation, ICU length of stay. ICU survivors were assessed for long term HRQOL during a phone interview in May 2021 by completing the Short-Form 36 questionnaire and the Impact of Event Scale screening for Post-Traumatic Stress Disorder (PTSD)-related symptoms.

**Results:** During that 13-year study period, 10 patients with AFE were treated with VA-ECMO. Seven patients had a cardiac arrest before ECMO and two were cannulated under cardiopulmonary resuscitation. Pre-ECMO hemodynamic was severely impaired with an inotrope score at 370 (55–1530) μg/kg/min, a severe left ventricular ejection fraction at 14 (0–40) %, lactate at 12 (2–30) mmol/L, SAPS II at 69 (56–81) and massive blood transfusion requirement. 70% of these patients were alive at hospital discharge, 50% reported ECMO-related complications. Median durations of ECMO and mechanical ventilation support were respectively 4 (1–6) and 5 (1–13) days. The median ICU length of stay was 12 (1–25) days. All infants survived. HRQOL was lower than age-matched controls and still profoundly impaired in the role-physical, bodily pain, and general health components after a median of 44 months follow-up (Fig. 1). Four out of seven patients returned to their initial work.

**Conclusion:** In this rare per-delivery complication, our results support the use of VA-ECMO despite extreme initial severity, intense disseminated intravascular coagulation and ongoing bleeding. However, long-term physical and mental status were still impaired after long-term evaluation. Future studies should therefore focus on customized, patient-centered, rehabilitation programs to improve HRQOL.

**Reference 1:** Clark SL, Romero R, Dildy GA, Callaghan WM, Smiley RM, Bracey AW, et al. Proposed diagnostic criteria for the case definition of amniotic fluid embolism in research studies. Am J Obstet Gynecol. 2016;215:408–12.

**Compliance with ethics regulations:** Yes in clinical research.
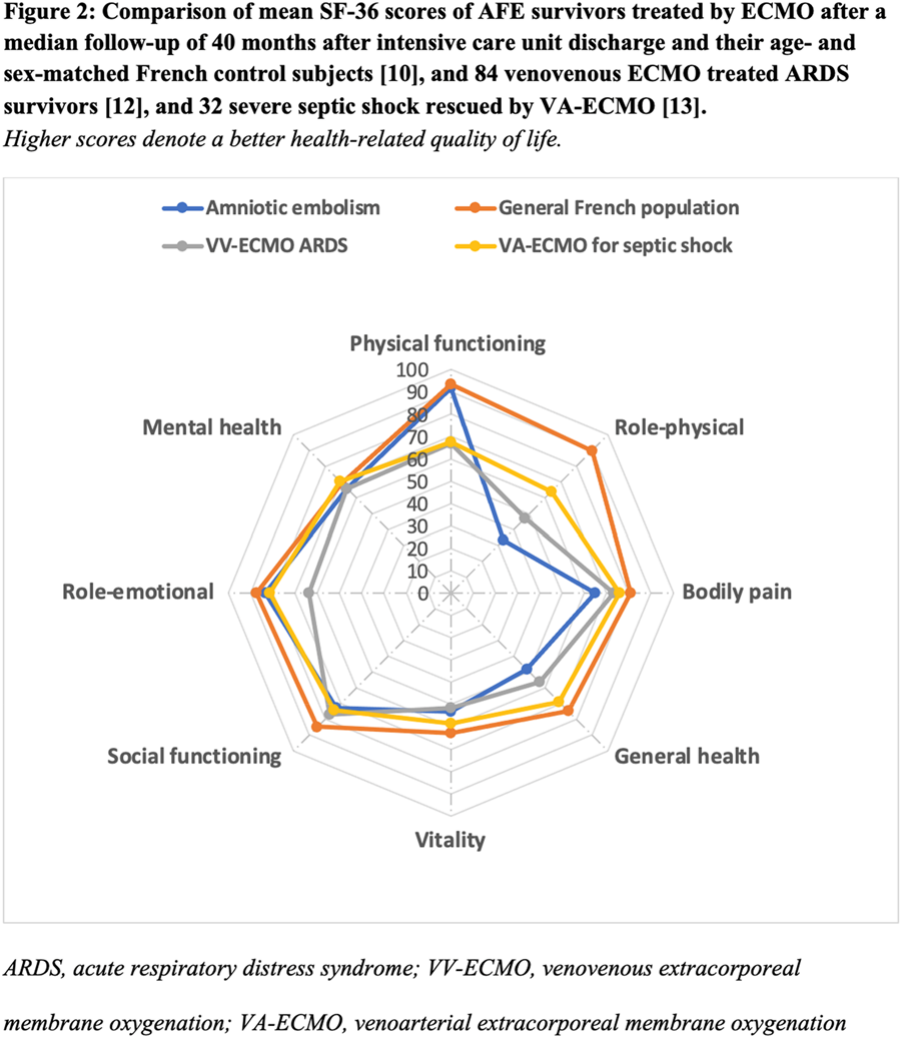


*Comparison of SF-36 scores of AFE survivors treated by ECMO after a median follow-up of 40 months after ICU discharge and their age- and sex-matched control subjects, 84 venovenous ECMO-treated ARDS survivors, and 32 septic shock rescued by VA-ECMO*

### CO-54 Extracorporeal life support allows lung transplant in anti-MDA5+ rapidly progressive-interstitial lung disease

#### BAY Pierre^1^, PINETON DE CHAMBRUN Marc^2^, ROUX Antoine^4^, BUNEL Vincent^6^, COMBES Alain^2^, ISRAËL BIET Dominique ^5^, ZUBER Benjamin^4^, NUNES Hilario^3^, ALLENBACH Yves^2^, UZUNHAN Yurdagul^3^

##### ^1^Hopital Henri Mondor, Creteil, France; ^2^Hopital Pitié Salpétrière, Paris, France; ^3^Hopital Avicenne, Paris, France; ^4^Hopital Foch, Suresnes, France; ^5^Hopital Européen Georges Pompidou, Paris, France; ^6^Hopital Bichat, Paris, France

###### Correspondence: Pierre BAY (pierrebay53@yahoo.fr)

*Annals of Intensive Care* 2022, **12(1):**CO-54

**Rationale:** Anti-melanoma differentiation-associated gene 5 antibody (anti-MDA5) dermatomyositis (DM) is a rare subtype of idiopathic inflammatory myopathy, associated with severe interstitial lung disease (ILD). A subset of anti-MDA5 DM patients with rapidly progressive ILD (RP-ILD) have a very poor prognosis with reported mortality rates reaching 80–84%. The use of extracorporeal life support (ECLS) is questionable, as reported in several studies that emphasize the futility of a bridge-to-recovery strategy. In this respect, emergency lung transplantation of previously unlisted patients on ECLS is under debate.

**Patients and methods/Materials and methods:** This French, multicenter, retrospective study, conducted from 2013 to 2021 included all patients with anti-MDA5 DM RP-ILD requiring ECLS.

**Results:** Fifteen patients requiring ECLS were included in the study: venovenous -ECMO n = 13, ECCO2R n = 1 and venoarterial-ECMO n = 1. The female-to-male ratio was 4 and the age at ICU admission was a mean of 50 [32–67] years. Two patients received ECLS support (1 ECMO VA, 1 ECMO VV) as a bridge-to-transplantation strategy and both underwent ECLS before mechanical ventilation. Five patients underwent lung transplantation after a median of 8 [4-20] days on ECMO, none previously listed for a lung transplantation. After a median follow-up of 25 [3–93] months, all transplanted patients were alive at the conclusion of the study (four discharged home, one still hospitalized) and no relapse of DM or ILD was noted. All other patients, not listed for lung transplantation, died after a median of 30 [4–52] days on ECMO (Fig. 1).

**Discussion:** The prognosis of anti-MDA5 RP-ILD seems inevitably poor despite aggressive immunosuppression and the use of ECLS. The results presented here provide crucial information for the management of anti-MDA5 RP-ILD. First, we highlight the refractory nature of the anti-MDA5 RP-ILD requiring ECLS. No anti-MDA5 RP-ILD patient, irrespective of the treatment regimen they receive, including the most recent biologics such as JAK inhibitors, should be weaned from the ECLS. Second, every patient that could be bridge-to-transplantation was discharged alive from ICU. None was previously listed for lung transplantation. Emergency lung transplantation was possible in patients treated with vasopressors, mechanical ventilation and ECLS.

**Conclusion:** The bridge-to-recovery strategy in anti-MDA5 RP-ILD patients requiring ECLS despite specific prior treatment leads to undesirable results, pointing to the drawbacks of this approach. In contrast, a bridge-to-emergency lung transplantation is not only feasible, but also associated with a favorable outcome and appears therefore as the sole hope of survival for patients requiring ECLS.

**Reference 1:** Vuillard C, Pineton de Chambrun M, de Prost N, Guérin C, Schmidt M, Dargent A, et al. Clinical features and outcome of patients with acute respiratory failure revealing anti-synthetase or anti-MDA-5 dermato-pulmonary syndrome: a French multicenter retrosp.

**Reference 2:** Rubin J, Black KE, Hallowell RW, Witkin AS, Lydston M, Shelton K, et al. Veno-Venous Extracorporeal Membrane Oxygenation (ECMO) for Myositis-Associated Rapidly Progressive Interstitial Lung Disease (RP-ILD). CHEST [Internet]. 2021 Jul 16 [cited 2021 Jul 1].

**Compliance with ethics regulations:** Yes in clinical research.
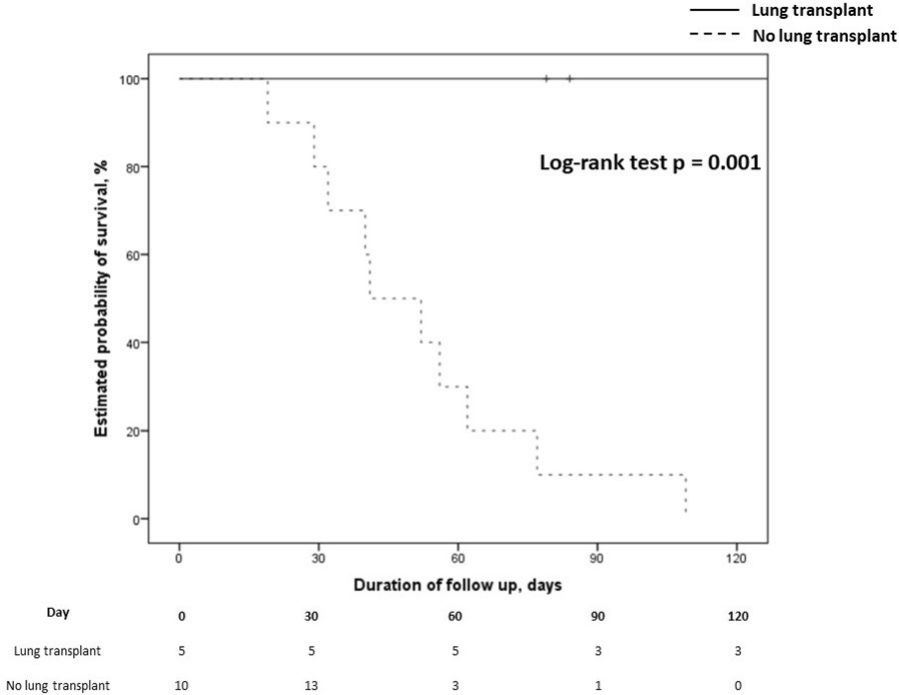


*Kaplan–Meier 120-days Survival Estimates according to lung transplantation status*

### CO-55 Trends in clinical characteristics and outcomes of Covid-19 critically ill adult patients in France: a national database study

#### NAOURI Diane^6^, BEDUNEAU Gaetan^4^, DRES Martin^2^, KIMMOUN Antoine^5^, COMBES Alain^2^, DEMOULE Alexandre^2^, MERCAT Alain^3^, PHAM Tai^2^, SCHMIDT Matthieu^2^, JAMME Matthieu^1^

##### ^1^Hopital privé de l'ouest parisien, Trappes, France; ^2^Assistance publique hopitaux de Paris, Paris, France; ^3^CHU Angers, Angers, France; ^4^CHU Rouen, Rouen, France; ^5^CHU Nancy, Nancy, France; ^6^Direction de la recherche, des études, de l'évaluation et des statistiques (DREES), Paris, France

###### Correspondence: Matthieu JAMME (mat.jamme@gmail.com)

*Annals of Intensive Care* 2022, **12(1):**CO-55

**Rationale:** Data concerning the association between time-period of ICU hospitalization and prognosis of patients admitted for coronavirus disease 2019 (COVID-19) are scarce. We aimed to describe the characteristics and the outcomes over time of critically ill patients cared in ICU for COVID-19.

**Patients and methods/Materials and methods:** We conducted a retrospective cohort study using the French administrative health care database (Système National des Données de Santé, SNDS). All adult patient hospitalized in French ICUs from March 1, 2020, to June 30, 2021 with at least one ICD-10 diagnosis code of COVID-19 was included. Three time-period corresponding to surge of COVID-19 ICU admission were defined: the first one between March 1, and June 30, 2020; the second between July 1, and December 31, 2020; and the third from January 1, to June 30, 2021. Risk factors of mortality and invasive mechanical ventilation (iMV) were identified by multivariate logistic regression models.

**Results:** 105979 COVID-19 patients were admitted in ICU during the study period with respectively 25150, 32689, and 48140 patients during the first, second, and third surge. First surge was remarkable with the highest proportion of iMV (42%, 32% and 31% for respectively surge 1, 2 and 3; p < 0.001), of renal replacement therapy (RRT) (9%, 7% and 5% for respectively surge 1, 2 and 3; p < 0.001) and use of vasopressors (36%, 26% and 24% for respectively surge 1, 2 and 3; p < 0.001). Second wave was composed with older patients (respectively 50% of more than 70 years vs 41% for wave 1 and 3) and higher IGS-2 score at ICU admission (33 [26–42]). Multivariate model identified age, male gender, Charlson score, IGS 2 score and ICU admission after the first wave as risk factors of in-hospital death. After stratification on age, the odds ratios of in-hospital death were higher only in patients older than 70 years for the third wave (aOR = 1.30; 95% CI = 1.23–1.37) and the second wave (aOR = 1.15; 95%CI = 1.08–1.22), compared to those younger than 69 years old. During the third wave, 4971 (9%) patients had been vaccinated at ICU admission. Vaccination was associated with a lower likelihood of invasive mechanical ventilation (OR = 0.50, 95% CI = 0.40–0.63) and death (OR = 0.74, 95% CI = 0.60–0.90).

**Conclusion:** Between March 2020 and July 2021, we reported a decline in iMV, vasopressors and RRT use for ICU patients admitted for COVID-19. Second and third wave were associated with in-hospital mortality for patients with 70 years and more. Finally, vaccination was associated with a lower likelihood of iMV and death.

**Compliance with ethics regulations:** Yes in clinical research.

### CO-56 Comparison of SARS-CoV-2 variants of concern Alpha (B.1.1.7) versus Beta (B.1.351) in critically ill patients: a multicenter cohort study in the northeast of France

#### LOUIS Guillaume^1^, BELVEYRE Thibaut^2^, GIBOT Sebastien^3^, GOETZ Christophe^5^, DUNAND Paul^4^, GACI Rostane^1^, CONRAD Marie^3^, CADOZ Cyril^1^, GETTE Sebastien^1,4^, PEREZ Pascale^6^, OUAMARA Nadia^5^, PICARD Yoann^1^, MELLATI Nouchan^1^

##### ^1^Intensive Care Unit, Metz-Thionville Regional Hospital, Mercy Hospital, Metz, France, Metz, France; ^2^Department of Anesthesiology and Intensive Care Medicine, University Hospital of Nancy, Vandoeuvre-Lès-Nancy, France, Vandoeuvre-Les-Nancy, France; ^3^Medical Intensive Care Unit, University Hospital of Nancy, Nancy, France, Nancy, France; ^4^Intensive Care Unit, Metz-Thionville Regional Hospital, Bel Air Hospital, Thionville, France, Thionville, France; ^5^Clinical research support Unit, Metz-Thionville Regional Hospital, Mercy Hospital, Metz, France, Metz, France; ^6^Department of Virology, Metz-Thionville Regional Hospital, Mercy Hospital, Metz, France, Metz, France

###### Correspondence: Guillaume LOUIS (gus_louis@yahoo.fr)

*Annals of Intensive Care* 2022, **12(1):**CO-56

**Rationale:** The clinical outcomes of the Beta (B.1.351) variant of concern (VOC) of the SARS-CoV-2 virus remain poorly understood. In early 2021, northeastern France experienced an outbreak of Beta that was not observed elsewhere. This outbreak slightly preceded and then overlapped with a second outbreak of the better understood VOC Alpha (B.1.1.7) in the region. This situation allowed us to contemporaneously compare Alpha and Beta in terms of the characteristics, management, and outcomes of critically ill patients.

**Patients and methods/Materials and methods:** A multicenter cohort study was conducted on all consecutive adult patients who had laboratory confirmed SARS CoV-2 infection, underwent variant screening, and were admitted to one of four intensive care units for acute respiratory failure between January 9th and May 15th 2021. Primary outcome was 60-day mortality. Differences between Alpha and Beta in terms of other outcomes, patient variables, management, and vaccination characteristics were also explored by univariate analysis. The factors that associated with 60-day death in Alpha- and Beta-infected patients were examined with logistic regression analysis.

**Results:** In total, 333 patients (median age, 63 years) were enrolled. Of these, 174 and 159 had Alpha and Beta, respectively. The two groups did not differ significantly in terms of 60-day mortality (19% vs. 23%), need for mechanical ventilation (60% vs. 61%), mechanical ventilation duration (14 vs. 15 days), other management variables, patient demographic variables, comorbidities, or clinical variables on ICU admission. The vast majority of patients were unvaccinated (94%). The remaining 18 patients had received a partial vaccine course and 2 were fully vaccinated. The vaccinated patients were equally likely to have Alpha and Beta.

**Discussion:** To date, this is one of the largest studies to compare the characteristics and outcomes of critically ill patients who were infected with either of two simultaneously circulating VOCs and who underwent similar treatment regimens in the same settings. A recent meta-analysis suggests an increased mortality of Beta variant compared to wild type strain. Plus, the Beta and Delta variants were described as risker than the Alpha and Gamma variants (1). Nevertheless, none of the studies cited in the meta-analysis was specific to critically ill patient or compare variants with each other. Plus, the Beta variant proportion was quite low compared to other variants in these studies (Alpha particularly).

**Conclusion:** Beta did not differ from Alpha in terms of patient characteristics, management, or outcomes in critically ill patients. Better understanding of these variants including ongoing and future ones is essential.

**Reference 1:** Lin L, Liu Y, Tang X, He D. The Disease Severity and Clinical Outcomes of the SARS-CoV-2 Variants of Concern. Frontiers in Public Health (2021) 9: https://www.frontiersin.org/article/10.3389/fpubh.2021.775224 [Accessed January 26, 2022].

**Compliance with ethics regulations:** Yes in clinical research.
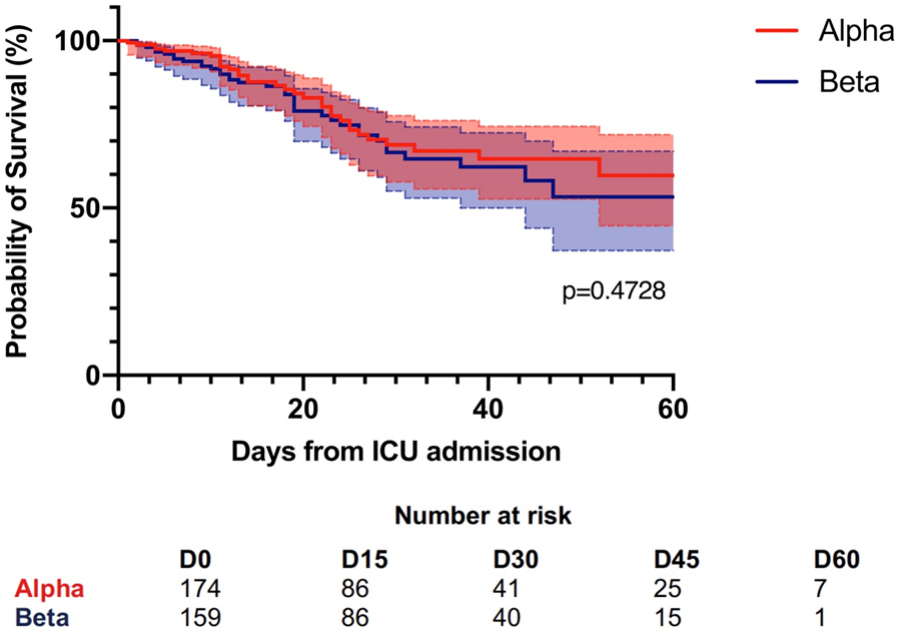


*Kaplan–Meier survival curves of the patients who were admitted to an ICU for an infection with Alpha or Beta (n* = *333).*

### CO-57 Critically ill patients with SARS-Cov2 pneumonia after vaccination: a multicenter cohort

#### FRIOL Alice^1^, MOREAU Anne-Sophie^4^, JUNG Boris^5^, JULLIEN Edouard^6^, BUREAU Côme^7^, DJIBRÉ Michel^8^, DE PROST Nicolas^9^, ZAFRANI Lara^10^, ARGAUD Laurent^11^, REUTER Danielle^12^, CALVET Laure^13^, DE MONTMOLLIN Etienne^14^, BENGHANEM Sarah^15^, PICHEREAU Claire^16^, PHAM Tai^17^, CACOUB Patrice^2^, BIARD Lucie^3^, SAADOUN David^2^, MIROUSE Adrien^2^

##### ^1^Institut Cochin - INSERM U1016 - CNRS UMR 8104 - Université de Paris, APHP, Paris, France; ^2^Département de Médecine Interne et Immunologie Clinique - hôpital Pitié Salpêtrière, APHP, Paris, France; ^3^Département de biostatistiques, hôpital Saint-Louis, APHP, Paris, France; ^4^Service de réanimation polyvalente, CHRU de Lille - hôpital Roger Salengro, Lille, France; ^5^Service de médecine intensive réanimation, CHU de Montpellier, Montpellier, France; ^6^Service de médecine intensive réanimation, hôpital Ambroise Paré, APHP, Boulogne, France; ^7^Service de médecine intensive réanimation, hôpital Pitié-Salpêtrière, APHP, Paris, France; ^8^Service de médecine intensive réanimation, hôpital Tenon, APHP, Paris, France; ^9^Service de médecine intensive réanimation, hôpital Henri Mondor, APHP, Créteil, France; ^10^Service de médecine intensive réanimation, hôpital Saint-Louis, APHP, Paris, France; ^11^Service de médecine intensive réanimation, hospices civils de Lyon, Lyon, France; ^12^Service de réanimation polyvalente, CH Sud-Francilien, Corbeil-Essonnes, France; ^13^Service de médecine intensive réanimation, CHU de Clermont-Ferrand, Clermont-Ferrand, France; ^14^Service de médecine intensive réanimation, hôpital Bichat, APHP, Paris, France; ^15^Service de médecine intensive réanimation, hôpital Cochin, APHP, Paris, France; ^16^Service de réanimation polyvalente, CH Pontoise, Pontoise, France; ^17^Service de médecine intensive réanimation, hôpital de Bicêtre, Paris, France

###### Correspondence: Alice FRIOL (alice.friol@aphp.fr)

*Annals of Intensive Care* 2022, **12(1):**CO-57

**Rationale:** Vaccination reduces risk of infection, hospitalization and death due to SARS-Cov2. Vaccinated patients may experience severe SARS-Cov2 disease.

**Patients and methods/Materials and methods:** Multicenter cohort study of patients with severe SARS-Cov2 disease admitted in 15 intensive care units in France between January and September 2021. Vaccinated patients were compared to a large European cohort of wave 2/3 critically ill SARS-Cov2 patients.

**Results:** One hundred patients (68 (68%) men, median age 64 [57–71]) were included. Immunosuppression was reported in 38 (38%) patients. Among available serology at intensive care unit (ICU) admission, 64% exhibited an optimal antibody level. Median SOFA score at ICU admission was 4 [4–6.25] and median PaO_2_/FiO_2_ ratio was 84 [69–128] mmHg. High flow nasal oxygen and non-invasive mechanical ventilation were implemented in 79 (79%) and 18 (18%) patients, respectively. Mechanical ventilation was initiated in 48 (48%) with a median duration of 11 [5–19] days. During a median ICU length-of-stay of 8 [4–20] days, 31 (31%) patients died. Age (OR 1.07 CI95% [1.00–1.13] per year, p = 0.035), and SOFA at ICU admission (OR 1.40 CI95% [1.14–1.72] per point, p = 0.002) were independently associated with mortality. Vaccinated patients exhibited less frequently diabetes (16 [16%] vs. 351 [27%], p = 0.029) but more frequently immunosuppression (20 [20%] vs. 109 (8.3%), p < 0.0001), chronic kidney disease (24 [24%] vs. 89 (6.8%), p < 0.0001), chronic heart failure (16 [16%] vs. 58 [4.4%], p < 0.0001), and chronic liver disease (3 [3%] vs. 8 [0.6%], p = 0.04) compared to unvaccinated patients. Despite similar severity, vaccinated patients required less frequently invasive mechanical ventilation at ICU-day 1 and during ICU stay (23 [23%] vs. 785 [59.7%], p < 0.0001, and 48 [48%] vs. 930 [70.7%], p < 0.0001, respectively). There was no difference concerning ICU mortality (31 [31%] vs. 379 [28.8%], p = 0.76).

**Conclusion:** Severe SARS-Cov2 infection occurs post-vaccination essentially in patients with immunosuppression, chronic kidney, heart or liver failure. Age and disease severity are independently associated with mortality. Vaccination might inflect the disease course, even in critically-ill patients.

**Compliance with ethics regulations:** Yes in clinical research.

### CO-58 Auto-antibodies against type I interferons in critically ill COVID-19 patients: a prospective multicentre study

#### ARRESTIER Romain^1,2,3^, BASTARD Paul^4^, BELMONDO Thibault^5^, VOIRIOT Guillaume^6^, URBINA Tomas^7^, LUYT Charles-Edouard^8,9^, BELLAÏCHE Raphaël^10^, PHAM Tai^11^, AIT-HAMOU Zakaria^12^, ROUX Damien^13^, CLERE-JEHL Raphaël^14^, AZOULAY Elie^14^, GAUDRY Stéphane^15^, MAYAUX Julien^16^, MONCOMBLE Elsa^1,2^, PARFAIT Mélodie^1^, MEKONTSO-DESSAP Armand^1,2,3^, SEGAUX Lauriane^3,17^, CANOUI-POITRINE Florence^3,17^, CASANOVA Jean-Laurent^4^, HUE Sophie^3,5^, DE PROST Nicolas^1,2,3^

##### ^1^Service de Médecine Intensive Réanimation, Hôpitaux Universitaires Henri Mondor, Assistance Publique-Hôpitaux de Paris, Créteil, France; ^2^Groupe de Recherche Clinique CARMAS, Faculté de Santé de Créteil, Université Paris Est Créteil, France; ^3^INSERM, IMRB, Université Paris Est Créteil, Créteil, France; ^4^Laboratory of Human Genetics of Infectious Diseases, Necker Branch, INSERM U1163, Necker Hospital for Sick Children, Paris, France; Imagine Institute, University of Paris, Paris, France; St. Giles Laboratory of Human Genetics of Infectious Diseases, Rocke, New York, Etats-Unis; ^5^Département d'Hématologie et d'Immunologie Biologiques, Assistance Publique-Hôpitaux de Paris, Groupe Hospitalo-Universitaire Chenevier Mondor, Créteil, France; ^6^Service de Médecine Intensive-Réanimation, Hôpital Tenon, Assistance Publique-Hôpitaux de Paris, Paris, France; ^7^Service de Médecine Intensive-Réanimation, Hôpital Saint-Antoine, Assistance Publique-Hôpitaux de Paris, France; ^8^Service de Médecine Intensive Réanimation, Sorbonne Université, Hôpitaux Universitaires Pitié Salpêtrière-Charles Foix, Assistance Publique-Hôpitaux de Paris (AP-HP), Paris, France; ^9^INSERM UMRS_1166-iCAN, Institute of Cardiometabolism and Nutrition, Paris, France; ^10^Service d'Anesthésie-Réanimation Chirurgicale, Assistance Publique-Hôpitaux de Paris, Hôpitaux Universitaires Henri Mondor, Créteil, France; ^11^Service de Médecine Intensive-Réanimation, AP-HP, Hôpital de Bicêtre, DMU 4 CORREVE Maladies du Cœur et des Vaisseaux, FHU Sepsis, Groupe de Recherche Clinique CARMAS, Le Kremlin-Bicêtre, France; ^12^Service de Médecine Intensive-Réanimation, Hôpital Cochin, Assistance Publique-Hôpitaux de Paris (AP-HP). Centre & Université de Paris, Paris, France; ^13^Médecine Intensive Réanimation, AP-HP, Hôpital Louis Mourier, DMU ESPRIT, Colombes, France; ^14^Service de médecine intensive et réanimation, Hôpital Saint-Louis, Assistance Publique Des Hôpitaux de Paris, Paris, France; ^15^Département de réanimation médico-chirurgicale, APHP Hôpital Avicenne, Bobigny, France; ^16^Groupe Hospitalier Pitié Salpêtrière, Assistance Publique Hôpitaux de Paris, Service de Pneumologie et Réanimation Médicale, Paris, France; ^17^Unité de Recherche Clinique AP-HP, Hôpitaux Henri-Mondor, Créteil, France; ^18^Service de Médecine intensive Réanimation, APHP.Sorbonne Université, Hôpital Pitie Salpêtriere, Paris, France

###### Correspondence: Romain ARRESTIER (romain.arrestier@aphp.fr)

*Annals of Intensive Care* 2022, **12(1):**CO-58

**Rationale:** SARS-CoV-2 infection leads to a broad spectrum of symptoms with a large inter-individual variability. Impaired interferon (IFN) type I response seems to be involved in patients with severe SARS-CoV-2 infection. Auto-antibodies (auto-Abs) neutralizing type I IFN-α2 and IFN-ω were found in 10% of severe COVID-19 cases compared with 0% in mildly or asymptomatic cases. Dertermining whether auto-Abs neutralizing type I IFNs are associated with outcomes in critically ill patients with COVID-19 could lead to individualized therapeutic interventions.

**Patients and methods/Materials and methods:** We conducted a prospective multicentre study including 11 intensive care units (ICU). Patients hospitalized in ICU with proven SARS-CoV-2 infection and acute respiratory failure requiring oxygen or mechanical ventilation support were included. Our objectives were to compare the mortality of patients with versus without auto-Abs neutralizing type I IFNs, to assess the rate of positivity of auto-Abs and the factors associated with their positivity.

**Results:** 925 critically ill COVID-19 patients were included in the study between March 2020 and May 2021. Auto-Abs neutralizing type I IFN were found in 96 patients (10.3%): 78.3% had auto-Abs against IFN-α2, 74% against IFN-ω and 12.5% against IFN-β. Baseline characteristics did not differ between patients with and without auto-Abs (Table 1). At ICU admission, positive patients required a higher FiO_2_ (100% (70–100) vs 90% (60–100), p = 0.01), and more frequently met the diagnosis criteria for the acute respiratory distress syndrome (92.7% vs 77.6%, p = 0.0005). Mortality at day 28 was not different between groups (18.7% vs 23.7%, p = 0.279). In multivariable analysis, age (adjusted odds ratio (aOR) = 1.06 [1.04–1.08], p < 0.001), SOFA score (aOR = 1.18 [1.12–1.23], p < 0.001) and immunosuppression (aOR = 1.82 [1.1–3.0], p = 0.02) were associated with 28-days mortality, but auto-Abs positivity was not (aOR = 0.69 [0.38–1.26], p = 0.23). There was a non-significant trend towards a higher proportion of men among positive patients (78 vs 70%) contrasting with the previous finding that auto-Abs were almost uniquely detected in men (94%)^1^. Compared to women without auto-Abs, positive women were significantly younger (45 (24–62) vs 60 years (45.5–67.5)) and more frequently displayed an auto-immune background with more frequent positive anti-nuclear antibody (28% vs 4%, p = 0.003). They required more frequent invasive mechanical ventilation (71% vs 47%, p = 0.04) at ICU admission but 28-days mortality was not different.

**Conclusion:** In ICU patients, auto-Abs against type I IFNs are present in 10% of patients but are not associated with higher 28 days mortality. Positive women seem to have an auto-immune background and more frequently required mechanical ventilation.

**Reference 1:** Bastard, P. et al. Autoantibodies against type I IFNs in patients with life-threatening COVID-19. Science 370, eabd4585 (2020).

**Compliance with ethics regulations:** Yes in clinical research.
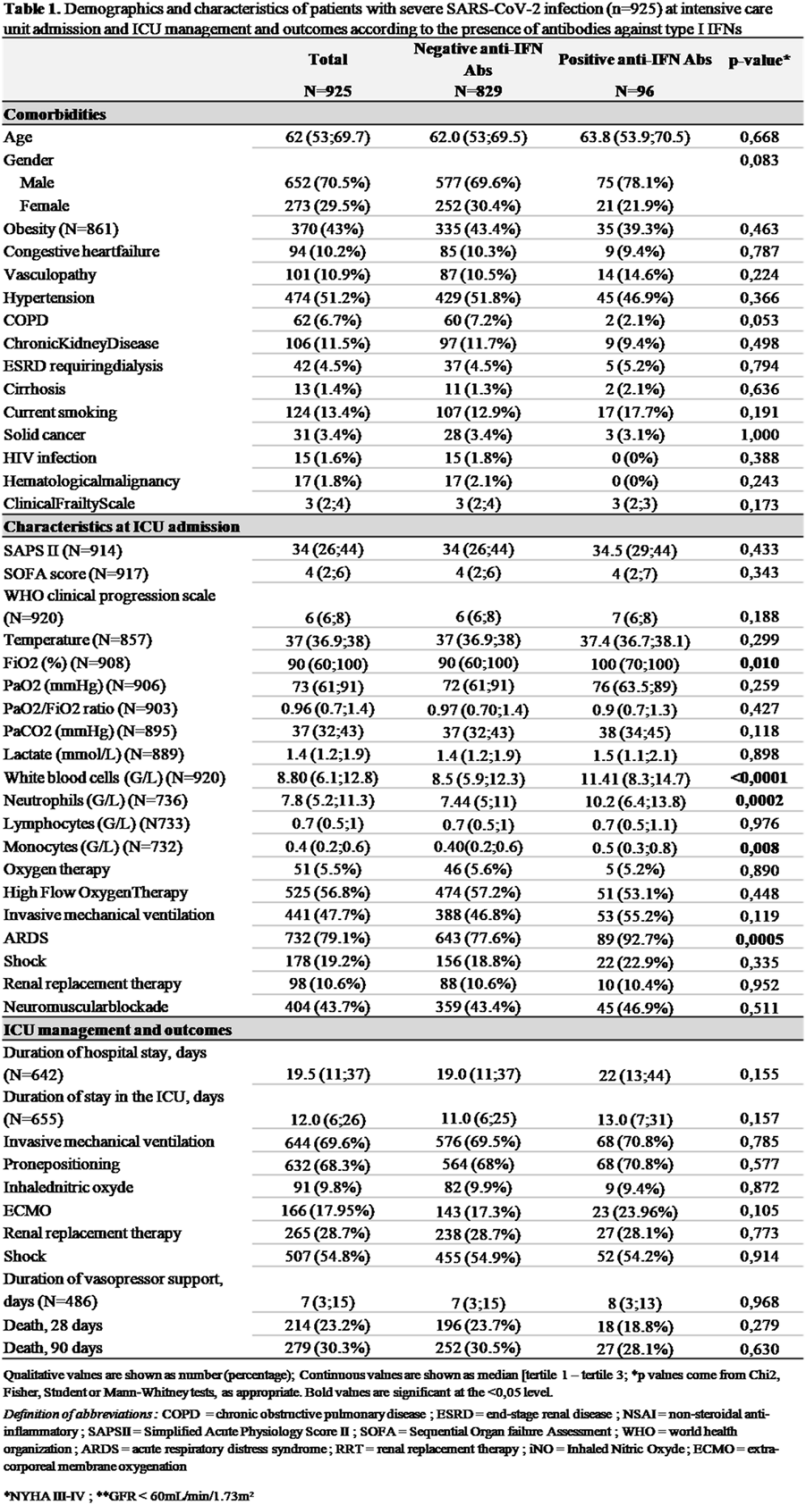


*Demographics and characteristics of patients with severe SARS-CoV-2 infection (n* = *925) at intensive care unit admission and ICU management and outcomes according to the presence of antibodies against type I IFNs*

### CO-59 Monocytic expression of HLA-DR independently anticipates the clinical course of undifferentiated patients with SARS-Cov-2 in Emergency Department

#### LAFON Thomas^1^, GUÉRIN Estelle^1^, CHAPUIS Nicolas^2^, DAIX Thomas^1^, FONTENAY Michaela^2^, VIGNON Philippe^1^, FRANÇOIS Bruno^1^, FEUILLARD Jean^1^

##### ^1^CHU Dupuytren, Limoges, France; ^2^Hôpital Cochin, AP-HP, Paris, France

###### Correspondence: Thomas LAFON (thomas.lafon@chu-limoges.fr)

*Annals of Intensive Care* 2022, **12(1):**CO-59

**Rationale:** Assessing the prognosis of patients with SARS-Cov-2 related pneumonia is fundamental in Emergency Department (ED) to manage the flow of patients. Previous cohort of patients highlighted that 1/3 of patients worsened after their admission. Standard scores and biomarkers used in ED are strongly associated with immediate severity but their prognostic performance to predict clinical course of patients is limited. COVID-19 leads to immunological impairment with lymphopenia, low expression of HLA-DR on monocytes and high neutrophil count. We evaluated the prognostic performance of immunological parameters (circulating lymphocyte subsets, neutrophils and immature granulocytes, MO1 immature to M3 inflammatory stage based on CD16 and CD14 expression, and mHLA-DR) to anticipate the clinical evolution of undifferentiated COVID-19 patients in ED.

**Patients and methods/Materials and methods:** We conducted a prospective multicenter study during 5 months in two ED: one for exploratory cohort and one for consolidation. In addition to clinical, biological and radiological parameters, circulating lymphocyte subsets, mature and immature granulocytes and mHLA-DR were analyzed using routine flow cytometry on the first blood sample. The primary endpoint was the non-deterioration determined by 3 clinicians who were blinded from cytometry results and defined from a composite criterion: (i) need for high flow oxygen, (ii) ICU admission, (iii) in-hospital mortality related to SARS-COV-2, (iiii) readmission within 5 days. Sample size of 245 patients (estimated deterioration = 20%).

**Results:** 284 patients were analyzed (men = 56%; mean age = 63 ± 17 yrs; onset of symptoms 7 ± 4 days, SpO_2_ = 95 ± 4%, respiratory rate = 23 ± 7 breaths/min) including 72 patients (25%) who were assigned to the deterioration group (high flow oxygen 86%, ICU admission 35%, in-hospital mortality 40%, readmission 8%). On the first series of 180 patients, among the 27 clinical and biological parameters tested, the 3 independent variables were P/F ratio (adjusted OR = 2.8, LCI = 1.2, UCI = 6.4, p = 0.014), lymphocyte count (adjusted OR = 4.2, LCI = 1.7, UCI = 10.2, p = 0.0019) and mHLA-DR (adjusted OR = 4.5, LCI = 2, UCI = 10.3, p = 0.0003). On the consolidating series of 104 patients, the independent variables were also P/F ratio (adjusted OR = 9.2, LCI = 3, UCI = 19, p = 0.0001), lymphocyte count (adjusted OR = 4, LCI = 1, UCI = 16, p = 0.049) and mHLA-DR (adjusted OR = 4.1, LCI = 1.2, UCI = 13.7, p = 0.02). A score that included P/F ratio > 300, lymphocyte count > 1.1 G/L and HLA DR intensity > 15 000 reached 100% of PPV for non-deterioration.

**Conclusion:** With the P/F ratio and the lymphocyte count, absence of DR loss on monocytes was strongly independently and robustly associated with clinical non-deterioration. A simple predictive score ratio seems interesting to allow safe rule-out, over-triage reduction and better allocation of hospital resources.

**Compliance with ethics regulations:** Yes in clinical research.

### CO-60 Incidence and outcome of invasive pulmonary aspergillosis in critically-ill COVID-19 patients: a French multicenter experience

#### DESMEDT Luc^1^, RAYMOND Matthieu^1^, ASFAR Pierre^2^, DARREAU Cedric^14^, REIZINE Florian^3^, COLIN Gwenhaël^4^, AUCHABIE Johann^13^, LACOMBE Béatrice^12^, KERGOAT Pierre^11^, HOURMANT Baptiste^6^, DELBOVE Agathe^7^, FRÉROU Aurélien^8^, MORIN Jean^15^, ERGRETEAU Pierre Yves^9^, SEGUIN Philippe^10^, REIGNIER Jean^1^, LASCARROU Jean-Baptiste^1^, CANET Emmanuel^1^

##### ^1^Service de Médecine Intensive Réanimation, CHU de Nantes, Nantes, France; ^2^Service de Médecine Intensive Réanimation, CHU d'Angers, Angers, France; ^3^Service de Médecine Intensive Réanimation, CHU de Rennes, Rennes, France; ^4^Service de Réanimation Polyvalente, CHD La Roche Sur Yon, La Roche Sur Yon, France; ^5^Service de Réanimation Polyvalente, CH Saint Nazaire, Saint Nazaire, France; ^6^Service de Réanimation Polyvalente, CHU de Brest, Brest, France; ^7^Service de Réanimation Polyvalente, CH de Vannes, Vannes, France; ^8^Service de Réanimation Polyvalente, CH de Saint Malo, Saint Malo, France; ^9^Service de Réanimation Polyvalente, CH de Morlaix, Morlaix, France; ^10^Service de Réanimation chirurgicale, CHU de Rennes, Rennes, France; ^11^Service de Réanimation Polyvalente, CH de Cornouille, Quimper, France; ^12^Service de Réanimation Polyvalente, CH Bretagne Sud, Lorient, France; ^13^Service de Réanimation Polyvalente, CH Cholet, Cholet, France; ^14^Service de Réanimation Polyvalente, CH du Mans, Le Mans, France; ^15^Unité de soins intensifs de Pneumologie, Nantes, France

###### Correspondence: Luc DESMEDT (Luc.desmedt@live.fr)

*Annals of Intensive Care* 2022, **12(1):**CO-60

**Rationale:** Recent studies identified coronavirus disease 2019 (COVID-19) as a risk factor for invasive pulmonary aspergillosis (IPA), with conflicting data on prevalence and impact on patients’ outcomes. We aimed to determine the incidence and outcome of COVID-19 associated pulmonary aspergillosis (CAPA) in mechanically ventilated COVID-19 patients.

**Patients and methods/Materials and methods:** We conducted a multicenter observational study. All Covid-19 patients admitted to 15 ICUs from Pays-de-la-Loire and Bretagne regions between February 1st 2020 and December 31th 2020 and treated with mechanical ventilation were included. The main objective was to assess the incidence and mortality of CAPA in COVID-19 patients with acute respiratory distress syndrome (ARDS). Each case of CAPA reported by local investigators was reviewed by an adjudication committee of 3 independent experts. CAPA were diagnosed and graded according to the 2020 ECMM/ISHAM consensus criteria [1].

**Results:** Among the 15 participating centers, 2 (13%) had a screening strategy for Aspergillus in mechanically ventilated patients, while in the other 13 (87%) centers CAPA was investigated in case of respiratory deterioration. During the study period, 644 mechanically ventilated patients were included (mean age 65 (SD 11) years-old; 523 (74%) men). Mean duration of mechanical ventilation was 21 (19) days and 90-day mortality rate was 34%. According to the EORTC criteria, 114 (16%) patients had underlying immunosuppression. Overall, 348 (49%) patients were treated with corticosteroids and no patients received Tocilizumab. Overall, 35 (5%) patients had proven/probable/possible CAPA. Among them, 18 (3%) patients fulfilled the criteria for probable CAPA and 17 (3%) patients met the criteria for possible CAPA (3%). No case of histologically proven CAPA was reported. Patients with probable CAPA were 70 (7) years-old and 4 (22%) were immunocompromised. Patients with possible CAPA were 68.64 (8.62) years-old and 3 (17.6%) were immunocompromised. Probable CAPA were diagnosed after 8 [4.25–21.75] days of mechanical ventilation and possible CAPA after 6 [1–10] days. At day-90, 16 (46%) of the 35 patients with CAPA were dead. Among the 18 patients with probable CAPA, the mortality at day-90 was 55% (n = 10) and the duration of mechanical ventilation was 27 [18–33.5] days. Patients with possible CAPA had a mortality rate at day-90 of 35% (n = 6) and a duration of mechanical ventilation of 16 [13–23] days.

**Conclusion:** In our study, the incidence of IPA in ARDS-Covid-19 patients treated with mechanical ventilation was low, and only probable CAPA was associated with a higher mortality at day-90.

**Reference 1:** Koehler, Philipp, Matteo Bassetti, Arunaloke Chakrabarti, Sharon C A Chen, Arnaldo Lopes Colombo, Martin Hoenigl, Nikolay Klimko, et al. « Defining and managing COVID-19-associated pulmonary aspergillosis: the 2020 ECMM/ISHAM consensus criteria for research and clinical guidance. Lancet Infect Dis 2021; 21: e149–e162.

**Compliance with ethics regulations:** Yes in clinical research.

### CO-61 Psychotraumatic impact of the child's visit to the adult intensive care unit: first results of the ENVIFAR study

#### NGUYEN Stéphanie^1^, VANGI Marie-Aude^2^, FOURNIER Alicia^1^, SOUPPART Virginie^4^, PILI FLOURY Sébastien^5^, QUENOT Jean Pierre^6^, DUBOST Jean-Louis^7^, BOUHEMAD Belaid^8^, CAPELLIER Gilles^9^, LAURENT Alexandra^2,10^

##### ^1^Université Bourgogne Franche-Comté, Dijon, France; ^2^ CHU Dijon, Réanimation Chirurgicale, Dijon, France; ^3^ Université Bourgogne Franche-Comté, Laboratoire Psy-DREPI, Dijon, France; ^4^APHP Saint Louis, Médecine Intensive Réanimation, Paris, France; ^5^CHU Besançon, Réanimation Chirurgicale, Besançon, France; ^6^CHU Dijon, Médecine Intensive Réanimation, Dijon, France; ^7^CH Pontoise, Réanimation Médico-Chirurgicale,, Pontoise, France; ^8^CHU Dijon, Réanimation Chirurgicale, Dijon, France; ^9^CHU Besançon, Réanimation Médicale, Besançon, France; ^10^Université Bourgogne Franche-Comté, Laboratoire Psy-DREPI, Dijon, France

###### Correspondence: Stéphanie NGUYEN (stephanielaurent@gmx.fr)

*Annals of Intensive Care* 2022, **12(1):**CO-61

**Rationale:** While Intensive Care Units (ICU) have widely opened their doors to relatives, the presence of visiting children and adolescents in ICU remains a sensitive issue. In a desire to protect the child from a potentially traumatic environment, some units refuse or restrict visits to children (Laurent et al., 2019). The aim of this study is to measure the psychotraumatic impact of a child’s visit to adult ICU and to identify the influence of the accompanying parent on the child’s visit.

**Patients and methods/Materials and methods:** This study was conducted in six ICU with 22 children, 15 accompanying parents. The psychotraumatic impact of the visit on the child was measured at 7 and 30 days of the visit using the CRIES-8 (Children’s revised impact of event scale, CRIES-8). We also measured the anxiety-depression of the accompanying parent during the visit using the HADS (REF). We complemented the quantitative approach with semi-structured interviews to capture the experience of the visit at 7 days.

**Results:** Of the 22 children included, 15 children were able to complete questionnaires at 7 days of the visit and 21 at 30 days. At 7 days of the visit, 9 out of 15 children showed acute stress. One month after, 9 out of 21 children suffered from a potential post-traumatic stress disorder. The thematic analysis shows that the visit is indeed disturbing for most of children. However, children expressed that this visit was reassuring to them. Certain dimensions are identified as a source of distress and can explain the CRIES-8 scores: the absence of the hospitalized parent, the distress of the accompanying parent, and the disruption of daily life. In the same sense, our study shows a positive trend correlation between the HADS scores of the accompanying parents and the CRIES-8 score of the children (r2 = .24, p = .062) at 7 days. Thus, the higher the anxiety-depressive symptomatology of the parents, the higher the acute stress symptomatology of the children tended to be.

**Conclusion:** More than the visit itself, these initial results show the importance of considering the visiting parent's experience during the child’s visit.

**Reference 1:** Laurent A., Leclerc P., Nguyen S., Capellier G. (2019) The effect visiting relatives in the adult ICU has on children. Intensive Care Medicine. https://doi.org/10.1007/s00134-019-05690-2.

**Reference 2:** Laurent A., Nguyen S., Leclerc P., Capellier G. (2020) L’enfant visiteur en réanimation adulte: vécu psychologique de la visite et dispositifs d’accompagnement. Pratiques psychologiques (2020).

**Compliance with ethics regulations:** Yes in clinical research.

### CO-62 Measuring ethical decision-making climate before and after the implementation of therapeutic perspective meetings—CLIMETHIC study

#### VALBRUN Jean-David^1^, NGUYEN Yên-Lan^2^

##### ^1^Gustave Roussy, Villejuif, France; ^2^CHU Cochin, Paris, France

###### Correspondence: Jean-David VALBRUN (jean-david.valbrun@orange.fr)

*Annals of Intensive Care* 2022, **12(1):**CO-62

**Rationale:** The perception of inappropriate care in end-of-life situations can be a source of conflict and burnout among caregivers. The purpose of this study is to describe caregivers' perceptions of end-of-life care in the surgical intensive care unit and intermediate care unit of our hospital before and after the implementation of therapeutic perspective meetings (TPMs).

**Patients and methods/Materials and methods:** Caregivers in the ICU were invited to participate in this survey before (August to October 2018) and after (August to October 2019) the implementation of TPMs. The “ethical decision-making climate” self-questionnaire was used. It explores 3 dimensions: working conditions, managerial skills and end-of-life decision making. Descriptive and analytical analyses were performed. This study was approved by the SFAR ethics commission (opinion n° IRB 00010254-2017-133).

**Results:** The implementation of TPMs improved caregivers’ perception of the existence of regular meetings to discuss projects (2 vs 3, p < 0.0001) and the quality of care delivered (2 vs 3, p < 0.0026). TPMs improved caregivers' perception of expressing disagreement with opinions or values (3 vs. 3, p = 0.02), nurses' presence during end-of-life interviews (3 vs. 3.5; p = 0.003) and nurses' involvement in these decisions (2 vs. 3, p < 0.0001). The TPMs did not change caregivers’ perception on the medical team management, on the realization of formalized debriefings after a difficult care or end-of-life situation, on the temporality of end-of-life decisions or on the admissions of patients with minimal chances of recovery.

**Conclusion:** The implementation of TPMs has improved the perception of caregivers regarding communication between caregivers on patient management, particularly for end-of-life situations, and has encouraged the involvement of nurses in these decisions as well as their presence during interviews with the families. There was no impact on perceptions of the temporality of end-of-life decision-making or on admissions of patients with minimal chance of recovery. Further studies are needed to assess the impact of TPMs on the prevalence of conflict, risk of burnout, and quality of care delivered.

**Reference 1:** Piers et coll., JAMA, 2011.

**Reference 2:** Bo Van den Bulcke et coll. BMJ, 2018.

**Compliance with ethics regulations:** Yes in clinical research.
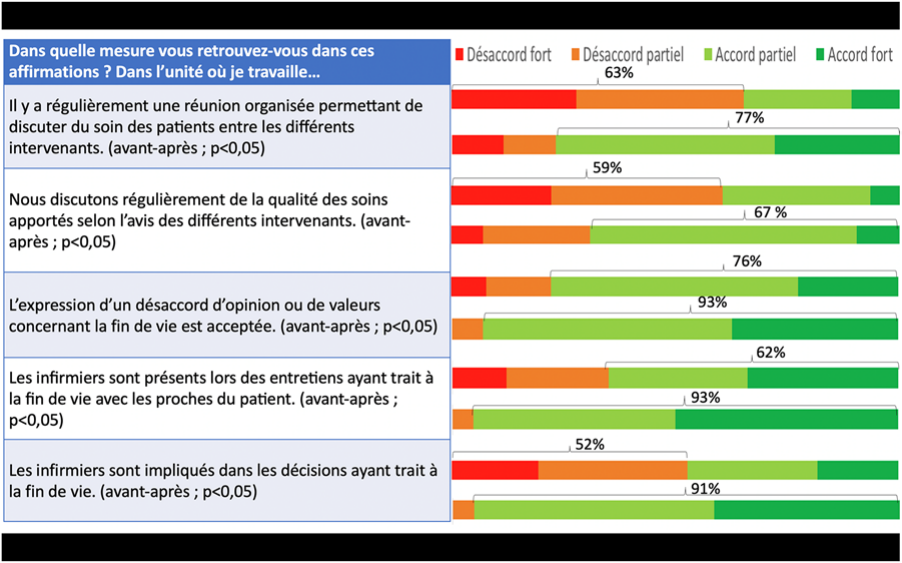


*Comparaison avant-après*

### CO-63 Intensivists’ differential ethical perceptions about withholding and withdrawing of treatment (Wh&Wd) decisions: a clinical ethics empirical investigation

#### SPRANZI Marta^1^

##### ^1^AP-HP, Paris, France

###### Correspondence: Marta SPRANZI (marta.spranzi-ext@aphp.fr)

*Annals of Intensive Care* 2022, **12(1):**CO-63

**Rationale:** Intensive care professional societies have issued policy recommendations regarding Wh&Wd decisions that consistently classify them as ethically equivalent and ethically neutral insofar as they correspond to “passive” actions. However, on the ground some are perceived as more “active” than others most notably Wd as opposed to Wh decisions. Recent studies also show that there is a personal “variability” in the way such decisions are made, and that further investigation is needed. Therefore it seemed necessary to explore intensivists’ differential ethical perceptions about Wh&Wd decisions and the ethical reasons underlying their spontaneous judgments.

**Patients and methods/Materials and methods:** A qualitative retrospective and multicentered study was initiated in 7 AP-HP intensive care units. In-depth interviews were conducted with 39 intensivists of different age groups and levels of professional experience, and with 5 department heads. We used an inductive (“grounded theory” inspired), multidisciplinary clinical ethics approach, aimed at eliciting and retrieving practitioners’ ethical intuitions about different Wh&Wd decisions. Interview transcripts were analyzed using a “thematic analysis” approach.

**Results:** Practitioners’ perceptions of Wh&Wd decisions vary along two continuous dimensions: “active”/passive and ethically problematic/unproblematic. Three groups have been identified and described: those who tend to consider Wh&Wd decisions (1) as passive and unproblematic, (2) as active and problematic, and (3) as active and unproblematic. More interestingly, these groups are differently correlated with three other ethically relevant variables: the role of consensus during the collegial procedure, the difference between withholding and withdrawing decisions, and the definition of one’s own overarching goal as professional.

**Discussion:** The study results confirm what is now called practitioners’ “variability” and the moral complexity of Wh&Wd decisions. Moreover, it shows that, contrary to the “moral neutrality” doctrine, practitioners consider different Wh&Wd decisions as more or less morally problematic along a continuum from passive to “very active”, although Wd&Wh practices are mostly described as different from euthanasia. It also shows that practitioners’ “variability” does not simply correspond to sociological variables which could be easily erased, but has deep-seated ethical roots.

**Conclusion:** Practitioners’ perception and ethical reasons about Wh&Wd decisions have never been analyzed in depth. Our results allow for the recognition of intensivists’ moral distress about Wh&Wd decisions, and open up new perspectives to deal with their deep-rooted variability, most notably by working on the rationale and format of the collegial procedure.

**Reference 1:** Nadig, N. R., & Ford, D. W. (2019). A Consensus: Everyone Agrees Collectively but No One Believes Individually. Critical care medicine, 47(10), 1470–1472.

**Reference 2:** Wilkinson, D. J., & Truog, R. D. (2013). The luck of the draw: physician-related variability in end-of-life decision-making in intensive care. Intensive care medicine, 39(6), 1128–1132.

**Compliance with ethics regulations:** N/A.
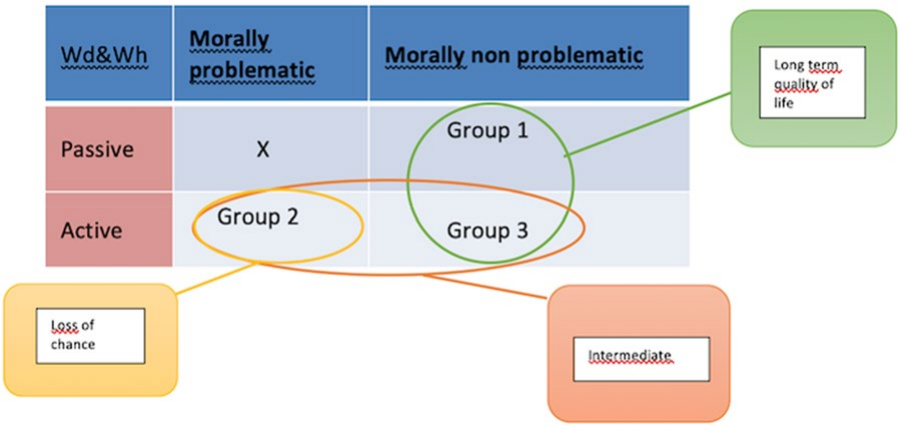


*Ethical perceptions of WD&Wh decisions and intensivists'values*

### CO-64 Association of nursing team composition with mortality in critically ill patients: a multicenter observational studY

#### GUÉRIN Claude^1,5^, PAYET Cécile^5^, BABOI Loredana^3^, ALLAOUCHICHE Bernard^2^, ARGAUD Laurent^1^, AUBRUN Fréderic^3^, BOHÉ Julien^2^, DAILLER Frédéric^4^, FELLAHI Jean-Luc^4^, LEHOT Jean-Jacques^5^, PIRIOU Vincent^2^, RIMMELÉ Thomas^1^, POLAZZI Stéphanie^5^, DUCLOS Antoine^5^

##### ^1^Hôpital Edouard Herriot Hospices Civils de Lyon, Lyon, France, France; ^2^Groupement hospitalier Lyon Sud, Lyon, France; ^3^Groupement hospitalier nord, Lyon, France; ^4^Groupement hospitalier Est, Lyon, France; ^5^Université de Lyon, Lyon, France

###### Correspondence: Claude GUÉRIN (claude.guerin@chu-lyon.fr)

*Annals of Intensive Care* 2022, **12(1):**CO-64

**Rationale:** In the Intensive Care Unit (ICU), patient-to-nurse ratio is associated with patient outcomes but little is known about the habit among staff members of working together. The goal of present study is to investigate the role of nursing team composition on patient ICU mortality.

**Patients and methods/Materials and methods:** Retrospective multicentre observational study in eight adult ICUs from the Greater Lyon area in France, analysing all patients present between January 1st 2011 and December 31st 2016. The team composition was evaluated using the familiarity among the ICU caregivers, which was measured by shift (from 7:00 am to 6:59 pm and from 7:00 pm to 6:59 am) as the mean number of previous collaborations between each nursing team member during previous shifts within the given ICU. Suboptimal collaboration was defined as less than 50. The patient-to-nurse ratio and patient-to-auxiliary nurse ratio were also considered (suboptimal ratio defined as higher than 0.5 and 0.25, respectively), as well as individual length of experience in ICU for every caregiver (suboptimal defined as less than 400 previous shifts with collaboration). The primary outcome was inpatient death at the time of ICU discharge, excluding patients for whom a decision to forego life-sustaining therapy was made. Inpatient death during the shift at admission was secondarily considered. A multiple Poisson regression was computed to identify the determinants of ICU mortality per shift, taking into account the ICU site, patients’ characteristics, and caregivers’ workload.

**Results:** A total of 43,479 patients were admitted to the ICUs of whom 3,311 (7.6%) died, corresponding to 8.8% shifts (3,101/35,072) with at least one death. The adjusted model showed an increased risk of patient mortality during shifts exposed to suboptimal team familiarity lower than 50 previous collaborations (Relative Risk 1.06, 95% Confidence Intervals [1.04–1.09]) and simultaneously with suboptimal patient-to-staffing ratio (RR 1.13, IC95% [1.07–1.19]). Suboptimal team composition reflected a total of 172 [116–241] shifts with potentially avoidable death, corresponding to 7.5% of all ICU shifts with occurrence of inpatient death. The risk of death at admission was also higher in case of suboptimal team composition (RR 1.27, IC95% [1.13–1.43]).

**Conclusion:** In conclusion, the familiarity between ICU nursing staff is significantly associated with inpatient death additionally to patient-to-caregiver ratio. Improving team composition should be a management goal in ICU.

**Compliance with ethics regulations:** Yes in clinical research.
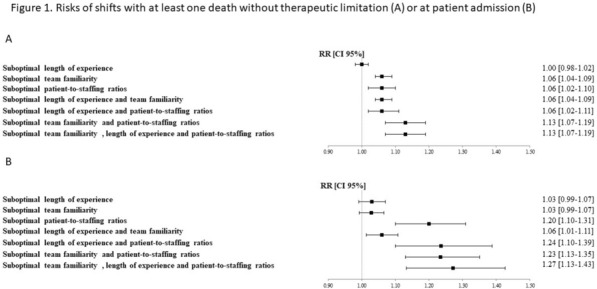


*Figure 1*

### CO-65 Healthcare workers cost awareness in intensive care unit: a prospective multicentric study

#### GABARRE Paul^1^, MAYLIN Rima^1^, STUDER Antoine^2^, GUITTON Christophe^3^, ANTIGNAC Marie^1^, DE PROST Nicolas^4^, URBINA Tomas^1^, BONNY Vincent^1^, MISSRI Louai^1^, EHRMINGER Sebastien^1^, BAUDEL Jean-Luc^1^, JOFFRE Jérémie^1^, AIT OUFELLA Hafid^1^, MAURY Eric^1^

##### ^1^Hôpital Saint Antoine, Paris, France; ^2^CHRU Strasbourg, Strasbourg, France; ^3^Centre Hospitalier du Mans, Le Mans, France; ^4^Hôpital Henri Mondor, Créteil, France

###### Correspondence: Paul GABARRE (paulgabarre@hotmail.com)

*Annals of Intensive Care* 2022, **12(1):**CO-65

**Rationale:** Physicians play an important role in controlling health care spending, by prescribing more or less expensive treatments. The aim of this study was to evaluate the knowledge of health care workers of the cost of the treatments they daily use in ICU.

**Patients and methods/Materials and methods:** We conducted a prospective, multicenter, volunteer-based study. A survey was delivered and completed anonymously by healthcare workers (HCWs) of four French ICUs, three university affiliated and one non-university affiliated. The survey proposed to estimate the price of 37 treatments frequently used in critical care. Cost estimations were expressed as percentage of the real cost; an estimation was considered correct if it was ± 50% of the true prices

**Results:** 158 HCWs answered to the survey, including 75 nurses and 82 physicians. Medical staff was composed of 36 seniors and 45 juniors. Median age of the respondents was 29 [25–36] years. They provided 5798 answers. Only 18% of estimations were within 50% of the real cost. In univariate analysis, Medical staff was more likely to respond correctly than nurses, with 20% vs. 16,8% of correct cost estimations (p = 0,001). Unsurprisingly, among the medical staff, seniors were better at evaluating the prices than juniors, with 22,58% of good answers vs. 18,3% p = 0,0051. There was no difference between university affiliated and non-university affiliated hospital’s HCWs. Antibiotics were the most often well estimated treatments, with 25% of correct estimations, followed by fluids (24%), vasopressors (19%), sedative drugs (14%), chemotherapy and immunosuppressive treatments (13%). Interestingly, the most expensive treatments prices were under evaluated whereas the cheapest ones were overvalued. For example, median estimation of the cost of eculizumab was only 9 [2.5–29]% of its real price.

**Conclusion:** ICU health care workers have a poor knowledge of the price of treatments frequently used in the ICU.

**Compliance with ethics regulations:** N/A.

### CO-66 How subjective determinants influence antibiotic prescribing behaviors among intensivists in French ICUs? A nation-wide cross-sectional survey

#### GIANNOLI Alice^1^, BAUDOT Amandine^1^, THIERY Guillaume^1^

##### ^1^CHU Nord Saint-Etienne, Saint Etienne, France

###### Correspondence: Alice GIANNOLI (alice.giannoli@gmail.com)

*Annals of Intensive Care* 2022, **12(1):**CO-66

**Rationale:** In order to improve antibiotic’s use, strategies, such as stewardship programs, have been developed. However, their efficacy remains flawed and antibiotics remain overused. One of the reasons lies in subjective determinants of the prescription. The aim of our study is to explore personal, social, cultural and contextual factors that can influence antibiotic prescribing behaviour in the ICU.

**Patients and methods/Materials and methods:** For this nation-wide study, we designed a questionnaire to explore 4 domains that may influence antibiotic prescription: demographic characteristics, knowledge and medical practice, social interactions, and psychological factors. The questionnaire was distributed to all ICUs in France through a personal email sent to the head of each ICU, asking them to send the questionnaire to doctors and residents of their team. Two reminders were sent, at 2 and 4 months.

**Results:** We received 803 responses (55% senior physicians, 15% fellows, 5% academic physicians and 25% residents). Primaries specialties was anesthesiology (47%), medical specialty (38%), emergency medicine (8%) and intensive care (DESMIR, 7%). Fifty-two percent worked in university hospitals, 41% had a combined activity. The population is comparable to the global demography in French ICUs (1). A large majority (84%) of the respondents felt concerned by antibiotic resistance, but 45% didn’t believe that a restrictive use could decrease it. Only 54% of the responders shared the statement “we must always seek the narrowest spectrum”. The decision to not initiate an antibiotic treatment was perceived as more difficult that initiating, adapting of interrupting it (p < 0.0001). For 85% of the responders, antibiotic prescription is a team decision. However, 30% declare that they don’t ask any advice and 22% report a large variability within their team. Twenty-two percent of the responders don’t hesitate to modify a prescription of their college and 13% of their boss if considered inappropriate. Seventy-eight percent of the responders consider themselves as “restrictive prescribers”. More academic physicians, senior physicians and physician having validated the subspecialisation in intensive care (DESC) declared themselves as “restrictive prescribers”, whereas more residents declared themselves “liberal prescribers”. Analysis of these “liberal prescribers” reveals that they are significantly more inexperienced, anxious and insecure, with a limited ability to take a step back.

**Conclusion:** This work confirms that subjective determinants hinder reasoned antibiotic prescribing. Factors such as prescriber anxiety and lack of self-confidence, linked in particular to a lack of knowledge, stand out. The fear of missing a septic etiology pushes the physician to prevail the short-term benefit taking over the long-term consequences.

**Compliance with ethics regulations:** Yes in clinical research.

### CO-67 Assessment of new methods for bedside measurement of airway opening pressure without the need for low-flow insufflation

#### HAUDEBOURG Anne-Fleur^1,3^, DELAMAIRE Flora^2^, LOUIS Bruno^3^, MEKONTSO DESSAP Armand^1,3^, CARTEAUX Guillaume^1,3^

##### ^1^C.H.U. HENRI MONDOR, Creteil, France; ^2^HÔPITAL PONTCHAILLOU, Rennes, France; ^3^INSTITUT MONDOR DE RECHERCHE BIOMEDICALE (IMRB), Creteil, France

###### Correspondence: Anne-Fleur HAUDEBOURG (annefleur.maignant@aphp.fr)

*Annals of Intensive Care* 2022, **12(1):**CO-67

**Rationale:** The gold standard method proposed to detect and measure airway opening pressure (AOP) in patients with acute respiratory distress syndrome (ARDS) requires a low-flow insufflation (i.e., 5 L/min). This ensures that the resistive pressure is negligible but might potentially be poorly tolerated. Theoretically, during usual constant flow insufflation (i.e., 30 to 60 L/min), the delta pressure comprised between the PEEP and the first inflection point of the airway pressure waveforme, subsequently called “conductive pressure”, comprises resistive pressure and potential AOP (Fig. 1). We assessed the accuracy and tolerance of calculating AOP as the conductive pressure minus resistive pressure during usual constant flow insufflation. We also assessed an automated computer-based detection of AOP.

**Patients and methods/Materials and methods:** AOP was measured in ARDS patients using three different methods: 1: gold standard: low-flow insufflation (5 L/min); 2: visual waveform analysis during standard ventilation with a constant flow rate of 30 and 60 L/min: AOP was identified as the conductive pressure minus the resistive pressure; 3: automated computer-based detection of the rupture of the airway pressure slope. We compared methods 2 and 3 to the gold standard and collected the lowest SpO_2_ during each measurement to assess the tolerance.

**Results:** 25 ARDS patients were included so far, of whom 13 (52%) had an AOP > 5 cmH_2_O with a median value of 7 cmH_2_O [6–8]. AOP obtained by visual waveform analysis during ventilation with usual flow rate showed a very strong correlation with reference value (r = 0.8 at flow 30 L/min and r = 0.93 at flow 60 L/min, p < 0.001, Fig. 1). Bland–Altman plot for visual waveform analysis at 60 L/min showed a bias of 0.08 with agreement limits between − 1.87 and 2.03 cmH_2_O. AOP obtained by computer-based detection showed a strong correlation with reference value (r = 0.66 at 30 L/min and r = 0.70 L/min, p < 0.001) but with a trend toward overestimation of AOP. Visual or computer-based waveform analysis during ventilation with usual flow rate was better tolerated than gold standard method (decrease in SpO_2_: − 0.5% [− 2–0] and 0% [− 2–0] at 30 and 60 L/min respectively versus − 2% [− 4.8 to − 1] at 5 L/min, p < 0.001).

**Conclusion:** These preliminary data suggest that measuring AOP is feasible at standard insufflation flow rate with a simple, quick and safe method based on visual inspection of airway pressure waveform.

**Compliance with ethics regulations:** Yes in clinical research.
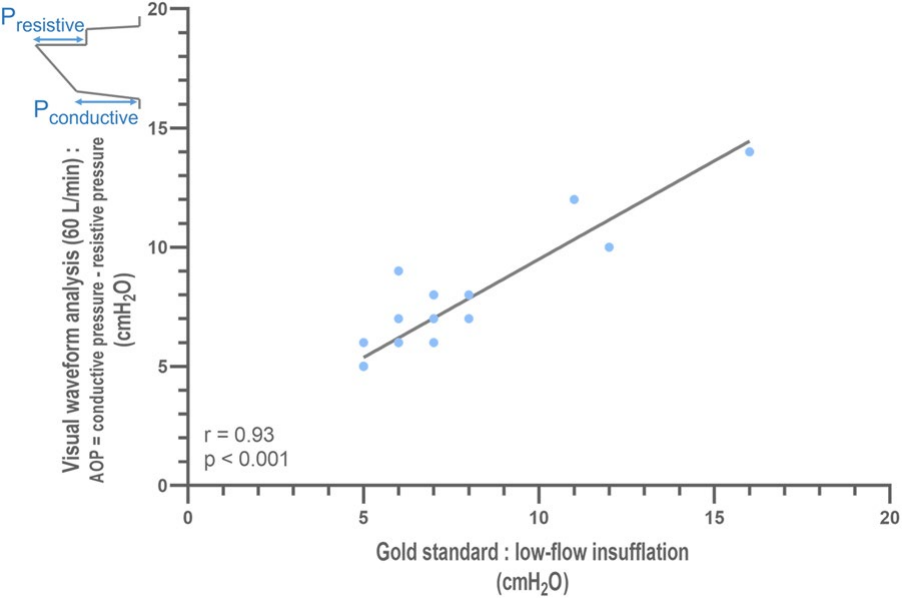


*Figure 1: Spearman correlation between AOP obtained by visual waveform analysis during standard ventilation at 60 L/min and gold standard at low-flow insufflation (full line: linear regression line).*

### CO-68 Detection and respiratory mechanic assessment of asymmetrical acute respiratory distress syndrome with electrical impedance tomography

#### ROZE Hadrien^1^, BONNARDEL Eline^1^, BOISSELIER Clément^1^, GRESS Gauthier^1^, REPUSSEAU Benjamin^1^, PERRIER Virginie^1^, CHRISTELLE Pellerin^1^, OUATTARA Alexandre^1^

##### ^1^CHU de Bordeaux, Bordeaux, France

###### Correspondence: Hadrien ROZE (hadrien.roze@chu-bordeaux.fr)

*Annals of Intensive Care* 2022, **12(1):**CO-68

**Rationale:** ARDS is a heterogeneous syndrome involving different phenotypes with distinct clinical and outcome characteristics. Low flow pressure volume (PV) curve reflects the global behavior of the lungs and can be misleading in ARDS. With Electrical Impedance Tomography (EIT) it is possible to measure the distribution of ventilation in each lung in order to describe asymmetrical lung injury. Moreover, each lung respiratory mechanic and PV curve can be assessed with an EIT derived method. In the present study, we hypothesized that some patients may have asymmetrical ARDS where EIT would provide different information from those obtained from global P-V curves.

**Patients and methods/Materials and methods:** Prospective study (NCT04386720). We recorded the low flow PV curve without PEEP of the ventilator in order to assess airway closure and the recruited volume.^1^ AT the same time with EIT we analyzed the derived PV curves of each lung.^2^ A difference of tidal ventilation of at least 20% between the 2 lungs was considered as asymmetrical ARDS. We compared respiratory mechanic between the 2 lungs in patients with asymmetrical ARDS.

**Results:** We analyzed 26 patients, 18 patients had asymmetrical lung injury, the most injured lung received 28.5 ± 8.0% of the tidal ventilation, its compliance was significantly lower than the less injured lung: 11.9 ± 7.8 vs 28.8 ± 14.3 ml cmH_2_O-1p < 0.0001 respectively. Thirteen patients had airway closure with an airway opening pressure > 4 cmH_2_O; Global PV curve AOP was 6.6 ± 3.4 cmH_2_O, similar to the less injured lung with 6.6 ± 3.3 cmH_2_O, whereas the most injured lung had a significantly higher AOP of 10 ± 3.9, p = 0.003. The PEEP recruited volume V_REC_ was significantly lower in the most injured lung: 67 [43–123] vs 120 [57–225] ml, p = 0.015. The compliance of the recruited lung (Crec) in the more injured lung was not different from the less injured 22.0 [5.6–32.3] vs 24.0 [11.8–41.0] ml.cmH_2_O−1 respectively, p = 0.460.

**Conclusion:** EIT can show asymmetrical ARDS and assess respiratory mechanic of each lung. Personalizing ventilator management in asymmetrical lung injury entails assessing each lung-specific risk of VILI with repeated opening and collapse of the most injured lung and overdistension in the less injured lung. A specific compromise between PEEP and VT in asymmetrical ARDS could attenuate these opposite risk of VILI.

**Reference 1:** AJRCCM 2018 197:132–136.

**Reference 2:** AJRCCM 2021;203:511–515.

**Compliance with ethics regulations:** Yes in clinical research.
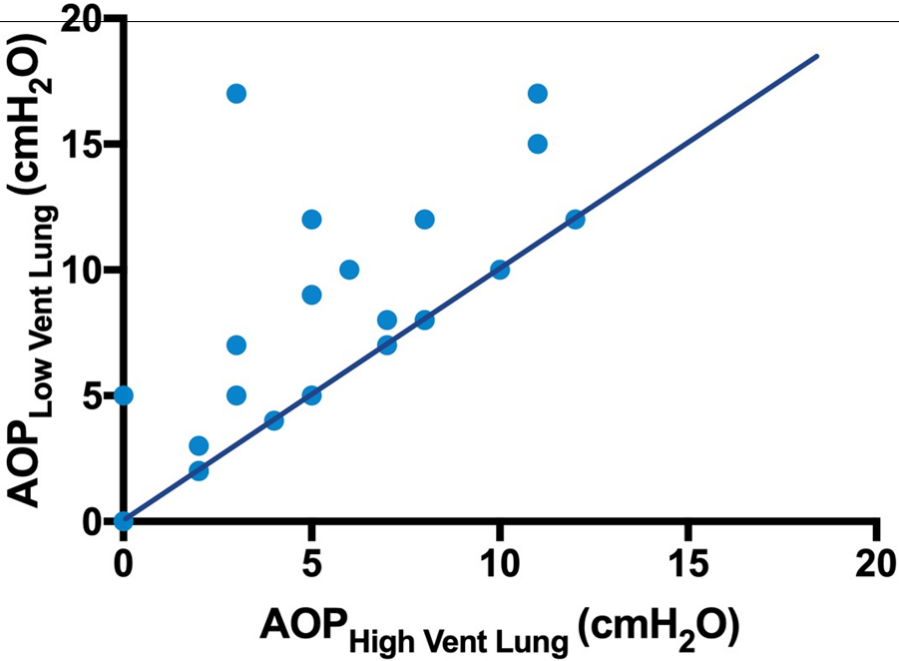


*Airway Opening Pressure measured with EIT in each lung*

### CO-69 Response to PEEP assessed on computed tomography in COVID-19 ECMO patients

#### RICHARD Jean-Christophe^1,2^, YONIS Hodane^1^, SIGAUD Florian^3^, MEZIDI Mehdi^1^, DAVILA SERRANO Eduardo^2^, ORKISZ Maciej^2^, ROUX Emmanuel^2^, GAILLET Maxime^1^, BAYAT Sam^3^, TERZI Nicolas^3^, BITKER Laurent^1,2^

##### ^1^Hopital de la Croix-Rousse, Lyon, France; ^2^Université de Lyon, Université Claude Bernard Lyon 1, INSA-Lyon, UJM-Saint Etienne, CNRS, Inserm, CREATIS UMR 5220, U1206, Lyon, France; ^3^CHU Grenoble Alpes, Grenoble, France

###### Correspondence: Jean-Christophe RICHARD (j-christophe.richard@chu-lyon.fr)

*Annals of Intensive Care* 2022, **12(1):**CO-69

**Rationale:** Lung potential for recruitment of COVID-19 ARDS is a matter of debate, and may be patient-dependent, favoring individualization of PEEP setting. PEEP selection in severe COVID-19 patients under ECMO may be more challenging as no study has assessed recruitment potential in this setting. The aim of the study was to compare potential for recruitment and the impact of PEEP on lung aeration with computed tomography (CT) in moderate, severe without ECMO and severe under ECMO ARDS patients.

**Patients and methods/Materials and methods:** We conducted a two-center prospective observational study in adult COVID-19 related ARDS patients who had an indication for CT. Main exclusion criteria were ARDS onset > 72 h in non-ECMO patients or ECMO onset > 72 h. Four low-dose CT acquisitions were performed at both end-expiration and end-inspiration at PEEP selected by attending physician, and at end-expiration at both PEEP 5 and 15 cmH_2_O.

**Results:** 99 patients (76% male, age 62 [54–71] year) were included, of whom 24 had severe ARDS under ECMO, 59 severe ARDS without ECMO and 16 moderate ARDS. ECMO patients were ventilated with significantly lower tidal volume (1.0 [1.0–1.0] vs 6.0 [5.9–6.0] ml/kg predicted body weight (PBW) and higher PEEP (15 [13–15] vs. 10 [5–10] cmH_2_O. The median amount of recruitable lung between PEEP 5 and 15 cmH_2_O was 6.2 [4.0–9.9]% of lung weight, and tidal hyperinflation amounted to 0.3 [0.1–1.0] ml/kg PBW. Tidal hyperinflation > 1 ml/kg PBW was observed in 19 (25%) non-ECMO patients and 0 (0%) ECMO patients. Non-inflated lung at PEEP 5 was significantly greater in ECMO than in non-ECMO patients (Fig. 1). End-expiratory aerated lung volume (EELV) at PEEP 5 was significantly lower in ECMO patients. Recruitment induced by PEEP increase from 5 to 15 cmH_2_O was not significantly different between groups, while PEEP-induced hyperinflation was significantly lower in the ECMO group and virtually inexistent. Tidal hyperinflation was significantly lower in ECMO patients. Compliance of the aerated lung between 5 and 15 cmH_2_O corrected for lung recruitability (CompliancePEEP5-15) was significantly lower in ECMO patients and was independently related to lung aeration at PEEP5 in multivariate analysis.

**Conclusion:** Lung recruitability of COVID-19 pneumonia is not significantly different among class of ARDS severity. ECMO patients exhibits lower hyperinflation levels of already aerated lung with PEEP increase from 5 to 15 cmH_2_O, as a consequence of compliance decrease of the baby lung at low end-expiratory lung volume. This suggests that PEEP higher than 15 could be safely applied in COVID-19 severe ARDS ECMO patients.

**Compliance with ethics regulations:** Yes in clinical research.
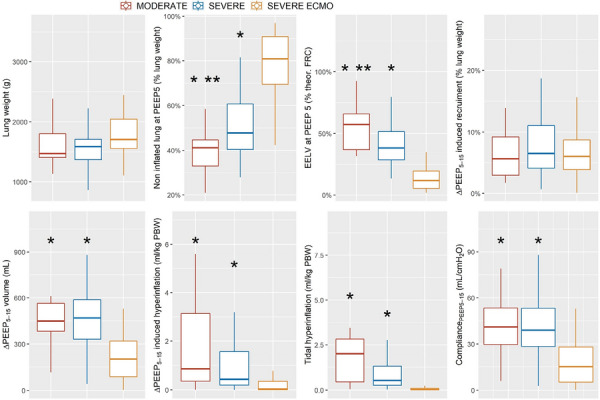


*Figure 1*

### CO-70 Physiological effects of positive end-expiratory pressure titration strategies based on electrical impedance tomography in patients with COVID-19 associated acute respiratory distress syndrome

#### COURTAIS Antonin^1^, CHEAN Dara^1^, PAVLOVSKY Bertrand^1^, LESIMPLE Arnaud^1^, RICHARD Jean Christophe^1^, MERCAT Alain^1^, BELONCLE François^1^

##### ^1^CHU Angers, Angers, France

###### Correspondence: Antonin COURTAIS (courtais.antonin@gmail.com)

*Annals of Intensive Care* 2022, **12(1):**CO-70

**Rationale:** Electrical Impedance Tomography (EIT) allows to provide an imaging of the gas distribution in the lung during ventilation. Various EIT-based strategies have been proposed to titrate positive end-expiratory pressure (PEEP) in patients with acute respiratory distress syndrome (ARDS). This study aimed to compare the physiological effects of two distinct EIT-based and one respiratory system mechanics-based PEEP titration strategies.

**Patients and methods/Materials and methods:** It was a randomized cross-over monocentric study. Patients with moderate to severe COVID-19 associated ARDS were enrolled within 72 h after intubation. Patients were ventilated in volume-controlled ventilation (tidal volume = 6 mL/kg predicted body weight). A decremental PEEP trial (PEEP from 20 to 5 cmH_2_O by steps of 3 cmH_2_O every 3 min) was performed in order to determine the PEEP levels associated with i the lowest Global Inhomogeneity index with a plateau pressure lower than 30 cmH_2_O (*GI strategy*) ii a percentage of collapsed lung tissue lower than 15% and the lowest hyperdistension percentage (*OD-CL strategy*) and iii with a plateau pressure between 28 and 30 cmH_2_0 (*Express strategy*). The PEEP level determined according to the three tested strategies was then applied in a randomized order (three periods of 45 min). Gas exchange, respiratory mechanics including airway and esophageal pressure measurements, hemodynamic and gas distribution using EIT were assessed at the end of each period.

**Results:** Twenty patients have been included in the analysis. PEEP levels determined by the *OD-CL* strategy were lower than those determined by *GI* and *Express* strategies (median [interquartile range], 11 [8–14] cmH_2_O, 20 [17–20] cmH_2_O, 17 [15–18] cmH_2_O, respectively, p < 0.001). PaO_2_/FiO_2_ ratio was higher with *GI* and *Express* strategies compared to *OD-CL* strategy (173 [133–224] mmHg, 158 [135–203] mmHg, 137 [113–172] respectively, p < 0.001). Respiratory system compliance and cardiac output (CO) did not differ between the three PEEP titration strategies. The expiratory transpulmonary pressure was ≤ 2 cmH_2_O in seven patients (35%) with *OD-CL* strategy and three with *Express* and *GI* strategies (p = 0.2). The inspiratory transpulmonary pressure calculated according to the elastance ratio was > 22 cmH2O in four patients with *OD-CL* strategy and five patients (25%) with *Express* and *GI* strategies (p = 0.9).

**Conclusion:** *OD-CL* strategy lead to lower PEEP levels than the *Express* and *GI* strategies. *OD-CL* strategy is associated with decreased oxygenation but similar respiratory mechanics and hemodynamic parameters.

**Compliance with ethics regulations:** Yes in clinical research.

### CO-71 Global Ventilation-Perfusion mismatch improvement related to PEEP increase in patients with highly recruitable lungs

#### PAVLOVSKY Bertrand^1,2^, PESENTI Antonio^2^, SPINELLI Elena^2^, SCARAMUZZO Gaetano^3^, MARONGIÙ Ines^2^, TAGLIABUE Paola^2^, SPADARO Savino^2^, GRASSELLI Giacomo^2^, MERCAT Alain^1^, MAURI Tommaso^2^

##### ^1^CHU Angers, Angers, France; ^2^Fundazione IRCCS Ca'Granda, Ospedale Maggiore Policlinico, Milan, Italie; ^3^Ospedale Universitario Sant'Anna, Ferrara, Italie

###### Correspondence: Bertrand PAVLOVSKY (bertrand.pavlovsky@gmail.com)

*Annals of Intensive Care* 2022, **12(1):**CO-71

**Rationale:** The Acute Respiratory Distress Syndrome (ARDS) is characterized by an important Ventilation-Perfusion (V/Q) mismatch. By recruiting the collapsed lung areas, Positive End Expiratory Pressure (PEEP) may enhance V/Q coupling, by decreasing shunt and allowing a redistribution of ventilation through perfused regions. We hypothesized that higher PEEP may improve V/Q mismatch in ARDS patients with highly recruitable lungs.

**Patients and methods/Materials and methods:** We enrolled fifteen patients with moderate or severe ARDS in this study, within 2 [2–5] days from intubation. Two PEEP levels (5 and 15 cmH_2_O) were applied in a random order. Gas exchange and respiratory mechanics were assessed at each step. V/Q mismatch was evaluated by the Electrical Impedance Tomography (EIT) 5% NaCl bolus method at each PEEP level. Respective fractions of ventilation and perfusion across all V/Q ratios were built, leading to precise assessment of their distribution throughout different V/Q mismatch compartments. The amount of wasted ventilation and perfusion were computed according to this model as follows: sum of (log(V/Q) * V or Q), for all pixels in the functional EIT image within each ROI, including only units with V/Q ratio < 1 and > 1, respectively for wasted ventilation and wasted perfusion. Recruitment between the two PEEP levels was measured by the recruitment-to-inflation ratio (R/I) method.

**Results:** Median age was 60 [48–68] years, and body mass index 27.3 [25.7–35.4] kg.m^−2^. Between PEEP 5 and 15 cmH_2_O, PaO_2_/FiO_2_ increased from 125 [69–194] to 162 [90–198] mmHg (p = 0.011), while PaCO_2_ decreased, albeit non significantly (49.8 [42.0–61.0] vs. 48.0 [42.0–52.1] mmHg, p = 0.403). Respiratory system compliance remained stable (36 [25–50] vs. 32 [24–42] mL.cmH_2_O^−1^, p = 0.164). Patients were characterized by an elevated lung recruitability (R/I ratio 1.29 [1.01–1.53]). In the global population V/Q mismatch improved, as shown by the mean distributions of ventilation and perfusion fractions (Figure, panel A). Both wasted ventilation (17 [14–20] vs. 14 [10–18] %, p = 0.109) and wasted perfusion (16 [13–22] vs. 14 [9–17] %, p = 0.005) decreased at higher PEEP levels. There was a correlation between the R/I ratio and the amount of decrease in both variables from PEEP 5 to 15 cmH_2_O (Figure, Panel B and C).

**Conclusion:** In patients with highly recruitable lungs, V/Q mismatch is improved by higher PEEP levels. This effect is correlated with lung recruitability.

**Compliance with ethics regulations:** Yes in clinical research.
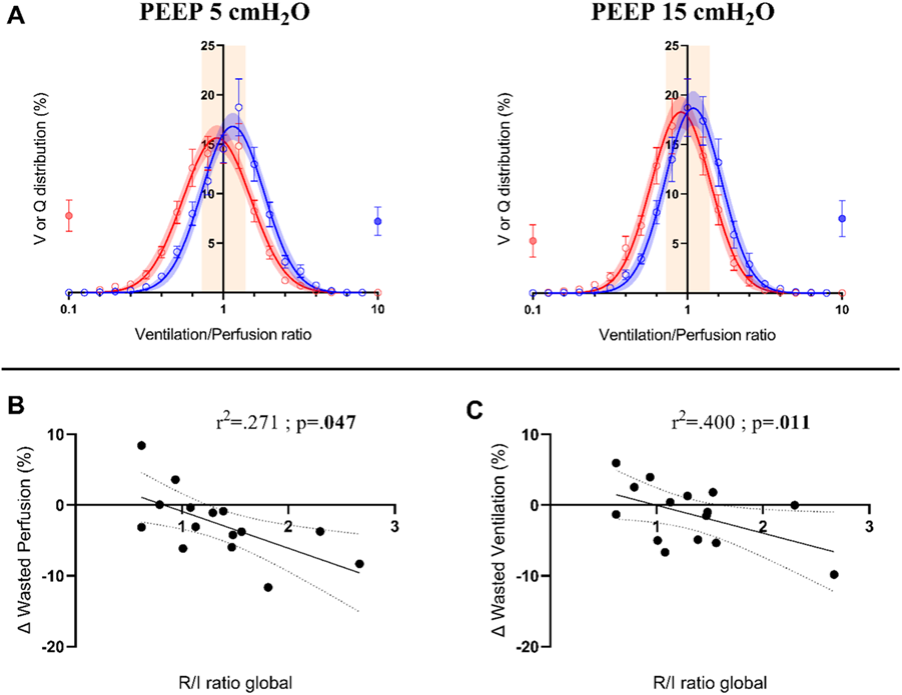


*Distribution of the fraction of ventilation (blue) and perfusion (red) across all V/Q ratios at PEEP 5 and 15 cmH*_*2*_ *O (A). Correlations between R/I ratio and the improvement in Wasted Ventilation (B) and Perfusion (C) between PEEP 5 and 15 cmH*_*2*_ *O.*

### CO-72 Reconnection to mechanical ventilation for 1 h after a successful spontaneous breathing trial in ICU: Physiological effects on alveolar recruitment

#### COUDROY Rémi^1^, LEJARS Alice^1^, RODRIGUEZ Maeva^1^, ARRIVÉ François^1^, REYNAUD Faustine^1^, BOISSIER Florence^1^, VEINSTEIN Anne^1^, CHATELLIER Delphine^1^, ROBERT René^1^, FRAT Jean-Pierre^1^, THILLE Arnaud W.^1^

##### ^1^CHU de Poitiers, Poitiers, France

**Correspondence:** Rémi COUDROY (remi.coudroy@chu-poitiers.fr)

*Annals of Intensive Care* 2022, **12(1):**CO-72

**Rationale:** After a successful spontaneous breathing trial (SBT), reconnection to mechanical ventilation for 1 h is associated with lower reintubation rates than direct extubation1. However, physiological explanations leading to this clinical effect remain unclear. We hypothesized that reconnection to mechanical ventilation for 1 h after a successful SBT induces alveolar recruitment. Our primary aim was to compare end-expiratory lung volume (EELV) at the end of a successful SBT and 1 h after reconnection to mechanical ventilation.

**Patients and methods/Materials and methods:** This is an ancillary study of a multicenter randomized controlled trial comparing T-piece versus pressure-support (pressure support of 8 cmH_2_O without positive end-expiratory pressure) for SBT before extubation. All patients included were at high-risk of extubation failure, i.e. intubated at least 24 h and older than 65 years or having underlying chronic cardiac or lung disease.2 In this physiological single-center study, EELV was measured using the nitrogen washin-washout technique before the SBT under mechanical ventilation, at the end of the SBT, and then 10 min, and 1 h after reconnection to mechanical ventilation. Regional ventilation using electrical impedance tomography was continuously recorded during the study. SBT was performed for around 1 h using T-piece or low pressure-support levels according to the randomization.

**Results:** From the 25 patients analyzed, 11 (44%) had SBTs performed using T-piece and 14 (56%) using pressure-support. Median age was 71 years [interquartile range 67–75] and duration of mechanical ventilation was 8 days [4–13]. All in all, median EELV was 1938 mL [IQR, 1370–2514] before SBT and decreased by 30% [95 CI, 23–37] at the end of the SBT (p < 0.001). The decrease in EELV at the end of the SBT was greater using T-piece than using pressure-support (decrease by 43% [95% CI, 35–51] vs. 20 [95% CI, 13–26], p < 0.001). EELV significantly re-increased from 1305 mL [IQR, 1074–1703] at the end of the SBT to 1927 mL [IQR, 1377–2470] after 1 h of reconnection to mechanical ventilation (mean difference of 587 mL [95% CI, 406–767], p < 0.001). After only 10 min of reconnection to mechanical ventilation, 101% [95% CI, 64–137] of this increased EELV had already been recovered. Regional ventilation was mainly distributed in the non-dependent lung regions and did not differ between T-piece and pressure-support.

**Conclusion:** SBT induced a marked alveolar derecruitment which was significantly greater after SBT using T-piece than using pressure-support. This lung volume loss was almost completely recovered after 10 min of reconnection to mechanical ventilation regardless the type of SBT.

**Reference 1:** Fernandez MM, González-Castro A, Magret M, Bouza MT, Ibañez M, García C, et al. Reconnection to mechanical ventilation for 1 h after a successful spontaneous breathing trial reduces reintubation in critically ill patients: a multicenter randomized control.

**Reference 2:** Thille AW, Coudroy R, Gacouin A, Ehrmann S, Contou D, Dangers L, et al. T-piece versus pressure-support ventilation for spontaneous breathing trials before extubation in patients at high risk of reintubation: protocol for a multicentre, randomised control.

**Compliance with ethics regulations:** Yes in clinical research.

### CO-73 Impact of extra-respiratory stimulations on dyspnea in critically ill mechanically ventilated patient—The sensopnea 2 study

#### BUREAU Côme^1^, NIÉRAT Marie-Cécile^1^, DECAVÈLE Maxens^1^, DANGERS Laurence^1^, CANTIER Marie^1^, VIROLLE Sara^1^, DELERIS Robin^1^, DELEMAZURE Julie^1^, MAYAUX Julien^1^, DRES Martin^1^, SIMILOWSKI Thomas^1^, DEMOULE Alexandre^1^

##### ^1^Hôpital Universitaire Pitié-Salpêtrière, Paris, France

**Correspondence:** Côme BUREAU (come.bureau@aphp.fr)

*Annals of Intensive Care* 2022, **12(1):**CO-73

**Rationale:** Half of patients undergoing mechanical ventilation (MV) in the intensive care unit report a dyspnea of moderate to severe intensity, which causes immediate suffering and post-traumatic stress disorders. Dyspnea has two distinct components, a sensory component and an emotional component. Our objective was to evaluate and to compare the respective impact of a modulation of the sensory component (respiratory afferents) and the emotional component (extra respiratory auditory and sensory stimulations) on the intensity of dyspnea in critically ill patients undergoing MV, either invasive or non-invasive.

**Patients and methods/Materials and methods:** Patients MV for more than 48 h with a dyspnea intensity ≥ 40 mm on a visual analog dyspnea scale (Dyspnea-VAS) were included. We studied the following interventions: (1) increase of pressure support level by 5 cmH2O vs. baseline ventilator settings as a control (sensory component), (2) a relaxing standardized music piece vs. a “pink” noise as a control (emotional component), and (3) fresh air directed toward the face vs. the thigh as a control (emotional component). A washout period separated each condition. Dyspnea was assessed with the Dyspnea-VAS and the A1 score of the Multidimensional Dyspnea Profile. The respiratory drive was assessed by the P0.1 and electromyographic activity of the Alea Nasi and parasternal muscles.

**Results:** We included 46 patients, 19 tracheostomized, 18 intubated and 9 under non-invasive ventilation. Median (interquartile range) age was 63 years (54–73) and duration of mechanical ventilation was 33 days (7–49). Compared to their respective control group the three intervention decreased Dyspnea-VAS: 1) pressure support increment 20 [20–40] mm vs. 70 [60–80], p < 0.0001, 2) auditory stimulation 40 [20–40] vs. 70 [60–80], p < 0.0001 and 3) sensory stimulation 40 [30–50] vs. 60 [50–80], p < 0.0001). Compared to their respective control group the three interventions decreased A1: 1) pressure support increment 5 [4–6] vs. 7 [6–8], p < 0.0001, 2) auditory stimulation 2 [0–2] vs. 7 [6–8], p < 0.0001 and 3) sensory stimulation 3 [2–4] vs. 7 [6–8], p < 0.0001. The electromyographic activity of Alea nasi and parasternal muscles decreased significantly after pressure support increment (p < 0.0001 for both) but did not change during auditory and sensory stimulation. P0.1 decreased more markedly with pressure support increment (3.7 [2.7–5.1] cmH_2_O vs. 6.5 [6.0–8.1], p < 0.0001) than with auditory (6.0 [5.7–7.4] vs. 6.3 [6.0–7.7], p = 0.002) and sensory (6.0 [5.3–7.6] vs. 6.5 [5.7–7.7], p = 0.001) stimulation.

**Conclusion:** In critically ill MV patients, auditory and sensory extra-respiratory stimulations decreased dyspnea without decreasing respiratory drive, suggesting a mechanism involving a modulation of the emotional component.

**Compliance with ethics regulations:** Yes in clinical research.

### CO-74 Accuracy of pulse oximetry (SpO_2_) with different oximeters—Oxygap study

#### BLANCHET Marie-Anne^1^, MERCIER Gabriel^1^, DELOBEL Antoine^1^, NAYET Emi^1^, BOUCHARD Pierre-Alexandre^1^, LELLOUCHE François^1^

##### ^1^Institut Universitaire de Cardiologie et de Pneumologie de Québec, Québec, Canada

###### Correspondence: Marie-Anne BLANCHET (marie-anne.blanchet.2@ulaval.ca)

*Annals of Intensive Care* 2022, **12(1):**CO-74

**Rationale:** An accurate SpO_2_ value is critical in order to optimally titrate the O2 flow or FiO_2_ delivered to patients under oxygen support and to follow oxygenation guidelines. It has been shown with closed-loop oxygen titration that small variations in SpO_2_ target greatly affect the oxygen flow required, which may have a relevant impact on clinical decisions. However, the oximeters’ accuracy appears to vary widely from model to model, leading to an underestimation or an overestimation of actual SaO_2_ values. This variability may represent an obstacle to an optimal delivery of oxygen therapy, including the implementation of guidelines. The objective of this study was to assess the accuracy and bias of the SpO_2_ value measured by several oximeters compared to the reference value, arterial oxygen saturation (SaO_2_) measured by arterial gases in intubated and spontaneously breathing patients in the intensive care unit.

**Patients and methods/Materials and methods:** (ClinicalTrials.gov ID: NCT04772183) The study was approved by the local ethics board with a waiver of consent. We included stable patients hospitalized in the ICU with an arterial catheter in place. Main exclusion criteria were: respiratory instability, poor SpO_2_ signal, infectious isolation, SpO_2_ > 96%. We evaluated six oximeters: Nonin (Plymouth, MN), Massimo (Irvine, CA), Philips (Eindhoven, Netherlands), Nellcor (Pleasanton, CA), Fingers were randomized at each patient. Arterial blood gases were drawn and simultaneously, SpO_2_ values for all oximeters were collected. SpO_2_ value were compared to the reference (SaO_2_ value) to determine bias and accuracy. The ability for oximeters to detect hypoxemia and the impact of oximeters on oxygen titration were evaluated.

**Results:** We included 210 patients (153 men; 57 women, mean age 66.3 ± 11.2 years). The skin pigmentation evaluated by Fitzpatrick showed 96.2% of patients were type 1 or 2. One oximeter overestimated SaO_2_ (Philips, + 0.9%) while the three others underestimated SaO_2_ (Nonin − 3.1%, Nellcor − 0.3%, Masimo − 0.2%). Oxygen saturation was underestimated with Nonin oximeter in 91.3% of the cases while it was overestimated in 55.2% of the cases with Philips oximeter. Hypoxemia was detected in 100%, 26%, 37% and 11% of the cases with Nonin, Nellcor, Masimo and Philips respectively (Table 1).

**Conclusion:** We found large systematic and random errors between the tested oximeters and the arterial blood gases, in the studied population. These discrepancies may have important clinical impact on the detection of hypoxemia or management of oxygen.

**Compliance with ethics regulations:** Yes in clinical research
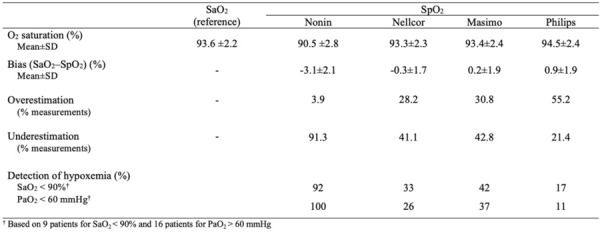


*Table 1. Main features of evaluated oximeters. Mean, standard deviation, percentage of over and underestimation of the SaO*_*2*_ *and detection of hypoxemia for tested oximeters.*

### CO-75 Effective FiO_2_ delivered by a new frugal CPAP system with low oxygen requirements: first clinical observations

#### DE BEAUFORT Eloïse^1,7,8^, MORIN François^2^, CARTEAUX Guillaume^3,7,8^, BOUJELBEN Mohamed^2,7,8^, BROC Alexandre^1^, LESIMPLE Arnaud^1,9^, TAILLANTOU-CANDAU Mathilde^2^, BIZOUARD Thomas^2^, SAVARY Dominique^2,4^, MERCAT Alain^2^, BELONCLE François^2^, BROCHARD Laurent^5,6^, RICHARD Jean-Christophe^1,2^, MEKONTSO-DESSAP Armand^3,7,8^

##### ^1^Air Liquide Medical Systems, Antony, France; ^2^Centre Hospitalier Angers, Angers, France; ^3^Centre Hospitalier Henri Mondor, Créteil, France; ^4^École des hautes études en santé publique, Rennes, France; ^5^St. Michael’s Hospital, Toronto, Canada; ^6^Interdepartmental Division of Critical Care Medicine, University of Toronto, Toronto, Canada; ^7^Institut Mondor de Recherche Biomédicale, Créteil, France; ^8^Université Paris-Est Créteil, Créteil, France; ^9^Institut MITOVASC, Angers, France

###### Correspondence: Eloïse DE BEAUFORT (eloise.debeaufort@airliquide.com)

*Annals of Intensive Care* 2022, **12(1):**CO-75

**Rationale:** In patients with COVID-19 related de novo acute respiratory failure, the application of continuous positive airway pressure (CPAP) improves respiratory mechanics, gas exchange and outcome [1]. In the context of a pandemic with a massive influx of hypoxemic patients, the high oxygen consumption required to achieve optimal inspired oxygen (FiO_2_) may jeopardize health care organization and oxygen delivery hospital capabilities. Within the framework of frugal innovation [2], we have designed a new Bag-CPAP device aiming at meeting oxygen delivery constraints. The aim of these clinical observations was to evaluate the performances of the Bag-CPAP in terms of FiO_2_ actually delivered, oxygen consumption, airway pressure and clinical tolerance.

**Patients and methods/Materials and methods:** The Bag-CPAP is intended to deliver a positive end expiratory pressure (PEEP of 5–10 cmH_2_O) with two levels of FiO_2_: moderate (50%–60%) or high (90%–100%) obtained with an oxygen flow rate of 5 and 15 L/min, respectively. The system operates with a 30L reservoir for gas accumulation to reduce oxygen consumption and guarantee FiO_2_ irrespective of respiratory demand.

After ANSM authorization, the clinical observation was conducted in two university hospitals in France, on 20 adult patients with de novo acute respiratory failure. PEEP level was adjusted at 7.5 cmH_2_O in all patients and FiO_2_ (moderate or high) was selected according to patients’ needs. Actual FiO_2_ and PEEP were regularly measured along with respiratory pattern and dyspnea score. Quantitative data are expressed as median [interquartile range], a P value < 0.05 was considered statistically significant.

**Results:** Median FiO_2_ were 53% [52%–53%] for moderate FiO_2_ target and 94% [93%–96%] for high FiO_2_ target with an oxygen flow rate of 8 [7–9] L/min and 15 [15–16] L/min respectively (Fig. 1). 98% of FiO_2_ values were within the expected target ranges for both moderate and high FiO_2_. Median PEEP was 7 [5–7] cmH_2_O. SpO_2_ significantly increased and Borg dyspnea Scale significantly decreased since the first hour of Bag-CPAP application (93% [90%–97%] vs. 97% [95%–98%], p = 0.001 and 4 [2–7] vs. 3 [2–4], p = 0.017, respectively). No significant effect was observed on respiratory rate.

**Conclusion:** This pilot clinical experience shows that the Bag-CPAP is well tolerated while allowing reaching high FiO_2_ with low oxygen consumption: greater than 50% and 90% with an oxygen flow rate of at least 5 [5 to 10] L/min and 15 [10 to 20] L/min respectively.

**Reference 1:** G. D. Perkins et al., « An adaptive randomized controlled trial of non-invasive respiratory strategies in acute respiratory failure patients with COVID-19», août 2021. https://doi.org/10.1101/2021.08.02.21261379.

**Reference 2:** A. Mekontso Dessap, « Frugal innovation for critical care», Intensive Care Med., vol. 45, no 2, p. 252?254, févr. 2019, https://doi.org/10.1007/s00134-018-5391-6.

**Compliance with ethics regulations:** Yes in clinical research.
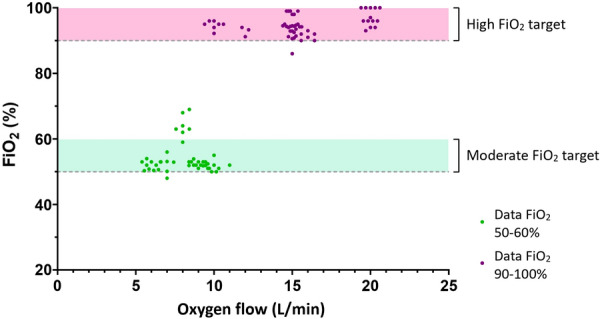


*FiO*_*2*_ *measurements according to oxygen flow rate during Bag-CPAP treatment and FiO*_*2*_ *target range (moderate: green or high: pink)*

### CO-76 Comparison of positive end-expiratory pressure titration strategies based on measured end-expiratory transpulmonary pressure and calculated end-inspiratory transpulmonary pressure in acute respiratory distress syndrome: a physiological study

#### CHEAN Dara^1^, COURTAIS Antonin^1^, PAVLOVSKY Bertrand^1^, YVIN Elise^1^, DESPREZ Christophe^1^, TAILLANTOU-CANDAU Mathilde^1^, RICHARD Jean-Christophe^1^, MERCAT Alain^1^, BELONCLE François^1^

##### ^1^CHU ANGERS, Angers, France

###### Correspondence: Dara CHEAN (dara.chean@gmail.com)

*Annals of Intensive Care* 2022, **12(1):**CO-76

**Rationale:** The optimal positive end-expiratory pressure (PEEP) titration strategy for patients with acute respiratory distress syndrome (ARDS) is still a matter of debate. Esophageal pressure monitoring allows to estimate pleural and transpulmonary pressures. Two distinct strategies based on measured end-expiratory transpulmonary pressure (P_L, exp_) or calculated end-inspiratory transpulmonary pressure (P_L, insp calc_) have been described but their physiological effects have never been compared to each other. This study aimed at comparing the short-term ventilatory and hemodynamic effects of these two strategies in patients with ARDS.

**Patients and methods/Materials and methods:** P_L, exp_ was computed as the difference between total PEEP and esophageal pressure measured at end-expiration. In P_L, exp_-based PEEP titration strategy, PEEP was set to maintain P_L, exp_ between 0 and 6 cmH_2_O using a P_L, exp_-FiO_2_ table^1^. In P_L, insp calc_-based PEEP titration strategy, P_L, insp calc_ was estimated using the lung to respiratory system elastance ratio and PEEP was set to achieve a P_L, insp calc_ between 20 and 22 cmH_2_O^2^. P_L, exp_-based and P_L, insp calc_-based strategies were consecutively applied in a randomized order in patients with moderate to severe ARDS. Gas exchange, respiratory mechanics, hemodynamics and ventilation regional distribution assessed with electrical impedance tomography were evaluated 45 min after the application of each PEEP titration strategy.

**Results:** Twenty patients were included in this study. Twelve (60%) patients had SARS-CoV2 associated pneumonia. The two different PEEP titration strategies led to similar median levels of PEEP (median [interquartile range], 14 [9–17] cmH_2_O with P_L, exp_-based strategy vs 16 [12–18] cmH_2_O with P_L, insp calc_-based strategy, p = 0.11) but 14 (70%) patients presented a difference of at least 3 cmH_2_O between the two determined PEEP levels. PaO_2_/FiO_2_, shunt fraction, dead-space, cardiac index and oxygen delivery were similar between the two strategies. The 12 (60%) patients for whom P_L, insp calc_-based strategy was associated with higher PEEP levels than P_L, exp_-based strategy were characterized by a lower body mass index. Compared to P_L, exp_-based strategy, P_L, insp calc_-based strategy was associated, in these patients, with better oxygenation but lower cardiac output, higher transpulmonary driving pressure, lower respiratory system compliance and lower non-dependant regional compliance, suggesting lung overdistension.

**Conclusion:** PEEP levels defined by the two distinct esophageal pressure measurements-based strategies differed of at least 3 cmH_2_O in 70% of ARDS patients. P_L, insp calc_-based strategy may be associated with lung overdistension in some patients.

**Reference 1:** Talmor, D. et al. Mechanical ventilation guided by esophageal pressure in acute lung injury. N. Engl. J. Med. 359, 2095–2104 (2008).

**Reference 2:** Grasso, S. et al. ECMO criteria for influenza A (H1N1)-associated ARDS: role of transpulmonary pressure. Intensive Care Med 38, 395–403 (2012).

**Compliance with ethics regulations:** Yes in clinical research.

### CO-77 Respiratory effects of lung recruitment maneuvers depend on the recruitment-to-inflation ratio

#### ZERBIB Yoann^1^, LAMBOUR Alexis^1^, MAIZEL Julien^1^, KONTAR Loay^1^, DE CAGNY Bertrand^1^, SOUPISON Thierry^1^, BRADIER Thomas^1^, SLAMA Michel^1^, BRAULT Clément^1^

##### ^1^CHU Amiens-Picardie, Amiens, France

**Correspondence:** Clément BRAULT (brault.clement@chu-amiens.fr)

*Annals of Intensive Care* 2022, **12(1):**CO-77

**Rationale:** In the context of acute respiratory distress syndrome (ARDS), the response to lung recruitment maneuvers (LRM) varies considerably from one patient to another. The LRM can be harmful, especially in patients with low recruitability, inducing lung overdistention and cardiac dysfunction. Recently, a single-breath maneuver with measurement of the recruitment-to-inflation (R/I) ratio has been developed to assess lung recruitability and identify patients who could benefit from the application of positive pressure. Here, we determined whether or not the R/I ratio could differentiate between patients according to the change in lung mechanics during LRM.

**Patients and methods/Materials and methods:** We included all adults patients admitted with a diagnosis of ARDS and an arterial oxygen partial pressure to fractional inspired oxygen (PaO_2_/FiO_2_) ratio lower than 150 mmHg. Starting at 20 cmH_2_O, the PEEP was increased in 5 cmH_2_O steps to 40 cmH_2_O, with each step lasting 2 min. We evaluated the changes in gas exchange, respiratory mechanics, and hemodynamic parameters induced by a stepwise LRM at a constant driving pressure of 15 cmH_2_O during pressure-controlled ventilation. Patients were dichotomized with regard to the median R/I ratio.

**Results:** We included 30 patients with a median [interquartile range] R/I ratio of 0.62 [0.42-0.83]. The PaO2/FiO2 ratio increased significantly after LRM in patients with low and high lung recruitability (Table 1). However, the mechanisms behind this improvement in oxygenation depend on each patient’s potential for alveolar recruitment, as measured by the R/I ratio. In patients with high recruitability (R/I ratio ≥ 0.62), the static respiratory system compliance (Crs) increased significantly after the LRM (33 [27–42] vs. 42 [35–60] mL/cmH_2_O; p < 0.001). There was a moderate but significant positive correlation between the change in Crs during the LRM and the R/I ratio (r = 0.56; p = 0.001). In patients with low recruitability (R/I ratio < 0.62), the increase in PaO_2_/FiO_2_ ratio was associated with a significant decrease in pulse pressure (70 [55–85] vs. 50 [51–67] mmHg; p = 0.01) but not with a significant change in Crs (33 [24–47] vs. 35 [25–47] mL/cmH_2_O; p = 0.74).

**Conclusion:** During an LRM, the mechanisms related to an increase in oxygenation depend on the potential for lung recruitment. Patients with high recruitability presented a significant increase in Crs (indicating a gain in ventilated area), while those with low recruitability presented a decrease in pulse pressure suggesting a drop in cardiac output and therefore in intrapulmonary shunt. The R/I ratio might help clinicians to identify patients in whom an LRM will lead to an increase in Crs.

**Compliance with ethics regulations:** Yes in clinical research.
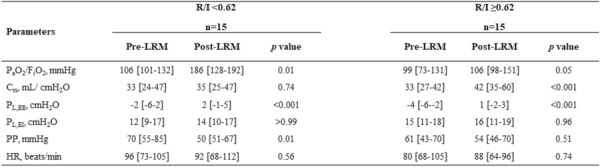


*Respiratory and hemodynamic parameters before and after the LRM, as a function of the patients’ lung recruitability*

### CO-78 Chest electrical impedance tomography and lung ultrasound to monitor extubation

#### JOUSSELLIN Vincent^1,2^, JANIAK Vincent^3,4^, BONNY Vincent^1,2^, CLERC Sébastien^1^, MAYAUX Julien^1^, MORAWIEC Elise^1^, TALLEC Gwendolyne^4^, SALEEM Umar^4^, PINNA Andrea^3^, DRES Martin^1,2^

##### ^1^AP-HP. Sorbonne Université, Hôpital Pitié-Salpêtrière, Service de Médecine Intensive – Réanimation (Département "R3S"), Paris, France; ^2^Sorbonne Université, INSERM, UMRS1158 Neurophysiologie respiratoire expérimentale et clinique, Paris, France; ^3^Sorbonne Université, CNRS, LIP6, Paris, France; ^4^Bioserenity, Paris, France

###### Correspondence: Vincent JOUSSELLIN (vincent.joussellin@gmail.com)

*Annals of Intensive Care* 2022, **12(1):**CO-78

**Rationale:** Chest Electrical Impedance Tomography (EIT) is a non-invasive technique that produces continuous cross-sectional images of regional lung ventilation. This study investigated the use of chest EIT and lung ultrasound in patients extubated after a successful spontaneous breathing trial. The hypothesis was that patients with extubation failure may exhibit early regional lung ventilation disturbances and higher lung ultrasound score (LUS) as compared to patients with extubation success.

**Patients and methods/Materials and methods:** Patients at high risk of extubation failure (> 65 years old, chronic cardiac or pulmonary disease) were included after a successful spontaneous breathing trail and after extubation was planned by the physicians. Lung ultrasound (with calculation of LUS score as a surrogate of loss of lung aeration) and chest EIT (with calculation of derived indices, such as Global inhomogeneity index (GI), front-back Centre of ventilation (CoV), Regional ventilation delay (RVD) and Surface available for ventilation) were performed before (H0) and two hours and six hours after extubation (H2 and H6 respectively). The primary outcome was the proportion of patients who presented extubation failure (defined as acute respiratory failure requiring re-intubation or not or death within 48 h after extubation).

**Results:** Thirty-four patients were included, of whom 10 (29%) were considered with extubation failure. Before extubation, the LUS score was higher in the extubation failure group as compared to patients who were successfully extubated (20 vs 11, p < 0,01). However, EIT derived indices were not different between groups. After extubation, GI index, RVD and LUS score were higher in the extubation failure group, whereas Surface was lower and the CoV didn’t change (See an example in Fig. 1).

**Conclusion:** Before extubation, a loss of lung aeration was observed in patients who developed extubation failure afterwards. After extubation, this loss persisted with adjunction of heterogeneity in air distribution observed with EIT.

**Compliance with ethics regulations:** Yes in clinical research.
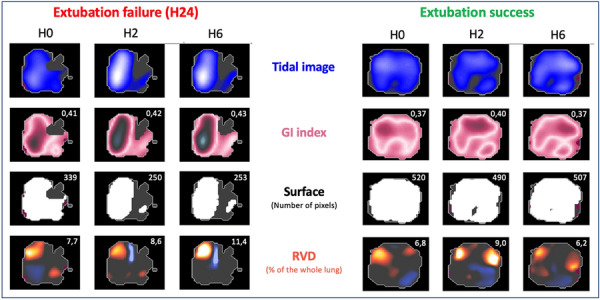


*EIT derived indices in a patient who presented extubation failure 24 h after extubation compared to a patient who were successfully extubated GI index: Global inhomogeneity index; RVD: Regional ventilation delay; H0: just before extubation; H2 and H6:*

### CO-79 Clinical and microbiological features of drowning associated pneumonia: a multicenter cohort study

#### REIZINE Florian^1^, AGATHE Delbove^2^, DOS SANTOS Alexandre^3^, BODENES Laetitia^4^, BOUJU Pierre^5^, FILLÂTRE Pierre^6^, FRÉROU Aurélien^7^, HALLEY Guillaume^8^, LESIEUR Olivier^9^, COUROUBLE Patricia ^10^, BERTEAU Florian^11^, MORIN Jean^12^, DELAMAIRE Flora^1^, MARNAI Rémy^13^, LE MEUR Anthony^14^, AUBRON Cécile^4^, REIGNIER Jean^12^, GACOUIN Arnaud^1^, TADIÉ Jean-Marc^1^

##### ^1^CHU de Rennes, Rennes, France; ^2^CH Vannes, Vannes, France; ^3^CH La Roche sur Yon, La Roche Sur Yon, France; ^4^CHU Brest, Brest, France; ^5^CH Lorient, Lorient, France; ^6^CH Saint Brieuc, Saint Brieuc, France; ^7^CH Saint Malo, Saint Malo, France; ^8^CH Quimper, Quimper, France; ^9^CH La Rochelle, La Rochelle, France; ^10^CH Saint Nazaire, Saint Nazaire, France; ^11^CH Morlaix, Morlaix, France; ^12^CHU Nantes, Nantes, France; ^13^CH Le Mans, Le Mans, France; ^14^CH Cholet, Cholet, France

###### Correspondence: Florian REIZINE (florian.reizine@gmail.com)

*Annals of Intensive Care* 2022, **12(1):**CO-79

**Rationale:** Pneumonia is the most frequent infectious complication among drowning patients requiring ICU admission. However, clinical and microbiological data on such pneumonia are scarce.

**Patients and methods/Materials and methods:** We conducted a retrospective multicenter study (2013–2020) of 270 consecutive patients admitted for drowning to 14 ICUs in the west of France. Baseline characteristics as well as clinical course of patients were compared according to the occurrence of drowning associated pneumonia (DAP) (diagnosed within 48 h of ICU admission). Pneumonia microbiological features and therapeutic strategies were also analyzed.

**Results:** Among the 270 patients admitted to the ICUs for drowning, 101 (37.4%) experienced pneumonia. Suicidal etiology of drowning, psychiatric comorbidities, and need for mechanical ventilation appeared to be risk factors for drowning associated pneumonia (DAP). DAP occurred less frequently in patients initially treated by antibiotics. Pneumonia occurrence was associated with higher severity scores at ICU admission (Median SAPS 2 score 34 [Interquartile range 25–55] versus 45 [28–67]; p = 0.006), longer ICU LOS (2 days [1–3] versus 4 [2–7]; p < 0.001), mechanical ventilation duration (2 days [1–4] versus 4.5 days [2–8]; p < 0.001), higher SOFA score at day 3 (1 [0-3] versus 5 [1–10]; p = 0.001) These patients also experienced acute respiratory distress syndrome (ARDS) more frequently (28.4% versus 53.5%; p < 0.001). Survival at day-28 appeared lower among these patients particularly in patients developing microbiologically proven pneumonia (14/33 versus 201/237; p < 0.0001). Interestingly, microbiological analysis of respiratory samples showed a high proportion of gram-negative bacilli (39.6%) that did not differ according to water salinity. Empirical antimicrobial management seemed frequently inappropriate (30.3%) mostly due to high proportions of resistance to Amoxicillin–Clavulanate on isolates (39.4%).

**Conclusion:** Pneumonia occurrence was found to be a common complication in critical drowning patients that worsen patients’ clinical courses. Due to a high proportion of resistant gram-negative bacilli among isolated pathogens, initial empirical antimicrobial strategy with Amoxicillin–Clavulanate should be discouraged.

**Compliance with ethics regulations:** Yes in clinical research.

### CO-80 Prognosis of adult patients hospitalized with respiratory syncytial virus infection: a multicenter retrospective observational cohort study

#### CELANTE Héloïse^1^, OUBAYA Nadia^1^, LAYESE Richard^1^, DE PROST Nicolas^1^

##### ^1^Hôpital Henri-Mondor, Creteil, France

###### Correspondence: Héloïse CELANTE (heloise.celante@aphp.fr)

*Annals of Intensive Care* 2022, **12(1):**CO-80

**Rationale:** Respiratory syncytial virus (RSV) is a common agent of viral respiratory infections. It causes significant morbidity and mortality in adults, especially in those with cardiorespiratory comorbidities and immunosuppression. Although there is a high burden of RSV respiratory infections in frail adults, few data are available in hospitalized patients. We set up a retrospective multicenter cohort to obtain large-scale data aiming at better depicting the clinical profile and prognosis of patients hospitalized with RSV infection.

**Patients and methods/Materials and methods:** Retrospective multicenter observational cohort study conducted in all Assistance Publique-Hôpitaux de Paris (AP-HP) hospitals, including patients hospitalized between January 1, 2015 and December 31, 2019 for documented respiratory RSV infection. Data were extracted from the AP-HP Health Data Warehouse (EDS). The selection of patients and the procedures performed in intensive care units (ICUs, n = 13) were identified using International Classification of Disease (ICD) 10 and CCAM (Classification Commune des Actes Médicaux) coding. The primary endpoint was in-hospital mortality.

**Results:** 1168 patients were hospitalized for RSV infection, including 880 patients admitted to 14 conventional inpatient units and 288 patients who required ICU admission. The median age of patients was 75 years, 54% of them were women. Patients frequently had comorbidities, most often cardiovascular (hypertension: 46.4%; heart failure: 34.4%), respiratory (28.6%), or immunodepression (29.5%). In-hospital mortality was 6.6% (n = 77/1168) in the whole cohort and 12.8% (n = 37/288) in ICU patients. By multivariable logistic regression, the factors associated with hospital mortality were age (adjusted odds ratio (aOR) = 1.03 95% CI [1.01–1.06], p = 0.006), presence of an acute respiratory failure at ICU admission (aOR = 2.35 [1.30–4.25], p = 0.04), acute respiratory distress syndrome (aOR = 6.49 [2.96-14.21], p < 0,001) and neutropenia (aOR = 5.57 [1.54–20.19], p = 0,009). 30% of patients admitted to the ICU required invasive mechanical ventilation support. Risk factors for intubation were age (aOR = 0.98 [0.96–0.99], p = 0.002), a previous chronic respiratory failure (aOR = 2.83 [1.72–4.65], p < 0,001), chronic heart disease (aOR = 1.90 [1.15–3.13], p = 0.012), obesity (aOR = 1.94 [1.09–3.45], p = 0.025) and the documentation of a bacterial co-infection (aOR = 2.51 [1.55–4.07], p < 0.001). A bacterial co-infection was documented in 18.2% of patients. Streptococcus pneumoniae and Pseudomonas aeruginosawere the two most frequently isolated bacteria.

**Conclusion:** In this large multicenter retrospective study of patients hospitalized with RSV infection, we found a mortality rate of 6.6%. Risk factors associated with mortality were age, the presence of acute respiratory distress, ARDS and neutropenia.

**Compliance with ethics regulations:** N/A.

### CO-81 Differences in clinical characteristics and outcomes between Covid-19 and influenza in critically ill adult patients: a national database study

#### NAOURI Diane^6^, PHAM Tai^2^, DEMOULE Alexandre^2^, MERCAT Alain^5^, BEDUNEAU Gaetan^3^, COMBES Alain^2^, KIMMOUN Antoine^4^, SCHMIDT Matthieu^2^, DRES Martin^2^, JAMME Matthieu^1^

##### ^1^Hopital privé de l'ouest parisien, Trappes, France; ^2^Assistance publique hopitaux de Paris, Paris, France; ^3^CHU Rouen, Rouen, France; ^4^CHU Nancy, Nancy, France; ^5^CHU Angers, Angers, France; ^6^Direction de la recherche, des études, de l'évaluation et des statistiques, Paris, France

###### Correspondence: Matthieu JAMME (mat.jamme@gmail.com)

*Annals of Intensive Care* 2022, **12(1):**CO-81

**Rationale:** Until March 2020 and the emergence of coronavirus disease 2019 (COVID-19), influenza was the leading cause of viral acute respiratory failure observed in ICU. While a few studies have compared flu patients vs. COVID-19, none of them have focused on the most severe forms. The aim of this study is to compare ICU patients admitted for COVID-19 vs. those admitted with influenza.

**Patients and methods/Materials and methods:** We conducted a retrospective cohort study using the French administrative health database (Système National des Données de Santé, SNDS). We included all adult patients hospitalised in French ICUs for whom a complete hospital history was available from 1 March 2020 to 30 June 2021. For the comparative group (influenza cohort), all adult patients hospitalised in ICUs from 1 January 2014 to 31 December 2019 were included. To be included in one of these two groups, the patient had to have an ICD-10 diagnosis code of COVID-19 or influenza regardless of its position in the hospital stay summary. The Chi-square test, Student’s t test or Wilcoxon test were used, as appropriate, to compare characteristics between patients with COVID-19 and influenza. To identify risk factors for invasive mechanical ventilation (IMV) and in-hospital death, we performed multivariate logistic regression models.

**Results:** Our study cohort included 105.979 COVID-19 patients and 18.763 influenza patients. Compared to influenza patients, COVID-19 patients were younger (p < 0.0001), more often male (p < 0.0001), and had a lower SAPS II score at ICU admission (p < 0.0001). During the ICU stay, patients with influenza more frequently required the use of iMV (47% versus 34%, p < 0.001), vasopressors (40% versus 27%, p < 0.001) and renal replacement therapy (22% versus 7%, p < 0.001). In-hospital mortality was higher in COVID-19 patients (25% vs. 21%, p < 0.001), especially in patients with iMV (40% vs. 33%, p < 0.001). After adjustment, while we did not observe an association between virus type and iMV use, we found a strong association between COVID-19 and in-hospital mortality compared to influenza (aOR = 1.92 [1.83–2.00], p < 0.001).

**Discussion:**

**Conclusion:** Using a very large cohort study, patients admitted for COVID-19 have a high probability of in-hospital death compared to patients admitted for influenza. This excess mortality does not appear to be related to a higher proportion of organ failure, particularly with respect to iMV use.

**Compliance with ethics regulations:** Yes in clinical research.

### CO-82 Lung microbiome in critical care: evolution and risk factors of VAP

#### FROMENTIN Mélanie^1,2^, MULLAERT Jimmy^2^, BRIDIER-NAHMIAS Antoine^2^, MERCIER-DELARUE Séverine^4^, VUILLARD Constance^2^, DO VALE Julien^2^, SALMONA Maud^4^, LE GOFF Jerome^4^, RICARD Jean-Damien^2,3^, ROUX Damien^2,3^

##### ^1^Ramsay Santé, Hopital privé Paul d'Egine, Champigny-Sur-Marne, France; ^2^Université de Paris, INSERM, U 1137 IAME Infection Antimicrobials Modelling Evolution, Paris, France; ^3^AP_HP, Hopital universitaire Louis Mourier, Colombes, France; ^4^Université de Paris, INSERM, U 976, HIPI Human Immunology, Pathophysiology & Immunotherapy, Paris, France

###### Correspondence: Mélanie FROMENTIN (mfromentin.anesthesierea@gmail.com)

*Annals of Intensive Care* 2022, **12(1):**CO-82

**Rationale:** We aimed to characterize and compare the temporal changes of lung bacterial microbiome whether ICU patients develop ventilator-associated pneumonia (VAP) or not. Our main objective was to identify early specific bacterial markers in the airway microbiome associated with the risk of ventilator-associated pneumonia (VAP) occurrence.

**Patients and methods/Materials and methods:** We conducted a case-control study based on a prospective cohort of mechanically ventilated (MV) patients. Controls were matched to VAP patients according to known risk factors of lung microbiota changes. We sequenced the specific V4 hypervariable region of 16S rRNA gene in samples collected every 72 h from oropharynx (OPS = oropharyngeal swabs) and lungs (ETA = endotracheal aspiration). The co-evolution of lung and oropharyngeal microbiota were described using non-linear mixed effects models.

**Results:** Nineteen VAP patients and 19 matched controls were included. We identified 281 taxa or operational taxonomic unit in 293 samples distributed in four predominant phyla. The dynamic evolution of the lung microbiota highlighted a constant increase in relative abundance of *Bacteroidetes* whereas these of *Firmicutes* decreased under mechanical ventilation (MV) (Fig. 1). On ICU admission, the *Bacteroidetes* phylum (p = 0.017) and the *Bacteroidia* class (p = 0.018) were more represented in patients who eventually developed a VAP. For all patients, the dynamic study highlighted a highly significant positive correlation between changes in lung and oropharyngeal microbiota for diversity (r = 0.87 (0.42;1), p < 0.001), for relative abundance of *Bacilli* (r = 0.91 (0.77;1), p < 0.001) and for relative abundance of *Bacteroidia* (r = 0.99 (0.94;1), p < 0.001). There was a significant decrease of the alpha diversity in both OPS (p < 0.001) and ETA during MV (p = 0,006), and a significant decrease of relative abundance of *Bacilli* in ETA (p < 0.001). On average, the relative abundance of *Bacteroidia* in the lungs was increased by an additional 14% per day of MV in VAP patients compared to controls (p = 0.025). The RA of *Bacilli* in the oropharynx decreased by an additional 9% per day of MV in VAP patients compared to controls (p = 0.049).

**Conclusion:** A lower initial RA of Bacteroidia, a faster decrease of Bacilli within the oropharynx as well as a faster increase of Bacteroidia within the lungs were associated with VAP development. This could represent a dysbiosis pattern that predisposed ICU patients to VAP. We aimed to characterize and compare the temporal changes of lung bacterial microbiome whether ICU patients develop ventilator-associated pneumonia (VAP) or not.

**Compliance with ethics regulations:** Yes in clinical research.
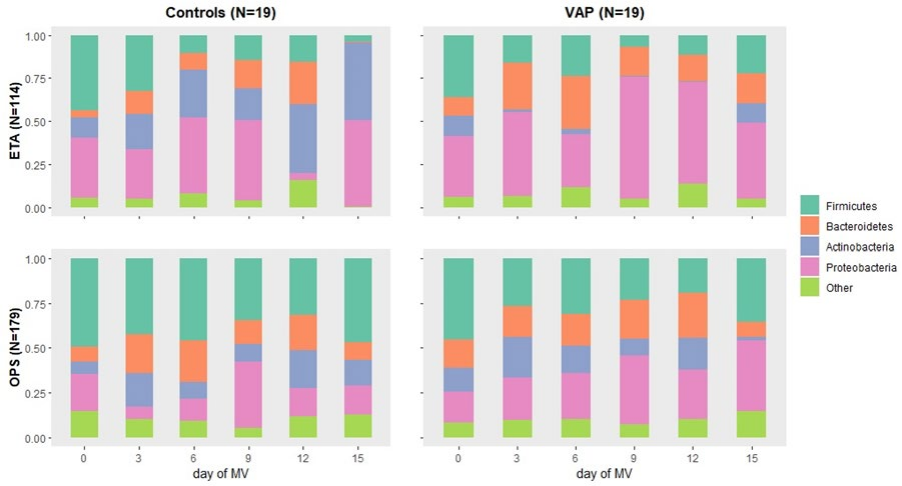


*Evolution of relative abundance of the four main phyla identified in the respiratory microbiota*

### CO-83 Involvement of lung epithelium in sepsis-induced immunosuppression

#### MALHERBE Jolan^1^, LADJEMI Maha Zohra^1^, ROUSSEAU Christophe^1^, PÉJU Edwige^1,2^, LLITJOS Jean-François^1^, BOULANT Léa^1^, MARTIN Clémence^1,3^, BURGEL Pierre-Régis^1,3^, PÈNE Frédéric^1,2^

##### ^1^Institut Cochin, Inserm U1016, CNRS UMR8104, Université de Paris, Paris, France; ^2^Service de Médecine intensive & Réanimation, Hôpital Cochin, Assistance Publique-Hôpitaux de Paris, Paris, France; ^3^Service de Pneumologie & Centre National de Référence Mucoviscidose, Hôpital Cochin, Assistance Publique-Hôpitaux de Paris, Paris, France

###### Correspondence: Jolan MALHERBE (jolan.malherbe@gmail.com)

*Annals of Intensive Care* 2022, **12(1):**CO-83

**Rationale:** Ventilator-associated pneumonia accounts for a leading cause of death in patients with septic shock. Altered lung immunity in post-septic hosts has been consistently ascribed to both quantitative and functional defects in most hematopoietic immune cells. In contrast, the role of airway epithelium has not been investigated in this setting despite its key role in first-line lung defense through various functions of physical barrier, tissue repair, pathogen sensing and crosstalk with immune cells. We raised the hypothesis that sepsis-induced alterations in airway epithelium contribute to defective lung defense towards secondary bacterial pneumonia. Our main goal was to assess the morphological and functional alterations of airway epithelium following non-pulmonary polymicrobial sepsis.

**Patients and methods/Materials and methods:** C57BL/6J female mice were used for the experiments. Mice were subjected to double-hit infectious insults through polymicrobial peritonitis induced by cecal ligation and puncture (CLP) or control sham surgery, followed eight days after by intratracheal instillation of *Pseudomonas aeruginosa*. The morphology and functions of lung epithelium were studied by immunohistochemistry and flow cytometry on days 3 and 7 after surgery, prior to any secondary bacterial challenge.

**Results:** As compared to sham-operated controls, post-CLP mice exhibited increased mortality towards secondary *P. aeruginosa* pneumonia, associated with increased systemic spread of the pathogen. Lung sections from post-CLP and sham-operated mice were subjected to histological analyses. Post-CLP mice exhibited reduced thickness of bronchial epithelium on day 3, associated with decreased proliferative capacity (as assessed by Ki67 immunostaining). However, bronchial epithelium became hypertrophied in post-CLP mice at delayed time point (day 7), thereby suggesting altered tissue repair. Tissue barrier functions were also altered as suggested by reduced epithelial expression of tight and adherens junctions (as assessed by *Zonula occludens* 1 and E-cadherin immunostainings). Increased expression of immune checkpoint inhibitors (day 3 post-sepsis) and Toll-like receptors 2 and 5 (day 7) were also observed in bronchial and alveolar epithelial cells from post-septic mice.

**Conclusion:** Our results suggest that non-pulmonary sepsis may modulate the main functions of airway epithelial cells and may thereby impact on lung compartmentalization. How this may affect mucosal immunity, possibly in relation with local dysbiosis, should now be investigated.

**Compliance with ethics regulations:** Yes in animal testing.

### CO-84 Risk factors and outcomes of recurrent ventilator associated pneumonia in COVID-19 patients: a retrospective multicentric study

#### GRAGUEB CHATTI Ines^1^, HYVERNAT Herve^2^, LOPEZ Alexandre^1^, AGARD Geoffray^1^, PAPAZIAN Laurent^1^, DELLAMONICA Jean^2^, LEONE Marc^1^, HRAIECH Sami^1^

##### ^1^APHM, Marseille, France; ^2^CHU L'Archet, Nice, France

###### Correspondence: Sami HRAIECH (sami.hraiech@ap-hm.fr)

*Annals of Intensive Care* 2022, **12(1):**CO-84

**Rationale:** High incidence of ventilator associated pneumonia (VAP) has been reported among critically ill patients with COVID-19. Among these patients, we aimed to specifically assess the frequency, risk factors and outcomes of recurrences of VAP.

**Patients and methods/Materials and methods:** We conducted an observational retrospective study in 3 French intensive care units (ICUs). Patients admitted for a documented COVID-19 from March 2020 to May 2021 and requiring mechanical ventilation (MV) for ≥ 48 h were included. The study main outcome was the rate of VAP recurrence. Secondary outcomes were the risk factors for recurrence, the Day-28 and Day-60 ventilator-free days (VFD), the ICU and hospital length of stay and the Day-90 mortality.

**Results:** From 26th February 2020 to 21th May 2021, 398 patients were included in the analysis. Among them, 236 (59%) presented at least one VAP and 109 (27%) presented more than one VAP during the ICU stay (VAP recurrence). The overall ICU mortality rate was 27.9%, and day-90 mortality was 28.6%. After 90 days follow-up, the day-90 mortality rate of patients without VAP was significatively lower than mortality of patients with one VAP or more (p = 0.021). In contrast, there was no day-90 mortality difference between patients with one VAP or more than one (recurrence) (36.2% vs 31.2%). Patients with VAP recurence had a longer duration of mechanical ventilation, less VFD at day-28 and day-60, a longer ICU and hospital length of stay. In multivariate analysis, a bacterial co-infection at ICU admsision, the use of dexamethasone, the association of 2 immunosuppressive therapies or more during the ICU stay, the duration of antibiotic therapy, the type of bacteria responsible for the 1st occurence of VAP were not significantly associated with VAP recurrence.

**Conclusion:** VAP recurrences are frequent among COVID-19 ICU patients and are associated with a longer MV duration, ICU and hospital length of stay but not a higher mortality as compared with patients that had only one VAP. Bacterial co-infection at ICU admsision, the use of dexamethasone, the association of 2 immunosuppressive therapies or more during the ICU stay, the duration of antibiotic therapy or the type of bacteria responsible for the 1st occurence of VAP were not identified as independant risk factors of VAP recurrence in our cohort.

**Compliance with ethics regulations:** Yes in clinical research.

### CO-85 Severe acute kidney injury in severe SARS COV2: “For a time they are a-changin’” For the Kidney Injury Working Initiative in Critically ill eurOpean patients during Coronavirus Outbreak (KIWI COCO)

#### CHAÏBI Khalil^1^, LOUIS Guillaume^4^, BOUBAYA Marouane^1^, BONNET Nicolas^1^, PAVOT Arthur^2^, ROUX Damien^5^, PICARD Yoann^5^, MELLATI Nouchan^4^, PHAM Tài^2^, GUMUCIO Victor Daniel^3^, DI PAOLO Fabio A.^3^, COHEN Yves^1^, DREYFUSS Didier^5^, PÉREZ-FERNANDEZ Xosé Luis^3^, GAUDRY Stephane^1^

##### ^1^CHU Avicenne, Bobigny, France; ^2^CHU Kremlin Bicêtre, Kremlin Bicêtre, France; ^3^Bellvitge University Hospital, Barcelone, Espagne; ^4^CHR Metz Thionvile, Metz, France; ^5^CHU Louis Mourier, Colombes, France

###### Correspondence: Khalil CHAÏBI (khalilchaibi@gmail.com)

*Annals of Intensive Care* 2022, **12(1):**CO-85

**Rationale:** Severe acute kidney injury (AKI) is frequent among critically-ill patients with SARS-COV2 infection and associated with high mortality[1]. The epidemiological changes in the rate of severe AKI according to the epidemic waves remain unclear. We evaluated the differences in incidence, risk factors and outcome of severe AKI in patients with SARS-CoV-2 infection between the first and third wave of COVID-19.

**Patients and methods/Materials and methods:** We performed a European multicenter, retrospective observational study in five hospitals in France and Spain from March 1 to March 31, 2020 (first wave) and from March 1 to March 31, 2021 (third wave). We included consecutive adult patients with acute respiratory distress syndrome (ARDS) and testing positive for SARS-CoV-2 in respiratory fluids. Patients with end-stage renal disease or who had sustained cardiac arrest before ICU admission were not included. We reviewed clinical electronic medical records, nursing records, laboratory findings, demographic data, comorbidities, ICU usual parameters, occurrence of severe AKI (defined by KDIGO stage 3) in the first 7 days after ICU admission and survival outcome 28 days after ICU admission.

**Results:** A total of 445 patients with severe SARS COV2 pneumonia met the inclusion criteria of whom 293 (66%) were admitted during the first wave and 152 (34%) during the third wave. Patients admitted during the third wave had respectively more chronic kidney disease (CKD) at baseline (16.6% VS 7.2% p = 0.020), received more iodinated contrast media (56.6% VS 3.1%), more aminoglycosides (3.9% VS 0.3% p = 0.007) but were less severely ill (SOFA 5 VS 7 p = 0.006). One hundred two (23%) patients experienced severe AKI in the first 7 days of ICU admission. Its cumulative incidence was higher during the first wave than the third (26.8% VS 16.8% p = 0.029) (Fig. 1). In a multivariate Fine and Gray analysis, BMI ≥ 30 was a risk factor of severe AKI during the first (SHR [95CI%] 1.29 [0.81–2.04] p = 0.28) and third wave (SHR [95CI%] 3.35 [1.16–9.64] p = 0.025) whereas CKD was a risk factor only during the first wave (SHR [95CI%] 2.56 [1.23–5.31] p = 0.012). Steroid’s administration was not associated with the occurrence of severe AKI.

**Conclusion:** In this multicenter retrospective study including COVID-19 patients with ARDS, severe AKI was more frequent during the first epidemic wave compared to the third one. This may be explained by improvement in critical care delivery and a greater attention to renal impairment.

**Reference 1:**. Chaibi K, Dao M, Pham T, Gumucio-Sanguino VD, Di Paolo FA, Pavot A, et al. Severe Acute Kidney Injury in Patients with COVID-19 and Acute Respiratory Distress Syndrome. Am J Respir Crit Care Med. 2020;202:1299–301.

**Compliance with ethics regulations:** Yes in clinical research.
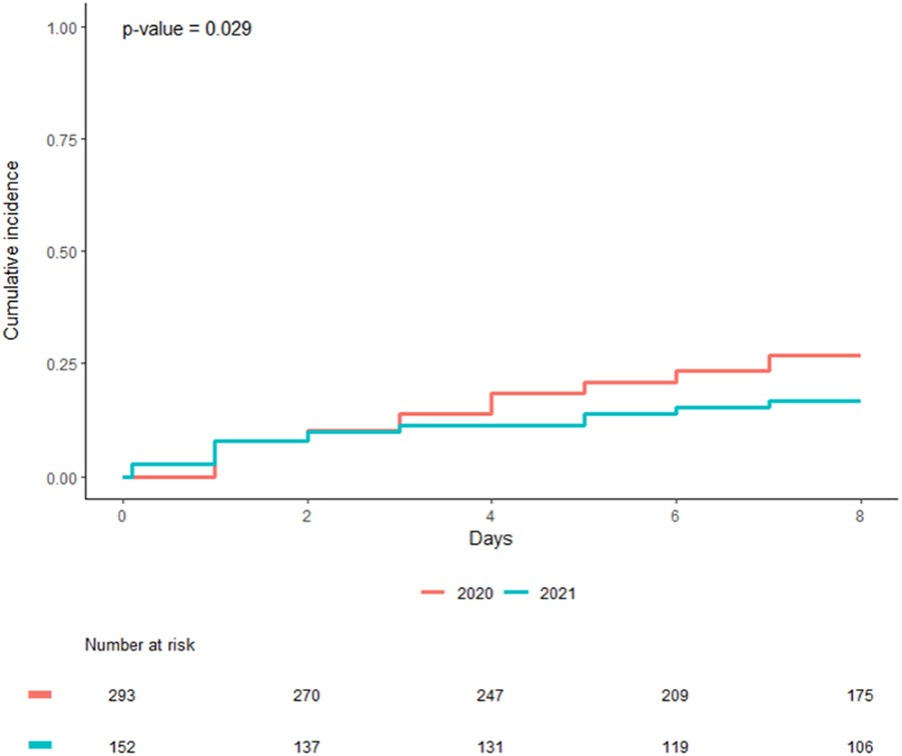


*Cumulative incidence of severe AKI in the first days after ICU admission according to the first and third wave*

### CO-86 Impact of dexamethasone use in severe COVID-19-induced acute kidney injury

#### ORIEUX Arthur^1^, LOUIS Guillaume^3^, GARRIC Antoine^2^, PICARD Yoann^3^, MELLATI Nouchan^3^, PREVEL Renaud^1^, GRUSON Didier^1,4^, RUBIN Sébastien^2,5^, BOYER Alexandre^1,4^

##### ^1^Médecine Intensive Réanimation - CHU de Bordeaux, Bordeaux, France; ^2^Néphrologie, Transplantation, Dialyse - Aphérèse - CHU de Bordeaux, Bordeaux, France; ^3^Réanimation Polyvalente - CHR Metz - Thionville - Hôpital de Mercy, Metz - Thionville, France; ^4^Unité INSERM U1045, Université de Bordeaux, Bordeaux, France., Bordeaux, France; ^5^Unité INSERM U1034, Université de Bordeaux, Bordeaux, France., Bordeaux, France

###### Correspondence: Arthur ORIEUX (arthur.orieux@chu-bordeaux.fr)

*Annals of Intensive Care* 2022, **12(1):**CO-86

**Rationale:** Since the first wave of COVID-19, the management of patients with severe SARS-CoV-2 infection in intensive care unit (ICU) has changed with the use of dexamethasone (DXM)^1^. Acute kidney injury (AKI) in ICU patients with severe COVID-19 was frequent (> 50%). Specific inflammatory process was previously suggested in AKI pathogenesis and could be improved by DXM. A previous study (n = 100 patients) reported a potential protective effect of DXM in AKI incidence^2^. The aim of this study was to investigate in a prospective multicentric study, the impact of DXM in severe COVID-19-induced AKI.

**Patients and methods/Materials and methods:** We carried out a prospective multicentric study in two French ICU from March 1st, 2020 to August 21st, 2021. All patients admitted for a severe COVID-19 in ICU were included. DXM was exclusively used from the second wave. AKI was defined according to the KDIGO classification. Acute kidney disease (AKD) was the persistence of the KDIGO criteria for AKI for ≥ 7 days.

**Results:** 1014 patients were included. 284/1014 patients (28%) were hospitalized in ICU1 and 730/1014 (72%) in ICU2. Mean age was 62.9 ± 12.2 years, 520/1014 (51%) patients had hypertension, 75/1014 (7%) suffered from previous chronic kidney disease (CKD) and 385/1014 (38%) required invasive mechanical ventilation (MV) in the first 24 h. Mean SAPSII was 38.9 ± 15.8 and non-renal SOFA was 4.2 ± 2.4. ICU mortality was 264/1014 (26%). AKI was present in 741/1014 (73%) patients: 266/741 (36%), 173/741 (23%) and 302/741 (41%) had respectively AKI KDIGO 1, 2 and 3 and 88/741 (12%) patients required renal replacement therapy. AKD was observed in 397/741 (54%) of AKI patients. In univariate analysis, DXM exposure decreased AKI incidence: 411/635 (65%) patients vs. 330/379 (87%) patients; OR = 0.27 [0.19–0.38]. In multivariate analysis, DXM use was independently associated with AKI: OR = 0.24 [0.11-0.53] (Table 1). After excluding patients who developed AKI before DXM exposure (n = 186 patients), DXM use remained independently associated with AKI in a similar multivariate model; OR = 0.26 [0.17–0.41]. In univariate analysis, DXM exposure decreased AKD incidence: 207/411 (50%) vs.190/330 (58%) patients; OR = 0.74 [0.55–0.99] but this effect did not persist in a similar multivariate analysis; OR = 1.17 [0.83–1.66].

**Conclusion:** In our study, DXM exposure decrease AKI incidence in severe COVID-19 patients. These results support the hypothesis that DXM can reduce “inflammatory” AKI incidence (specific of COVID-19 infection) but has no impact on “maladaptive repair” lesions secondary to AKI that can lead to an AKD.

**Reference 1:** The RECOVERY Collaborative Group. Dexamethasone in Hospitalized Patients with Covid-19. N. Engl. J. Med. 384, 693–704 (2021).

**Reference 2:** Orieux, A., Khan, P., Prevel, R. et al. Impact of dexamethasone use to prevent from severe COVID-19-induced acute kidney injury. Crit Care 25, 249 (2021).

**Compliance with ethics regulations:** Yes in clinical research.
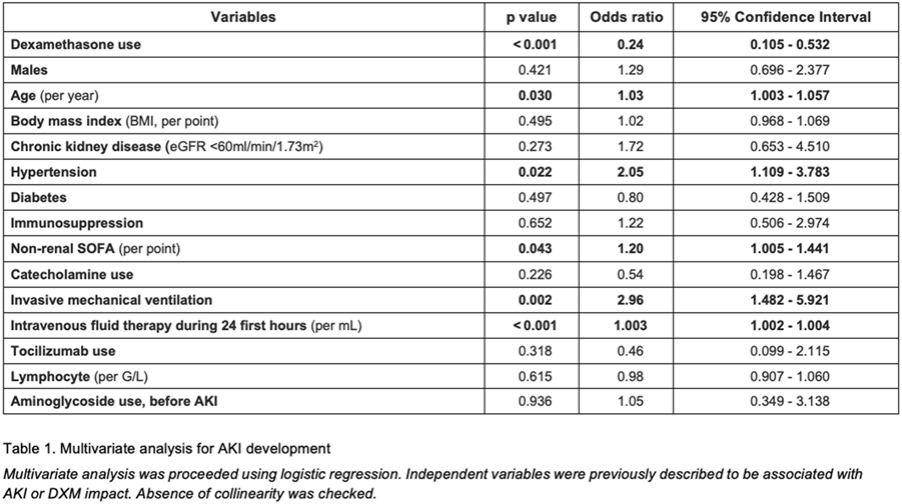


*Table 1. Multivariate analysis for AKI development*

### CO-87 KUNGFU-ECMO Study, kidney upgrades net global filtration under ECMO

#### PENAUD Victor^1^, DUBURCQ Thibault^4^, BUREAU Côme^2^, SALMON-GANDONNIERE Charlotte^5^, ARRESTIER Romain^6^, HENRI Samuel^4^, DRES Martin^2^, JACQUIER Sophie^5^, DE PROST Nicolas^6^, GIRAUD Raphael^3^, RICARD Jean-Damien^1^, ROUX Damien^1^, UHEL Fabrice^1^, LEGOUIS David^3^, VERNEY Charles^1^

##### ^1^AP-HP, Hôpital Louis Mourier, Colombes, France; ^2^AP-HP, Hôpital Pitié Salpêtrière, Paris, France; ^3^Hôpitaux Universitaires de Genève, Genève, Suisse; ^4^Centre Hospitalier Universitaire de Lille, Lille, France; ^5^Centre Hospitalier Régional Universitaire de Tours, Tours, France; ^6^AP-HP, Hôpital Henri Mondor, Créteil, France

###### Correspondence: Charles VERNEY (charlesverney@gmail.com)

*Annals of Intensive Care* 2022, **12(1):**CO-87

**Rationale:** Acute kidney injury (AKI) is frequently observed in acute respiratory distress syndrome (ARDS). Risk factors for AKI include positive end expiratory pressure (PEEP), severity of ARDS and fluid overload, possibly linked to renal congestion. There is no data on the evolution of renal function in patients presenting severe ARDS and requiring extra-corporeal membrane oxygenation (ECMO) implantation. Veino-veinous ECMO (VV-ECMO) could prevent AKI by alleviating this renal congestion with the aspiration of the femoral canula. We aim to study how a VV-ECMO modified the renal function in this population.

**Patients and methods/Materials and methods:** We performed a multicentre retrospective study, between 2011 and 2021, in six intensive care units in France and Switzerland. Inclusion criteria were: adult patients presenting a severe ARDS requiring VV-ECMO, with available data regarding daily urine output and blood tests 2 days before and three days after implantation. Patients receiving renal replacement therapy (RRT) before day three or veino-arterial ECMO or those who did not agree to participate were excluded. The primary outcome was the evolution of the serum creatinine level after VV-ECMO implantation. Daily urine output and evolution of biochemical parameters in urine samples after implantation were the secondary outcomes.

**Results:** Ninety-nine patients were included, with a median age of 53 years, of which 30% were women. ECMO implantation occurred after a median duration of mechanical ventilation of 6 days. COVID-19 was the main cause in 72% of ARDS. The evolution of the serum creatinine level did not significantly differ before and after implantation (p = 0.2). In contrast, ECMO implantation was associated with a significant increase in daily urine output (+ 6.3 mL/kg/day, p < 0.001), even after adjustment for potential confounding factors (including PEEP, duration of mechanical ventilation, fluid balance, cause of ARDS, and diuretics). Fractional excretion of urea and natriuresis were also significantly increased after ECMO implantation (respectively + 10%, p < 0.001 et + 20 mmol/L, p < 0.001). Finally, the increase in urine output under ECMO was negatively associated with the risk to receive RRT after day three (p = 0.047).

**Conclusion:** VV-ECMO implantation for severe ARDS is associated with an increase in daily urine output and natriuresis. This diuretic like effect does not modify the glomerular filtration following implantation. Prospective studies are necessary to better define renal hemodynamic and tubular modifications occurring under VV-ECMO, and the potential improvement in the management of fluid overload in this context.

**Compliance with ethics regulations:** Yes in clinical research.
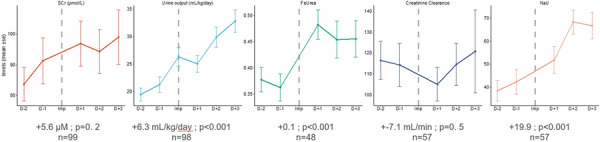


*Evolution of serum creatinine level, daily urine output, Fractional excretion or urea, mesured creatinine clearance with UV/P formula and natriuresis, between 2 days before and 3 days after implantation of VV-ECMO.*

### CO-88 Urinary CCL14 biomarker to predict renal replacement therapy initiation in critically ill patients with severe acute kidney injury?

#### CHAÏBI Khalil^1^, PLACIER Sandrine^2^, LOUIS Guillaume^3^, MARTIN-LEFEVRE Laurent^4^, TITECA Dimitri^5^, LACOMBE Béatrice^6^, BESSET Sébastien^7^, BADIE Julio^8^, CHEVREL Guillaume^9^, CHUDEAU Nicolas^10^, BARBAR Saber^11^, VINSONNEAU Christophe^12^, FOREL Jean-Marie^13^, THEVENIN Didier^14^, LACAVE Guillaume^15^, NSEIR Saad^16^, POIRSON Florent^1^, MAYAUX Julen^17^, KLOUCHE Kada^18^, REIGNIER Jean^20^, QUENOT Jean-Pierre^21^, HADCHOUEL Juliette^2^, DREYFUSS Didier^2^, GAUDRY Stéphane^1^

##### ^1^CHU Avicenne, Bobigny, France; ^2^INSERM CORAKID UMRS1155, Paris, France; ^3^CHR Metz-Thionville Hôpital de Mercy, Metz, France; ^4^CHR départementale La Roche Sur Yon, La Roche Sur Yon, France; ^5^CHU d’Amiens Picardie, Amiens, France; ^6^CH de Bretagne Sud, Lorient, France; ^7^Hôpital Louis Mourier, Colombes, France; ^8^Hôpital Nord Franche-Comte, Belfort, France; ^9^CH Sud Francilien, Corbeil Essones, France; ^10^CH du Mans, Le Mans, France; ^11^Hôpital Caremeau, Nimes, France; ^12^CH Bethune Beuvry – Bermont et Gauthier, Bethune, France; ^13^Hôpital Nord, Marseille, France; ^14^CH Dr Schaffner, Lens, France; ^15^Hôpital André Mignot, Versailles, France; ^16^CHRU de Lille, Hôpital Roger Salengro, Lille, France; ^17^Hôpital Pitié Salpêtrière, Paris, France; ^18^Hôpital Lapeyronnie, Montpellier, France; ^19^Hôpital Avicenne, Bobigny, France; ^20^Hôtel Dieu, Nantes, France; ^21^François Mitterrand University Hospital, Dijon, France

###### Correspondence: Khalil CHAÏBI (khalilchaibi@gmail.com)

*Annals of Intensive Care* 2022, **12(1):**CO-88

**Rationale:** Recent trials confirmed that an early renal replacement therapy (RRT) initiation does not confer any survival benefice compared with a delayed one during severe acute kidney injury (AKI) when no severe complications are present. Tools to enable personalized management are still needed and evidence for biomarkers guided RRT initiation are lacking[1]. In a cohort of critically ill patients with AKI, an elevation in urinary CCL14 could predict the persistence/progression of AKI [2]. We aimed to assess the utility of urinary CCL14 for the prediction of RRT initiation in a conservative approach in Intensive Care Unit (ICU).

**Patients and methods/Materials and methods:** In an ancillary study of AKIKI2 (Comparison of two delayed strategies for renal replacement therapy initiation for severe acute kidney injury), we included ICU patients with severe AKI (stage 3 KDIGO) and available urinary sample at the first day of severe AKI. We did not include patients with chronic kidney disease. CCL14 was measured by enzyme-linked immunosorbent assay (ELISA) in urinary samples at D0. The primary endpoint was the occurrence of a criteria for RRT (according to a conservative approach of RRT initiation) within the 72 h after severe AKI. Those criteria were: life threatening complication of AKI, or oliguria/anuria (urine output < 0·3 mL/kg per h or < 500 mL/day) for more than 72 h, or serum urea concentration of 40 mmol/L or more.

**Results:** A total of 230 patients were enrolled. Ninety patients (39.1%) met the primary endpoint. Urinary CCL14 was not associated with serum creatinine concentration at D0 (Pearson correlation r2 = 0.024). Median urinary CCL14 was 15.03 (IQR 24.88) mg/ml in patients reaching the primary endpoint and 5.8 (IQR 12.18) mg/ml in patients who did not (p < 0.001). In multivariate analysis, urinary CCL14 (OR = 1.02, [1.01;1.04], p = 0.0021) was associated with higher rate of occurrence of a criteria of RRT initiation. The area under the ROC curve was 0.701 (95% CI: [0.633;0.770]) for urinary CCL14 to predict the occurrence of a criteria of RRT initiation within 72 h after severe AKI (Fig. 1).

**Conclusion:** Although interesting, urinary CCL14 alone might not be precise enough to discriminate patients with RRT initiation criteria in a conservative approach. However, it might be useful when used in combination with other clinical or biological variables.

**Reference 1:** Ostermann M, Lumlertgul N. Wait and see for acute dialysis: but for how long? The Lancet. 2021;397:1241–3.

**Reference 2:** Hoste E, Bihorac A, Al-Khafaji A, Ortega LM, Ostermann M, Haase M, et al. Identification and validation of biomarkers of persistent acute kidney injury: the RUBY study. Intensive Care Med. 2020;46:943–53.

**Compliance with ethics regulations:** Yes in clinical research.
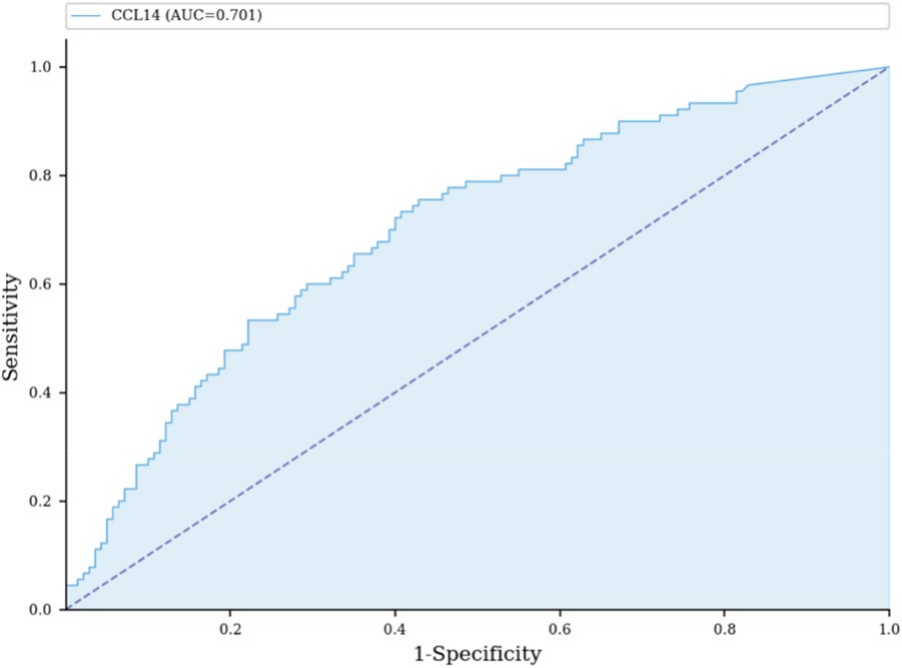


*Receiver operating characteristic (ROC) curve for prediction of RRT initiation criteria with urinary CCL14*

### CO-89 Concurrent treatment with angiotensin 1-7 in septic shock

#### GARCIA Bruno^1^, ANNONI Filippo^1^, SU Fuhong^1^, HERPAIN Antoine^1^, TACCONE Fabio^1^, CRETEUR Jacques^1^

##### ^1^Laboratoire experimental des soins intensifs de l'hôpital Erasme, Bruxelles, Belgique

###### Correspondence: Bruno GARCIA (br.garcia@icloud.com)

*Annals of Intensive Care* 2022, **12(1):**CO-89

**Rationale:** Renin-angiotensin system is activated during sepsis and septic shock. Its main effector, angiotensin II, is responsible for pro-inflammatory effects and endothelial dysfunction. Angiotensin (1–7) (Ang (1–7)), an active peptide produced by angiotensin-converting enzyme type 2 (ACE2), has opposite effects and has been reported to prevent organ damage during sepsis in mice. Our study aimed to assess the effect of concurrent treatment with ang (1–7) on sepsis outcome.

**Patients and methods/Materials and methods:** Sepsis was induced by fecal peritonitis in sixteen mechanically ventilated, hemodynamically monitored female sheep. Animals were randomized to two groups: control or concurrent treatment group (n = 8 each) after surgical preparation and stabilization. Angiotensin (1–7) at 10 mcg/kg/h was started at sepsis inducement in the concurrent treatment group. Four hours after feces injection, fluid resuscitation (pulse pressure variation ≤ 13%), antibiotherapy, and peritoneal lavage were administrated, and norepinephrine was given to maintain mean arterial blood pressure ≥ 65 mmHg if necessary. The experiment lasted for 24 h.

**Results:** No difference was found among groups regarding baseline demography. During the first 4 h, MAP was dropped significantly in the control group. All the animals in the control group developed a septic shock. A significantly lower dose of norepinephrine was utilized in the concurrent treatment group. Creatinine, platelet count, PaO_2_/FiO_2_, pH, and lactate levels were significantly lower in the concurrent treatment group. Two animals died in the control group before the end of the experiment.

**Discussion:** In this mechanical ventilated, hemodynamically monitored sepsis model; concurrent treatment with ang (1–7) significantly reduced norepinephrine requirements and attenuated organ dysfunction. Ang (1–7) can reduce inflammation by the activation of the Mas receptor, and reduce cytokine storm. However, the risk of the effects on the immune system is not well described in the literature and could expose to secondary infections. This is the first use of Ang (1–7) in a large clinically relevant animal model characterized by fluid resuscitation based on dynamic parameters, broad-spectrum antibiotherapy, peritoneal lavage, and vasopressors. Polymicrobial sepsis, induced by feces injection, was able to develop a severe septic shock with organ dysfunction. The protocol followed the recent guidelines on septic shock resuscitation and guidelines for Minimum Quality Threshold in Pre-Clinical Sepsis Studies (MQTiPSS).

**Conclusion:** Concurrent treatment with Ang (1–7) reduced severity of septic shock. Further studies are needed to assess the timing of administration, dose and duration of Ang (1–7) in sepsis.

**Compliance with ethics regulations:** Yes in animal testing.
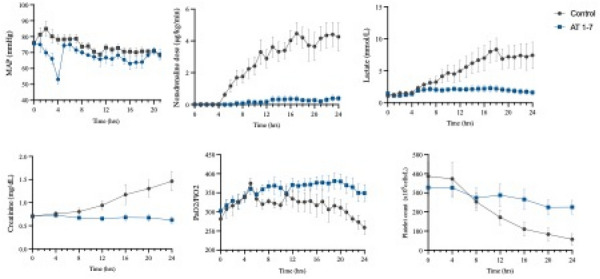


*Results*

### CO-90 Renal prognosis of elderly TTP patients on caplacizumab therapy (the Repeto-capla study)

#### CARNIATO Flavie^1^, LESCROART Mickael^1^, ZAFRANI Lara^1^, AZOULAY Elie^1^, VEYRADIER Agnès^2^, COPPO Paul^3^, MARIOTTE Eric^1^

##### ^1^Hôpital Saint-Louis, Paris 10E, France; ^2^Hôpital Lariboisière, Paris 10E, France; ^3^Hôpital Saint-Antoine, Paris 12E, France

###### Correspondence: Flavie CARNIATO (flavie.carniato@gmail.com)

*Annals of Intensive Care* 2022, **12(1):**CO-90

**Rationale:** Thrombotic thrombocytopenic purpura (TTP) is a thrombotic microangiopathy caused by a severe ADAMTS13 activity deficiency. Mortality in TTP sharply decreased with the introduction of emergency therapeutic plasma exchanges (TPE) and immunomodulation; however, elderly patients remain at high risk of mortality and severe organ involvements, including acute renal failure. More recently, the adjunction of Caplacizumab has improved time to recovery in TTP patients. The association of caplacizumab administration with improved renal function in elderly patients with TTP is unknown.

**Patients and methods/Materials and methods:** In this retrospective study, we described the characteristics and outcome of TTP patients aged over 60 years. They were enrolled from 2000 to 2021 in the French national reference center for thrombotic microangiopathy registry according to treatment with standard of care (SOC, consisting of TPE, steroids and for most rituximab) with or without Caplacizumab (C+ and C− groups). Results are presented as number (%) or median (interquartile range). Comparisons were made using Chi^2^ or Mann–Whitney tests as appropriate.

**Results:** Sixty-three patients met inclusion criteria, including 35 females (55%) aged 70 yo (65–75). Thirty-four (54%) patients received C− and 28 C+. Patients’ medical history and baseline characteristics were similar between both groups, including frequency of chronic kidney disease (14%), neurological involvement (79%), platelet rate (16G/L(9-24)), hemoglobin level (8.9 g/dL(8–10)), elevated troponin (68%) and Glomerular Filtration Rate (GFR, 51 ml/min/1.73 m^2^(35–77)). All C+ patients received Rituximab versus 65% of C− (p < 0.05). Caplacizumab was administered 2 (1.5–7.5) days after admission, for 30 (21–37) days, with frequent adverse effects (53%). Time to platelet count normalization was shorter with C+ (4 days vs 8, p < 0.05). C+ patients needed less TPE (5(4-8) sessions vs 11(6–16), p < 0.05) and experienced no case of refractory TTP. All patients requiring renal replacement therapy (RRT) belonged to the C− group (18% vs 0%, p = 0.056). For others, GFR was similar at discharge (C+ 78(59–94) mL/min/1.73 m^2^ vs C− 62(42–86) mL/min/1.73 m^2^, p = 0.11). Hospital mortality was higher in the C− group than in the C+ group (38% vs 3.5%, p < 0.05). After a follow up of 165 (24–630) days, 1/3 of patients requiring RRT during initial hospitalization were RRT-free. For others, GFR was similar (C+ 78(64–90) mL/min/1.73 m^2^ vs C− 62(42–80) mL/min/1.73 m^2^, p = 0.24).

**Conclusion:** Our results should be influenced by the fact that C− patients were managed during the earlier period of the study and received different SOC, including a lower frequency of rituximab use. In this study, Caplacizumab seemed to improve outcome in elderly TTP patients with no clear effect on renal dysfunction.

**Compliance with ethics regulations:** Yes in clinical research.

## Flash communications

### FC-001 Epidemiological profile of burns in children admitted to the casablanca national burns center

#### CHAKIR Anass^1^, MOKAKO Jacques^2^, HABLA Marouane^2^, ELHARTI Anas^2^, DIOURI Mounia^2^

##### ^1^Département d'anesthésie et de réanimation, Centre hospitalier universitaire Ibn Rochd, Casablanca, Maroc; ^2^Centre national des brûlés et de chirurgie réparatrice, Centre Hospitalier Universitaire Ibn Rochd, Casablanca, Maroc

###### Correspondence: Youssef HAOUAS (h.youssef414@gmail.com)

*Annals of Intensive Care* 2022, **12(1):**FC-001

**Rationale:** This retrospective work of the last 3 years (January 2018–March 2021) analyzes the epidemiological characteristics of 1156 cases of burnt children aged 0–17 years admitted to the National Burn Center emergency room so that these data serve as a basis for the development of a program of prevention, adequate, fast and effective treatment of burnt children. The age group from 1 month to 2 years was the most affected with 587 cases or 50.77% of patients.

**Patients and methods/Materials and methods:** Male involvement is found in 54% of cases, i.e. 624 patients. Burns occur at home in 99.48% of cases, i.e. 1,150 patients, and accidentally in 99.39% of cases, i.e. 1,149 patients.

**Results:** Thermal burns accounted for 98.78% of cases or 1,142 patients, dominated by liquids in 77.94% of cases or 901 patients. The burnt skin surface was ≥ 10% in 44% of cases, i.e. 509 patients. The burn mainly concerns upper limbs 57% of cases or 659 patients. The mortality rate was 7.61% or 39 patients.

**Conclusion:** This work also includes a retrospective comparison of the COVID period (March 2020–March 2021) to the pre-COVID period (March 2019–February 2020) during which the number of emergencies had increased by 24 additional patients (370 versus 346) with hospitalization an increase of 3.14% (171 versus 149) and during which 1 child had tested positive for COVID 19. During this comparative period the mortality rate was down by 1.77% (5 versus 7).

**Compliance with ethics regulations:** Yes in clinical research.

### FC-002 Changes in management practices for moderate to severe bronchiolitis in infants between 2010 and 2020: a retrospective study conducted in the pediatric intensive care unit

#### LESUEUR Simon^1^, SAVY Nadia^1^

##### ^1^CHU Clermont Ferrand, Clermont Ferrand, France

###### Correspondence: Simon LESUEUR (simon.lesueur31@gmail.com)

*Annals of Intensive Care* 2022, **12(1):**FC-002

**Rationale:** Acute bronchiolitis represents 8 to 13% of respiratory distress hospitalised in Pediatric Intensive Care Units (PICUs). The main challenge of care is the early management of respiratory failure and digestive intolerance. In the PICU, our practice has been to use the High Flow Nasal Cannula (HFNC) almost exclusively, due to its ease of use and good tolerance, despite the fact that the latest trials do not demonstrate its non-inferiority compared to CPAP. With this study, we would like to highlight an improvement in the quality of care and well-being of our patients with moderate to severe bronchiolitis.

**Patients and methods/Materials and methods:** This is a retrospective descriptive study conducted over two periods P1 (2010–2013) and P2 (2017–2020) in the PICU on infants with moderate to severe acute bronchiolitis. The main objective of our study is to evaluate the improvement of the quality of care over time through a composite criterion: the use of antibiotic therapy and the time to complete enteral feeding, by comparing them over the two distinct periods. The secondary endpoints were comparisons of clinical and therapeutic data.

**Results:** Our analysis involved 106 patients, 45 in the first period P1 and 61 in the second period P2, matched for severity and age. Antibiotic use decreased over the decade (66.7% vs. 37.7%, p < 0.05) and completion of full enteral feeding tended to be shorter (3.1 (± 1.5) days vs. 2.6 (1.2) days respectively; p = 0.08). The use of HFNC as first-line treatment increased significantly over the decade (33.3% vs 96.7%; p < 0.05) at the expense of CPAP, which is no longer used on P2. More aggressive care tended to decrease with lower rates of intubation (1.6% vs 8.9%; p = 0.16) and less intravenous glucose solution infusion (60.7% vs 91.1%; p < 0.05). Complication rates were low and similar in both periods. However, the total duration of ventilation was shorter on P1 with 4.5 (± 3.0) days compared to 5.5 (± 2.7) days on P2; p = 0.07.

**Conclusion:** The quality of care in our unit has improved over the last decade with greater adherence to international guidelines regarding decreased antibiotic use and preference for enteral intake over infusion.

**Compliance with ethics regulations:** Yes in clinical research.

### FC-003 Impact of the Nusinersen on the number of hospitalisations in pediatric intensive care units for respiratory decompensation in children with spinal muscular atrophy over the period 2015–2021

#### BAKAYOKO Awa^1^, VAUGIER Isabelle^1^, MBIELEU Blaise^1^, ESSID Aben^1^, ZINI Justine^1^, BELTAIEF Emna^1^, GUILLON Maud^1^, FAYSSOIL Abdallah^1^, GOMEZ GARCIA DE LA BANDA Marta^1^, QUIJANO ROY Susana^1^, BERGOUNIOUX Jean^1^

##### ^1^ Hôpital Raymond-Poincaré, Garches, France

###### Correspondence: Awa BAKAYOKO (awa.bakayoko@aphp.fr)

*Annals of Intensive Care* 2022, **12(1):**FC-003

**Rationale:** Spinal muscular atrophy is a neuromuscular disease of genetic origin. It is the leading genetic cause of infant mortality. Worldwide, the prevalence of this disease is estimated at 1 in 10,000 births. The genes involved are the SMN1 and SMN2 genes, located on chromosome 5, whose deletion leads to the destruction of motor neurons, which results in an impairment of respiratory function. Nusinersen is the first curative drug developed for the treatment of spinal muscular atrophy. It is an antisense oligonucleotide that binds to the pre-messenger RNA of the SMN2 gene, allowing the production of the SMN protein, and thus leading to improved motor control. The aim of this study was to evaluate the impact of Nusinersen on the admission to the pediatric intensive care unit in our hospital, for respiratory decompensation in children with spinal muscular atrophy.

**Patients and methods/Materials and methods:** Retrospective comparative study of the reasons for admission to a pediatric intensive care unit in children with spinal muscular atrophy, treated or not by Nusinersen, over the period 2015–2021.

**Results:** 69 patients with spinal muscular atrophy were hospitalised in our Intensive Care Unit between 2015–2021; 108 passages were related to respiratory decompensation, representing 41 patients. There has been a significant decrease in hospital admissions for respiratory decompensation in SMA over the last 4 years, which we were able to statistically correlate with the implementation of Nusinersen treatment (results under analysis).

**Conclusion:** We observed a significant decrease in hospitalisations for respiratory decompensation in the intensive care of unit of patients with AMSI and this correlated with the administration of Nusinersen.

**Compliance with ethics regulations:** N/A.

### FC-004 Work of breathing during non invasive ventilation in severe acute bronchiolitis

#### VEDRENNE-CLOQUET Meryl^1^, KHIRANI Sonia^1^, GRIFFON Lucie^1^, COLLIGNON Charlotte^1^, RENOLLEAU Sylvain^1^, FAUROUX Brigitte^1^

##### ^1^Necker-Enfants Malades, Paris, France

###### Correspondence: Meryl VEDRENNE-CLOQUET (meryl.vedrenne@aphp.fr)

*Annals of Intensive Care* 2022, **12(1):**FC-004

**Rationale:** Continuous positive airway pressure (CPAP) has been shown to reduce the work of breathing (WOB) during severe acute bronchiolitis. Noninvasive Positive Pressure Ventilation (NIPPV) is also used in this condition but no study has compared NIPPV to CPAP. We aimed to compare WOB during CPAP and NIPPV in infants with acute bronchiolitis.

**Patients and methods/Materials and methods:** Infants < 6 months of age with bronchiolitis were included if they needed noninvasive respiratory support (NRS) within the first 24 h. Exclusion criteria included contra-indication for nasogastric tube, imminent intubation, neuromuscular disease, parental refusal. NRS was initiated by the attending physician according to local practice: CPAP + 7 cmH_2_O was the first-line setting, with NIPPV, set on clinical parameters and child’s comfort, being reserved for CPAP failure. Oesophageal (P_ES_) and gastric pressures were measured using a nasogastric catheter (Gaeltec™). The study started with a first period with the baseline NRS (CPAP or NIPPV), followed by a 5-min washout (spontaneous breathing). Afterward, a second period was performed with the other NRS. Within each period, a first recording was performed with the clinical settings (Clin) and a second with a physiological setting (Phys) aiming at normalizing WOB. For CPAPPhys, the CPAP level was increased from 6 to 10 cmH_2_O. For NIPPVPhys, initial inspiratory pressure was set at + 4 cmH_2_O above CPAP, and progressively increased, with inspiratory and expiratory triggers set to optimize synchronization. The “optimal” NRS was the one associated with an optimal reduction in clinical symptoms, P_TC_CO_2_ (Sentec™), and estimated WOB (PTP_ES_/min = AUC of P_ES_ during inspiration × Respiratory Rate) over 10 stable breaths.

**Results:** 20 children were included, with a median [IQR] age of 1.4[0.8; 2.9] months and a median weight of 4[3.5; 5.2] kilograms. Twelve infants had CPAP at baseline: CPAP remained the optimal mode in 7, whereas NIPPV was superior in 5. Eight infants had NIPPV at baseline: NIPPV remained the optimal mode in 2, whereas CPAP was superior in 6 (Figure).

**Conclusion:** CPAP was associated with a decrease in WOB in the majority of infants with acute bronchiolitis. NIPPV may be superior to CPAP in some infants. Predictive factors associated with a need for NIPPV were not identified in the present study.

**Compliance with ethics regulations:** Yes in clinical research.
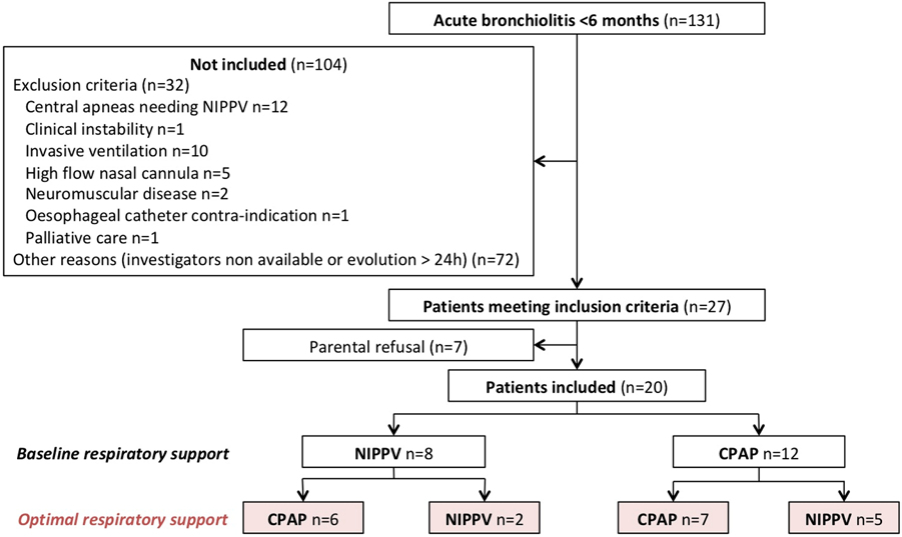


*Study flow chart*

### FC-005 Continuous determination of optimal mean arterial pressures based on cerebral autoregulation after pediatric cardiac surgery

#### TABONE Laurence^1^, EL-TANNOURY Jihad^1^, LEVY Michaël^3^, SAUTHIER Michael^1^, JORAM Nicolas^2^, BOURGOUIN Pierre^2^, AL-OMAR Sally^1^, EMERIAUD Guillaume^1^, THIBAULT Céline^1^

##### ^1^CHU Sainte Justine, Montréal, Canada; ^2^CHU de Nantes, Nantes, France; ^3^CHU Robert Debré, Paris, France

###### Correspondence: Laurence TABONE (lau.tabone@gmail.com)

*Annals of Intensive Care* 2022, **12(1):**FC-005

**Rationale:** Neuroprotection is a major concern in children with congenital heart diseases. Cerebrovascular autoregulation (CAR) is the capacity of cerebral vessels to ensure a constant blood flow in a given range of mean arterial pressures (MAP) and may be a modifiable perioperative factor influencing outcomes. CAR has been shown to be altered before and during cardiopulmonary bypass, but has not yet been described in the postoperative period. The cerebral oximetry index (COx) estimates CAR by correlating regional cerebral oxygenation and MAP. By plotting COx against MAP, the optimal MAP (opt-MAP) and the lower and upper limits of MAP corresponding to optimal CAR (LLA and ULA) can be determined. Our aim was to evaluate the feasibility of continuously determining the opt-MAP, LLA, and ULA in children within 48 h of cardiac surgery and to describe CAR parameters according to age and postoperative time frame.

**Patients and methods/Materials and methods:** Using a high-frequency database, we retrospectively included all children admitted in a single pediatric intensive care unit (PICU) after cardiac surgery between May 2020 and June 2021. An algorithm for the continuous calculation of the opt-MAP, ULA, and LLA was developed using a weighted combination of 40 time windows for the calculation of COx and opt-MAP. CAR results were described according to four predefined age groups and postoperative time frames. We calculated the percentage of time with MAP outside the range of LLA to ULA. Results are reported as median [interquartiles] and comparisons between age groups and postoperative time periods were performed using using Wilcoxon and Kruskal-Wallis tests.

**Results:** Fifty-one children were included with a median monitoring time of 42 [23–44] hours. Opt-MAP, LLA, and ULA were determined 92% [88–95] of the time, with first results available 74.0 [IQR 60.0–105.0] minutes after PICU admission. Lower opt-MAP were observed in neonates < 1 month old (Table). Children spent 25% [18–31] of their time with MAP outside the optimal CAR range, with no significant difference between age groups or periods.

**Conclusion:** The continuous calculation of Opt-MAP, LLA and ULA is feasible in children after cardiac surgery using signals routinely available in clinical practice. Children spent a significant proportion of time outside the optimal range of MAP based on CAR. Further research is warranted to explore the potential impacts of this deviation on neurological outcomes.

**Compliance with ethics regulations:** Yes in clinical research.
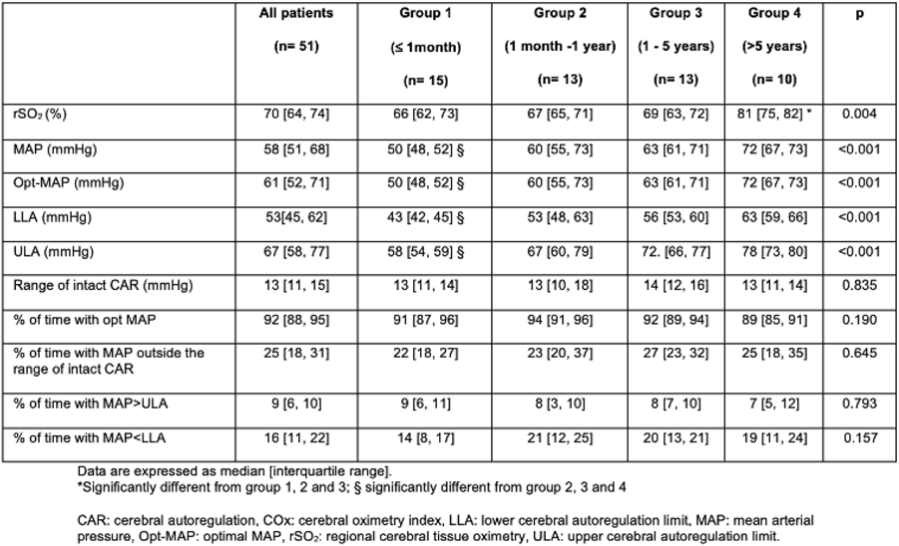


*Cerebral autoregulation parameters according to age groups*

### FC-006 Optical thermography infrastructure to assess thermal distribution in critically ill children

#### SHCHERBAKOVA Monisha^1,2^, NOUMEIR Rita^2^, LEVY Michael^1^, BRIDIER Armelle^1^, LESTRADE Victor^1^, JOUVET Philippe^1^

##### ^1^CHU Sainte Justine, Montreal, Canada; ^2^Ecole de technologie supérieure, Montreal, Canada

###### Correspondence: Monisha SHCHERBAKOVA (monisha.shcherbakova.hsj@ssss.gouv.qc.ca)

*Annals of Intensive Care* 2022, **12(1):**FC-006

**Rationale:** The body temperature distribution at the skin surface could be used to monitor changes in cardiac output [1]. The aim of this study was to explore infrared thermography (IRT) as a non-invasive and reliable method to analyze core body temperature of critically ill children and assess the clinical value of temperature evolution across different parts of the body.

**Patients and methods/Materials and methods:** Patients admitted to the pediatric intensive care unit (PICU) were included in this study after parental consent was obtained (approved by the CHU Ste-Justine Research Ethics Board). An infrared sensor (Lepton 3.0 & 3.5 Thermal Camera, Teledyne FLIR, Canada) was used to take infrared (IR) images in clinical conditions. The infrared core and limb temperatures (θ_c_ & θ_l_) were extracted. A line was drawn on the IR images starting from the center (eyes/internal canthus or thorax) and running along the limbs to the extremities. The temperature along this line was extracted and plotted on a graph, to study how the temperature modulates across the body. The gradient was calculated by taking the difference between the core (θ_c_) and limb (θ_l_) temperatures extracted from the images. The correlation between values was assessed by Spearman's correlation coefficient.

**Results:** In total, 36 patients were included. Their median age was 8 months [1–64.5], 7.4 kg [4.5–24.6]. The median [interquartile range] for θ_c_ extracted from the images was 33.9 °C [32.7–34.2 °C] and the median θ_l_ was 30.2 °C [28.9–33.1 °C]. There was a good correlation between the θ_c_ and the clinical axillary temperature (rho = 0.39 and p-value = 0.016). The median thermal gradient was 3.2 °C [1.1-4.6] and the maximum gradient was 6.0 °C. The temperature gradient across the body was subject to many artefacts (Fig. 1). Due to the low number of patients with hemodynamic instability, we could not study the correlation between thermal gradient and clinical status. Surprisingly, we observed a child with a negative thermal gradient (− 1.14 °C) i.e. higher temperature on the extremities (fingers) compared to the thorax. This child had a cardiac surgery and was treated with a vasodilator (milrinone).

**Conclusion:** The study documented that the thermal gradient was easily manually calculated. The automatic thermal gradients calculation needs further work to have an adequate segmentation and to identify artefacts. Further work is also needed to document the added value of monitoring changes in the thermal gradient over time to assist in the management of critically ill children with hemodynamic instability.

**Reference 1:** [1] Ferraris A, Bouisse C, Mottard N, Thiollière F, Anselin S, Piriou V, et al. Mottling score and skin temperature in septic shock: Relation and impact on prognosis in ICU. PLoS One. 2018;13(8):e0202329.

**Compliance with ethics regulations:** Yes in clinical research.
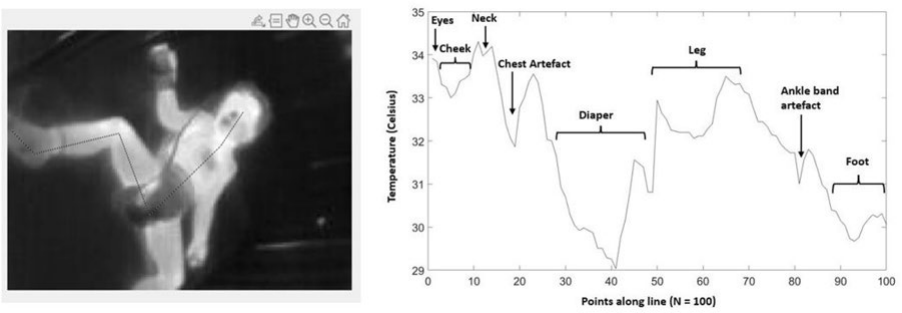


*IR image taken as part of the study, with a line joining the core (eyes) down to the extremities of the leg. The adjoining graph depicts the temperature as it changes along this line.*

### FC-007 Trend over three decades of the stated practice pattern on the hemoglobin (Hb) threshold that would guide red blood cell (RBC) transfusion practice in pediatric intensive care units, on behalf of the CCCTG, PCCS, JICRG and GFRUP

#### GALLAND Anne^1^, TUCCI Marisa^1^, LETEURTRE Stéphane^2^, SARFATTI Avishay^3^, RAY Samiran^4^, STANWORTH Simon^5^, FONTELA Patricia^6^, KAWAGUCHI Atsushi^7^, DEMARET Pierre^8^, DUCRUET Thierre^9^, LACROIX Jacques^1^, DU PONT-THIBODEAU Geneviève^1^

##### ^1^Division of Pediatric Critical Care Medicine, Department of Pediatrics, CHU Sainte-Justine, Université de Montréal, Montreal, Canada; ^2^Réanimation et Surveillance Continue Pédiatriques, Hôpital Jeanne de Flandre, CHRU Lille, Lille, France; ^3^Department of Pediatrics, Oxford University Hospitals NHS Foundation Trust, Oxford, Royaume-Uni; ^4^Department of Pediatrics, Great Ormond Street Hospital, London, Royaume-Uni; ^5^Transfusion Medicine, Department: Haematology, NHS Blood & Transplant/Oxford Radcliffe Hospitals, Oxford, Royaume-Uni; ^6^Pediatric Critical Care, Departments of Pediatrics and of Epidemiology, Biostatistics, and Occupational Health, The Montreal Children's Hospital, McGill University, Montreal, Canada; ^7^Pediatric Intensive Care Unit, Tokyo Women's Medical University, Department of Intensive Care Medicine, Tokyo, Japon; ^8^Pediatric intensive care unit, Department of Pediatrics, CHC Liège, Liège, Belgique; ^9^Unité de recherches cliniques appliquées, Research Centre, CHU Sainte-Justine, Montréal, Canada

###### Correspondence: Jacques LACROIX (jlacroix052@gmail.com)

*Annals of Intensive Care* 2022, **12(1):**FC-007

**Rationale:** To determine the trend over three decades of the stated practice pattern of pediatric intensivist on the Hb threshold that guides their RBC transfusion practice.

**Patients and methods/Materials and methods:** Three scenario-based self-administered questionnaires were filled by 163, 125 and 132 respondents in 19971, 20102 and 2021, respectively. Three scenarios were similar in these surveys: 1) previously healthy 4-year-old boy mechanically-ventilated after a severe multiple trauma; 2) idem with septic shock; 3) 5-month-old boy admitted to a Pediatric Intensive Care Unit (PICU) following corrective surgery for tetralogy of Fallot. Patients were hemodynamically stable in all scenarios. The question asked after each scenario was: what Hb concentration would prompt you to prescribe an RBC transfusion to this patient?

**Results:** Figure 1 reports the practice pattern stated in 1997, 2010 and 2021. For all scenarios, all respondents (100%) should have chosen to prescribe an RBC transfusion only if the Hb level was

**Conclusion:** The TRIPICU study, a non-inferiority randomized controlled trial (RCT) led by Lacroix et al. (N Engl J Med 2007;356:1609–19), enrolled 637 hemodynamically stable critically ill children who had Hb concentration ≤ 9.5 g/dL; it showed that a Hb threshold of 7 g/dL for RBC transfusion is not inferior to a threshold of 9.5 g/dL (absolute risk reduction with the restrictive strategy, 0.4%; 95% confidence interval, − 4.6 to 5.4) and that it can decrease transfusion requirements in PICU patients without increasing adverse outcomes. The main recommendation of TRIPICU was not giving an RBC transfusion to hemodynamically stable PICU patients if their Hb concentration is ≥ 7 g/dL. The RCT led by Cholette et al. (Ann Thorac Surg 2017;103:206–14) that enrolled 134 cardiac children weighing ≤ 10 kg undergoing biventricular repair cardiac surgery and the RCT led by Holst et al. (N Engl J Med 2014;371:1381–91) that enrolled 1005 adults in septic shock with an Hb concentration ≤ 9 g/dL recommended the same Hb threshold (7 g/dL). The gaps in 2021 between the expected proportion of respondents who should have chosen a Hb threshold of 7.0 g/dL (100%) and the proportion who chose a higher Hb threshold remained important in 2021.

**Reference 1:** Laverdière C, Gauvin F, Hébert PC, Infante-Rivard C, Hume H, Toledano BJ, et al. Survey of transfusion practices in pediatric intensive care units. Pediatr Crit Care Med 2002;3:335–40.

**Reference 2:** Du Pont-Thibodeau G, Tucci M, Ducruet T, Lacroix J. Survey on stated transfusion practices in PICU. Pediatr Crit Care Med 2014;15:409–16.

**Compliance with ethics regulations:** Yes in clinical research.
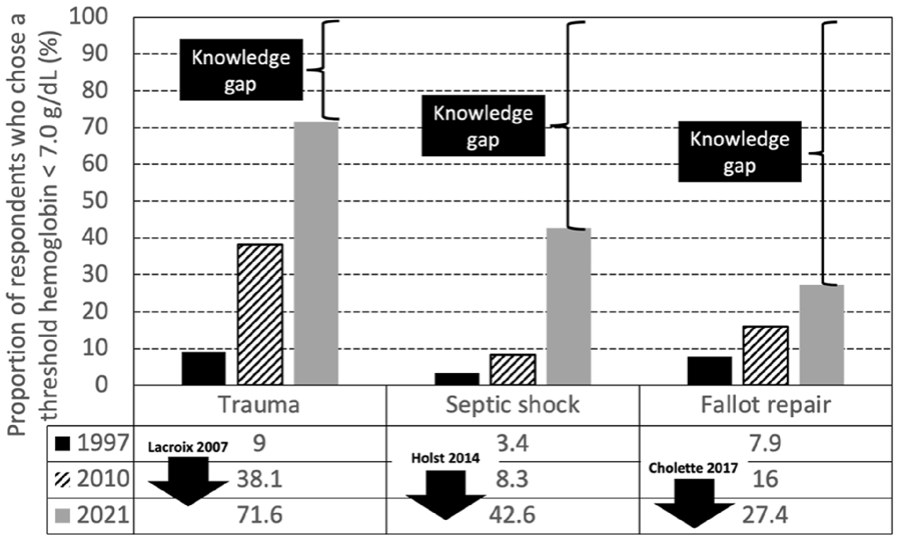


*Trajectory of the stated practice pattern of pediatric intensivists on the Hb concentration that would prompt them to prescribe an RBC transfusion in cases of pediatric trauma, septic shock and post Fallot repair, taking into account 3 RCTs (arrows).*

### FC-008 Visiting policies and parental presence in care: a survey in French Pediatric Intensive Care Units

#### MANON Bergerat^1^, DE SAINT BLANQUAT Laure^1^, BERANGER Agathe^1^

##### ^1^Necker enfants malades, Paris, France

###### Correspondence: Agathe BERANGER (agathe.beranger@gmail.com)

*Annals of Intensive Care* 2022, **12(1):**FC-008

**Rationale:** The European charter of the hospitalized child recommends a 24-h presence of parents at the bedside. Despite major changes in the last decade, family presence in Pediatric Intensive Care Units (PICUs) still remain controversial within and across countries. The objective of this study was to describe the visiting policies and the parental presence during care in French PICUs.

**Patients and methods/Materials and methods:** A structured questionnaire was emailed to the 35 French PICUs. Data regarding visiting policies, involvement in care, evolution of policies and general characteristics were collected from April 2021 to May 2021. Descriptive analysis were conducted.

**Results:** Response rate was 80% with 28 PICUs included. A 24-h access for parents was available for all PICUs (n = 28) with many facilities. Other authorized visitors were principally grandparents (n = 20, 71%) and siblings (n = 18, 64%) with a professional support. Number of visitors at the same time was mainly restricted to two visitors in 82% (n = 23) PICUs. Family presence was always permitted during medical rounds for 19 (68%) PICUs and sometimes authorized during cardiopulmonary resuscitation for 8 (29%) PICUs. Most of the units never included parents during invasive procedures.

**Conclusion:** An unrestricted access at any time for both parents was found. Restriction regarding type and number of persons at bedside were still applied. Parental presence during care were heterogenous and still restricted. National guidelines and educational programs are needed to support family wishes and promote acceptance by healthcare providers.

**Compliance with ethics regulations:** N/A.

### FC-009 Impact of hyperglycemia on critically ill COVID-19 patient outcomes

#### ALILA Ilef^1^, KHARRAT Sana^1^, HADDED Amina^1^, JERBI Salma^1^, BACCOUCH Najeh^1^, BAHLOUL Mabrouk^1^, BOUAZIZ Mounir^1^

##### ^1^hopital Habib Bourguiba Sfax, Sfax, Tunisie

###### Correspondence: Ilef ALILA (ilefalila1323@gmail.com)

*Annals of Intensive Care* 2022, **12(1):**FC-009

**Rationale:** Diabetes is a known risk factor for mortality in Coronavirus disease 19 (COVID-19) patient. Our objective was to identify prevalence of hyperglycemia in COVID-19 patients and its effect on patient outcomes.

**Patients and methods/Materials and methods:** We conducted a retrospective study including critically ill patients with confirmed SARS-COV2 infection in an intensive care unit in between September 2020 and December 2021.

**Results:** During the study period, 586 patients were included with a mean age of 59.5 ± 14.7 years and a sex ratio of 1.6. The median SAPSII and SOFA score were respectively of 29 ± 14.8 and 4 ± 2.6. Most patients had comorbidities, including hypertension (36%), obesity (32.1%) and diabetes mellitus (36.2%). The mean rate of serum glucose on admission was 12.5 ± 6.5 mmol/l. Hyperglycemia occurred in 225 (38.4%) patients; 66 of these hyperglycemic patients (29.3%) had no prior history of diabetes. The mean PH and the mean HCO3- at admission were significantly lower in patients with hyperglycemia (7.37 ± 0.1 vs. 7.41 ± 0.08; p < 0.001 and 22.7 ± 5.6 vs. 23.8 ± 4.7; p = 0.014 respectively). The use of invasive mechanical ventilation was significantly higher in patient with hyperglycemia (p = 0.002). The rate of bacterial infections and the length of stay were not significantly different between patients developing hyperglycemia or not. Mortality was significantly higher in patients developing hyperglycemia during hospitalization (58.2% versus 43% p < 0.001; OR = 1.8[1.3–2.5]).

**Conclusion:** Hyperglycemia without prior diabetes was common and was associated with an increased use of invasive mechanical ventilation and a higher risk of death.

**Compliance with ethics regulations:** Yes in clinical research.

### FC-010 Cortisol serum level prediction of outcome in critically ill COVID-19 patients

#### HADDED Amina^1^, ALILA Ilef^1^, BEN KHALIFA Atraa^1^, KHARRAT Sana^1^, BACCOUCH Najeh^1^, BAHLOUL Mabrouk^1^, BOUAZIZ Mounir^1^

##### ^1^hopital Habib Bourguiba Sfax, Sfax, Tunisie

###### Correspondence: Ilef ALILA (ilefalila1323@gmail.com)

*Annals of Intensive Care* 2022, **12(1):**FC-010

**Rationale:** The COVID-19 epidemic has been a health threat worldwide causing multi-organ damage. However, the endocrine system and especially cortisol level has been less studied in this pathology. The aim of this study was to explore the role of cortisolaemia in the prediction of outcome in critically ill COVID-19 patients.

**Patients and methods/Materials and methods:** We conducted a retrospective study in a medical ICU over a period of 16 months [September 2020–December 2021] including patients with SARS-COV2 infection. Cortisolaemia were measured during the first 24 h of hospitalization.

**Results:** During study period, 132 patients were included with mean age of 62.5 ± 12.7 years, gender ratio of 1.9. The median SAPS II score was 34 ± 14.3 and the median SOFA score was 4 ± 2.4. Most patients had comorbidities, including hypertension (45.5%), obesity (34.8%) and diabetes (37%). Median cortisol level was 71.2 ng/ml with extremes between 5.2 and 643 ng/ml. Severe acute respiratory distress syndrome (ARDS) was diagnosed in 103 (95%) patients, invasive mechanical ventilation was required in 65 (49.2%) of cases and nosocomial infections were diagnosed in 58 (44%). Median ICU length of stay was 7 days ± 6.3 and overall mortality was of 49.2%. Hypocortisolemia was found in 78 (59%) patients. Fifty-seven patients (73%) with cortisol deficiency had severe ARDS versus only 46 (85%) in the group with a normal cortisolaemia (p = 0.325). Twenty-nine patients (37%) with cortisol deficiency required intubation vs 36 (67%) in the normal group; p = 0.001. The occurrence of infections during hospitalization was observed in 30 (38.5%) patients with cortisol deficiency versus 28 (52%) in the other group (p = 0.15). Mortality was observed in 32 patients with cortisol deficiency (41%) versus 33 (61%) in the other group.

**Conclusion:** Cortisol deficiency was frequently observed in our study but it was not associated with poor outcome in critically ill COVID-19 patients.

**Compliance with ethics regulations:** Yes in clinical research.

### FC-011 Nutrition in critically ill patients: experience of a multidisciplinary improvement quality program

#### DE KEYSER Aude^1,2^, MICHEL Bruno^1,2^, GALERNEAU Louis-Marie^1^, LUMALE Sophie^1^, FERRAND Benoit^1^, CALVINO-GUNTHER Silvia^1^, GIROUD Benoit^1^, MAHI Lena^1^, IMBERT Wendnonga^1^, CHAPUIS Claire^1^, SCHWEBEL Carole^1^

##### ^1^Centre Hospitalier Universitaire Grenoble-Alpes, Grenoble, France; ^2^Cliniques Universitaires Saint-Luc, Bruxelles, Belgique

###### Correspondence: Aude DE KEYSER (aude.dekeyser@saintluc.uclouvain.be)

*Annals of Intensive Care* 2022, **12(1):**FC-011

**Rationale:** Critically ill patients face significant catabolic situations. Nutrition is part of management beside various organ supply. The Intensive Care Unit (ICU) of our 2200-bed tertiary university hospital starts a quality and continuous improvement program for year 2021 targeting artificial nutrition. The objective of the work was to involve healthcare providers in evaluation and improvement of nutrition management in daily ICU practice.

**Patients and methods/Materials and methods:** A multidisciplinary board composed of physicians (3), clinical pharmacists (3), nurses (3), nursing auxiliaries (2), physiotherapists (2) and dietitian (1) was set up in January 2021. A baseline audit was conducted in April 2021 in the entire unit (18 beds) focusing on concordance with current recommendations between prescribed and administered nutritional intakes and traceability of written transmission. The board identified specific areas for quality improvement for each professional category. Impact of implemented measures was evaluated through a second identical audit in February 2022.

**Results:** The audit conducted over 10 days in April 2021 highlighted proper artificial nutrition practices. All included patients (n = 18; 60 ± 15 yo; 92 ± 23 kg; IGS2 48 ± 7) had enteral nutrition (EN) within 48 h of admission. Half of patients had a BMI over 30. Included population mean ICU duration was 11,3 days and nutrition support was lasting for 10,9 days at time of audit. No patients received parenteral nutrition (PN). At observation patients reached an average of 96% of recommended caloric targets with only 14/18 having nutritional objectives achieved at Day-7 of nutritional support. Other discrepancies included: adaptation to ideal body weight (0/9), protein target achievement (6/18), absence of complementary PN, lack of traceability of nutrition interruption (5/18), mismatch between either location or diameter of feeding tube (11/18), wrong or incomplete bedside EN identification (18/18). Major contributions resulted in weekly theorical and practical training sessions, digital tools for ICU providers, updated and standardized procedures for nutrition support initiation and follow up in various patient profile. Second audit is taking place during February 2022.

**Conclusion:** Nutrition brings ICU caregivers in a continuous improvement program. It favours interprofessional interactions for direct patients benefit and proved importance and performance of multidisciplinary approach for quality improvement. Preliminary data issued from second audit are encouraging but sustaining efforts are required for long lasting results. Other areas of ICU management may be eligible according to the same model.

**Reference 1:** D. Hurel, J. Y. Lefrant, N.J. Cano, C. Ichai, J. C. Preiser, F. Tamion. Nutrition artificielle en réanimation. Réanimation (2014) 23:332–350.

**Reference 2:** P. Singer, A.R. Blaser, M.M. Berger, W. Alhazzani, P.C. Calder, M. Casaer, M. Hiesmayr, K Mayer, J.C. Montejo, C. Pichard, J-C.Preiser, A.R.H. van Zanten, S. Oczkowski, W. Szczeklik, S.C. Bischoff. ESPEN guideline on clinical nutrition in the intensive ca.

**Compliance with ethics regulations:** N/A.

### FC-012 Assessment of nutritional support in a medical ICU

#### PERRIN Clémence^1^, CIDERON Cédrick^1^, LABRO Guylaine^1^, DUREAU Anne Florence^1^, KUTEIFAN Khaldoun^1^

##### ^1^GHRMSA, Mulhouse, France

###### Correspondence: Clémence PERRIN (clemenceperrin@gmail.com)

*Annals of Intensive Care* 2022, **12(1):**FC-012

**Rationale:** According to the recommendations, for all patients admitted to intensive care and likely to stay longer than 48 h nutrition must be quickly introduced. Lack of access to indirect calorimetry requires calculating the necessary nutritional intake according to the weight and the duration of hospitalization in ICU. Our objective is to assess caloric intake (nutritional and non-nutritional) in our medical intensive care unit.

**Patients and methods/Materials and methods:** This study is descriptive and retrospective. All patients admitted between July 1 and September 30, 2021 and hospitalized more than 3 days, were included. We collected daily nutritional and non-nutritional caloric intake during the whole hospitalization. The weight used for calculation was the one measured at the admission (BMI < 30 kg/m^2^) or the adjusted weight (BMI ≥ 30 kg/m^2^).

**Results:** 180 patients were admitted and 140 were included. The average age was 59.7 years (± 12), 61% were male. The average of SOFA score was 7.8 (± 4.3) and the average of SAPS II score was 45 (± 19.4). The average length of ICU stay was 8.1 days (± 5.9) and the mortality rate was 23.8%. At 48 h, 62 patients (44.3%) received nutrition. Among these patients, 49 received oral nutrition and 13 received enteral artificial nutrition. None of them received a parenteral artificial nutrition. From 140 patients, excluding 49 patients with an unquantifiable oral diet, the average caloric intake was 5.9 kcal/kg/day (± 2.8) for 91 patients. This caloric intake was distributed as follows: 49% from glucose solutions, 26.4% from lipids contained in propofol, 25.1% from artificial nutrition. At 7 days, 54 patients still hospitalized In ICU and 39 patients (72%) received an enteral nutrition. The caloric intake was 18.4 (± 7.4) kcal/kg/day.

**Discussion:** At 48 h, most of patients fed received a standard oral nutrition with an unknown number of calories ingested. During the first 3 days, if patients did not eat in the standard way, they had little or no nutritional support. Most of the calories ingested during the first 3 days of resuscitation are provided by therapies such as propofol and glucose. These therapies are administered either for sedation, or as a carrier or solvent, or for hydration, but not for nutritional purpose. The caloric intake targets were not reached according to the recommendations in force. However, the mortality rate is average according to the SAPS score.

**Conclusion:** In our department, patientscaloric intake was lower than recommended. Many of caloric intake was brought by non-nutritional caloric intake in acute phase.

**Reference 1:** Guidelines for Nutrition Support in Critically Ill Patient, Réanimation (2014) 23:332–350.

**Reference 2:** ESPEN guideline on clinical nutrition in the intensive care unit. Singer P, et al. Clin Nutr. 2019.

**Compliance with ethics regulations:** Yes in clinical research.
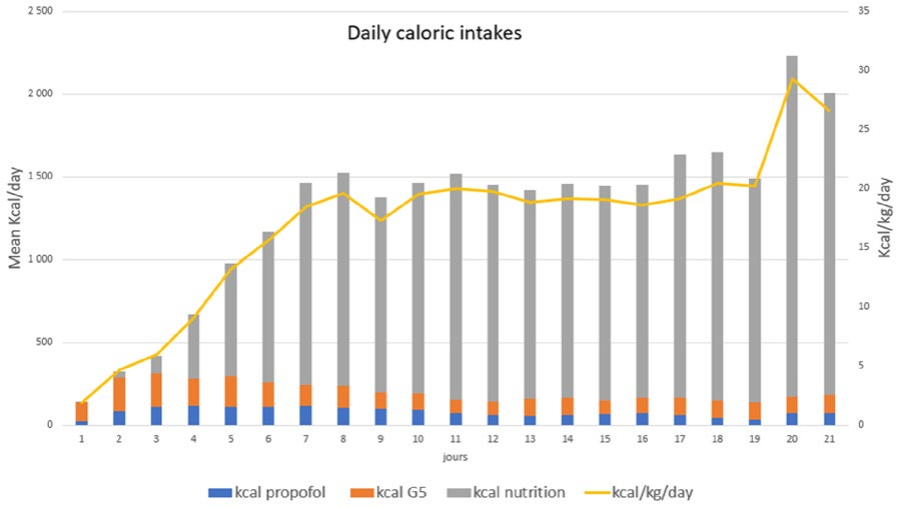


*Daily caloric intake*

### FC-013 Obese tool box evaluation

#### MAYENCO CARDENAL Nicolas^1^, SZTRYMF Benjamin^1^

##### ^1^Hôpital Antoine Béclère, Clamart, France

###### Correspondence: Nicolas MAYENCO CARDENAL (nmayencocardenal@gmail.com)

*Annals of Intensive Care* 2022, **12(1):**FC-013

**Rationale:** Obese patients are more and more frequent in the Intensive Care Units (ICU). This group of patients has unique pathophysiologic features involving all organs. They raise special challenges about their management in ICUs. Aspects like mechanical ventilation, artificial nutrition, thromboprophylaxis or antibiotic dosing can be difficult. Guidelines and clinical data are available about obese patient care, but their application in every day’s practice is uncertain. In the ICU in Antoine Beclere’s hospital (Clamart), we have created a clinical decision support system that brings the existing guidelines about those four features to the physicians in a friendly-user interface. In this study we have assessed the impact of this support system on the physician’s prescriptions.

**Patients and methods/Materials and methods:** In this retrospective study we have collected data about prescriptions and outcomes of obese patients during two periods, before and after the support system was made available. The endpoint was the number of prescriptions following the existing guidelines. We have also assessed the physician’s satisfaction through a survey.

**Results:** Over a 3-year period, 39 patients have been included (23 before and 15 after the availability of the support system). They represent 500 days of ICU admission. Patients were comparable in the two groups. They had a mean age of 62.35 years, mean BMI of 39.13 kg/m^2^ and SOFA score of 4.71. For patients in the second group, prescribed tidal volume was lower (6.57 ml/kg of ideal body weight vs. 7.03 ml/kg; p < 0,001). Less overfeeding was observed (26.64 kcal/kg of adjusted body weight vs 28.32 kcal/kg; p = 0,03) without improving protein intake. Thromboprophylaxis prescriptions were more frequently in accordance with guidelines (74.8% vs 57,7%; p = 0,001). No difference was observed in antibiotic prescription or plasmatic concentration measurements. Surveyed physicians considered thromboprophylaxis and nutrition advice the most useful. They have used the support system several times when caring for an obese patient.

**Conclusion:** In our UCI, the availability of a computer clinical decision support system for prescription in obese patients brought physician’s prescriptions closer to existing guidelines. Physicians thought the system was useful and user-friendly, especially for nutrition and thromboprophylaxis aspects.

**Compliance with ethics regulations:** Yes in clinical research.

### FC-014 Non thyroidal illness syndrome in critically ill patients with SARS-CoV-2 infection

#### TURKI Olfa^1^, SMAOUI Ayoub^1^, CHTARA Kamilia^1^, BRADAII Sabrine^1^, BAHLOUL Mabrouk^1^, BOUAZIZ Mounir^1^

##### ^1^CHU Habib Bourguiba Sfax, Sfax, Tunisie

###### Correspondence: Olfa TURKI (olfa.turki.rea@gmail.com)

*Annals of Intensive Care* 2022, **12(1):**FC-014

**Rationale:** “Non thyroidal illness syndrome” (NTIS) or “euthyroid sick syndrome” (ESS) is a possible biochemical finding in euthyroid patients with severe diseases. It is characterized by a reduction of serum T3 (fT3). The relationship between thyroid hormones levels and mortality is well known. The sudden spread of the 2019 novel coronavirus (SARS-CoV 2) infection (COVID-19) and its high mortality become a world healthcare problem. Our purpose was to investigate the incidence of this endocrinal perturbation in COVID-19 infected patients and the relationship between thyroid function and severity of this infection.

**Patients and methods/Materials and methods:** We prospectively considered patients admitted in SARS-CoV2 ICU Unit within 2 months. The SARS-Cov-2 infection was confirmed in all patients by PCR testing. Blood samples were collected within 48 h of admission and prior to any treatment that may affect thyroid hormone, on the third day and on the seventh day of ICU stay. NTIS was defined as serum FT3 levels < 2 pg/ml, FT4 and TSH levels within or below the normal reference ranges.

**Results:** We enrolled 57 patients. The average age was 64 ± 11.7 years with a sex ratio at 1.28. The average ICU stay was 7 days (2-31 days). Only 16 (28%) patients had normal thyroid hormone levels while 41 (72%) had developed NTIS. Thirty-one patients (54.4%) developed NTIS on admission. On the third day, 42% of ICU patients developed NTIS and they were 14 of 21 patients (66.6%) who developed this thyroid perturbation on the seventh day. Overall, troponin, cortisol-level, severe ARDS, ventilation and septic shock predicted disease severity and death. Patients with NTIS had worse symptomatology, worse profiles of inflammatory and tissue injury markers. But neither the NTIS, nor the Rt3 level predicted poor issue in our cohort.

**Conclusion:** NTIS pattern is common and relates to the severity of disease rather than SARS-CoV-2 infection. Thus, understanding the pathophysiology and evolution of NTIS is crucial even though it rarely require specific treatment. It is suggested that thyroid hormone monitoring in COVID-19 should not differ from other critically ill patients and it is important that endocrinologists recognize them to ensure appropriate management, particularly in the acute phase.

**Compliance with ethics regulations:** Yes in clinical research.

### FC-015 Diabetic ketoacidosis in intensive care unit: epidemiological, clinical and evolutionary features

#### GUISSOUMA Jihene^1,2^, ALLOUCHE Hend^1,2^, BEN ALI Hana^2^, ABDOU Ghada^1,2^, TRABELSI Insaf^1,2^, GHADHOUNE Hatem^1,2^

##### ^1^Faculté de médecine de Tunis, Bizerte, Tunisie; ^2^Université Tunis El Manar, Tunis, Tunisie

###### Correspondence: Jihene GUISSOUMA (guissouma.jihene@gmail.com)

*Annals of Intensive Care* 2022, **12(1):**FC-015

**Rationale:** Diabetic ketoacidosis (DKA) is a serious metabolic complication of diabetes. It is a potentially life-threatening condition and usually requires intensive care unit (ICU) hospitalization. We aimed to describe the epidemiological, clinical and evolutionary features of DKA managed in ICU.

**Patients and methods/Materials and methods:** A 7-year retrospective, descriptive and longitudinal single-center study including all patients admitted to a 6-bed ICU for DKA. Statistical analysis was performed using SPSS 23.

**Results:** Sixty-two patients were enrolled. The mean age was 36 ± 16 years with a female predominance (sex ratio 0.55). The most common type of diabetes was type 1 (60%), followed by type2 (21%) and inaugural diabetes (19%). The mean duration of diabetes was 9 ± 8 years. The most common comorbidities were hypertension (28%) and chronic renal failure (23%). The mean duration of symptoms before hospitalization was 20 ± 8 h. The most common were asthenia (92%) and nausea, vomiting with abdominal pain (69%). Confusion or less alertness and increase in polydipsia and polyuria were noted in 37% and 32% of cases respectively. On admission, 47% of patients had dehydration, 22% shock and 16% were comatose. Fifty percent had acute kidney injury. The mean capillary blood glucose measurement was 5.8 ± 1.6 g. The average levels of pH and bicarbonates were 7.12 ± 0.14 and 6 ± 4 respectively. DKA was severe, moderate and mild in 84%, 11% and 5% of cases respectively. The mean IGS II and APACHE II were 22 ± 18 and 13 ± 10 respectively. Infection was the predominant precipitating factor (50%) followed by non-compliance to treatment (30%). The symptoms regressed after restoration of fluid deficits, electrolyte replacement and continuous intravenous insulin therapy. Vasopressors and mechanical ventilation (MV) were imperative in 19% and 11% of cases respectively. Only 8% required hemodialysis. DKA resolved within 20 h (extremes 5 and 33 h). The mean length of stay in ICU was 5 ± 3 days and mortality rate was 13%. In univariate analysis: IGS II (p < 10^−3^), APACHE II (p < 10^−3^), GCS (p < 10^−3^), shock on admission (p = 0.01) and MV (p < 10^−3^) were all predictors of mortality. Besides APACHE II (p = 0.03) and MV (p = 0.009) were the two independent prognostic factors in multivariate analysis.

**Conclusion:** DKA occurs mainly in type1 diabetes. Although it was severe in most cases, it resolved within a few hours. However, mortality was relatively high in this study and was significantly associated to high APACHE II and need of mechanical ventilation. Adequate follow up and better diabetes education are necessary to avoid this serious metabolic complication.

**Compliance with ethics regulations:** Yes in clinical research.

### FC-016 Early-onset acute kidney injury in COVID-19 critically ill patients, a monocentric retrospective analysis (SARCOV-AKI)

#### RUAULT Alice^1^, DUPUIS Claire^1^, EVRARD Bertrand^1^, CALVET Laure^1^, THOUY François^1^, GRAPIN Kévin^1^, GUIDO Olivia^1^, HERNANDEZ Gilles^1^, DOPEUX Loïc^1^, MASCLE Olivier^1^, BOUZGARROU Radhia^1^, ADDA Mireille^1^, BONNET Benjamin^1^, SAPIN Vincent^1^, PHILIPPONNET Carole^1^, SOUWEINE Bertrand^1^

##### ^1^CHU Gabriel Montpied, CHU Clermont Ferrand, Clermont Ferrand, France

###### Correspondence: Claire DUPUIS (cdupuis1@chu-clermontferrand.fr)

*Annals of Intensive Care* 2022, **12(1):**FC-016

**Rationale:** The aim of the study was to assess the epidemiology of early-onset Acute kidney injury (EO-AKI) in COVID-19 pneumonia patients admitted to ICU.

**Patients and Methods/Materials and Methods:** This study, approved by the ethical committee of the French Intensive Care Society (CE-SRLF20-20), was a prospective single center study, performed in the medical ICU of the university hospital of Clermont-Ferrand France. All consecutive adult patients aged ≥ 18 years, admitted between March 20th, 2020 and August 31th, 2021 for COVID-19 pneumonia were enrolled. Patients with chronic kidney disease, referred from another ICU, and ICU length of stay (LOS) ≤ 72 h were excluded. Baseline patients’ characteristics, variables recorded regarding ICU admission, organ support and main laboratory features during ICU stay and outcomes were collected. Biological testing on admission included mHLA-DR, plasma cytokines (IL-6, CXCL8, IFN-alpha, IL-10 and IL1Ra); and AKI urinary (L-FABP, PODXL, TIMP-2IGFP7) and serum biomarkers(s-Rage, SuPAR). AKI was defined according to the KDIGO classification and classified according to the timing of their recovery as transient, persistent and acute kidney disease (AKD) (ref 1). EO-AKI was defined as the first episode of AKI occurring within 7 days of ICU admission. MAKE (Major Adverse Kidney Events) including death or new RRT or no renal recovery during the first 90 days after ICU admission were recorded. Uni and multivariate analyses were performed to determine factors associated with the presence of AKI and predictors of MAKE.

**Results:** 264 patients were included in this study. Patients’ characteristics are described in Table 1. EO-AKI occurred in 87 patients (33%), including 43, 17 and 27 patients with EO-AKI stage 1, 2 and 3, respectively. EO-AKI was classified as transient, persistent and AKD in 31 (35.6%), 14 (16%) and 42 (48.2%) patients, respectively. The median ICU LOS was 8 days [5;13]. The ICU mortality was 30.4% (N = 80), and increased with EO-AKI occurrence and severity: 15.8%, 46.5%, 64.7% and 77.8% in patients without AKI, and with EO-AKI stage 1, 2 or 3, respectively (P < 0.001). ICU mortality in patients with transient, persistent AKI and AKD were 50%, 50% and 77.4%, respectively and was significantly lower in patients with EO-AKI not developing AKD than in patients developing (P < 0.01).

**Conclusion:** In this single center study, EO-AKI developed in 30% of COVID patients admitted to ICU. EO-AKI stage 3 or AKD was associated with a fatal outcome in ≥ 77% of cases. Preventing EO-AKI occurrence and achieving EO-AKI recovery is of paramount importance in COVID-19 patients.

**Reference 1:** Darmon, ICM, 2018.

**Compliance with ethics regulations:** Yes in clinical research.
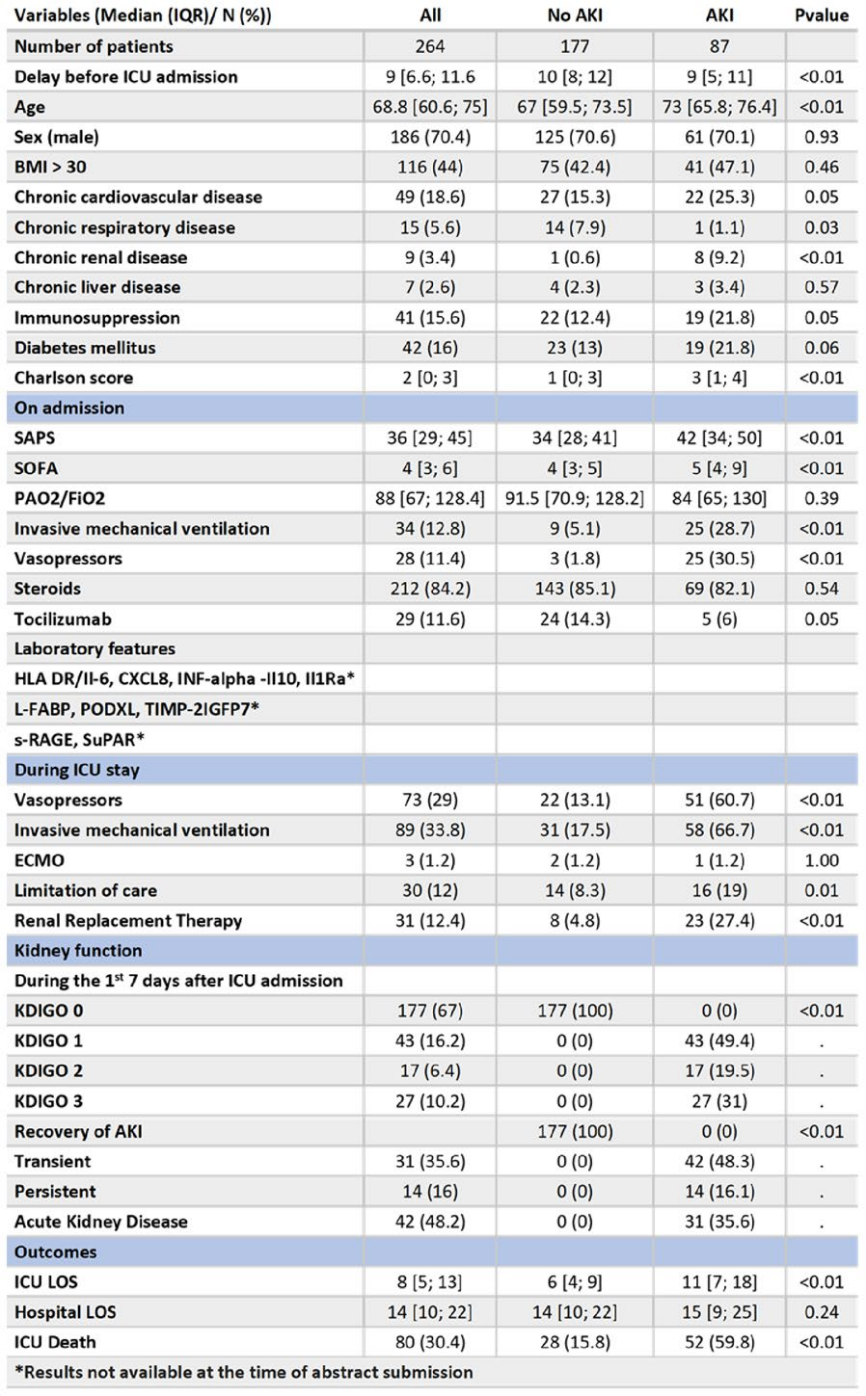


*Characteristics and comparisons of COVID-19 ICU patients with and without Early Onset AKI*

### FC-017 Acute kidney injury in critically ill COVID 19 patients: clinical features

#### BEN MILED Cherifa^1^, SOUILHI Jawhar^1^, DRIDI Amira^1^, NAIMI Skander^1^, FITOUHI Nizar^1^, OUERGHI Sonia^1^, MESTIRI Tahar^1^

##### ^1^Hôpital Abderrahman Mami- Ariana, Tunis, Tunisie

###### Correspondence: Cherifa BEN MILED (cherifa.bm@gmail.com)

*Annals of Intensive Care* 2022, **12(1):**FC-017

**Rationale:** Acute kidney injury is a frequent complication in critically ill COVID 19 patients and is associated with severe outcomes. This study aims to describe the clinical characteristics of this Acute kidney injury and to assess its incidence.

**Patients and methods/Materials and methods:** A retrospective and descriptive study was conducted including all covid-19 patients admitted in our ICU who presented an acute kidney injury defined by the KDIGO criteria, during a 6-month period. We collected demographic and anthropometric parameters, underlying comorbidities, nephrotoxic medicine prescription, clinical features of the acute renal failure with the KDIGO grade, mechanical ventilation characteristics, requirement of kidney replacement therapy and outcomes. Data analysis was performed using SPSS 28.0 software.

**Results:** During our study period, 147 patients were admitted in our ICU. AKI incidence was 52,2% (71 patients out of 147). According to KDIGO criteria, 16.9% were staged KDIGO 1, 35.2% KDIGO 2 and 47.8% KDIGO 3. The mean age was 61 years, ranged from 27 to 93 years. 38% were considered as elderly (> 65 years old). The main comorbidities were arterial hypertension (66%), diabetes mellitus (54.9%), atrial fibrillation (28.1%) and obesity (22.5%). Acute kidney injury occurred mainly around the 6th day of ICU and resolved after 7 days. For 18.3% of the patients, the AKI was transient, defined as a return to the baseline serum creatinine levels or its decrease of 50% or more within the 72 h after the onset of the AKI. Most of the patients were intubated (87%) and 96% were ventilated with a high PEEP (> 12 cmH_2_O). AKI occurred after the intubation in 73.2% of the cases, 2 days after on average. In 25.4% of the cases, AKI was associated with septic shock. Prescription of nephrotoxic drugs was identified in 77.4% of cases. 12 patients (16.9%) required a replacement kidney therapy, but only 2 of them had a dialysis session, due to the lack of dialysis facilities in our ICU. The 28-day mortality rate was 84,5%.

**Conclusion:** The incidence of Acute kidney injury in our ICU was 52.2% and occurred mainly in elderly patients with underlying comorbidities. Most patients required a mechanical ventilation with high levels of PEEP. The 28-day mortality was 84,5%.

**Compliance with ethics regulations:** Yes in clinical research.

### FC-018 Clinical features and outcomes of Acute Kidney Injury in severe to critical COVID-19 patients

#### BOUGUEZZI Nabil^1^, BEN SAIDA Imen^1,2^, TOUMI Radhouane^1,2^, MAATOUK Iyed^1^, ZGHIDI Maroua^1^, ZOUARI Hajer^1^, MEDDEB Khaoula^1,2^, BOUSSARSAR Mohamed^1,2^

##### ^1^Farhat Hached University Hospital, Sousse, Tunisie; ^2^Research Laboratory N° LR12SP09. Heart Failure. Farhat Hached University Hospital, Sousse, Tunisie

###### Correspondence: Nabil BOUGUEZZI (dr_nabil@live.fr)

*Annals of Intensive Care* 2022, **12(1):**FC-018

**Rationale:** Coronavirus disease 2019 (COVID-19) is a respiratory illness caused by an emerged virus SARS-CoV-2. An alarming number of patients with SARS-CoV-2 infection especially severe cases have been reported to develop Acute Kidney Injury (AKI). Our study is aimed to investigate features, outcomes and risk factors for Acute Kidney Injury in Critically Ill COVID-19 patients.

**Patients and methods/Materials and methods:** It is a retrospective observational study conducted from March 2020 to October 2021, in a Medical ICU. Information regarding demographic, clinical characteristics and outcomes of Critically Ill COVID-19 patients was obtained from medical records. AKI was defined according to the Kidney Disease Improving Global Outcomes (KDIGO) clinical practice Guideline. Multivariate analysis was performed to evaluate risk factors for AKI.

**Results:** 442 critically ill COVID-19 patients were admitted to ICU during the study period. Median age, 64 [54–71] years; 279(63.1) were males. Hypertension, 197(44.6); diabetes mellitus, 187(42.3) and chronic kidney disease 36(8,1). Median SAPS II, 28 [22–35]. All patients had acute hypoxic respiratory failure and needed either noninvasive or invasive mechanical ventilatory support. 215(48.6) patients required invasive mechanical ventilation (IMV) and 195(44) received a vasoactive drug. 209(47,2) developed AKI during hospitalization. Among the 209 patients with AKI, 31(14.8), 22(10.5) and 156(74.6) had stage 1, 2, and 3, respectively. AKI occurred within a median of 5[1–24] days of hospitalization. 37(21,0) patients received renal replacement therapy, with a total of 117 sessions. The causes of acute kidney injury can be divided into two categories, hypoxic acute tubular necrosis in 177(84) and type 1 cardiorenal syndrome in 31(14.8) patients. The mortality was significantly higher in AKI group compared to those without (54% vs 43%, p 0.01). The mortality rate was 6.6% (n = 14), 3.8% (n = 8), and 42% (n = 89) among those with AKI stages 1, 2, and 3, respectively. On univariate analysis, the factors predicting AKI were mechanical ventilation(p = 0.05), cardiogenic shock (p = 0.001), positive fluid balance (p = 0.000), vasopressors use (p = 0,019); Multivariate regression model identified the following factors as independently associated to AKI, cardiogenic shock, (OR, 2.59; 95% CI, [1.5–4.3]; p = 0.000); positive fluid balance, (OR, 1.76; 95% CI, [1.3–2.2]; p = 0.000).

**Conclusion:** AKI among patients with severe to critical COVID-19 was common. Several factors were shown to contribute to its occurrence. This complication was highly associated with mortality.

**Compliance with ethics regulations:** Yes in clinical research.

### FC-019 Incidence and risk factors of acute kidney injury in critical forms of COVID-19

#### GUISSOUMA Jihene^1,2^, BEN ALI Hana^2^, TRABELSI Insaf^1,2^, ALLOUCHE Hend^1,2^, SAMET Mohamed^2^, BRAHMI Habib^2^, GHADHOUNE Hatem^1,2^

##### ^1^Faculté de médecine de Tunis, Bizerte, Tunisie; ^2^Université Tunis El Manar, Tunis, Tunisie

###### Correspondence: Jihene GUISSOUMA (guissouma.jihene@gmail.com)

*Annals of Intensive Care* 2022, **12(1):**FC-019

**Rationale:** Acute kidney injury (AKI) is a common metabolic complication in the COVID-19 particularly among critical forms. Besides the viral tropism the pathogenesis of AKI is likely multifactorial. We aimed to describe the epidemiological, clinical and biological features of patients admitted to intensive care unit (ICU) for critical forms of COVID-19 in order to deduce the risk factors of AKI.

**Patients and methods/Materials and methods:** A 16-month prospective analytic study (September 2020–December 2021) including all patients (without a chronic kidney failure history) admitted to ICU for critical forms of COVID-19. The patients were divided into two groups: -Group 1: patients who developed AKI during ICU stay. -Group 2: patients who have maintained normal kidney function. The KDIGO classification was adopted to stratify the AKI. The statistical analysis was performed using SPSS 23.

**Results:** Overall 140 patients were included. Twenty-three per cent developed AKI (33 cases in group 1 and 107 in group 2). The mean age was 58 ± 13 with male predominance (sex ratio 1.2). The most common comorbidities were hypertension (64%) and diabetes (62%). The mean IGS II and APACHE II were 40 ± 13 and 13 ± 6 respectively. The mean base creatinine level was 79 ± 22 mmol/l. The mean creatinine level at the onset of AKI was 295 ± 201 mmol/l. According to the KDIGO criteria the group 1 patients were staged class I (4 cases), class II (11 cases) or class III (18 cases). The mean period for developing AKI was 8 days. Sepsis and hypotension were the two predominant precipitating factors of the AKI (45% and 33% of cases of group 1). Hemodialysis was indicated in 40% of cases. The AKI was irreversible in 72% of the cases. In univariate analysis, there were no significant differences between the two groups in gender, comorbidities, IGS II and ICU length of stay. However, age (p = 0.007), Apache II (p = 0.01) and septic shock (p < 10-3) were the main risk factors of AKI. Only septic shock (p < 10-3) was an independent factor in multivariate analysis. Besides group 1 patients required more invasive ventilation (p = 0.001) and developed much more healthcare-associated infections (p < 10-3). Overall mortality rate was 69%. It was higher in group 1 (87%) compared to group 2 (63%) with a significant difference (p = 0.029).

**Conclusion:** The occurrence of AKI in critical forms of COVID-19 was frequent in our study and was associated to a higher mortality. Septic shock was the main risk factor of AKI. These results need to be confirmed by further larger studies.

**Compliance with ethics regulations:** Yes in clinical research.

### FC-020 Impact of dexamethasone and inhaled nitric oxide on severe acute kidney injury in critically ill patients with COVID-19

#### BOBOT Mickaël^1,2,7^, TONON David^3^, PERES Noémie^2^, GUERVILLY Christophe^2^, LEFÈVRE Flora^1^, MAX Howard^4^, BOMMEL Youri^4^, VOLFF Maxime^4^, LEONE Marc^5^, LOPEZ Alexandre^5^, SIMEONE Pierre^4^, CARVELLI Julien^6^, HRAIECH Sami^2^, PAPAZIAN Laurent^2^, VELLY Lionel^4^, BOURRENNE Jérémy^6^, FOREL Jean-Marie^2^

##### ^1^Centre de Néphrologie et Transplantation Rénale, Hôpital de la Conception, AP-HM, Aix-Marseille Université, Marseille, France; ^2^Service de Médecine Intensive Réanimation, Hôpital Nord, AP-HM, Aix-Marseille Université, Marseille, France; ^3^Département d'Anesthésie Réanimation, Hôpital de la Conception, AP-HM, Aix-Marseille Université, Marseille, France; ^4^Département d'Anesthésie Réanimation, Hôpital de la Timone, AP-HM, Aix-Marseille Université, Marseille, France; ^5^Service d'Anesthésie Réanimation, Hôpital Nord, AP-HM, Aix-Marseille Université, Marseille, France; ^6^Service de Réanimation et Surveillance Continue, Hôpital de la Timone, AP-HM, Aix-Marseille Université, Marseille, France; ^7^C2VN, INSERM 1263, INRAE 1260, Aix-Marseille Université, Marseille, France

###### Correspondence: Mickaël BOBOT (mickael.bobot@ap-hm.fr)

*Annals of Intensive Care* 2022, **12(1):**FC-020

**Rationale:** Kidney failure is the second most frequent condition after acute respiratory distress syndrome (ARDS) in critically ill patients with severe COVID-19 and is strongly associated with mortality. The aim of this multicentric study was to assess the impact of the specific treatments of COVID-19 and ARDS on the risk of severe acute kidney injury (AKI) in critically ill COVID-19 patients.

**Patients and methods/Materials and methods:** In our cohort study, we retrospectively analysed a database of consecutive patients hospitalized in 6 ICUs for COVID-19. The incidence and severity of AKI were monitored during the entire ICU stay. Patients older than 18 years admitted to the ICU for COVID-19-related ARDS requiring mechanical ventilation were included.

**Results:** 164 patients were included in the final analysis, 97 (59.1%) displayed AKI, of which 39 (23.8%) severe stage 3 AKI and 21 (12.8%) requiring renal replacement therapy (RRT). In univariate analysis, severe AKI was associated with Angiotensin Converting Enzyme inhibitors (ACEI) exposure (p = 0.016), arterial hypertension (p = 0.029), APACHE-II score (p = 0.004) and mortality at D28 (p = 0.008), D60 (p < 0.001) and D90 (p < 0.001). In multivariate analysis, the factors associated with the onset of stage 3 AKI were: exposure to ACEI (OR: 4.238 (1.307–13.736), p = 0.016), APACHE II score (without age) (OR: 1.138 (1.044–1.241), p = 0.003) and iNO (OR: 5.694 (1.953–16.606), p = 0.001). Protective factors were prone positioning (OR: 0.234 (0.057–0.967), p = 0.045) and dexamethasone (OR: 0.194 (0.053–0.713), p = 0.014).

**Discussion:** Our study confirms the negative impact of AKI on mortality in COVID-19 in critically ill patients, and then the need to be careful about risk factors of AKI and to implement strategies to limit nephrotoxicity in the ICU. We report for the first time an independent association of iNO with both severe AKI and the need for RRT in severe COVID-19 patients. In our study, iNO did not improve mortality rates or the duration of MV and was associated with AKI and RRT. Thus, we suggest against its using in COVID-19-related ARDS, when other therapeutic strategies are available. Dexamethasone could decrease glomerular and interstitial inflammation observed in kidneys, decreasing the risk of AKI during severe COVID-19, which may have contributed to a decrease in the AKI rate between the first and the second wave of COVID pandemics.

**Conclusion:** Dexamethasone was associated with a prevention of the risk of severe AKI and RRT, and iNO was associated with severe AKI and RRT in critically ill patients with COVID-19; iNO should be used with caution in COVID-19 related ARDS.

**Reference 1:** Orieux A et al. Impact of dexamethasone use to prevent from severe COVID-19-induced acute kidney injury. Crit Care Lond Engl. 2021 Jul 16;25(1):249.

**Reference 2:** Ruan SY et al. Inhaled nitric oxide therapy and risk of renal dysfunction: a systematic review and meta-analysis of randomized trials. Crit Care. 2015 Dec;19(1):137.

**Compliance with ethics regulations:** Yes in clinical research.
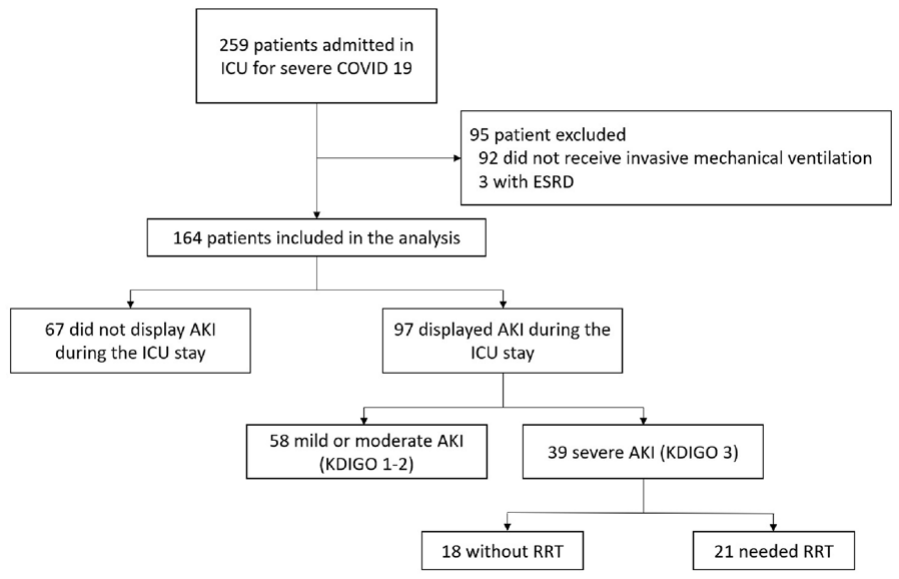


*Figure 1: Flow chart of the study AKI: Acute Kidney Injury, ESRD: End-stage renal disease, ICU: Intensive Care Unit, RRT: Renal replacement therapy*

### FC-021 Is hyponatremia associated with poor outcome in critically ill COVID-19 patients?

#### SDIRI Ines^1^, JARRAYA Fatma^1^, RACHDI Emna^1^, JAMOUSSI Amira^1^, AYED Samia^1^, BEN KHELIL Jalila^1^

##### ^1H^ôpital Abderrahmane Mami, Ariana, Tunisie

###### Correspondence: Fatma JARRAYA (fatma.jarraya8@gmail.com)

*Annals of Intensive Care* 2022, **12(1):**FC-021

**Rationale:** Patients with COVID-19 have multiple clinical conditions that may cause electrolyte imbalances. Hyponatremia is a prevalent metabolic disorder that could be involved with adverse outcomes in critically ill COVID-19 patients. The aim of this study was to describe the impact of hyponatremia on outcomes of COVID-19 patients, including mortality.

**Patients and methods/Materials and methods:** This was retrospective observational cohort study included all consecutive COVID19 patients admitted to the intensive care unit from March 2020 to September 2021. All COVID-19 patients in whom serum sodium was measured at admission were included. Patients were divided into two groups: GI patients without hyponatremia; G II patients with hyponatremia (serum sodium < 135 mmol/L). Outcomes such as mortality, need for invasive mechanical ventilation (IMV), sepsis, and acute kidney injury (AKI) were assessed.

**Results:** During the study period, 520 patients were collected and divided into two groups (G I n = 394; G II n = 126). The median age was 61 years [52-69] with a gender ratio (H/F) of 1.65. The mean of the SAPS II score was 27.9 ± 10. The most common comorbidities were hypertension (38.2%) and diabetes (36.5%). The mean body mass index was 30.79. The incidence of hyponatremia at admission was 23.4% and it was generally mild (n = 100; 79%). Demographic characteristics were similar between the two groups. Transit disorders particularly emesis prior to admission were more common in the hyponatremia group (21% vs 13.3%; p = 0.055). Pulmonary bacterial co-infection occurred more in GII (14.3% vs 7.8%; p = 0.030). Hyponatremic patients had higher ferritin levels (1500 μg/l [205–1500] vs 401 μg/l [250–1030]; p = 0.05). Hyponatremia was not associated with increased risk of AKI (48.3% vs 41.8%; p = 0.403) or sepsis (35.8% vs 26.1%; p = 0.140). IMV and death were not significantly more frequent in hyponatremic patients compared with normonatremic patients (61.1% vs 55.3%; p = 0.257; 59.5% vs 52.3%; p = 0.156, respectively).

**Conclusion:** Mild hyponatremia at admission is common but is not associated with poor outcomes in patients with critical COVID-19.

**Compliance with ethics regulations:** Yes in clinical research.

### FC-022 Hypokalemia in critically ill COVID-19 patients

#### HADDED Amina^1^, ALILA Ilef^1^, KHARRAT Sana^1^, BRADAI Sabrine^1^, BAHLOUL Mabrouk^1^, BOUAZIZ Mounir^1^

##### ^1^hopital Habib Bourguiba Sfax, Sfax, Tunisie

###### Correspondence: Ilef ALILA (ilefalila1323@gmail.com)

*Annals of Intensive Care* 2022, **12(1):**FC-022

**Rationale:** Patients with COVID-19 have multiple clinical conditions that can cause electrolyte imbalances, including hypokalemia. This study aimed to estimate the prevalence and outcomes of hypokalemia in patients withCOVID-19 admitted to intensive care units (ICU).

**Patients and methods/Materials and methods:** We conducted a retrospective study in an ICU over a period of 16 months [September 2020–December 2021] including patients with SARS-COV2 infection.

**Results:** During the study period, 586 patients were included with a mean age of 59.5 ± 14.7 years and a sex ratio of 1.6. The median SAPSII and SOFA score were respectively of 29 ± 14.8 and 4 ± 2.6. Most patients had comorbidities, including hypertension (36%), obesity (32.1%) and diabetes mellitus (36.2%). A total of 52 patients (9%) had hypokalemia. Severe ARDS was observed in 63% of the first group versus 72% of the second group (p = 0.109). No significant difference was found in terms of use of NIV (59.6% versus 65%; p = 0.4) and invasive mechanical ventilation (75% versus 72.8%; p = 0.73). The mean duration of mechanical ventilation was longer in the hypokalemia group: 7.7 days versus 4.4 days in the second group (p < 0.001). Similarly, the average length of stay in the intensive care unit was higher in the first group than in the second group: 9.3 days versus 7.3 days (p = 0.021). Mortality was higher in patients with hypokalemia but the difference was not significant 59.6% versus 47.9% (p = 0.108). The use of diuretics and corticosteroids was comparable between two groups.

**Conclusion:** Hypokalemia in patients with covid-19 admitted to the ICU is associated with prolonged duration of mechanical ventilation and ICU stay but does not influence mortality.

**Compliance with ethics regulations:** Yes in clinical research.

### FC-023 Hyponatremia in critically ill COVID-19 patients

#### HADDED Amina^1^, KHARRAT Sana^1^, ALILA Ilef^1^, AMMAR Rania^1^, BEN KHALIFA Atraa^1^, BAHLOUL Mabrouk^1^, BOUAZIZ Mounir^1^

##### ^1^hopital Habib Bourguiba Sfax, Sfax, Tunisie

**Correspondence:** Ilef ALILA (ilefalila1323@gmail.com)

*Annals of Intensive Care* 2022, **12(1):**FC-023

**Rationale:** Dysnatremia is common in patients hospitalized in the intensive care units, often worsening the prognosis of lung disease and severe illness. The main objective of this study is to assess the prevalence of hyponatremia in patients with COVID-19 admitted to the IC U and to evaluate the impact of hyponatremia on severity and outcomes in COVID-19 patients.

**Patients and Methods/Materials and methods:** We conducted a retrospective study including all COVID-19 patients hospitalized in an intensive care unit during a period of 16 months. We compared two groups of patients: the first (G1) group of patients with hyponatremia and the second (G2) group of patients with normal natremia.

**Results:** A total of 126 patients (21.5%) developed hyponatremia in our study. There were more male patients in hyponatremia group 91 (72.2%) versus 269(58.5%) (p = 0.005). The average age was comparable between the two groups (59.1 year in G1 versus 60.9 year in G2; p = 0.24). Pulmonary lesions on chest CT performed during hospitalization were significantly more extensive in the first group (p = 0.023). The prevalence of renal failure was higher in the hyponatremia group with a significant difference (49.2% versus 38.5%; p = 0.03). Regarding the use of invasive ventilation, the two groups were comparable (p = 0.96) and there was no significant difference in terms of duration of mechanical ventilation (p = 0.6). Hyponatremia was a risk factor for prolonged stay in intensive care units 8.9 days i versus 7.1 days (p = 0.03). Mortality was higher in the hyponatremia group but the difference was not significant 52.4% versus 48% (p = 0.38).

**Conclusion:** Hyponatremia had poor outcome in our study. It was associated with more extensive parenchymal damage, an increased prevalence of renal failure and a longer stay in ICU.

**Compliance with ethics regulations:** Yes in clinical research.

### FC-024 Incidence, characteristics, and long-term outcomes of new-onset atrial fibrillation in a medical intensive care unit

#### DOYEN Denis^1^, LABBAOUI Mohamed^1^, SQUARA Fabien^1^, HYVERNAT Hervé^1^, BERNARDIN Gilles^1^, JOZWIAK Mathieu^1^, DELLAMONICA Jean^1^

##### ^1^Centre Hospitalier Universitaire de Nice, Nice, France

###### Correspondence: Denis DOYEN (doyen.d@chu-nice.fr)

*Annals of Intensive Care* 2022, **12(1):**FC-024

**Rationale:** Atrial fibrillation is the most common acquired arrhythmia in the intensive care unit (ICU) and is associated with increased morbidity and mortality. However, patient characteristics, particularly echocardiographic features of new-onset atrial fibrillation (New-AF), are scarce. In addition, little is known about the long-term outcomes of patients with New-AF.

**Patients and methods/Materials and methods:** We conducted a single-center prospective observational study in a medical intensive care unit. All consecutive patients admitted were included. Continuous electrocardiogram monitoring was checked daily for New-AF. Patients were divided into the following groups: New-AF patients, patients with a history of AF, and No-AF patients when no AF was previously diagnosed or documented during hospitalization. Transthoracic echocardiography, electrocardiogram, and blood analysis were performed at admission and in case of New-AF. The following outcomes were collected 6 months after ICU discharge: re-hospitalizations, strokes or other thromboembolism, and mortality.

**Results:** Over a 5-month period, 110 patients were included: 21% (23) New-AF patients, 13% (14) with a history of AF, and 66% (73) No-AF patients. New-AF patients were predominantly male (61%), were significantly older (70 versus 58 years, p < 0.01), and had more comorbidities than patients without AF. Patients with New-AF had higher levels of high-sensitivity troponin I, B-type natriuretic peptide and protein C reactive than No-AF patients (respectively 73 vs 18 ng/L, 408 vs 55 pg/L, and 114 vs 32 mg/L; p < 0.01). At the time of AF onset in the New-AF group, kalemia was lower than at admission (3.5 vs 4.4 mmol/L, p < 0.01). In New-AF patients, TTE showed more left ventricular systolic and diastolic dysfunction, right ventricular systolic dysfunction, pulmonary hypertension and more enlarged left atrial and left ventricular index volumes than in No-AF patients. New-AF patients had more acute pulmonary edema and cardiogenic shock than No-AF patients (respectively 48% versus 6% and 26% versus 4%; p < 0.01). New-AF was significantly associated with greater use of inotropic or vasopressor drugs, mechanical ventilation, and renal replacement, and had higher in-hospital mortality (44% vs 12%, p < 0.01) than No-AF patients. During follow-up, there was no difference in re-hospitalizations, thromboembolism, or mortality between all groups (42% versus 36%, 8% versus 2%, and 15% versus 10%, respectively, between patients with AF and No-AF patients; p > 0.05).

**Conclusion:** In a medical intensive care unit, the incidence of New-AF was 21%. New-AF patients had more echocardiographic abnormalities, organ failure, and higher in-hospital mortality than No-AF patients. No difference in outcomes was observed 6 months after ICU discharge between all groups.

**Compliance with ethics regulations:** Yes in clinical research.

### FC-025 Lactate/Pyruvate ratio as a marker of tissue hypoxia in cardiac surgery

#### KOLSI Hichem^1^, JAWADI Wael^1^, KAMMOUN Anas^1^, SELLAMI Bouthaina^1^, FOURATI Mahdi^1^, KETATA Salma^1^, CHEIKHROUHOU Hichem^1^, TRIKI Zied^1^, ZOUARI Dhouha^1^, JALLOULI Dana^1^, NAIFAR Manel^1^, AYADI Fatma^1^

##### ^1^Centre hospitalo-universitaire Habib Bourguiba, Sfax, Sfax, Tunisie

###### Correspondence: Hichem KOLSI (hichem.kolsi17@gmail.com)

*Annals of Intensive Care* 2022, **12(1):**FC-025

**Rationale:** Cardiac surgery with cardiopulmonary bypass (CPB) has a high risk to induce tissue hypoperfusion and oxygenation impairment. This can lead to complications and organ failure. Blood lactate elevation is a classic marker of anaerobic metabolism and hypoperfusion. But lactate increasing can result from aerobic glycolysis acceleration or impaired clearance. Lactate/Pyruvate ratio (L/P) was studied in septic shock and seems to have a better specificity of anaerobic metabolism. The aim of this study is to assess whether the L/P is associated with adverse outcome after cardiac surgery.

**Patients and methods/Materials and methods:** This is a prospective, observational, monocentric clinical study being performed. We included 26 patients over 18 years undergoing cardiac surgery with CPB, from October 2021. We have an objective of 80 patients. We did not include patients with severe hepatocellular impairment, preoperative hemodynamic instability, or severe renal failure. We have excluded patients who have died during surgery, patients who can only leave the operating room with a circulatory assistance technique. Arterial blood lactate and pyruvate were collected, lactate in sodium fluoride tube, pyruvate in perchloric acid tube. The samples were transported at 4 °C, and then analyzed by enzymatic colorimetric reaction for lactate and spectrophotometry for pyruvate. We have analyzed receiving operating curve (ROC) of L/P for complications occurring. We tested then the correlation of the variables with prognosis interest to L/P by Spearman test.

**Results:** Complications rate was 46.2%. Mortality rate was 26.9%. Mean age was 61.96 ± 9.34. Median Euroscore II was 1.5 (1.09; 2.13). 23 patients have lactate level > 2 mmol/l. Only 18 (78.26%) of these patients have L/P > 16 (a cutoff used previously in septic shock). L/P was predictive of complications with area under ROC 0.857, higher than area under ROC of lactate which was 0.777. Best threshold was 20.34. LP median was significantly higher in group with complications and in non survivor group. We found that L/P is correlated to Euroscore II, duration of CPB and SAPSII at one and two days postoperative.

**Conclusion:** We conclude that L/P is associated with poor outcome after cardiac surgery. L/P is more efficient than lactate to predict postoperative complications. L/P could be used as an additional tool to identify patients with high risk of adverse outcome after cardiac surgery. Nevertheless, these results should be confirmed in larger multicentric studies. A randomized trial comparing a standard therapy strategy with a strategy based on L/P is needed to allow clinicians to make a therapy decision using LP.

**Compliance with ethics regulations:** Yes in clinical research.

### FC-026 Phenotypic heterogeneity of covid-19-related myocarditis in adults

#### BARHOUM Petra^1^, PINETON DE CHAMBRUN Marc^1^, DORGHAM Karim^1^, KERNEIS Mathieu^1^, BURREL Sonia^1^, QUENTRIC Paul^1^, PARIZOT Christophe^1^, CHOMMELOUX Juliette^1^, BRECHOT Nicolas^1^, LEBRETON Guillaume^1^, BOUSSOUAR Samia^1^, SCHMIDT Matthieu^1^, YSSEL Hans^1^, LEFEVRE Lucie^1^, MIYARA Makoto^1^, CHARUEL Jean-Luc^1^, MAROT Stephane^1^, LUYT Charles-Edouard^1^, LEPRINCE Pascal^1^, AMOURA Zahir^1^, MONTALESCOT Gilles^1^, REDHEUIL Alban^1^, COMBES Alain^1^, GOROCHOV Guy^1^, HEKIMIAN Guillaume^1^

##### ^1^GROUPE HOSPITALIER PITIE SALPETRIERE, Paris, France

###### Correspondence: Petra BARHOUM (pbarhoum@gmail.com)

*Annals of Intensive Care* 2022, **12(1):**FC-026

**Rationale:** Adults who have been infected with SARS-CoV-2 can develop a multisystem inflammatory syndrome (MIS-A), including myocarditis. Yet, several patients fail to meet MIS-A criteria, suggesting the existence of distinct phenotypes of COVID-19 related myocarditis. The objective of this study was to compare the characteristics and clinical outcome between patients with COVID-19-related myocarditis fulfilling MIS-A criteria (MIS-A+) or not (MIS-A−).

**Patients and methods/Materials and methods:** We retrospectively reviewed the prospectively constituted database of our 26-bed ICU between March 2020 and June 2021, and included all patients admitted for suspected myocarditis with proven SARS-CoV-2 infection, without vaccination against COVID-19. The primary endpoint was in-hospital mortality. The secondary outcomes included left ventricle ejection fraction (LVEF) evolution, and long-term survival.

**Results:** Between March 2020 and June 2021, 38 patients required ICU admission (male: 66%; mean age: 32 ± 15 years) for suspected COVID-19-related myocarditis. All had positive SARS-CoV-2 RT-PCR (37%) or serology (68%). In-ICU treatment for organ failures included dobutamine (79%), norepinephrine (60%), mechanical ventilation (50%), VA-ECMO (42%) and renal replacement therapy (29%). In-hospital mortality was 13%. Twenty-five (66%) patients met the MIS-A criteria. MIS-A− compared to MIS-A+ patients were characterized by a shorter time lapse between the first COVID-19 symptoms and myocarditis (3 vs. 8 days, p = 0.04), a lower left ventricle ejection fraction (LVEF 10 vs. 30%, p = 0.01; LVOT-VTI 5 vs.13 cm, p < 0.0001), as well as higher in-ICU organ failure, need for mechanical circulatory support with VA-ECMO (92% vs.16%, p < 0.0001), and in-hospital mortality (31% vs. 4%, p = 0.04). The main differences between the phenotypes of MIS-A+ and MIS-A− patients are summarized in the Central Illustration Median LVEF at ICU and hospital discharge was 42% [30–54] and 60% [50–64] respectively. At the last follow-up (median [IQR] 235 [155–359] days), 32 patients were alive, all but one with normal LVEF, and one was lost of follow-up. The main differences between the phenotypes of MIS-A+ and MIS-A− patients are summarized in the Central Illustration.

**Conclusion:** MIS-A+ and MIS-A− COVID-19-related myocarditis patients have two distinct phenotypes in term of clinical presentation, timing, prognosis, and immunological profile. This study differentiated for the first time these two phenotypes, and it seems relevant for patients’ management and further understanding of their pathophysiology.

**Compliance with ethics regulations:** Yes in clinical research.
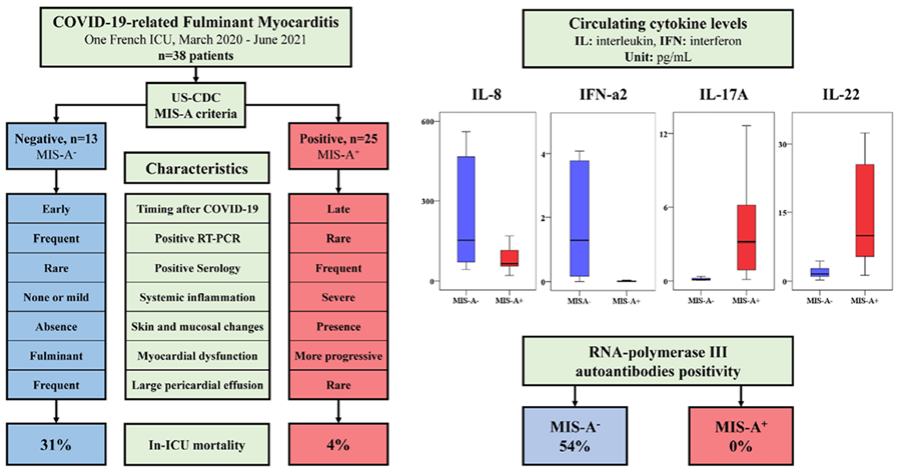


*Main phenotypic differences between (MIS-A*+*) and (MIS-A−) COVID-19-related myocarditis.*

### FC-027 Impaired microvascular endothelial reactivity in critically ill COVID-19 patients

#### RAIA Lisa^1^, URBINA Tomas^1^, GABARRE Paul^1^, BONNY Vincent^1^, HARIRI Geoffroy^1^, ERHMINGER Sébastien^1^, BIGÉ Naïke^1^, BAUDEL Jean-Luc^1^, GUIDET Bertrand^1^, MAURY Eric^1^, JOFFRE Jérémie^1^, AIT-OUFELLA Hafid^1^

##### ^1^Hôpital Saint Antoine, Paris, France

###### Correspondence: Lisa RAIA (lr.lisa.raia@gmail.com)

*Annals of Intensive Care* 2022, **12(1):**FC-027

**Rationale:** The COVID-19 outbreak, caused by the Severe Acute Respiratory Syndrome coronavirus 2 (SARS-CoV2), has affected unprecedently all regions of the world. Critically ill COVID-19 patients exhibit inflammatory syndrome and frequent arterial and venous thrombosis affecting large vessels and microcirculation. Plus, clinical and histological studies have reported that SARS-CoV2 infection may damage the endothelium. However, the impact of this virus on endothelial function in vivo remains poorly characterized. This prospective observational study aimed to compare skin microvascular endothelial reactivity in critically-ill COVID-19 to patients admitted in the ICU for non-COVID-19 bacterial pneumonia (NCBP).

**Patients and methods/Materials and methods:** We conducted a prospective observational study in an 18-bed ICU. We included consecutive COVID-19 adult patients and patients with non-COVID-19 bacterial pneumonia admitted to our ICU for acute respiratory failure. Microvascular endothelial reactivity was assessed in the forearm skin area within the first 24 h of admission using acetylcholine iontophoresis coupled with Laser doppler. This non-invasive technique allows transdermal diffusion of acetylcholine across the skin to subcutaneous capillaries which results in endothelium-dependent vasodilatation and increased blood flow. The endothelial reactivity was quantified by the area under the curve (AUC) of the blood flow curve within a standardized 10-min recording. Hemodynamic and tissue perfusion parameters were also recorded.

**Results:** During 3 consecutive months, 32 COVID-19 patients and 11 control NCBP patients with acute respiratory failure were included. The median age was 59 [50–68] and 69 [57–75] years in COVID-19 and NCBP groups, respectively (P = 0.11). No significant difference in comorbidities or medications was observed between the two groups, except for body mass index, which was higher in COVID-19 patients. NCBP patients had higher SAPS II score compared to COVID-19 patients (46 [32–51] vs. 23 [18-30], P < 0.0001) but SOFA score was not different between groups (4 [3–4] vs 4 [2–5.3], P = 0.51). Global hemodynamic and peripheral tissue perfusion parameters were not different between groups. COVID-19 patients had significant lower skin basal blood flow compared to NCBP patients (7.9 [5.5–9.9] vs. 10.4 [9.4–12.1] UI, P = 0.02). In addition, endothelium-dependent microvascular reactivity was threefold lower in COVID-19 patients compared to NCBP patients (AUC 3911 [1725–6318] vs. 14280 [5038–19743], P = 0.008) **(Fig. 1)**

**Conclusion:** In critically ill COVID-19 patients, we evidenced a drastically impaired skin microvascular endothelium-dependent vasoreactivity at the early phase of the disease compared to non-COVID-19 bacterial pneumonia patients. This result supports the hypothesis of a singular and clinically relevant SARS-CoV 2-associated endotheliopathy.

**Compliance with ethics regulations:** Yes in clinical research.
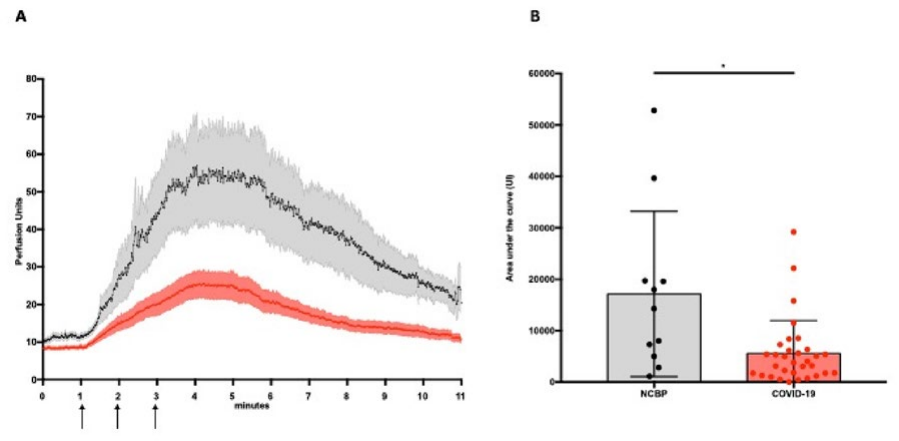


*Figure 1. A. Skin microvascular blood flow in response to three stimulation of acetylcholine (arrows) in NCBP patients (grey) and in COVID-19 patients (red), represented as mean*+*/ SEM every seconds for 10 min after the first electric stimulation. B.*

### FC-028 Venous Thromboembolism events in critically ill COVID-19 patients

#### ALILA Ilef^1^, HADDED Amina^1^, KHARRAT Sana^1^, CHTARA Kamilia^1^, BAHLOUL Mabrouk^1^, BOUAZIZ Mounir^1^

##### ^1^hopital Habib Bourguiba Sfax, Sfax, Tunisie

###### Correspondence: Ilef ALILA (ilefalila1323@gmail.com)

*Annals of Intensive Care* 2022, **12(1):**FC-028

**Rationale:** There is some evidence that Covid 19 pneumonia is associated with prothrombotic status and increased risk of venous thromboembolic events (1). The aim of this study was to determine the incidence of PE in patients with COVID-19, risk factors and its prediction of outcome in critically ill COVID19 patients.

**Patients and methods/Materials and methods:** We conducted a retrospective study in a medical ICU over a period of 16 months [September 2020–December 2021] including patients with SARS-COV2 infection.

**Results:** During the study period, 586 patients were included with a mean age of 59.5 ± 14.7 and a gender ratio of 1.6. The median SAPSII and SOFA score were respectively of 29 ± 14.8 and 4 ± 2.6. Most patients had comorbidities, including hypertension (36%), obesity (32.1%) and diabetes (36.2%). 509 (87%) patients received therapeutic anticoagulation on admission versus only 77 (13%) received prophylactic anticoagulation. Twenty-eight (4.7%) patients had venous thromboembolic events: PE was diagnosed through CT pulmonary angiography in 22 (3.8%) of 586 hospitalized patients with COVID-19 and deep venous thrombosis was diagnosed in 10 (1.7%) patients through doppler ultrasound of lower limb. Four patients had both complications. The comparison between 2 groups with or without thromboembolic (TE) complications showed that the risk factors usually associated the development of thromboembolic complications were not significantly different (high blood pressure in 7 (25%) vs 204 (36.6%) p = 0.2, diabetes in 9 (32.1%) vs 203 (36.4%) p = 0.8, obesity in 13 (46.4%) vs 175 (31.3%) p = 0.09, smoking in 6 (21.4%) vs 125 (22.4) p = 0.9). Invasive mechanical ventilation was required in 264 (45.1%) patients, 20 (71.4%) in the group with TE complications and 244 (43.7%) in the other group, p = 0.004. Mortality was similar between the 2 groups (15 (53.5%) vs 272 (48.7%); P = 0.6).

**Conclusion:** TE complications were rare in our cohort. These complications are associated with higher use of mechanical ventilation but not with higher mortality.

**Reference 1:** (1) Pompilio Faggiano, Andrea Bonelli, Sara Paris, Giuseppe Milesi, Stefano Bisegna, Nicola Bernardi, Antonio Curnis, Eustachio Agricola, Roberto Maroldi: Acute pulmonary embolism in COVID-19 disease: Preliminary report on seven patients.

**Compliance with ethics regulations:** Yes in clinical research.

### FC-029 Anti-RNA-polymerase III autoantibodies-associated fulminant myocarditis

#### PINETON DE CHAMBRUN Marc^1^, CHARUEL Jean-Luc^1^, DORGHAM Karim^1^, QUENTRIC Paul^1^, KERNEIS Mathieu^1^, LEBRETON Guillaume^1^, MIYARA Makoto^1^, SCHMIDT Matthieu^1^, LUYT Charles-Edouard^1^, LIFERMANN Francois^2^, MELKI Isabelle^3^, AMOURA Zahir^1^, GOROCHOV Guy^1^, HEKIMIAN Guillaume^1^, COMBES Alain^1^

##### ^1^Hôpital La Pitié-Salpêtrière, Paris, France; ^2^CH DAX, Dax, France; ^3^Hôpital Robert Debré, Paris, France

###### Correspondence: Marc PINETON DE CHAMBRUN (marc.dechambrun@gmail.com)

*Annals of Intensive Care* 2022, **12(1):**FC-029

**Rationale:** Anti-RNA-polymerase III autoantibodies (RNApol3)-associated fulminant myocarditis is a recently discovered entity associating recurrent viral (mostly influenzae)-induced fulminant myocarditis and/or severe pericarditis in patients with RNApol3 but no overt systemic sclerosis. It is a serious condition owing the severity of myocarditis episodes and the risk of relapse. The pathophysiology of the disease is unknown. We conducted this study to better delineate the clinical characteristics and the outcome of these patients.

**Patients and methods/Materials and methods:** Retrospective, monocenter study between January 2013 and January 2022 including every patients admitted to a 26-bed intensive care unit for myocarditis and/or severe pericarditis in the presence of RNApol3.

**Results:** Twenty-five patients (women 80%, mean age at first episode 35 ± 11.4 years) were included in the study. Seven (28%) patients died after a median follow-up 39 [6–50] months: during a first myocarditis episode (n = 2), a relapse (n = 4) or from other cause (n = 1). The mean number of episodes per patients was 1.6 ± 0.9 and 40% patients had at least one relapse. Every patient was admitted at least once in critical care for a median duration of 9 [5–14] days. The lowest left ventricle ejection fraction value was 5 [5–10] % and the highest troponin value 82 [19–370] fold over ULN. Pericardial effusion was reported in 94% cases, requiring drainage in 40% cases. Inflammatory parameters were mildly elevated: C-reactive protein 7 [5–14] mg/L, procalcitonin 0.1 [0.06–0.4] ng/mL and fibrinogen 3 [2.4–3.4] g/L. Conduction and rhythm disorders were infrequent: 3 and 7% respectively. In-ICU organ-failure treatment frequencies were: dobutamine 83%, VA-ECMO 77%, vasopressors 70%, mechanical ventilation 67% and renal replacement therapy 30%. Two patients received cardiac transplantation because of unrecovering cardiac failure while all survivors could be weaned from ECMO and recovered normal cardiac function at distant follow-up. The etiology of myocarditis were: influenza 52%, COVID-19 44%, unknown viral infection 12% and other virus 4%. Four patients had a myocarditis episode on the occasion of both influenzae virus infection and COVID-19. RNApol3 were confirmed on distant follow-up in every patient (n = 17) after a mean duration of 8 [2.5–16.5] months. Two patients only had systemic sclerosis classification score ≥ 9 without visceral involvement.

**Conclusion:** RNApol3-associated fulminant myocarditis is a new severe entity. Our study shows that both influenza and COVID-19 are responsible for myocarditis in these patients. A significant number of patients have been diagnosed on the occasion of COVID-19 pandemic. The pathophysiology of this disease needs further investigation.

**Compliance with ethics regulations:** Yes in clinical research.

### FC-030 Safety, diagnostic yield and therapeutical consequences of myocardial biopsy in unexplained acute heart failure requiring extracorporeal life support

#### PINETON DE CHAMBRUN Marc^1^, YANN Marquet^1^, ROUVIER Philippe^1^, KERNEIS Mathieu^1^, BRECHOT Nicolas^1^, SCHMIDT Matthieu^1^, CHOMMELOUX Juliette^1^, MOYON Quentin^1^, BARHOUM Petra^1^, LEFEVRE Lucie^1^, SAURA Ouriel^1^, LEVY David^1^, ASSOULINE Benjamin^1^, LUYT Charles-Edouard^1^, COMBES Alain^1^, HEKIMIAN Guillaume^1^

##### ^1^Hôpital La Pitié-Salpêtrière, Paris, France

###### Correspondence: Marc PINETON DE CHAMBRUN (marc.dechambrun@gmail.com)

*Annals of Intensive Care* 2022, **12(1):**FC-030

**Rationale:** Myocardial biopsy is strongly recommended in patients with unexplained acute heart failure, especially in case of cardiogenic shock, despite a very low level of evidence. The safety, the diagnostic yield and the therapeutical consequences of myocardial biopsy in patients requiring extracorporeal life support (ECLS) is poorly investigated.

**Patients and methods/Materials and methods:** Retrospective, monocenter study between January 2002 and January 2018 including every patients admitted to a 26-bed intensive care unit undergoing a myocardial biopsy (surgical or endomyocardial) for unexplained acute heart failure while being under ECLS. All patients had a myocardial biopsy altogether with a comprehensive noninvasive diagnosis work-up. The primary endpoint was the rate of therapeutical modifications as a direct consequence of myocardial biopsy results.

**Results:** Forty-seven patients (women 53%, mean age at admission 39 ± 11 years) were included in the study. Forty-two (89%) received veno-arterial extracorporeal membrane oxygenation (VA-ECMO), requiring centralization in 26 (62%) and 15 (32%) were given ventricle assist device. Twenty-six (55%) patients died in hospital, 16 (34%) could be weaned from ECLS and 7 (15%) required cardiac transplantation. According the Bonaca myocarditis classification, 75% had definite and 25% probable myocarditis. Biopsy was endomyocardial in 17 (36%) patients and surgical in other cases. Organ failure treatments on biopsy-day were: ECLS 100%, inotropes/vasopressors 96%, mechanical ventilation 75% and renal replacement therapy 30%. Endomyocardial and surgical biopsy was followed by tamponade in 29% and 10% cases respectively. One patient undergoing endomyocardial biopsy died as a direct consequence of the procedure. The biopsy-based and the noninvasive heart failure mechanism diagnosis work-up resulted in the following diagnosis respectively: myocarditis 51 and 13%; none 45 and 57% and alternated diagnosis 4 and 30%. The biopsy-based and the noninvasive etiology diagnosis work-up led to a diagnosis in 25 and 47% cases respectively. The biopsy-based and the noninvasive diagnosis work-up results in a therapeutical modification in 13 and 19% patients respectively (Fig. 1). Among the 6 patients whose biopsy led to a treatment change, 2 survived to hospital discharge, including one whose etiological diagnosis was also given by the noninvasive work-up.

**Conclusion:** Myocardial biopsy in patients with acute heart failure of unknown etiology requiring ECLS has low diagnostic yield and therapeutical consequences and is associated with significant adverse events. The benefit/risk ratio of this procedure should be carefully weighted in these patients.

**Compliance with ethics regulations:** Yes in clinical research.
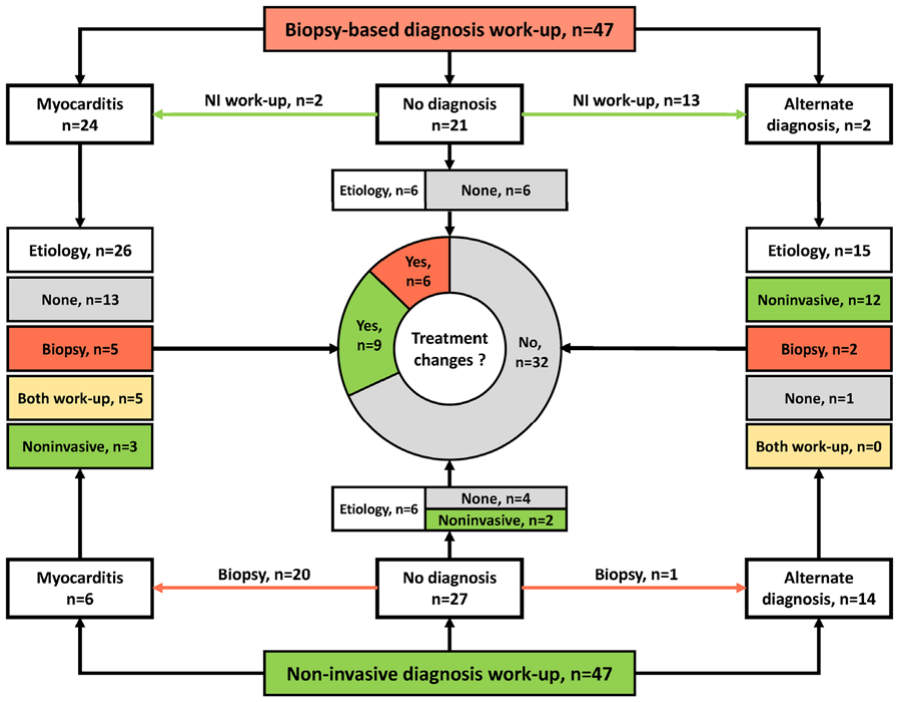


*Figure 1. Diagnosis and therapeutic consequences according biopsy-based or noninvasive diagnosis work-up*

### FC-031 Stent thrombosis in cardiac arrest patients: prevalence, risk factors and long-term outcome

#### BOIS Antoine^1^

##### ^1^Hôpital Henri Mondor, Villejuif, France

###### Correspondence: Antoine BOIS (antoinebois01@gmail.com)

*Annals of Intensive Care* 2022, **12(1):**FC-031

**Rationale:** Ischemic heart disease is the leading cause of cardiac arrest (CA) and stent thrombosis (ST) is ten times more frequent when acute coronary syndrome is initially associated with CA. Data are missing about impact and risk factors of this complication.

**Patients and Methods/Materials and Methods:** We conducted a monocenter and retrospective study from January 2010 to September 2019 including all patients hospitalized after a CA who were treated by stent andhad a second coronary angiography in the 3 following months. Demographics and characteristics of patient care were compared according to ST. Long-term follow-up was provided using the French National Healthcare Insurance database.

**Results:** 22 patients met the inclusion criteria during the study period. The incidence of ST was 50%. Stent length was associated with ST occurrence (26.8 mm vs 17.0 mm, p = 0.023), whereas there was no significant difference on age, gender, coronary lesion features, type of stent or drugs used. Long-term follow-up of 7 patients, including 6 cases of ST, revealed no major adverse cardiac event.

**Conclusion:** Stent thrombosis is a frequent complication after CA and appears to be associated to stent’s length. Larger prospective studies, are needed to define the prognosis and risk factors of ST.

**Compliance with ethics regulations:** Yes in clinical research.
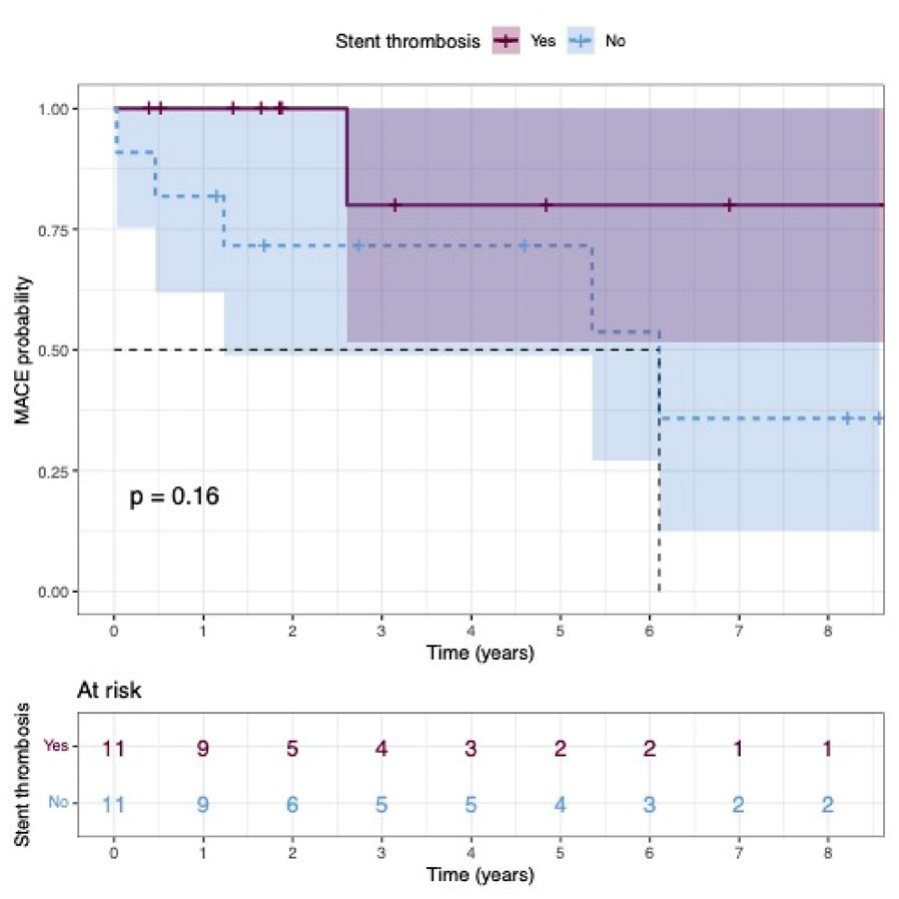


*Kaplan–Meier plot of probability of MACE occurrence according to the presence of ST*

### FC-032 SSEP N20 and P25 amplitudes predict poor and good neurologic outcomes after cardiac arrest

#### BENGHANEM Sarah^1,2^, NGUYEN Lee Son^1^, GAVARET Martine^2^, MIRA Jean Paul^1^, PENE Frederic^1^, CHARPENTIER Julien^1^, MARCHI Angela^2^, CARIOU Alain^1^

##### ^1^Hôpital Cochin APHP, Paris, France; ^2^Unité INSERM 1266 Institut de Psychiatrie et Neurosciences Paris IPNP, Hopital Sainte Anne, Paris, France

###### Correspondence: Sarah BENGHANEM (sarah.benghanem@aphp.fr)

*Annals of Intensive Care* 2022, **12(1):**FC-032

**Rationale:** Background: To assess in comatose patients after cardiac arrest (CA) if amplitudes of two somatosensory evoked potentials (SSEP) responses, namely N20-baseline (N20-b) and N20-P25, are predictive of neurological outcome.

**Patients and methods/Materials and Methods:** Methods: Monocenterand prospective study in a tertiary cardiac center between November 2019 and July 2021. All patients comatose at 72 h after CA with at least one SSEP recorded were included. The N20-b and N20-P25 amplitudes were automatically measured in microvolts (µV), along with other recommended prognostic markers (status myoclonus, neuron-specific enolase levels at 2 and 3 days, and EEG pattern). We assessed the predictive value of SSEP for neurologic outcome using the best Cerebral Performance Categories (CPC 1 or 2 as good outcome) at 3 months (main endpoint) and 6 months (secondary endpoint). Specificity and sensitivity of different thresholds of SSEP amplitudes, alone or in combination with other prognostic markers, were calculated.

**Results:** Results: Among 82 patients, a poor outcome (CPC 3-5) was observed in 78% of patients at 3 months. The median time to SSEP recording was 3(2–4) days after CA, with a pattern “bilaterally absent” in 19 patients, “unilaterally present” in 4, and “bilaterally present” in 59 patients. The median N20-b amplitudes were different between patients with poor and good outcomes, i.e., 0.93 [0–2.05] µV vs 1.56 [1.24–2.75] µV respectively (p < 0.0001), as the median N20-P25 amplitudes (0.57 [0–1.43] µV in poor outcome vs 2.64 [1.39–3.80] µV in good outcome patients p < 0.0001). A N20-b > 2 µV predicted good outcome with a specificity of 73% and a moderate sensitivity of 39%, although a N20-P25 > 3.2 µV was 93% specific and only 30% sensitive. A low voltage N20-b < 0.88 µV and N20-P25 < 1 µV predicted poor outcome with a high specificity (specificity of 94% and 93% respectively) and a moderate sensitivity (sensitivity of 50% and 66% respectively). Association of “bilaterally absent or low voltage SSEP” patterns increased the sensitivity significantly as compared to “bilaterally absent” SSEP alone (58 vs 30%, p = 0.002) for prediction of poor outcome.

**Conclusion:** Conclusion: In comatose patient after CA, both N20-b and N20-P25 amplitudes could predict both good and poor outcomes with high specificity but low sensitivity. Our results suggest that SSEP amplitudes are inversely related to the severity of neurological injury, although this indicator should be combined with other indicators in a multimodal approach for prognostication.

**Reference 1:** Nolan JP, Sandroni C, Böttiger BW, Cariou A, Cronberg T, Friberg H, et al. European Resuscitation Council and European Society of Intensive Care Medicine guidelines 2021: post-resuscitation care. Intensive Care Med 2021;47:369–421.

**Reference 2:** Scarpino M, Lolli F, Lanzo G, Carrai R, Spalletti M, Valzania F, et al. SSEP amplitude accurately predicts both good and poor neurological outcome early after cardiac arrest; a post-hoc analysis of the ProNeCA multicentre study. Resuscitation 2021;163:162

**Compliance with ethics regulations:** Yes in clinical research.
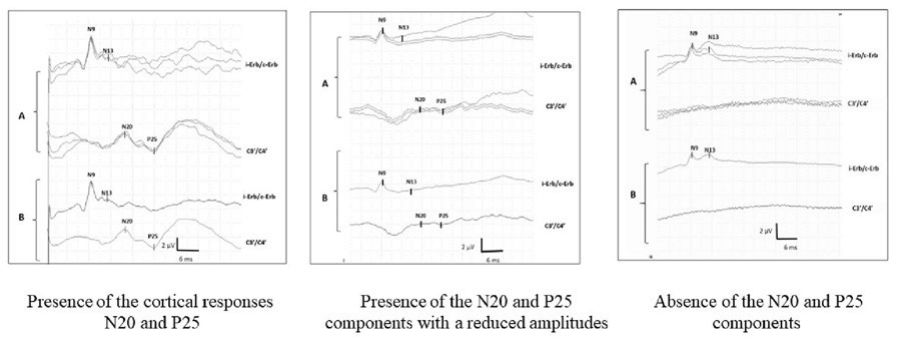


*In a normal SSEP (panel 1), the channels show the cortical responses N20 and P25, the spinal component (N13) and the peripheral component (N9). In the panel 2, N20 and P25 were presented but amplitudes were reduced. In the panel 3, N20 and P25 were absent*

### FC-033 Head-up position reduces intra-cranial pressure during extracorporeal resuscitation following refractory cardiac arrest in swine

#### LEVY Yael^1^, HUTIN Alice^2^, POLGE Nicolas^3^, LIDOUREN Fanny^3^, FERNANDEZ Rocio^3^, KOHLHAUER Matthias^3^, LEGER Pierre-Louis^1^, DEBATY Guillaume^4^, LURIE Keith^5^, LAMHAUT Lionel^2^, GHALEH Bijan^3^, TISSIER Renaud^3^

##### ^1^CHU Armand Trousseau, Paris, France; ^2^CHU Necker, Paris, France; ^3^Ecole Nationale Vétérinaire d’Alfort, Maison-Alfort, France; ^4^CHU de Grenoble, Grenoble, France; ^5^Hennepin Healthcare Research Institute, Minneapolis, Etats-Unis

###### Correspondence: Yael LEVY (yael.levy@aphp.fr)

*Annals of Intensive Care* 2022, **12(1):**FC-033

**Rationale:** Head-up position was shown to mitigate cerebral alterations and improve cerebral perfusion pressure during conventional cardiopulmonary resuscitation. Since extracorporeal cardiopulmonary resuscitation (E-CPR) is more and more used for the management of refractory cardiac arrest, we aimed at determining whether head-up position could also modify cerebral hemodynamics during E-CPR. Accordingly, we compared this hemodynamics in swine submitted to E-CPR in flat position as compared to head position (head-up position at 30 °C). Hypothesis: Our goal was to determine whether a standardized head-up could improve intracranial pressure (ICP) and cerebral autoregulation during E-CPR in a swine model of cardiac arrest.

**Patients and methods/Materials and methods:** Pigs were anesthetized and instrumented for the continuous evaluation of carotid blood flow, intracranial pressure (ICP), cerebral perfusion pressure, pressure reactivity index (PRx) and systemic hemodynamics. They were submitted to 15 min of untreated ventricular fibrillation followed by 30 min of E-CPR. Defibrillations were then delivered until resumption of spontaneous circulation (ROSC). Extracorporeal circulation was initially set to an average flow of 40 ml/kg/min. Epinephrine was delivered to achieve a mean arterial pressure ≥ 65 mmHg. An automated head-up position device (over two minutes, 30°) was used in head-up group. Animals were followed during 120-min after ROSC.

**Results:** Six animals were included in both groups. Cerebral hemodynamic parameters are illustrated in Figure. During E-CPR and ROSC, ICP decreased in head-up vs flat position and cerebral perfusion pressure tended to be improved at the end of the follow-up (NS). Cerebral oxygen saturation, carotid blood flow and PRx, which is the correlation coefficient between arterial blood pressure and ICP, were not different among groups.

**Conclusion:** During E-CPR, head-up is associated with lower ICP with a trend toward higher cerebral perfusion pressure. This study supports the need for further investigations to confirm that the early decrease in ICP could be associated with an improvement in neurological outcome after E-CPR.

**Compliance with ethics regulations:** Yes in animal testing.
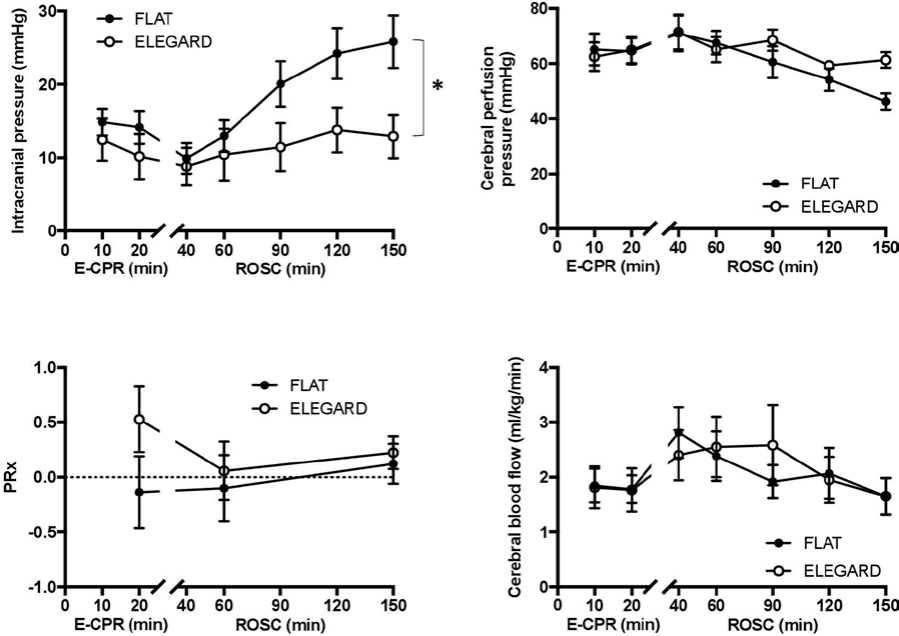


*Cerebral hemodynamic parameters after cardiac arrest*

### FC-034 Targeted temperature management after in-hospital cardiac arrest: an ancillary analysis of hyperion trial data

#### BLANC Alexiane^1^, COLIN Gwenhaël^2^, CARIOU Alain^3,4^, MERDJI Hamid^5,6^, GRILLET Guillaume^7^, GIRARDIE Patrick^8^, COUPEZ Elisabeth^9^, DEQUIN Pierre-François^10,11^, BOULAIN Thierry^12^, FRAT Jean-Pierre^13,14,15^, ASFAR Pierre^16^, PICHON Nicolas^17,18^, LANDAIS Mickael^19^, PLANTEFEVE Gaëtan^20^, QUENOT Jean-Pierre^21^, CHAKARIAN Jean-Charles^22^, SIRODOT Michel^23^, LEGRIEL Stéphane^24^, MASSART Nicolas^25^, THEVENIN Didier^26^, DESACHY Arnaud^27^, DELAHAYE Arnaud^28^, BOTOC Vlad^29^, VIMEUX Sylvie^30^, MARTINO Frederic^31^, REIGNIER Jean^1^, TACCONE Fabio Silvio^32^, LASCARROU Jean-Baptiste^1,3^

##### ^1^Centre Hospitalier Universitaire de Nantes, Médecine intensive réanimation, Nantes, France; ^2^Centre Hospitalier Départemental de La Roche-sur-Yon, La Roche-Sur-Yon, France; ^3^Centre de recherche cardiovasculaire INSERM U970, Paris, France; ^4^Centre Hospitalier Universitaire de Cochin, Médecine intensive réanimation, Paris, France; ^5^Hôpitaux universitaires de Strasbourg, Nouvel Hôpital Civil, Médecine intensive réanimation, Strasbourg, France; ^6^Fédération de Médecine Translationnelle de Strasbourg UMR 1260, Strasbourg, France; ^7^Centre hospitalier de Bretagne sud Lorient, Médecine intensive réanimation, Lorient, France; ^8^Centre Hospitalier Universitaire de Lille, Médecine intensive réanimation, Lille, France; ^9^Centre Hospitalier Universitaire de Clermont-Ferrand, Médecine intensive réanimation, Clermont-Ferrand, France; ^10^Centre Hospitalier Universitaire de Tours, Médecine intensive réanimation, Tours, France; ^11^Centre d’Étude des Pathologies Respiratoires, Université de Tours, Tours, France; ^12^Centre Hospitalier Régional d'Orléans, Médecine intensive réanimation, Orléans, France; ^13^Centre Hospitalier Universitaire de Poitiers, Médecine intensive réanimation, Poitiers, France; ^14^INSERM, CIC-1402, Poitiers, France; ^15^Faculté de Médecine et de Pharmacie de l'Université de Poitiers, Poitiers, France; ^16^Centre Hospitalier Universitaire d'Angers, Médecine intensive réanimation, Angers, France; ^17^Centre Hospitalier Universitaire de Limoges, Service de Réanimation Polyvalente, Limoges, France; ^18^Centre Hospitalier Universitaire de Limoges, CIC 1435, Limoges, France; ^19^Centre Hospitalier du Mans, Service de réanimation, Le Mans, France; ^20^Centre Hospitalier d'Argenteuil, Service de réanimation, Argenteuil, France; ^21^Centre Hospitalier Universitaire de Dijon, Médecine intensive réanimation, Dijon, France; ^22^Centre Hospitalier de Roanne, Service de réanimation, Roanne, France; ^23^Centre Hospitalier d'Annecy, Service de réanimation, Annecy, France; ^24^Centre Hospitalier de Versailles, Service de réanimation, Versailles, France; ^25^Centre Hospitalier de Saint Brieuc, Service de réanimation, Saint Brieuc, France; ^26^Centre Hospitalier de Lens, Service de réanimation, Lens, France; ^27^Centre Hospitalier d'Angoulême, Service de réanimation, Angoulême, France; ^28^Centre Hospitalier de Rodez, Service de réanimation, Rodez, France; ^29^Centre Hospitalier de Saint Malo, Service de réanimation, Saint Malo, France; ^30^Centre Hospitalier de Montauban, Service de réanimation, Montauban, France; ^31^Centre Hospitalier Universitaire de Pointe-à-Pitre, Médecine intensive réanimation, Pointe-À-Pitre, France; ^32^Centre Hospitalier Universitaire Erasmus, Université de Bruxelles, Bruxelles, Belgique

###### Correspondence: Alexiane BLANC (alexiane.blanc@hotmail.fr)

*Annals of Intensive Care* 2022, **12(1):**FC-034

**Rationale:** Targeted temperature management (TTM) is currently the only treatment with demonstrated efficacy in attenuating the harmful effects on the brain of ischemia-reperfusion injury after cardiac arrest. However, whether TTM is beneficial in the subset of patients with in-hospital cardiac arrest remains unclear. Is hypothermia at 33 °C associated with better neurological outcomes after in-hospital cardiac arrest in a non-shockable rhythm, compared to targeted normothermia at 37 °C?

**Patients and methods/Materials and methods:** We performed a post hoc analysis of data from the published Targeted Temperature Management for Cardiac Arrest with Nonshockable Rhythm (HYPERION) randomized controlled trial in 584 patients. We included the 159 patients with in-hospital cardiac arrest; 73 were randomized to 33 °C and 86 to 37 °C. The primary outcome was survival with a good neurological outcome (Cerebral Performance Category [CPC] score 1 or 2) at day 90. Mixed multivariable adjusted logistic regression analysis was performed to determine whether survival with CPC 1 or 2 on day 90 was associated with type of temperature management after adjustment on baseline characteristics not balanced by randomization.

**Results:** Compared to targeted normothermia for 48 h, hypothermia at 33 °C for 24 h was associated with a higher percentage of patients who were alive with good neurological outcomes at day 90 (16.4% vs. 5.8%; P = 0.03). Day-90 mortality was not significantly different between the two groups (68.5% vs. 76.7%; P = 0.24). By mixed multivariable analysis adjusted on the CAHP score and circulatory shock, hypothermia was significantly associated with good day-90 neurological outcomes (2.40 [1.17;13.03]; P = 0.03).

**Conclusion:** Hypothermia at 33 °C was associated with better day-90 neurological outcomes after in-hospital cardiac arrest in a non-shockable rhythm, compared to targeted normothermia. However, our limited sample size resulted in wide confidence intervals. Further studies of patients after cardiac arrest from any cause, including in-hospital cardiac arrest, are needed.

**Compliance with ethics regulations:** Yes in clinical research.

### FC-035 Impact of Methylene Blue for post resuscitation syndrome in a pig model of refractory cardiac arrest resuscitated with veno-arterial ECMO

#### PEQUIGNOT Benjamin^1^, LESCROART Mickael ^1^, ALBUISSON Eliane^1^, ORLOWSKI Sophie^1^, PINA Héloise^1^, TRAN Nguyen ^1^, GRANDMOUGIN Daniel^1^, LEVY Bruno^1^

##### ^1^CHRU de Nancy, Vandœuvre-Lès-Nancy, France

###### Correspondence: Benjamin PEQUIGNOT (b.pequignot@chru-nancy.fr)

*Annals of Intensive Care* 2022, **12(1):**FC-035

**Rationale:** Selected patients with a refractory cardiac arrest could benefit from veno-arterial extracorporeal membrane oxygenation (VA-ECMO) as rescue with good prognosis. Circulatory flow recovery with ECMO is associated with vasoplegia and vasopressor need. The aim of the present work is to assess the interest of Methylene Blue perfusion in a pig model of ischemic refractory cardiac arrest implanted by VA-ECMO.

**Patients and methods/Materials and methods:** Ischemic refractory cardiac arrest was performed in 20 pigs. After a low flow period of 30 min, VA-ECMO was initiated and pigs were randomly assigned to standard care group (norepinephrine + crystalloids) or methylene blue group (IV methylene blue + standard care). Macrocirculatory and metabolic parameters were assessed by lactate clearance. Microcirculatory parameters were assessed by sublingual microcirculation with Sidestream Dark Field (SDF). Pulmonary oedema was evaluated by measuring lung wet/dry weight ratio. Severity of ischemic digestive lesions were assessed by Chiu/Park scale.

**Results:** 18 pigs were included in statistical analyses. There was no significant difference between the groups with regard to lactate clearance levels (29.16 [12.5–39.32], 46.10 [22.61–64.54] % for control and methylene blue group). There was no significant difference between the groups regarding sublingual capillary microvascular parameters assessed by SDF. Total crystalloid load was significantly reduced with Methylene Blue infusion (5000 [6000–8000] mL vs. 17000 [10000–19000] mL p = 0.007, Methylene Blue versus control group). Methylene blue infusion significantly reduced catecholamine requirements (0.31 [0.14–0.44] μg.kg1.min^−1^ vs. 2.32 [1.17–5.55] μg.kg1.min^−1^, methylene blue versus control group p = 0.004). The was not difference in Chiu/Park scale and Lung wet/dry weight ratio between groups.

**Discussion:** The main result of this study is that in an experimental pig model of refractory cardiac arrest resuscitated with veno-arterial ECMO, the use of methylene blue infusion was not associated with a faster lactate clearance. Importantly, the adjunction of methylene blue was associated with sharp reduction of crystalloid fluid load and catecholamine perfusion. There were also some limitations: no return of spontaneous circulation was achieved for any animal of the experiment. The study time over a 6-h period represents a major limitation as it could be mis-scaled to assess benefits and drawbacks of catecholamine or fluid restriction.

**Conclusion:** Methylene blue infusion was efficient for catecholamine and fluid restriction respectively but failed to improve lactate clearance in a pig model of post resuscitation syndrome after refractory cardiac arrest treated with VA-ECMO.

**Compliance with ethics regulations:** Yes in animal testing.
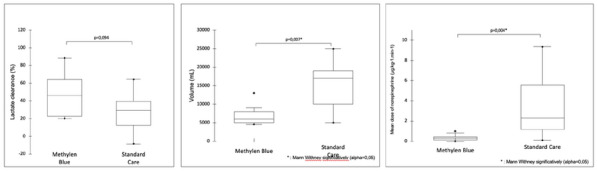


*Lactate clairance, Volume and cathecolamine dose between groups*

### FC-036 Right ventricular changes before and after veno-venous ECMO implantation in refractory COVID-19 ARDS

#### LÉVY David^1^, DESNOS Cyrielle^1,3^, THÉRY Guillaume^1,2^, SCHMIDT Matthieu^1^, BRECHOT Nicolas^1^, PINETON DE CHAMBRUN Marc^1^, CHOMMELOUX Juliette^1^, COMBES Alain^1^, HEKIMIAN Guillaume^1^

##### ^1^Pitié-Salpêtrière, Paris, France; ^2^CHU de Reims, Reims, France; ^3^Hôpital Tenon, Paris, France

###### Correspondence: David LEVY (dlevy88@gmail.com)

*Annals of Intensive Care* 2022, **12(1):**FC-036

**Rationale:** Right ventricular (RV) failure is a common complication during acute respiratory distress syndrome (ARDS). Veno-venous ECMO (VV-ECMO) is considered in refractory ARDS and might be responsible for RV function changes. Our objective was to describe RV function before and after VV-ECMO course in refractory ARDS COVID-19 patients.

**Patients and methods/Materials and methods:** This retrospective observational single-center study was performed between April 2020 and April 2021. Patients included were refractory ARDS secondary to SARS-CoV-2 infection and required VV-ECMO according to EOLIA criteria. Patients underwent serial echocardiographic examinations (Philips, CX-50) before and after VV-ECMO implantation.

**Results:** 15 patients were included. Median age was 54 years (46–62), 9/15 (60%) were male. Before ECMO cannulation, median PaO_2_/FiO_2_ was 60 (56–72) mmHg, delay between ventilation and cannulation was 6 (4–11) days. SAPS-II at cannulation was 46 (30–55). Inhaled nitric oxide and norepinephrine were used in 8/15 patients (53.3%) before ECMO. Fractional area change of RV (FAC-RV) was greater after ECMO implantation, (36 [20–44] % before ECMO, 42 [35–45] % at day 1 and 42 [40–46] % at day 3, p = 0.008). Estimated systolic pulmonary pressure (sPAP) decreased over time (47 [40–55] mmHg before, 40 [36.3–40.1] mmHg at day 1 and 37 [34–45] mmHg at day 3, p = 0.048). PCO_2_ was higher before ECMO implantation (56 [49–63] mmHg versus 44 [39–47] mmHg at day 1 and 41 [39–44] mmHg at day 3, p = 0.001). FAC-RV/sPAP increased after ECMO cannulation (0.68 [0.31–0.97] before vs. 1 [0.74–1.2] at day 1 and 1.1 [0.96–1.3] at day 3, p = 0.027). Tricuspid valve systolic velocity (S’ wave) and TAPSE were similar before and after ECMO implantation (S’wave, 17 [13–20] vs. 15 [12–20] cm/sec at day 1 and 14 [11–26] cm/sec at day 3, p = 0.28; TAPSE, 21 [19–27] mm before ECMO, 21 [17–26.5] mm at day 1 and 23 [19–25] mm at day 3, p = 0.44). Arterial blood lactate level decreased after cannulation (2 [1.6–2] mmol/L before vs. 1.7 [1.5–2.3] mmol/L at day 1 and 1.6 [1.6 1–1.7] mmol/L at day 3, p = 0.006). Also, inotropic score tended to be lower (40.7 [24.5–75.8] before ECMO vs. 4.9 [0–20.8] at day 1 and 0 at day 3, p = 0.05). Median time of VV-ECMO course was 20 (15–41) days and 7/15 (46.6%) were alive at ICU discharge.

**Conclusion:** Based on this small-sample study in refractory ARDS, VV-ECMO could be responsible for RV systolic function improvements while decreasing sPAP, and could provide hemodynamic stability through right ventricular-pulmonary artery coupling.

**Compliance with ethics regulations:** Yes in clinical research.
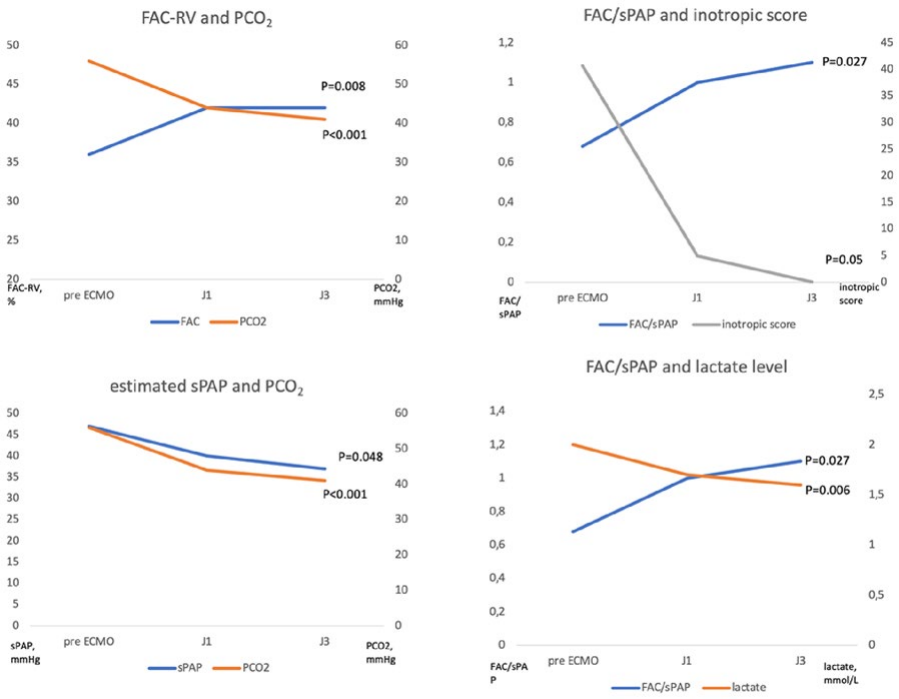


*Right ventricular metrics and hemodynamic changes before and after VV-ECMO implantation*

### FC-037 Interest of albumin infusion for post resuscitation syndrome in a porcine model of refractory cardiac arrest resuscitated with veno-arterial ECMO

#### LESCROART Mickael^1,2^, PEQUIGNOT Benjamin^1,2^, GRANDMOUGIN Daniel^1,2^, TRAN N’Guyen^2^, ALBUISSON Eliane^1^, LEVY Bruno^1,2^

##### ^1^CHRU Nancy, Vandoeuvre, France; ^2^Université de Lorraine, Nancy, France

###### Correspondence: Mickael LESCROART (dr.lescroart@gmail.com)

*Annals of Intensive Care* 2022, **12(1):**FC-037

**Rationale:** Hemodynamic instability is common in post resuscitation syndrome and could worsen survival and neurological outcomes. The myocardial and vascular dysfunction may be exacerbated by veno-arterial extracorporeal membrane oxygenation (VA-ECMO) implanted as a rescue therapy for refractory cardiac arrest. Standard care of haemodynamic management after VA-ECMO initiation are based on norepinephrine and crystalloids. The aim of the present work is to assess the interest on haemodynamics of albumin perfusion in a swine model of ischemic refractory cardiac arrest implanted by VA-ECMO.

**Patients and methods/Materials and methods:** Ischemic refractory cardiac arrest was performed in 20 pigs. After a low flow period of 30 min, VA-ECMO was initiated and pigs were randomly assigned to standard care group (norepinephrine + crystalloids) or albumin group (10 ml/kg of 20% albumin + standard care). Hemodynamical assessment was performed over the 6 h of the experiment

**Results:** 18 pigs were included in statistical analyses. There was no significant difference between groups with regard to lactate clearance (29.16% [12.5–39.32] and 10.09% [6.78–29.36] for control and albumin group respectively). Blood albumin concentration rapidly decreased after ECMO priming in the sham group while it remained stable in the albumin group. Total crystalloid load was significantly reduced with albumin infusion (1000 [1000–2278] mL vs. 17000 [10000–19000] mL, albumin versus control group respectively, p < 0.001).

**Conclusion:** This is the first study assessing albumin for fluid resuscitation in post resuscitation syndrome rescued by VA-ECMO. Albumin infusion did not improve lactate clearance but was highly efficient to reduce fluid loading in a porcine model of post resuscitation syndrome after refractory cardiac arrest treated with VA-ECMO.

**Compliance with ethics regulations:** Yes in animal testing.
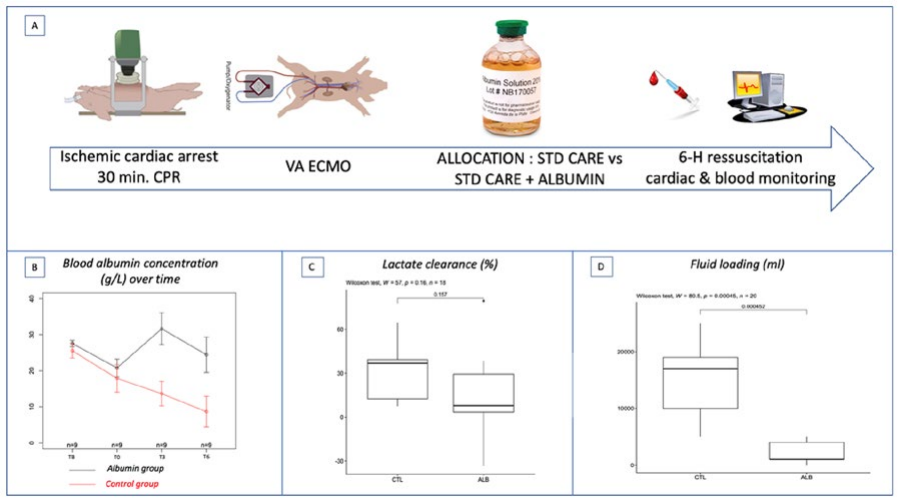


*Panel A: experimental protocol. Panel B: albumin concentration over time (in hours). Panel C: Lactate clearance 6 h after initial resuscitation. Panel D: fluid loading over a 6 h resuscitation. ALB: albumin group. CTL: control group.*

### FC-038 Clinical characteristic, injuries pattern and management of pediatric polytraumatized patients: an experience of an emergency departement

#### MALLEK Mariam^1^, KARRAY Rim^1^, BEN ALI Hana^1^, ZOUARI Alaeddine^1^, BEN SALEM Imen^1^, KSENTINI Hana^1^, NASRI Abdennour^1^, CHAKROUN Olfa^1^, REKIK Noureddine^1^

##### ^1^Service des Urgences et SAMU 04 CHU Habib Bourguiba Sfax Tunisie, Sfax, Tunisie

###### Correspondence: Rim KARRAY (karray_rim@medecinesfax.org)

*Annals of Intensive Care* 2022, **12(1):**FC-038

**Rationale:** Caring for pediatric trauma patient requires an understanding of the distinct anatomy and pathophysiology of the pediatric population compared with adult. As a result, the degree of awarning towards polytraumatized children must be higher. The aim of our study is to identify clinical and therapeutic aspects of pediatric polytraumatized patients.

**Patients and methods/Materials and methods:** A 5-month prospective study including polytraumatized children admitted to ICU of Emergency Department. Demographic clinical and radiological data are described. Management and outcomes of patients are also reported.

**Results:** We enrolled 20 pediatric polytraumatized patients. Fifty percent of them were boys. Road traffic was the main cause of pediatric trauma in 75% of cases. Half of the polytraumatized patients were conscious and 20% of children had a severe traumatic brain injury with a Glasgow coma scale under 8. In the majority of cases, pupils were normodilated. Convulsion and agitation were reported in 15% of patients. The most frequent brain injury was brain contusion in 55% of cases. Extradural, subdural hematoma and subarachnoid hemorrhage were present in 25% of cases. Chest trauma was present in 40% of cases and the main chest injury was thoracic contusion in 35% of cases. One in four children had an abdominal trauma with abdominal hemorrhage in 15% of cases, hepatic contusion in 10% and splenic contusion in 5% of cases. Pelvic fracture was present in 15% of cases. Airway management was performed in 35% of cases for neurological distress. Fluid administration was performed only in 15% of cases and transfusion in 30% of cases. A brain surgery was necessary in 20% of patients. No children died and only 10% referred to home after a stay in the emergency department. Overall, 85% of patients were transferred: 30% to the intensive care unit, 40% to neurosurgery service and 10% to a surgical pediatric unit.

**Conclusion:** Trauma is the cause of more than 45% of deaths in children aged 1 to 14 years. Since multiple injuries are common in children, the emergency physician must assess all organs of a child with high-energy injuries, regardless of the mechanism of the trauma.

**Compliance with ethics regulations:** Yes in clinical research.

### FC-039 Impact of pre-hospital intubation on the outcome of polytraumatic patients

#### MALLEK Mariam^1^, KARRAY Rim^1^, BEN ALI Hana^1^, MZOUGHI Faten^1^, BEN SALEM Imen^1^, CHAARI Leila^1^, CHAKROUN Olfa^1^, CHAARI Adel^1^, REKIK Noureddine^1^

##### ^1^service des Urgences et SAMU 04 CHU Habib Bourguiba Sfax Tunisie, Sfax, Tunisie

###### Correspondence: Rim KARRAY (karray_rim@medecinesfax.org)

*Annals of Intensive Care* 2022, **12(1):**FC-039

**Rationale:** Polytrauma is a leading cause of mortality and disability all over the world. Respiratory, hemodynamic and neurological distress are very frequent and correlated with high mortality. Therefore, an early energetic therapy controlling these distresses, by pharmacological methods and airway management can improve the outcome of polytraumatized patients. The aim of our study is to evaluate the impact of an early airway management on patient prognosis of patient in terms of length of stay in emergency department, mortality and neurological outcomes.

**Patients and methods/Materials and methods:** In this prospective study, only intubated patients were enrolled. The enrolled patients were randomly assigned into two groups: group A: intubated in prehospital phase and group B: intubated in hospital phase. Basic characteristics of injury, length of stay in emergency department, mortality and neurological outcomes according to the GOS score at one month (GOS1: death, GOS2: vegetative state, GOS3: severe state, GOS4: moderate state, GOS5: good recovery) lwere compared.

**Results:** During the study period, we enrolled 116 polytraumatized patients and 54 patients were intubated. The main indication of airway management was coma with a Glasgow coma scale < 8 in almost half of the population and cardiac arrest in 12% of cases. Seven percent and 3% of patients were intubated for hemodynamic and respiratory distress respectively. The two groups did not differ regarding to injury severity assessed by the ‘Injury severity score’ (p < 0.001). The mean length of stay in the emergency department was 23 ± 2 h in group B vs 20 ± 2 h in group A. There was no difference iin terms of transfer to an intensive care between the two groups (94% in each group). Two patients (10%) in group A and six patients (17%) in group B died. There was no difference in terms of neurological outcome: in Group A, 55% of patients had a GOS 5, 20% a GOS 3 and10% a GOS 2. In Group B, 40% of patients had a GOS 5,18% a GOS 3 and 12% a GOS 2 (p > 0.05 for each one).

**Conclusion:** Prehospital intubation reduces the length of stay in emergency department and mortality but has no influence on the orientation of patient or on their neurological evolution. However, ambulance practitioner should be familiar with airway management and should be trained in such a challenging treatment that could worsen the prognosis of polytraumatized patients if not performed correctly.

**Compliance with ethics regulations:** Yes in clinical research.

### FC-040 Chest trauma in children: a monocenter study

#### CHAARI Zied^1^, BEN AYED Aymen^1^, KAMMOUN Jaweher^1^, ABID Walid^1^, BEN AYED Ahmed^1^, HENTATI Abdessalem^1^, FRIKHA Imed^1^

##### ^1^Université de Sfax - CHU Habib Bourguiba, Sfax, Tunisie

###### Correspondence: Zied CHAARI (chaari.zied1@gmail.com)

*Annals of Intensive Care* 2022, **12(1):**FC-040

**Rationale:** Children are vulnerable to injuries most often occurring following a road accident. As part of polytrauma, pediatric thoracic trauma often requires special care.

**Patients and methods/Materials and methods:** This is a retrospective and analytical study including all children aged under 17 years of age, victims of thoracic trauma (whether open or closed), and hospitalized in our department (after or without admission in intensive care or pediatric resuscitation unit) between January 2015 and December 2021.

**Results:** During the study period, 25 children were admitted to our department following severe chest trauma with an average age of 9.7 ± 5.3 years (2–16 years) and a male predominance (88%). Road accidents (48%), domestic accidents (28%), and assaults (16%) were the most frequently reported circumstances. Seven children (28%) presented a penetrating thoracic trauma including 2 (28%) of ballistic origin. The most common lesions were pneumothorax (44%), cerebral contusions (43%), pulmonary contusions (40%), intra-abdominal lesions (36%), rib fractures (32%), pneumothorax (20%) and pneumomediastinum (12%). Cranial, peripheral, or pelvic fractures were associated in 28%, 16%, and 4% of patients respectively. The median length of stay in our service was 2 days (1-38 days). Urgent surgery was necessary for 6 children (24%) divided into 2 abdominal, 2 pulmonary, one pleural, and one cardiac surgery. The follow-up was favorable for the majority of patients (60%). Complications were mainly observed in patients who underwent surgery or were intubated for more than 4 days. No deaths were noted in our series.

**Conclusion:** The management of thoracic trauma in children must be multidisciplinary involving clinicians, emergency physicians, intensivists, as well as surgeons. Associated peripheral and cranial lesions influence the prognosis.

**Compliance with ethics regulations:** N/A.

### FC-041 Value of early thoracic computed tomography in severe blunt chest trauma

#### BOUGDAL Dalila^1^, SADAT Souhila^1^, ZEGHDOUD Dalila^1^

##### ^1^Etablissement SalimZemirli, Alger, Algerie

###### Correspondence: Dalila BOUGDAL (bougdalila@yahoo.fr)

*Annals of Intensive Care* 2022, **12(1):**FC-041

**Rationale:** Blunt chest trauma is common. Severe chest trauma can be life-threatening immediately or secondarily after a period of apparent calm. The objective of this study is to determine the contribution of early injury assessment to the morbidity and mortality of patients with severe blunt chest trauma.

**Patients and methods/Materials and methods:** prospective, observational study including 90 patients with severe blunt chest trauma, hospitalized in the ICU during the period March 2015–March 2017. We studied the lesion mechanism, the severity scores, we collected the pleuro-pulmonary lesions, their treatment and the future of the patients. We noted the hourly delays between the accident and the realization of the thoracic computed tomography (CT), as well as the delay between the admission to the hospital and the realization of the thoracic CT. We investigated the impact of thoracic CT on morbidity and mortality. The data were entered with the EPIINFO 7 software and analyzed using the SPSS statistics 23 software. The statistical tests are significant from p ≤ 0.05

**Results:** The mean age of the patients was 33.6 ± 17.9 years with a male predominance (sex-ratio = 4M/1F). 98.8% were polytraumatized. The mean ISS score was 34.5 ± 8.9; 68% of patients arrived with at least one vital distress. The delay between the thoracic radiological assessment and the time of the accident was less than 6 h in 73% of the patients. Furthermore, early assessment of injuries improved mortality (p = 0.02).

**Conclusion:** Early thoracic CT in severe blunt chest trauma allows an exhaustive diagnosis of thoracic lesions and improves mortality.

Keywords: Severe blunt chest trauma, Early chest CT.

**Compliance with ethics regulations:** N/A.

### FC-042 Flail chest: what are the associated morbidity and mortality risk factors?

#### CHAARI Zied^1^, BEN AYED Aymen^1^, KAMMOUN Jaweher^1^, BEN AYED Ahmed^1^, ABID Walid^1^, HENTATI Abdessalem^1^, FRIKHA Imed^1^

##### ^1^Université de Sfax - CHU Habib Bourguiba, Sfax, Tunisie

###### Correspondence: Zied CHAARI (chaari.zied1@gmail.com)

*Annals of Intensive Care* 2022, **12(1):**FC-042

**Rationale:** Rib fractures are frequently observed in patients with chest or multiple trauma. Flail chest represents a particular entity in terms of prognosis and management. Rare are the studies that have focused on the search for morbidity and mortality associated factors for patients with traumatic flail chest.

**Patients and methods/Materials and methods:** This was a retrospective and analytical study including all patients hospitalized in our department for a flail chest (with or without going through an intensive care or resuscitation unit) between January 1987 and December 2021.

**Results:** During the study period, 2342 patients were admitted to our department for chest trauma including 159 patients with confirmed flail chest (7%). The mean age was 53 ± 16 years (11–90 years) and a male predominance was noted (sex ratio = 6.22). The mean number of rib fractures was 12.9 ± 4.9 fractures (6–22 fractures). An intensive care unit hospitalization was necessary for 66% of patients. Conservative treatment (thoracic drainage, analgesia, nebulization and respiratory physiotherapy) was performed in the majority of cases (95%), and surgical treatment was necessary for only 8 patients (5%). During hospital stay we have reported complications for 56 patients (35%) and 7 patients died (hospital mortality of 4%). The mean injury severity score (ISS) was 20 (13–50). The factors associated with high morbidity rates were: ISS score > 25, hospitalization in intensive care unit, the need for intubation, and length of hospitalization > 7 days. For all possible mortality-studied factors, only an ISS score > 25 was associated with an increase in mortality rates.

**Conclusion:** Flail chest are associated with several factors influencing vital prognosis, as well as post-traumatic consequences during hospital or intensive care unit stays. A good knowledge of these factors would make it possible to better manage patients and improve subsequent follow-up.

**Compliance with ethics regulations:** N/A.

### FC-043 Antivenoms to treat Bothrops genus snakebite envenoming in the French Territories in the Americas: comparative experimental studies

#### FLORENTIN Jonathan, RESIERE Dabor^1^, KALLEL Hatem^2^, FLORENTIN Jonathan^1^, GUTIÉRREZ José Maria^3^, MEHDAOUI Hossein^1^, NEVIERE Remi^4^, MEGARBANE Bruno^5^

##### ^1^Critical Care Unit, University Hospital of Martinique (French West Indies), Fort-De-France, Martinique; ^2^Intensive Care Unit, Cayenne General Hospital, Cayenne, Guyana; ^3^Instituto Clodomiro Picado, Facultad de Microbiología, Universidad de Costa Rica, San José, Costa Rica; ^4^Department of cardiovascular surgery, University Hospital of Martinique, Fort-De-France, Martinique; ^5^Department of Medical and Toxicological Critical Care, Lariboisière Hospital, Paris University, INSERM UMRS1144, Paris, France

###### Correspondence: Dabor RESIERE (dabor.resiere@chu-martinique.fr)

*Annals of Intensive Care* 2022, **12(1):**FC-043

**Rationale:** In the French Territories in the Americas, *Bothrops* snakebite envenoming represent a public health issue. In Martinique, *Bothrops lanceolatus* is the only snake responsible for envenoming with thrombotic complications. In French Guiana, *Bothrops atrox* is responsible for most envenomings. The first antivenom specific to *Bothrops lanceolatus*, Bothrofav^®^1, produced in 1991, reduced complications. However, in 2004, an upsurge in cases of ischemic stroke despite early antivenom infusion, suggested a decline in its effectiveness. A new antivenom, Bothrofav^®^2 was produced in 2011 and manufactured in France. However, its marketing should be stopped in 2022. Polival-ICP^®^ (manufactured in Costa Rica) and Antivipmyn Tri^®^ (manufactured in Mexico) are successfully used to treat *Bothrops atrox* envenoming in French Guiana. We compared the effectiveness of all three antivenoms on *Bothrops lanceolatus* and *B. atrox* venoms using an experimental bench protocol.

**Patients and methods/Materials and methods:** We conducted third-generation antivenomics quantitative analyses, in vivo mouse and in vitro assays comparing Bothrofav^®^2, Polival-ICP^®^ and Antivipmyn Tri^®^ on *Bothrops lanceolatus* and *B. atrox* venoms.

**Results:** Bothrofav^®^2 immunocaptured all major *Bothrops lanceolatus* venom protein components, underscoring its high neutralizing efficacy (Table 1). Our in vivo and in vitro assays demonstrated its effectiveness in the neutralization of lethal, local and systemic haemorrhagic, oedema forming, myotoxic, thrombocytopenic, proteinase and phospholipase A2 activities, showing a higher preclinical efficacy as compared to previous batch used in the past. Regarding the neutralization of *B. atrox* venom, Polival-ICP^®^ has higher neutralizing activity than Antivipmyn Tri® against lethal, haemorrhagic and in vitro coagulant activities. Antivipmyn Tri^®^ and Polival-ICP^®^ antivenoms similarly neutralized the venom-induced myotoxic effects of *B. atrox* venom, while Antivipmyn Tri^®^ did not neutralize the lethal activity at the highest antivenom level tested.

**Conclusion:** Based on preclinical investigations, Bothrofav2^®^ was highly effective in the neutralization of the venom of *B. lanceolatus*, whereas Polival-ICP^®^ and Antivipmyn Tri^®^ neutralized the venom of *B. atrox*, albeit with different efficacies.

**Compliance with ethics regulations:** Yes in animal testing.

### FC-044 Association between healthcare trajectories before critical illness and 1-year survival among elderly patients hospitalized in ICU for acute respiratory infection—To predict the future, you need to know the past

#### TCHATAT WANGUEU Lionel^1^, GABORIT Christophe^1^, LAURENT Emeline^1^, GRAMMATICO-GUILLON Leslie^1^, GUILLON Antoine^1^

##### ^1^CHRU Tours, Tours, France

###### Correspondence: Lionel TCHATAT WANGUEU (tchatatlegrand@outlook.fr).

*Annals of Intensive Care* 2022, **12(1):**FC-044

**Rationale:** Intensive care unit (ICU) hospitalizations of elderly patients with acute respiratory infection (ARI) have increased; however, we observed an important mortality during the ICU stay and in the first year of hospital discharge. Yet many physicians have doubts as to whether elderly patients benefit from ICU admission. Our hypothesis is that healthcare trajectories before critical illness might be used as surrogate of frailty that can be useful for the decision-making. The aim of this study was to assess whether the healthcare trajectories in the 3 months preceding ICU admission were associated with 1-year survival in elderly patients with ARI.

**Patients and methods/Materials and methods:** A national population-based cohort study was performed from hospital discharge databases (2013–2017) and included ICU patients ≥ 80 years old with an ARI. Patient characteristics were collected as well as ICU procedures and their healthcare trajectories before and after discharge from ICU. Healthcare trajectories before critical illness were evaluated by the number of emergency room visits and the number of cumulative days of hospitalization 3 months before admission in ICU. Mortality refers to mortality at hospital or 1 year after discharge, during the follow-up period. The vital status of patients was assessed during at least 3-year follow-up. Logistic regression was used to find prehospital factors associated with mortality. Odd ratios (OR) and 95% confidence intervals (95% CI) were calculated. Results are in median (IQR).

**Results:** 40 327 patients aged 80 years or older were hospitalized in ICU for ARI during the studied period. Among them, 35 666 (88%) had a known vital status at 1 year of which 19 379 (54%) died during ICU stay or the first year of discharge. Patient characteristics were: age 84 [82–87] years old, male 19 708 (55.2%), SAPS II 30 [20; 44], invasive mechanical ventilation 9241 (26%). During the 3-month period preceding the ICU-admission, ≥ 1 day of hospitalization or ≥ 1 visit to the emergency room were independently associated with increased 1-year mortality: OR 1.27 [1.19–1.35] and 1.13 [1.06–1.20], respectively. Other independent factors associated with a worse outcome were: age (OR 1.05 [1.04–1.06]), male sex (OR 1.40 [1.34–1.46]), ≥ 3 comorbidities (OR 1.10 [1.03–1.18]) and frailty score ≥ 5 (OR 1.17 [1.11–1.25]).

**Conclusion:** Healthcare consumption 3 months prior to ICU was associated with 1-year mortality among elderly patients hospitalized for ARI. Healthcare consumption is an objective and easy-to-obtain information that may be used to implement a clinical score designed to help the decision-making for ICU admission of elderly with ARI.

**Compliance with ethics regulations:** Yes in clinical research.

### FC-045 Evaluation of preoperative frailty in cardiac surgery patients using the Edmonton Frail Scale. “Frail heart” study, a preliminary analysis

#### NTWALI Francis^1^, MOMENI Mona^1^, JACQUET Luc^1^, VANCAENEGEM Olivier^1^, LATERRE Pierre-François^1^, DECHAMPS Melanie^1^

##### ^1^UCLouvain, Bruxelles, Belgique

###### Correspondence: Francis NTWALI (francisntwali@gmail.com)

*Annals of Intensive Care* 2022, **12(1):**FC-045

**Rationale:** Frailty is a state of increased vulnerability to a stressor event, resulting in a poor resolution of homeostasis which increases the risk of adverse outcome. Recent studies have shown association between pre-operative frailty in older patients and mortality after cardiac surgery. The Edmonton Frail Scale (EFS) is a valid, quick to use tool which allows bedside assessment of preoperative frailty without the need of any special training. Frailty is usually assessed for patients aged 65 years and older, but prevalence in younger patients admitted to hospital is significant and is associated with adverse outcome. The primary objective of this study is to assess the association between frailty and intensive care unit (ICU) length-of-stay, hospital discharge after 30 days and one-year mortality in patients undergoing cardiac surgery, independently of age.

**Patients and methods/Materials and methods:** This is a prospective, monocenter and observational study in a tertiary care academic hospital. The study has been approved by the ethics comity of the hospital and all patients signed an informed consent. Overall, 233 consecutive patients who met the inclusion criteria (18 years old and older undergoing any cardiac surgery under cardiopulmonary bypass) and agreed with the study protocol were included between 25th April 2019 and 24th February 2020 and were evaluated preoperatively by EFS. Exclusion criteria were salvage surgery and patients with cirrhosis CHILD B or C.

**Results:** 191 (82%) patients were not frail, 42 (18%) were vulnerable or frail. The mean age of the entire cohort was 66.88 ± 12.01 years old. 80 patients were under 65 years old and 10 (12.5%) of them were vulnerable or frail. 153 were 65 years old or more and 32 (20.9%) of them were vulnerable or frail. Functional independence was the frailty domain with the strongest correlation with the overall EFS score. Prealbumin was the biological value with the strongest correlation with frailty (r = − 0.40, p < 0.05). In the non-frail group, 184 patients (96.3%) were discharged at day 30 compared to 34 (80.9%) in the frail group. The mean ICU length of stay was 3.38 ± 2.17 days in the non-frail group and 8.26 ± 14.95 days in the frail group. One year after surgery, 4 (2.3%) patients died in the non-frail group and 7 (21.9%) died in the frail group (p < 0.05).

**Conclusion:** Frailty significantly affects patients undergoing cardiac surgery, even under the age of 65, and is associated with significantly longer ICU length-of-stay, lower discharge at 30 days and higher one-year mortality.

**Compliance with ethics regulations:** Yes in clinical research.
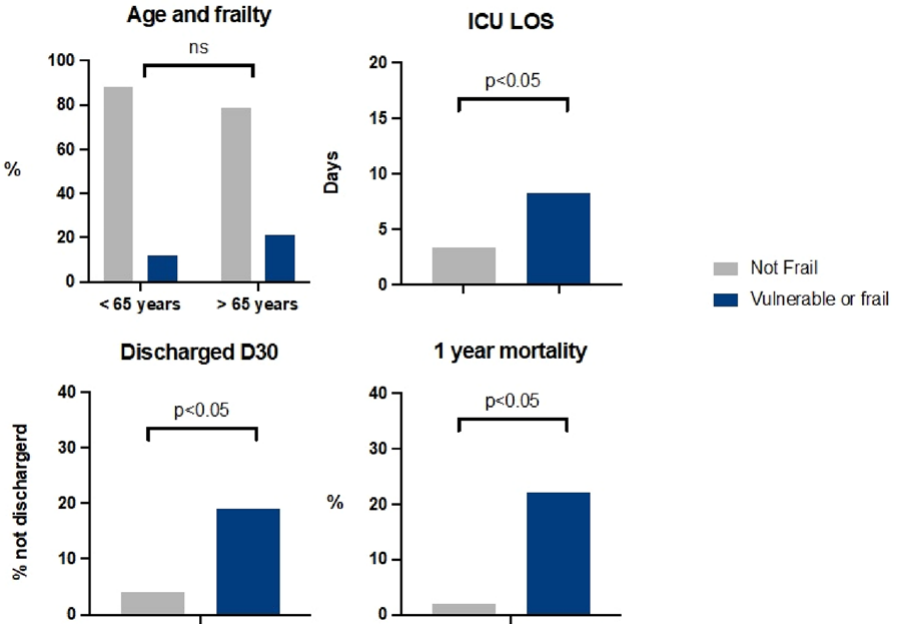


*Results*

### FC-046 Community peritonitis in the elderly

#### JARIR Ibtissam^1^, KHALLEQ Khalid^1^, BAZA Sanaa^1^, HARRAR Rachid^1^, BOUHOURI Mohamed Amine^1^, HATTABI Khalid^1^

##### ^1^CHU IBN ROCHD, Casablanca, Maroc

###### Correspondence: Ibtissam JARIR (ibtissamjarir30@gmail.com)

*Annals of Intensive Care* 2022, **12(1):**FC-046

**Rationale:** Community peritonitis in the elderly is a medical-surgical emergency, acquired by the patient in an extra hospital environment. The diagnosis is usually late due to the heterogeneity of clinical signs, this leads to a delay in management. The objective of our study is to evaluate the prognostic factors of an epidemiological, clinical, paraclinical, etiological, therapeutic and evolutionary order of the community peritonitis in the elderly in our context.

**Patients and methods/Materials and methods:** We conducted an analytical descriptive retrospective study of 50 cases of community peritonitis hospitalized in the surgical emergency resuscitation department over a period from January 2018 to June 2020. All patients over 65 years of age who were admitted for community peritonitis and who received medical and surgical care were included in this study. The parameters studied are demographic data, clinical and paraclinical signs, management and patient evolution. The Statistical analysis was performed using SPSS software, the evaluation of prognostic factors using a univariate and multivariate analysis.

**Results:** The study showed that the mean age was 70.08 ± 6.07 years, with a sex ratio of 2.12. The medical history was dominated by smoking (38%), the extra-abdominal signs (hemodynamic failure (70%), renal failure (68%), hematological disorders (48%), respiratory disorders (34%) andneurological disorders (32%)). Therapeutic management was based on perioperative resuscitation, treatment of organ failure, probabilistic antibiotic therapy and median laparotomy surgery. The main etiologies of community peritonitis were: peptic ulcer perforation (44%), intestinal perforation (22%), intestinal necrosis (12%), cholecystitis (4%). The bacteriological samples carried out in per operative allowed to have the following bacteriological profile: predominance of Gram-negative bacteria (61%) dominated by *E. coli* (39%), the average length of hospitalization was 6.32 ± 5.25 days. The mortality rate was 68%. The main prognostic factors identified in our univariate analysis study were: high blood pressure, organ failure, increased gravity scores, site of intervention, duration of intervention, use of catecholamines, development of septic shock, as well as duration of mechanical ventilation and duration of stay in intensive care unit. Multivariate analysis showed a statistically significant association between high blood pressure and the development of hemodynamic failure, creatinine > 13 mg/L, C-reactive protein > 150 mg/L, Occlusion, norepinephrine + epinephrine, duration of ventilation > 2.5 days and mortality.

**Conclusion:** Community peritonitis in the elderly is a serious condition, fraught with high mortality. The improvement of its prognosis is based on a screening of risk factors, and an updating of medical—surgical protocols.

**Compliance with ethics regulations:** Yes in clinical research.

### FC-047 COVID-19 and elderly population

#### CHOUCHÈNE Salma^1^, FATHALLAH Ines^1^, FAZZENI Hayfa^1^, MAHMOUD Jihene^2^, KOURAICHI Nadia^1^

##### ^1^Hôpital régional de Ben Arous, Ben Arous, Tunisie; ^2^hopital Sahloul, Sousse, Tunisie

###### Correspondence: Salma CHOUCHÈNE (Zamitisalma92@gmail.com)

*Annals of Intensive Care* 2022, **12(1):**FC-047

**Rationale:** To describe the clinical and prognostic particularities of COVID-19 infection in elderly patients hospitalized for a SARSCOV 2 infection.

**Patients and methods/Materials and methods:** Descriptive and comparative retrospective study conducted from September 2020 to September 2021. We included all patients admitted to intensive care with a serious SARSCOV2 infection. The infection was confirmed by RT-PCR, a rapid test or a typical chest CT scan. Elderly patients were defined as whom aged over 65 years old.

**Results:** We included 217 patients, 93 (42%) of whom were elderly subjects. The gender ratio was 1.11. The average IGSII score was 38 ± 14. For the past history, hypertension (56%) was at the top of the list followed by diabetes (43%). Twenty-eight patients were treated at home, 9 of whom required oxygen therapy. The first clinical examination showed anxiety and agitation in 17 (18%) and 18 (19%) patients respectively. Mechanical ventilation (MV) was used in 55 patients (59%) with prone positioning in 41 of cases (44%). Acute renal failure was noted in 46 (51%) patients and six (7%) patients required dialysis. Forty-one patients presented septic shock, 10 trophic disorders and five resuscitation delirium. The median durations of MV and intensive care unit (ICU) stay were respectively 7 [2;14] and 10 [6;16] days. The overall mortality was 53%. In the young population, more agitation (p = 0.033) and anxiety (p = 0.04) were noted with a longer duration of ventilation (11 vs 7 days (p = 0.004)). Elderly subjects were more severe on admission [IGSII score 37 vs 29 (p < 0.001)] and they developed more acute renal failure (51 vs 28%. (p < 0.001)). There was no significant difference between the two groups (old and young subjects) in terms of intubation time and mortality.

**Conclusion:** The elderly population was more severe on admission and the ICU stay was complicated by more acute renal failure. There was no difference in terms of mortality between young and old subjects.

**Compliance with ethics regulations:** Yes in clinical research.

### FC-048 Utilization of automated oxygen titration in patients managed at the emergency department for suspected or confirmed COVID-19

#### DALLAIRE Léa^1^, GUIROY Antoine^1^, BOUCHARD Pierre-Alexandre ^1^, LELLOUCHE Francois^1^

##### ^1^Institut Universitaire de Cardiologie et de Pneumologie de Québec, Québec City, Canada

###### Correspondence: Francois LELLOUCHE (francois.lellouche@criucpq.ulaval.ca)

*Annals of Intensive Care* 2022, **12(1):**FC-048

**Rationale:** During the COVID-19 pandemic, automated oxygen titration has been implemented at the emergency department (ED) to maintain the patients within the oxygenation targets and to reduce the interventions from healthcare workers. We report here our experience to manage patients with suspected or confirmed COVID-19 at the ED.

**Patients and methods/Materials and methods:** We retrospectively collected data from the automated oxygen titration device (O_2_ flow, SpO_2_, SpO_2_ target, respiratory rate and heart rate) in patients managed at the ED between April 2021 and February 2022. We analyzed the time within the SpO_2_ target (set SpO_2_ ± 2%), with hypoxemia (SpO_2_ < target − 5%), with hyperoxemia (SpO_2_ > target + 5%). We analyzed the whole population and the subgroup of patients with confirmed COVID-19. We evaluated the number of patients with oxygen weaning during the management. We evaluated separately the impact of the SpO_2_ target on oxygen flow when the target was modified by the clinicians by 2% or more. In this subgroup of patients, we evaluated the mean oxygen flowrate 15 min before and after the modification of the SpO_2_ target.

**Results:** We included 98 patients (mean age 72 ± 16 years, 57% were men) admitted to the ED with acute respiratory distress and suspected (n = 77) or confirmed (n = 21) COVID-19 requiring oxygen therapy. The mean duration of utilization of automated oxygen titration device was 14.3 ± 4.7 h. A SpO_2_ signal was present 91.9% of the time. Main SpO_2_ targets set by the clinicians were 90% (43%), 88% (27%), 92% (20%), 94% (4%). Oxygen weaning was possible in 35/98 patients (36%). For the whole population/COVID-19 patients, the time in the SpO_2_ target was 78/81%, time with hypoxemia was 3/3%, time with hyperoxemia was 2/0.6%. In a subgroup of 11 patients, the effect of modifying the SpO_2_ target was evaluated. The mean initial SpO_2_ target was 91.9% and the mean final target was 89.5%. The mean initial and final oxygen flow were 3.3 ± 0.6 and 1.9 ± 0.6 L/min respectively, P = 0.0076.

**Conclusion:** In patients with acute respiratory failure, the utilization of automated oxygen therapy was feasible to manage patients with suspected or confirmed COVID-19 with potential benefits. Similarly to other studies with automated oxygen therapy, the time in the oxygenation target was high, and oxygen weaning was potential in 1/3 of the patients. SpO_2_ target has a significant impact on oxygen flow rates and is a useful tool with this new device.

**Compliance with ethics regulations:** Yes in clinical research.
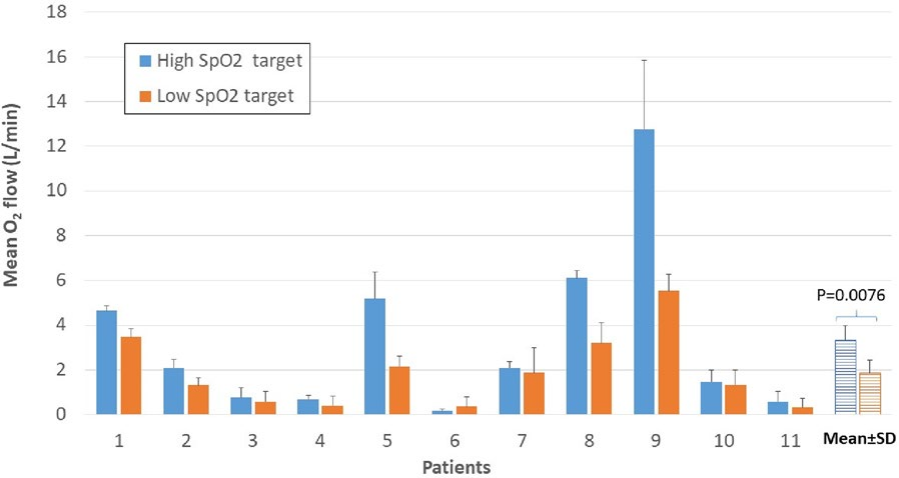


*impact of the SpO*_*2*_ *target on oxygen flowrate in a subgroup of 11 patients with modified target. With high SpO*_*2*_ *target (mean 91.9%) reduced to low SpO*_*2*_ *target (mean 89.5%), oxygen flow was significantly reduced from 3.3 to 1.9 L/min.*

### FC-049 Acute heart failure in the emergency department: prognosis value of PaO_2_/FiO_2_ ratio

#### GHABARA Racha^1^, BAHRI Badra^1^, SEDGHIANI Ines^1^, KHIARI Saoussen^1^, DOGHRI Hamdi^1^, TOUJ Hager^1^, ZAGHDOUDI Imen^1^, FALFOUL Nebiha^1^

##### ^1^Hopital Habib Thameur, Tunis, Tunisie

###### Correspondence: Racha GHABARA (Rsha993@gmail.com)

*Annals of Intensive Care* 2022, **12(1):**FC-049

**Rationale:** Heart failure is a common presentation in the emergency department. Searching for risk factors is a challenging step in the emergency physicians. The purpose of our study was to search for prognosis values of ratio PaO_2_/FiO_2_ in patients admitted to the emergency department for acute heart failure.

**Patients and methods/Materials and methods:** Prospective monocenter observational study, conducted between November 2021 and January 2022. We included patients over the age of 18, we established diagnosis of heart failure on clinical grounds. We performed blood gases at admission. The primary endpoint was in-hospital mortality.

**Results:** We included 100 patients. Average age: 73 ± 11 years and sex ratio M/F = 51/49. The most common comorbidities were hypertension: 81(82%), diabetes 46 (47%), coronary artery disease 47(47%) patients; 68% had a left ventricular ejection fraction (LVEF) more than 40%, with an average pro-BNP of 9,440 pg/mL; 37% of patients had permanent atrial fibrillation. 24% returned in < 30 days with an average of readmissions/year of 1.75(± 0.96). Seventy-two patients (76%) required ventilatory support, 5% required the use of invasive mechanical ventilation. The mortality rate was 12%. There was no significant difference in terms of comorbidity between the group of deceased and surviving patients. However, deceased patients had a higher SOFA score (4 vs 2; p = 0.001), a lower LVEF (43% vs 25%; p = 0.18). D-dimers and CRP were higher in the deaceased patients, with a median value of 3636 vs 1385 µg/L (p = 0.001) and 98 vs 56 mg/L (p = 0.08) respectively. The PaO_2_/FiO_2_ ratio in admission was 195 in the deceased patients vs 298 in the surviving patients (p = 0.01). The PaO_2_/FiO_2_ ratio was as an independent mortality factor (OR = 0.99, CI [0.98; 1.00], p = 0.05). Using the ROC curve, the best cut-off for the PaO_2_/FiO_2_ ratio was 160. A PaO_2/_FiO_2_ ratio ≤ 160 was predictive of mortality with an area under curve of 0.74, a sensitivity of 86% and a specificity of 76%.

**Conclusion:** A PaO_2_/FiO_2_ ratio ≤ 160 is an independent factor of death in patients admitted to the emergency department with acute heart failure.

**Compliance with ethics regulations:** Yes in clinical research.

### FC-050 Care prognosis and outcomes in elderly patients admitted to the intensive care unit

#### JERBI Mouna^1^, GHORBEL Rezk^1^, REKIK Achraf^1^, BEN JEDDOU Kais^1^, GHARBI Emna^1^, BEN AMAR Boutheina^1^, CHAKROUN Olfa^1^, REKIK Noureddine^1^

##### ^1^Centre Hospitalo-universitaire Habib Bourguiba sfax, Sfax, Tunisie

###### Correspondence: Rezk GHORBEL (rezkghorbel3@gmail.com)

*Annals of Intensive Care* 2022, **12(1):**FC-050

**Rationale:** To describe the medical histories, demographic characteristics and analyze the standardized geriatric assessment in elderly patients admitted to emergency department intensive care unit (EDUCI). To determine the pathologies found in these patients as well as the therapeutic and outcomes.

**Patients and methods/Materials and methods:** This is a prospective descriptive study carried out at the EDUCI of the Habib Bourguiba University Hospital, during a period of four months. We included all people aged 65 and older admitted to our intensive care unit. We observed their therapeutic management as well as their demographic and evolutionary characteristics. We also calculated the ADL score for all patients and followed their evolution at discharge from the emergency room.

**Results:** Our study involved 262 patients during the enrollment period. The average age was 77 ± 8 years with a sex ratio of 1.1. From pathological medical history was noted in most patients (82%) dominated by hypertension and diabetes mellitus. The majority of patients (74%) are polymedicated. The history of hospitalization was found in 42% of cases. Data anamnestics have shown the presence of cognitive disorders and Alzheimer’s disease in 21% and 7% of cases, respectively. The ADL score showed a loss of autonomy for 39% of the population. The reasons of hospitalizations were mainly medical and, to a lesser extent, traumatic and visceral reasons while the deterioration of the general state was involved in 5% cases. Overall, 11% of patients required mechanical ventilation and 9% of patients required a blood transfusion. The average length of hospital stay was 3 ± 4 days. The evolution was favorable in the majority of cases (75%). Among home leavers (41%), a quarter sustained moderate or severe sequelae. These sequelae were essentially neurological deficit, resuscitation neuromyopathy and/or pressure sores. The evolution has been fatal in 17% of cases, in the EDICU. The main causes of death were cerebral suffering (8%), septic shock and hypoxemia.

**Conclusion:** The admission of geriatric patients to the ICU is increasing. Frailty assessment may play an important role in the clinical evaluation of such individuals for triage, but should not be considered a priori as an exclusion criterion for admission. Physicians are aware of the difficulties faced with such patients and the need to promote short admission pathways.

**Compliance with ethics regulations:** Yes in clinical research.

### FC-051 French translation and validation of the Healthy Aging Brain Care-Monitor, Hybrid Version (HABC-M-HV): a new tool for remote post-intensive care syndrome screening

#### BOUGARD Laurine^1^, BEAUDART Charlotte^1^, COLSON Camille^1^, HORLAIT Geoffrey^2^, BORNHEIM Stephen^3^, BRUYERE Olivier^1,3^, MISSET Benoit^1^, ROUSSEAU Anne-Françoise^1^

##### ^1^CHU Liège, Liège, Belgique; ^2^CHU UCL Namur, Namur, Belgique; ^3^University of Liège, Liège, Belgique

###### Correspondence: Laurine BOUGARD (laurine_bougard@hotmail.com)

*Annals of Intensive Care* 2022, **12(1):**FC-051

**Rationale:** Patients surviving a stay in an intensive care unit (ICU) may experience new or worsening disorders that have been labeled as “post-intensive care Syndrome” (PICS). PICS includes physical weakness, mental disorders and neurocognitive impairments that can affect the patient’s quality of life. Authors of the Healthy Aging Brain Care Monitor have developed a hybrid version (HABC-M-HV) suited to the daily needs of their post-ICU follow-up clinic. This is a 30-item questionnaire with 4 subscales for cognitive, functional, psychological and quality of life assessment. The hybrid version of the HABC-M questionnaire (HABC-M-HV) has not yet been validated in its English version. Using rigorous methodologies, the objectives of this cross-sectional observational study were to translate the HABC-M-HV questionnaire into French (HABC-M-HV-F, Fig. 1) and to evaluate the main measurement properties of this new version.

**Patients and methods/Materials and methods:** The questionnaire was translated following a five-stage validated method for the translation and cross-cultural adaptation of questionnaires. A convenience sample of ICU survivors was recruited in our follow-up clinic to validate the questionnaire. The HABC-M-HV-F was administered by phone. The measurement performances of the questionnaire were tested using internal consistency, test–retest reliability, standard error of measurement (SEM) and smallest detectable change (SDC) calculation, floor and ceiling effect measurement and construct validity.

**Results:** A total of 51 patients with 14/51 (27.5%) women were recruited between February and September 2021. Their median age was 63 [55–71] years. The internal consistency was very good (Cronbach’s alpha coefficient 0.79). The intra- and inter-examinator reliabilities were excellent (Intraclass Coefficient Correlation = 0.99 and 0.97, respectively). Total scores of the HABC-M-HV-F were very similar between test and retest with the same examinator, respectively 9 [4–15] and 8 [4–16], as well as with two examinators, respectively 12 [6–24] and 12 [4–23]. The SEM was 0.62 and the SDC was 1.72. No floor nor ceiling effects were observed. The convergent validity was almost entirely confirmed with 71.4% of our hypothesis confirmed.

**Conclusion:** The HABC-M-HV-F has been shown to be a valid and reliable tool for standardized PICS screening and follow-up among French-speaking ICU survivors. A remote administration by phone is feasible, making it an advantageous alternative in the growing context of telemedicine.

**Compliance with ethics regulations:** Yes in clinical research.
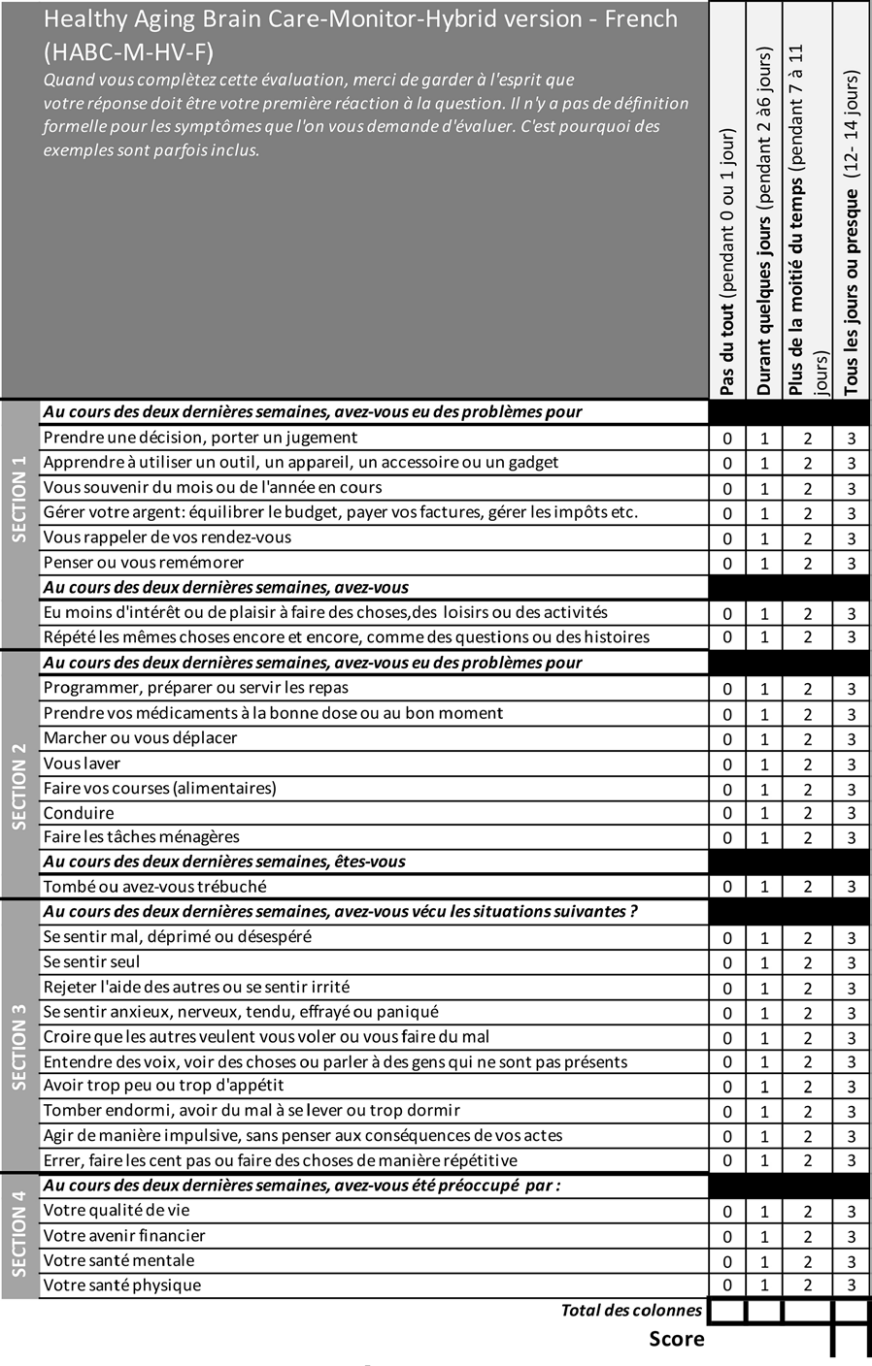


*Healthy Aging Brain Care Monitor Hybrid Version in French*

### FC-052 Ceftolozane/Tazobactam use for the treatment of bacterial infections in France: focus on patients with abnormal renal functions

#### RUIMY Raymond^1^, AKRICH Brune^2^, BOURGE Xavier^2^, BOUTOILLE David^3^, BRASSAC Isabelle^2^, CASTAN Bernard^4^, LORIEAU-THIBAULT Raphaèle^2^, MACKOSSO Carole^2^, MONTEIRO TAVARES Linsay^5^, MOOTIEN Joy^6^, RUIZ Fabrice^5^, TIMSIT Jean-François^7^

##### ^1^CHU Nice, Nice, France; ^2^MSD France, Puteaux, France; ^3^CHU Nantes, Nantes, France; ^4^CH Périgueux, Périgueux, France; ^5^ClinSearch, Malakoff, France; ^6^CHU Mulhouse, Mulhouse, France; ^7^AP-HP Bichat, Paris, France

###### Correspondence: Gabriella PASSONI (gabriella.passoni@clinsearch.net)

*Annals of Intensive Care* 2022, **12(1):**FC-052

**Rationale:** The Conduct study was initiated following a request from the French Health Authorities, to describe the use of Ceftolozane/Tazobactam (C/T) in current clinical practice.

**Patients and methods/Materials and methods:** This was an observational, prospective, multicenter, French study. Any patient having received at least one dose of C/T was eligible to participate and followed-up upon inclusion until stop of C/T. This analysis aims to examine C/T outcomes relative to renal functions, in patients with pneumonia, or treated for complicated intra-abdominal (cIAI) or urinary tract infections (cUTI) and pyelonephritis (other indications). Current recommended daily C/T doses are 6 g/3 g for pneumonia treatment (indication approved in August 2019), and 3 g/1.5 g for the other indications, with decreased daily doses recommended in patients with renal impairment. Normal renal functions were defined as creatinine clearance (CLCR) between 50–150 mL/min, augmented renal clearance (ARC) was defined as CLCR > 150 mL/min, and renal impairment (RI) as CLCR < 50 mL/min.

**Results:** Between October 2018 and December 2019, 260 patients were enrolled and data on renal functions were available for 240. Of these, 133 (55.4%) presented with pneumonia (of whom, 30.8% [N = 41/133] included after the approved indication), and 50 (20.8%) received C/T for the three other approved indications (the remaining 57 received C/T for another indication—data not shown). Baseline demographic and clinical characteristics are presented in Table 1. Among patients treated for pneumonia and included after the indication was approved, all RI patients received a dose adaptation (less than 6 g/3 g). On the other hand, 3/5 (60%) of ARC patients and 10/29 (34.5%) of patients with normal renal functions received doses lower than 6 g/3 g. For the other indications, the majority of ARC and normal renal function patients received the standard dose (57.1% [N = 4/7] and 71% [22/31], respectively); 42.8% of ARC patients and 22.6% with normal renal function received higher doses (between 4 g/2 g and 6 g/3 g). Among RI patients, dose adaptation was not strictly followed (8/12 received 3 g/1.5 g, and one patient received 4.5 g/2.25 g). In both groups, the most commonly reported reasons for treatment termination were complete or partial cure (100%, 93.1%, 85.7%, for ARC, normal renal functions, and RI pneumonia patients respectively, and 57.1%, 71%, 66.6%, for the same subgroups in patients with other indications).

**Conclusion:** These results suggest that C/T dose adaptation is followed by most clinicians, and provides important cure rates among all renal function groups.

**Compliance with ethics regulations:** Yes in clinical research.
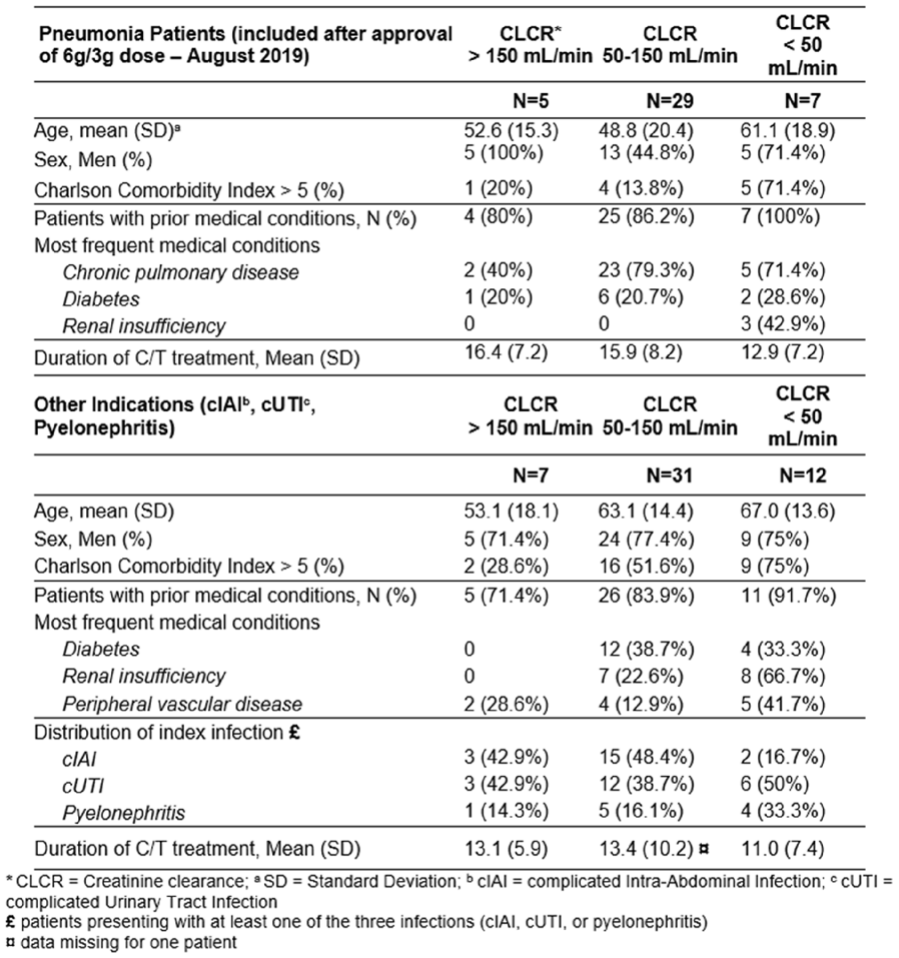


*Table 1*

### FC-053 Does the cefepime plasma concentration at steady-state in critical ill patients frequently exceed the maximum target assumed to be neurotoxic?

#### JEAN-MICHEL Vanessa^1^, HOMEY Corentin^1^, CAULIER Thomas^1^, DELANNOY Pierre-Yves^1^, BOUSSEKEY Nicolas^1^, GEORGES Hugues^1^

##### ^1^CH TOURCOING, Tourcoing, France

###### Correspondence: Vanessa JEAN-MICHEL (vanessa.jeanmichel@gmail.com)

*Annals of Intensive Care* 2022, **12(1):**FC-053

**Rationale:** Cefepime (CEF) is one of antimicrobials commonly prescribed for nosocomial infections in intensive care unit (ICU). Neurotoxicity of cefepime can occur for plasma concentration > 35 mg/L, when administered by continuous infusion [1,2]. We aimed to assess the risk factors of plasma CEF concentration at steady state above 35 mg/L. We also determined the relationship between neurotoxicity, CEF concentration and general co-morbidities.

**Patients and methods/Materials and methods:** We performed a retrospective study of adult ICU patients treated with CEF for at least 48 h between January 2019 and September 2021.

**Results:** Fifty-seven patients were included. Twenty-one patients had a renal clearance < 60 mL/min/1.73 m^2^ before CEF initiation. The mean starting daily dose of CEF was 5.4 (1.3) g. The mean time to first CEF monitoring was 2.9 (1.7) days and the incidence of CEF concentration above 35 mg/L was 46/57 (80.7%) patients. Factors independently associated with CEF concentration above 35 mg/L were age upper to 65 years old [OR = 4.1 (95% CI 1.1–62.1), p = 0.04] and initial daily delivery of 6 g of CEF [OR = 2.7 (95% CI 1.2–68.9); p = 0.03]. Presumed CEF neurotoxicity was 12/57 (21.1%) patients. Mean CEF concentration was higher for patients who developed neurotoxicity: 85 (31.2) mg/L vs 50.9 (23.4) mg/L; p = 0.001. No patient with neurotoxicity had a CEF concentration of 35 mg/L or less. The mean treatment duration was 5.4 (2.6) days. The mean time to develop neurotoxicity related to CEF was 4.8 (1.8) days. Seventeen patients were not neurologically assessable due to deep sedation. The mean time from CEF change to neurological improvement was 2.8 (1.6) days. CEF concentration above 40 mg/L (p = 0.01) was more frequently associated with neurotoxicity in univariate analysis but did not appear to be an independent factor. The only independent factor for presumed CEF neurotoxicity was estimated glomerular filtration rate below 60 mL/min/1.73 m^2^ at initiation of treatment [OR = 8.6 (95% CI 1.4–51.9); p = 0.02].

**Conclusion:** CEF concentration above 35 mg/L is frequent and therefore should be systematically investigated to prevent neurotoxicity.

**Reference 1:** Guilhaumou R, Benaboud S, Bennis Y, Dahyot-Fizelier C, Dailly E, Gandia P, et al. Optimization of the treatment with beta-lactam antibiotics in critically ill patients. Crit Care Lond Engl. 2019;23:104.

**Reference 2:** Huwyler T, Lenggenhager L, Abbas M, Ing Lorenzini K, Hughes S, Huttner B, et al. Cefepime plasma concentrations and clinical toxicity: a retrospective cohort study. Clin Microbiol Infect. 2017 Jul;23(7):454–9.

**Compliance with ethics regulations:** Yes in clinical research.

### FC-054 Population pharmacokinetics of fluconazole and dosing simulations in critically ill patients

#### VASSAL Olivia^1^, MATUSIK Elodie^2,3^, FERRY Tristan^4,5^, MILLET Aurelien^6^, BOHE Julien^1^, GUITTON Jerome^6^, FRIGGERI Arnaud^1,5^, GOUTELLE Sylvain^2,3^

##### ^1^Service Anesthésie Reanimation Medecine Intensive, Groupement Hospitalier Sud, Hospices Civils de Lyon, Pierre Benite, France; ^2^Service Pharmacie/Pharmacologie, Groupement Hospitalier Nord, Hospices Civils de Lyon, Lyon, France; ^3^CNRS UMR 5558 LBBE, Université Lyon 1, Lyon, France; ^4^Service des maladies infectieuses et tropicales, Groupement Hospitalier Nord, Hospices Civils de Lyon, Lyon, France; ^5^Inserm U111, CNRS UMR 5308, Centre International de Recherche en Infectiologie, Lyon, France; ^6^Laboratoire de Pharmacologie toxicologie, Groupement Hospitalier Sud, Hospices Civils de Lyon, Lyon, France.

###### Correspondence: Olivia VASSAL (Oliviavassal@gmail.com)

*Annals of Intensive Care* 2022, **12(1):**FC-054

**Rationale:** Fluconazole pharmacokinetics (PK) may be altered in critically ill patients, but changes and implications for drug dosing remain unclear. We aimed to describe the PK and dosage requirements of fluconazole in intensive care unit (ICU) patients.

**Patients and methods/Materials and methods:** This was a PK study performed in an ICU over 1 year. All patients who were prescribed fluconazole with at least two measured concentrations were included. A second dataset from a medical unit was also analyzed for comparison. Population PK modelling was performed by with Monolix software. Classical criteria were used for model evaluation including goodness-of-fit and simulation-based diagnostics. Patient covariates were investigated. Dosing simulations were performed with the final model with Simulx software. Various loading (LD) and maintenance doses (MD) were evaluated, and probability of target attainment (PTA) was computed in simulated patients (n = 1000). PK targets were set as a trough concentration (Cmin) of 15 mg/L and a daily AUC of 400 mg.h/L.

**Results:** A total of 202 plasma concentrations were available. They were 33 men and 19 women, with mean age of 61 ± 16 years and mean initial weight of 72 ± 21 kg. 59% of patients had proven invasive candidiasis (*C. albicans* 35%, *C. tropicalis* 8%) and 4% has cryptococcosis. In ICU patients, a LD of 800 mg was administered in 83% of patients. Data were best described with by a one-compartment model with first-order elimination. The mean (CV%) of body clearance (CL) and volume of distribution (Vd) parameters were as follows: 0.46 L/h (56%) and 40.72 L (10%). ICU hospitalization was associated with a 80% increase in CL. The optimal dosage regimen was based on a LD of 800 mg q12h over the first 24 h followed by a MD of 600 mg/24 h. This dosage was associated with median [percentiles 5^th^–95th] Cmin of 29.5 mg/L [19.2–37.3] at 24 h and 30.9 mg/L [8,6–62,8] at 120 h and a median AUC of 583.7 mg.h/L [461.4–706.5] at 24 h and 895.0 mg.h/L [356.8–1597.0] at 120 h. PTA at 24 h and 120 h were as follows 98.7% and 99.9% for Cmin; 99.2% and 93.4% for AUC, respectively. Only 6% of patients had a concentration over 80 mg/L after 5 days.

**Discussion:** We identified increased fluconazole clearance in ICU patients. LD and MD higher than currently recommended are necessary to achieve PK/PD targets in most patients.

**Conclusion:** This study confirms interindividual variability of fluconazole PK and the need for higher dosage in ICU patients. Early therapeutic drug monitoring may be useful for dosage individualization.

**Compliance with ethics regulations:** Yes in clinical research.

### FC-055 Factors associated with failure to achieve meropenem plasma concentration target in critically ill patients

#### TOURNAYRE Sarah^1^, MATHIEU Olivier^1^, VILLIET Maxime^1^, KLOUCHE Kada^1^, LARCHER Romaric^2^

##### ^1^CHU MONTPELLIER, Montpellier, France; ^2^CHU NIMES, Nimes, France

###### Correspondence: Sarah TOURNAYRE (tournayresarah@gmail.com)

*Annals of Intensive Care* 2022, **12(1):**FC-055

**Rationale:** Optimal management of septic shock requires prompt and adequate administration of broad-spectrum antibiotic therapy, ensuring that effective therapeutic levels are achieved. We aimed to determine factors associated with failure to achieve meropenem plasma concentration target in critically ill patients.

**Patients and methods/Materials and methods:** Between January 2019 and March 2020, charts of all patients admitted in medical intensive care unit (ICU) for septic shock and treated with meropenem and who had at least one meropenem plasma concentration measurement were reviewed. Adequate target meropenem plasma concentration was ranged from 10 to 50 mg/L. Factors associated with meropenem plasma concentration target non-attainment were assessed by multivariable logistic regression (R software version 4.1.2).

**Results:** 117 meropenem plasma concentrations were measured 3 days after initiation of meropenem or change on its dosage regimen, in 70 ICU patients. Meropenem was administered by prolonged intermittent infusion in 34 patients and by continuous infusion in 36. Factors independently associated with failure to achieve 10 mg/L of meropenem plasma concentration were female sex, age < 65 years old, prolonged intermittent rather than continuous infusion and low daily doses of meropenem (Fig. 1). Factors associated with plasma concentrations above 50 mg/L were low body weight, renal clearance < 25 ml/min and higher daily doses of meropenem. Of note, only 3 patients had neurotoxicity.

**Conclusion:** In ICU patients, age < 65 years old, female sex, prolonged intermittent infusion and low dose of meropenem were significantly associated with failure to achieve meropenem plasma concentration target. Some patients experienced a high meropenem plasma concentration but few of them had clinical signs of toxicity. Our results suggest to prefer continuous infusion of meropenem over 24 h rather than prolonged intermittent infusion, particularly in patients with normal or increased renal clearance.

**Reference 1:** Abdul-Aziz, M.H., Alffenaar, JW.C., Bassetti, M. et al. Antimicrobial therapeutic drug monitoring in critically ill adult patients: a Position Paper#. Intensive Care Med 46, 1127–1153 (2020).

**Reference 2:** Li C, Du X, Kuti JL, Nicolau DP..Clinical pharmacodynamics of meropenem in patients with lower respiratory tract infections. Antimicrob Agents Chemother 51:1725–1730 (2007).

**Compliance with ethics regulations:** Yes in clinical research.
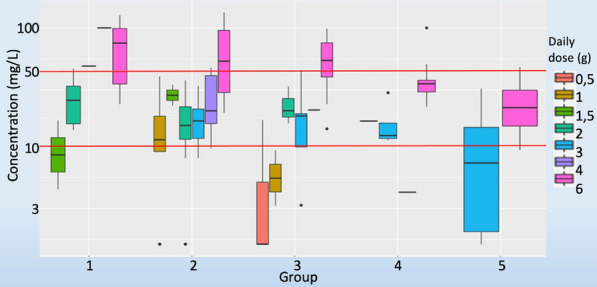


*Meropenem concentration after different daily dose among patients with different renal clearance. Group 1* = *CrCl* < *25 ml/min, Group 2* = *CrCl 25–50 ml/min or CRRT, Group 3* = *CrCl 50–90 ml/min, Group 4* = *CrCl 90–130 ml/min, Group 5* = *CrCl* > *130 ml/min.*

### FC-056 Optimisation of piperacillin-tazobactam dosing regimen for critically-ill patient with sepsis: the OPT-TAZ population pharmacokinetics study

#### SARFATI Sacha^1^, WILS Julien^1^, CARPENTIER Dorothée^1^, NILES Christopher^2^, GOUIN Philippe^1^, BRAULT Clément^3^, IMBERT Laurent^1^, GAËTAN Béduneau^1^, NSEIR Saad^2^, DAHYOT Sandrine^1^, MISSET Benoit^4^, LAMOUREUX Fabien^1^

##### ^1^CHU de Rouen, Rouen, France; ^2^CHRU de Lille, Lille, France; ^3^CHU d'Amiens, Amiens, France; ^4^CHU de Liège, Liège, Belgique

###### Correspondence: Sacha SARFATI (sacha.sarfati@gmail.com)

*Annals of Intensive Care* 2022, **12(1):**FC-056

**Rationale:** In critically-ill patient with sepsis, the piperacillin-tazobactam (pip-taz) combination is one of the most used antibiotics. Although pharmacokinetics of pip-taz exhibits very high inter-individual variability mainly based on volume of distribution, renal clearance, patient’s weight and serum albumin level, a standard dosing protocol is generally used (1). Moreover, these covariates are highly variable in sepsis and predictive algorithms based on clinical and biological parameters may help to optimize dosing and improve outcomes. The aim of the study is to use a population pharmacokinetics study to build a Bayesian model predicting the optimal dosing regimen of pip-taz in sepsis patients.

**Patients and methods/Materials and methods:** The OPT-TAZ (optimisation of pip-taz) study is a prospective multicenter open-label non-comparative cohort study designed with a 60-patient learning cohort and a 30-patient validation cohort. Patients will be included prospectively in 4 ICUs in France. The protocol plan to analyze pharmacokinetics of 60 patients then development of a predictive model validated on 30 patients. We present here the analysis of the 60 first patients. Standard dosing of pip-taz 4 g/0,5 g each 6 h (group 1) or 8 h (group 2) in 3 h infusion has been chosen according to clinician choice. Serum concentration of piperacillin and tazobactam has been measured at H0, 1.5, 3, 4, 5 (G1) or 6 (G2), 6 (G1) or 8 (G1), 12 (G1) or 16 (G2), 24, 48 and 120 with a liquid chromatography coupled with tandem mass spectrometry (LC–MS/MS).

**Results:** In our cohort, we showed that pharmacokinetics of pip-taz present a very high inter-individual variability. Residual concentration was lower than recommended target concentration in more than 50% of cases. Dosing of 4 g/0.5 g every 8 h and augmented renal clearance were risk factor for failure of PK/PD target attainment. PK of piperacillin and tazobactam show a strong association and tazobactam concentration was of the order of 10% of piperacillin concentration.

**Conclusion:** In conclusion, pip-taz dosing regimen must be adapted to patient characteristics. Creatinine clearance is one of the most important covariates to take into consideration for dose adaptation. The dose of 4 g/0.5 g each 8 h must be considered of high risk of failure of PK/PD attainment in patient with no impaired renal function. Therapeutic drug monitoring is an essential tool to assess the match of dosing regimen with PK/PD target.

**Reference 1:** (1) Huttner A, Harbarth S, Hope WW, Lipman J, Roberts JA. Therapeutic drug monitoring of the ?-lactam antibiotics: what is the evidence and which patients should we be using it for? J Antimicrob Chemother2015;70(12):3178–83.

**Compliance with ethics regulations:** Yes in clinical research.
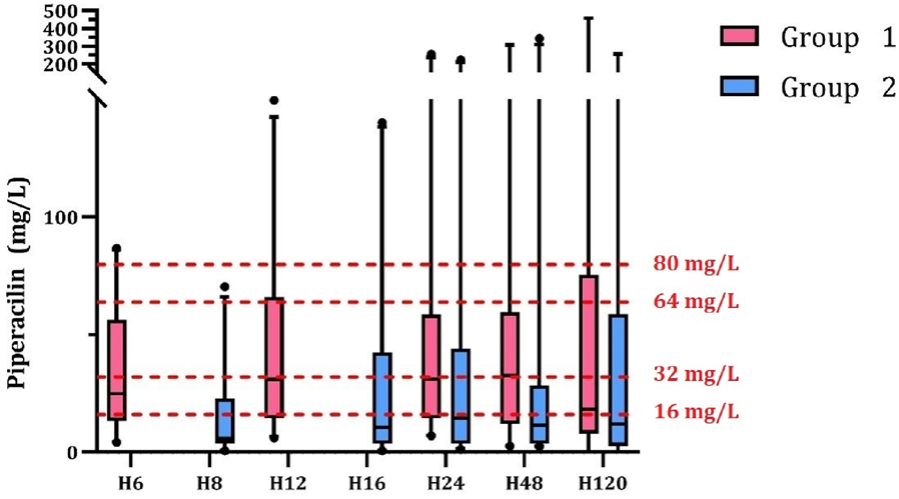


*Residual concentration of piperacillin over time in group 1 (4 g each 6 h) and group 2 (4 each 8 h) compared to different level of PK/PD target.*

### FC-057 Should we really ban 3rd generation cephalosporin (C3G) use for the treatment of Enterobacter cloacae infection in ICU patients?

#### BERNIER Juliette^1^, TANKOVIC Jacques^1^, VILLA Antoine^1^, URBINA Tomas^1^, BONNY Vincent^1^, GABARRE Paul^1^, AIT OUFELLA Hafid^1^, MAURY Eric^1^

##### ^1^CHU Saint Antoine, Paris, France

###### Correspondence: Juliette BERNIER (j.bernier1505@laposte.net)

*Annals of Intensive Care* 2022, **12(1):**FC-057

**Rationale:** Limiting the ecological impact of antibiotics through accurate prescription is one of the major goals of antibiotic stewardship policy. However, an antibiotic-sparing strategy must not have a potential deleterious impact on patient’s prognosis. Group 3 Enterobacteriaceae possess the AmpC gene encoding a cephalosporinase. Antibiotic therapy with a 3rd generation cephalosporin (C3Gs) might lead to treatment failure when cephalosporinase hyperproducing mutants (Hcase) emergence occurs making them resistant to all penicillins and C3Gs. Thus, expert recommendations advise against the use of C3Gs as monotherapy in this case (1). This risk would be increased for Enterobacter cloacae species. This statement, based on old studies, is now being debated (2). This study aimed at evaluating mutants Hcase emergence when treating Enterobacter cloacae complex wild-type infections in ICU patients with C3Gs.

**Patients and methods/Materials and methods:** Methods: We performed a multicenter retrospective study including microbiological files of patients more than 18, admitted to intensive care unit, between January 2015 and August 2021, and who were treated for wild-type Enterobacter cloacae infection and assessed mutants emergence defined by a subsequent sample finding mutants Hcase emergence whatever clinical evolution.

**Results:** Among the 5275 Enterobacter cloacae species found from the microbiology files during the study period, 373 were associated to infections occurring in ICU patients and finally 165 ICU patients infected with C3Gs susceptible E cloacae were included in the analysis. C3Gs resistance emergence was observed in 19 cases (11.5%), 13 being related to Hcase mechanism and 6 to extended spectrum beta-lactamase (ESBL) production. C3G were used in 56 cases (35%) before identification (n = 12), after identification (n = 18) or exclusively (n = 26). Cefepime was used in 67 cases (41%) before identification (n = 11), after identification (n = 28), exclusively (n = 21), or combined with a fluoroquinolone (n = 3), an aminoglycoside (n = 3) or sulfamethoxazole (n = 1). Mutants Hcase emergence occurred most often after an exclusive treatment by cefotaxime (n = 5), cefepime (n = 2), tazocillin (n = 3). Three were observed following tazocillin/aminoglycoside association (n = 1), tazocillin followed by sulfamethoxazole (n = 1) or cefepime/fluoroquinolone association (n = 1). None was observed after carbapenem use. Considering patients receiving monotherapy following identification, mutants Hcase emergence was more frequent in the C3G group than in the cefepime group (11,4 vs 4% respectively, (p = 0.3).

**Conclusion:** Enterobacter cloacae mutants Hcase emergence could be more frequent following C3G use than cefepime. The data reported here are not significantly different but are obtained on a limited population. A prospective study would be necessary to answer this question.

**Reference 1:** 1. 2021. CASFM / EUCAST AVRIL 2021 V1.0. Société Française de Microbiologie. https://www.sfm-microbiologie.org/2021/04/23/casfm-avril-2021-v1-0/. Retrieved 23 January 2022.

**Reference 2:** 2. Mizrahi A, Delerue T, Morel H, Le Monnier A, Carbonnelle E, Pilmis B, Zahar JR. 2020. Infections caused by naturally AmpC-producing Enterobacteriaceae: Can we use third-generation cephalosporins? A narrative review. International Journal of Antimicrob.

**Compliance with ethics regulations:** Yes in clinical research.

### FC-058 Anticoagulation, thromboembolic and bleeding events in COVID-19 VV-ECMO patients in the French Territories in the Americas

#### DAOUD Laura^1^, VALLY Shazima^1^, KALLEL Hatem^2^, CHAPLAIN Agathe^1^, FLORENTIN Jonathan^1^, MEGARBANE Bruno^3^, MEHDAOUI Hossein^1^, VALENTINO Ruddy^1^, RESIERE Dabor^1^

##### ^1^Centre Hospitalier Universitaire, Fort-De-France, Martinique; ^2^Cayenne General Hospital, French Guiana, Cayenne, Guyane Francaise; ^3^Lariboisière Hospital, Paris University; Paris, France

###### Correspondence: Dabor RESIERE (dabor.resiere@chu-martinique.fr)

*Annals of Intensive Care* 2022, **12(1):**FC-058

**Rationale:** Since December 2019, COVID-19 has affected more than 400 million people and caused more than 5 million deaths, causing a truly global pandemic. While most people with COVID-19 will have a minor or moderate form of the disease, some people will develop severe acute respiratory distress syndrome (ARDS), requiring admission to intensive care and the use of mechanical ventilation (VM) and sometimes venovenous extracorporeal membrane oxygenation (VV-ECMO). However, COVID-19 has been associated with an increased risk of thromboembolic and bleeding events, and heparin resistance during VV-ECMO treatment in critically ill patients admitted to the intensive care unit (ICU). The therapeutic target for anticoagulation under VV-ECMO is not really known. The anticoagulant treatment of choice according to the ELSO recommendations is unfractionated heparin with anti-Xa targets between 0.2 and 0.4. The objective of this cohort study was to evaluate the association between severe COVID-19 infection and the occurrence of thromboembolic and bleeding complications following VV-ECMO.

**Patients and methods/Materials and methods:** We conducted a monocenter retrospective observational study in the ICU department of the University Hospital of Martinique from January 2020 to December 2021. Our center has an on-call ECMO mobile unit (UMAC) which includes a cardiothoracic surgeon and a perfusionist nurse. Requests for VV-ECMO French West Indies (Guadeloupe, Martinique, and French Guyana) are regulated by the intensivist on duty at the Martinique University Hospital.

**Results:** We included the records of 40 patients with 25 COVID-19 patients and 15 non-COVID-19 patients over the period January 2020 to December 2021. Six non-COVID-19 patients were excluded: 3 for deaths within 24 h, one patient with acute myeloid leukemia type 3, and 2 patients with a contraindication to heparin therapy. The median age was 46 years old for COVID-19 patients et 49 year old for non-COVID-19 patients. COVID-19 patients experienced thromboembolic complications in 55% of cases (11) and non-COVID-19 patients in 24% of cases (2), p = 0.1

**Conclusion:** Our study confirms that the management of anticoagulation in VV-ECMO represents a particular challenge in COVID-19 patients who appear to be at increased risk of thromboembolic risk complications without sparing the bleeding consequences of the assistance. On the other hand, the difference in thromboembolic and hemorrhagic complications in our two groups was not significant.

**Compliance with ethics regulations:** Yes in clinical research.

### FC-059 Bilateral acral ischemia in COVID-19 patients: a peculiar symptom and treatment options

#### SGHAIER Ameni^1^, RHAIEM Sirine^1^, JAMOUSSI Amira^1^, AYED Samia^1^, RACHDI Emna^1^, JARRAYA Fatma^1^, BESBES Mohamed^1^, BEN KHELIL Jalila^1^

##### ^1^Hopital Abderrahman Mami, Ariana, Tunisie

###### Correspondence: Ameni SGHAIER (sghaier.ameni@caramail.com)

*Annals of Intensive Care* 2022, **12(1):**FC-059

**Rationale:** Although primarily a respiratory virus, SARS-CoV-2 has shown clear impact on hemostasis, often resulting in hypercoagulability state. Deep vein thrombosis, pulmonary embolism and arterial occlusion have all been increasingly reported and investigated during the COVID-19 pandemic. Few studies have however focused on the occurrence of bilateral acral ischemia (BAI). The aim of our study was to examine the characteristics of COVID-19 patients who developed BAI, as well as the various therapy options that might be offered to them.

**Patients and methods/Materials and methods:** We conducted a retrospective study including patients admitted to our intensive-care unit for acute respiratory distress (ARDS) due to COVID-19 between January and August 2021. Clinical characteristics, laboratory findings and vasopressive treatment doses were recorded and used to compare between patients who developed BAI and those who did not. Fischer bilateral exact test and Mann–Whitney-u test were used for comparison between groups.

**Results:** During the study period, 398 patients were admitted; 238 male and 160 female. Of these patients, 8 developed BAI (7 men). Mean age was 67 years old. Medical history of diabetes was present in 7 patients, and hypertension in all 8 patients. All patients who developed BAI had severe ARDS. Only one patient remained on non-invasive ventilation support at the time of BAI occurrence and until discharge. The main affected sites of BAI were fingers and toes although ear lobules were also involved in 3 patients. The mean time to development of BAI starting from the day of admission was 4.9 days. Among patients who were intubated, the mean time of BAI occurrence after intubation was 1.2 days. 4 patients were not under any vasopressive treatment at the time of BAI development. The mean norepinephrine dose among the other 4 patients was 0.65 mg/h. When compared to the other group of patients, there was no statistically significant difference in gender (p = 0.12), median age (56 vs 67 years old; p = 0.054), baseline prothrombin time levels (p = 0.08), d-Dimer levels (p = 0.13), platelet count (p = 0.22) and blood fibrinogen levels (p = 0.061) when available. Survival rates between the two groups were not statistically significant (196 vs 1 patient; p = 0.068). Anticoagulant treatment using heparin was given to 4 patients, with only one patient improved over the course of 10 days. One patient received thrombolysis for subsequent pulmonary embolism with improvement in less than 2 days.

**Conclusion:** BAI has been increasingly reported in critically-ill COVID-19 patients with no apparent other risk factors to predict its occurrence and limited options for treatment and prevention.

**Compliance with ethics regulations:** N/A.

### FC-060 Comparative profiles of routine and specialized biomarkers of coagulopathy and endotheliopathy in moderate and severe COVID-19 disease: a prospective observational study

#### BOUSQUET Giovanni^1^, LACROIX Romaric^3^, PAHUS Laurie^1^, VALERA Sabine^1^, HEZARD Nathalie^2^, ARNAUD Laurent^3^, ABDILI Evelyne^3^, BAUMSTARCK Karine^4^, CHANEZ Pascal^1^, DIGNAT-GEORGE Françoise^3^, PAPAZIAN Laurent^1^, FOREL Jean-Marie^1^, HRAIECH Sami^1^, MORANGE Pierre^2^, GUERVILLY Christophe^1^

##### ^1^Centre Hospitalier Universitaire de Marseille - Nord, Marseille, France; ^2^Centre Hospitalier Universitaire de Marseille - La Timone, Marseille, France; ^3^Centre Hospitalier Universitaire de Marseille - La Conception, Marseille, France; ^4^Faculté de Médecine de Marseille - La Timone, Marseille, France

###### Correspondence: Giovanni BOUSQUET (giovanni.bousquet@icloud.com)

*Annals of Intensive Care* 2022, **12(1):**FC-060

**Rationale:** Endothelial injury and coagulation activation are prominent axis in coronavirus disease 19 (COVID-19) pathogenesis. However, few studies have compared comprehensive profiles of biomarkers of coagulopathy/endotheliopathy with regard to the initial severity of COVID-19 and the time course of the disease.

**Patients and methods/Materials and methods:** In a prospective longitudinal study, we explored coagulation and endothelial function biomarkers in two cohorts of severe and moderate COVID-19 patients and according to their respective outcomes.

**Results:** We found that Tissue factor pathway inhibition (TFPI), Extracellular vesicles—tissue factor (EV-TF), von Willebrand factor antigen (VWF:Ag) and soluble thrombomodulin (sTM) at admission were associated with disease severity. With regard to outcome, d-dimers, TFPI, EV-TF, VWF:Ag/ADAMTS13 ratio and sTM at admission, at day 3 and at day 7 were associated with death and mechanical ventilation. TFPI with a cut-off value of 37.5 ng/mL had 87% sensitivity and 94% specificity and EV-TF with a cut-off value of 13 fM had 87% sensitivity and 83% specificity. ROC curves analysis for severe outcome was significant with AUC = 0.90 for TFPI and AUC = 0.88 for EV-TF (p < 0.001 for both).

**Conclusion:** We found a specific profile of specialized biomarkers of coagulopathy/endotheliopathy associated with the severe forms of COVID-19 and its clinical outcome. We identified TFPI and EV-TF to be of potential interest in stratifying patients’ risk or to design specific treatment targeting coagulation pathways. Additional studies are warranted to confirm our results.

**Compliance with ethics regulations:** Yes in clinical research.
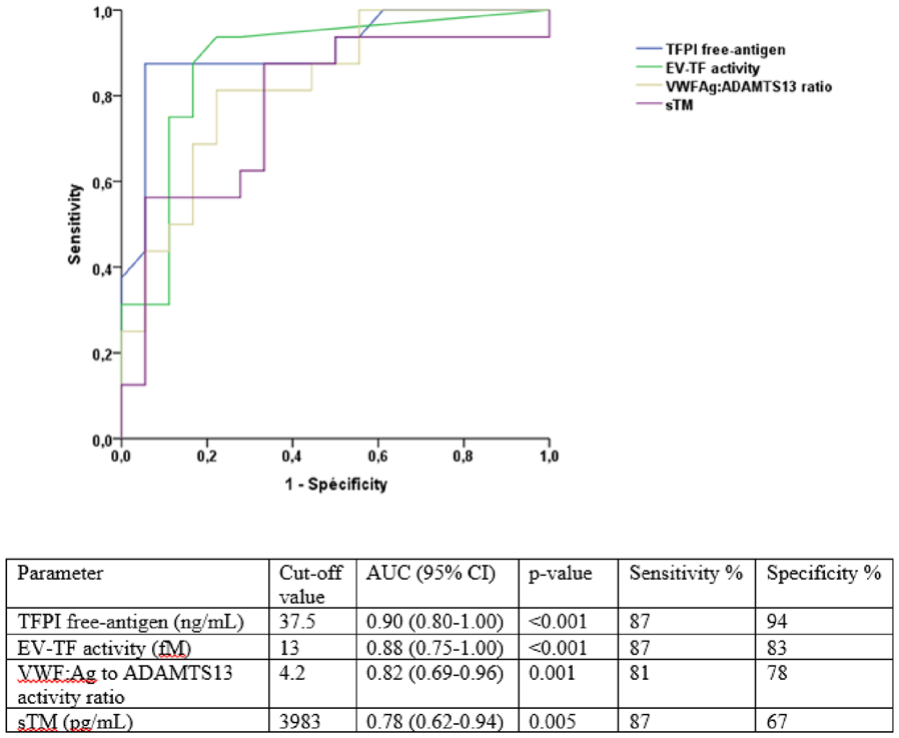


*ROC curves of specialized biomarkers at admission for severe outcome (death or mechanical ventilation) Definition of abbreviations: AUC, area under the curve; TFPI, tissue factor pathway inhibitor; EV-TF, extracellular vesicles—tissue factor; VWF, vo.*

### FC-061 Bedside ACT anticoagulation monitoring in patients infected with SARS-CoV-2 comparing to anti-Xa activity

#### MOUNIR Anass^1^, FALAHI Salma^1^, SAADAOUI Soufiane^1^, CHERKAB Rachid^1^, EL KETTANI Chafik^1^, BARROU Houcine^1^

##### ^1^Réanimation chirurgicale P17, CHU IBN ROCHD, Casablanca, Maroc.

###### Correspondence: Salma FALAHI (salmafalahi73@gmail.com)

*Annals of Intensive Care* 2022, **12(1):**FC-061

**Rationale:** Patients with coronavirus disease 2019 appear to be at high risk for thrombotic disease in both venous and arterial circulations due to excessive inflammation, platelet activation, endothelial dysfunction, and immobility. Anti-Xa and aPTT are prone to monitor therapeutic range of unfractionated heparin of patients with COVID-19 and severe illness. These biological parameters are certainly effective but are not momentary. Hence the idea of proposing ACT (Activated Clotting Time), as an element for monitoring anticoagulation in patients infected with SARS-CoV-2, given its multiple advantages: simple, fast, inexpensive, and which can be done at the bedside.

**Patients and methods/Materials and methods:** We performed a study comparing the results of anticoagulation monitoring by ACT with anti-Xa and TCA activity in 20 patients infected with COVID-19 and treated with curative anticoagulation (LMWH 0.8UIx2/day) over a one month-period. We excluded from the study patients with potentials that could interfere with TCA results: Hypofibrinogenaemia, clotting factor deficiencies, haemodilution, Aprotinin, thrombocytopaenia, qualitative platelet abnormalities, oral anticoagulants.

**Results:** Our results are shown in Table 1. The mean age of the population was 54 ± 10. 50% of the patients were diabetic, 25% were hypertensive, one patient was asthmatic, and one patient had dyslipidemia. 50% of patients had parenchymal involvement ≥ 75%. 35% of patients presented a high risk of thromboembolism. No agreement was found between ACT results and anti-Xa activity in our sample. In contrast, in 95% of cases, there was a linear trend between ACT and TCA results.

**Discussion:** The ACT is routinely used to monitor anticoagulation during cardiopulmonary bypass surgery and endovascular procedures. ACT is determined predominantly by the anti-IIa activity of the anticoagulant. Thus, ACT is higher with heparin or bivalirudin than with LMWH for the same intensity of anticoagulation. However, ACT can be used to monitor LMWH anticoagulation with appropriate adjustments. Several studies have attempted to compare the concordance between ACT results with anti-Xa and TCA activity in patients on low-grade UFH anticoagulation. They have shown that In Intensive care unit patients, no relationship between ACT and either UFH dose, aPTT and anti-Xa was observed. All the studies that were found were in IV anticoagulation with UFH. The literature remains very limited about monitoring by ACT for anticoagulation by LMWH.

**Conclusion:** COVID-19 infection presents a problem with the management of anticoagulation. The biological monitoring parameters are effective but are not momentary. However, bedside ACT anticoagulation monitoring cannot be recommended in patients infected with SARS-CoV-2.

**Compliance with ethics regulations:** Yes in clinical research.
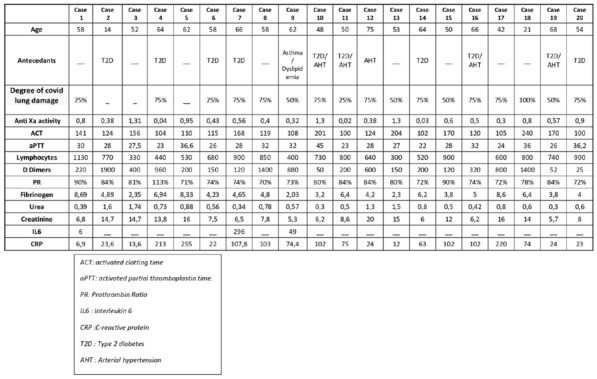


*Table 1: Clinico-biological and radiological parameters of our patients.*

### FC-062 Is there a place to sulodexide in COVID-19 critically ill patients?

#### DHIA Boudour^1,4^, BEN ISMAIL Khaoula^1,4^, ESSAFI Fatma^1,4^, TALIK Imen^1,4^, BAYOUDH Aida^3,4^, FESSI Ilhem^3,4^, CHKIRBENE Mariem^2^, JERBI Tahany^2^, MERHBENE Takoua^1,4^

##### ^1^Service de réanimation Médicale, Hôpital Régional de Zaghouan, Zaghouan, Tunisie; ^2^Département pharmacie, Hôpital Régional de Zaghouan, Zaghouan, Tunisie; ^3^Service de pneumologie, Hôpital Régional de Zaghouan, Zaghouan, Tunisie; ^4^Faculté de médecine de Tunis, Université Tunis El Manar, Tunis, Tunisie

###### Correspondence: Boudour DHIA (bidourabdbg2014@gmail.com)

*Annals of Intensive Care* 2022, **12(1):**FC-062

**Rationale:** Several studies have demonstrated that endothelitis is prominent in COVID-19. Sulodexide (glycosaminoglycan with endothelial protective, vascular anti-inflammatory and antithrombotic activities) was identified to be helpful in hypoxemic patients. The aim of this study was to assess the contribution of sulodexide addition in severe SARS-CoV-2 pneumonia and to determine its impact on prognosis.

**Patients and methods/Materials and methods:** Double-arm interventional clinical trial conducted at the medical intensive care unit (ICU) of the regional hospital of Zaghouan (Tunisia) from April 2021 to December 2021, after approval of the ethics committee. Consenting COVID-19 patients over 18 years were included. Two groups were identified: Group 1 (G1) interventional group and Group 2 (G2) control. Sulodexide (500 LSU) was administered from the first day of ICU hospitalization to discharge for a total of 21 days. Epidemiological and prognostic data were analyzed. The study was conducted anonymously. No conflict of interest to be declared.

**Results:** During the study period, 149 patients were admitted with severe SARS-CoV-2 pneumonia. Seventy-two patients agreed to participate in the clinical trial (G1). The rest defined the control group. On admission, the two groups were comparable in terms of demographic characteristics, clinical presentation and initial severity. Comparison of outcome parameters showed that patients in group 1 had developed less thromboembolic complications (23.1% vs 39.6%, p = 0.016), need for invasive mechanical ventilation (16.8% vs 21.3%, p = 0.031), with lower mortality in G1 (19% vs 36%, p = 0.047). More intra-hospital cardiovascular complications were noted in G2 (37% vs 12% p = 0.03). There were no significant differences in terms of bleeding complications’ occurrence, hemodynamic instability, incidence of healthcare-associated infections, barotrauma complications and length of ICU stay. Multivariate analysis showed that sulodexide use was an independent protective factor against thromboembolic events (OR = 0.57; 95% CI [0.6–0.8]; p = 0.04).Follow-up of patients after three months of discharge showed no difference in terms of cardiovascular complications or post-COVID effects.

**Conclusion:** In this preliminary study, it appears that sulodexide may reduce the risk of thromboembolic and cardiovascular complications in severe COVID-19 patients without affecting the final outcome. Further prospective, multicentre studies with endothelial function studies are needed to confirm this contribution.

**Compliance with ethics regulations:** Yes in clinical research.

### FC-063 Impact of corticosteroids on coagulation profile of critically ill COVID-19 patients—a before-after study

#### GABARRE Paul^1^, URBINA Tomas^1^, CUNAT Sibylle^2^, MERDJI Hamid ^2,3^, BONNY Vincent^1^, LAVILLEGRAND Jean-Rémi^1,4^, RAIA Lisa^1^, BIGE Naike^1^, BAUDEL Jean-Luc^1^, MAURY Eric^1^, GUIDET Bertrand^1^, HELMS Julie^2,3^, AIT-OUFELLA Hafid^1,4^

##### ^1^Hôpital Saint Antoine, Paris, France; ^2^Hôpital civil, Strasbourg, France; ^3^INSERM, UMR 1260, Regenerative Nanomedicine (RNM), FMTS, Strasbourg, France; ^4^INSERM U970, Centre de Recherche Cardiovasculaire de Paris (PARCC), Paris, France.

###### Correspondence: Paul GABARRE (paulgabarre@hotmail.com)

*Annals of Intensive Care* 2022, **12(1):**FC-063

**Rationale:** Several studies have reported an increased risk of thrombotic events in COVID-19 patients, but the pathophysiology of this procoagulant phenotype remains poorly understood. In addition to direct virus-induced endothelial injury, systemic release of pro-inflammatory cytokines may be involved. We hypothesized that corticosteroids may attenuate this procoagulant state through their anti-inflammatory effects.

**Patients and methods/Materials and METHODS:** We conducted a before/after bi-centric cohort study among ICU patients hospitalized for severe COVID-19 and receiving therapeutic anticoagulation by unfractionated heparin (UFH). Before and after the standardized use of dexamethasone (DXM), we compared inflammatory and coagulation profiles, as well as the kinetics of heparin requirement, adjusted for weight and anti-Xa activity.

**Results:** Eighty-six patients were included, 35 in the no-DXM group, and 51 in the DXM group. At admission, CRP and fibrinogen levels were not different between groups, neither were UFH infusion rates. At day 3 after ICU admission, CRP (178 ± 94 mg/L vs 99 ± 68 mg/L, p < 0.0001) and fibrinogen (7.2 ± 1.4 g/L vs 6.1 ± 1.4 g/L, p = 0.0001) significantly decreased in the DXM group, but did not in the no-DXM group. From day 4, UFH infusion rates became lower in the DXM group (Day 4: 435 ± 190 vs 360 ± 96 UI/kg/24 h p = 0.038) without any significant difference in plasma anti-Xa activity. CRP variations correlated with heparin dose variations between Day 0 and Day 3 (r = 0.39, p = 0.009). Finally, the incidence of venous thromboembolic events during in-ICU stay was significantly reduced in the DXM group (4 vs 43%, p < 0.0001).

**Conclusion:** In this before-after bi-centric cohort of critically ill COVID-19 patients, dexamethasone use was associated with a decrease in both pro-inflammatory and procoagulant profile.

**Compliance with ethics regulations:** Yes in clinical research.

### FC-064 Pulmonary embolism in patients with COVID-19 versus non COVID-19 pneumonia

#### TOUJ Hager^1^, SAAD Soumaya^1^, SEDGHIANI Ines^1^, KDEYMI Henedi^1^, AOUAINI Khalil^1^, CHELBI Rym^1^, BORSALI FALFOUL Nebiha^1^

##### ^1^hôpital Habib Thameur, Tunis, Tunisie

###### Correspondence: Hager TOUJ (hagertouj@gmail.com)

*Annals of Intensive Care* 2022, **12(1):**FC-064.

**Rationale:** We aim to compare epidemiological, clinical and prognostic characteristics of patients diagnosed with pulmonary embolism (PE) at the emergency department (ED), which we divided into two groups: patients with confirmed COVID-19 pneumonia versus those without it.

**Patients and methods/Materials and methods:** We conducted a single-centre, retrospective observational study in the period between April 2020 and February 2022. We included patients over 18 years admitted at the ED and diagnosed with PE following computed tomography pulmonary angiography (CTPA). SARS-CoV-2 pneumonia was confirmed by RT-PCR performed in all patients included in our study. Patients were divided into two groups: those affected with COVID-19 Pneumonia and those not affected by it. Patient’s demographics, comorbidities, clinical state and prognosis were collected in both.

**Results:** 144 patients were included. The mean age was 68 ± 15 years with a sex-ratio of 0.9. 79 patients (55%) were diagnosed with confirmed COVID-19 pneumonia. The mean length of stay at ED was 7 days. The clinical characteristics of both groups are presented in Table 1. The intra-hospital mortality rate was 30%.

**Conclusion:** The association of SARS-CoV-2 pneumonia and PE leads to a higher morbidity. Our findings therefore suggest that the phenotype of COVID-19 associated PE indeed differs from PE in patients without COVID-19.

**Compliance with ethics regulations:** Yes in clinical research.
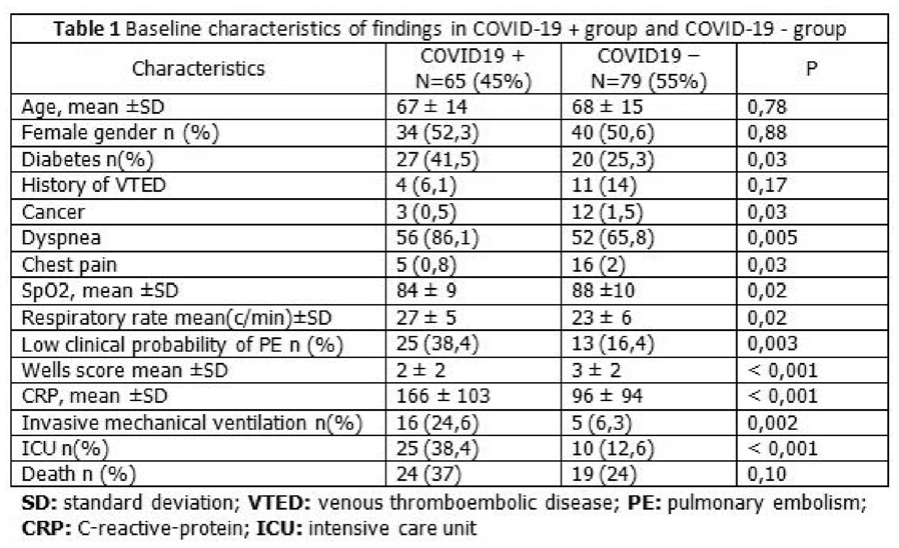


*Table 1 Baseline characteristics in COVID-19* + *group and COVID-19—group.*

### FC-065 Neutrophil phenotypes and microbicidal activity in SARS-CoV-2 associated ARDS patients

#### QUELVEN Quentin^1^, GRÉGOIRE Murielle^2^, COIRIER Valentin^1^, ROUSSEL Mikael^2^, TARTE Karin^2^, TADIÉ Jean-Marc^1^, LESOUHAITIER Mathieu^1^

##### ^1^CHU Rennes, Pontchaillou, Rennes, France; ^2^Unitée MIcroenvironment, Cell differentiation, iMmunology And Cancer (MICMAC), UMR INSERM 1236, Rennes, France

###### Correspondence: Quentin QUELVEN (quentinquelven@hotmail.fr)

*Annals of Intensive Care* 2022, **12(1):**FC-065

**Rationale:** The SARS-CoV-2 infection can lead to a severe acute respiratory distress syndrome (ARDS) with prolonged mechanical ventilation and high mortality rate, sharing clinical and biological features with sepsis-induced immune dysfunction. Since neutrophils play a critical role in host defense against infections, the aim of our study was to investigate phenotypic and functional alterations of neutrophils in patients with SARS-CoV-2 associated ARDS.

**Patients and methods/Materials and methods:** SARS-CoV-2 associated ARDS patients admitted to the ICU, without known immunosuppression, were included. Neutrophil phenotypic maturation and functions (migration, adhesion, Neutrophil Extracellular Trap (NET) release, Reactive Oxygen Species (ROS) production, bacteria/aspergillus killing and phagocytosis) were evaluated within 24 h of ICU admission and at day 7. Furthermore, in a second set of experiments, we investigated the effects of bronchoalveolar lavage (BAL) fluids from SARS-CoV-2 associated ARDS patients on neutrophils.

**Results:** Forty-nine patients were included (Table 1). We found that SARS-CoV-2 associated ARDS induced a systemic inflammation with an increase of circulating mature neutrophils. However, we found a significant decrease in CD66b expression (a marker of granulocyte activation involved in adhesion to endothelial cells, degranulation, and reactive oxygen species (ROS) production). Neutrophil antifungal activity against *Aspergillus fumigatus* was not impaired in patients with SARS-CoV-2 associated ARDS.

**Conclusion:** Although neutrophils ROS production from patients with SARS-CoV-2 associated ARDS was significantly decreased, our results do not support that the increased risk of invasive aspergillosis is linked to neutrophil dysfunction.

**Compliance with ethics regulations:** Yes in clinical research.
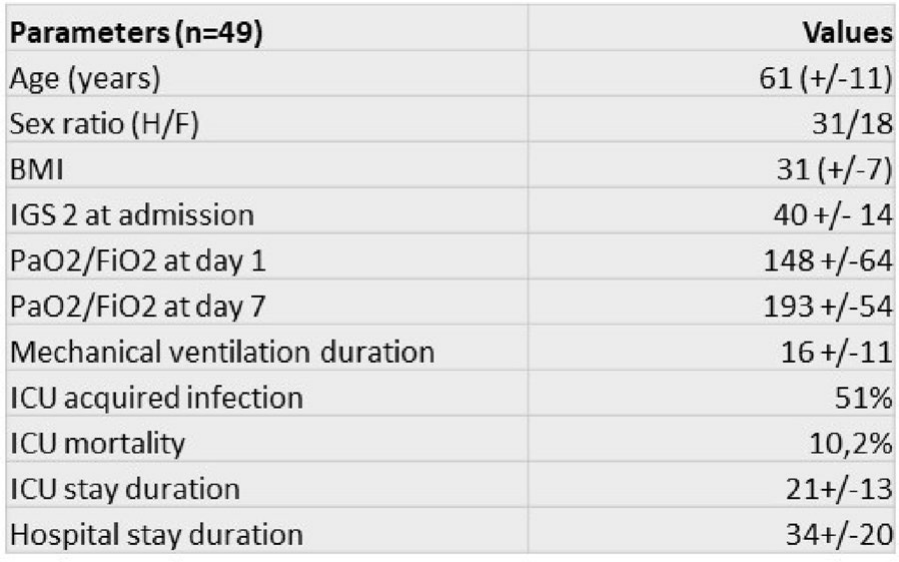


*Table: Demographic data and outcomes of patients admitted to intensive care for SARS-CoV-2 associated ARDS.*

### FC-066 High plasma hyaluronate is associated with mortality in critically-ill patients with SARS-CoV-2 infections

#### BONNY Vincent^1^, URBINA Tomas^1^, ELABBADI Alexandre^2^, GABARRE Paul^1^, MISSRI Louaï^1^, EHRMINGER Sebastien^1^, DESNOS Cyrielle^2^, GUIDET Bertrand^1^, MAURY Eric^1^, VOIRIOT Guillaume^2^, CHANTRAN Yannick^1^, MARIO Nathalie^1^, JOFFRE Jérémie^1^, AIT-OUFELLA Hafid^1^

##### ^1^Hôpital Saint-Antoine, Paris, France; ^2^Hôpital Tenon, Paris, France

###### Correspondence: Vincent BONNY (vincent.bonny@aphp.fr)

*Annals of Intensive Care* 2022, **12(1):**FC-066

**Rationale:** SARS-CoV-2 infection could be responsible for life-threatening severe lung damage with inflammatory cell infiltration, increased vascular permeability and fluid leakage. Hyaluronic acid (HA) is an extracellular matrix polysaccharide, detected in large amounts in lung alveoli in lethal cases of COVID-19 patients. HA which is released by inflammatory cells may be involved in the pathophysiology of alveolar exudate formation through fluid retention. We aimed to evaluate the relationship between plasma HA levels and the outcome of critically ill COVID-19 patients.

**Patients and methods/Materials and methods:** We performed an observational, bi-center study including patients admitted to intensive care unit with a confirmed laboratory SARS-CoV-2 infection, for acute respiratory failure between April 2020 and December 2020. Plasma level of hyaluronan was measured by ELISA.

**Results:** Overall, 120 patients were included in this study, median age was 62 [53–70] years, 25 patients (21%) were female, median SOFA score was 3 [2–6] at ICU admission. 76 patients (63%) required invasive mechanical ventilation. At admission, median PaO_2_/FiO_2_ ratio was 157 [112–221] mm Hg, mortality at day 28 was 20%. Median HA level was 177 [68–356] ng/mL at admission. HA levels were twofold higher in non-survivors (379 [90–914] vs 150 [62–283] ng/mL), p = 0.01) whereas CRP and fibrinogen were not different according to the outcome. HA levels correlated with PaO_2_/FiO_2_ (R = − 0.22, p = 0.02, Pearson test) but did not correlate with CRP.

**Conclusion:** Plasma HA levels were associated with poor outcome in critically ill patients with SARS-CoV-2 infections.

**Compliance with ethics regulations:** Yes in clinical research.

### FC-067 Serum complement C3 and C4 prediction of mortality in critically ill COVID19 patients

#### MESSAOUD Linda^1^, JAMOUSSI Amira^1^, AYED Samia^1^, RACHDI Emna^1^, JARRAYA Fatma^1^, YAALAOUI Sadok^1^, BESBES Mohamed^1^, BEN KHELIL Jalila^1^

##### ^1^hôpital Abderrahmen Mami, Ariana, Tunisie

###### Correspondence: Linda MESSAOUD (lyndamessaoud1991@gmail.com)

*Annals of Intensive Care* 2022, **12(1):**FC-067

**Rationale:** The complement system has many protective effects against infectious agents following activation during innate immunity, through the alternative and lectin pathways, and acquired immunity, through the classical pathway. However, adverse events were observed in COVID-19 following activation of the complement system by the prolonged release of pro-inflammatory mediators. We aimed to investigate ability of serum complement C3, C4 to predict mortality in patients with critical COVID-19.

**Patients and methods/Materials and methods:** Prospective study carried in respiratory medical intensive care unit of Abderrahmen Mami teaching hospital. It included critically ill patients diagnosed with COVID-19 between September 2020 and December 2021. Complement C3 and C4 were measured during the first 24 h of hospitalization. C3 and C4 complement deficiency was defined respectively by a concentration below 0.9 g/L and 0.16 g/L respectively. Association with ICU mortality was then analyzed.

**Results:** During the study period, we enrolled 34 patients. Mean age was of 54 ± 13 years and gender ratio of 1.8. The most common comorbidities were diabetes (36%), obesity (35%) and hypertension (18%). Nineteen patients were transferred to general ward or discharged (survival group) and fifteen died in hospital (non-survival group). The condition of 9 of these patients (27%) degraded during hospitalization and needed invasive mechanical ventilation. The time from hospital admission to intubation varied from 1 to 8 days (median 1 day). During the first 24 h, median C3 was 1.72 g/L [0.6–2.61], and C4 was 0.38 g/L [0.06–0.73]. Six patients (19%) had a C3 deficit and 5 (16%) had a C4 deficit on admission. The overall mortality rate was 56% and the mean length of hospital stay was 10 days [6–17]. Patients who died had lower C3 concentrations in comparison to survivors (1.54 vs 1.85 g/L; p = 0.015). However, no significant difference was found in C4 concentrations (0.36 vs 0.40 g/L; p = 0.595). The level of complement C3 was also lower in patients who required invasive mechanical ventilation (1.42 vs 1.87 p = 0.016) compared to the non-invasive ventilation group.

**Conclusion:** Serum complement C3 and C4 deficiency can be observed in critical COVID-19 patients. C3 concentrations was significantly lower among non-survivors and invasively ventilated patients.

**Compliance with ethics regulations:** Yes in clinical research.

### FC-068 Early decrease of CD4 and HLA-DR expression on monocytes predicts the severity of COVID-19 pneumonia

#### ALLARDET-SERVENT Jerome^1^, AIT BELKACEM Ines^2,3^, MILOUD Tewfik^3^, BENAROUS Lucas^1^, GALLAND Franck^2^, HALFON Philippe^1^, MEGE Jean-Louis^4^, BUSNEL Jean-Marc^3^, MALERGUE Fabrice^3^

##### ^1^HOPITAL EUROPEEN MARSEILLE, Marseille, France; ^2^CENTRE D'IMMUNOLOGIE MARSEILLE-LUMINY, Marseille, France; ^3^BECKMAN COULTER IMMUNOTECH, Marseille, France; ^4^INSTITUT HOSPITALO-UNIVERSITAIRE DE MALADIES INFECTIEUSES, Marseille, France

###### Correspondence: Jerome ALLARDET-SERVENT (j.allardetservent@hopital-europeen.fr)

*Annals of Intensive Care* 2022, **12(1):**FC-068

**Rationale: **Early prediction of COVID-19 pneumonia course remains challenging for physicians. Characterization of blood immune cell status may improve patient stratification^1^. This study investigates the value of a rapid one-step flow cytometry method in patients hospitalized for moderate to severe COVID-19 pneumonia.

**Patients and methods/Materials and methods:** Thirty patients with laboratory confirmed COVID-19 pneumonia were prospectively included and compared to 19 healthy controls. End-points of interest were the need for intensive care unit (ICU) admission and mechanical ventilation (MV) initiation. Whole-blood immunophenotyping was performed within the first 48 h of hospital admission using three antibody panels (Beckman Coulter, Brea, USA) focusing on leucocytes (DURAClone IM Phenotyping Basic panel), granulocytes (DURAClone IM Granulocytes panel), and myeloid activation markers (IOTest Myeloid Activation Antibody Cocktail: CD169, CD64, HLA-DR). Leucocyte staining and lysis were performed following the one-step method^2^. Comparisons were performed using Mann–Whitney U test. Receiver operating characteristic (ROC) analysis was used to determine the area under the curve (AUC) of factors associated with ICU admission and MV requirement. Continuous data are presented as median and interquartile.

**Results:** COVID-19 patients were mostly male (87%), aged 61 [50,71] years, and 12 (40%) were obese. Nine COVID-19 patients (30%) received standard oxygen therapy in the ward while 21 (70%) required ICU admission. Among ICU patients, 7 (23%) received high flow oxygen therapy and 14 (47%) required MV of whom 3 also received extracorporeal membrane oxygenation. Four patients (13%) deceased. We confirmed a COVID-19 signature including: 1) a reduction of HLA-DR expression; 2) decreased basophils, eosinophils, T-cells, NK cells, and non-classical monocyte count; and 3) an upregulation of CD169, CD64, the adhesion/migration markers (CD62L and CD11b), and the checkpoint inhibitor CD274. COVID-19 patients requiring MV presented significantly lower expression of HLA-DR and CD4 on monocytes, as well as reduced CD8+ T-cell count. These three parameters predicted MV requirement with respective AUC of 0.87, 0.87 and 0.79 but their combination increased the AUC up to 0.991 (Fig. 1). Similarly, ICU admission was best predicted by the combination of HLA-DR and CD4 on monocytes with CD4+ T-cell count, yielding an AUC of 0.947.

**Discussion:** This study further describes the immunophenotypic alterations associated with COVID-19, emphasizes the role of monocytes, and strengthens the clinical potential of a rapid, one-step, flow cytometry method.

**Conclusion:** HLA-DR and CD4 expression on monocytes, when combined to CD4+ and CD8+ T-cells count, may facilitate the early prediction of ICU admission and MV initiation in hospitalized patients with COVID-19 pneumonia.

**Reference 1:** Chevrier, S et al. A Distinct Innate Immune Signature Marks Progression from Mild to Severe COVID-19. Cell Rep Med 2021, 2 (1), 100166.

**Reference 2:** Ait Belkacem, I et al. One-Step White Blood Cell Extracellular Staining Method for Flow Cytometry. Bio-protocol 2021, 11 (16), e4135–e4135.

**Compliance with ethics regulations:** Yes in clinical research.
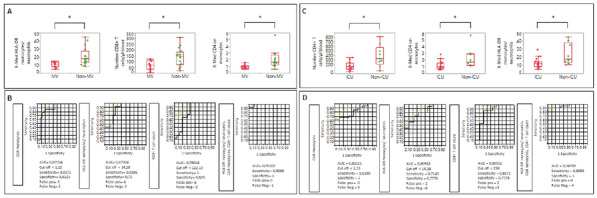


*Immune parameters at hospital admission in COVID-19 patients. A-D: Comparison of HLA-DR and CD4 expression on monocytes and CD4*+ *and CD8*+ *T cell count according to MV or ICU admission. B–D: Individual and combined ROC curves of the three best discriminant.*

### FC-069 Lymphocytic alveolitis in severe COVID-19 pneumonia

#### REYNAUD Faustine^1^, RODRIGUEZ Maeva^1^, ARRIVE François^1^, VEINSTEIN Anne^1^, CHATELLIER Delphine^1^, BOISSIER Florence^1^, FRAT Jean-Pierre^1^, COUDROY Rémi^1^, THILLE Arnaud W.^1^

##### ^1^CHU de Poitiers, Poitiers, France

###### Correspondence: Faustine REYNAUD (faust.reynaud@gmail.com)

*Annals of Intensive Care* 2022, **12(1):**FC-069

**Rationale:** Neutrophil alveolitis has been reported in the early course of mechanically ventilated patients with COVID-19^1,2^. Whereas lymphocytic alveolitis is described in organizing pneumonia, whether this pattern occurs during the course of COVID-19-related respiratory failure is unknown.

**Patients and methods/Materials and methods:** Retrospective observational cohort study performed in a single-center. All patients with COVID-19-related respiratory failure admitted to our ICU between September 2020 and June 2021 (second and third wave) and who had broncho-alveolar lavage (BAL) were included. BAL was performed for secondary worsening or lack of improvement after excluding ventilator-associated pneumonia or pulmonary embolism. Secondary worsening was defined as a one-point increase on the World Health Organization 7-point Clinical Progression Scale (WHO-CPS) occurring after steroid discontinuation and after a first step of improvement (at least one-point drop on the WHO-CPS). Lack of improvement was defined as no point-drop on WHO-CPS after ten days of steroids. The primary outcome was the prevalence of lymphocytic alveolitis defined by a lymphocyte count at least 20% of total cellular count.

**Results:** Among the 182 patients admitted for COVID-19-related respiratory failure over the study-period, BAL was performed in 20 patients with secondary worsening (n = 12) or lack of improvement (n = 8). As compared to the others, patients who underwent BAL were more likely to be intubated (18 of 20 patients, 90% vs. 76 of 162 patients, 47%; p < 0.001), and had longer duration mechanical ventilation (24 days in median [14–39] vs. 10 days [8–15]; p < 0.001). Among the 20 patients who underwent BAL, 10 (50%) had lymphocytic alveolitis including 50% of patients who had BAL for secondary worsening (6 out of 12 patients), and 50% of patients who had BAL for lack of improvement (4 out of 8 patients). All patients with lymphocytic alveolitis received a second course of steroid therapy after BAL. Mortality was 75% in patients with lack of improvement (3 of 4 patients) and 17% (1 of 6 patients) in those with secondary worsening.

**Conclusion:** Lymphocytic alveolitis was found in half of patients who underwent BAL for secondary worsening or lack of improvement and was associated with good outcomes when a second regimen of steroid was administered to patients with secondary worsening.

**Reference 1:** Baron A, Hachem M, Tran Van Nhieu J, Botterel F, Fourati S, Carteaux G, De Prost N, Maitre B, Mekontso-Dessap A, Schlemmer F. Bronchoalveolar lavage in patients with COVID-19 with invasive mechanical ventilation for acute respiratory distress syndrome. An.

**Reference 2:** Cornelissen C, Bergs I, Müller A, Daher A, Kersten A, Balfanz P, Lemmen S, Marx G, Marx N, Dreher M, Müller T. Broncho-alveolar lavage in patients with acute respiratory distress syndrome due to COVID-19. Internal Medicine Journal 51 (2021) 965–967.

**Compliance with ethics regulations:** N/A.

### FC-070 Predictive factors of mortality in critically ill patients with SARS-CoV-2 pneumonia: a prospective study of 301 patients

#### MORATELLI Giulia^1^, REGAIEG Kais^1^, NAKAA Sabrine^1^, FRAJ Nesrine^1^, KALLEL Myriam^1^, AL HARACH Amir^1^, GAZAIGNE Laure^1^, GOLDGRAN-TOLEDANO Dany^1^

##### ^1^Groupe Hospitalier Intercommunal Le Raincy Montfermeil, Montfermeil, France

###### Correspondence: Giulia MORATELLI (giulia.moratelli@gmail.com)

*Annals of Intensive Care* 2022, **12(1):**FC-070

**Rationale:** We want to describe the epidemiological, clinical and outcome of critically ill patients with SARS-CoV-2 pneumonia and to identify risk factors for in-hospital mortality in these patients.

**Patients and methods/Materials and methods:** We conducted a prospective cohort study of critically ill adults admitted into ICU from Mars 2020 to February 2022 for SARS-CoV-2 pneumonia.

**Results:** A total of 301 patients were admitted into our ICU for acute respiratory failure due to SARS CoV-2 pneumonia. Mean age was 62 years (25–86). Mean delay to ICU hospitalization was 8 (1–27) days and mean duration of ICU stay was 8 (1–149) days. Mean SAPS was 32 (12–75). Sex ratio (M/F) was 2. Mean body mass index was 30 (18–68). 169patients (56%) had hypertension, 100 (33%) were diabetics and 33 (11%) had chronic kidney diseases. Within the study period, 258 patients (86%) have needed non-invasive ventilation and 250 (83%) had high flow nasal oxygen as first line therapy.143 patients (48%) received mechanical ventilation for a mean duration of 12 days (1–47) and 67 (47%) of them were extubated. 129 patients (43%) needed catecholamine, 41 (14%) renal replacement therapy and 11 (7,7%) ECMO. Different immunomodulator’s therapies were used during study period: 124 patients (41%) received dexamethasone 6 mg/day for ten days, 113 (38%) received dexamethasone 0,1 mg/kg/days for ten days and 26 (8,6%) received bolus of methylprednisolone 250 mg/days for three days as rescue therapy. We have administrated tocilizumab in 22 patients (7,3%). In the univariate analysis, risk factors associated with mortality were age, hypertension, SAPS II, chronic renal diseases, AKI, use of catecholamine and mechanical ventilation. In the multivariate analysis, risk factors associated with mortality were mechanical ventilation and use of catecholamine.

**Conclusion:** Our experience during this pandemic period is similar to the results of most European studies. The use of mechanical ventilation and catecholamine were associated with a poor outcome. More studies are needed on this subject.

**Compliance with ethics regulations:** Yes in clinical research.

### FC-071 Epidemiological and clinical characteristics of 600 COVID-19 patients

#### MERBOUH Manal^1^, EL AIDOUNI Ghizlane^1^, ALKOUH Rajae^1^, TAOUIHAR Salma^1^, ZAID Ikram^1^, EL MEZZIOUI Sanae^1^, BOUABDELLAOUI Amine^1^, AFTISS Fatima^1^, LAARIBI Ilias^1^, CHOUKRI Bahouh^1^, BENCHAIB Rajae^1^, MIMOUNI Hamza^1^, MAARAD Mohammed^1^, MEKKAOUI Ikram^1^, JEBAR Khaoula^1^, OUJIDI Younes^1^, BENSAID Amine^1^, DOUQCHI Badie^1^, CHETOUANI Badie^1^, HOUSSAM Bkiyar^1^, ABDA Naima^1^, HOUSNI Brahim^1^

##### ^1^CHU Mohammed VI, Oujda, Maroc

###### Correspondence: Manal MERBOUH (manal.mrb@gmail.com)

*Annals of Intensive Care* 2022, **12(1):**FC-071

**Rationale:** COVID-19 is a global health crisis. The evolution of the disease is unpredictable with disastrous socio-economic consequences. The aim of the present work is to study the epidemiological, clinical, diagnostic, therapeutic and evolutionary aspects, as well as risk factors associated with SARS-CoV-2 infection in 600 patients, admitted to the intensive care unit of a university hospital during the first wave of the pandemic.

**Patients and methods/Materials and Methods:** This is a retrospective descriptive and analytical study conducted on patients with COVID-19 between 22 March and 31 December of 2020. Data were collected using computerized medical records on HOSIX and were analyzed by the SPSS software.

**Results:** Of 600 patients, the median age was 64 years (IQR 15–100), 403 patients were male (sex ratio = 2.05), 387 patients had a BMI above normal, 191 had a history of hypertension, 188 had a history of diabetes. Fever, cough, dyspnea were the predominant symptoms, 379 patients had more than 50% lung involvement on chest CT. The majority of patients had desaturation on admission. Respiratory distress, lymphopenia, thrombocytopenia, elevated CRP and hyperglycemia were significantly associated with an unfavorable outcome. The management was based on synthetic antimalarials associated with azithromycin, corticosteroids, ventilatory support as well as specific management of complications with a good evolution in 67.5% of cases, mean duration of hospitalization in the intensive care unit was 8.2 days varying from 2 to 49 days.

**Conclusion:** The results of our study are consistent with other series. COVID 19 remains a malignant disease if management is delayed and leads to the development of several complications. Barrier measures remain the best means of prevention against covid-19. Mass vaccination to acquire herd immunity is the way to control this infection.

**Compliance with ethics regulations:** Yes in clinical research.

### FC-072 Prognosis of critically ill patients with acute respiratory failure due to the SARS-CoV-2 501Y.V2 variant: a multicenter retrospective matched cohort study

#### PUECH Bérénice^1^, LEGRAND Antoine^1^, SIMON Olivier^1^, COMBE Chloé^1^, JAFFAR-BANDJEE Marie-Christine^1^, CARON Margot^1^, VIDAL Charles ^1^, MAVINGUI Patrick^1^, BLONDE Renaud ^1^, BOUE Yvonnick^1^, BERGUIGUA Hamza^1^, ALLYN Jerome^1^, FERDYNUS Cyril^1^, ALLOU Nicolas^1^

##### ^1^CHU Felix Guyon, Saint Denis, Reunion

###### Correspondence: Bérénice PUECH (berenice.puech@gmail.com)

*Annals of Intensive Care* 2022, **12(1):**FC-072.

**Rationale:** The aim of this study was to compare the prognosis of patients with acute respiratory failure (ARF) due to the severe acute respiratory syndrome coronavirus 2 (SARS-CoV-2) variant 501Y.V2 to that of patients with ARF due original strain.

**Patients and methods/Materials and methods:** This retrospective matched cohort study included all consecutive patients who were hospitalized for ARF due to SARS-CoV-2 in our University Hospital between March 2020 and April 2021. Twenty-eight in hospital mortality was evaluated before and after matching.

**Results:** A total of 218 patients with ARF due to SARS-CoV-2 were enrolled in the study. Of these, 83 (38.1%) were infected with the 501Y.V2 variant. During intensive care stay, 104 (47.7%) patients received invasive mechanical ventilation and 20 (92%) patients were supported by venovenous extracorporeal membrane oxygenation. Patients infected with the 501Y.V2 varient were younger (58 [51–68] versus 67 [56–74] years old, p = 0.003), had less hypertension (54.2% vs 68.1%, p = 0.04), and had less chronic kidney disease (13.3% vs 31.9%, p = 0.002) than patients infected with the original strain. After controlling for confounding variables (62 matched patients in each group), 28-day mortality was higher in the group of patients infected with the 501Y.V2 variant (30.6%) than in the group of patients infected with the original strain (19.4%, p = 0.04).

**Conclusion:** In our department, where SARS-CoV-2 incidence remained low until February 2021 and the health care system was never saturated, mortality was higher in patients with ARF infected with the 501Y.V2 variant than in patients infected with the original strain.

**Compliance with ethics regulations:** Yes in clinical research.

### FC-073 Characteristics and outcome of critically ill COVID-19 patients

#### HADDED Amina^1^, ALILA Ilef^1^, KHARRAT Sana^1^, TURKI Olfa^1^, BAHLOUL Mabrouk^1^, BOUAZIZ Mounir^1^

##### ^1^hopital Habib Bourguiba Sfax, Sfax, Tunisie

###### Correspondence: Ilef ALILA (ilefalila1323@gmail.com)

*Annals of Intensive Care* 2022, **12(1):**FC-073

**Rationale:** The Covid-19 pandemic has hit our country as well as countries around the world since December 2019 with its beginning in Wuhan, China. This virus has threatened the life of human beings by attacking various organs, causing especially respiratory distress requiring hospitalization in intensive care unit and causing significant mortality. The aim of this study was to describe the clinical characteristics and outcome of patients with coronavirus disease-2019 (COVID-19) hospitalized in intensive care unit (ICU).

**Patients and methods/Materials and methods:** We conducted a retrospective study in a medical ICU over a period of 16 months [September 2020–December 2021] including patients with SARS-COV2 infection.

**Results:** During the study period, 586 patients were included with a mean age of 59.5 ± 14.7 years and a sex ratio of 1.6. The median SAPSII and SOFA score were respectively of 29 ± 14.8 and 4 ± 2.6. Most patients had comorbidities, including hypertension (36%), obesity (32.1%) and diabetes mellitus (36.2%). A total of 419 (71.5%) patients had severe acute respiratory distress syndrome (ARDS). Severe lung damage ranging between 50 and 75% of the lung parenchyma was estimated in 175 patients (30%). 117 (20%) had lung parenchyma damage ≥ 70%. Invasive mechanical ventilation was required in 264 patients (45.1%) for a median of 3 days. During the ICU stay, vasopressor therapy was required in 232 patients (39.6%). Thromboembolic complications were found in 32 (5.5%) patients. Pulmonary embolism was diagnosed in 22 patients (3.8%) and thrombophlebitis in 10 (1.7%). 239 patients (40.8%) developed renal failure during their hospitalization, and only 37 (6.3%) required hemodialysis. Median ICU length of stay was 6 [IQR 3–10] days and overall mortality was 49% (287 patients). Multivariate logistic analysis showed that an age older than 60 years, CRP value > 75 mg/l at admission, invasive mechanical ventilation, occurrence of infection or acute kidney injury were independent predictors of mortality in patients with severe COVID-19.

**Conclusion:** In this cohort of patients admitted to the intensive care unit, COVID-19 pneumonia was associated with high morbidity and mortality rates. The main clinical predictors were age, CRP value at admission, invasive mechanical ventilation, occurrence of infection or acute kidney injury.

**Compliance with ethics regulations:** Yes in clinical research.

### FC-074 Epidemiology and demographic profile characteristics and outcome of covid-19 patients in intensive care unit at the medical emergency department

#### TABET AOUL Nabil^1,2^, ALACHAHER Djamel^1,2^, GOULMANE Mourad^1,2^, OUALI DADA Soufiane^1,2^, SIDI AISSA Nabil^1,2^, AZZA Mohammed^1,2^, BOUDADI Hajer^2^

##### ^1^Faculty of Medicine of Oran, University Oran 1, Oran, Algerie; ^2^Medical Emergency Department, University Hospital Center of Oran, Oran, Algerie

###### Correspondence: Nabil TABET AOUL (tabetrea@yahoo.fr)

*Annals of Intensive Care* 2022, **12(1):**FC-074

**Rationale:** The recent emergence of COVID-19 has confronted us with an unprecedented global health crisis with a saturation of hospitals, particularly in ICUs and consequently a rate of very high mortality. Our first priority is to improve the prognosis particularly with early management. The aim of this study was to evaluate the characteristics of COVID-19 patients admitted to ICUs and prognosis factors.

**Patients and methods/Materials and methods:** We conducted a retrospective analysis of COVID-19 patients’ mortality and predictive factors of mortality in ICU at the medical emergency department of UHC of Oran in Algeria between 01/01/2021 au 01/01/2022. Non-survivors were compared to survivors using multivariable analysis. We analyzed age, comorbidities, vaccination status, CT scan results, D-dimer rate at admission and invasive ventilation.

**Results:** During this period between January 2021, to January, 2022, 223 COVID-19 patients required ICU admission with a peak of 26,9% patients in July and august. In these patients with confirmed COVID-19, the sex ratio was 0.66, patients were older than 60 years in the majority of cases against only 4% under 40 years, and the majority of patients was Unvaccinated (those who have not received any dose of a COVID-19 vaccine). The presence of comorbidities (86%), the peribronchovascular distribution of lesions, the number of zones involved, and especially percentage greater than 50% in 55% cases and D-dimer greater than 1000 (62%) were associated with increased risk of ICU admission. The mortality of this group was higher (66%). Non-survivors were older and had more frequently various comorbid diseases (56% of patients were hypertensive and 43% were diabetic) than survivors. The use of invasive mechanical ventilation was independently associated with an increased mortality in ICU using the multivariable analysis.

**Conclusion:** In our cohort, patients with the higher mortality were older and had more frequently various comorbid diseases and received invasive mechanical ventilation despite adequate and early management. The initial chest CT scan can predict COVID-19 positivity, ICU admission, mortality, and disease severity.

**Compliance with ethics regulations:** N/A.

### FC-075 Derivation and validation of the CERES score for the prediction of late intubation in Covid-19

#### GAUDET Alexandre^1,2^, GHOZLAN Benoit^1^, DUPONT Annabelle^3,4^, PARMENTIER—DECRUCQ Erika^1,2^, BAYON Constance^1^, TSICOPOULOS Anne^2^, SUSEN Sophie^3,4^, POISSY Julien^1,5^

##### ^1^CHU Lille, Department of Intensive Care Medicine, Critical Care Center, Lille, France; ^2^Univ. Lille, CNRS, Inserm, CHU Lille, Institut Pasteur de Lille, U1019-UMR9017-CIIL-Centre d'Infection et d'Immunité de Lille, Lille, France; ^3^CHU Lille, Service D'hémostase et Transfusion, Centre de Biologie Pathologie, Lille, France; ^4^Univ. Lille, Inserm, CHU Lille, Institut Pasteur de Lille, U1011 EGID, Lille, France; ^5^Univ. Lille, Inserm U1285, CHU Lille, CNRS, UMR 8576, UGSF, Unité de Glycobiologie Structurale et Fonctionnelle, Lille, France

###### Correspondence: Alexandre GAUDET (alexandre.gaudet@chu-lille.fr)

*Annals of Intensive Care* 2022, **12(1):**FC-075

**Rationale:** Predictive scores assessing the risk of respiratory failure in Covid-19 mostly focused on the prediction of early intubation (1). A combined assessment of clinical parameters and biomarkers of endotheliopathy could allow to predict late worsening of acute respiratory failure (ARF) subsequently warranting intubation in Covid-19 (2).

**Patients and methods/Materials and methods:** Retrospective single-center derivation (N = 92 subjects) and validation cohorts (N = 59 subjects), including severe Covid-19 patients with non-invasive respiratory support for at least 48 h following ICU admission. We used stepwise regression to construct the Covid endothelial and respiratory failure (CERES) score in a derivation cohort, and secondly assessed its accuracy for the prediction of late ARF worsening requiring intubation within 15 days (D15) following ICU admission in an independent validation cohort.

**Results:** Platelets, fraction of inspired oxygen and endocan measured on ICU admission were identified as the top 3 predictive variables for late ARF worsening and subsequently included in the CERES score. The area under the ROC curve of the CERES score to predict late ARF worsening was respectively calculated in the derivation and validation cohorts at 0.834 and 0.780 (Fig. 1A). A CERES value ≥ 140 showed the best sensitivity to predict late ARF worsening on D15, observed respectively at 1 and 0.93 in the derivation and validation cohorts, while specificity was found at 0.48 and 0.44, respectively. Further, the best specificity for the prediction of late ARF worsening on D15 was observed for a CERES value ≥ 333, being then respectively found at 0.9 and 0.93 in the derivation and validation cohorts, while sensitivity was measured at 0.45 and 0.5 with this cut-off (Fig. 1B).

**Discussion:** Two distinct clinical applications may be derived from our results. First, we found that a CERES value ≥ 140 has a very high sensitivity for the detection of patients eventually requiring invasive respiratory support. Accordingly, the non-invasive strategy was successful in nearly all patients with a CERES score < 140. We could therefore propose to maintain non-invasive strategies in patients with a CERES score < 140. In the same view, a CERES score ≥ 333, which detected the further need for invasive support with a high specificity, could be used to propose earlier intubation in patients with the highest risk of failure of non-invasive strategies.

**Conclusion:** In patients with severe Covid-19 undergoing non-invasive respiratory support, CERES values < 140 and ≥ 333 appear as accurate predictors of late worsening of ARF requiring subsequent intubation.

**Reference 1:** Prakash J, Bhattacharya PK, Yadav AK, Kumar A, Tudu LC, Prasad K. ROX index as a good predictor of high flow nasal cannula failure in COVID-19 patients with acute hypoxemic respiratory failure: A systematic review and meta-analysis. J Crit Care. 2021 Dec;

**Reference 2:** Dupont A, Rauch A, Staessens S, Moussa M, Rosa M, Corseaux D, et al. Vascular Endothelial Damage in the Pathogenesis of Organ Injury in Severe COVID-19. Arterioscler Thromb Vasc Biol. 2021 May 5;41(5):1760–73.

**Compliance with ethics regulations:** Yes in clinical research.
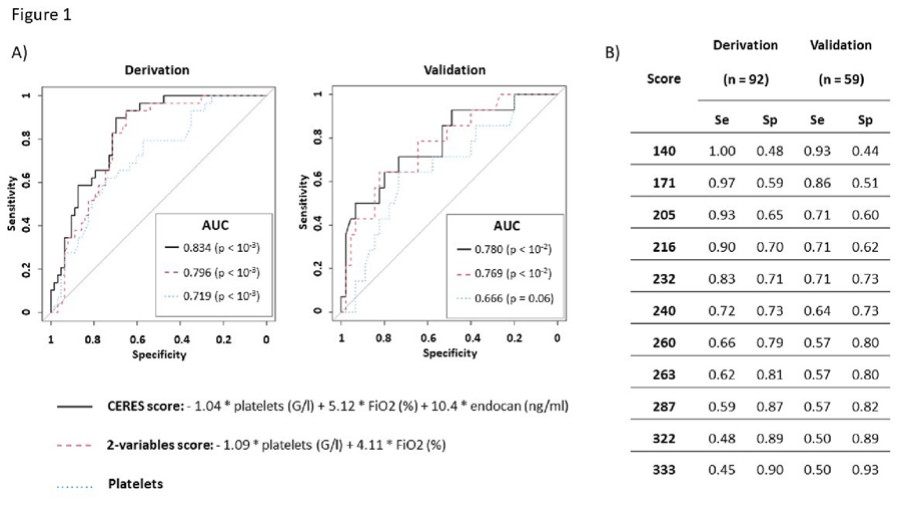


*Figure 1. ROC curves for the prediction of late ARF worsening at D15 in the derivation and validation cohorts. A) ROC curves were computerized in derivation and validation cohorts for the CERES score (black plain line), for the top 2 predictive variables.*

### FC-076 Early vs late intubation in COVID-19 acute respiratory distress syndrome

#### MESSAOUD Lynda^1^, JARRAYA Fatma^1^, RACHDI Emna ^1^, JEMMALI Rim^1^, JAMOUSSI Amira^1^, AYED Samia^1^, BEN KHELIL Jalila^1^

##### ^1^Hôpital Abderrahmane Mami, Ariana, Tunisie

###### Correspondence: Fatma JARRAYA (fatma.jarraya8@gmail.com)

*Annals of Intensive Care* 2022, **12(1):**FC-076

**Rationale:** Although management of acute respiratory failure with coronavirus 2019 disease often involves the use of mechanical ventilation, the optimal time to initiate invasive mechanical ventilation (IMV) remains unknown. The aim of this study was to assess the impact of the timing from admission to intubation on mortality in patients with SARS-CoV-2 acute respiratory distress syndrome (ARDS).

**Patients and methods/Materials and methods:** This retrospective study was conducted in an intensive care unit (ICU) between March 2020 and September 2021. We included adult patients with ARDS secondary to SARS-CoV-2 who required IMV. We did not include patients who were intubated prior to admission. The median time from admission to intubation was 72 h. We defined “early” intubation as intubation within 72 h of ICU admission and “late” intubation as intubation occurring at any time after 72 h. Patients were divided into two groups: early intubation (G1) (≤ 72 h) or late intubation (G2) (> 72 h) for analysis.

**Results:** During the study period, 298 patients required IMV of which 175 were intubated early (58.7%) and 123 were intubated late (41.3%). The two groups were comparable in terms of age (61 ± 12 years vs 63 ± 11 years; p = 0.086, G1, G2 respectively) and gender ratio (1.69 vs 2.41, G1, G2 respectively). However, patients in G1 had higher SAPSII at admission (32.3 ± 11 vs 28 ± 7; p = 0,002). Patients in the G1 had a worse PaO_2_/FiO_2_ ratio (median, 100 mm Hg vs 130 mm Hg; p = 10–3), at the time of intubation in comparison with the G2. Lower static compliance (22 ml/cm H_2_O vs 28 ml/cm H_2_O; p = 0.562) and higher plateau pressure (29 cm H_2_O vs 22 cm H_2_O; p = 0.180) were noted in the G2, although these values were not statistically significant. The length of stay in the ICU was similar between both groups (8 days [4–15] vs 13 days [9–18]; p = 0.156). Even among survivors who were successfully weaned from mechanical ventilation (n = 25; 8.4%), length of stay was similar between both groups (30 days [12–45] vs 31 days [18–41]; G1, G2 respectively; p = 0.635). There was no significant difference in mortality between patients in G1 and G2 (57.5% versus 42.5% p = 0.159). The overall mortality rate was 91.6% and the average length of hospital stay was 11 days [6–17].

**Conclusion:** An early intubation strategy was not associated with decreased mortality and morbidity compared to late intubation in patients with ARDS due to SARS-CoV-2.

**Compliance with ethics regulations:** Yes in clinical research.

### FC-077 Correlation between chest electrical impedance variation and tidal volume in mechanically ventilated patients

#### JOUSSELLIN Vincent^1,2^, JANIAK Vincent^3,4^, BONNY Vincent^1,2^, BUREAU Come^1,2^, MAYAUX Julien^1^, MORAWIEC Elise^1^, TALLEC Gwendoline^4^, SALEEM Umar^4^, PINNA Andrea^3^, DRES Martin^1,2^

##### ^1^AP-HP. Sorbonne Université, Hôpital Pitié-Salpêtrière, Service de Médecine Intensive – Réanimation (Département "R3S"), Paris, France; ^2^Sorbonne Université, INSERM, UMRS1158 Neurophysiologie respiratoire expérimentale et clinique, Paris, France; ^3^Sorbonne Université, CNRS, LIP6, Paris, France; ^4^Bioserenity, Paris, France.

###### Correspondence: Vincent JOUSSELLIN (vincent.joussellin@gmail.com)

*Annals of Intensive Care* 2022, **12(1):**FC-077

**Rationale:** Chest Electrical Impedance Tomography (EIT) is a non-invasive technique that produces continuous cross-sectional images of regional lung ventilation. Tidal impedance variation has been recently proposed to estimate the tidal volume of patients with acute respiratory failure by using a calibration factor given by the ratio of tidal volume divided by tidal impedance variation (1). Whether this calibration factor can be used in all patients is unknown. This study hypothesized that K-factor varies between patients.

**Patients and methods/Materials and methods:** We took the opportunity of a study evaluating chest electrical impedance tomography in mechanically ventilated patients. EIT was connected to the ventilator and a 5-min recording was performed while patients were still under pressure support. Median chest impedance variation and median tidal volume were calculated through 100 respiratory cycles over the 5-min period for each patient (or less if respiratory rate was below 20 per minute). K-factor was calculated by dividing median tidal volume by median chest impedance variation and was then expressed in mL/arbitrary units.

**Results:** Thirty-four patients were included, but only twenty-six had full EIT and ventilator data available. The median K-factor was 0.16 mL/UA (IQR 0.14–0.23 mL/UA) (Fig. 1). After applicating a Pearson correlation, factors associated with K-factor were height (r2 = 0.15, p = 0.04), weight (r2 = 0.50, p < 0.01), level of PEEP (r2 = 0.40, p < 0.01) and hematocrit (r2 = 0.30, p < 0.01).

**Conclusion:** K-factor largely varies among patients and a single value cannot accurately estimate the tidal volume. K-factor was correlated with other variables than chest impedance variation alone.

**Reference 1:** 1. Mauri T, Turrini C, Eronia N, et al. Physiologic Effects of High-Flow Nasal Cannula in Acute Hypoxemic Respiratory Failure. Am J Respir Crit Care Med. 2017;195(9):1207–1215.

**Compliance with ethics regulations:** Yes in clinical research.
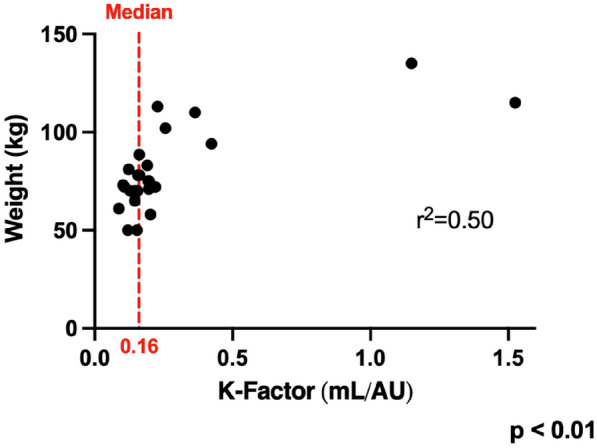


*Correlation between K-Factor and weight in 26 patients (K- factor median is represented with a red dash line).*

### FC-078 Effects of Almitrine administration as rescue therapy in spontaneously breathing patients with severe pneumonia related to SARS-COV-2 treated with high-flow nasal canula oxygen therapy

#### SACCHERI Clément^1^, MORAND Lucas^1^, DOYEN Denis^1^, HYVERNAT Hervé^1^, LOMBARDI Romain^1^, DEVANLAY Raphaël^1^, PANICUCCI Emilie^1^, DELLAMONICA Jean^1^, JOZWIAK Mathieu^1^

##### ^1^CHU de Nice, Nice, France

###### Correspondence: Clément SACCHERI (clement.saccheri@gmail.com)

*Annals of Intensive Care* 2022, **12(1):**FC-078

**Rationale:** Hypoxemia in SARS-CoV-2 pneumonia results from abnormal ventilation/perfusion ratio related to blunted hypoxic vasoconstriction. Almitrine is a selective pulmonary vasoconstrictor that has been reported to improve the oxygenation in mechanically ventilated patients with acute respiratory distress syndrome related to SARS-CoV-2 pneumonia, but its effects in spontaneously breathing patients treated with high-flow nasal canula oxygen therapy (HFNO) as first-line ventilatory support remain to be determined.

**Patients and methods/Materials and methods:** In this single-center, observational and physiological study, a bolus of Almitrine was administered (0.5 mg/kg over 30 min) as rescue therapy in spontaneously breathing patients with severe pneumonia related to SARS-CoV-2 treated with HFNO and with persistent severe hypoxemia (PaO_2_/FiO_2_ ratio < 100 with FiO_2_ > 80%) after awake prone positioning failure. Arterial blood gases and transthoracic echocardiography were performed just before and after the Almitrine bolus. Almitrine responder was defined by an increase in PaO_2_/FiO_2_ ratio > 20%, that was analyzed in light of thoracic CT-Scan patterns (acute fibrinous and organizing pneumonia (AFOP); diffuse alveolar damage (DAD); bi-basal dependent areas of consolidation) obtained at hospital or ICU admission.

**Results:** Overall 62 patients were included (34% of female, 60 ± 11 years old). No patient experienced right ventricular dysfunction or acute core pulmonale after Almitrine administration. Almitrine increased the PaO_2_/FiO_2_ ratio from 68 (61–79) to 109 (85–156) (p < 0.001) and 46 (74%) patients were responders. Almitrine also decreased the PaCO_2_ (33 vs. 31 mm Hg, p < 0.001) while the respiratory rate remained unchanged (27 vs. 28 cycles/min, p = 0.45). The CT-Scan patterns distribution was: AFOP in 36 (58%) patients, DAD in 20 (32%) patients, and bi-basal dependent areas of consolidation in 6 (10%) patients. The proportion of Almitrine responders was similar across the different CT-Scan patterns (75% for AFOP, 70% for DAD and 83% for bi-basal dependent areas of consolidation, respectively). The responders had a lower intubation rate (33 vs. 88%, p < 0.001) and shorter ICU length of stay (5(3–9) vs. 10(6–17), p < 0.01) than non-responder patients.

**Conclusion:** Almitrine iv administration as a rescue therapy in severe COVID-19 pneumonia under HFNO improved oxygenation in responders with a decreased rate of intubation rate. Patterns of the thoracic CT-Scan performed on hospital or ICU admission did not help to predict the response to Almitrine in terms of oxygenation. Further clinical trials are needed to confirm these results.

**Compliance with ethics regulations:** Yes in clinical research.

### FC-079 The use of inhaled nitric oxide (iNO) in Covid19 ARDS patients: a French retrospective registry

#### VIEILLARD BARON Antoine^1^, MEKONTSO DESSAP Armand^2^, PAPAZIAN Laurent^3^, MERCAT Alain^4^, HOUETO Patrick^14^, SCHALLER Manuella^14^, RAMIREZ Juan Fernando^14^, LECOURT Laurent^13^, MEGARBANE Bruno^5^, HAUDEBOURG Luc^6^, TIMSIT Jean-François^7^, TEBOUL Jean-Louis^8^, KUTEIFAN Khaldoun^9^, GAINNIER Marc^10^, SLAMA Michel^11^, NSEIR Saadalla^12^

##### ^1^CH Ambroise Paré (AP-HP), Boulogne Billancourt, France; ^2^CHU de Créteil_Henri Mondor (AP-HP), Creteil, France; ^3^CHU MARSEILLE (APHM), Marseille, France; ^4^CHU d'ANGERS, Angers, France; ^5^CH LARIBOISIERE (AP-HP), Paris, France; ^6^CH Pitié Salpêtrière (AP-HP), Paris, France; ^7^CH BICHAT (AP-HP), Paris, France; ^8^CH BICETRE (AP-HP), Paris, France; ^9^CH de MULHOUSE, Mulhouse, France; ^10^Hôpital La Timone (APHM), Marseille, France; ^11^CHU Amiens, Amiens, France; ^12^CHU Lille, Lille, France; ^13^Air Liquide Healthcare, Gentilly, France; ^14^Air Liquide Healthcare, Les Loges Josas, France.

###### Correspondence: Laurent LECOURT (laurent.lecourt@airliquide.com)

*Annals of Intensive Care* 2022, **12(1):**FC-079

**Rationale:** Inhaled Nitric Oxide (iNO) has been widely used all over the world during COVID19 pandemia^1^. Previous studies reported conflicting results on the effect of iNO on oxygenation improvement. The objective of this registry was to describe the use of iNO in a large cohort of Covid19 ARDS (CARDS) patients.

**Patients and methods/Materials and methods:** Multi-center, retrospective cohort registry conducted in 12 French hospitals during the first year of pandemia on CARDS patients treated with iNO. Patients’ characteristics, clinical respiratory support, nitric oxide therapy safety and efficacy parameters and patients’ clinical outcomes were collected. iNO response was defined as PaO_2_/FiO_2_ ratio improving by > = 20% after iNO initiation.

**Results:** From March to December 2020, 300 CARDS patients (22.3% female) were included in the registry. At ICU admission, their median (IQR) age, SAPS II, and SOFA scores were 66 (57–72) years, 37 (29–48), and 5 (3–8), respectively. Patients were still hypoxemic despite protective ventilation (in all patients) and prone positioning sessions (in 68%). At iNO initiation, 2%, 37%, and 61% patients had mild, moderate, and severe ARDS, respectively. Median (IQR) delay between iNO therapy initiation and ICU admission, ARDS diagnosis, and intubation were 7 (3–12), 6 (2–11) and 4 (1–10) days respectively. The median duration of iNO was 2.8 (1.1–5.5) days with a median dosage of 10 (7–13) ppm at initiation. Responders were analyzed at different stages: 45.7% of patients were responders within 6 h following iNO initiation, 56.9% based on the best PaO_2_/FiO_2_ ratio within 24 h after iNO initiation and 70.3% patients had at least one response during iNO administration. The severity of ARDS is the only predictive factor associated with iNO response considering the best PaO_2_/FiO_2_ ratio obtained within 24 h (Fig. 1). This arterial oxygenation improvement did not impact the patients’ outcomes (length of ICU/hospital stay, ventilation duration, mortality) in the global population. Regarding safety aspects, renal replacement therapy was initiated in 23.5% patients during iNO administration, and 60.5% patients presented a Kdigo score I, II and III within 24 h following iNO initiation, without a formal causal link with iNO.

**Conclusion:** This registry confirmed the benefits of iNO for arterial oxygenation improvement in severe ARDS patients still hypoxemic after optimization of ventilation and prone positioning.

**Reference 1:** Clinical characteristics and day-90 outcomes of 4244 critically ill adults with COVID-19: a prospective cohort study Intensive Care Med. 2020 Oct 29: 1–14. https://doi.org/10.1007/s00134-020-06294-x PMCID: PMC7674575 PMID: 33211135.

**Compliance with ethics regulations:** Yes in clinical research.
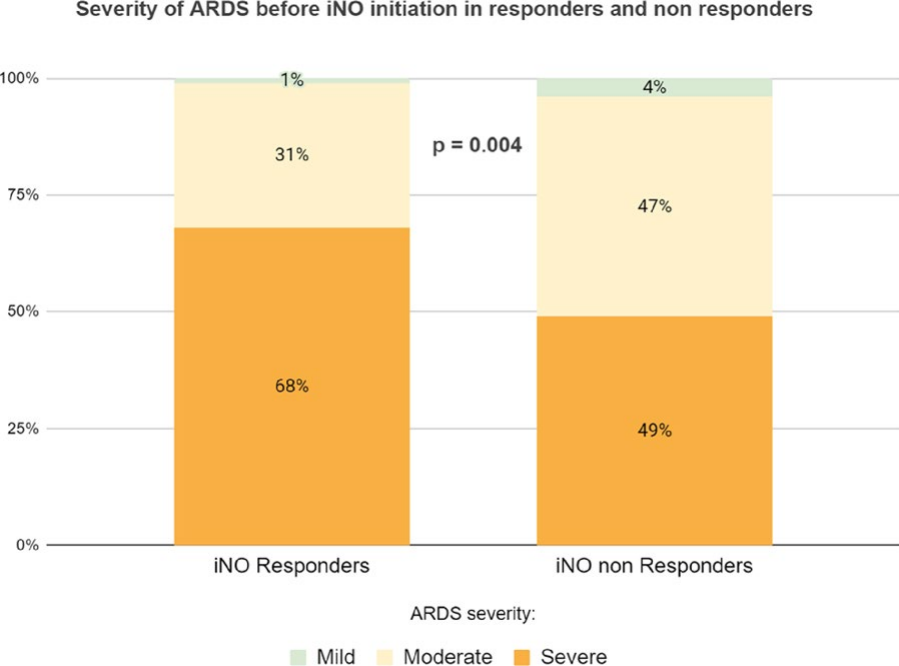


*Severity of ARDS at iNO initiation in iNo responders and non-responders. The distribution of ARDS severity between iNO responders and iNo non-responders was compared using Fisher's exact test.*

### FC-080 Predicting the outcome of patients with acute respiratory distress syndrome using a miniature transesophageal ultrasound probe

#### EDDERKAOUI Mehdi^1^, POULY Olivier^1^, GOUTAY Julien^1^, BOUREL Claire^1^, ONIMUS Thierry^1^, FAVORY Raphael^1^, DURAND Arthur^1^, PREAU Sébastien^1^

##### ^1^CHRU LILLE, Lille, France

###### Correspondence: Mehdi EDDERKAOUI (mehdi-edderkaoui@hotmail.fr)

*Annals of Intensive Care* 2022, **12(1):**FC-080

**Rationale:** The objective of the study was to test whether sequential monitoring of the superior vena cava (SVC), by allowing measurement of the superior vena cava collapsibility index (SVCci), could be a prognostic marker in patients with acute respiratory distress syndrome (ARDS).

**Patients and methods/Materials and methods:** 50 patients admitted to 5 intensive care units (ICU) with moderate to severe ARDS and receiving invasive mechanical ventilation for less than 24 h were prospectively included. The minimum (inspiratory) and maximum (expiratory) telediastolic diameter of the SVC were monitored every 8 h using a miniature ClariTEETM transesophageal ultrasound probe (CLT0110-1, IMACOR, New York NY, USA) for up to 72 h after inclusion. The SVCci was calculated as follows: (maximum diameter − minimum diameter)/maximum diameter. The primary outcome was 90-day mortality.

**Results:** In-hospital mortality censored at 28 and 90 days was of 30% (n = 15) and 34% (n = 17), respectively. The median (interquartile range) length of stay in the intensive care unit (ICU) was 14 days (8–23). The number of days alive without mechanical ventilation at 28 days was 17 (0–23). The mean SVCci measured over the first 24 h after inclusion (SVCci-D1) was significantly higher in survivors compared to non-survivors at 90 days: 0.23 (0.16–0.31) versus 0.17 (0.14 0.21), p = 0.048. The mean SVCci measured over the first 72 h after inclusion (SVCci-D3) was significantly higher in survivors compared to non-survivors at 90 days: 0.22 (0.17–0.24) versus 0.17 (0.14–0.21), p = 0.028. A SVCci-D1 < 0.20 and a SVCci-D3 < 0.22 predicted 90-day mortality with areas under ROC curve (± standard error of the mean) of 0.692 (± 0.079) and 0.692 (± 0.078), sensitivities of 64% and 58%, and specificities of 71% and 88%, respectively.

**Conclusion:** The mean SVCci measured over the first days after ICU admission, appears to be a prognostic marker of 90-day mortality in patients with moderate to severe ARDS.

**Compliance with ethics regulations:** Yes in clinical research.

### FC-081 Assessment of airway opening pressure with electrical impedance tomography in patients with acute respiratory distress syndrome

#### LOUIS Bruno^2^, COUR Martin^1^, ARGAUD Laurent^1^, GUÉRIN Claude^1^

##### ^1^Hôpital Edouard Herriot Hospices Civils de Lyon, Lyon, France, France; ^2^Institut Mondor de Recherches Biomédicales, Créteil, France

###### Correspondence: Claude GUÉRIN (claude.guerin@chu-lyon.fr)

*Annals of Intensive Care* 2022, **12(1):**FC-081

**Rationale:** Airway opening pressure (AOP) determined in patients with acute respiratory distress syndrome (ARDS) from the inspection of volume-pressure curve (1) should mean that lung ventilation increases once AOP is overcome. To address this issue, we compared AOP measured from both inspiratory volume-pressure curve and electrical impedance tomography (EIT) simultaneously recorded during low flow inflation of the respiratory system. If AOP reflects a critical opening pressure it should correlated with AOP measured with EIT (AOP_EIT_).

**Patients and methods/Materials and methods:** In this secondary analysis of a previous study (2) 10 ARDS patients were investigated at positive end-expiratory pressure of 5, 10 and 15 cm H_2_O in the semi-recumbent position under continuous sedation and paralysis. Airway pressure (Paw) and flow signals were measured at the proximal tip of the endotracheal tube and sent to MP 150 datalogger (Biopac Inc.). Pulmovista EIT device (Drager) was used to assess lung ventilation. The respiratory system was inflated at a 7 L/min constant flow inflation delivered by the ventilator (Evita XL) and airway pressure, flow and EIT signals recorded simultaneously. AOP was defined as the pressure where the volume delivered to the patient was 4 ml greater than the volume delivered to the occluded circuit (1). AOP_EIT_ was estimated during low insufflation from Paw-EIT-derived Volume curves fitted by a sigmoidal equation. When the lower inflexion point was below PEEP, AOP_EIT_ was considered equal to PEEP. The values of AOP and AOP_EIT_ were analysed by using the rho Spearman correlation coefficient and the Bland and Altman representation over 30 pairs. P < 0.05 was set as the statistically significant threshold.

**Results:** AOP and AOP_EIT_ were significantly correlated (Spearman rho = 0.73, P = 0.000005). The mean bias was 0.44 cm H_2_O. In 96% (29/30) of the cases the differences observed between the two methods were less than 2 cm H_2_O.

**Conclusion:** AOP and AOP_EIT_ are significantly correlated.

**Reference 1:** (1) Chen et al. AJRCCM 2018.

**Reference 2:** (2) Guérin et al. J Appl Physiol 2020.

**Compliance with ethics regulations:** Yes in clinical research.

### FC-082 Pattern of FRC variation following prone positioning in Covid-ARDS: evaluation by the nitrogen washin-washout method

#### LAHMAR Manel^1^, CHIHAOUI Abir^1^, BEN AHMED Hedia^1^, HAMMOUDA Zeineb^1^, SAADAOUI Oussema^1^, DACHRAOUI Fahmi^1^, ABROUG Fekri^1^, BESBES OUANNES Lamia^1^

##### ^1^CHU Fattouma Bourguiba Monastir, Monastir, Tunisie

###### Correspondence: Manel LAHMAR (firassmal4@gmail.com)

*Annals of Intensive Care* 2022, **12(1):**FC-082

**Rationale:** Acute Respiratory Distress Syndrome (ARDS) is characterized by reduction of all lung volumes (in particular that of FRC), and a decrease in respiratory system compliance (Crs). Prone positioning which is recommended in severe ARDS allows a more uniform distribution of the ventilation/perfusion ratios with alteration in Crs properties resulting in lung recruitment, FRC increase, and improvement of oxygenation and survival. The alveolar recruitment process is time-dependent with uncertainty about the optimal daily prone duration. The aim of our study is to characterize the pattern of FRC increase (kinetic and magnitude) during the first 24 h following prone positioning in patients with Covid-19 related ARDS (C-ARDS).

**Patients and methods/Materials and methods:** a prospective observational study conducted between January and October 2021, including consecutive patients admitted to the ICU for severe C-ARDS (PaO_2_/FiO_2_ ≤ 150 mm Hg) and requiring tracheal intubation and mechanical ventilation. Patients were ventilated in supine position with a lung protective strategy (Vt ≤ 6 ml/kg of P BW, the highest PEEP level to obtain a plateau pressure ≤ 30 cm H_2_O). Patients were systematically turned prone and FRC was measured immediately after prone position and every 2 h during the first 24 h. The measurements were carried out using the Nitrogen Wash-in Wash-out technique available on the CARESCAPE R860 respirator (General Electrics, Wisconsin, USA). The usual oxygenation (SaO_2_/FiO_2_) and ventilatory mechanics (Csr, and strain) variables were also recorded.

**Results:** 20 patients (14 men, mean age: 65 ± 9 years, PaO_2_/FiO_2_: 105 ± 42, mean static compliance: 28.5 ± 5, mean PEEP: 14 ± 2) were included in the study.

**Conclusion:** Prone positioning evokes a rapid and progressive improvement in FRC and oxygenation. These effects are associated with improved strain on the lungs.

**Compliance with ethics regulations:** Yes in clinical research.
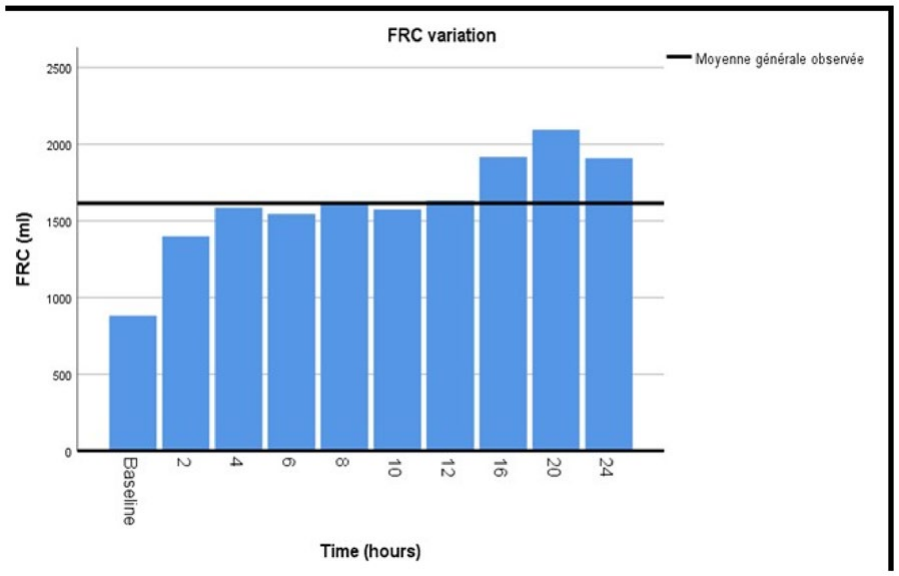


*the FRC variation during the first 24 h following prone positioning*

### FC-083 Regional airway closure and patients positioning in acute hypoxemic respiratory failure

#### PAVLOVSKY Bertrand^1^, LEPROVOST Pierre^1^, LESIMPLE Arnaud^1^, DESPREZ Christophe^1^, TAILLANTOU-CANDAU Mathilde^1^, RICHARD Jean-Christophe^1^, MERCAT Alain^1^, BELONCLE François^1^

##### ^1^CHU Angers, Angers, France

###### Correspondence: Bertrand PAVLOVSKY (bertrand.pavlovsky@gmail.com)

*Annals of Intensive Care* 2022, **12(1):**FC-083

**Rationale:** Airway closure leading to an interruption of ventilation in the downstream alveoli may be associated with ventilation inhomogeneity and ventilation-induced lung injury. This phenomenon may be detected at the bedside by performing a low-flow insufflation pressure–volume or pressure–time curve. Airway opening may vary across the lung. We aimed to study the regional distribution of airway opening in semi-recumbent and strict supine position in a series of patients with acute hypoxemic respiratory failure (AHRF).

**Patients and methods/Materials and methods:** Twenty patients admitted to a Medical ICU and intubated for AHRF (PaO_2_/FiO_2_ ratio < 300 mm Hg) were enrolled within the 12 first hours after intubation. Patients were ventilated in volume-controlled ventilation (tidal volume 6 mL.kg^−1^ predicted body weight PBW) and a positive end-expiratory pressure of 5 cm H_2_O and positioned (1) in the semi-recumbent position (head of bed at 30°) and (2) in the strict supine position (0°). Measurements of end-expiratory lung volume (EELV) using the nitrogen wash-out wash-in technique and reported to PBW, respiratory mechanics, gas distribution using electrical impedance tomography and a low-flow insufflation to detect airway closure were performed after 30 min in each position. One global (obtained with the volume changes measured by the ventilator at the airway opening) and two regional (using regional impedance waveforms to estimate the non-dependent and dependent real-time volume changes) pressure–volume curves were constructed offline using a dedicated software.

**Results:** Age was 63 [52–71]. PaO_2_/FiO_2_ ratio was 240 [140–287] mm Hg, and respiratory system compliance (CRS) was 52 [46–59] mL.cm H_2_O^−1^. Twelve patients fulfilled acute respiratory distress syndrome (ARDS) criteria. An airway closure was detected on the airway pressure–volume curve in 3 patients (15%). A regional airway closure was observed in the dependent regions in 6 patients (30%). No difference in PaO_2_/FiO_2_ (240 [157–317] vs 227 [135–295], p > 0.999) nor CRS (51 [41–57] vs 53 [47–61] mL.cm H_2_O^−1^, p > 0.999) was observed between patients with or without regional airway closure. But EELV/PBW was lower in patients with regional airway closure (16 [15–17] vs 28 [23–34] mL.kg^−1^ PBW, p < 0.001). Supine position was associated with a decrease in EELV/PBW (25 [18–33] vs 21 [16–34] mL.kg^−1^, p = 0.009) and an increase (albeit non-significant) in regional airway closure prevalence (45% vs 30%, p = 0.515).

**Conclusion:** In patients with AHRF, airway closure seems frequent in dependent regions, in particular in strict supine position.

**Compliance with ethics regulations:** Yes in clinical research.
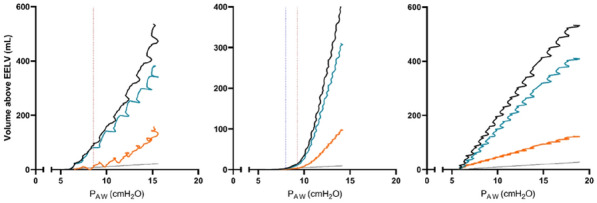


*Examples of airway closure patterns in three representative study patients. Left panel: partial airway closure; central panel: global airway closure, with differencial closure between the regions; right panel: absence of airway closure.*

### FC-084 Corticosteroid for acute exacerbations of COPD in ICU. A French cohort: OUTCOMEREA database

#### GALERNEAU Louis-Marie^1,2^, BAILLY Sébastien^2^, TERZI Nicolas^1^, RUCKLY Stéphane^3^, GARROUSTE-ORGEAS Maité^4^, COHEN Yves^5^, HONG TUAN HA Vivien^6^, GAINNIER Marc^7^, SIAMI Shidasp^8^, DUPUIS Claire^9^, DARMON Michael^10^, AZOULAY Elie^10^, FOREL Jean-Marie^11^, SIGAUD Florian^1^, ADRIE Christophe^12^, GOLDGRAN-TOLEDANO Dany^13^, BRUNEEL Fabrice^14^, DE MONTMOLLIN Etienne^15^, ARGAUD Laurent^16^, REIGNIER Jean^17^, PEPIN Jean-Louis^1,2^, TIMSIT Jean-François^15^

##### ^1^CHU Grenoble Alpes, La Tronche, France; ^2^Université Grenoble Alpes, INSERM U1300 HP2,, Grenoble, France; ^3^Département de biostatistiques, Outcomerea, Paris, France; ^4^Hôpital Franco-Britannique, Levallois-Perret, France; ^5^AP-HP, Hôpital Avicenne, Paris, France; ^6^Grand Hôpital de l'Est Francilien, Meaux, France; ^7^Hôpital de ta Timone, Marseille, France; ^8^Centre Hospitalier Sud Essonne, Étampes, France; ^9^CHU Gabriel-Montpied,, Clermont-Ferrand, France; ^10^AP-HP, Hôpital Saint-Louis, Paris, France; ^11^Hôpital Universitaire Marseille Nord, Marseille, France; ^12^Hôpital Delafontaine Hôpital, Saint-Denis, France; ^13^Groupe Hospitalier Intercommunal Le Raincy Montfermeil, Montfermeil, France; ^14^Centre Hospitalier de Versailles, Le Chesnay, France; ^15^AP-HP, Hôpital Bichat, Paris, France; ^16^Hospices civils de Lyon, Hôpital Edouard Herriot, Lyon, France; ^17^CHU de Nantes, Nantes, France.

###### Correspondence: Louis-Marie GALERNEAU (lmgalerneau@chu-grenoble.fr)

*Annals of Intensive Care* 2022, **12(1):**FC-084

**Rationale:** Acute exacerbations of chronic obstructive pulmonary disease (AECOPD) are a very frequent in intensive care unit (ICU). However, there are few data and conflicting results about corticosteroids therapy for critically ill patients with an AECOPD.

**Patients and methods/Materials and methods:** In this study, using data from the observational longitudinal cohort OutcomeRea™ database, with 32 french ICU centres participating, we assessed the impact of corticosteroid therapy for patients in ICU for an AECOPD. The prescription of corticosteroids therapy at admission was defined as a daily dose ≥ 0.5 mg/kg of prednisone during the first 24 h after admission in ICU. We assessed the effect of corticosteroids on a main composite criteria including death and invasive mechanical ventilation (IMV) at day 28 after admission in ICU using an inverse probability of treatment weight (IPTW) estimator. Secondary outcomes were: survival analyse at D-28 and D-90, NIV failure, length of stay in ICU and in hospital, duration of ventilation, consumption of antibiotics and adverse effects of corticosteroids.

**Results:** We included 1,247 patients of which 31,4% was treated by a corticosteroid therapy at admission. Corticosteroids administration at admission was significantly found as protector on the main composite outcome (RR = 0.693 [0.489; 0.98], p = 0.038). For the subgroup of patients with a very severe COPD, the protective effect of corticosteroid therapy on the primary composite outcome was not found (RR = 1,205 [0.577; 2.517], p = 0.6203). There was no significant impact of corticosteroids on failures of NIV, on lengths of stay in ICU and in hospital or on durations of ventilation. We did not observe statistical relation between the corticosteroid prescription at global prescription of antibiotics. However, corticosteroid therapy tended to be associated with longer duration of antibiotics treatment for patients in ICU at least 10 days (effect of corticosteroid therapy on antibiotic-free days at 10-day for the 395 patients with a length of stay in ICU ≥ 10 days: IRR = 0. 776 [0.597; 1.009], p = 0.059)). Nosocomial infectious had the same prevalence in the two groups of patients. But the maximum systolic blood pressure and the levels of urea was higher and the glycemic disorders more frequent when patient received corticosteroids.

**Conclusion:** A corticosteroids administration at admission in ICU to patients with AECOPD had a protective effect on death or invasive mechanical ventilation at Day 28. Studies on doses to use or the profile of patients to treat are needed.

**Compliance with ethics regulations:** Yes in clinical research.

### FC-085 Use of high-flow oxygen nasal cannula oxygenation in adult patients with sickle cell disease admitted in ICU: a pilot study

#### PONS Bertrand^1^, BERTHOD Antoine^1^, CARLES Michel^2^, MARTINO Frédéric^1^

##### ^1^CHU Guadeloupe, Les Abymes, Guadeloupe; ^2^CHU NICE, Nice, France

###### Correspondence: Bertrand PONS (pons.bertrand@gmail.com)

*Annals of Intensive Care* 2022, **12(1):**FC-085

**Rationale:** Sickle cell disease (SCD) is a life-threatening genetic disorder associated with many chronic and acute complications [1]. Oxygen therapy is one of the cornerstones of management of patients with SCD admitted to the hospital. Recently, high flow nasal cannula (HFNC) oxygenation has emerged, and clinical practice guidelines have been released for the use of HFNC in the overall population [2]. But at this very time, no evidence has been reported of the use of HFNC in patients with SCD. The objective of this pilot study was to evaluate the efficacy and tolerance of HFNC in comparison of conventional oxygen therapy via facial mask (FM) in adult patients with SCD admitted in ICU.

**Patients and methods/Materials and methods:** Consecutive adult patients with SCD (SS, SC, or SBeta thalassemia) admitted in ICU with clinical symptoms compatible with acute chest syndrome (ACS) were eligible. Early-stage ACS and management of all patients included was defined accordingly to the actual national French guidelines. Randomization defined the initial oxygen therapy during a 4-session course of 5-h oxygen therapy with, alternately, HFNC (FiO_2_ 60%, flow rate 60 L/min) and FM (FiO_2_ 60%, flow rate 15 L/min). Blood samples were analyzed at the beginning and end of each session. Comfort, dyspnea, and tolerance were assessed at multiple predefined times during the study. Accordingly, to the French law, written informed consent was obtained from all patients prior to enrollment.

**Results:** Seven patients were included and 24 sessions of oxygen therapy have been completed. There was no significant difference in clinical and biological variables between the beginning (H0) and the end (H5) of oxygen therapy session (Tab 1). Median PaO_2_ throughout the session was higher with HFNC (49 [23, 85] mm Hg) compared to FM (18 [− 26, 30] mm Hg, p = 0.04). Incidence of episodes of desaturation was greater in HFNC vs FM (4 episodes (33%) vs 1 (8%) respectively, p = 0.04) without any episode of severe desaturation (SpO_2_ < 90%). Comfort was statistically non different between the 2 techniques (9.4 [7–10] under FM vs 8.3 [4.7–9.4] under HFNC, p = 0.31).

**Discussion:** In our study, HFNC was associated with a greater benefit in term of oxygenation compared to conventional oxygen therapy. Despite the limitations of this study, our results suggest a potential benefit of HFNC use, in term of oxygenation, in SCD patients with acute respiratory failure.

**Conclusion:** In adult patients with SCD admitted in ICU for acute respiratory failure, HFNC could be of interest to improve oxygenation, compared to conventional oxygen therapy.

**Reference 1:** 1. Yawn BP, Buchanan GR, Afenyi-Annan AN, et al.: Management of sickle cell disease: summary of the 2014 evidence-based report by expert panel members [published correction appears in JAMA 2014; 312(18):1932] [published correction appears in JAMA 2015 Feb].

**Reference 2:** 4. Rochwerg B, Einav S, Chaudhuri D, et al.: The role for high flow nasal cannula as a respiratory support strategy in adults: A clinical practice guideline. Intensive Care Med 2020; 46(12):2226–2237.

**Compliance with ethics regulations:** Yes in clinical research.
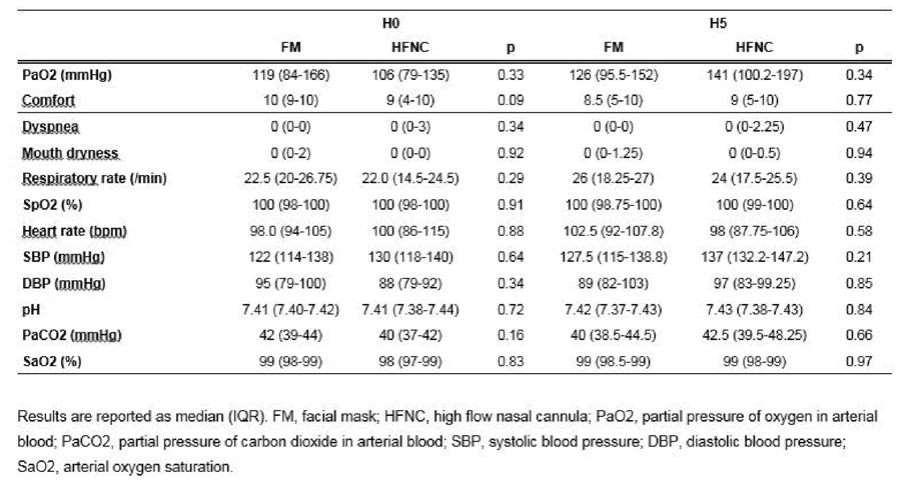


*Clinical and biological during oxygen therapy*

### FC-086 Long-term exposure to ambient air pollution is associated with an increased incidence and mortality of acute respiratory distress syndrome in a large French region

#### GUTMAN Laetitia^1,2^, PAULY Vanessa^2^, ORLEANS Veronica^2^, PIGA Damien^3^, CHANNAC Yann^3^, ARMENGAUD Alexandre^3^, BOYER Laurent^2^, PAPAZIAN Laurent^1,2^

##### ^1^Assistance publique des hôpitaux de Marseille - Hôpital nord, Marseille, France; ^2^Faculté de sciences médicales et paramédicales Aix Marseille, Marseille, France; ^3^ATMOSUD, Marseille, France

###### Correspondence: Laetitia GUTMAN (laetitiagutman@gmail.com)

*Annals of Intensive Care* 2022, **12(1):**FC-086

**Rationale:** Air pollution exposure is suspected to alter both the incidence and mortality in acute respiratory distress syndrome (ARDS). The impact of chronic air pollutant exposure on the incidence and mortality of ARDS from various etiologies in Europe remains unknown. The main objective of this study was to evaluate the incidence of ARDS in a large European region, 90-day mortality being the main secondary outcome.

**Patients and methods/Materials and methods:** The study was undertaken in the Provence-Alpes-Cote-d’Azur (PACA) region. Nitrogen dioxide (NO_2_), particulate matter (PM2.5 and PM10) and ozone (O_3_) were measured. The Programme de Médicalisation des Systèmes d’Information (PMSI), which captures all patient hospital stays in France, was used to identify adults coded as ARDS.

**Results:** From 2016 to 2018, 4,733 adults with ARDS treated in intensive care units were analysed. The incidence rate ratios for 1-year average exposure to PM2.5 and PM10 were 1.207 ([95% confidence interval (95% CI), 1.145–1.390]; P < 0.01) and 1.168 (95% CI, 1.083–1.259; P < 0.001), respectively. The same trend was observed for both 2- and 3-year exposures, while only chronic 1- and 2-year exposure NO_2_ exposures were related to a higher incidence of ARDS. Increased PM2.5 exposure was associated with a higher 90-day mortality for both 1- and 3-year exposures (OR 1.096 (95% CI, 1.001–1.201) and 1.078 (95% CI, 1.009–1.152), respectively). O_3_ was not associated with either of incidence or mortality.

**Conclusion:** Increased exposures to NO_2_, PM2.5 and PM10 were associated with an increased incidence of ARDS. Increased exposure to PM2.5 was also associated with increased 90-day ARDS mortality.

**Compliance with ethics regulations:** Yes in clinical research.

### FC-087 Clinical practice survey: assessment and ventilatory management of sickle cell patients in intensive care

#### HAGRY Julien^1^, MEKONTSO DESSAP Armand^2^, MONNET Xavier^1^, RAZAZI Keyvan^2^, CHANTALAT Christelle^1^, DE PROST Nicolas^2^, PHAM Tài^1^

##### ^1^CHU Bicêtre, Le Kremlin-Bicêtre, France; ^2^CHU Henri Mondor, Créteil, France

###### Correspondence: Julien HAGRY (hagryjulien@gmail.com)

*Annals of Intensive Care* 2022, **12(1):**FC-087

**Rationale:** Sickle cell disease, a common genetic disease in France, is a haemoglobinopathy characterised by a β-globin abnormality and the production of haemoglobin S. Hyperalgesic vaso-occlusive crisis (VOC) and acute chest syndrome (ACS) are the main complications leading to intensive care units (ICU) admissions. Despite frequent hospitalizations in ICUs, there is heterogeneity in treatment and global management due to a lack of ICU-specific guidelines. We aimed at gathering the medical practices of ICUs nationwide.

**Patients and methods/Materials and methods:** We carried out an online survey that was sent to medical teams of all ICUs of French hospitals identified as expert centers in the Orphanet database (https://www.orpha.net). Physicians from the same unit sent consensus answers to capture the usual care in their ICU, providing one survey answer per unit. The survey included sections related to: demography; usual pain management, and three clinical situations (hyperalgesic VOC, VOC with chest pain, and ACS with chest pain, dyspnea and fever).

**Results:** Of 55 centers contacted, 29 (53%) answered the survey, including 18 (62%) university-hospitals, 10 (34.5%) general hospital centers and 1 (3.5%) private hospital. The median [IQR] number of ICU beds was 23 [18–28]. We grouped centers according to the annual number of admissions: 16 (55%) centers admitted less than 10 patients with sickle cell disease per year. Pain management: nitrogen monoxide-oxygen mixture was used in 72% of centers. Nonsteroidal anti-inflammatory drugs were more likely used by centers admitting more than 10 patients per year, though not achieving statistically difference (61.5% vs 25%, p = 0.108). Oxygenation and physiotherapy: conventional oxygen therapy was decided regardless of the oxygen saturation in 52% for VOC and 55% in VOC associated with chest pain or ACS). Physiotherapy and incentive spirometry was systematically prescribed in 69% of the centers during a VOC, 82% in case of VOC with chest pain and 62% during ACS. Ventilatory support: according to physicians, non-invasive ventilation main benefit during ACS was the effect on alveolar recruitment and hypercapnia (90%), but patients’ tolerance seems to be its main limitation for 69%. High-flow nasal oxygen (HFNO) was considered easy to use (86%) and well-tolerated by patients (83%), but to 76% of the responders, its efficacy during ACS needs to be proven by large studies.

**Conclusion:** Pain and respiratory management of patients with sickle cell disease acute complications seems to be heterogeneous amongst expert centers. Incentive spirometry to treat or prevent ACS needs to be generalized. Further studies evaluating the benefits of HFNO are needed.

**Compliance with ethics regulations:** Yes in clinical research.
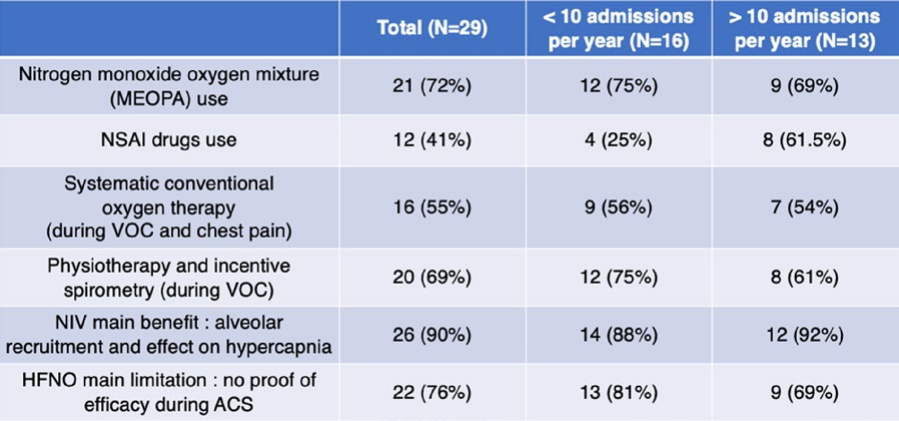


*Table 1: main management and respiratory devices effects according to the number of patients with sickle cell disease admitted per year.*

### FC-088 Acute exacerbations in chronic obstructive pulmonary diseases: eosinophil-guided corticosteroid therapy versus standard care

#### BEN ABA Feriel^1^, GHARBI Rim^2^, HAMMOUDA Zeineb^1^, LAHMAR Manel^1^, SIKALI Habiba^2^, FEKIH HASSEN Mohamed^2^, BESBES Lamia^1^, ELATROUS Souheil^2^, ABROUG Fekri^1^

##### ^1^Service de réanimation polyvalente CHU Fattouma Bourguiba, Monastir, Tunisie; ^2^Service de réanimation médicale CHU Tahar Sfar, Mahdia, Tunisie

###### Correspondence: Feriel BEN ABA (benaba.feriel@gmail.com)

*Annals of Intensive Care* 2022, **12(1):**FC-088

**Rationale:** Administration of systemic corticosteroid (SC) in acute exacerbations of chronic obstructive pulmonary diseases (AECOPD) patients requiring mechanical ventilation (VM) is controversial. There are biomarkers such as plasmatic eosinophilia (PE) that could predict a beneficial response to SC. The aim of our study was to assess whether a strategy of CS administration based on PE level was not inferior to the standard strategy consisting in systematic administration of CS regardless of the severity.

**Patients and methods/Materials and methods:** In a bi-centric, prospective, randomized, controlled, and single-blind trial conducted between December 2018 and December 2020, eligible patients over 18 years of age, hospitalized for an AECOPD and requiring ventilatory support (n = 43) were included and randomized to usual care (n = 25) or eosinophil-guided strategy (n = 18). The prescription of SC in the eosinophil-guided group was based on PE (1 mg/kg of prednisone if PE > 2%; no SC if PE ≤ 2%). The primary end- point was whether patients still needed MV in the sixth day or not.

**Results:** There was no between-group difference for demographics, COPD and acute episode characteristics. Regarding the primary end-point, there was no difference for the type of ventilation modality in the sixth day following the inclusion (Still on MV: 32% versus 39% in the standard group and the eosinophilic group, respectively). A difference of 7% that is less than the 10% which confirm the non-inferiority hypothesis. The relative risk to be still on MV by the 6th day was 1.2 (IC 95%: 0.54–2.7) p = 0.640, reaching the prior fixed non difference margin. Both study groups showed similar secondary outcomes: Treatment failure (p = 0.712), MV duration (p = 0.620), length of stay (p = 0.429) and mortality (p = 0.701). Hyperglycemic episodes due to SC occurred more frequently in the control group (6 cases versus 2) but no statistical significance was found (p = 0.434).

**Conclusion:** This preliminary study confirms the similarity between a CS sparing strategy based on PE count and the standard systematic administration of CS.

**Compliance with ethics regulations:** Yes in clinical research.

### FC-089 Effect of beta-blockers prescription on the outcome of severe acute exacerbation of chronic obstructive pulmonary disease (AECOPD) requiring ICU admission

#### HAMMOUDA Zeineb^1^, BOUATTIA Maroua^1^, BEDHIAFI Emir^1^, MAATOUK Iyed^1^, HOURI Fadoua^1^, LAHMAR Manel^1^, DACHRAOUI Fahmi^1^, ABROUG Fekri^1^, BESBES OUANES Lamia^1^

##### ^1^CHU Fattouma Bourguiba MONASTIR, Monastir, Tunisie

###### Correspondence: Zeineb HAMMOUDA (zanoubia83@hotmail.com)

*Annals of Intensive Care* 2022, **12(1):**FC-089

**Rationale:** Cardiovascular diseases are frequent comorbidities in patients with COPD and are conditions with large use of beta-blockers (BB). We aimed to evaluate the influence of BB prescription on AECOPD outcome.

**Patients and methods/Materials and methods:** This is a single-centre retrospective study conducted in a 16-bed ICU of Fattouma-Bourguiba University-hospital. From January 2016 to June 2019, all consecutive patients admitted for an AECOPD and aged ≥ 18 years were included. Exclusion criteria were: AECOPD requiring specific treatment and unavailable long-term follow-up. Patients were divided on 2 groups: (BB+) previously receiving Beta-blockers or requiring Beta-blockers agents upon their admission and (BB-) who did not receive beta-blockers neither previously nor during ICU stay. Data were collected on socio-demographic characteristics, past history and COPD GOLD stage, clinical presentation and labs findings. Severity was assessed using SAPS II and ABG’s parameters. We also recorded details of ICU course. Follow-up data included mortality at 6 months of ICU discharge and life quality assessment using the Clinical COPD Questionnaire (CCQ).

**Results:** 220 patients were included. Median age was 68 (IQR: 61–75), 88.2% were male with a BMI at 26.12 (23.6–30.1). Main comorbidities were hypertension 83 (37.7%), chronic heart disease 60 (27.3%) and diabetes 40 (18.2%). Median SAPS II was 45 (40–53). Main exacerbation causes were acute left heart failure in 99 (45%) followed by tracheobrochitis 75 (34.1%). NIV was the first-line ventilatory treatment in 88.2% with a failure rate at 26.8%. Median LOS was 16 (IQR 10–27) days with a survival rate at 80.5%. 62 (28.2%) were assigned to BB+ group versus 158 (71.8%) to BB-. Bisoprolol was the most used molecule (96.8%) with a median posology of 2.5(2.5–5) mg. 15 (24.2%) patients were previously receiving BB, indications for BB upon admission were hypertension 39 (62.5%) followed by left heart failure and tachycardia. Comparing the two groups, no major differences in severity assessment, ventilatory management or NIV failure were found. BB+ were older [73 (65–78) versus 67 (60–73.5), p = 0.001] with higher BMI [27.7 (24.7–31.2) versus 24.9 (22–30), p = 0.02] without difference in GOLD staging or long-term NIV requirement (p = 0.4). BB− had lower LOS 15(9.7–23) versus 20.5(12.7–30.2) p = 0.004 with similar rates of ICU complications and mortality. Thus, 6 months mortality was significantly higher in BB- (34.2 versus 17.7%, p = 0.016) [Fig. 1], and recurrence of AECOPD at one-year was lower in BB+ (43.5% versus 58.2%; p = 0.04). No significant difference in CCQ assessment was found (p = 0.2).

**Conclusion:** Despite similarities in ICU evolution, patients under beta-blockers appears to have better log-term outcome after AECOPD. Further studies might be needed to confirm this result.

**Compliance with ethics regulations:** Yes in clinical research.
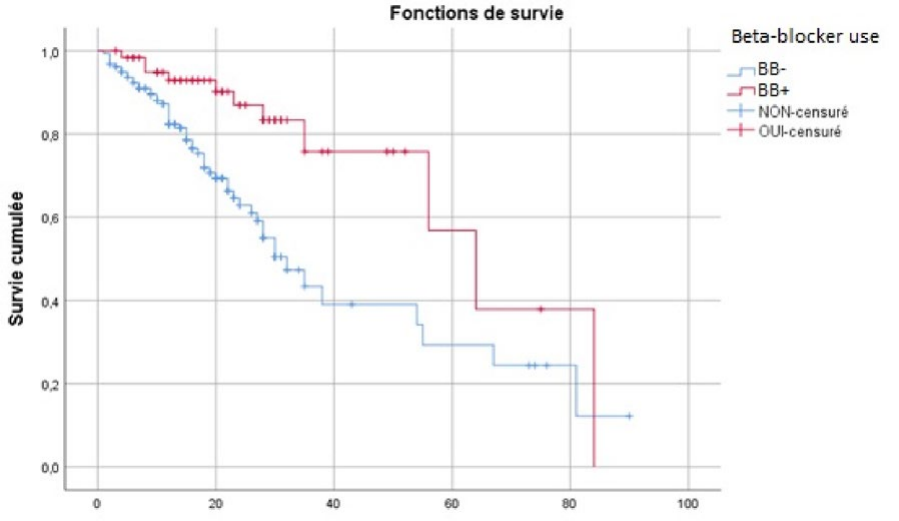


*Figure 1: Kaplan–Meier survival curves (6 month mortality) according to beta-blockers use status*

### FC-090 Impact of intubation on the evolution of fractions of inspired oxygen in patients treated with high flow oxygen therapy for COVID-19 associated pneumonia

#### LAFAY Valentin^1^, WINISZEWSKI Hadrien^1^, GUILLIEN Alicia^2^, CAPELLIER Gilles^1^

##### ^1^CHU de BESANCON, service de Réanimation Médicale, Besançon, France; ^2^Institut pour l’avancée des biosciences, Equipe d’épidémiologie environnementale appliquée au développement et à la santé respiratoire, Grenoble, France

###### Correspondence: Valentin LAFAY (valentin.lafay@sfr.fr)

*Annals of Intensive Care* 2022, **12(1):**FC-090

**Rationale:** Since second wave, High Flow Oxygen Therapy (HFOT) is a first line treatment for COVID-19 associated respiratory failure. However, patients treated with HFOT may be exposed for a long time to high Fractions of Inspired Oxygen (FiO_2_), potentially leading to hyperoxic alveolar injuries. We aimed to determine whether intubating patients after HFOT failure was associated with a decrease in high FiO_2_ exposure within the 24 first hours following intubation.

**Patients and methods/Materials and methods:** Among 218 patients admitted for COVID-19 associated pneumonia in Medical Intensive Care Unit (ICU) at Besançon teaching hospital between October 2020 and October 2021, 27 treated with HFOT for more than 24 h before intubation were retrospectively included. The number of hours exposed to FiO_2_ > 60% and > 90% during the 24 h preceding intubation and the 24 h following intubation were calculated and compared.

**Results:** Most of the 27 patients were men, with a median age of 71 years-old. Twenty-one patients performed awake prone positioning (PP) under HFOT. The median PaO_2_/FiO_2_ was 79 mm Hg before intubation, and 193 mm Hg 24 h after protective ventilation using a median Positive End-Expiratory Pressure (PEEP) of 12 cm H_2_O. The median dynamic compliance of the respiratory system was 40 mL/cm H_2_O. All ventilated patients benefited from neuromuscular blockade and PP. Seven (26%) died in ICU. The median number of hours with FiO_2_ > 60% decreased from 22,5 (Interquartile range—IQR—[20,0; 24,0]) within the 24 h under HFOT preceding intubation to 6,0 [4,0; 9,8] within the 24 h following intubation, corresponding to a median difference of − 15,0 [− 18,0; − 8,8], statistically significant (p < 0,001). The median number of hours with FiO_2_ > 90% decreased from 3,0 [1,3; 15,5] to 2,0 [1,3; 3,0], corresponding to a median difference of − 2,0 [− 10,0; 0], statistically significant (p = 0,005).

**Discussion:** Intubation itself likely doesn’t explain the decrease in FiO_2_. High PEEP, neuromuscular blockade, and prone positioning are confounding factors. FiO_2_ is also dependent from the target of oxygenation set as a goal. To the best of our knowledge, this study is nevertheless the first caring about this pragmatic issue. Its main limitations are a retrospective and monocentric design, and a small sample size.

**Conclusion:** In COVID-19 patients treated with high flow oxygen therapy > 24 h, intubation is associated with a statistically significant decrease in high FiO_2_ exposure. Whether intubation could decrease hyperoxic alveolar injury need to be investigated.

**Compliance with ethics regulations:** Yes in clinical research.
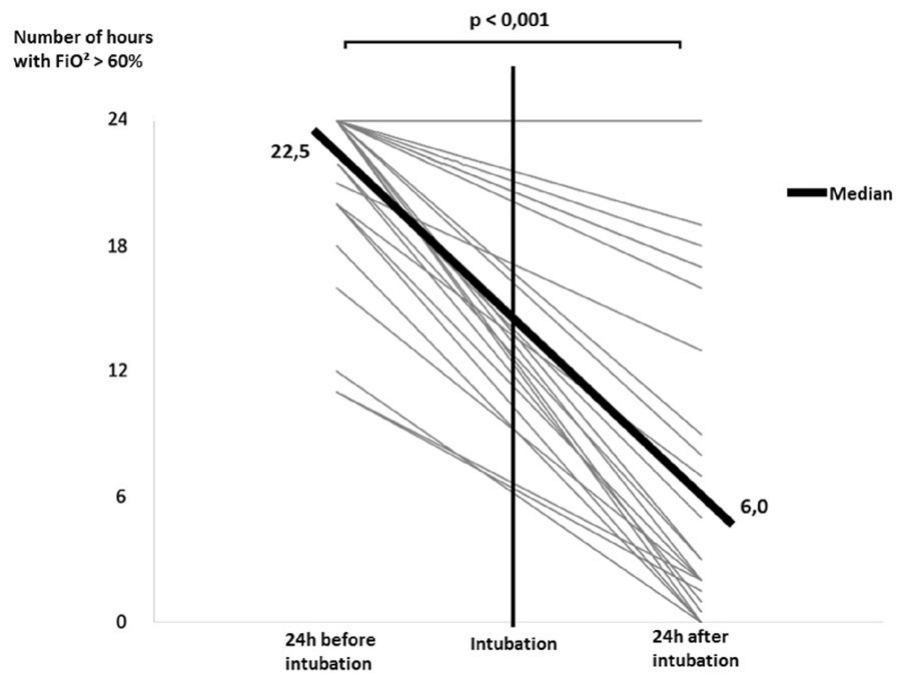


*Evolution of the number of hours exposed to FiO* ^*2*^ *> 60% during the 24 h preceding and the 24 h following intubation for each patient*

### FC-091 Importance of antithrombotic treatment monitoring in ventricular assisted patients

#### HANAFIA Omar^1^, PRIEUR-GARROUSTE Julie^1^, BERTAULT-PERES Pierre^1^, HONORE Stéphane^1^, MICHEL Fabrice^1^

##### ^1^Hopitaux Universitaires de Marseille, Marseille, France

###### Correspondence: Omar HANAFIA (omarhanafia@yahoo.fr)

*Annals of Intensive Care* 2022, **12(1):**FC-091

**Rationale:** We looked at a long-term external ventricular assist device (VAD), it’s a mechanical, pulsatile heart support system. It is indicated for patients with severe heart failure waiting for transplantation or recovery of their ventricular function. VAD imposes a stable and effective antithrombotic strategy. In pediatric resuscitation patients, inter- and intra-individual pharmacokinetic variability makes the use of these treatments with hemorrhagic and ischemic risks difficult. We conducted an evaluation of the professional practices of this strategy within the hospital’s pediatric resuscitation unit.

**Patients and methods/Materials and methods:** Our monocentric, retrospective study included a population of VAD-implanted patients from December 2017 to January 2020. Clinical data was collected from the computerized patient record. The prescriptions for anticoagulants and antithrombotics were followed on the shipboard prescription. The results of the aggregation tests and the anti-Xa assays were recorded on the laboratory software.

**Results:** Of the 36 months of the study: 5 middle-aged patients 12 months (sd = 7.9) were implanted with a left mono VAD. 4 patients had dilated cardiomyopathy and one had cardiogenic shock. We had 14 serious vascular incidents of which 4 were hemorrhagic and 10 ischemic. The frequency of tests does not exclude accidents and 67% of them had clinical repercussions. Anti-Xa measurements were 53% (n = 352) in the targets, the arachidonic acid test was effective in 58% (n = 44) of cases and ineffective in 15%, the adenosine diphosphate test was effective in 22% (n = 17) and ineffective in 58%. Dosage re-evaluations were proposed by the pharmacy when targets were not met. These results highlight the difficulty in obtaining and maintaining target rates. Our study did not establish a correlation between the duration under VAD and the number of accidents. By studying the 7 days before each accident, we found that even for patients previously anticoagulated and antiaggregated effectively, 71% of accidents are explained by a punctual but frank exit from the objective.

**Conclusion:** The number of accidents is not proportional to the frequency of tests, nor to the duration of implantation and it is essential to be constantly in the targets. A study described a decrease in accidents through the appointment of a referral physician to analyze the results on a daily basis and increased awareness of the care team. This allowed us to establish a sustainable strategy throughout the management of the patient, the pharmaceutical team already participates in and that could be improved.

**Compliance with ethics regulations:** Yes in clinical research.

### FC-092 Levosimendan use in pediatric resuscitation: clinical impact and proper use of the medication

#### HANAFIA Omar^1^, LESLUYES Bruno^1^, HONORE Stéphane^1^, BERTAULT-PERES Pierre^1^, MICHEL Fabrice^1^

##### ^1^Hopitaux Universitaires de Marseille, Marseille, France

###### Correspondence: Omar HANAFIA (omarhanafia@yahoo.fr)

*Annals of Intensive Care* 2022, **12(1):**FC-092

**Rationale:** Levosimendan is used as a last resort in severe or decompensated chronic heart failure. For pharmacoeconomic reasons its delivery in our center is subject to a prior agreement, which is why the problem of positioning of this molecule in the therapeutic arsenal of our establishment arises. We carried out a study within the pediatric cardiac resuscitation unit, the objective of which was to analyse the habits of the department and the prescription conditions as well as the profile and the outcome of patients who received a levosimendan cure.

**Patients and methods/Materials and methods:** This is a retrospective, monocentric study from January 2017 to June 2019. We analysed the service’s paper prescriptions thanks to the prescriptions and the computerized patient record, we recorded the cures, the prescription modalities, the length of hospitalization. We also sought to determine the clinical impact of the first cure via the serum level of nt-proBNP at D-1, D1 and D9 as well as the fate of patients at D30 and D60.

**Results:** 18 patients were included, the average age was 12.8 months, and the H/F ratio was 0.63. On the first cure: only 6 cures respected the good practices, 7 patients had dobutamine prescribed before the first cure. 11 patients received several cures (2.9 total cures on average). The average length of hospitalization was 72 days. The rate of nt-proBNP decreased by an average of 49% between D-1 and D1 in 10 patients, and increased by 203% in the remaining 8 patients. Between D1 and D9, this rate decreased by an average of 52% in 8 patients and increased by 178% in the remaining 9 patients. At D30 of the first treatment, 1 patient had returned home, 10 were hospitalized, 3 were hospitalized under circulatory assistance and 4 had died. At D60 of the first treatment, 4 patients were at home, 6 were hospitalized, 3 were hospitalized under circulatory assistance, 5 died. The efficacy of levosimendan is evaluated by the improvement of the ventricular ejection fraction, unfortunately this ultrasound parameter does not appear in the PDI for each patient and therefore could not be analyzed in our study.

**Conclusion:** Levosimendan brings a proven benefit in certain physio-pathological terrains thanks to its singular mechanism of action. However, according to the literature, it is no more effective than dobutamine. Given its cost, it would be fair to continue this multidisciplinary work to redefine the place of this treatment in the therapeutic arsenal of our institution.

**Compliance with ethics regulations:** Yes in clinical research.

### FC-093 Risk factors associated with prolonged ECMO in children: a referral center cohort study

#### BERGEZ Léa^1^, LEVY Yael^1^, LEGER Pierre Louis^1^, RAMBAUD Jerome^1^.

##### ^1^Armand-Trousseau, Paris, France

###### Correspondence: Jerome RAMBAUD (jerome.rambaud@aphp.fr)

*Annals of Intensive Care* 2022, **12(1):**FC-093

**Rationale:** This study aimed to investigate the characteristics and clinical outcomes of children supported with prolonged ECMO (≥ 28 days) for severe acute respiratory failure or cardiac failure.

**Patients and methods/Materials and methods:** We conducted a retrospective study in our referral center for ECMO between 2009 and 2020. All pediatric patients age from 28 days to 18 years-old supported with ECMO for ≥ 28 days were included. All neonatal indications for ECMO were excluded. We looked for pre-ECMO treatment such as mechanical ventilation settings, the use of prone positioning, nitric oxide, exogenous surfactant, neuromuscular blockers. We also gathered the ventilation settings at day 1, 3, 7, 14 and 21, outcome criteria as the median duration of ECMO, the length of invasive mechanical ventilation, the length of intensive care stays and the survival rate following intensive care discharge and 6 months after ICU discharge.

**Results:** On the 223 patients treated by ECMO during the study period, 14 (7%) patients underwent an ECMO run longer than 28 days. Median ECMO run duration was 44 days (28–122). Patients requiring a long ECMO run were younger (574 vs 1079 days), had a significantly lower PaO2/FiO2 ratio (51 vs 62, p < 0.001) and higher mean airways pressure (22 vs 18, p: 0.03). A lower tidal volume at day 7 of ECMO was significantly associated with long run (2.3 vs 4.6, p: 0.02). Higher FiO2 requirement on oxygenator and mechanical ventilator were significantly associated with long-run ECMO at day 7 and 14. Patients having a long run were suffering from more bleeding and infectious complications. Half of the deaths for long run patients were related to palliative care. Survival rate was lower for patients having a long run.

**Conclusion:** Long run ECMO represent a minority of all case of ECMO. Ventilator parameters at day 7 of ECMO may help to identified theses runs. Early identification of early patients at risk of a long run could be useful to prevent bleeding complication and to prepare potential bridge to transplantation.

**Compliance with ethics regulations:** Yes in clinical research.

### FC-094 Cefepime population pharmacokinetics and dosing regimens optimization in pediatric intensive care unit

#### DE CACQUERAY Noémie^1^, HIRT Deborah^2^, ZHENG Yi^2^, BILLE Emmanuelle^1^, LEGER Pierre Louis^3^, RAMBAUD Jerome^3^, TOUBIANA Julie^1^, CHOSIDOW Anais^3^, VIMONT Sophie^3^, CHOUCHANA Laurent^1^, BÉRANGER Agathe^1^, TRELUYER Jean-Marc^1,2^, BENABOUD Sihem^2^, OUALHA Mehdi^1^

##### ^1^Necker, Paris, France; ^2^Cochin, Paris, France; ^3^Trousseau, Paris, France

###### Correspondence: Noémie DE CACQUERAY (n_a_cacqueray@hotmail.fr)

*Annals of Intensive Care* 2022, **12(1):**FC-094

**Rationale:** Cefepime is commonly used in pediatric intensive care units (PICU) while patients are subject to unpredictable variability in pharmacokinetic (PK) parameters leading to drug concentrations modification. This study aimed to build a population PK model for cefepime in critically ill children, to optimize and individualize initial dosing regimens.

**Patients and methods/Materials and methods:** Children (age > 1 month and < 18 years, weight > 3 kg) receiving cefepime were included. Cefepime total plasma concentrations were measured using high performance liquid chromatography. Data were modelled using Monolix software and Monte Carlo simulations were performed using a PK target of 100% fT > MIC.

**Results:** Fifty-nine patients with median (range) age of 13.5 months (1.1 month-17.6 years) and 129 cefepime concentrations were included in the analysis. Cefepime data were best fitted by a one-compartment model and the selected covariates were body weight (BW) through allometric scaling and estimated glomerular filtration rate (eGFR) on clearance (CL). Mean population values for CL and volume (V) were 1.2 L.h^−1^ and 5.01 L, respectively. According to the simulations, 150 mg.kg^−1^.day^−1^ q6h over 3 h or in continuous infusions better achieved the PK target in case of normal or augmented renal clearance.

**Conclusion:** Extended and more frequent or continuous cefepime infusions are needed in critically ill children with normal or augmented renal clearance. Cefepime displays narrow therapeutic index and require therapeutic drug monitoring.

**Compliance with ethics regulations:** Yes in clinical research.

### FC-095 Meropenem and piperacillin population pharmacokinetics and dosing regimen optimization in critically ill children receiving continuous renal replacement therapy

#### THY Michael^1,3^, URIEN Saik^3,4^, FOISSAC Frantz^3,4^, BOUAZZA Naim^3,4^, BILLE Emmanuelle^5^, BÉRANGER Agathe^2,3^, RAPP Mélanie^2,3^, LUI Gabrielle^3,6^, LESAGE Fabrice^2^, RENOLLEAU Sylvain^2^, TRÉLUYER Jean-Marc^3,4,6^, OUALHA Mehdi^2,3^

##### ^1^Assistance Publique-Hôpitaux de Paris, Infectious and tropical diseases department, Bichat University Hospital, Université de Paris, Paris, France, Paris, France; ^2^Assistance Publique-Hôpitaux de Paris, Paediatric Intensive Care Unit, Necker-Enfants Malades University Hospital, Université de Paris, Paris, France, Paris, France; ^3^EA 7323 - Pharmacology and Therapeutic Evaluation in Children and Pregnant Women, Université de Paris, France, Paris, France; ^4^Unité de recherche Clinique-Centre d’Investigation Clinique, Hôpital Cochin-Necker, Université Paris Descartes, Sorbonne-Paris Cité, 149 rue de Sèvres, 75015 Paris, France, Paris, France; ^5^Laboratoire de microbiologie, Hôpital Necker Enfants- Malades, Université Paris Descartes, Sorbonne-Paris Cité, 149 rue de Sèvres, 75015 Paris, France, Paris, France; ^6^Service de pharmacologie clinique, Hôpital Cochin, Université Paris Descartes, Sorbonne-Paris Cité, 27 rue du Faubourg Saint-Jacques, 75014 Paris, France, Paris, France.

###### Correspondence: Michael THY (michael245thy@gmail.com)

*Annals of Intensive Care* 2022, **12(1):**FC-095

**Rationale:** High variability in critically ill children receiving Continuous Renal Replacement Therapy (CRRT) increases the risk of inadequate concentrations. We aimed to develop a meropenem (MRP) and piperacillin (PIP) population pharmacokinetic (PK) model in this population and simulate dosing regimens to optimize patient exposure.

**Patients and methods/Materials and methods:** MRP and PIP plasma concentration was quantified by high-performance liquid chromatography. PK was investigated using a non-linear mixed-effect modeling approach. Monte Carlo simulations were performed to determine the most appropriate therapeutic dosing regimens.

**Results:** Respectively, for MRP and PIP, 27 patients with an age of 4 [0–11] years old, weight of 16 [7–35] and 32 children with a median (IQR) postnatal age of 2 years (0–11), body weight (BW) of 15 kg (6–38), receiving continuous replacement renal therapy (CRRT) were included. For both MRP and PIP, concentration–time courses were best described by one-compartment model with first-order elimination with body weight (BW) and CRRT flow (Qd) for MRP and residual diuresis (Qu) for PIP as covariates explaining the lower between-subject variabilities on volume of distribution (V) and clearance (CL). For a 70-kg subject patient i, the final equations were: -For MRP: CLi = (CLpop × (BWi/70)0.75) × (Qd/1200)0.4), where CLpop and Vpop are 6 L/h and 35 L respectively. -For PIP: Vi = Vpop × (BW/70)1 and clearance (CL): CLi = (CLpop × (BWi/70)0.75) × (Qu/0.06)0.12), where CLpop and Vpop are 7 L/h and 53 L respectively. After Monte-Carlo simulations for MRP and PIP, we suggested dosing regimens for a target of 100% fT > 4xMIC in Fig. 1.

**Conclusion:** Optimal antibiotic exposure in critically ill children under CRRT needs an adaptation of the doses to CRRT flow rate for MRP and to residual diuresis for PIP.

**Compliance with ethics regulations:** Yes in clinical research.
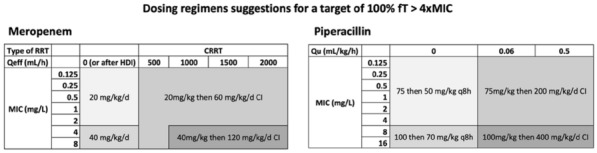


*Fig. 1 Dosing regimens suggestions for a target of 100% fT* > *4xMIC.*

### FC-096 Septic shock in severe trauma patients in pediatric intensive care

#### KALOUCH Samira^1^, FAKHR Kawtar^1^, AISSAOUI Wissal^1^, CHLILEK Abdelaziz^1^

##### ^1^CHU Ibn Rochd, Casablanca, Maroc

###### Correspondence: Samira KALOUCH (dr.kalouch@gmail.com)

*Annals of Intensive Care* 2022, **12(1):**FC-096

**Rationale:** Sepsis is the result of complex interactions between infectious microorganisms and the host's immune, inflammatory, and coagulant responses. The signs of sepsis constitute a continuum of increasing severity, ranging from the trivial association of tachycardia, tachypnea and fever with hyper leukocytosis or leukopenia to the occurrence of circulatory abnormalities, then organ dysfunction, and finally a state of shock that responds more or less easily to vascular filling and vasopressors.

**Patients and methods/Materials and methods:** This is a descriptive and analytical retrospective study spread over 5 years (January 1, 2015–December 31, 2020) in the pediatric intensive care unit including 47 cases. Statistical analysis is done using SPSS software.

**Results:** In this study, we report 47 cases of septic shock in severe trauma patients collected in the pediatric intensive care unit of the children's hospital of CHU Ibn Rochd in Casablanca during the years 2015–2020. The study was carried out according to an operating sheet including demographic, clinical, biological, radiological and therapeutic data, followed by statistical analysis using SPSS software 20. The average age is 75 months with a male predominance. The sites of infection were, in descending order, respiratory infection, bacteremia, urinary tract and skin. The germs mainly isolated were Acinetobacter baumanii, Pseudomonas aeruginosa, Staphylococcus aureus and Klebsiella spp. The evolution was fatal in 59.5% of cases. The prognostic factors retained were the delay of transfer to the intensive care unit > 4 h (p = 0.003), the presence of more than 2 failing organs (p = 0.004), the need for filling > 60 ml/kg (p = 0.02), the use of more than one active substance (p = 0.006), the use of adrenaline (p = 0.01) and the delay of antibiotic therapy > 1 h (p = 0.0006).

**Conclusion:** Identification of the deficiencies in the management of septic shock and the application of the recommendations of the survivor sepsis campaign would improve the prognosis.

**Compliance with ethics regulations:** Yes in clinical research.

### FC-097 First nosocomial infections in children supported by veno-arterial extracorporeal membrane oxygenation (VA-ECMO)

#### COUSIN Vladimir^1^, RODRIGUEZ-VIGOUROUS Robert^1^, KARAM Oliver^2^, RIMENSBERGER Peter^1^, POSFAY-BARBE Klara^1^

##### ^1^Hôpitaux Universitaires de Genève, Genève, Suisse; ^2^Children’s Hospital of Richmond at VCU, Richmond, Va, Etats-Unis

###### Correspondence: Vladimir COUSIN (vladimir.cousin@hcuge.ch)

*Annals of Intensive Care* 2022, **12(1):**FC-097

**Rationale:** Veno-arterial (VA) Extracorporeal Membrane Oxygenation (ECMO) is a standard rescue procedure for patient with refractory shock in Pediatric Intensive Care Unit (PICU). They are at high risk of nosocomial infection with potential severe consequences. Aim of study was to determine the frequency, timing and microbiological specificity of VA-ECMO-related infection.

**Patients and methods/Materials and methods:** Study period spanned from 01/2008 to 12/2014 with a retrospective charts review; inclusion criteriawere patients with a VA-ECMO support for > 6 h. It was approved by local Ethics Committee (CE 14–231), who waived the need for informed consent. We recorded the first PICU infection during VA-ECMO support. Infection was defined as a positive microbiological sample with clinical signs of infection or clinical signs of severe infection without positive sample. Time to infection, length of VA-ECMO support, PICU stay and risk factors for infection were evaluated.

**Results:** During study period 41 patients were included, with a mortality of 53%. Mean time with VA-ECMO was 5,6 d (SD 4,7), mean time in PICU was 17.3 d (SD 14,3) (Fig. 1, stratified by infection status). Overall, 34% patients developed an infection, with an incidence of 60/1000 VA-ECMO days. Mean time to first infection was 3.8 d (SD 1.7), with Pseudomonas spp. as the most commonly detected microorganism (42%). Infected sites were ventilator-associated pneumonia (9/14), sternotomy infection (2/14), bloodstream (2/14) and urinary tract infections (1/14). Only length of VA-ECMO support and length of PICU stay were associated with infection: longer VA-ECMO support (> 5 d) (OR 5.9 (CI 95% 1.4–24.6; p = 0.01) or longer PICU stay (> 14 d) (OR 12 (95% CI 2.2–65.5; p = 0.004).

**Discussion:** In this monocentric study, we described the outcome and infectious complications in 41 patients with pediatric VA-ECMO. Our results underline the high proportion of infections in this population, its occurrence early during the VA-ECMO support period. We highlighted the association of the time with VA-ECMO support and PICU length of stay with the occurrence of infection. However, such findings may be surrogate markers for sicker patients, who are intrinsically more at risk for nosocomial infections rather than a direct impact of duration of VA-ECMO support.

**Conclusion:** Pediatric patient with refractory shock supported by VA_ECMO are at high risk of nosocomial infection. Strategies aimed at preventing these infections may improve the outcome of these critically ill children.

**Compliance with ethics regulations:** Yes in clinical research.
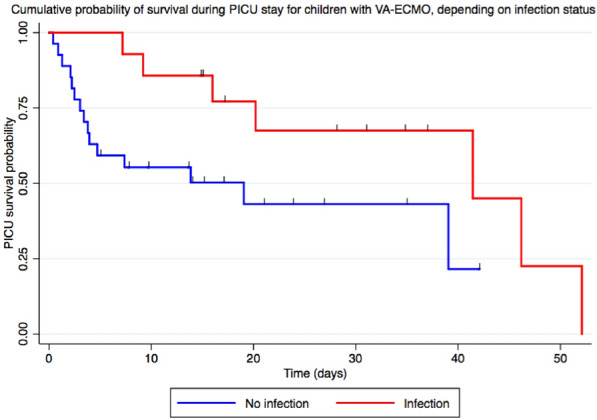


*Kaplan–Meier cumulative probability of survival during PICU stay for patient with veno-arterial exatracorporeal membrane oxygenation (VA-ECMO), depending on infection status. Comparison of curve using log-rank test found significant difference.*

### FC-098 Non-invasive evaluation of the response to vascular filling in the first three hours of sepsis and septic shock by comparison of two diagnostic strategies

#### YALAOUI Ilia^1,2^, BENMATI Abdellatif^1,2^, BOUHROUM Abdelhafid^1^, TEBOUL Jean Louis^3^

##### ^1^University 3 of Constantine, Faculty of Medicine, Constantine, Algerie; ^2^Preventive Medicine of Chronic Diseases Laboratory (Med Prev AC), Constantine, Algerie; ^3^The Kremlin-Bicêtre, Paris XI University, France, Paris, France

###### Correspondence: Ilia YALAOUI (yalabox@live.fr)

*Annals of Intensive Care* 2022, **12(1):**FC-098

**Rationale:** Evaluating preload to predict response to vascular filling (RVF) prompts the clinician to have criteria that predict the effectiveness of vascular filling (VF). Changes in the time velocity integral (TVI) of aortic or subaortic blood flow and cardiac output (CO) measured by echocardiography, as well as the passive leg raise (PLR) test, are excellent methods for predicting preload reserve. The objective of this work is to decrease hospital mortality (HM) and to improve survival, quality of RVF prediction and occurrence of incidents related to VF in patients with sepsis or septic shock and in spontaneous ventilation.

**Patients and methods/Materials and methods:** Through a prospective randomized prognostic trial, comparing two methods of evaluating the prediction of RVF, clinical and echocardiographic, of two groups of adult patients with sepsis or septic shock, in spontaneous ventilation and not previously infused. The clinical hemodynamic measurements collected before and after a 30 ml/kg of crystalloid filling test are: mean arterial pressure (MAP) and urine output (UO) in the control group (CG), as well as the TVI of the flow subaortic (TVIsa) and CO in the preload dependency group (PDG). In the PDG, TVIsa and CO measurements are also taken before and after PLR and VF.

**Results:** HM in responder patients is zero in sepsis and higher in septic shock and CG more than PDG (83,3% vs 72,7%). Survival is better in patients with PDG, in sepsis and in septic shock. RVF is significantly elevated in PDG compared to CG (92% vs 61,5%), and better in septic shock (77,3%) compared to sepsis (71,4%). There are more incidents in the GC compared to the GPD (19,2% vs 8%). The incidents are absent in sepsis and weak in septic shock (23,8% GC versus 8,7% GPD).

**Discussion:** Our study provided evidence in the acute phase of sepsis and septic shock in spontaneously ventilated patients, in predicting RVF and managing VF non-invasively and effectively by echocardiography, as well as in improving HM in septic shock, survival and the occurrence of VF-induced incidents.

**Conclusion:** The non-invasive prediction of RVF, by TVIsa measured by echocardiography, makes it possible to identify responders and non-responders in VF in real time, to test the hemodynamic efficiency of VF, to limit incidents related to overload during VF, to decrease mortality and to improve survival.

**Reference 1:** Rhodes A, et al. Surviving Sepsis Campaign: International Guidelines for Management of Sepsis and Septic Shock: 2016. Intensive Care Med. 2017; 43(3):304–77.

**Reference 2:** Jozwiak M, Monnet X, Teboul JL. Implementing sepsis bundles. Ann Transl Med. 2016; 4(17):332.40.

**Compliance with ethics regulations:** Yes in clinical research.
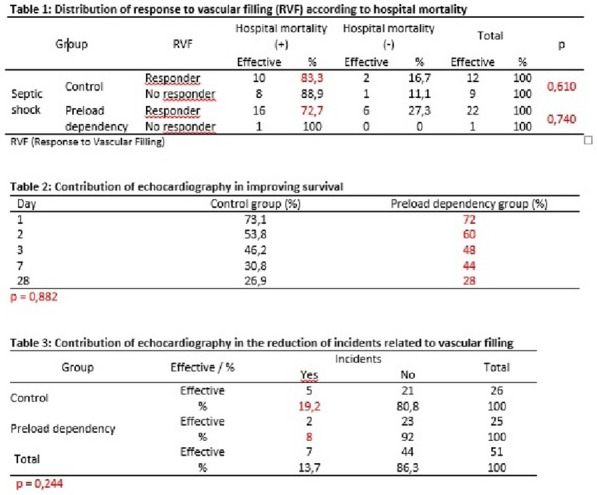


*Contribution of echocardiography in the evaluation of the response to vascular filling.*

### FC-099 Ultrasound-guided subclavian vein catheterization: infraclavicular versus supraclavicular approach

#### DAFFEF Saoussen^1^, TRABELSI Becem^1^, BEN TALEB Ibtissem^1^, KTATA Hiba^1^, BEN ALI Mechaal^1^

##### ^1^Mohamed Taher Maamouri Teaching Hospital, Anesthesiology and intensive care unit, Nabeul, Tunisie

###### Correspondence: Saoussen DAFFEF (sawssendaffef91@gmail.com)

*Annals of Intensive Care* 2022, **12(1):**FC-099

**Rationale:** The subclavian vein (SCV) is the preferred site for central venous catheterization (CVC) in intensive care unit due to its ability to stay patent in hypovolemic situations and lower risk of infections. Difficulty in its ultrasonic visualisation with the infraclavicular (IC) approach is the reason why this approach has fallen out of favour. The ultrasound (US) guided supraclavicular (SC) approach seems to be a good alternative. The aim of this study was to compare the IC and the SC approaches in US-guided SCV catheterization.

**Patients and methods/Materials and methods:** This was a prospective randomized study (NCT04637347). We included adult patients requiring a CVC in the superior vena cava area. Patients with infection at puncture site, coagulopathy, contralateral pneumothorax, trauma of clavicle and distorted anatomy of the neck were excluded from the study. Patients were randomly divided into two groups: US-guided SCV catheterization using either SC approach (SCGr) or IC approach (ICGr). We used long-axis scanning in combination with in-plane technique for all catheterizations. The primary outcome was the first attempt success rate. Overall success rate, venous scanning time, number of punctures, number of redirections, puncture time, guidewire insertion time, overall catheterization time, incidence of difficulties in catheterization and mechanical complications were secondary outcomes.

**Results:** We included 110 patients in this study: 55 in the SCGr and 55 in the ICGr. First attempt success rate was significantly higher in SCGr compared to ICGr (81.8% versus 63.6%; p < 10–3). The overall success rate was significantly higher in SCGr compared to ICGr (96.4% versus 85.5%; p < 10–3). Venous scanning time was significantly longer in the ICGr compared to the SCGr (21.7 ± 15.6 s versus 15.35 ± 8.9 s; p < 10–3). The number of punctures was significantly lesser in the SCGr compared to the ICGr (1.2 ± 0.5 versus 46 ± 0.7; p < 10–3). Regarding the number of redirections, the puncture time and the guidewire insertion time, no significant difference was observed between the two groups. The overall catheterization time was significantly shorter in SCGr compared to ICGr (63.13 ± 31.7 s versus 76.34 ± 33.6 s; p < 10–3). The rate of mechanical complications were higher in the ICGr without significant difference (14% versus 5%; p = 0.065). No cases of pneumothorax or malposition were observed in the SCGr however we observed three cases of pneumothorax and two cases of malposition in the ICGr. The incidence of arterial punctures and hematoma formation were comparable between the two groups.

**Conclusion:** US-guided in-plane catheterization of SCV using SC approach is safe and seems to be a good alternative to the classic IC approach.

**Compliance with ethics regulations:** Yes in clinical research.

### FC-100 Transcranial doppler: easy or difficult technique?

#### SADAT Souhila^1^, ZEGHDOUD Dalila ^1^, BOUGDAL Dalila^1^.

##### ^1^EHS Salim Zemirli, Alger, Algeria

###### Correspondence: Souhila SADAT (sadatsouhila@hotmail.fr)

*Annals of Intensive Care* 2022, **12(1):**FC-100

**Rationale:** Transcranial Doppler (DTC) is a specific monitoring of cerebral hemodynamics in the brain-damaged, allowing the evaluation of cerebral blood flow. Purpose of the study: Evaluate the DTC learning curve, because the acquisition of a medical technical gesture is part of a quality approach in the training of junior doctors.

**Patients and methods/Materials and methods:** The study included ten residents, it took place in three phases, -The first phase: The participating doctors benefited from a theoretical presentation on DTP followed by a practical demonstration lasting three hours. -A second phase: Over a period of three weeks consisted for the junior doctors, a phase in which between 25 to 35 DTCs were carried out. The evaluation by the referent was carried out at each DTC in order to detect any anomalies. -The third phase: Follows the previous phase, it consists of performing ten DTCs by each candidate in order to complete their learning curve. The data for each exam was collected by the candidate. The candidate also provides the results of his DTC as well as that carried out by the referent. This examination concerns the search for the right and left middle cerebral artery with the values of the systolic (Vs), average (Vm) and diastolic (Vd) velocities, the value of the pulsatility index (IP) and the duration of each examination.

**Results:** The failure rate of completing the DTC was higher in the first and second attempts by our ten learners, which ranged from 60 to 80%, the success and failure rates were similar in the third and fourth trials. From the fifth attempts the success rates are higher than those of failures which vary from 20 to 30%. The comparison of the mean values of Vs, Vm, Vd and IP of the candidates compared to those of the referent made it possible to conclude that there is no significant difference between the results obtained. The comparison of the time taken to perform the DTC by the candidates compared to the referring doctor revealed the presence of a significant difference in the results obtained (P < 0.001).

**Conclusion:** The DTC is an easy technique, quick to learn, but an evaluation of the sustainability of the acquisition of this technique by the learners is essential.

**Compliance with ethics regulations:** N/A.

### FC-101 Teaching intensive care nurses to recognize the misplacement of endotracheal and nasogastric tubes on chest radiographs

#### KAMEL Toufik^1^, SAUVAGE Brice^1^, LAKHAL Karim^2^, OTTAVY Gregoire^3^, JANSSEN-LANGENSTEIN Ralf^4^, JACQUIER Marine^5^, LARRAT Charlotte^6^, JACQ Gwenaëlle ^7^, E. DAUVERGNE Jérôme ^2^, MAUGARS Diane^3^, LABRUYERE Marie^5^, SIMEON Véronique ^6^, CUGNART Cécile ^8^, GIRAULT Christophe^9^, BOULAIN Thierry^1^

##### ^1^Service de Médecine Intensive Réanimation, Centre hospitalier régional d'Orléans,, Orleans, France; ^2^Service d'Anesthésie-Réanimation, hôpital Laënnec, Centre Hospitalier Universitaire,, Nantes, France; ^3^Service de médicine intensive-réanimation, CHU de Nantes, France, Nantes, France; ^4^Service de Médecine Intensive et Réanimation – Hautepierre, CHU de Strasbourg,, Strasbourg, France; ^5^Service de Médecine Intensive et Réanimation, CHU de Dijon,, Dijon, France; ^6^Service de Médecine Intensive et Réanimation CHRU de Tours,, Tours, France; ^7^Service de Réanimation médico-chirurgicale, centre hospitalier de Versailles, Versailles, France; ^8^Rouen University Hospital, Medical Intensive Care Unit,, Rouen, France; ^9^Normandie Univ, UNIROUEN, EA 3830, Rouen University Hospital, Medical Intensive Care Unit, Rouen, France

###### Correspondence: Toufik KAMEL (toufik.kamel@chr-orleans.fr)

*Annals of Intensive Care* 2022, **12(1):**FC-101

**Rationale:** To assess the effectiveness of a single standardized training on the ability of intensive care registered nurses (RN) to recognize the misplacement of endotracheal and nasogastric tubes on bedside chest radiographs of intensive care unit (ICU) patients.

**Patients and methods/Materials and methods:** In 8 French ICUs, RNs received a 110-min standardized teaching on the position of endotracheal and nasogastric tubes on chest radiographs. Their knowledge was evaluated within the subsequent weeks. On 20 chest radiographs, each with an endotracheal and a nasogastric tube, they had to indicate whether each tube was in proper or incorrect position. A lower bound of the 95% confidence interval (95%CI) of the mean correct response rate (CRR) was chosen to indicate the training success when > 90%. Residents of the participating ICUs were subjected to the same evaluation (without prior specific training).

**Results:** One hundred eighty-one RNs were trained and evaluated, and 110 residents evaluated. The mean CRR in RNs was 84.6% (95%CI: 83.3–85.9), significantly higher than in residents (81.4% [95%CI: 79.7–83.2]) (P = 0.003 by t test). Linear mixed modelling handling the centers as random effect variable confirmed the significant difference (P < 0.0001) in CRR between RNs and residents as shown in Fig. 1. For misplaced nasogastric tubes, the mean CCR in RNs was 95.9% (95% CI: 93.9–98.0). Age, sex, seniority, delay between training and evaluation (for RNs) and period of evaluation within the semester of internship (for residents) did not significantly impact (all P > 0.05) on the mean CRR, either in the whole set of participants or in RNs and residents taken separately.

**Conclusion:** The ability of trained RNs to detect tube misplacement was higher than that observed among residents and was considered satisfactory for detecting misplaced nasogastric tubes. This is encouraging but insufficient to ensure patients safety. Transferring responsibility for reading radiographs to detect the misplacement of endotracheal tubes to intensive care RNs will need a more advanced or more in-depth teaching method than that used in this study.

**Compliance with ethics regulations:** N/A.
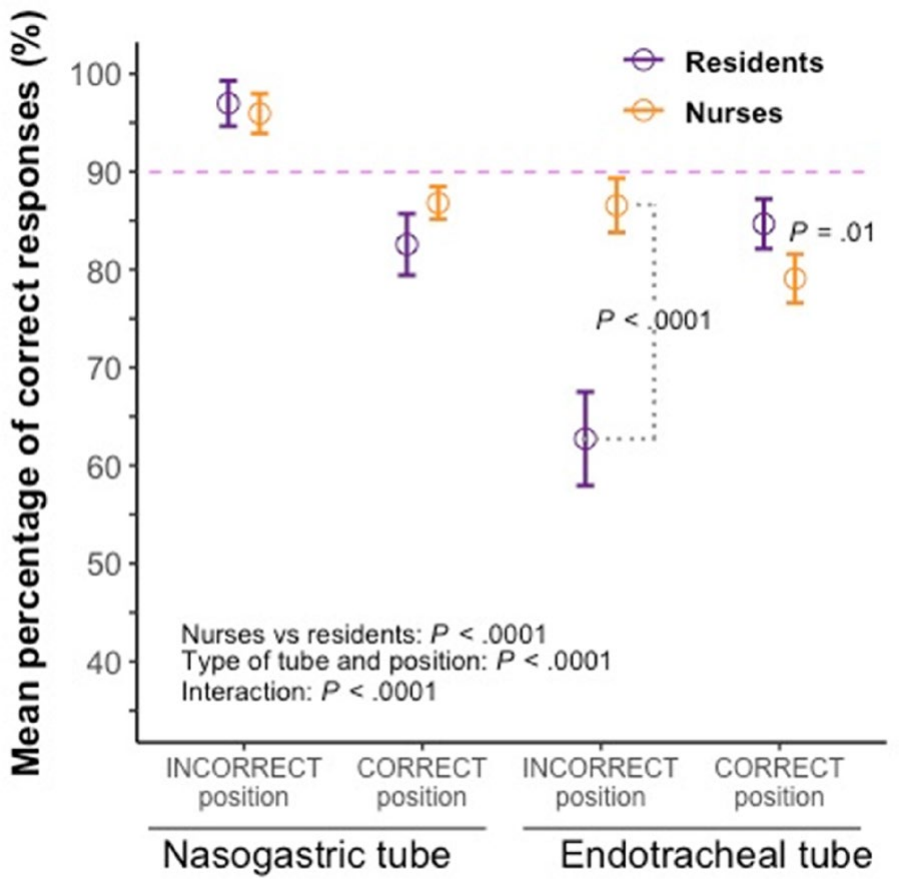


*Titre: Mean percentage of correct responses by type of tube and by position (correct or incorrect)*

### FC-102 Involvement of healthcare teams in the evaluation and selection of smart-pumps in intensive care

#### BRUAIRE Léo-Paul^1^, BECK Nathalie^1^, PIERRE DE LA BRIÈRE Jean-Charles^1^, LEVEAUFRE Guillaume^1^, ROUSSEL Damien^1^, LECOUTOUR François^1^, JORET Aurélie^1^, GOYER Isabelle^1^, BROSSIER David^1^

##### ^1^CHU de Caen, Caen, France

###### Correspondence: David BROSSIER (david_brossier@yahoo.fr)

*Annals of Intensive Care* 2022, **12(1):**FC-102

**Rationale:** Medication errors are a major public health issue. As part of the project of dematerialization of the patient record in our intensive care units, the computerization of the medical prescription and the securing of the administration of the drug were among our objectives. In order to secure the drug circuit and reduce the drug errors, many scientific societies recommend the use of injectable drug delivery devices connected to the computerized medical record, called “smart-pumps”. The main objective of the study was to compare 2 ranges of smart-pumps available in France. The secondary objective was the involvement of the paramedical teams in the choice of their future work tools.

**Patients and methods/Materials and methods:** We carried out a prospective monocentric, comparative, interventional study, without human involvement of the ranges proposed by B.Braun and a second company between September the 23rd and October, the 15th of 2019, in adult, pediatric and neonatal intensive care units of the Caen university hospital. This assessment was coupled with a satisfaction survey. All staff nurses received a training in the use of the equipment. Subsequently, the nurses trained on the 2 ranges proceeded to the successive evaluation of these throughout two scenarios of application of medical prescriptions. The order in which each nurse evaluated the 2 ranges was randomized. The main criterion of judgement was the overall satisfaction of nursing staff with the use of the devices. The secondary criteria concerned the handling of materials and consumables as well as navigation in drug libraries and the duration of each scenario, timed by a project member.

**Results:** 77 agents (25% of the nurses concerned) were trained, 54 of whom evaluated the two ranges. The fulfilment of the medical prescription and the installation of the tubing in the pump was considered longer with the range proposed by B.Braun. No other significant difference was observed. However, 56% of nurses advised the range proposed by B.Braun which appeared safer, more ergonomic and less bulky.

**Conclusion:** To our knowledge, no such study has ever been published. Without observing major differences in use between the two companies, this comparison process allowed us to determine the equipment that seemed to best meet the expectations of the paramedical teams. Moreover, by involving and following the teams in the choice of their work equipment we hope to have strengthened the adherence of the teams to the change of practice induced by the use of smart pumps.

**Compliance with ethics regulations:** Yes in clinical research.

### FC-103 Influence of clinical pharmacist presence on acceptance and delivery of pharmaceutical interventions in a pediatric resuscitation unit

#### HANAFIA Omar^1^, HACHE Guillaume^1^, BERTAULT-PERES Pierre^1^, HONORE Stéphane^1^, MICHEL Fabrice^1^

##### ^1^Hopitaux Universitaires de Marseille, Marseille, France

###### Correspondence: Omar HANAFIA (omarhanafia@yahoo.fr)

*Annals of Intensive Care* 2022, **12(1):**FC-103

**Rationale:** Studies have shown that medication errors are common in intensive care units. The drug management of pediatric and neonatal populations is particularly at risk of iatrogenia due to the pathophysiological characteristics of these populations, the lack of suitable pharmaceutical forms and based on weak agreements. In addition, the clinical situations of these patients are serious and often require complex therapeutic strategies. Securing drug prescriptions is critical in these situations. The objective of this study was to measure the influence of the presence of the clinical pharmacist in the clinical department on the safety of drug prescriptions.

**Patients and methods/Materials and methods:** We conducted an observational study, before/after a pharmacist was involved in a 30-bed critical care unit. The analysis and daily pharmaceutical validation of prescriptions were performed from the pharmacy on the first part of the study (before). The issuance of Pharmaceutical Interventions (PI) was conducted by telephone and plotted on the printed prescription. For the second part (after), the pharmaceutical validation of the prescriptions was carried out in the service and the PI transmitted orally to the prescribers in person and plotted in the same way. The main criterion for evaluating the securing of prescriptions was the number of PI issued. We also characterized the nature of the PI, the consensus achieved orally, and the achievement of the prescription change suggested by the PI.

**Results:** We have 351 PI out of 126 days before, these PI mainly concerned overdoses (52%), route and/or inappropriate administration (17%) and non-conformities to the repository (10.8%). 179 children were affected by these IP. We found 1120 IP over 287 days, these IP mainly concerned monitoring (27.8%), overdoses (21.8%) and under dosing (10.1%). 300 children were affected by these IP.

We observed an increase in the number of IP performed per day (2.8 vs 3.9), the average number of IP per patient (2 vs 3.7). An increase in the number of IP accepted (267 vs 756; p = 0.036) and the number of implementations (183 vs 629; p < 0.001) was highlighted.

**Conclusion:** The presence of the pharmacist in the clinical department increased the number and relevance of PI performed. The nature of PI has evolved with a more patient-centred orientation. Acceptance and achievement rates are better, the percentage of PI achieved is higher, and these results show that PI are both better monitored and better monitored. Risk reduction is best with pharmaceutical expertise through the presence of a clinical pharmacist in the care unit.

**Compliance with ethics regulations:** Yes in clinical research.

### FC-104 A prospective pharmacist review of drug-related problems after ICU discharge: preliminary data

#### ANDRÉ Sébastien^1^, ROUSSEAU Anne-Françoise^2,3^, COLSON Camille^2,3^, MISSET Benoît^2,3^, GILLET Manon^1^

##### ^1^Hospital Pharmacy Department, University Hospital of Liège, Liège, Belgique; ^2^Intensive Care Department, University Hospital of Liège, Liège, Belgique; ^3^University of Liège, Liège, Belgique

###### Correspondence: Sébastien ANDRÉ (sebastien.andre@chuliege.be)

*Annals of Intensive Care* 2022, **12(1):**FC-104

**Rationale:** A stay in an intensive care unit (ICU) and the transitions of care are known to be at risk of drug-related problems (DRPs). These problems may contribute to readmissions and development of post-intensive care syndrome. The objective of this monocenter prospective study was to describe the prevalence of specific DRPs in patient’s post-ICU drug treatments.

**Patients and methods/Materials and methods:** Adults with an ICU stay ≥ 7 days between 16th November 2021 and 25th January 2022 were included if they were enrolled in our post-ICU follow-up program. A pharmacist conducted a full medication review including medication reconciliation. This review was planned in general ward, during the week following ICU discharge. The pharmacist identified potential DRPs that were classified using the Pharmaceutical Care Network Europe Classification for Drug-Related Problems. Tailored interventions were also delivered to general wards clinicians based on identified DRPs.

**Results:** We included 29 patients (72.4% men, age 59 [33–76] years, ICU stay 19 [7–95] days). Drug treatments were reviewed 5 [2–9] days after ICU discharge. A total of 148 DRPs were identified: 27/29 patients (93.1%) experienced at least 1 DRP and a median of 5 [0–12] DRPs were observed per patient. Most DRPs referred to (potential) adverse drug events (86/148, 58.1%), (potential) non-optimal effect of drug treatment (26/148, 17.6%) and unnecessary drug-treatment (26/148, 17.6%). The main cause of DRPs was related to drug selection (80/160, 50.0%) comprising absence of indication, inappropriate drug according to guidelines and inappropriate combination of drugs. Other causes of DRPs included prolonged duration of treatment (36/160, 22.5%), inappropriate dose or dosage regimen (23/160, 14.4%) and medication reconciliation problem (7/160, 4.4%). Drugs involved in DRPs belonged mainly to the nervous system group, the alimentary tract and metabolism group, and the cardiovascular system group. The most common drugs implied were tramadol and pantoprazole. Based on identified DRPs, 147 pharmacist interventions were discussed with the clinicians. Withdrawal of a drug was the predominant intervention (75/147, 51.0%) followed by provision of information about the DRP, dosage regimen modification, dose change, initiation or resumption of a drug and drug switch. 74.2% of all interventions were accepted by the clinicians.

**Conclusion:** DRPs were common after ICU discharge. Drugs of the nervous system group and proton pump inhibitors probably require sustained attention. Future research should evaluate the impact of pharmaceutical interventions on mid-term outcomes of ICU survivors.

**Compliance with ethics regulations:** Yes in clinical research.

### FC-105 Evaluation of a negative pressure aerosol protection box to prevent airborne transmission of SARS-COV-2 to healthcare providers in pediatric intensive care

#### CECÍLIA Rotava Buratti^1,2^, JOUVET Philippe^1^, BRIDIER Armelle^1^, VEILLETTE Marc^3^, DUCHAINE Caroline^3^

##### ^1^Sainte Justine Research Center, University of Montreal, Montreal, Canada; ^2^Postgraduate program in child and adolescent health, Universidade Federal do Rio Grande do Sul, Porto Alegre, Bresil; ^3^Research Center of the institut universitaire de cardiologie et de pneumologie de Québec, Quebec City, Canada.

###### Correspondence: Rotava Buratti CECÍLIA (ceciliaburatti@gmail.com)

*Annals of Intensive Care* 2022, **12(1):**FC-105

**Rationale:** High contagiousness of SARS-COV-2 is caused by bioaerosols’ emission^1^. The risk of healthcare providers (HCPs) developing infectious diseases through contact with patients is well recognized, and clinical situations involving tissue manipulation with high viral load called “aerosol-generating procedures” (AGP) may increase this risk^2^. The aim of this study was to investigate the impact of the aerosol protection box, the Splash-Guard Caregivers (SGGC), on the presence of viral particles after an AGP.

**Patients and methods/Materials and methods:** Prospective observational study conducted between April and June 2020, including the HCPs in charge of children admitted to a Pediatric Intensive Care Unit who tested positive for COVID-19. SGCG (https://rsr-qc.ca/Splashguard-cg/) was not used systematically and room patients analyzed were divided in: SGCG+ and SGCP−. Virus detection was performed in the single room patient’s environment using two methods (air pumps and swabs) on several sites: 1) on the air one meter from the patient’s head (wearable pumps), 2) inside the SGGC from the patient’s air (if used), also 3) on the air near each HCPs (wearable pumps), and 4) at each HCPs forehead (swab) after an AGP. Samples were analyzed for SARS-COV-2 RNA by qPCR.

**Results:** Eight batches of samples were performed in the single room of SARS-COV-2 + child (SGCG+ n = 3 and SGCG− n = 5). Five samples (11.4%) were qPCR positive for SARS-COV-2 among the 44 analyses. Three of these (14.3%) among the 21 analyses from the group SGCG−: in the air before the AGP (n = 1), in the air near HCP’s head after the AGP (n = 1), and in the HCP forehead swab after the AGP (n = 1). In the group SGCG+, 2 positive samples (8.7%) were observed among the 23 analyses in the HCP forehead swab (n = 1) and in the air near HCP’s head (n = 1), both after an AGP **(see Supplementary Table)**. None of the HCPs studied were infected by SARS-COV2.

**Conclusion:** Our results document the presence of SARS-COV2 in infected children environment. The protection effect of SGCG needs additional research.

**Reference 1:** van Doremalen N et al.: Aerosol and Surface Stability of SARS-CoV-2 as Compared with SARS-CoV-1. N Engl J Med 2020.

**Reference 2:** Nguyen LH et al.: Risk of COVID-19 among front-line health-care workers and the general community: a prospective cohort study. Lancet Public Heal 2020.

**Compliance with ethics regulations:** Yes in clinical research.
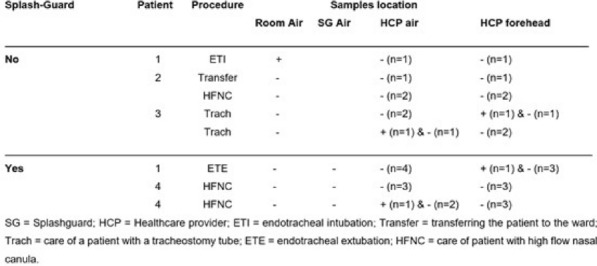


*Supplementary Table: Sample characterization*

### FC-106 Acute kidney injury in patients with necrotizing soft tissue infection in intensive care unit: a comparative retrospective study

#### ROZENBLAT David^1^, URBINA Tomas^4^, HUA Camille^5^, RAZAZI Keyvan^1,2,3^, MEKONTSO-DESSAP Armand^1,2,3^, DE PROST Nicolas^1,2,3^, ARRESTIER Romain^1,2,3^

##### ^1^Service de Médecine Intensive Réanimation, Hôpitaux Universitaires Henri Mondor, Assistance Publique-Hôpitaux de Paris, CEDEX, Créteil, 94010 Paris, France, Créteil, France; ^2^Groupe de Recherche Clinique CARMAS, Faculté de Santé de Créteil, Université Paris Est Créteil, CEDEX, Créteil, 94010 Paris, France, Créteil, France; ^3^INSERM, IMRB, Université Paris Est Créteil, CEDEX, Créteil, 94010 Paris, France, Créteil, France; ^4^Service de Médecine Intensive-Réanimation, Hôpital Saint-Antoine, Assistance Publique-Hôpitaux de Paris, France, Paris, France; ^5^Service de Dermatologie, Hôpitaux Universitaires Henri Mondor, Assistance Publique-Hôpitaux de Paris, CEDEX, Créteil, 94010 Paris, France, Créteil, France.

###### Correspondence: Romain ARRESTIER (romain.arrestier@aphp.fr)

*Annals of Intensive Care* 2022, **12(1):**FC-106

**Rationale:** Necrotizing soft tissue infections (NSTI) are life-threatening bacterial infections and often requires admission to intensive care unit (ICU). Severity and specificities of this pathology, such as micro-organisms involved, rhabdomyolysis and nephrotoxic drugs used, could lead to a higher incidence of acute kidney injury (AKI) than in other sepsis conditions. Our objectives were to describe the clinical and biological characteristics of NSTI related AKI and to compare its frequency to a cohort of non-NSTI septic shock.

**Patients and methods/Materials and methods:** We conducted a single-center retrospective comparative study of patients with NSTI admitted in ICU between 2006 and 2021. Patients < 18 years old, patients with end-stage renal disease requiring dialysis and patients with nosocomial NSTI acquired after 7 days of hospitalization were excluded. Clinical, biological characteristics and outcomes of NSTI patients with AKI were compared to NSTI patients without AKI. A matching analysis of NSTI patients to a historical cohort of patients with septic shock non related to NSTI and admitted in ICU between 2010 and 2013 was performed.

**Results:** Overall, 77 patients with NSTI were included. Clinical and biological characteristics are described in Table 1. 65 patients (84,4%) developed AKI and 29 (44,6%) displayed stage 3 KDIGO with fourteen patients (21,5%) needed renal replacement therapy (RRT). Time from ICU admission to AKI onset was 1 (0–2) days. Shock at ICU admission was independently associated with AKI (OR = 6.2 [1.3–44.0], p = 0.045). Initiation of antibiotic therapy before ICU admission (OR = 0.05 [0.0–0.6], p = 0.045) and *Enterobacteriaceae* infections (OR = 0.17 [0.0–0.7], p = 0.02) were associated with a lower risk of AKI. Rhabdomyolysis was not associated with AKI. Total ICU length of stay and mortality were slightly higher for patient with AKI but not statistically different. 45 patients with septic shock non related to NSTI were matched to 45 NSTI patients. The rate of AKI (95,6% vs 84,4%, p = 0,16) and the need for RRT (31,1% vs 26,7%, p = 0,64) were not statistically different between the two groups.

**Conclusion:** AKI is common in patients with NSTI but is not significantly associated with outcome. Risk factors for AKI in patients with NSTI are shock at ICU admission and late initiation of antibiotherapy, however NSTI is not a risk factor of AKI compared to other etiology of septic shock.

**Compliance with ethics regulations:** Yes in clinical research.
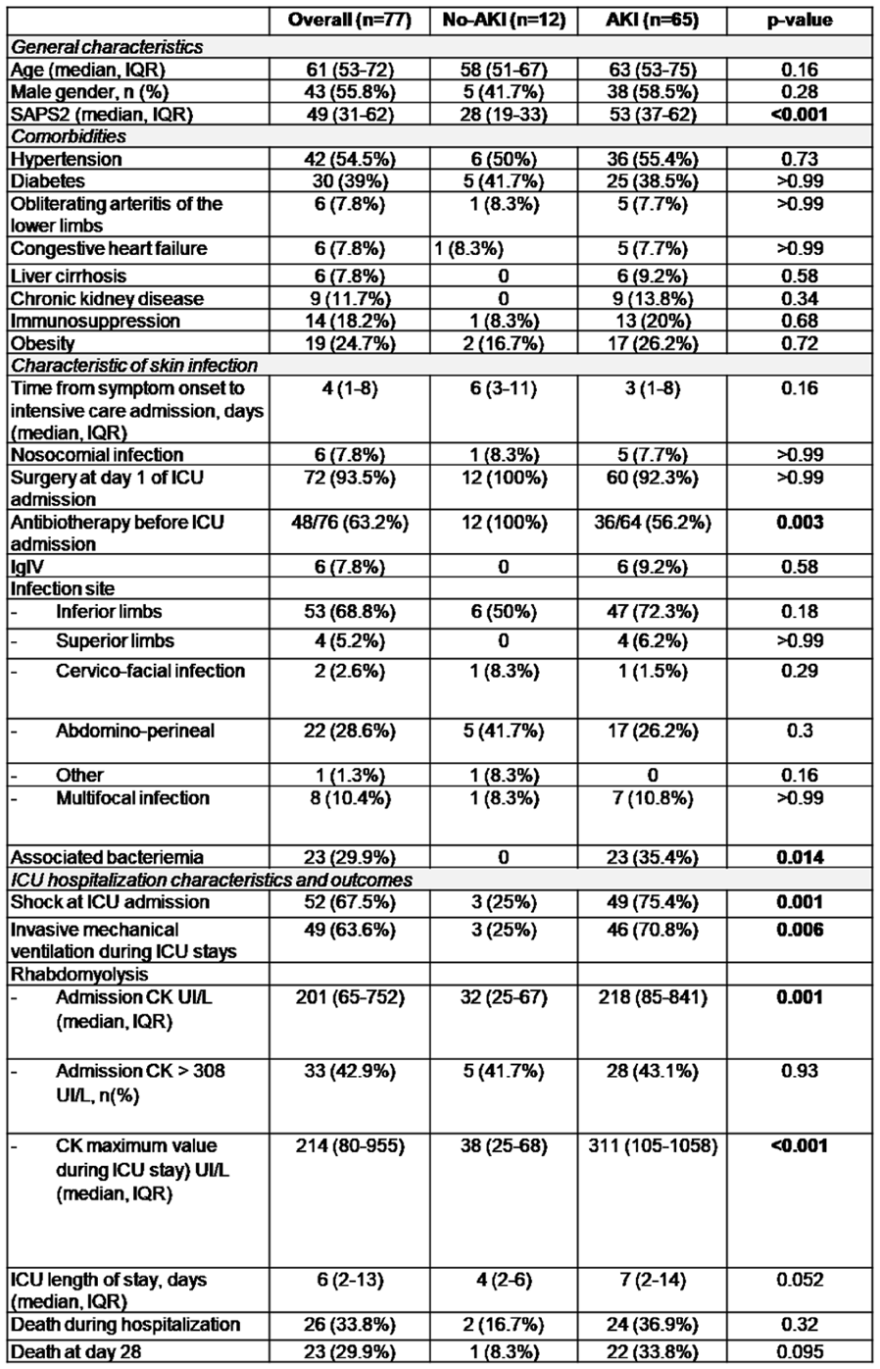


*Characteristics and outcome of NSTI patients with and without AKI*

### FC-107 Cardiorenal syndrome in piglets: development and characterization of a preclinical model

#### ORIEUX Arthur^1,2^, PIERONI Laurence^2,4^, DROUIN Sarah^2,4^, SAMSON Chloé^2^, DANG VAN Simon^3^, MIGEON Tiffany^2^, HADCHOUEL Juliette^2^, GUIHAIRE Julien^3^, MERCIER Olaf^3^, GALICHON Pierre^2,4^

##### ^1^Service de Médecine Intensive Réanimation - CHU de Bordeaux, Bordeaux, France; ^2^INSERM UMR_S1155, CoRaKiD - Sorbonne Université, Hôpital Tenon, Paris, France; ^3^INSERM UMR_S999, Hôpital Marie Lannelongue, GHPSJ, Université Paris Saclay, Le Plessis-Robinson, France; ^4^AP-HP, Paris, France.

###### Correspondence: Arthur ORIEUX (arthur.orieux@chu-bordeaux.fr)

*Annals of Intensive Care* 2022, **12(1):**FC-107

**Rationale:** Cardio-renal syndromes (CRS) type 1 and 2 are complex disorders in which cardiac dysfunction leads to kidney dysfunction. However, the mechanisms remain incompletely explained, in particular in right heart failure (RHF). Our objective was to develop an original preclinical model of CRS secondary to RHF associated with post embolic pulmonary hypertension (PH) in piglets.

**Patients and methods/Materials and methods:** Twelve 2-month-old large white piglets were randomized: group with induction of PH by ligation of the left pulmonary artery followed by weekly iterative embolization (soft tissue adhesive) of the right lower pulmonary artery, or SHAM control group (sham interventions) (Fig. 1). The sacrifice is made after 5 embolizations. We performed hemodynamic evaluation (right cardiac catheterization), echocardiography, laboratory blood, and urine tests. Histological evaluation and immunostaining of renal damage and repair were fulfilled. Measured glomerular filtration rate (GFR) procedures by injection of an exogenous tracer were repeated weekly.

**Results:** At sacrifice, mean pulmonary artery pressure (mPAP), pulmonary vascular resistance (PVR) and central venous pressure (CVP) were significantly higher in the PH group: 32 ± 10 vs 13 ± 2 mmHg (p = 0.001); 9.3 ± 4.7 vs 2.5 ± 0.4 mmHg (p = 0.004) and 10 ± 4 vs 6 ± 2 mmHg (p = 0.04) while the cardiac index (CI) was not different. Piglets with PH had right ventricular dysfunction and higher troponin I (302 [53–3054] vs 33 [17–47] ng/mL; p = 0.03). PH piglets showed increased histological marker of kidney injury (acute tubular necrosis-ATN-) and repair (proliferation assessed by Ki67). Albuminuria was increased (4.4 ± 0.6 vs. 2.9 ± 0.4 mg/L; p < 0.001) while serum creatinine (SCr) and uremia were not significantly different between the two groups. Unlike the estimated GFR, the measured GFR decreases from the 3rd week of the protocol. We reported a correlation between PH parameters and kidney function: mPAP (R2 = 0.4, p = 0.03), PVR (R2 = 0.50, p = 0.01) and CVP (R2 = 0.37; p = 0.04), but not with CI.

**Conclusion:** We reported the first porcine model of SCR on RHF secondary to PH. Renal dysfunction was characterized by ATN, increased albuminuria, and low measured GFR. The GFR estimated from SCr did not informed on renal dysfunction in these young piglets, unlike the measured GFR.

**Compliance with ethics regulations:** Yes in animal testing.
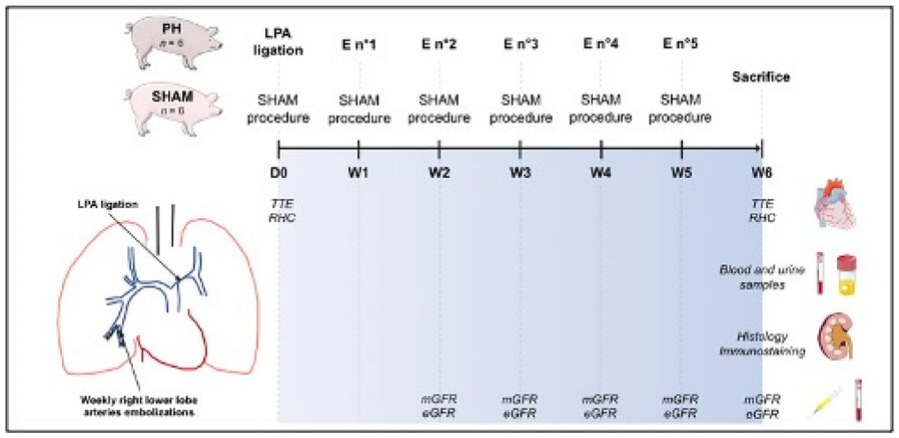


*Figure 1. Study protocol of pulmonary hypertension induction. D: day; E: embolization; mGFR: measured glomerular filtration rate; LPA: left pulmonary artery; PH: pulmonary hypertension; RHC: right heart catheterization; TTE: transthoracic echocardiography.*

### FC-108 Impact of positive fluid balance on outcomes in COVID-19 critically ill patients with ARDS

#### BOUGUEZZI Nabil^1^, TOUMI Radhouane^1,2^, MAATOUK Iyed^1^, BEDHIAFI Amir^1^, ZOUARI Hajer^1^, MAATOUK Khalil^1^, MEDDEB Khaoula^1,2^, BEN SAIDA Imene^1,2^, BOUSSARSAR Mohamed^1,2^

##### ^1^Medical Intensive Care Unit, Farhat Hached University Hospital, Sousse, Tunisie; ^2^Research Laboratory N° LR12SP09. Heart Failure. Farhat Hached University Hospital, University of Sousse, Sousse, Tunisie.

###### Correspondence: Nabil BOUGUEZZI (dr_nabil@live.fr)

*Annals of Intensive Care* 2022, **12(1):**FC-108

**Rationale:** COVID-19 pandemic represents a major public concern worldwide. Acute respiratory distress syndrome (ARDS) and lung failure are the main lung diseases found in COVID-19 patients. In ARDS patients, positive fluid balance can be associated with prolonged mechanical ventilation, more intensive care unit (ICU) complications, and higher mortality. Objective: The aim of this study was to evaluate the effect of fluid balance on morbidity and mortality in COVID-19 patients admitted to the ICU.

**Patients and methods/Materials and methods:** We conducted a retrospective observational study from March 2020 to October 2021 in a Medical ICU. Data concerning daily fluid balance, cumulative fluid balance at day 7, PaO_2_/FiO_2_ (P/F) ratio and outcomes of Critically Ill COVID-19 patients were obtained from medical records. The acute kidney injury (AKI) severity was classified according to the Kidney Disease Improving Global Outcomes guidelines. The study sample was divided into 2 groups according to fluid balance. Different outcomes (length of stay, invasive mechanical ventilation, acute kidney injury and death) were compared between the 2 groups.

**Results:** During the study period, 442 critically ill COVID-19 patients were admitted to our ICU. Positive fluid balance was found in 46% of patients (n = 207), and negative fluid balance was found in 53% of cases (n = 235), with a mean of 2271 ± 2285 ml and -2144 ± 2369 ml respectively. The mean cumulative fluid intake at day 7 was − 76 ± 3206 ml. Patients with positive fluid balance had a higher incidence of AKI (58.9%, n = 122) versus (37%, n = 87) (p < 0.001), of capillary leak (39%, n = 80) versus (27.8%, n = 63) (p = 0.013) and a higher mortality (54.4%, n = 112) versus (43.8%, n = 102) (p = 0.027). Mean length of stay was the same for the 2 groups (10.16 ± 6.15 days versus 9.99 ± 6.68 days, p = 0.79). The majority (50.6%, n = 118) required invasive mechanical ventilation (p = 0.11). P/F improved from 132 ± 77 on day 1 to 197 ± 90 on day 7 in negative fluid balance group (p < 10–3). Mean P/F was stable from day 1 and day 7 in positive fluid balance group (123 ± 55 and 129 ± 51 respectively, p = 0.36). Mean fluid balance at day 7 was significantly less for survivors than non survivors (− 505 ± 3364 ml and 413 ± 2961 ml respectively, p = 0.002).

**Conclusion:** Our study showed that patients with excessive fluid balance had more ICU complications and higher hospital mortality. Therefore, restrictive fluid strategies should be used to improve oxygenation and reduce mortality among COVID-19 patients.

**Compliance with ethics regulations:** Yes in clinical research.

### FC-109 Acute Kidney Injury (AKI) in the intensive care unit (About 151 cases)

#### KHALEK Khalid^1^, OULAHIANE Malika^1^, BOUHOURI Aziz^1^, AL HARRAR Rachid^1^

##### ^1^Service de réanimation chirurgicale des urgences (P.33), CHU Ibn Rochd, Casablanca, Casablanca, Maroc

###### Correspondence: Oulahiane MALIKA (oulahiane2238@gmail.com)

*Annals of Intensive Care* 2022, **12(1):**FC-109

**Rationale:** Acute kidney injury (AKI) is a frequent complication in intensive care units (ICU). The mortality remains high due to the aging of the affected population, the multiplicity of causes generating it, the association with other visceral failures and the frequent evolution in a septic context. The aim of this study is to study the different epidemiological and therapeutic aspects of AKI and try to determine the predictive factors of mortality in our population.

**Patients and methods/Materials and methods:** a descriptive and analytical cross-sectional study, which focused on 151 records of patients admitted to the ICU, who presented an AKI on admission or developed it during hospitalization. This study is spread over a period of 24 months, from 01/2019 to 12/2020.

**Results:** The incidence of AKI in our series was 17.32%. The average age of the patients was 49 ± 18 years with a male predominance (sex ratio M/F 2.14). The most common risk factors were: injection of contrast (77.9%), mechanical ventilation (67.8%), use of vasopressors (64.2%), nephrotoxic drugs (58%), diabetes (23.3%) and arterial hypertension (19.2%). The time of appearance of AKI was 2 ± 3 days. KDIGO stage 3 was predominant in our patients with a percentage of 53.6% followed by stage 1 (23.8%), and lastly stage 2 with a percentage of 22.5%. Dialysis was used in 18% of the patients. The mortality rate in our series was 62.33% and the factors found to have a significant relationship with death were: age, mechanical ventilation, use of vasopressors, multivisceral failure and septic shock.

**Conclusion:** A better knowledge of the risk and prognostic factors of AKI could be a major asset for a more effective management.

**Reference 1:** John R. Prowle, Christopher J. Kirwan and Rinaldo Bellomo. Fluid management for the prevention and attenuation of acute kidney injury. Nat. Rev. Nephrol. 10, 37–47 (2014):11. https://doi.org/10.1038/nrneph.2013.232.

**Reference 2:** Gaudry S, Ricard J-D, Leclaire C, Rafat C, Messika J, Bedet A, et al. Acute kidney injury in critical care: Experience of a conservative strategy. J Crit Care. déc 2014;29(6):1022–7.

**Compliance with ethics regulations:** Yes in clinical research.

### FC-110 Association of nitrogen balance trajectories with clinical outcomes in critical ill COVID-19 patients: a retrospective single center cohort study

#### DUPUIS Claire^1^, BRET Alexandre^1^, JANER Alexandra^1^, GUIDO Olivia^1^, BOUZGARROU Radhia^1^, DOPEUX Loïc^1^, HERNANDEZ Gilles^1^, MASCLE Olivier^1^, CALVET Laure^1^, THOUY François^1^, GRAPIN Kévin^1^, ADDA Mireille^1^, BOIRIE Yves^1^, SOUWEINE Bertrand^1^

##### ^1^CHU Gabriel Montpied, CHU Clermont Ferrand, Clermont Ferrand, France

###### Correspondence: Claire DUPUIS (cdupuis1@chu-clermontferrand.fr)

*Annals of Intensive Care* 2022, **12(1):**FC-110.

**Rationale:** The intensity and duration of the catabolic phase in COVID-19 patients might differ between survivors and no survivors. The purpose of the study was to assess the association between nitrogen balance (NBAL) trajectories and outcome in critical ill COVID-19 patients.

**Patients and methods/Materials and methods:** This retrospective study was conducted in intensive care at Clermont Ferrand University Hospital, France. From January 2020 to May 2021. The present study was approved by the Ethics Committee of Clermont Ferrand. Patients over 18 years old with a severe COVID-19 disease were eligible. Patients were excluded if they were referred from another ICU, if their ICU length of stay was < 72 h, and if they were treated by renal replacement therapy during the first seven days after ICU admission. The primary objective was to compare the profile of NBAL during the first two weeks after ICU admission between the patients with ICU death and the other patients. All data were prospectively collected and comprised details on ICU admission and during ICU stay. Vital status was collected at day 60. NBAL was estimated as proposed by Dickerson et all (Ref 1). Statistical analysis Comparisons between the evolution of NBAL depending on outcome was achieved using 2 level ANOVA. At day 3, 5 and 7, to represent the relationship between NBAL and protein intake, linear and non nonlinear models were achieved and the protein intakes necessary to reach a zero NBAL were determined. Sub-group analyses were achieved according to BMI, age, gender.

**Results:** 99 patients were included in the study. Their characteristics are reported in Table 1. On day 3, a negative similar NBAL was observed in survivors and non survivors: -16.4 g/d [− 26.5; − 3.3] and − 17.3 g/d [− 22.2; − 3.8] (p = 0.54). Then, the trajectories of NBAL over time differ between survivors and non survivors (p = 0.01). In survivors, NBAL increased over time (Patients with a net protein catabolism: 73.9% at day 3 versus 45.5% at day 14) whereas in non survivors, NBAL decreased from day 2 to day 6, and thereafter increased slowly up to day 14 (Patients with a net protein catabolism: 73.3% at day 3 vs 78.9% at day 14). Administrating higher protein amounts were associated with higher NBALs.

**Conclusion:** We reported a prolonged catabolic state in COVID patients which seemed more pronounced in non survivors than in survivors. Our study underlined the need for monitoring urinary nitrogen excretion to guide protein intakes in COVID-19 patients.

**Reference 1:** Dickerson, Nutrition, 2005.

**Compliance with ethics regulations:** Yes in clinical research.
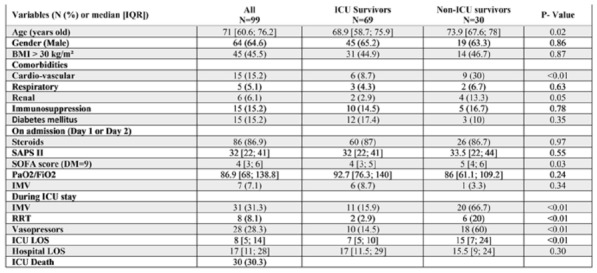


*Characteristic and comparisons of ICU COVID-19 patients between survivors and no survivors*

### FC-111 Management of renal replacement therapy in adults French intensive care units: a survey practices at the bedside (READIAL)

#### JOLLY Florian^1^, JACQUIER Marine^1,5^, PECQUEUR Delphine^2,3^, LABRUYÈRE Marie^1,2^, VINSONNEAU Christophe^4^, FOURNEL Isabelle^2^, QUENOT Jean-Pierre^1,2,5^

##### ^1^CHU Dijon, Dijon, France; ^2^INSERM, CIC 1432, Module Epidémiologie Clinique, Dijon, France; ^3^CHU Dijon-Bourgogne, Centre d’Investigation Clinique, Module Epidémiologie Clinique/Essais Cliniques, Chu Dijon-Bourgogne, Centre D’Investigation Clinique, Module Epidémiologie Clinique/essais Cliniques, France; ^4^Centre Hospitalier de Bethune, Service de Médecine Intensive Réanimation-Unité de Sevrage Ventilatoire et Réhabilitation, Bethune, France; ^5^Equipe Lipness, centre de recherche INSERM UMR1231 et LabEx LipSTIC, université de Bourgogne-Franche Comté, Dijon, France.

###### Correspondence: Jean-Pierre QUENOT (jean-pierre.quenot@chu-dijon.fr)

*Annals of Intensive Care* 2022, **12(1):**FC-111

**Rationale:** In France, recommendations for renal replacement therapy (RRT) in the intensive care unit (ICU) were published in 2015 and their uptake was recently assessed in a nationwide self-report online survey^1^. We performed an evaluation of real-life practices in the field in terms of RRT in a representative sample of ICUs across France.

**Patients and methods/Materials and methods:** The READIAL study was performed from 1 July to 5 October 2021. It was an evaluation of professional practices, and each centre was required to prospectively include 5 consecutive patients. Patients who required initiation of a first RRT using a hemodialysis catheter were eligible for inclusion. Patients with chronic renal failure requiring RRT through a tunneled catheter or an arterio-venous fistula were excluded.

**Results:** A total of 67 centres (83% of those contacted) agreed to participate, and included a total of 295 analysable patients (8 patients were erroneously included and therefore excluded from analysis). We found divergences between previously declared practices1 and actual practice observed in this study. The main indications for initiation of RRT are detailed in Fig. 1 (left panel), and the main sites of insertion of the first hemodialysis catheter are shown in Fig. 1 (right panel). The vast majority of centres used citrate, unfractionated heparin or NaCl as a catheter lock solution (47%, 24% and 21% respectively).

**Conclusion:** In this evaluation of professional practices that collected data at the bedside, the modalities of initiation of RRT in the ICU in patients with acute kidney failure were found to be globally in line with guidelines and literature data. The results should be interpreted in light of the limitations inherent to this type of study.

**Reference 1:** Quenot JP, Amrouche I, Lefrant JY, et al. Blood Purification 2021;4:1–10.

**Compliance with ethics regulations:** Yes in clinical research.
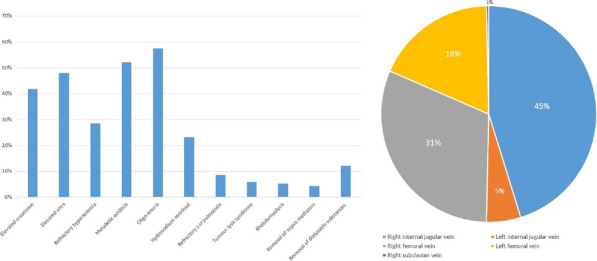


*Figure.*

### FC-112 Scleroderma cardiac crisis: a-life-threatening but reversible complication of systemic sclerosis

#### VIGNERON Clara^1^, PÈNE Frédéric^1^, CHARPENTIER Julien^1^, MOUTHON Luc^1^, CHAIGNE Benjamin^1^

##### ^1^Hôpital Cochin, Paris, France

###### Correspondence: Clara VIGNERON (claravigneron@hotmail.fr)

*Annals of Intensive Care* 2022, **12(1):**FC-112

**Rationale:** Systemic sclerosis (SSc) heart involvement account for 30% of SSc patient’s deaths.

**Patients and methods/Materials and methods:** We retrospectively studied patients with previous diagnosis of SSc admitted to the ICU for acute cardiac dysfunction between 2012 and mid-2021.

**Results:** Nine female patients were included, mainly with diffuse SSc (n = 7, 78%). Six (67%) had digital ulcers. All but one patient complained about physical cardiac symptoms (n = 8, 89%), 5 (56%) had electrocardiogram modifications. Biological exams revealed elevated troponin (705 µg/l [421–1582]) and Nt-pro-BNP (16,062 ng/l [10419–40738]). Patients exhibited severe left ventricular ejection fraction (LVEF) impairment (20% [10–20] vs 58% [53–60] before ICU admission (p = 0.0002)) requiring vasopressors and/or inotropes for 7 patients (78%) and mechanical ventilation or renal replacement therapy for 4 patients (44%). LVEF spontaneously improved during ICU stay (LVEF 40% [30–40] vs 20% [10–20], p = 0.0007) and returned to baseline within 6 months following ICU discharge (LVEF 53% [40–63] vs 58% [53–60]) (Fig. 1). Seven (78%) patients survived the ICU-stay and 4 (44%) were alive at 6 months.

**Conclusion:** We report an uncommon and specific severe acute life-threatening cardiac dysfunction in SSc patients, which can be reversible but remains associated with a poor long-term prognosis.

**Compliance with ethics regulations:** N/A.
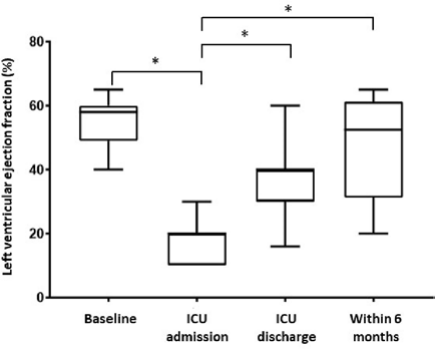


Figure 1. Evolution of left ventricular ejection fraction (%): at baseline, at ICU admission and discharge and within 6 months post-ICU discharge in ICU-survivors. *p < *0.05.*

### FC-113 A simplified algorithm to predict left ventricular systolic function in septic shock patients

#### BRAULT Clément^1^, ZERBIB Yoann^1^, MAIZEL Julien^1^, SLAMA Michel^1^

##### ^1^CHU Amiens-Picardie, Amiens, France

###### Correspondence: Clément BRAULT (brault.clement@chu-amiens.fr)

*Annals of Intensive Care* 2022, **12(1):**FC-113

**Rationale:** Septic cardiomyopathy (SCM) is characterized by a transient biventricular dysfunction with reduced contractility. Left ventricular (LV) ejection fraction (LVEF) is the conventional method to determine LV systolic function in septic shock, but is unhelpful to predict mortality. In contrast, the longitudinal strain (LVLS) might aid in early diagnosis of SCM, and was strongly associated with mortality. In this study, we developed an echocardiographic algorithm, using simple bedside parameters, to predict LVEF and LVLS in patients with septic shock.

**Patients and methods/Materials and methods:** We included all consecutive patients admitted with septic shock (defined in accordance with the Sepsis-2 criteria) or developing a septic shock during the ICU stay. We measured septal and lateral mitral annular plane systolic excursion (MAPSE), septal and lateral mitral S-wave velocity, and the left ventricular longitudinal wall fractional shortening (LV-LWFS). We used a conditional inference tree method to build a stratification algorithm; recursive partitioning was used to stratify the population of interest into subgroups. The best predictor and the corresponding optimal cutoff value were calculated. Bonferroni correction for multiple testing was applied to univariate p values. The tree-building process continued as long as a predictor led to significantly different child nodes.

**Results:** We included 71 patients (males: 59%; mean ± SD age: 61 ± 15 years). We found that 31% and 73% had an impaired LV systolic function assessed by LVEF and LVLS, respectively. The septal MAPSE, with a threshold of 1.2 cm, was the best parameter to predict the LV systolic function (Fig. 1). Other parameter (such as the S-wave or LV-LWFS) did not allow improving the algorithm’s performance. Septal MAPSE ≥ 1.2 cm predicted normal LVEF with near certainty. Conversely, septal MAPSE < 1.2 cm almost always predicted impaired LVLS, while almost half patients remained with preserved LVEF.

**Discussion:** Septal MAPSE, used as the main parameter to predict LV systolic function, has several advantages. First, it only requires a simple M-mode measurement, available in all ultrasound devices including handheld machines. Second, MAPSE is easily recorded even in patients with poor acoustic window (including obese patients) because of the high echogenicity of the atrioventricular plane. Third, its reproducibility is excellent even in inexperienced users, and require only minimal training.

**Conclusion:** Septal MAPSE, easily measurable at bedside and with good reproducibility, might be useful to support clinicians in early detection of SCM, especially when strain is not available or feasible.

**Compliance with ethics regulations:** Yes in clinical research.
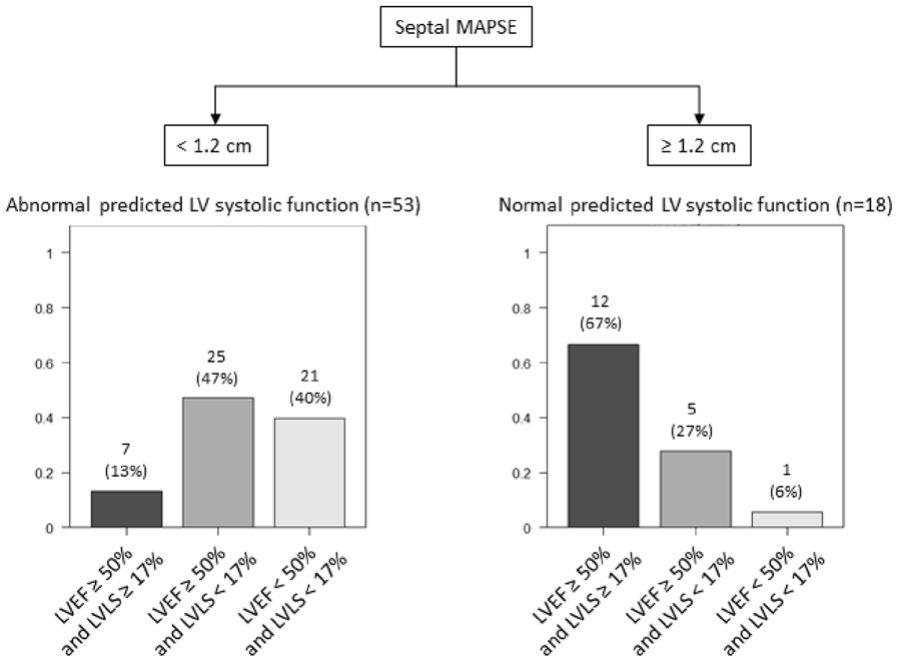


*Proposed algorithm for predicting LV systolic function*

### FC-114 An impaired global longitudinal strain at speckle tracking is associated with poorer haemodynamic condition irrespectively of the left ventricular ejection fraction during septic cardiomyopathy

#### GOBÉ Thibaut^1^, CARELLI Simone^1^, ROGER Guillaume^1^, LAI Christopher^1^, PHAM Tài^1^, TEBOUL Jean-Louis^1^, MONNET Xavier^1^

##### ^1^Hôpital de Bicêtre, Le Kremlin Bicetre, France

###### Correspondence: Thibaut GOBÉ (thibautgobe@gmail.com)

*Annals of Intensive Care* 2022, **12(1):**FC-114

**Rationale:** Global Longitudinal Strain (GLS) is more sensitive than left ventricular ejection fraction (LVEF) to detect impaired contractility during septic cardiomyopathy. However, its evolution over time has been poorly described. Moreover, the clinical importance of an impaired GLS is unclear. In particular, the haemodynamic consequences of an impaired GLS per se have never been reported.

**Patients and methods/Materials and methods:** In 61 adult patients with septic shock, we performed transthoracic echocardiography to measure LVEF and GLS with speckle tracking within the first 24 h (day-0), at day-1, day-3 and on the day when vasopressors were stopped. Simultaneously, central venous oxygen saturation (ScvO_2_), blood lactate and cardiac index (PiCCO2 device) were assessed.

**Results:** Eleven patients were excluded for poor echogenicity or prone positioning at the time of inclusion. The prevalence of a low LVEF (< 45%) was 36% at day-0, 39% at day-1, 38% at day-3 and 14% on the day of vasopressors weaning. At these time points, systolic arterial pressure was 118 [109; 127], 121 [116; 133], 123 [114; 132] and 130 [120; 134] mmHg, respectively. The prevalence of an impaired GLS (> − 16%) was 70% at day-0, 72% at day-1, 75% at day-3 and 58% at vasopressors weaning. Pooling all time points, GLS was correlated with blood lactate (rho = 0.369, 95% confidence interval: [0.213;0.506], p < 0.0001), and ScvO_2_ (rho = − 0.293 [− 0.457; − 0.110], p < 0.0021). At each measurement time, we classified the cardiac status into three groups: normal LVEF and normal GLS (LVEF_n_-GLS_n_, n = 40), normal LVEF and impaired GLS (LVEF_n_-GLS_impaired_ n = 52) and low LVEF and impaired GLS (LVEF_low_-GLS_impaired_, n = 36). Compared to the LVEF_n_-GLS_n_ group, the LVEF_n_-GLS_impaired_ group exhibited a lower cardiac index (2.6 [2.3;3.1] vs. 3.1 [2.6;3.9] L/min/m^2^, p = 0.03), a higher lactate level (1.7 [1.3;2.3] vs. 1.4 [1.0;2.1] mmol/L, p = 0.04) and lower ScvO2 (67 [63;74] vs. 72 [67;78]%, p = 0.02). In the LVEF_low_-GLS_impaired_ group, cardiac index was lower (2.4 [2.2;2.8] vs. 2.6 [2.3;3.1] L/min/m^2^, p = 0.03), lactate higher (2.8 [2.0;3.8] vs. 1.7 [1.3;2.3] mmol/L, p = 0.01) than in the LVEF_n_-GLS_impaired_ group and ScvO_2_ was not significantly different (69 [57;74] vs. 67 [63;74]%, p = 0.76) (Fig. 1).

**Conclusion:** During septic cardiomyopathy, GLS remains impaired in a large proportion of patients when vasopressors are stopped. An impaired GLS is associated with lower cardiac index and poorer tissue oxygenation irrespectively of the value of LVEF. This suggests that an impaired GLS has clinical meaning per se. The study is ongoing.

**Compliance with ethics regulations:** Yes in clinical research.
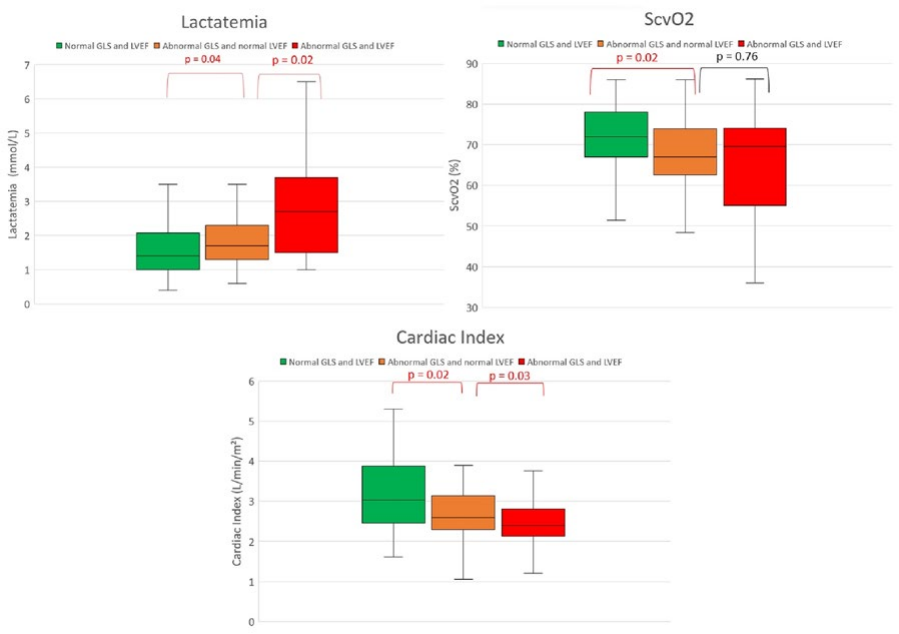


*Comparison of markers of tissue oxygenation between cardiac status subgroups*

### FC-115 Decrease in lactate level and norepinephrine dosage in the first 24 h of resuscitation is strongly associated with survival in 502 patients with refractory shock

#### HUGEROT Antonin^1^, CLERE-JEHL Raphaël^1^, SUBLON Cédric^1^, STEIN Julien^1^, GARIJO Carlos^1^, GUILLOT Max^1^, HERBRECHT Jean-Etienne^1^, JANSSEN-LANGENSTEIN Ralf^1^, LEROUX Justine^1^, PEREZ Yonatan^1^, SCHENCK-DHIF Maleka^1^, SCHNEIDER Francis^1^, CASTELAIN Vincent^1^

##### ^1^Hôpitaux Universitaires de Strasbourg, Strasbourg, France

###### Correspondence: Antonin HUGEROT (antonin.hugerot@gmail.com)

*Annals of Intensive Care* 2022, **12(1):**FC-115

**Rationale:** To determine the prognostic impact of maximum norepinephrine dosage received in the first 24 h following admission to the intensive care unit, regardless of indication, and to determine factors associated with in-ICU and in-hospital mortality.

**Patients and methods/Materials and methods:** All patients receiving norepinephrine in the first 24 h after admission to our University Hospital intensive care unit between January 1, 2015 and January 1, 2020 were included, retrospectively. We collected for each patient the maximum dosage of norepinephrine and all diagnostic, clinical and biological parameters of interest. Univariate and multivariate inferential statistical analysis was performed.

**Results:** We included 2009 patients, mean age 66 years, 40% of whom were women, and presenting with septic shock in 53% (n = 1073). Mean SAPS II reached 59 ± 19 points. Mechanical ventilation and renal replacement therapy were required in 77% and 23%, respectively. Intra-ICU and intra-hospital mortality were 32% and 39%, respectively. The median maximum norepinephrine dosage received during the first 24 h was 0.4 µg/kg/min (IQR 0.2–0.85). A significant increase in mortality was observed in uni- and multivariate analysis in patients who received more than 0.85 µg/kg/min of norepinephrine corresponding to the 3rd quartile distribution of maximum norepinephrine dosage, (in-ICU mortality 62%, p < 0.001). Among patients who received more than 0.85 µg/kg/min norepinephrine, defined as refractory shock, decreasing kinetics of lactate and norepinephrine dosage at 24 h were found to be highly significantly and independently associated with patient survival (in-ICU mortality 43%). On the other hand, the need for extrarenal purification or the use of adrenaline were independently associated with excess mortality in this group.

**Conclusion:** In our study, the threshold of 0.85 µg/kg/min is an independent marker of mortality in patients admitted to the ICU and could be considered to define refractory shock, regardless of diagnosis. However, when dosages higher than this threshold are used, lactate level and norepinephrine doses rapid decrease are strongly associated with a better prognosis. These findings need to be confirmed by further work in other patient populations before being used in clinical practice.

**Compliance with ethics regulations:** Yes in clinical research.
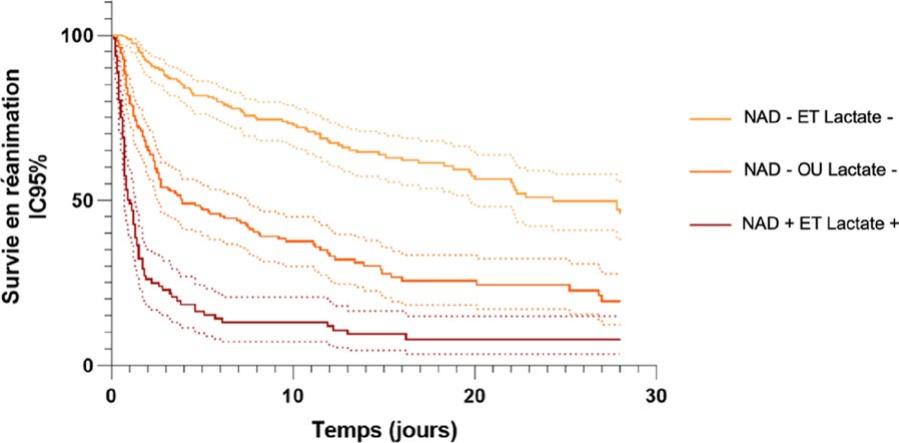


*Survival in patients with refractory shock according to kinetics of lactate level and norepinephrine dosage at 24 h*

### FC-116 Prehospital norepinephrine administration reduces 30-day mortality among septic shock patients

#### HAJJAR Adèle^1^, JOUFFROY Romain^1,8^, GILBERT Basile^2^, TOURTIER Jean Pierre^3^, BLOCH-LAINE Emmanuel^4^, ECOLLAN Patrick^5^, BOULARAN Josiane^6^, BOUNES Vincent^6^, VIVIEN Benoit^8^, GUEYE Papa^7^.

##### ^1^APHP - CHRU Ambroise Paré, Boulogne-Billancourt, France; ^2^Department of Emergency Medicine, SAMU 31, University Hospital of Toulouse, Toulouse, France; ^3^Paris Fire Brigade, Paris, France; ^4^Emergency Department, Cochin Hospital, Paris, France & Emergency Department, SMUR, Hôtel Dieu Hospital - Assistance Publique - Hôpitaux Paris, Paris, France; ^5^Intensive Care Unit, SMUR, Pitie Salpêtriere Hospital - Assistance Publique - Hôpitaux Paris, Paris, France; ^6^SAMU 31, Centre Hospitalier Intercommunal Castres-Mazamet, Castres, France; ^7^SAMU 972 University Hospital of Martinique,, Fort-De-France Martinique, France; ^8^Intensive Care Unit, Anaesthesiology, SAMU, Necker Enfants Malades Hospital, Assistance Publique - Hôpitaux Paris, Paris, France

###### Correspondence: Romain JOUFFROY (romain.jouffroy@gmail.com)

*Annals of Intensive Care* 2022, **12(1):**FC-116

**Rationale:** Despite differences in time of sepsis recognition, recent studies support that early initiation of norepinephrine in patients with septic shock (SS) improves outcome without adverse effects increase. This study aims to investigate the relationship between 30-day mortality in patients with SS and prehospital norepinephrine infusion in order to reach a mean blood pressure (MAP) at 65 mmHg at the end of the prehospital stage.

**Patients and methods/Materials and methods:** From April 06th, 2016 to December 31st, 2020, patients with SS requiring prehospital Mobile Intensive Care Unit intervention (MICU) were retrospectively analysed. To consider cofounders, propensity score method was used to assess the relationship between prehospital norepinephrine administration in order to reach a MAP at 65 mmHg at the end of the prehospital stage and 30-day mortality.

**Results:** Four hundred seventy-eight patients were retrospectively analysed, among which 309 patients (65%) were male. The mean age was 69 ± 15 years. Pulmonary, digestive, and urinary infections were suspected among 44%, 24% and 17% patients, respectively. One third of patients (n = 143) received prehospital norepinephrine administration with a median dose of 1.0 [0.5–2.0] mg.h^−1^, among which 84 (69%) were alive and 38 (31%) deceased on day 30 after hospital-admission. 30-day overall mortality was 30%. Cox regression analysis after propensity score showed a significant association between prehospital norepinephrine administration and 30-day mortality: adjusted hazard ratio of 0.42 [0.25–0.70], p < 10−3. Multivariate logistic regression of IPTW retrieved a significant decrease of 30-day mortality among prehospital norepinephrine group: ORa = 0.75 [0.70–0.79], p < 10−3.

**Conclusion:** In this study, we report that prehospital norepinephrine infusion in order to reach a MAP at 65 mmHg at the end of the prehospital stage is associated with a decrease in 30-day mortality in patients with SS cared for by a MICU in the prehospital setting. Further prospective studies are needed to confirm that very early norepinephrine infusion allows to decrease septic shock mortality.

**Compliance with ethics regulations:** Yes in clinical research.
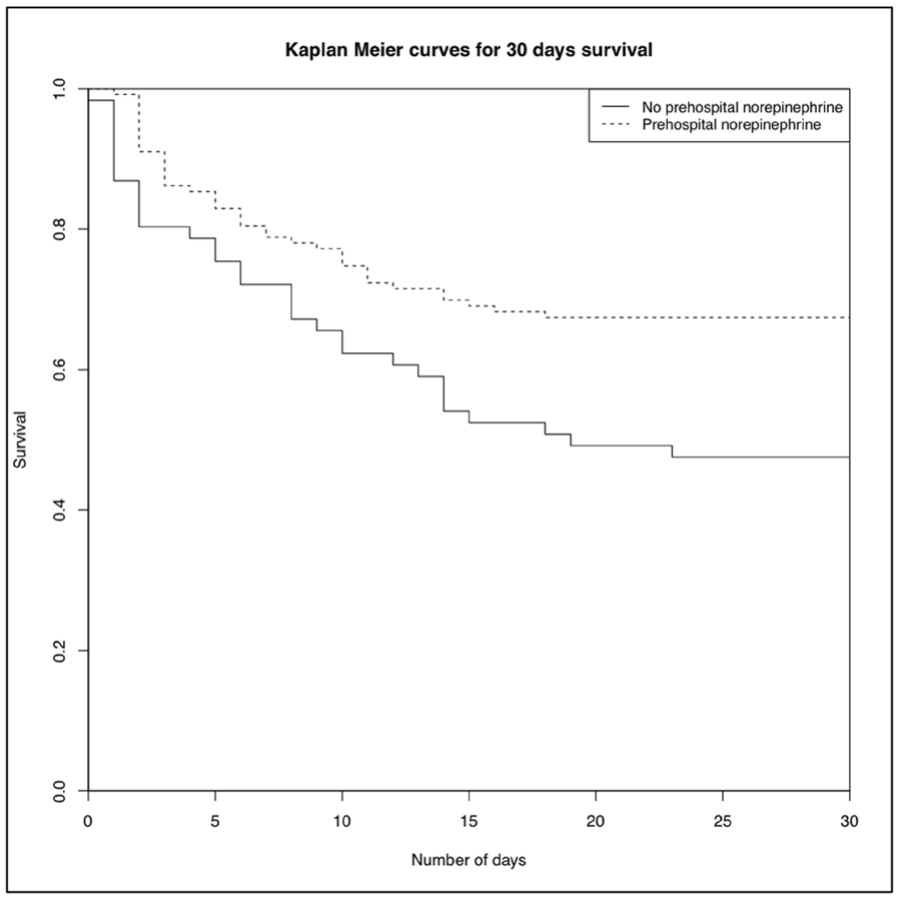


Kaplan Meier curves for 30-days survival between patients with prehospital norepinephrine administration and those without prehospital norepinephrine administration

### FC-117 Time for restauration of radial arterial pressure after a transient arm vascular occlusion correlates with outcome in critically ill patients: preliminary results of an observational study

#### LAI Christopher^1^, SHI Rui^1^, TEBOUL Jean-Louis^1^, MORETTO Francesca^1^, GUÉRIN Laurent^1^, XAVIER Monnet^1^

##### ^1^AP-HP, Service de médecine intensive-réanimation, Hôpital de Bicêtre, DMU 4 CORREVE, Inserm UMR S_999, FHU SEPSIS, CARMAS, Université Paris-Saclay, Le Kremlin-Bicêtre, France

###### Correspondence: Christopher LAI (christopher.lai@aphp.fr)

*Annals of Intensive Care* 2022, **12(1):**FC-117

**Rationale:** Impairment of vascular reactivity is one of the characteristics of septic shock and is associated with a poor outcome in critically ill patients. It can be assessed by the resaturation slope of the muscular tissue oxygen saturation after a transient vascular occlusion (TVO). Another means for assessing vascular reactivity might be to observe the time of the increase in radial arterial pressure after a TVO at the arm level.

**Patients and methods/Materials and methods:** Mechanically ventilated patients hospitalized in intensive care unit and equipped with a radial artery catheter were prospectively included. A brachial cuff was rapidly inflated to induce a transient arterial stop-flow. Arterial pressure thus decreased down to the level of mean systemic pressure (Pms_arm_). After 60 s of TVO, the cuff was abruptly deflated and time to return from Pms_arm_ to baseline systolic arterial pressure was measured (T_revasc_).

**Results:** We included 41 patients, among whom 19 (46%) where in shock, including 13 (32%) septic shock, 10 (24%) had acute respiratory distress syndrome and 9 (22%) were intubated for neurological impairment. Norepinephrine was infused in 25 (61%) patients (0.3 [0.17–1.10] μg/kg/min). Measurements were obtained 3 (2–6) days after onset of mechanical ventilation or vasopressors infusion. Mean arterial pressure was 84 ± 12 mmHg, Pms_arm_ was 29 ± 9 mmHg, central venous pressure was 12 ± 4 mmHg and the (Pms_arm_-CVP) gradient was 16 ± 8 mmHg, with no difference between patients with or without shock. In the whole population, T_revasc_ was 26 (20–33) sec. It was significantly increased in patients with shock compared to those without shock (33 ± 18 *vs.* 23 ± 10 s., respectively, p = 0.028). Among patients with shock, T_revasc_ was longer in patients with septic shock than in the other ones (33 (28–44) *vs.* 16 (9–30) sec., respectively, p = 0.048). T_revasc_ was significantly increased in the 21 (51%) patients who died (11 patients with shock) compared to survivors (33 ± 19 vs. 23 ± 10 s., respectively, p = 0.037).

**Conclusion:** In critically ill patients under mechanical ventilation, T_revasc_ was increased in patients with shock compared to patients without shock, especially in septic shock patients. T_revasc_ was also higher in non-survivors. This new variable might indicate vasoreactivity at bedside and, as such, be associated with severity and mortality. More data are needed to confirm these preliminary results and inclusions are ongoing.

**Compliance with ethics regulations:** Yes in clinical research.

### FC-118 Vitamin C improves microvascular reactivity and peripheral tissue perfusion in septic shock patients

#### LAVILLEGRAND Jean-Rémi^1^, RAIA Lisa^1^, URBINA Tomas^1^, HARIRI Geoffroy^1^, GABARRE Paul^1^, BONNY Vincent^1^, BIGÉ Naike^1^, BAUDEL Jean Luc^1^, BRUNEEL Arnaud^2^, DUPRE Thierry^1^, GUIDET Bertrand^1^, MAURY Eric^1^, AIT OUFELLA Hafid^1^

##### ^1^APHP-CHU Saint Antoine, Paris, France; ^2^APHP-CHU Bichat, Paris, France

###### Correspondence: Jean-Rémi LAVILLEGRAND (jrlavillegrand@gmail.com)

*Annals of Intensive Care* 2022, **12(1):**FC-118

**Rationale:** Vitamin C has potential protective effects through antioxidant and anti-inflammatory properties. However, the effect of vitamin C supplementation on microvascular function and peripheral tissue perfusion in human sepsis remains unknown. We aimed to determine vitamin C effect on microvascular endothelial dysfunction and peripheral tissue perfusion in septic shock patients.

**Patients and methods/Materials and methods:** Patients with septic shock were prospectively included after initial resuscitation. Bedside peripheral tissue perfusion and skin microvascular reactivity in response to acetylcholine iontophoresis in the forearm area were measured before and 1 h after intravenous vitamin C supplementation (40 mg/kg). Norepinephrine dose was not modified during the studied period.

**Results:** We included 30 patients with septic shock. SOFA score was 11 [8–14], SAPS II was 66 [54–79], and in-hospital mortality was 33%. Half of these patients had vitamin C deficiency at inclusion. Vitamin C supplementation strongly improved microvascular reactivity (AUC 2263 [430–4246] vs 5362 [1744–10585] UI, p = 0.0004). In addition, vitamin C supplementation improved mottling score (p = 0.06), finger-tip (p = 0.0003) and knee capillary refill time (3.7 [2.6–5.5] vs 2.9 [1.9–4.7] s, p < 0.0001), as well as and central-to-periphery temperature gradient (6.1 [4.9–7.4] vs 4.6 [3.4–7.0] °C, p < 0.0001). The beneficial effects of vitamin C were observed both in patients with or without vitamin C deficiency.

**Conclusion:** In septic shock patients being resuscitated, vitamin C supplementation improved peripheral tissue perfusion and microvascular reactivity whatever plasma levels of vitamin C.

**Compliance with ethics regulations:** Yes in clinical research.

### FC-119 Noradrenaline dose change-related effects on mean arterial pressure: preliminary results from the NoVaMAP study

#### MORETTO Francesca^1^, SHI Rui^1^, TEBOUL Jean-Louis ^1^, PAVOT Arthur^1^, LAI Christopher^1^, FAGE Nicolas^1^, PHAM Tài^1^, MONNET Xavier^1^

##### ^1^Université Paris-Saclay, AP-HP, Service de médecine intensive-réanimation, Hôpital de Bicêtre, DMU CORREVE, Inserm UMR S_999, FHU SEPSIS, Groupe de recherche clinique CARMAS, Le Kremlin-Bicêtre, France

###### Correspondence: Francesca MORETTO (francescamoretto90@gmail.com)

*Annals of Intensive Care* 2022, **12(1):**FC-119

**Rationale:** Responsiveness to norepinephrine (NE) dose change in terms of mean arterial pressure (MAP) change is highly variable among patients with acute circulatory failure. This preliminary study aimed to investigate the factors influencing the pharmacodynamic effect of NE on MAP in critically ill patients.

**Patients and methods/Materials and methods:** This monocentric, observational, prospective study was conducted in patients with acute circulatory failure requiring NE and invasive arterial pressure monitoring. To characterize the responsiveness of MAP to NE change, the maximal amplitude of MAP change over the amplitude of change in the NE (deltaMAPmax/deltaNE) was calculated.

**Results:** From January to July 2021, 29 patients presenting 86 occurrences of change in the NE dose, including 55 dose-increases and 31 dose-decreases, were included. The most common origin of shock was sepsis in 59 (69%) cases, followed by hypovolemic/hemorrhagic shock in 16 (18%) cases and non-septic vasoplegia in 11 (13%) cases. Septic shock was characterized by significantly lower baseline values of MAP (66 [58–86] vs. 82 [71–103] mmHg) and of diastolic arterial pressure (50 [44–64] vs. 64 [55–78] mmHg) and by a larger amplitude of the change in NE dose (0.08 [0.05–0.12] vs. 0.04 [0.03–0.06] µg/kg/min). DeltaMAPmax/deltaNE was significantly lower in septic shock than in hypovolemic/hemorrhagic shock or non-septic vasoplegia (315 [161–590] vs. 575 [401–776] vs. 446 [336–1119] mmHg/µg/kg/min, respectively, p = 0.03). At multiple linear regression analysis, preexisting hypertension, body temperature at the study time and shock etiology were associated with deltaMAPmax/deltaNE (p = 0.002). In septic shock patients, body temperature and C-reactive protein levels independently influenced deltaMAPmax/deltaNE (p = 0.003).

**Conclusion:** Septic shock is characterized by lower vascular reactivity compared to other shock etiologies and MAP responsiveness to NE change is not identical to other types of distributive shocks. The results of this ongoing study will be entered in a model of artificial intelligence with the aim of adapting the dose of NE required to reach an individualized target level of MAP.

**Compliance with ethics regulations:** Yes in clinical research.

### FC-120 Is precariousness a risk factor for COVID-19 mortality in intensive care units?

#### GILLIBERT Adrien^1^, LAINE Laurent^1^

##### ^1^Delafontaine, Paris, France

###### Correspondence: Adrien GILLIBERT (gillibertadrien@gmail.com)

*Annals of Intensive Care* 2022, **12(1):**FC-120

**Rationale:** During the SARS-CoV-2 pandemic, the first wave overwhelmed hospitals in Paris area (Ile-de-France) with a variable impact depending on the territory. Several studies highlighted variable ICU mortality rates during COVID-19 surges across territories (10 to 60%) with higher rates in those most affected by poverty^1,2^. We assessed the impact of precariousness, as an independent risk factor, on mortality linked to Covid-19 between ICUs at Delafontaine hospital, Saint-Denis, and Ambroise Paré hospital, Boulogne-Billancourt, two Paris suburbs.

**Patients and methods/Materials and methods:** We carry out a retrospective observational cohort study of consecutive ICU patients aged ≥ 18 years admitted at Delafontaine and Ambroise Paré hospitals during the first wave of the Covid-19 outbreak in order to compare mortality rates according to predefined risk factors (age, diabetes, arterial hypertension, BMI, active solid or haematological cancer, IGS2, poverty rate at the threshold of 60% (%) according to the island grouped for statistical information (IRIS)37 of the patient, invasive ventilation or not) that include precariousness.

**Results:** We found a difference for mortality in the two groups (p = 0,033) and with the univariate analysis the poverty rate was a risk factor for mortality at 90 days (p = 0,04). But we could not find a statistical relation between deprivation and mortality at 90 days in Covid-19 in intensive care in multivariate analysis.

**Conclusion:** Despite lake of significative statistical relationship between precariousness and covid-19 mortality in intensive care, it seems we could find correlation between importance of precariousness and mortality. We should realise a prospective study with more important population to find a relationship between deprivation and mortality at Covid-19 in intensive care.

**Reference 1:** 1. Quenot, Jean-Pierre et al. “Influence of deprivation on initial severity and prognosis of patients admitted to the ICU: the prospective, multicentre, observational IVOIRE cohort study.” Annals of intensive care vol. 10,1 20. 11 Feb. 2020, doi:10.1186/s.

**Reference 2:** 2. Rahi, Mayda et al. “Sociodemographic characteristics and transmission risk factors in patients hospitalized for COVID-19 before and during the lockdown in France.” BMC infectious diseases vol. 21,1 812. 13 Aug. 2021, https://doi.org/10.1186/s12879-021-06419-7

**Compliance with ethics regulations:** Yes in clinical research.
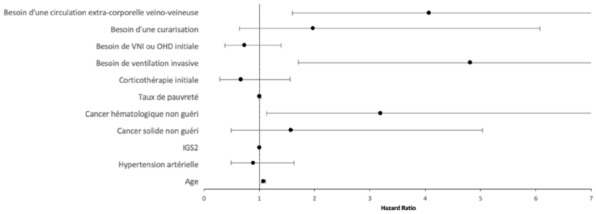


*Forrest plot for relationship between mortality at 90 days et risk factors in multivariate analysis*

### FC-121 Management of a massive inflow of patients in a very large cluster of covid-19 cases

#### POINTURIER Valentin^1^, POIDEVIN Antoine^1^, DEGOUL Samuel^1^, VIROT Edouard^1^, MOOTIEN Yoganaden^1^, PINTO Luis^1^, MATHIEN Cyrille^1^, LABRO Guylaine^1^, RABOUEL Yannick^1^, ETIENNE Arnaud^1^, KUTEIFAN Khaldoun^1^

##### ^1^Hôpital Emile Muller, Mulhouse, France

###### Correspondence: Valentin POINTURIER (valentin.pointurier@ghrmsa.fr)

*Annals of Intensive Care* 2022, **12(1):**FC-121

**Rationale:** Our general tertiary hospital had to face an unprecedented wave of respiratory distress in early 2020 that quickly overwhelmed its 40 ICU beds. The aim of this study was to report on ICU admission process and in-flow management at time of Covid-19 outbreak.

**Patients and methods/Materials and methods:** We conducted a retrospective analysis of every consecutive patient requiring the advice of the intensivist team between March 2 and May 1, 2020 from a call register filled prospectively on daily basis.

**Results:** Local ICU capacities have more than doubled (92 beds) thanks to the deprogramming of the scheduled surgical activity and the deployment of a military mobile hospital. Medical ICU physician on duty centralized all calls for Covid-19 admission. Priority was given to patient requiring immediate intubation. The others were placed on a waiting list according to their severity and expected prognosis based on their comorbidities, their frailty and their age until a ventilator was available. At the peak, 46 patients required the advice of the intensivist daily. A total of 591 patients were proposed for ICU admission. 75% had acute respiratory distress due to severe Covid-19, other were medical (14%) and surgical (11%) non covid related situations. ICU admission was decided for 370 patients. Mean age was 62 years (± 12), 69% were male. The most serious patients and those with contraindications to transfer were kept in local ICU (154 patients). 244 patients were transferred to France, Germany, Switzerland, and Luxembourg representing more than one third of all French transfers of the Covid-19 first wave. The patients managed in standard ward were older (mean age: 69 years (± 12)) and had more significant cardiovascular diseases (12,7% vs 5%), malignant tumors (10,5% vs 3,1%) and hematological diseases (8,2% vs 4,7%).

**Conclusion:** In situation of high tension on ICU capacities, priority must be given to increasing resources and optimizing means. The establishment of triage rules respecting the ethical principles of equality, beneficence, and transparency guarantees equitable access to intensive care and maximization of the number of lives saved.

**Compliance with ethics regulations:** Yes in clinical research.

### FC-122 Evaluation of communication training in paediatric intensive care

#### DUCERT Floriane^1^, OHNOUNA Rachel^1^, VANEL Noémie^1^, GRIMALDI Céline^1^, RONCIN Cesar^1^, MICHEL Fabrice^1^

##### ^1^Hopital Timone 2, Marseille, France

###### Correspondence: Fabrice MICHEL (fabrice.michel@ap-hm.fr)

*Annals of Intensive Care* 2022, **12(1):**FC-122

**Rationale:** Communication with patients requires interpersonal skills and is a challenge for physicians, especially in paediatric critical care units. The need for good communication contrasts with the lack of training in communication skills in medical studies, and there is little experience in the literature of evaluating communication with families training in paediatric critical care. The main objective of this study was to heteroevaluate the impact of communication training for residents in a paediatric intensive care unit.

**Patients and methods/Materials and methods:** 14 residents were evaluated on their performance in communicating with families through video recordings of simulated breaking bad news to parents, before and after a 2-day training in communication with families including theoretical teaching and situational practice in relational simulation. The skills were also evaluated 3 month after the training to measure maintenance of acquisition over time. Residents were confronted to 3 standardized situations in a predetermined order, different for each of them. The ‘’modified Breaking Bad News Assessment Scale’’ (mBAS) was used as the evaluation tool scored by resident an not involved in the training, and analyzing the videos in a totally random order. The duration of interviews, speaking and silence times were recorded. Self-assessment was also performed using the ‘’Breaking Bad News’’ and ‘’Cungi and Rey’’ scales scored after each session.

**Results:** Both self-assessment scores showed important and significant improvement of the participants after the training, and maintenance of acquisitions over time. The global mBAS score decrease (corresponding to skills improvement) non-significantly, immediately after training (55.8 ± 8.1 vs. 49.0 ± 8.0; p = 0.071), but there was a significant improvement in two of the five score domains (A: setting the scene, C: eliciting concerns). Stress of residents after interviews decreased non significantly between the 3 measures (5,0 ± 1,7;4,4 ± 2,1;3,7 ± 1; p = 0,214). Duration of interviews and silence did not vary significantly.

**Conclusion:** Our study showed that training in communication with parents in the paediatric intensive care unit resulted in an improvement in the resident’s autoevaluation. This effect was less pronounced during heteroevaluation, which should encourage teachers to evaluate their training by objective methods.

**Compliance with ethics regulations:** Yes in clinical research.

### FC-123 Mental health problems among Tunisian healthcare workers in the second year of Covid-19 pandemic

#### ZGHIDI Marwa^1^, BEN SAIDA Imen^1,2^, HAMDI Dhouha^1^, BOUBTANE Rihab^1^, BOUSSARSAR Mohamed^1,2^

##### ^1^Farhat Hached University Hospital, Medical Intensive Care Unit, Sousse, TUNISIA, Sousse, Tunisie; ^2^Research Laboratory N° LR12SP09. Heart Failure. Farhat Hached University Hospital, Sousse, Tunisia, Sousse, Tunisie

###### Correspondence: Marwa ZGHIDI (marwa_zghidi@outlook.fr)

*Annals of Intensive Care* 2022, **12(1):**FC-123

**Rationale:** The fear to contaminate and being contaminated, the quality of preparedness, the availability of personal protection equipment (EPI), diagnostic and therapeutic blur of an emergent disease and patients’ severity leading to high workload have added a burden of acute emotional stress in healthcare workers. The aim was to evaluate the impact of Covid-19 pandemic on healthcare workers mental health and identify factors associated with adverse mental outcomes.

**Patients and methods/Materials and methods:** It is a cross-sectional study conducted in critical care and emergency departments of 3 hospitals (Hached; Sahloul and Military hospital of Instruction of Tunis) from January 18, 2021, to March 13, 2021.Mental health outcomes: depression, anxiety, stress, post-traumatic stress disorder and burnout were assessed by depression anxiety stress scales (DASS), Impact of event scale revised(IES-r) and Maslach Burn out Inventory (MBI), respectively. Multivariate logistic regression analysis was performed to identify factors associated with those adverse mental health outcomes.

**Results:** One hundred and ten healthcare workers filled the questionnaire with a response rate at 86.6%. Respondents characteristics’ were: median age, 32 [28–37] years; female 68(61.8%); nurses 57(51.8%); 63(57.3%) worked in emergency departments. Fifty-two respondents (47.3%) had been contaminated by the SARS COV2. 88.2% of the respondents reported a feeling of fear of personal and family physical safety,74.5% felt an increased work overload and 72.7% confirmed a poor working condition. 43 respondents (43.6%) were considering quitting their profession because of severe psychological distress. 48.2%, 42.7% and 48.98% of respondents had respectively severe stress, severe anxiety and severe burnout. 49.1% of participants had probable PTSD according to IES-r score. On the multivariate regression, female sex (OR, 9.7; CI [2.5–36.7]; p < 0.001), age (OR, 0.083; IC [0.013–0.537]; p = 0.009), work overload (OR, 5.6; IC [1.3–23.7]; p = 0.018), were factors associated with stress. Age (OR, 0.6; IC [0.5–0.87]; p = 0.000), work overload (OR, 29; IC [3–63]; p = 0.002); change of work location (OR, 11.3; IC [1.4–88.7]; p = 0.021); poor work conditions (OR,20.7; IC [12–66]; p < 0.001) were associated with anxiety. Lack of psychological support (OR, 5.8; IC [2–16]; p < 0.001) was the only risk factor associated with depression. Age (OR, 0.6; IC [0.4–0.73]; p < 0.001); fear of personal and family safety (OR,7.2; IC [1.1–15]; p < 0.001); working with suspected or confirmed patients with COVID-19 (OR, 29; IC [2–69]; p = 0.009) were factors independently associated with PTSD. Female sex (OR, 1.1; IC [1.04–1.2]; p = 0.003), poor work conditions (OR,3.5; IC [1.7–4.6]; p = 0.047) were predictors of burnout.

**Conclusion:** The current study highlights that health care workers are at high risk of psychological distress during the covid-19 outbreak. Early psychological interventions may be beneficial.

**Compliance with ethics regulations:** Yes in clinical research.

### FC-124 Prevalence of post-traumatic stress disorder in intensive care workers

#### DELTOUR Victoire^1,3^, LAURENT Alexandra^1,2^, POUJOL Anne-Laure^3,4^

##### ^1^Université de Bourgogne Franche-Comté, Psy-DREPI EA 7458, Dijon, France; ^2^Service de réanimation chirurgicale, CHU Dijon, Dijon, France; ^3^Ecole de Psychologues Praticiens, Paris, France; ^4^Service de réanimation chirurgicale polyvalente, La pitié Salpêtrière, Paris, France

###### Correspondence: Victoire DELTOUR (victoire.deltour@hotmail.fr)

*Annals of Intensive Care* 2022, **12(1):**FC-124

**Rationale:** Working in intensive care unit confronts professionals with extreme and unpredictable situations that have a traumatic dimension and constitute a risk of post-traumatic stress disorder (PTSD). Since 2019 this extreme context has been accentuated by unprecedented epidemic waves. We will present a systematic review to better identify the traumatic impact of ICU on healthcare and associated factors before and during the health crisis.

**Patients and methods/Materials and methods:** We used Pubmed; Science direct and Ovid (APA) databases to identify all quantitative, qualitative and mixed studies examining PTSD in Intensive care workers between 2009 and 2022.

**Results:** Of the 704 articles identified, only 8 met our inclusion criteria. 4 studies that were conducted before the COVID period showed that 3.3% to 18.2% of the surveyed intensive care workers had PTSD symptoms. The associated risk factors were life-threatening emergencies; exposure to death; management of organ and body donations; and situations experienced by caregivers as therapeutic overkill. Individual factors such as personality also appear to play a role in the development of traumatic symptomatology (Cho and Kang 2017). Four studies conducted during the COVID period showed a significant increase in the prevalence of trauma among intensive care workers, with between 16% and 73.3% showing traumatic symptoms. The already potentially traumatic factors of ICU were exacerbated and others specific to the crisis increased this risk (Carmassi et al. 2022; Laurent et al. 2022). Feeling safe in one's department, supported by one's team, and having sufficient material and human backup seemed to be protective factors (Heesakkers et al. 2021).

**Conclusion:** Intensive care workers seem to be particularly at risk of developing PTSD, and this has increased during the health crisis period. However, there are few studies on this subject and they deserve to be developed in order to propose prevention and adapted care devices.

**Compliance with ethics regulations:** Yes in clinical research.

### FC-125 A qualitative study of reinforcement workers’ perceptions and experiences of working in intensive care during the COVID-19 pandemic: a PsyCOVID-ICU substudy

#### PERRAUD Florian^1^, ECARNOT Fiona^2,3^, LOISEAU Mélanie^4^, LAURENT Alexandra^5,6^, FOURNIER Alicia^5^, LHEUREUX Florent^7^, BINQUET Christine^8^, RIGAUD Jean-Philippe^9,10^, MEUNIER-BEILLARD Nicolas^11,12^, QUENOT Jean-Pierre^11,13,14,15^

##### ^1^University Hospital Dijon, and Université de Bourgogne Franche-Comté, Dijon, France, Dijon, France; ^2^Department of Cardiology, University Hospital Besançon, Besançon, France; ^3^EA3920, University of Burgundy-Franche-Comté, Besaançon, France; ^4^Service de Médecine Légale, University Hospital Dijon; Cellule d’Urgence Médico-Psychologique de Bourgogne Franche-Comté, Dijon, France; ^5^Laboratoire de Psychologie: Dynamiques Relationnelles Et Processus Identitaires (PsyDREPI), Université de Bourgogne Franche-Comté, Dijon, France; ^6^Department of Anaesthesiology and Critical Care Medicine, University Hospital Dijon, Dijon, France; ^7^Laboratoire de Psychologie, University of Burgundy-Franche-Comté, Besançon, France; ^8^Inserm CIC1432, module Épidémiologie Clinique (CIC-EC)- CHU Dijon-Bourgogne, UFR des Sciences de Santé, Dijon, France; ^9^Service de Médecine Intensive-Réanimation, Hospital Centre of Dieppe, Dieppe, France; ^10^Espace de Réflexion Éthique de Normandie, Université de Caen, Caen, France; ^11^CIC 1432, Clinical Epidemiology, University of Burgundy, Dijon, France; ^12^Direction de la Recherche Clinique et de l’Innovation, University Hospital Dijon, Dijon, France; ^13^Service de Médecine Intensive-Réanimation, University Hospital Dijon, Dijon, France; ^14^Equipe Lipness, centre de recherche INSERM UMR1231 et LabEx LipSTIC, université de Bourgogne-Franche Comté, Dijon, France; ^15^Espace de Réflexion Éthique Bourgogne Franche-Comté (EREBFC), Besançon, France.

###### Correspondence: Florian PERRAUD (flperraud@gmail.com)

*Annals of Intensive Care* 2022, **12(1):**FC-125

**Rationale:** During the COVID pandemic, many hospitals had to mobilize reinforcement healthcare workers (HCW), especially in intensive care units (ICUs). We investigated the perceptions and experiences of reinforcement workers deployed to ICUs, and the impact of deployment on their personal and professional lives.

**Patients and methods/Materials and methods:** For this qualitative study, a random sample of reinforcement workers was drawn from 4 centres participating in the larger PsyCOVID-ICU study. These HCW worked in the intensive care unit as support staff during either of the two waves of 2020 (March to May 2020 (wave 1) or September to November 2020 (wave 2)) in any of the four participating centres. Individual semi-structured interviews were held by telephone, recorded, transcribed and analyzed by thematic analysis.

**Results:** Thirty interviews were performed from April to May 2021 (22 nurses, 2 anesthesiology nurses, 6 nurses’ aides). Average age was 36.8 ± 9.5 years; 7 participants had no ICU experience. Four major themes emerged, namely: (1) difficulties with integration, especially for those with no ICU experience; (2) lack of training; (3) difficulties with management, notably a feeling of insufficient communication; (4) mental distress relating to the unusual work and fear of contaminating their entourage.

**Conclusion:** HCWs deployed as reinforcements to ICUs at the height of the pandemic had a unique experience of the crisis, and identified important gaps in organization and preparation. They also suffered from a marked lack of training, given the stakes in the management of critically ill patients in the ICU.

**Compliance with ethics regulations:** Yes in clinical research.

### FC-126 Direct OptimizatioN of cOmmunicaTion uSing PersonAlized Multimodal feedback between investigators to increase efficiency (DO NOT SPAM study)

#### MULLER Grégoire^1,2^, TAVERNIER Elsa^3,4^, LECLERC Marie^5^, BOULAIN Thierry^1,2^, EHRMANN Stephan^6^

##### ^1^Centre Hospitalier Régional d'Orléans, Orléans, France; ^2^CRICS-TriggerSEP F-CRIN research network, Tours, France; ^3^Clinical Investigation Center, INSERM 1415, CHRU Tours, Tours, France; ^4^Methods in Patients-Centered Outcomes and Health Research, INSERM UMR 1246, Nantes, France; ^5^Délégation à la Recherche Clinique et à l’Innovation, CHRU Tours, Tours, France; ^6^Médecine Intensive Réanimation, CIC 1415, CRICS-TriggerSEP F-CRIN research network and Centre d'étude des Pathologies Respiratoires, INSERM U1100, Université de Tours, Centre Hospitalier Régional Universitaire de Tours, Tours, France

###### Correspondence: Grégoire MULLER (muller.gregoire.chro@protonmail.com)

*Annals of Intensive Care* 2022, **12(1):**FC-126

**Rationale:** Only few clinical trials are completed in the expected timeframe, in part because of low inclusion rates. The specific context of “emergency trials” (short inclusion timeframes, very selective criterion) aside of time pressure associated with critical illness represents an extra challenge. Studies evaluating means to improve trial recruitment were performed outside of the ICU setting and aimed to increase the rate of patient consent rather than targeting investigator motivation. We hypothesize that an optimized communication strategy targeted at increasing investigator motivation may significantly improve trial recruitment.

**Patients and methods/Materials and methods:** Study centres participating in the EVERDAC trial (evaluating early vs differed arterial catheter in ICU patients with acute circulatory failure, NCT03680963),[1] were randomized between two communication strategies: conventional communication or upgraded communication strategy (intervention) including personalized computer-generated emails of congratulations for inclusions according to inclusion rate. The primary outcome was the number of patients included in the EVERDAC trial 12 months after centre opening, analyzed in a quasi-Poisson regression considering the number of ICU beds available in the unit as an offset.

**Results:** 264 patients were included in the EVERDAC study in the nine participating centres, 119 from intervention centres (median 28.5 [18.0;46.9]), 145 from conventional centres (median 23.0 [7.0;25.0] (RR 0.53 [0.11;2.34], p = 0.42, Fig. 1). Respectively 115 and 145 emails were addressed to the investigator with respectively 13.0 [13.0; 14.0] and 8.0 [8.0;10.0] recipients in carbon copy (coinvestigators and study team).

**Discussion:** Our study failed to demonstrate that an optimized communication strategy could improve inclusion rate. First our study may be underpowered. Secondly, one large centre with no inclusion randomized to intervention and smaller centre which made by far most of the trial inclusions randomized to the control group, may represent outliers significantly impacting overall results. One could argue these centres would have included the same inclusion rate whatever the communication strategy applied (ie, our communication strategy may improve inclusions in centres with middle rate of inclusion). However, results were not modified when excluding those outlier centres from analysis (RR 0.6 [0.23;2.34]). Thirdly, the number of emails sent could have a negative effect on inclusions.

**Conclusion:** This study failed to demonstrate a benefit of an email based optimized communication strategy between the study coordinator and site investigators and staff on trial inclusion rate in the ICU.

**Reference 1:** Muller G, BMJ Open. 2021;11:e044719.

**Compliance with ethics regulations:** N/A.
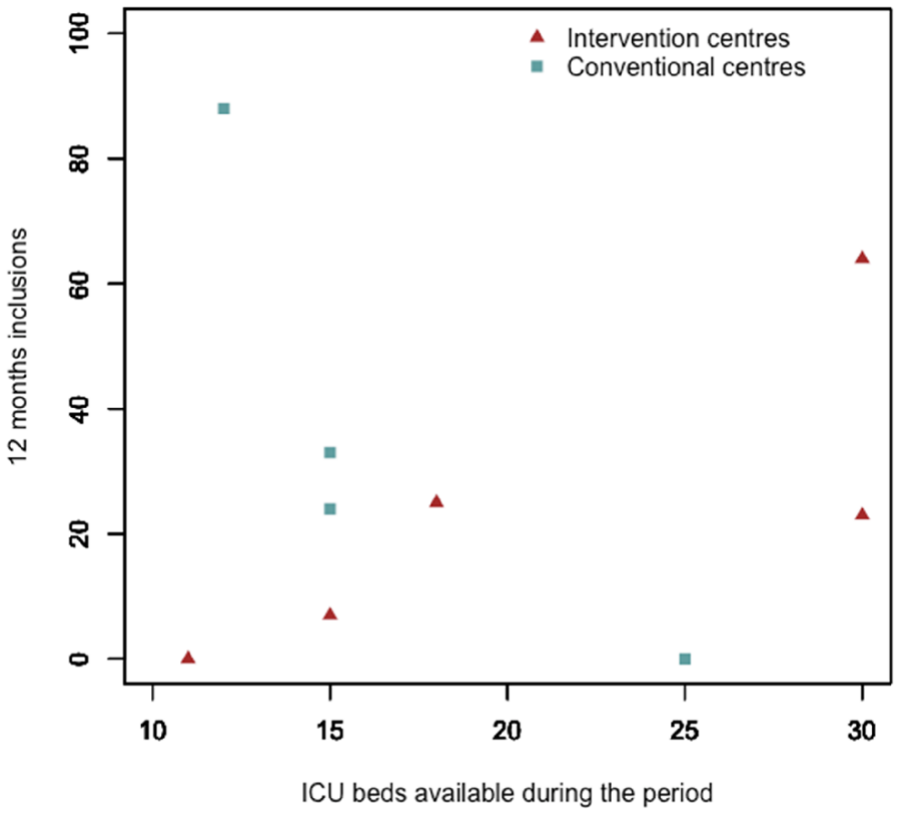


*12 months inclusion according to the number of beds available in the ICU*

### FC-127 Epidemiology of people living with HIV admitted to an ICU of the Caribbean region between 2015 and 2019

#### TEISSEDRE Célia^1^, CARLES Michel^1^, LAMAURY Isabelle^1^, TRESSIERES Benoit^1^, MELOT Bénédicte^1^

##### ^1^CHU de la Guadeloupe, Les Abymes, Guadeloupe

###### Correspondence: Célia TEISSEDRE (melotb@gmail.com)

*Annals of Intensive Care* 2022, **12(1):**FC-127

**Rationale:** People leaving with HIV (PLWHIV) from the Caribbean, due to the geographical and historical situation of this region, have very specific epidemiological characteristics, including a higher prevalence compared to other regions. The primary objective of this work was to describe the characteristics of PLWHIV treated in the ICU of our hospital to improve their care. The secondary objectives were to study factors associated to prognosis and assess the impact of HIV on ICU morbidity and mortality.

**Patients and methods/Materials and methods:** We included all patients treated in the ICU of our hospital between January 2015 and December 2019 with the main or associated diagnosis “HIV” during their hospitalization. We first carried out a descriptive analysis using paper and computer files. We then performed a bivariate and multivariate analysis to study the variables associated with mortality in PLWHIV and then to compare the prognosis in intensive care, at 1 month and at 1 year in PLWHIV versus non-HIV, adjusted for the other variables.

**Results:** A total 114 PLWHIV and 228 patients without HIV were analyzed, i.e. an average of 23 PLWHIV per year, including 9 patients diagnosed at the time of their admission to the ICU, and 2 with a “fortuitous” diagnosis. The median age was 54 years old, the sex ratio 2, the median CD4 count was 218/mm^3^ and the median viral load 185 copies, 1/4 had co-infection with hepatitis B, 81% had at least 1 opportunistic infection, 1/3 had a renal replacement therapy (RRT), half a vasopressor therapy, 3/4 mechanical ventilation. The mortality in the ICU was 31% (Table). PLWHIV were significantly more ventilated, on vasopressor therapy, and on RRT than non-HIV patients, and variables associated to higher ICU mortality in PLWHIV were intubation, RRT, vasopressor therapy, and single hospitalizations. However, mortality in ICU, at 1 month and 1 year, although slightly higher in PLWHIV didn't vary significantly.

**Conclusion:** PLWHIV hospitalized in the ICU of our hospital are frequent, more severe and require heavier care, compared to non-HIV patients. Factors related to the poor prognosis, like diagnosis delay due to HIV lack of knowledge, should be assessed. Due to the large number of PLWHIV admitted to the ICU (2 per month) in this area, specific approaches should be implemented: adaptation of human resources, infectious disease referent, specific and regular training of ICU physicians and paramedics to increase their skills regarding HIV specific aspects, in order to improve PLWHIV ICU prognosis.

**Compliance with ethics regulations:** Yes in clinical research.
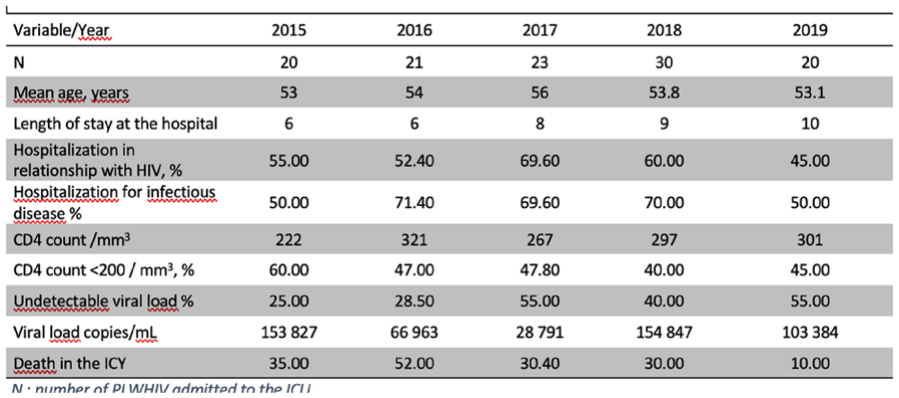


*Table: Evolution of the characteristics of PLWHIV admitted to the ICU of a Caribbean hospital from 2015 to 2019, n* = *114.*

### FC-128 Effect of antibiotics on catheter associated urinary tract infections in the ICU: a non-inferiority retrospective trial

#### PONCELET Arthur^1^, MICHEL Charlotte^1^, HITES Maya^1^, PLETSCHETTE Zoé^1^, MARTINY Delphine^1^, CRÉTEUR Jacques^1^, JACOBS Frédérique^1^, GRIMALDI David^1^

##### ^1^CUB Erasme - ULB, Bruxelles, Belgique

###### Correspondence: Arthur PONCELET (arthur.poncelet@ulb.be)

*Annals of Intensive Care* 2022, **12(1):**FC-128

**Rationale:** Catheter associated urinary tract infections (CAUTI) are the most frequent nosocomial infections, although rarely severe. In this setting the benefit/risk ratio of antibiotic therapy is unclear since overconsumption leads to antimicrobial resistance. Catheter associated asymptomatic bacteriuria should not be treated but distinction between asymptomatic bacteriuria and urinary infection is not easily made in ICU patients. We therefore hypothesized that the abstention of treatment of positive urine cultures associated with systemic signs of inflammation is not related with an increased sepsis occurrence in the following 7 days.

**Patients and methods/Materials and methods:** Comparative retrospective monocentric study with a non-inferiority design. Patients hospitalized between 2012 and 2018 for at least 48 h in the ICU, with a bladder catheter and a positive urinary culture presenting fever (> 38 °C) or a CRP level above 100 mg/l were included. Exclusion criteria were pregnancy, kidney transplant recipients, planned urological surgery, neutropenia, bacteriemic urinary tract infection the day of inclusion, other source of infection requiring antibiotics for which the urinary bacteria was susceptible or septic shock. Based on a previous study we expected a sepsis occurrence of 10%. To provide a power of 80% with a non-inferiority margin of 10% we planned to include 112 patients in each group.

**Results:** Among 222 included patients, 100 (45%) received antimicrobial therapy and 122 (55%) did not. Groups were comparable in terms of age, sex, SAPS II score and co-morbidities. SOFA score the day of inclusion was higher in the groups who received antibiotics (median 4 versus 3 p = 0,004). Escherichia Coli (50,4%), was the most frequent bacteria identified. Amoxicillin-clavulanic acid (42%), piperacillin/tazobactam (19%) and ciprofloxacin (16%) were the most prescribed antibiotics with a median duration of 5 days. Sepsis occurrence 7 days after the positive urinary culture were similar between the two groups (treated: 7% no treatment: 1,6% p = 0,089). Moreover, we found no difference between groups in terms of mortality (6% versus 1,6% p = 0,08) or bacteremia (2% versus 2,4%p = 0,671).

**Conclusion:** Absence of antibiotic treatment for urinary catheter associated positive culture and inflammatory syndrome in ICU patients was not associated with an increase in sepsis occurrence the next 7 days following the urinary culture. Further studies are needed to better define the indication of antibiotic treatment for CAUTI in ICU setting.

**Compliance with ethics regulations:** Yes in clinical research.

### FC-129 Coagulase-negative staphylococcus in intensive care unit.

#### BENHAMZA Sabah^1,2^, LAZRAQ Mohamed^1,2^, ATAK Badreddine^1,2^, MILOUDI Youssef^1,2^, BENSAID Abdelhak^1,2^, EL HARRAR Najib^1,2^

##### ^1^Hôpital 20 Août 1953, CHUIR, Casablanca, Maroc; ^2^Université Hassan II, faculté de médecine et de pharmacie, Casablanca, Maroc

###### Correspondence: Sabah BENHAMZA (benhamzasabah5@gmail.com)

*Annals of Intensive Care* 2022, **12(1):**FC-129

**Rationale:** *Staphylococci* are one of the most incriminated pathogens in human infections (1), in recent years there has been the emergence of resistant strains, especially coagulase negative (CNS). Our objective was to evaluate the incidence of CNS in a general intensive care unit and to study their sensitivity to antibiotics.

**Patients and methods/Materials and methods:** retrospective study over 4 years conducted in the intensive care unit of the August 20 hospital. The patients included have at least one bacteriological sample positive for Staphylococcus. Demographic, epidemiological, bacteriological, therapeutic and evolutionary data were analyzed and then compared in an CNS group.

**Results:** 74 patients were included (mean age 44 ± 19.18 years, 54 men and 20 women). The incidence of CNS was 5.50%: 66% in the blood culture (n = 58) and 34% in the catheter. 78.37% of patients were admitted to the intensive care unit from the emergency reception service. The average length of hospitalization was 18.52 days ± 14.22 days. The methicillin resistance rate was 43%. On the other hand, vancomycin and teicoplanin were effective. 23 patients were treated with anti-staphylococcal antibiotic therapy, 51 did not receive it, there was no statistically significant difference between these two groups of patients in terms of mortality (p = 0.45). The most common cause of death was septic shock with no significant difference between the two treated and untreated groups.

**Conclusion:** the incidence of CNS remains low but their mortality is worrying. 43% of CNS are resistant to methicillin. Glycopeptides have always been considered effective in the treatment of staphylococcal infections. Except that in recent years we have seen the appearance of cases of resistance of CNS  to these glycopeptides.

**Reference 1:** 1- BILAL AHMAD MIR1, Dr. SRIKANTH. Prevalence and antimicrobial susceptibility of methicillin resistant staphylococcus aureus and coagulase-negative staphylococci in a tertiary care hospital. Asian J Pharm Clin Res, Vol 6, Suppl 3, 2013, 231–234.

**Compliance with ethics regulations:** Yes in clinical research.

### FC-130 Exploring blood stream infection incidence in ICU patients treated by therapeutic plasma exchange-A retrospective multicenter study

#### FODIL Sofiane^2^, MAYAUX Julien^3^, HARIRI Geoffroy^3^, LAVILLEGRAND Jean-Rémi^2^, GUIDET Bertrand^2^, MAURY Eric^2^, AZOULAY Elie^1^, DEMOULE Alexandre^3^, MARIOTTE Eric^1^, AIT-OUFELLA Hafid^2^

##### ^1^Hôpital Saint-Louis, Paris, France; ^2^Hôpital Saint-Antoine, Paris, France; ^3^Hôpital de la Pitié-Salpêtrière, Paris, France

###### Correspondence: Sofiane FODIL (fodilsofiane@gmail.com)

*Annals of Intensive Care* 2022, **12(1):**FC-130

**Rationale:** Therapeutic plasma exchange (TPE) is a technique which separates out plasma from other components of blood. The plasma is removed and replaced with a surrogate solution (1). TPE has immunosuppressive impact which may increase the risk for infectious complications (2). However, the incidence of infections following TPE in ICU patients remains poorly investigated.

**Patients and methods/Materials and methods:** We conducted a retrospective cohort study including patients treated by plasma exchange in the medical ICU of three tertiary teaching hospital in Paris, France (Saint-Antoine, Saint-Louis et Pitié-Salpétrière), between January 1st 2010 and December 31th 2019. Clinical, biological and microbiological parameters were collected with a focus on bloodstream infection (BSI).

**Results:** Overall, 264 patients were included with 5 (3–7) plasma exchange procedures. Median SOFA at admission was 5 (2–6) and ICU mortality was low (8%). The median age was 48 (34–63) years old, mainly women (59%) with a BMI at 25 (22–29). The main indications for TPE were thrombotic microangiopathy (59%), followed by CNS inflammatory diseases (14%) and hyperviscosity syndrome (7%). The majority of patients were treated by at least one immunosuppressive drug (89%). Overall, 32 patients had positive blood cultures, 9 were considered contaminated, leaving 23 BSI for analysis (9%). BSI occurred after 4 (1–8) TPE procedures, double-lumen catheter being identified as the main origin for bloodstream infection (35%). Main identified germs were *K. Pneumonia* (26%), %), *Staph. aureus* (16%), *Ps. aeruginosa* (8%), *M. morganii* (8%), *E. coli* (8%). Comparing patients with and without BSI, we found higher BMI (25 (22–28) vs 28 (23–33), P = 0.04), and more frequent catheter-related thrombosis in the infection group (5% vs 17%, P = 0.06). No significant different was observed between groups regarding comorbidities, number of immunosuppressive drugs and central catheter position (femoral or jugular).

**Conclusion:** In ICU patients treated with TPE, BSI is not frequent and can be observed within the first week of treatment.

**Reference 1:** Pham HP, Staley EM, Schwartz J. Therapeutic plasma exchange—A brief review of indications, urgency, schedule, and technical aspects. Transfus Apher Sci. 2019 Jun;58(3):237–46.

**Reference 2:** Reeves HM, Winters JL. The mechanisms of action of plasma exchange. British Journal of Haematology. 2014;164(3):342–51.

**Compliance with ethics regulations:** Yes in clinical research.

### FC-131 Endotracheal bacterial kinetic changes from intubation to onset of pneumonia during mechanical ventilation: the core of VAP pathophysiology?

#### MEYER Sylvain^1^, DANCHE Estelle^1^, GUICHARD Elie^2^, HERNANDEZ PADILLA Ana Catalina^1^, FEDOU Anne-Laure^1^, VIGNON Philippe^1^, BARRAUD Olivier^1^, FRANÇOIS Bruno^1^

##### ^1^CHU Dupuytren, Limoges, France; ^2^CHRU de Tours, Tours, France

###### Correspondence: Sylvain MEYER (sylvain.meyer@unilim.fr)

*Annals of Intensive Care* 2022, **12(1):**FC-131

**Rationale:** The presence of a tracheal tube for invasive mechanical ventilation (MV) may facilitate bacterial colonization of the lower respiratory tract (LRT) but also will change patients’ respiratory microbiota, ultimately leading to the development of Ventilator-Associated Pneumonia (VAP) directly or through a Ventilator-Associated Tracheobronchitis step in some patients. Nevertheless, even if this concept is widely accepted, little is known regarding the exact bacterial kinetic changes in LRT secretions during MV. Although the first days of intubation seem critical for microbiological evolution, current knowledge of such colonization process is limited, and descriptions are not conclusive due to important clinical heterogeneity among patient populations. The objective of the study was to exhaustively follow bacterial kinetic changes in all endotracheal secretions during the first seven days of MV starting right after intubation in a highly homogeneous and clean population.

**Patients and methods/Materials and methods:** We conducted a prospective study in adult patients requiring MV for at least 7 days. In order to have a very “clean” population, patients had to be free from acute or chronic pulmonary disease, from any acute infectious disease and from antibiotic treatment for at least 2 weeks before inclusion. Patients were included from intubation and all endotracheal secretions collected as part of routine care by bedside nurses were collected around the clock until D7. MV patients were classified in VAP or no VAP group by an independent blinded adjudication committee, according to clinical, microbiological and radiological criteria. Quantitative microbiological culture was performed from each single endotracheal aspirate (ETA) following ESCMID recommendations. Common putative pathogens were identified with the Vitek MS (bioMérieux) and quantified.

**Results:** Forty-eight patients were included, 28 (58%) were men with a mean age of 58 (± 15) years. Apache II score of 14.3 (± 5.3), and Charlson Comorbidity Index of 2.8 (± 1.9). Ten patients (21%) developed VAP. A total of 1556 ETA samples were collected (34 ETA on average per patient). *Staphylococcus aureus* and *Haemophilus influenzae* were the two most frequent pathogens identified in VAP and no VAP patients, 6 vs 20 patients and 8 vs 18 patients respectively. Mean number of common putative pathogens per patient was 2.6 (± 1.3) and 3.6 (± 1.7) in VAP and no VAP group respectively. Microbial colonization showed no similar patterns or differences in terms of CFU evolution between both groups (Figure).

**Conclusion:** Bacterial quantification from ETA samples during intubation of patients with no confounding factors showed a high intra and intervariability that is not correlated to VAP onset.

**Compliance with ethics regulations:** Yes in clinical research.
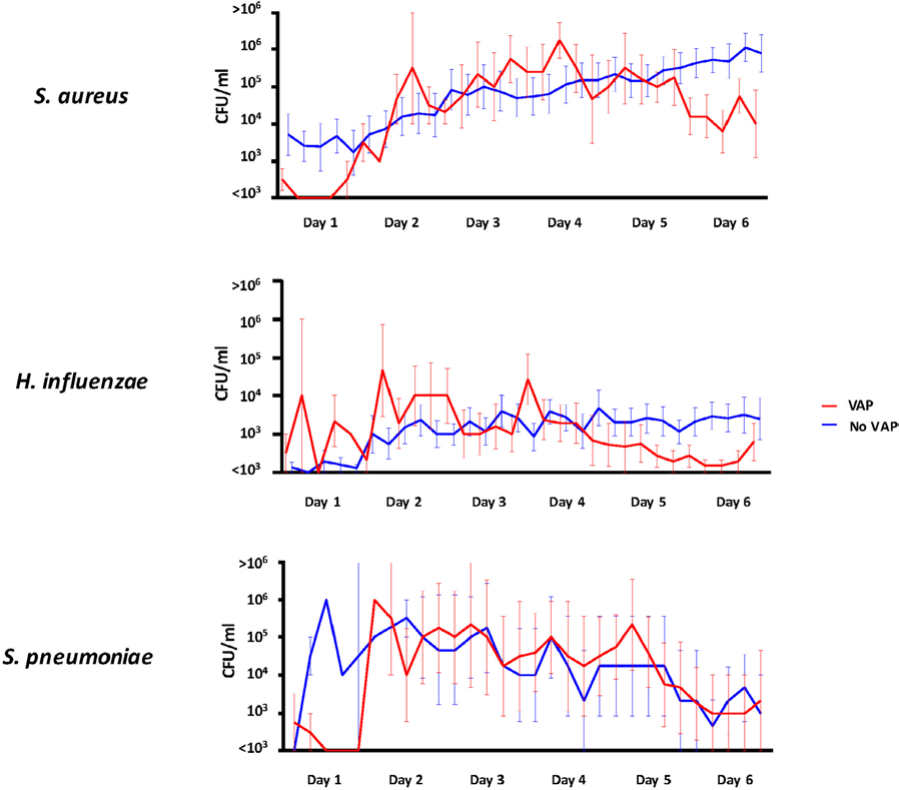


*Comparative kinetics of main putative pathogens in VAP (red) and no VAP (blue) groups expressed in mean ± standard error of mean*

### FC-132 Lung mycobiota? diversity is linked to survival in critically ill patients with hypercapnic acute exacerbation of chronic obstructive pulmonary disease

#### SIONIAC Pierre^1^, ENAUD Raphaël^1^, IMBERT Sébastien^1^, JANVIER Pierre-Laurent^1^, BUI Hoang-Nam^1^, PILLET Odile^1^, ORIEUX Arthur^1^, CAMINO Adrian^2^, BOYER Alexandre^1^, DUPIN Isabelle^2^, BERGER Patrick^2^, GRUSON Didier^1^, DELHAES Laurence^1^, PRÉVEL Renaud^1^

##### ^1^CHU Bordeaux, Bordeaux, France; ^2^Université Bordeaux, Bordeaux, France

###### Correspondence: Renaud PRÉVEL (renaud.prevel@hotmail.fr)

*Annals of Intensive Care* 2022, **12(1):**FC-132

**Rationale:** Chronic obstructive pulmonary disease (COPD) is a frequent disease affecting more than 200 million persons worldwide. The chronic course of COPD is frequently worsened by acute exacerbations (AECOPD). Mortality in patients hospitalized for hypercapnic AECOPD (the most severe) remains dramatically high and the mechanisms underlying those exacerbations are poorly understood. Lung microbiota has been demonstrated to be associated with COPD course and outcomes in non-hypercapnic AECOPD but no study specifically investigated hypercapnic AECOPD patients. The aim of this study is thus to compare lung microbiota composition between hypercapnic AECOPD survivors and non-survivors.

**Patients and methods/Materials and methods:** Induced sputum or endotracheal aspirate was collected at admission of every consecutive hypercapnic AECOPD from October 2018 to March 2019. After DNA extraction, V3–V4 and ITS2 regions were amplified by PCR. Deep-sequencing was performed on MiSeq sequencer (Illumina^®^); data were analysed using DADA2 pipeline.

**Results:** Among 47 patients admitted for hypercapnic AECOPD, 25 (53%) with samples of sufficient quality were included, 21/25 (84%) of ICU survivors and 4/25 (16%) of ICU non-survivors. AECOPD non-survivors had lower lung mycobiota (p: 0.03, p: 0.05, p: 0.05), but not bacteriobiota (p: 0.71, p: 0.543, p: 0.70), α-diversity than survivors. Both lung bacteriobiota and mycobiota were not dissimilar between survivors and non-survivors (respectively, p: 0.91 and p: 0.88). Similar results were demonstrated comparing patients receiving invasive mechanical ventilation (n: 13 (52%)) with those receiving non-invasive ventilation (n: 12 (48%). Previous systemic antimicrobial therapy and inhaled corticosteroids, but not systemic one, can alter the lung microbiota composition in hypercapnic AECOPD patients.

**Conclusion:** Non-survivors and patients requiring invasive mechanical ventilation have lower lung mycobiota, but not bacteriobiota, α-diversity than survivors and patients only receiving non-invasive ventilation respectively. This study encourages for large multicentre cohort study investigating the role of lung microbiota in hypercapnic AECOPD and urges to investigate the role of fungal kingdom of lung microbiota in hypercapnic AECOPD pathophysiology.

**Compliance with ethics regulations:** Yes in clinical research.

### FC-133 Citrulline enteral administration markedly reduces immunosuppressive plasma cell expansion in a preclinical model of sepsis

#### GAUTHIER Juliette^2^, GREGOIRE Murielle^2,3^, REIZINE Florian^1,2,3^, LESOUHAITIER Mathieu^1,2,3^, DESVOIS Yoni^2^, TARTE Karin^2,3^, TADIE Jean-Marc^1,2,3^, DELALOY Céline^2^

##### ^1^Service des Maladies Infectieuses et Réanimation Médicale, Centre Hospitalier Universitaire de Rennes, Rennes, France; ^2^UMR 1236, University of Rennes 1, INSERM, Établissement Français du Sang, LabexIGO, Rennes, France; ^3^Laboratoire Suivi Immunologique des Thérapeutiques Innovantes, Centre Hospitalier Universitaire de Rennes,, Rennes, France

###### Correspondence: Jean-Marc TADIE (jeanmarc.tadie@chu-rennes.fr)

*Annals of Intensive Care* 2022, **12(1):**FC-133

**Rationale:** The mechanisms responsible for the sustained immunosuppression induced in sepsis that account for increased susceptibility to infection remain not entirely understood. Plasmablast and plasma cell subsets whose primary function is to secrete antibodies, have emerged as important suppressive populations expanded in infection and sepsis. The natural LAG_3_ + regulatory plasma cells can suppress immunity through IL-10 production, and sepsis supports CD39^hi^ plasmablast metabolic reprogramming towards aerobic glycolysis responsible for their adenosine-mediated suppressive activity. Arginine deficiency described in sepsis has been linked to increased risk of recurrent infections. Overcoming arginine shortage by means of citrulline enteral administration was shown to efficiently improve sepsis-induced immunosuppression in the caecal ligation and puncture (CLP) model and secondary infection. Therefore, we tested here the hypothesis that citrulline administration may decrease plasmablast and plasma cell suppressive functions in sepsis.

**Patients and Methods/Materials and methods:** Splenic suppressive plasma cells have been characterized in a mouse model of sepsis. Animals underwent cecal ligation and puncture (CLP), received antibiotic therapy and fluid resuscitation. From day 0 to day 5, mice were enterally fed by citrulline (150 mg/kg/day), arginine (150 mg/kg/day) or an isonitrogenous placebo. Five days after surgery, immune dysfunctions following sepsis were studied.

**Results:** Citrulline administration markedly reduced the immunosuppressive LAG_3_ + plasma cells and the CD39^hi^ plasmablast population expanded in CLP, a mechanism that could contribute significantly to the beneficial effect of citrulline.

**Conclusion:** Our study reveals sepsis induced-immunosuppressive plasma cells as highly responsive to nutrition and further supports the development of citrulline-based clinical studies to balance the immune system in sepsis.

**Reference 1:** Beneficial effects of citrulline enteral administration on sepsis-induced T cell mitochondrial dysfunction. Proc Natl Acad Sci U S A. 2022 Feb 22;119(8).

**Compliance with ethics regulations:** Yes in animal testing.

### FC-134 Incidence and bacteriological profile of healthcare-associated infections (HAIs) in patients hospitalized for ARDS due to COVID-19

#### HAMMOUDA Zeineb^1^, BEN AHMED Hédia^1^, SAADAOUI Oussama^1^, JERBI Salma^1^, MAATOUK Iyed^1^, LAHMAR Manel^1^, DACHRAOUI Fahmi^1^, ABROUG Fekri^1^, BESBES OUANES Lamia^1^

##### ^1^CHU Fattouma Bourguiba MONASTIR, Monastir, Tunisie

###### Correspondence: Zeineb HAMMOUDA (zanoubia83@hotmail.com)

*Annals of Intensive Care* 2022, **12(1):**FC-134

**Rationale:** Healthcare-associated infections (HAIs) are associated with an increase in morbidity and mortality in patients hospitalized in intensive care. The objective of this study is to determine the incidence of HAIs and to describe their bacteriological profile in the particular context of the COVID-19 pandemic.

**Patients and methods/Materials and methods:** This is a retrospective study carried out between September 1, 2020 and September 30, 2021 in the intensive care unit of CHU FATTOUMA BOURGUIBA in MONASTIR, collecting the observations of patients admitted for ARDS due to COVID-19 whose stay was complicated by healthcare-associated infection. We analyzed the demographic, clinical and evolutionary characteristics as well as the results of the microbiological samples taken from these patients.

**Results:** 440 patients were admitted to the ICU, 79 patients developed a HAI (18%). They were 45 men with an average age of 65 years. BMR screening was done in 53 patients and was positive in 17.7% of cases. The most frequent site of infection was the pulmonary site (52%) followed by hemato-vascular (9%) and urinary (2%) infection. The responsible micro-organisms are dominated by acinetobacter (24%) followed by pseudomonas aeruginosa (3.8%) and KP ESBL (3%). Of note, 17% of IAS were polymicrobial infections and in 30% of cases the micro-organism was not identified. The median length of stay was 15 days with a median prolongation of 5 days for the occurrence of an HAI. Curative antibiotic therapy was empirical in 62% of cases based on the combination tigecycline/colimycin (63%). The most common complication was septic shock which occurred in 81% associated with multi-visceral failure in 71% of cases. The mortality rate was at 84.8%.

**Conclusion:** In this study, the incidence of IAS was significant with a substantial impact on the outcome of patients with high mortality in case of infection.

**Compliance with ethics regulations:** Yes in clinical research.

### FC-135 Healthcare-related infections in critical patients with COVID-19: epidemiology, risk factors and outcomes

#### TRIFI Ahlem^1^, MASSEOUD Lynda^1^, MEFTEH Amal^1^, SELLAOUTI Selim^1^, ABDENNEBI Cyrine^1^, ABDELLATIF Sami^1^, BEN LAKHAL Salah^1^

##### ^1^Medical ICU, la Rabta hospital, Faculty of Medicine of Tunis, Tunis, Tunisie

###### Correspondence: Ahlem TRIFI (trifiahlem2@gmail.com)

*Annals of Intensive Care* 2022, **12(1):**FC-135

**Rationale:** The speed expansion of ICU beds and the redistribution of resources during the COVID-19 pandemic have made it difficult to comply with healthcare-related infection (HCRI) prevention measures. In Tunisia, one of the countries hardest affected by this pandemic, little or no data is available on the characteristics of HCRI in the COVID-19 era. Objectives: To study the epidemiology of HCRI in patients severely affected with COVID-19, to identify risk factors and to determine their prognostic impact.

**Patients and Methods/Materials and methods:** retrospective, observational and analytical study in critical patients with COVID-19. Microbiological samples were taken if HCRI was suspected. Factors influencing the occurrence of HCRI were: demographic criteria, co-morbidities, exposure to antibiotics and steroids, invasive procedures, clinical and paraclinical characteristics. Outcome parameters were sepsis/septic shock and mortality.

**Results:** 157 patients were included, of which 60 (38.2%) presented at least one HCRI of mean age at 60 + 12 years, sex ratio = 1.3 and of SOFA at 4 + 1.7. Hypertension (n = 30) and diabetes (n = 23) were the predominant defects. 117 HCRIs were recorded corresponding to an incidence density (ID) of 69.2 IN / 1000 HD and divided into VAP (n = 38), bacteremia (n = 32), urinary tract infection (UTI, n = 24), catheter-related infection (CRI, n = 12) and fungal infection (n = 11). Compared to 2017, there was an increase of HCRIs, and the time to onset was earlier (attached figure). *Acinetobacter baumanii* (39.5%) and *Klebsiella pneumoniae* were the most isolated (27%) of which 89% had a profile sensitive only to colistin and 64.5% produced broad spectrum beta-lactamase respectively. Factors independently associated to HCRI were hyperglycemia (OR = 12.8 [3.1–51]), elevated CRP (> 115 mg/l, OR = 1.009 [1.001–1.017]), steroid use duration > 10 d (OR = 1.25 [1.08–1.44]), arterial catheter (OR = 6.57 [1.35–31]) and bladder catheterization (OR = 43 [4–466]). Patients developing HCRI presented more septic origin (91% vs 72%, p < 10–3). HCRI was an independent factor of mortality which multiplied it by 8 (OR = 8.49, p = 0.004). Other factors were also independently linked to mortality: mechanical ventilation (OR = 23.5, p = 10–3) and stage 3 ARDS (OR = 7.22, p = 0.007).

**Conclusion:** During the COVID period, the rate of HCRIs increased mainly VAP and UTI. Multi-resistant *Acinetobacter baumanii* and *Klebsiella pneumoniae* remained the most isolated pathogens. In addition to the common risk factors often reported even outside of COVID, hyperglycemia, elevated CRP, and prolonged corticosteroid duration were the original factors found associated with HCRIs in our cohort. When the HCRI grafted onto the evolutionary process of those critically ill with COVID, it amplified the risk of death by eight.

**Reference 1:** Trifi A, Abdellatif S, Oueslati M, Zribi M, Daly F, Nasri R, Mannai R, Fandri C, Ben Lakhal S. Nosocomial infections: current situation in a resuscitation-unit. Tunis Med. 2017 Mar;95(3):179–184. PMID: 29446811.

**Compliance with ethics regulations:** N/A.
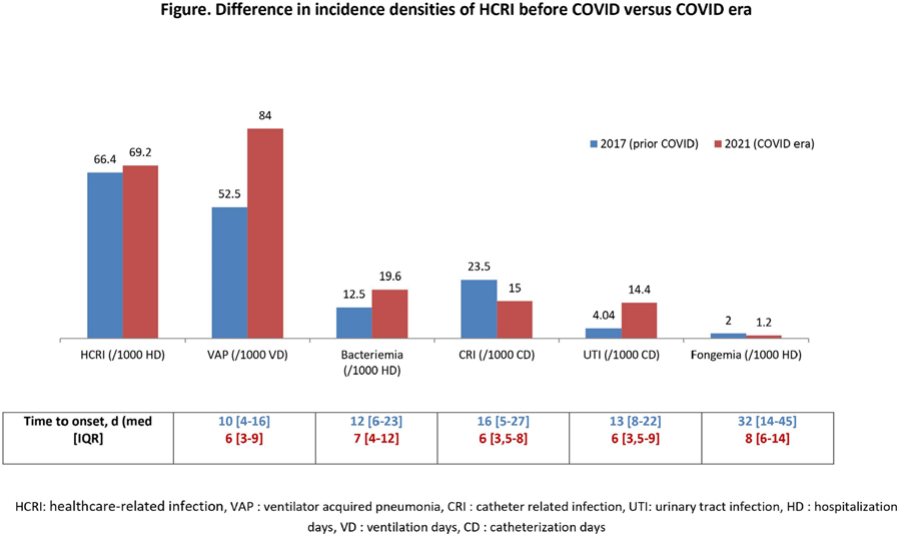


*Figure. Difference in incidence densities of HCRI before COVID versus COVID era.*

### FC-136 Prevalence and outcome of bacteremia among Covid 19 critically ill patients

#### ALILA Ilef^1^, KHARRAT Sana^1^, HADDED Amina^1^, CHTARA Kamilia^1^, BAHLOUL Mabrouk^1^, BOUAZIZ Mounir^1^

##### ^1^hopital Habib Bourguiba Sfax, Sfax, Tunisie

###### Correspondence: Ilef ALILA (ilefalila1323@gmail.com)

*Annals of Intensive Care* 2022, **12(1):**FC-136

**Rationale:** The COVID-19 epidemic has been a health threat worldwide causing multi-organ damage. This viral infection (COVID-19) is not the only cause of deaths in this pandemic. A usual complication of viral infections is a secondary bacterial infection or a superinfection (1). The aim of this study was to assess prevalence, characteristics and outcome of bacteremia in critically ill COVID-19 patients.

**Patients and methods/Materials and methods:** We conducted a retrospective study in an ICU over a period of 16 months [September 2020–December 2021] including patients with SARS-COV2 infection.

**Results:** During the study period, 586 patients were included with a mean age of 59.5 ± 14.7 and a gender ratio of 1.6. The median SAPSII and SOFA score were respectively of 29 ± 14.8 and 4 ± 2.6. Most patients had comorbidities, including hypertension (36%), obesity (32.1%) and diabetes (36.2%). Bacteremia was diagnosed in 42 (7.2%) patients after a mean hospital stay of 7.4 ± 4.5 day. The most frequently found germs were: *Klebsiella pneumoniae* in 10 (23.8%) patients, *Enterococcus faecalis* in 7 (16.7%) patients and *Acinetobacter baumanii* in 6 (14.3%). Those with bacteremia had significantly higher rates of septic shock (71.4% vs 41.2%; p < 0.001. Bacteremia was associated with longer length of stay. In addition, the use of invasive mechanical ventilation and mortality were significantly higher in bacteremia group (Table I).

**Conclusion:** Bacteremia is a severe complication in patients suffering SARS-COV2 infection and requiring ICU admission. The development of Bacteremia is associated with poor outcome.

**Reference 1:** (1)Nag V.L. · Kaur N: Superinfections in COVID-19 Patients: Role of Antimicrobials.

**Compliance with ethics regulations:** Yes in clinical research.
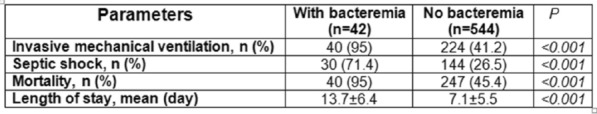


Table I: Outcome comparison according to bacteremia

### FC-137 Impact of closed tracheal suction systems on bacterial respiratory samples in patients with SARS-CoV2 infection: a monocentric study

#### MURGAT Pierre-Henri^1^, CARRICAJO Anne^1^, THIERY Guillaume^1^, PERINEL-RAGEY Sophie^1^

##### ^1^CHU de Saint Etienne, Saint-Priest-En-Jarez, France

###### Correspondence: Pierre-Henri MURGAT (murgat.ph@gmail.com)

*Annals of Intensive Care* 2022, **12(1):**FC-137

**Rationale:** During the COVID-19 pandemic, use of closed tracheal suction systems (CTSS) has become widespread in intensive care units (ICU) to reduce health care workers and environmental contaminations. These systems are frequently mobilized to aspirate the tracheo-bronchial secretions. Furthermore, they may be used to perform bacteriological samples by tracheal aspirations in case for example of suspected pneumonia. CTSS are set up for 3 days according to manufacturer and due to pandemic shortage conditions sometimes longer in several ICU. The aim of this study is to assess the influence of CTSS use on respiratory bacteriological samples.

**Patients and methods/Materials and methods:** This prospective observational monocentric study realized in an ICU of University Hospital enrolled patients between April 2021 and February 2022 after ethical committee approval. Inclusion criteria were: active SARS-Cov-2 infection, CTSS use under invasive mechanical ventilation and need to change this system according to local practice recommendations. At time of CTSS replacement, we performed a microbiological analysis of the tracheal secretions before the removal. A second sample of tracheal secretions was taken through the new CTSS at first need of aspiration for the patient. Finally, a microbiological analysis was also carried out on the distal part of the CTSS removed.

**Results:** 15 patients were enrolled, 3 were excluded for missing data. The mean duration of CTSS use was 6,3 days (± 4.8) Of the 12 samples carried out on previous CTSS, 6 were sterile (50%). For these 6 patients, bacterial samples made through new CTSS were also sterile. CTSS culture was also sterile with the exception of two patients. The other 6 tracheal secretions made on previous CTSS have found bacteria classically implicated in ventilation acquired pneumonia (VAP) (1). There was no qualitative bacteriological difference between these 6 samples and the tracheal secretions made on the new CTSS. Cultures of the CTSS distal part found the same bacteria with one exception.

**Discussion:** We found a good agreement between bacteriological samples performed on previous CTSS and samples made through new CTSS. Consequently, no contamination of the device in place for several days was observed. This result is of importance as higher risk of VAP and of multi drug resistant bacteria was observed for SARS-CoV-2 ICU patients (1). Yet, these data do not inform on impact of CTSS for respiratory tract bacterial colonization and further studies are mandatory.

**Conclusion:** The study suggests that the realization of tracheal secretions through CTSS does not lead to bacterial contamination of the respiratory samples.

**Reference 1:** (1) “Risks of ventilator-associated pneumonia and invasive pulmonary aspergillosis in patients with viral acute respiratory distress syndrome related or not to Coronavirus 19 disease”, K Razazi and al; Crit Care. 2020 Dec 18;24(1):699. https://doi.org/10.1186/s13054

**Compliance with ethics regulations:** Yes in clinical research.

### FC-138 Healthcare-associated infections in critical COVID-19 forms

#### GUISSOUMA Jihene^1,2^, BEN ALI Hana^2^, TRABELSI Insaf^1,2^, ALLOUCHE Hend^1,2^, BRAHMI Habib^1,2^, SAMET Mohamed^2^, GHADHOUNE Hatem^1,2^

##### ^1^Faculté de médecine de Tunis, Bizerte, Tunisie; ^2^Université Tunis El Manar, Tunis, Tunisie

###### Correspondence: Jihene GUISSOUMA (guissouma.jihene@gmail.com)

*Annals of Intensive Care* 2022, **12(1):**FC-138

**Rationale:** Healthcare-associated infections (HCAI) have been well described in previous viral epidemics but reports about secondary infections associated to COVID-19 are limited. We aimed to discern the risk factors of HCAI in patients admitted to intensive care unit (ICU) for critical COVID-19 forms and their prognostic impact.

**Patients and methods/Materials and methods:** A 16-month (September 2020–December 2021) prospective descriptive study including all patients admitted to ICU for critical COVID-19 forms. The patients were divided into two groups: -Group1: patients who developed HCAI during ICU stay. -Group2: patients who didn’t develop HCAI.

The statistical analysis was performed using SPSS 23.

**Results:** Overall 140 patients were included. The mean age was 58 ± 13 with male predominance. The most common comorbidities were hypertension (64%) and diabetes (62%). Four patients had auto immune disease history with immunosuppressor ongoing treatment. The mean IGS II and APACH II were 40 ± 13 and 13 ± 6 respectively.

Fifty-nine bacterial HCAI were reported in 55 patients (group1 and group2 counted 55 and 85 patients respectively). Healthcare-associated pneumonia was the most common one (44 cases equal 74% of HCAI) followed by bacteremia (6 cases), urosepsis (3 cases), infection related to a central-line catheter (2 cases), infection of bedsores (2 cases) and colitis (2 cases). Only 24 among the 59 HCAI reported were documented. The most common microorganisms isolated were: *Acinetobacter baumannii* (8 cases), *Pseudomonas aeruginosa* (4 cases) and *Stenotrophomonas maltophilia* (2 cases). Besides, fungal infection was considered in 15 cases but only 7 were confirmed by cultures which isolates *Candida albicans* in 4 cases and *non albican*s in 3 cases. In the remaining cases, the diagnosis was based on candida score, clinical and biological criteria without a microbiological laboratory confirmation. The mean length of ICU stay was significantly higher (p < 10^–3^) in group1 (17 days) compared to group2 (8 days).

Overall mortality was 69%. It was higher in group 1 (85%) compared to group 2 (60%) with a statically significant difference (p = 0,01). In univariate analysis, auto immune disease history (p = 0,03), prior antibiotherapy (p = 0,05), septic shock on admission (p < 10^–3^) and invasive ventilation (p < 10^–3^) were the main risk factors for acquiring HCAI. Only septic shock was an independent risk factor in multivariate analysis (p = 0,05).

**Conclusion:** The occurrence of HCAI in critical COVID-19 forms was frequent in our study. It was associated to a poorer outcome and a longer ICU stay. Septic shock on admission was the only independent risk factor for acquiring HCAI. These results need to be confirmed by further larger studies.

**Compliance with ethics regulations:** Yes in clinical research.

### FC-139 The Impact of hospital-acquired infections on critically ill COVID-19 patients’ outcome

#### GHOZZI Mohamed Khalil^1^, JAMOUSSI Amira^1^, SDIRI Ines^1^, AYED Samia^1^, RACHDI Emna^1^, JARRAYA Fatma^1^, BESBES Mohamed^1^, BEN KHELIL Jalila^1^

##### ^1^Hôpital Abderrahmen Mami, Ariana, Tunisie

###### Correspondence: Mohamed Khalil GHOZZI (medkhalilghozzi@gmail.com)

*Annals of Intensive Care* 2022, **12(1):**FC-139

**Rationale:** Hospital-acquired infections (HAI) have been constantly reported in critically ill patients with COVID-19. The aim of the present study was to assess characteristics and outcome of critically ill COVID-19 patients whom ICU stay was complicated with HAI.

**Patients and methods/Materials and methods:** We conducted a retrospective single-center, case–control study including patients with COVID-19 infection admitted to the ICU between March 2020 and September 2021. We evaluated clinical features and outcome of critically ill COVID-19 patients with HAI.

**Results:** During the study period, 544 patients were collected. The mean age was 59 ± 13 years [18–95] with a gender-ratio (H/F) of 1.66. The mean SAPS II and APACHEII scores were 28 ± 10 and 10.3 ± 5, respectively. Invasive mechanical ventilation was required in 57.2% of patients. Two hundred thirty-seven (43.5%) patients developed a HAI. The most common HAI was pneumoniae (28.3%), followed by urinary tract infection (13.2%), bacteremia (11%) and central line-associated bloodstream infection (4.6%). Comparison of clinical and outcome findings between HAI and non-HAI patients is reported in Table 1.

**Conclusion:** HAIs are frequent among critically ill COVID-19 patients. They substantially worsen patients’ outcome and significantly extend ICU length of stay. As it has always been in ICU, HAIs management and prevention represents a serious challenge to healthcare professionals globally during the COVID-19 pandemic.

**Compliance with ethics regulations:** Yes in clinical research.
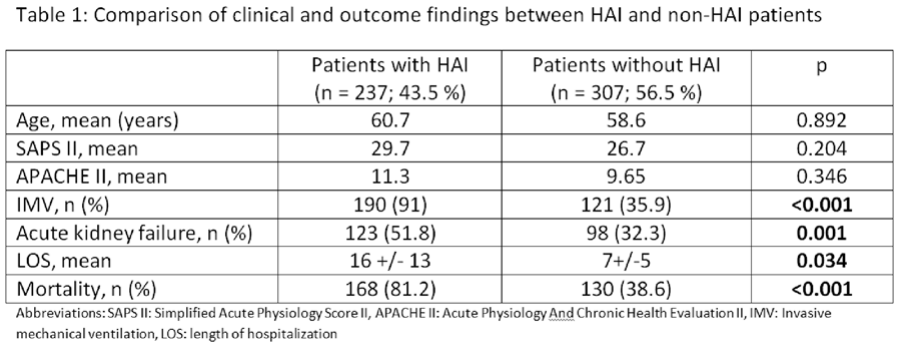


*Table 1: Comparison of clinical and outcome findings between HAI and non-HAI patients.*

### FC-140 Nosocomial infection profile in critically-ill covid-19 patients

#### SDIRI Ines^1,2^, RACHDI Emna^1,2^, GHOZZI Mohamed Khalil^1,2^, JAMOUSSI Amira^1,2^, JARRAYA Fatma^1,2^, BESBES Mohamed^1,2^, AYED Samia^1,2^, BEN KHELIL Jalila^1,2^

##### ^1^Hopital Abderrahman Mami, Ariana, Tunisie; ^2^Faculté de médecine de Tunis, Tunis, Tunisie

###### Correspondence: Emna RACHDI (e.rachdi@yahoo.fr)

*Annals of Intensive Care* 2022, **12(1):**FC-140

**Rationale:** Nosocomial bacterial and fungal infections (NI) have been reported in patients with COVID-19, resulting from an immunosuppressed state. However, few data are available on these infections in critically ill patients. The objective of this study was to evaluate the characteristics of ICU-acquired infections in COVID-19 patients and to establish the microbiological profile.

**Patients and methods/Materials and methods:** It was a retrospective descriptive study including adults with COVID-19 admitted in ICU of Abderahmen Memi Hospital, from March 2020 to December 2021. Demographic, clinical, microbiological and therapeutic data were collected. We evaluated the epidemiological and microbiological features of NI.

**Results:** During the study period, 544 patients were collected. The median age was 59 years [18–95] with a gender ratio of 1.66. Mean SAPS II and APACHEII scores were 27.9 ± 10 and 10.3 ± 5, respectively. The most common histories were hypertension (39%) and diabetes (36.6%) with a mean BMI of 30.79 kg/m^2^. All patients received dexamethasone and 44% had antibiotic therapy on admission. Four hundred fifty-one patient (82.9%) required noninvasive ventilation and 311 (57.2%) needed invasive ventilation with a median delay of 3 days [0–24 d] from admission. NI complicated 237 patients (43.5%) with a total of 333 episodes (90 patient had 1 episode, 33 had 2, 20 had 3 and 23 had more than 3 episodes), 9% of them were acquired in other departments and discovered at admission. Pneumopathy acquired under mechanical ventilation was the major NI and represented 81%. Bacteria isolated in these cases were non-fermenting germs (65%) (*Pseudomonas aeruginosa* and *Acinetobacter baumanii* imipenem resistant) and Enterobacteriaceae (47%), in particular Klebsiella pneumoniae BLSE. Urinary infection (13.2% of IN) was dominated by Enterococci in 27 cases (among them 2 cases of ERV), *Escherichia coli* in 12 and *Klebsiella pneumoniae* in 4 (1 was ESBL). Bacteremia and catheter-related bloodstream infections were observed in 11% and 4.6% of cases respectively. Four percent of NI was fungal infection and the most identified germ was *Candida albicans*. NI was associated with septic shock in 74.5% of cases. Mortality rate was 54.8% due to septic shock with multiple organ failure in 33% of cases.

**Conclusion:** Bacterial and fungal nosocomial infection is a common complication seen in ICU patients, more observed and repeated in COVID-19 patients. It is often a severe form of infection, dominated by Gram-negative resistant bacteria acquired during ICU stay and associated with a poor prognosis.

**Compliance with ethics regulations:** Yes in clinical research.

### FC-141 Delivery of high flow oxygen through nasal versus tracheal canula. A bench study

#### GUÉRIN Claude^1,2^, COUR Martin^1^, DEGIVRY Florian^1^, ARGAUD Laurent^1^, LOUI Bruno^2^

##### ^1^Hôpital Edouard Herriot Hospices Civils de Lyon, Lyon, France, France; ^2^Institut Mondor de Recherches Biomédicales, Créteil, France

###### Correspondence: Claude GUÉRIN (claude.guerin@chu-lyon.fr)

*Annals of Intensive Care* 2022, **12(1):**FC-141

**Rationale:** High flow oxygen therapy (HFOT) has increasingly been used in the Intensive Care Units, in particular during the SARS-Cov2 pandemic. Furthermore, the kind of devices to perform HFOT has also increased with dedicated devices and ICU ventilators equipped with this function. Finally, HFOT can be delivered through nasal or tracheal canula. This bench study was motivated by the comparison of the work of breathing (WOB) across HFOT devices and canula.

**Patients and methods/Materials and methods:** Seven devices were tested: the original device (Optiflow), two HFOT dedicated devices (Airvo2 and HM80) and 4 ICU ventilators offering this function (G5, T60, V500 and V60). Each device was connected to a lung model (ASL 5000) set with compliance 40 ml/cmH_2_O, resistance 10 cmH_2_O/L/s at a simulated inspiratory effort of – 10 cmH_2_O delivered at 30 breaths/min. Nasal (Optiflow 3S medium size, Fisher and Paykel) and tracheal (OPT 970, Fisher and Paykel) canula were attached to a specific manikin head and connected to the HFOT device on one hand and to the lung model on the other hand. Each device was tested in each canula at a 40 L/min oxygen flow delivered at ambient temperature (heated humidifier set in the circuit but off) and FIO_2_ 0.21 for 2 min. The data were recorded with ASL software and analyzed via an application developed in the Matlab environment. On each breath the WOB was measured by using the Campbell diagram, then multiplied by the respiratory rate and expressed as J/min. The effects of canula and device were tested by two-factor ANOVA. The values are expressed in median (1–3 quartiles) for the reference device (Optiflow) and in relative variation from it for each device.

**Results:** With the Optiflow reference device the WOB was 3.7 (3.7–3.8) and 3.46 (3.5–3.5)  J/min with the nasal and the tracheal canula, respectively (P < 0.05). With the nasal canula the relative variations from the reference device were 4.6, 6.2, 2.4, 4.5, and 2.2% for Airvo2, G5, HM80, T60, V500 and V60, respectively (P < 0.05 vs the reference for each device). For the tracheal canula the corresponding values of the devices were 1.2, 1.5, 2.6, 0.6, 1.2 and 2.6%, respectively (P < 0.05 for each device). The relative variations were significantly different between nasal and tracheal canula as was the interaction between canula and devices.

**Conclusion:** The WOB was lower with tracheal than with nasal canula for every device. The differences between devices may not be clinically relevant.

**Compliance with ethics regulations:** N/A.

### FC-142 Evaluation of the new heated wire humidifier FP950 under varying ambient temperature conditions

#### BOUCHARD Pierre-Alexandre^1^, ROUSSEAU Emilie^1^, LELLOUCHE François^1^

##### ^1^Institut Universitaire de Cardiologie et de Pneumologie de Québec, Québec City, Canada

###### Correspondence: François LELLOUCHE (francois.lellouche@criucpq.ulaval.ca)

*Annals of Intensive Care* 2022, **12(1):**FC-142

**Rationale:** It has been previously shown that heated wire humidifiers performances were influenced by ambient air temperature. When ambient temperature is high, heater plate temperature decreases to maintain chamber temperature stable at 37 °C. Consequently, the humidification performances are significantly reduced, well below 30 mgH_2_O/L of absolute humidity with risk of endo-tracheal tube occlusion. These performances are partially improved with specific settings (increased chamber temperature to 40 °C or activation of compensation algorithm) (1). The aim of the study was to evaluate the new generation heated wire humidifier FP950 that adds parameters in its algorithm (including ambient temperature) to maintain a stable humidity delivered to patients.

**Patients and methods/Materials and methods:** We measured on bench the hygrometry of inspiratory gases delivered by (i) FP950 (Fisher&Paykel Healthcare, Auckland, New Zealand) (ii) MR850 with usual settings (37 at the chamber/40 at the Ypiece) (iii) MR 850 with no temperature gradient (40/40), and (iv) MR850 with compensation algorithm activated. Hygrometry was measured with the psychrometric method (1) after at least 1 h of stability while varying the room temperature from 20 to 30 °C. In another set of experiments, we continuously measured ambient temperature in several ICU rooms (with different exposure), at our institution with (EL (EasyLog) -USB-1 temperature data logger, Lascar Electronics) during several months. One measure every 30 min is recorded by this device.

**Results:** We performed 178 hygrometric measurements at steady state for the different conditions tested. The main results are shown in the figure. With the new heated wire heated humidifier MR950, the mean humidity delivered remained stable at 35.4 ± 1.3 mgH_2_O/L of delivered absolute humidity, despite variations of the ambient temperature from 19.9 to 30.3 °C. Minimum values of absolute humidity were 31.1 mgH_2_O/L with FP950, 19.8, 26.6 and 25.5 with MR850 set at 37/40, 40/40 and with activated compensation respectively. With ambient temperature above 27.5 °C, absolute humidity was 34.2 mgH_2_O/L, compared to 23.1 mgH_2_O/L with MR850 (37/40), P < 0.001, 31.6 mgH2O/L with MR850 (40/40), p = 0.004, and 29.3 mgH_2_O/L with MR850 and compensation algorithm activated (P < 0.001). The continuous measurement of the ambient temperature in ICU rooms showed that the temperature tested in the bench study were relevant to the clinical practice in Quebec City with temperatures varying from 20 to 29 °C.

**Conclusion:** The new heated wire heated humidifier FP950 demonstrated stable performances while varying ambient temperature from 20 to 30 °C. The humidification performances were better than did previous generation of heated humidifier when ambient temperatures were high.

**Reference 1:** Lellouche L, Taillé S, Maggiore SM, Qader S, L'Her E, Deye N, et al. Influence of ambient air and ventilator output temperature on performances of heated-wire humidifiers. Am J Respir Crit Care Med 2004;170:1073–1079.

**Compliance with ethics regulations:** N/A.
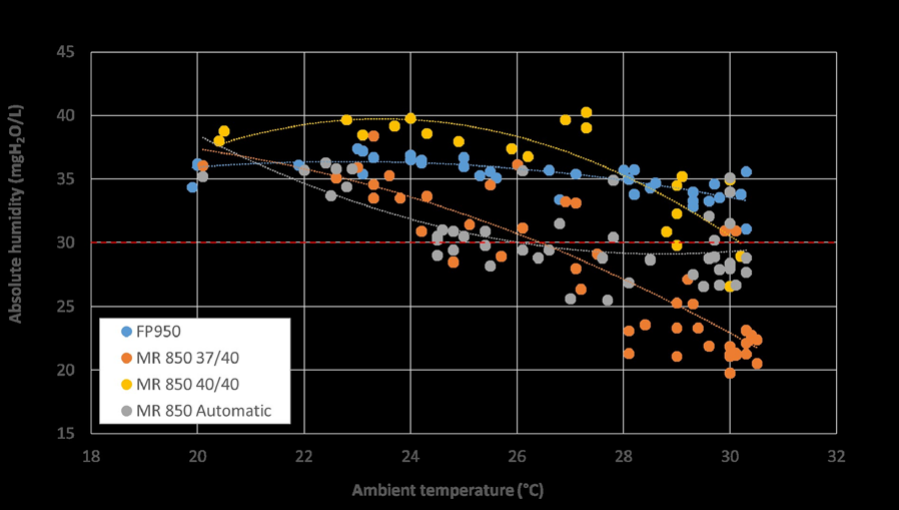


*Absolute Humidity at different ambient temperatures delivered by heated humidifiers FP950 and MR 850 (at different settings: 37/40, 40/40 and with compensation activated). The value of 30 mgH*_*2*_ *O/L is indicated with red dotted line*

### FC-143 Monitoring of heated wire humidifier hygrometric performances with heater plate temperature

#### LELLOUCHE François^1^, SIMARD Serge^1^, BOUCHARD Pierre-Alexandre^1^

##### ^1^Institut Universitaire de Cardiologie et de Pneumologie de Québec, Québec City, Canada

###### Correspondence: François LELLOUCHE (francois.lellouche@criucpq.ulaval.ca)

*Annals of Intensive Care* 2022, **12(1):**FC-143

**Rationale:** Under-humidification and associated complications may occur with heated humidifiers. Endo-tracheal tube occlusions and sub-occlusions have been shown even with these devices. Hygrometric performances of heated wire humidifiers are reduced by high ambient and high outlet ventilator temperatures. Currently, there is no reliable monitoring tool to evaluate humidification performances of heated wire humidifiers in the daily practice. The objectives of this study were to demonstrate the relation between humidity delivered by heated wire humidifiers and different parameters that could be used to monitor humidity of gas delivered to patients.

**Patients and methods/Materials and methods:** On a bench test, we measured heater plate temperature, inlet chamber temperature and delivered humidity with MR850 system (Fisher & Paykel). Temperature displayed on the humidifier was also recorded. The measurements were performed at different ambient temperatures and 5-min ventilation levels (5, 7.5, 10, 12.5, 15 l/min) set on an ICU ventilator. Inlet chamber temperatures varied from 20 to 40 °C. In each condition, hygrometric measurements with the psychrometric method were performed at steady state. We evaluated the relationship between heater plate temperature and humidity delivered and the factors that may alter this relationship.

**Results:** We performed 279 hygrometric measurements at steady state including all conditions and concomitant heater plate, ambient and chamber temperature were recorded. We found a good correlation between heater plate temperature and absolute humidity delivered (R2 = 0.82). This relationship was hardly affected by ambient temperature, but minute ventilation had more effect (Figure). For different minute ventilations, the correlation between heater plate temperature and absolute humidity delivered were very good with coefficient of determination R2 from 0.87 to 0.98 (Figure). Heater plate temperature above 62 °C was a very good predictor of absolute humidity delivered above 30 mgH2O/L (AUC = 0.96, sensibility 79%, specificity 94%). No correlation existed between humidity delivered and the outlet chamber temperature (displayed on the humidifier).

**Conclusion:** In this bench study, we have shown a good correlation between heater plate temperature and humidity delivered with a heated wire humidifier. This parameter could be used as a surrogate of humidity to improve the humidification monitoring.

**Compliance with ethics regulations:** N/A.
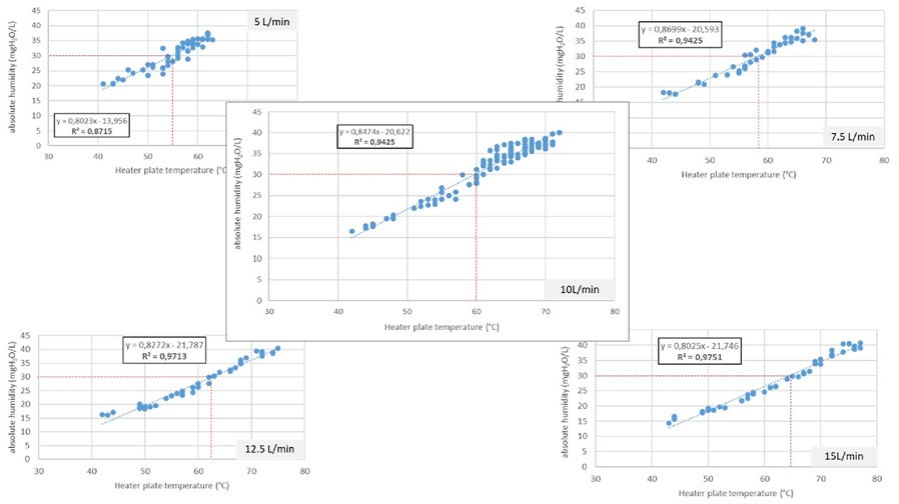


*Relationship between absolute humidity (mgH2O/L) delivered at the Y piece and heater plate temperature (°C) for different minute ventilation (from 5 to 15 L/min). The blue dotted lines represent the regression lines, the equations and the coefficient of d.*

### FC-144 Hygrometric performances of Heat and Moister Exchangers with low dead space

#### BOUCHARD Pierre-Alexandre^1^, ROUSSEAU Emilie^1^, BRANSON Richard^2^, LELLOUCHE Francois^1^

##### ^1^Institut Universitaire de Cardiologie et de Pneumologie de Québec, Québec City, Canada; ^2^Department of Surgery, Division of Trauma & Critical Care, University Cincinnati, Cincinnati, Etats-Unis

###### Correspondence: Francois LELLOUCHE (francois.lellouche@criucpq.ulaval.ca)

*Annals of Intensive Care* 2022, **12(1):**FC-144

**Rationale:** Providing gas humidification greater than 28 mgH_2_O/L is mandatory for patients with prolonged intubation, with either heat and moisture exchangers (HME) or heated humidifiers (HH). In patients requiring lung protective ventilation, HH is the first line humidification device to minimize dead space. When transporting these patients, it is important to use HMEs with low dead space and sufficient humidity delivered. In this study, we have evaluated hygrometric performances of 7 devices (4 HMEs and 3 filters) with low deadspace.

**Patients and methods/Materials and methods:** We tested on a bench, the hygrometric performances of four HMEs: BACT HME 6310 (47 ml), Pharma system—DYNJAAHME10 (36 ml), Medline—HUMID VENT 2S (28 ml), Teleflex Medical—DAR HME small (51 ml), Medtronic and three Filters: HEPASHIELD (47 ml), Flexicare—AQUASURE (32 ml), Bomimed—DAR filter (36 ml), Medtronic. We have used psychrometric method to measure hygrometry at steady state, with expiratory humidity of 35 mgH_2_O/L simulated as previously described. For each condition, 3 hygrometric measurements were performed with similar conditions for ambient air temperature 25 ± 0.5 °C, and ventilator settings (respiratory rate 20/min, tidal volume 500 ml, FiO_2_ 21%, PEEP 5 cmH2O, square flow 60 l/min). We compared data obtained on bench with data provided by manufacturers.

**Results:** We performed 3 hygrometric measures for each tested device and compared to the data provided by the manufacturers. Large differences were shown between HME (from 29.2 to 25.4 mgH_2_O/L) and Filters (18.2 to 10.4 mgH_2_O/L). Large differences existed between humidity measured by the psychrometric method (our bench) and the data provided by the manufacturers (available for 6 devices). The main results are presented on the figure.

**Conclusion:** Several HMEs with small dead space provide sufficient gas humidity and may be used during transport for patients with lung protective ventilation. However, important differences were found in terms of humidification performances for small dead space HMEs and filters. For prolonged utilization, humidity delivered should be above 28 mgH2O/L. For short-term utilization (few hours), it may be acceptable to use HMEs with humidity delivered above 25 mgH2O/L, but filters without sufficient humidity must not be used. Data provided by manufacturers obtained with gravimetric method may not be reliable.

**Compliance with ethics regulations:** N/A.
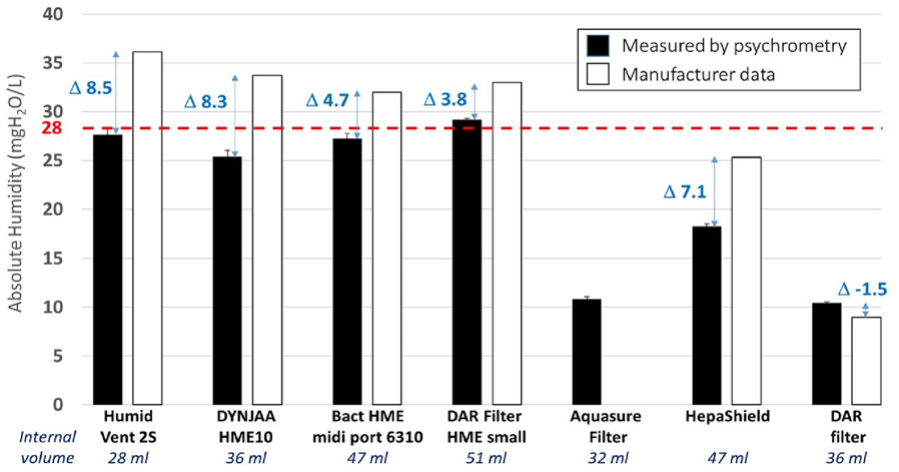


*Absolute Humidity according to manufacturer’s (white bars) and to humidity measurements with the psychrometric method (black bars) with small dead space HMEs (Humid Vent 2S, DYNJAHME10, Bact HME midi port 6310, DAR filter HME small) and small filters (Aqu.*

### FC-145 Aerosoltherapy and mechanical ventilation: effect of the position and orientation of an inhalation chamber into an adult ventilation circuit on in vitro drug delivery

#### ECKES Myriam^1^, HERVIEU Brenda^1^, BEQUET Quentin^1^, PORÉE Thierry^1^

##### ^1^Laboratoire optimHal, Valognes, France

###### Correspondence: Myriam ECKES (meckes@ohps-lab.com)

*Annals of Intensive Care* 2022, **12(1):**FC-145

**Rationale:** Positioning the pressurized metered dose inhaler (pMDI) device on the inspiratory branch of a mechanical ventilation circuit is safer especially with a high internal volume spacer. Indeed, the volume of the device is added to the dead space. However, inserting the devices between the Y piece and the endotracheal tube could be convenient especially when using tubing bonded with Y piece. Tubing dimensions are not the same depending on the position of the device on the circuit. inhaler devices conceived to be inserted into the inspiratory branch of an adult MV circuit can only be used oriented at the opposite as indicated in instructions for use (IFU) when devices are inserted between the Y piece and the endotracheal tube (ETT). Otherwise, adapters are needed. This study aimed to evaluate the effect of position of two different inhaler devices and orientation of pMDI nozzle on drug delivery.

**Patients and methods/Materials and methods:** A ventilator was used in volume-controlled mode with adult respiratory parameters. The inhaler devices, CombiHaler^®^ (Laboratoire OptimHal) and a T adapter (Intersurgical) were inserted into two different positions: 1) in the inspiratory limb with the pMDI nozzle oriented to the patient and 2) between the ETT and the Y piece with the orientation reversed. Ten doses containing 100 µg of salbutamol were actuated in the prototypes during inspiration. The delivered dose was collected on a filter inserted between the ETT and the test Lung.

**Results:** Results showed an equivalent drug delivery when CombiHaler^®^ is inserted in the inspiratory branch and between the ETT and the Y piece with orientation reversed. The drug dose delivered is higher when using the T adapter in the inspiratory branch in comparison with after the Y piece with the orientation reversed. The results could be explained by the high volume of the spacer which could keep the drug particles into the spacer and avoid impaction into the Y piece and tubings.

**Conclusion:** Using the spacer in the reverse orientation doesn’t seem to affect drug delivery while orientation of the aerosol spray with the T adapter has an impact on drug delivery.

**Compliance with ethics regulations:** N/A.

### FC-146 Comparison of aerosol deposition in the respiratory tract under spontaneous and non-invasive ventilation

#### BONSIGNORE Jordan^1^, LARA Leclerc^2^, POURCHEZ Jérémie^2^, PREVOT Nathalie^1^, PERINEL-RAGEY Sophie^1^

##### ^1^CHU SAINT-ETIENNE, Saint-Priest-en-Jarez, France; ^2^MINES SAINT-ETIENNE, Saint-Etienne, France

###### Correspondence: Jordan BONSIGNORE (jord.bonsignore@gmail.com)

*Annals of Intensive Care* 2022, **12(1):**FC-146

**Rationale:** Nebulization is a common route of administration for medications in ICU. For example, aerosols are fundamentals in association with non-invasive ventilation (NIV) for an exacerbations of chronic obstructive pulmonary disease (COPD). Nevertheless, nebulization practices are numerous, frequently led only by data from in vitro studies. To improve our practices, the AIM of this study is to evaluate the aerosol deposition when aerosols are realized during NIV or spontaneous breathing in an ex vivo preclinical model.

**Patients and methods/Materials and methods:** The ex vivo respiratory model, was previously validated (1), composed of a porcine respiratory tract (RT), placed in a sealed enclosure. Spontaneous breathing was performed through a head replica with facial mask or a NIV mask according to the condition. During NIV we used a medical ventilator set in spontaneous breathing with pressure support; for the face mask an additional gas flow of 1 L/min or 6 L/min was used according to manufacturer recommendations. The vibrating mesh nebulizer was filed with 99m-Technetium-labeled diethylene-triamine-penta-acetic acid. We performed a gamma camera acquisition to quantify the deposited fractions in each part of the setting.

**Results:** The model was setup 3 times for each condition. The mean deposited fractions in each component lead to the Fig. 1. For NIV conditions, (PART A), they reached respectively (results express in % of nominal dose): 17% (± 6%) in RT, 3% (± 2%) in face mask, 26% (± 8%) in head replica, 14% (± 1%) in inspiratory limb and 22% (± 2%) in expiratory limb and pump filter with 18% (± 2%) of leaks. For face mask conditions with gas flow of 1 L/min then 6 L/min (PART B), they reached: 36% (± 8%) and 25% (± 3%) in RT, 36% (± 8%) and 20% (± 1%) in face mask, 21% (± 3%) and 21% (± 2%) in head replica with 5% (± 2%) and 21% (± 0%) leaks.

**Discussion:** With vibrating mesh nebulizer, aerosols performed with face mask and smallest gas flow increased the deposited fractions in RT from 17% under NIV to 25% at 6 L/min 36% at 1 L/min. These results are in accordance with França et al. (2) who showed a deposited fraction reduced of 50% in healthy volunteers when performing aerosols during NIV.

**Conclusion:** The ventilation mode and interface impact the aerosol deposition, other conditions being reproductive. The optimal condition using a facemask with gas flow of 1 L/min must be taken in consideration by clinician to optimize nebulization practices when it is safe for patients.

**Reference 1:** Montigaud Y, Georges Q, Pourchez J, Leclerc L, Goy C, Clotagatide A, et al. Aerosol delivery during invasive mechanical ventilation: development of a preclinical ex vivo respiratory model for aerosol regional deposition. Sci Rep. 29 nov 2019;9(1):17930.

**Reference 2:** França EET, Andrade AFD de, Cabral G, Filho PA, Silva KC, Filho VCG, et al. Nebulization associated with Bi-level noninvasive ventilation: Analysis of pulmonary radioaerosol deposition. Respir Med. 1 avr 2006;100(4):721–8.

**Compliance with ethics regulations:** Yes in animal testing.
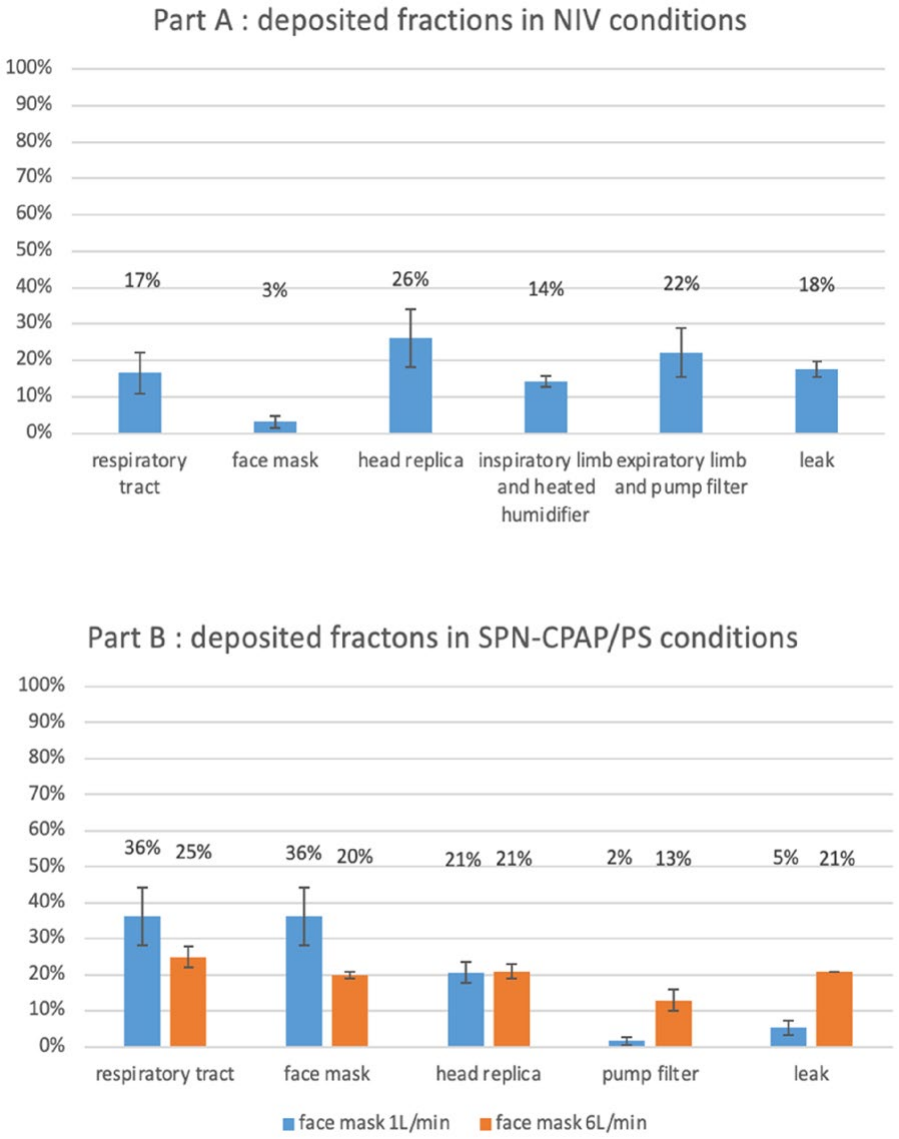


*Figure 1: for PART A and PART B, results express in % of nominal dose, mean and standard deviation.*

### FC-147 Clinical impact of the oximeters’ inaccuracy (bias and errors)—secondary analysis of the oxygap study

#### DELOBEL Antoine^1^, BLANCHET Marie-Anne^1^, MERCIER Gabriel^1^, NAYET Émi^1^, BOUCHARD Pierre-Alexandre^1^, SIMARD Serge^1^, L'HER Erwan^2^, BRANSON Richard^3^, LELLOUCHE François^1^

##### ^1^Centre de recherche, Institut Universitaire de Cardiologie et de Pneumologie de Québec, Québec (Qc), Canada; ^2^Medical Intensive Care Unit, CHRU Brest – La Cavale Blanche, Brest, France; ^3^Department of Surgery, Division of Trauma & Critical Care, University Cincinnati, Cincinnati, Etats-Unis.

###### Correspondence: Antoine DELOBEL (antoine.delobel.1@ulaval.ca)

*Annals of Intensive Care* 2022, **12(1):**FC-147

**Rationale:** Pulse oximetry is daily used worldwide to measure SpO_2_ in order to monitor and titrate oxygen support. We previously showed that four among the most frequently used oximeters have significant bias (systematic errors from − 3% to + 1%) and inaccuracy (random error) in comparison with the gold standard SaO_2_. The main objective of this study was to highlight the impact of the oximeters’ variability on monitoring and clinical decisions in acutely ill patients.

**Patients and methods/Materials and methods:** We prospectively included 210 stable ICU patients with an arterial catheter in place. For all included patients, we compared SpO_2_ values for each of the four evaluated oximeters (Nonin, Nellcor, Massimo and Philips) and concomitant PaO_2_ and SaO_2_ values from the arterial blood gases. When the patients met the criteria for hypoxemia (SaO_2_ < 90% or PaO_2_ < 60 mmHg), we evaluated which oximeter could detect hypoxemia (SpO_2_ < 90%). For each measurement, we evaluated for each oximeter when the oxygen support should be maintained even, increased or decreased to maintain a SpO_2_ value between 92 and 96%.

**Results:** Mean age of the patients was 66.3 year, 73% were men, skin pigmentation was light (Fitzpatrick 1 or 2 in 96.2% of the patients). The SpO_2_–SaO_2_ bias went from − 3.1% (Nonin), to + 0.9% (Philips) (P < 0.001). With Nellcor and Masimo, bias were − 0.3 (P < 0.03) and − 0.2% (P = 0.17) respectively. Nonin oximeter underestimated arterial oxygenation in 91% of the cases but detected 100% of hypoxemia defined by PaO_2_ < 60 mmHg. Philips overestimated oxygenation in 55% of the cases but detected 11% of episodes of hypoxemia. Based on the oximeter used, the management of oxygen support to maintain the patients within a SpO_2_ target of 92–96% differed a lot. To keep the patients within this SpO2 target, in the cohort of 210 patients, it would be required to increase oxygen in 60.6% vs. 19.1% vs. 18.7% vs. 10.4% and to decrease oxygen in 1.0% vs. 6.2% vs. 9.1% vs. 20.9% of the cases (p < 0.0001) when using Nonin, Nellcor, Masimo or Philips oximeter respectively (see Figure).

**Conclusion:** The variability of SpO_2_ measurements between oximeters has a significant impact on the monitoring and management of oxygen support. The bias of each oximeter should be known by clinicians and SpO_2_ targets should be adapted to the oximeter brand used by clinicians.

**Compliance with ethics regulations:** Yes in clinical research.
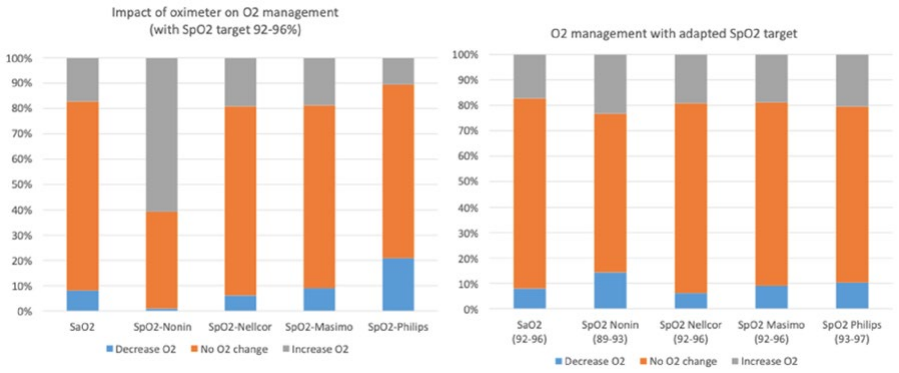


*Figure: impact of oximeter on oxygen management to maintain a SpO*_*2*_ *target between 92 and 96% (left panel) and impact with adapted SpO*_*2*_ *target to the oximeter (right panel).*

### FC-148 Accuracy and reliability of low-cost versus high-cost oximeters—a secondary analysis of the OXYGAP study

#### MERCIER Gabriel^1^, BLANCHET Marie-Anne^1^, DELOBEL Antoine^1^, NAYET Emi^1^, BOUCHARD Pierre-Alexandre^1^, LELLOUCHE François^1^

##### ^1^Institut de cardiologie et de pneumologie de Québec, Québec, Canada

###### Correspondence: Gabriel MERCIER (gabriel.mercier.3@ulaval.ca)

*Annals of Intensive Care* 2022, **12(1):**FC-148

**Rationale:** Pulse oximetry is a technology used regularly to monitor oxygen saturation and titrate oxygen needs. However, these machines are expensive, limiting access in universal health care especially in low-income countries. In recent years, low-cost oximeters have become more available, but there is little validation on their effectiveness. In the main study, we examined the bias between different high-cost and two low-cost oximeters. The main goal of this study was to compare the accuracy and reliability between some low-cost and high-cost oximeters to determine if low-cost oximeters can be adequate options in chosen situations.

**Patients and methods/Materials and methods:** This prospective study included 210 stable ICU patients with arterial catheter. Six oximeters were evaluated including four high-cost (Nonin (Plymouth, MN), Massimo (Irvine, CA), Philips (Eindhoven, Netherlands) and Nellcor (Pleasanton, CA)), as well as two low-cost oximeters (Contec CMS50DL and Beijing Choice C20). The low-cost ones were chosen based on a previous study on accuracy of low-cost oximeters were only the Contec CMS50DL and Beijing Choice C20 met the International Organization for Standardization (ISO) criteria for accuracy. Comparisons between pulse oximeter readings (SpO_2_) and arterial saturation (SaO_2_) were used to calculate bias (SpO_2_–SaO_2_). Reliability of each oximeter was evaluated by the percentage of times the oximeter could give a reading.

**Results:** Mean arterial saturation was 93.6%. High-cost oximeters had a mean bias between -3.1% with Nonin and 0.9% with Philips. In comparation, low-cost oximeters had a bias of − 2.9% for Contec CMS50DL and − 0.8% for Beijing Choice C20. While the majority of high-cost oximeter (Nellcor, Massimo, Philips) had a bias less than ± 1.0%, only Beijing Choice C20 had similar result. However, low-cost oximeters were less reliable than high-cost ones with Beijing Choice C20 giving readings 92.9% of the times vs high-cost oximeters giving readings > 97% of the times (See figure).

**Conclusion:** We found that some low-cost oximeters can have a similar accuracy with high-cost oximeters in some clinical settings. These low-cost oximeters could help giving more access to universal health care, especially in low-income countries.

**Reference 1:** Lipnick MS, Feiner JR, Au P, Bernstein M, Bickler PE. The Accuracy of 6 Inexpensive Pulse Oximeters Not Cleared by the Food and Drug Administration: The Possible Global Public Health Implications. Anesth Analg. 2016 Aug;123(2):338–45. https://doi.org/10.1213/ANE.000

**Compliance with ethics regulations:** Yes in clinical research.
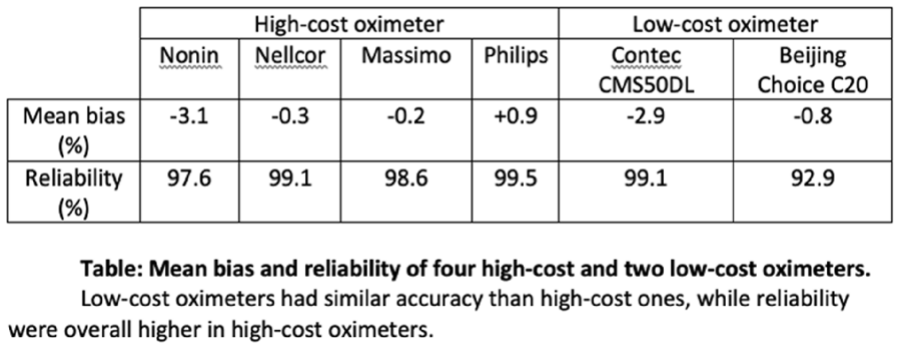


### FC-149 Invasive mechanical ventilation management and trends in COVID-19-related acute respiratory distress syndrome

#### TOUMI Radhouane^1,2^, MEDDEB Khaoula^1,2^, BOUGUEZZI Nabil^1^, ZOUARI Hajer^1^, BOUABDALLAH Nermine^1^, EL KACEH Rim^1^, BEN SAIDA Imen^1,2^, BOUSSARSAR Mohamed^1,2^

##### ^1^Medical Intensive Care Unit, Farhat Hached University Hospital, Sousse, Tunisie; ^2^Research Laboratory N° LR12SP09. Heart Failure. Farhat Hached University Hospital, University of Sousse, Sousse, Tunisie

###### Correspondence: Radhouane TOUMI (Radhouane.toumi@gmail.com)

*Annals of Intensive Care* 2022, **12(1):**FC-149

**Rationale:** COVID-19-related acute respiratory distress syndrome (CARDS) requiring invasive mechanical ventilation (IMV) is generally associated to a prolonged length of IMV and to a poor prognosis. Adapting IMV management according to patients’ elastic properties is mandatory to help reduce mortality. The aim of the study is to describe IMV management as in ventilator settings and parameters and trends in the management of CARDS requiring IMV.

**Patients and methods/Materials and methods:** This is a descriptive retrospective study carried out in a 12-bed medical intensive care unit (MICU) of a University Hospital from March 2020 to September 2021. All patients admitted for a critical CARDS requiring IMV were included. Data on patients’ characteristics at ICU admission, clinical presentation, ARDS and COVID-19 severity, ventilatory management, ventilatory settings, airways pressures, compliance, oxygenation index[(Plateau * FiO_2_ * 100)/PaO_2_)], ventilatory ratio [[VR = (Minute ventilation * PaCO_2_)/(Predicted body weight * 100 * 37.5)]] and outcomes were collected. Evaluation was carried out at H1 post-intubation, day5 and day10, at a fixed time. This is the second paper of the database served to inquire about several aspects of CARDS.

**Results:** During the study period, a total of 214 patients underwent IMV for CARDS; 200 (93.5%), severe to critical COVID-19. They were median aged 67.5 [59.75–73] y; male, 146 (68.2%); Hypertension, 106 (49.5%); diabetes, 104 (48.6%) and obesity, 89 (41.6%). Moderate ARDS, 69 (32.2%); severe ARDS, 141 (65.9%); median P/F ratio at admission, 95 [77–132]; moderate to severe acute respiratory failure 185 (86.4%); median SAPSS II, 33 [27–38]. Chest-CT scan was performed in 114 (53.3%) patients showing 68 (59.6%) with at least 50% of lesion extension. Table 1 displays trends in ventilator settings and physiological parameters at H1, day 5 and day 10. Overall mortality rate was 180 (84.1%).

**Conclusion:** Albeit poor outcome in mechanically ventilated CARDS patients, management was mostly in line with ARDS guidelines, however, trends seem to show progressive worsening of patient elastic properties.

**Compliance with ethics regulations:** Yes in clinical research.
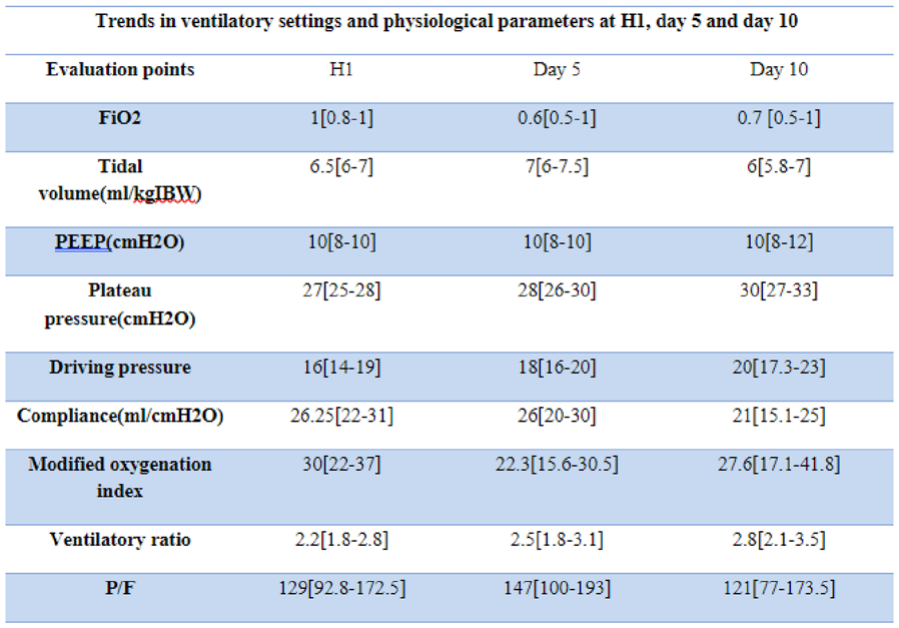


*Trends in ventilator settings and physiological parameters*

### FC-150 Hospital morbidity of COVID-19 patients: hospitalized patients from December 2021 to date

#### KADI Ahmed^1^, MEGDOUD Fella^1^, BOUGHERARA Abdelhak^1^, ZIDOUNI Noureddine^1^, GHARNAOUT Merzak^1^

##### ^1^CHU BENIMESSOUS ALGER ALGERIE, Alger, Algerie

###### Correspondence: Ahmed KADI (kadihh@hotmail.fr)

*Annals of Intensive Care* 2022, **12(1):**FC-150

**Rationale:** identify the characteristics of hospitalized patients and bring out the seriousness factors concerning COVID-19.

**Patients and methods/Materials and methods:** This observational, descriptive and retrospective study concerned 100 cases of SARS/COV2 infections from December 2021 to the present day, hospitalized in the pneumo-phtisiology department.

**Results:** The average age of the cases admitted to the study was 62 years with a male predominance (60%), the 2 most frequent comorbidities were respectively hypertension (44%) and diabetes (18%). Unvaccinated patients were 60% of cases, no patient had a history of COVID-19 disease. Family exposure represented 55% of cases, while occupational exposure was found in only 11% of cases. The involvement on chest CT was estimated at less than 10% in 3% of cases, between 10 and 25% in 15% of cases, between 25 and 50% in 40% of cases, between 50 and 75% in 28% of cases, and above 75% in 9% of cases. Lymphopenia was present in 93% of patients, CRP was elevated in 97% of patients. D. dimers were elevated in 52% of patients. Renal involvement was observed in 22% of patients. Cytolysis was present in 19% of hospitalized patients. Deaths accounted for 18% of hospitalized patients, half of whom were vaccinated.

**Conclusion:** Viral infection by the coronavirus is a new pathology with clinical manifestations of varying severity, digestive manifestations were more frequent in the series studied. Familial exposure was frequent. Inflammatory syndrome is common causing lesional pulmonary edema in some patients.

**Reference 1:** Wu Y, Ho W, Huang Y, Jin D-Y, Li S, Liu S-L, et al. SARS-CoV-2 is an appropriatename for the new coronavirus. Lancet 2020; 395 (10,228): 949–50.

**Reference 2:** Zhu N, Zhang D, Wang W, Li X, Yang B, Song J, et al. A novel coronavirus from patients with pneumonia in China, 2019. N Engl J Med 2020; 382 (8):727–33.

**Compliance with ethics regulations:** Yes in clinical research.

### FC-151 COVID-19 and underlying lung disease: what is known about risk and care management?

#### GHABARA Racha^1^, JARRAYA Fatma^1^, RACHDI Emna^1^, JAMOUSSI Amira^1^, AYED Samia^1^, BEN KHELIL Jalila^1^

##### ^1^Hôpital Abderrahmane Mami, Ariana, Tunisie

###### Correspondence: Fatma JARRAYA (fatma.jarraya8@gmail.com)

*Annals of Intensive Care* 2022, **12(1):**FC-151

**Rationale:** Patients with COVID-19 who have underlying chronic lung disease (ULD) are at increased risk for developing a severe illness related to the disease. However, to date, few studies have been published about patients with ULD. The aim of this study was to evaluate the impact of ULD on clinical characteristics, adverse outcomes, and mortality in patients with critical COVID-19.

**Patients and methods/Materials and methods:** It was a retrospective comparative cohort study including all patients hospitalized in ICU for COVID-19 between March 2020 and September 2021. Two groups were studied according to the presence or absence of ULD. Primary outcomes were the use of mechanical ventilation (MV), ICU length of stay (LOS), and death.

**Results:** We included 148 patients of whom 102 were male (ULD group, n = 74 versus no ULD group, n = 74). ULD corresponded to sleep apnea (SA; n = 12), chronic obstructive pulmonary disease (COPD; n = 31), asthma (n = 20), diffuse interstitial lung disease (n = 3), bronchiectasis (n = 4) and COPD/SA (n = 2). Long-term oxygen or home MV were required in 11 cases. Obesity was more frequent in ULD group (45% vs 39%; p = 0.01). COVID clinical presentation was similar between both groups (cough, dyspnea, struggle signs, respiratory rate, digestive signs, myalgia, anosmia). Pulmonary lesions on the first thoracic CT-scan performed during hospitalization were significantly more extensive in the no ULD group (p = 0.05). Ventilator parameters and lung mechanics were similar between both groups (Table 1). The occurrence of ARDS, thrombo-embolic events, and septic shock was comparable between both groups. Requirement and duration of MV did not differ (55% vs 45%; p = 0.25 and 7.84 vs 8.97 days; p = 0.52, respectively), but mortality rate was significantly higher in ULD group (n = 47, 63.5% vs n = 31, 41.9%; p = 0.008).

**Conclusion:** Unexpectedly, we didn’t find any major differences regarding the clinical presentation, severity scores at ICU admission, and the MV requirement between critical COVID-19 patients with ULD versus without ULD. However, the severity of CT scan injury and respiratory parameters were greater in the no ULD group. The mortality rate was more marked in the ULD group.

**Compliance with ethics regulations:** Yes in clinical research.
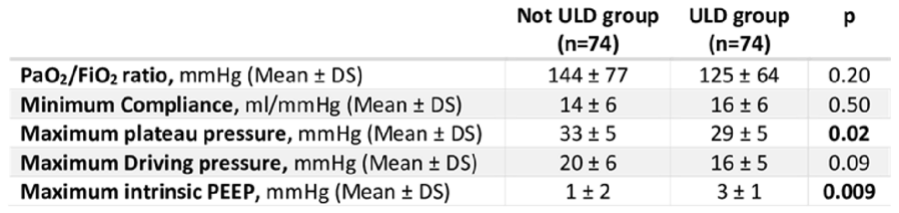


*Table 1: comparison of ventilator parameters and lung mechanics between both groups.*

### FC-152 Characteristics and prognosis of mechanical ventilated patients with SARS CoV2 pneumonia: a prospective analysis of 143 patients

#### MORATELLI Giulia^1^, REGAIEG Kais^1^, NAKAA Sabrine^1^, FRAJ Nesrine^1^, KALLEL Myriam^1^, AL HARACH Amir^1^, GAZAIGNE Laure^1^, GOLDGRAN-TOLEDANO Dany^1^

##### ^1^Groupe Hospitalier Intercommunal Le Raincy Montfermeil, Montfermeil, France

###### Correspondence: Giulia MORATELLI (giulia.moratelli@gmail.com)

*Annals of Intensive Care* 2022, **12(1):**FC-152.

**Rationale:** The aim of this study was to describe the epidemiological and clinical characteristics of patients with SARS CoV2 pneumonia mechanically ventilated admitted to an ICU of a general care center in France and to identify risk factors associated with mortality.

**Patients and methods/Materials and methods:** We performed a prospective cohort study of patients admitted to the ICU from March 2020 to February 2022 for SARS CoV2 pneumonia and mechanically ventilated.

**Results:** During the study period, we included 143 patients and the mortality rate was 51% (n = 72). Median ICU stay was 16 (1–149) days and median delay to ICU hospitalization was 8 (1–24) days. Median age was 62 (29–84), sex ratio M/F was 1.94 and median SAPS was 33 (18–75). Median BMI was 30 (19–68). 64% of patients had hypertension (n = 91) and 42% (n = 60) were diabetic. Patients presenting an acute kidney failure were 36% (n = 51) and 26% of them (n = 37) needed a renal replacement therapy. 82% of patients (n = 117) had non-invasive ventilation and 79% (n = 113) had high flow nasal oxygen as first-line therapy. Median duration of ventilation was 12 days (1–47), 47% of patients (n = 67) were extubated and 7.7% (n = 11) needed ECMO. 85% (n = 122) needed at least one prone position session. 85% of patients (n = 122) needed catecholamines. 73% of the patients (n = 105) had ventilatory acquired pneumonia, 17% (n = 25) had thrombotic complications while 23% (n = 33) had hemorrhagic complications. 37% of the patients (n = 53) received dexamethasone 6 mg/day for 10 days, 37% (n = 53) received dexamethasone 0.1 mg/kg/days for 10 days and 15% (n = 22) of them received bolus of methylprednisolone 250 mg/day for 3 days as rescue therapy. Tocilizumab was administrated to 9.8% of the patients (n = 14). In the univariate analysis, risk factors associated with mortality were age, hypertension, SAPS II, AKI, chronic renal disease, the use of catecholamines and hemorrhagic complications. In the multivariate analysis, only age (OR 1.09 [1.04; 1.14], p < 0.001) was associated with mortality.

**Conclusion:** The mortality of patient mechanically ventilated with SARS CoV 2 pneumonia associated is very high. Others studies are necessary to determine risk factors associated with mortality.

**Compliance with ethics regulations:** Yes in clinical research.

### FC-153 Is there a benefit to high-flow nasal cannula therapy in critically-ill COVID-19 patients ?

#### BEN ISMAIL Khaoula^1,4^, BEN DHIA Boudour^1,4^, TALIK Imen^1,4^, ESSAFI Fatma^1,4^, BAYOUDH Aida^2,4^, FESSI Ilhem^2,4^, JERBI Tahany^3^, CHKIRBENE Mariem^3^, MERHBENE Takoua^1,4^

##### ^1^Service de réanimation médicale Hôpital régional de Zaghouan, Zaghouan, Tunisie; ^2^Service de pneumologie Hôpital régional de Zaghouan, Zaghouan, Tunisie; ^3^Département de pharmacie de l'hôpital régional de Zaghouan, Zaghouan, Tunisie; ^4^Faculté de médecine de Tunis,Université Tunis El Manar, Tunis, Tunisie

###### Correspondence: Boudour DHIA (bidourabdbg2014@gmail.com)

*Annals of Intensive Care* 2022, **12(1):**FC-153

**Rationale:** During COVID-19 pandemic, high-flow nasal cannula therapy (HFNC) has been proposed as a ventilation device aiming to improve oxygenation and respiratory mechanic. However, its effectiveness has not been clearly demonstrated in terms of secondary need to invasive ventilation and reduction of morbidity and mortality. Our aim was to evaluate the efficacy and safety of HFNC in the management of non-hypercapnic acute respiratory failure secondary to SARS-CoV-2 infection.

**Patients and methods/Materials and methods:** Prospective, monocentric, interventional study conducted in the medical intensive care unit (ICU) of Zaghouan, Tunisia over 18 months (March 2020–September 2021). Patients admitted for non-hypercapnic acute respiratory failure secondary to SARS-CoV-2, aged over 18 years, were included. Two groups were identified G1 = patients who received HFNC alone or in combination with other non-invasive ventilation device and group G2 = patients who received conventional oxygen therapy in combination with non-invasive ventilation. We compared demographics, clinical, paraclinical and evolutionary data.

**Results:** 181 patients were included. HFNC was used in 111 patients (61.3%). It was associated with non-invasive ventilation and awake prone position in 83% and 92% of cases respectively. On admission, the two groups were comparable in terms of demographic characteristics, clinical presentation and initial severity. Mean P/F ratios were 152 and 139 mmHg respectively (p = 0.08). Comparison of outcome parameters showed that G1 patients need less likely invasive mechanical ventilation (27% vs 56.4%, p = 0.002) and developed fewer healthcare-associated infections and barotrauma (42.1% vs 62%, p = 0.03; 12% vs 22%, p = 0.03). Mortality was significantly lower in G1 (22% vs 39%; p = 0.02). Duration of total mechanical ventilation was comparable between two groups but length of ICU stay was longer in G1. Occurrence of pulmonary fibrosis was more frequent in G1 (18% vs 6%, p = 0.02). Multivariate analysis showed that use of HFNC was a protective factor against need to invasive ventilation (OR = 0.42; 95% IC [0.3–0.7], p = 0.03) and healthcare-associated infections (OR = 0.8; 95% IC [0.1–0.7], p = 0.04).

**Conclusion:** In this preliminary study, it appears that HFNC seems beneficial in severe COVID-19 patients. Further studies are needed to confirm these findings.

**Compliance with ethics regulations:** Yes in clinical research.

### FC-154 Risk factors of high-flow nasal canula oxygen therapy failure in critically ill patients with COVID-19

#### AIT HAMOU Zakaria^2^, LEVY Nathan^2^, CHARPENTIER Julien^2^, MIRA Jean-Paul^2^, JAMME Matthieu^3^, JOZWIAK Mathieu^1^

##### ^1^CHU de Nice, hôpital l'Archet 1, Nice, France; ^2^Hôpitaux Universitaires Paris centre, APHP, Hôpital Cochin, Paris, France; ^3^Hôpital Privé de l’Ouest Parisien, Trappes, France

###### Correspondence: Mathieu JOZWIAK (jozwiak.m@chu-nice.fr)

*Annals of Intensive Care* 2022, **12(1):**FC-154

**Rationale:** The use of high-flow nasal oxygen therapy (HFNO) as first-line support ventilatory might be associated with lower intubation rate in critically-ill COVID-19 patients. However, risk factors of HFNO failure remain to be determined.

**Patients and methods/Materials and methods:** In this retrospective study, we included all consecutive COVID-19 patients admitted in our 24-bed intensive care unit (ICU) in the first (March–May 2020) and second (August 2020–February 2021) pandemic waves. Patients with limitations for intubation were excluded. Risk factors of HFNO failure were identified through a landmark time-dependent cause-specific Cox model. The ability of the 6-h ROX index to detect HFNO failure was assessed by generating receiver operating characteristic (ROC) curve. The impact of HFNO use was analyzed in the whole cohort and after constructing a propensity score. The relationship between HFNO use and risk of intubation was assessed with a Cox cause-specific analysis.

**Results:** Overall 200 patients were included: HFNO was used in 114(57%) patients, non-invasive ventilation (NIV) in 25 (12%) patients and 145 (72%) patients were intubated with a median delay of 0 (0–2) days after ICU admission. Patients with HFNO were older and less severe than those without HFNO and 78 (68%) had HFNO failure. Patients with HFNO failure had a higher ICU mortality rate (34 vs 11%, p = 0.02) than those without. At landmark time of 48 and 72 h, SAPS-2 score, extent of CT-Scan abnormalities > 75% and HFNO duration (CSH = 0.11, 95% CI (0.04–0.28), per + 1 day, p < 0.001 at 48 h and CSH = 0.06, 95% CI (0.02–0.23), per + 1 day, p < 0.001 at 72 h) were associated with HFNO failure. The 6-h ROX index was lower in patients with HFNO failure but could not reliably predict HFNO failure with an area under ROC curve of 0.65 (95%CI (0.52–0.78), p = 0.02). In the matched cohort, there was a negative association between HFNO use and the risk of intubation (CSH = 0.32, 95% CI (0.19–0.57), p < 0.001).

**Conclusion:** In critically-ill COVID-19 patients, while HFNO use as first-line ventilatory support was associated with a lower risk of intubation, more than half of the patients had HFNO failure. The risk of HFNO failure could not be predicted by the 6-h ROX index but decreased after a 48-h HFNO duration.

**Compliance with ethics regulations:** Yes in clinical research.

### FC-155 Endotracheal intubation rate is lower in critically-ill SARS-CoV-2 patients requiring high-flow nasal oxygen receiving additional face-mask noninvasive ventilation: a retrospective bicentric cohort with propensity score analysis

#### URBINA Tomas^1^, ELABBADI Alexandre^2^, GABARRE Paul^1^, BIGÉ Naïke^1^, TURPIN Matthieu^2^, BONNY Vincent^1^, DESNOS Cyrielle^2^, BAUDEL Jean-Luc^1^, LAVILLGRAND Jean-Rémi^1^, HARIRI Geoffroy^1^, FARTOUKH Muriel^2^, GUIDET Bertrand^1^, DUMAS Guillaume^3^, VOIRIOT Guillaume^2^, AIT-OUFELLA Hafid^1^

##### ^1^Hôpital Saint-Antoine, Assistance Publique-Hôpitaux de Paris, Paris, France; ^2^Hôpital Tenon, Assistance Publique-Hôpitaux de Paris, Paris, France; ^3^Hôpital Saint-Louis, Assistance Publique-Hôpitaux de Paris, Paris, France

###### Correspondence: Tomas URBINA (tomas.urbina@aphp.fr)

*Annals of Intensive Care* 2022, **12(1):**FC-155

**Rationale:** SARS-CoV-2 pneumonia is responsible for unprecedented numbers of acute respiratory failure requiring invasive mechanical ventilation (IMV). We aimed to assess whether adding face-mask noninvasive ventilation (NIV) to high-flow nasal oxygen (HFNO) was associated with a reduced need for endotracheal intubation.

**Patients and methods/Materials and methods:** This retrospective cohort study was conducted from July 2020 to January 2021 in two tertiary care intensive care units (ICUs). Patients admitted for laboratory confirmed SARS-CoV-2 infection with acute hypoxemic respiratory failure requiring HFNO with or without NIV were included. The primary outcome was the rate of endotracheal intubation. Secondary outcomes included day-28 mortality, day-28 respiratory support and IMV free days, ICU and hospital length-of-stay. Sensitivity analyses with both propensity score matching and overlap weighting were used.

**Results:** 128 patients were included, 88 (69%) received HFNO alone and 40 (31%) received additional NIV. Additional NIV was associated with a reduced rate of endotracheal intubation in multivariate analysis (53 (60%) vs 15 (38%), HR = 0.46 (95% CI, 0.23–0.95), p = 0.04) (Fig. 1). Sensitivity analyses by propensity score matching (HR = 0.45 (95%IC, 0.24–0.84), p = 0.01) and overlap weighting (HR = 0.52 (95% CI, 0.28–0.94), p = 0.03) were consistent. Day-28 mortality was 25 (28%) in the HFNO group and 8 (20%) in the NIV group (HR = 0.75 (95%CI, 0.15–3.82), p = 0.72). NIV was associated with higher IMV free days (20 (0–28) vs 28 (14–28), p = 0.015). All sensitivity analyses were consistent regarding secondary outcomes.

**Conclusion:** Need for endotracheal intubation was lower in critically-ill SARS-CoV-2 patients receiving face-mask noninvasive mechanical ventilation in addition to high-flow oxygen therapy. Further prospective trials are needed before this treatment can be generally recommended.

**Compliance with ethics regulations:** Yes in clinical research.
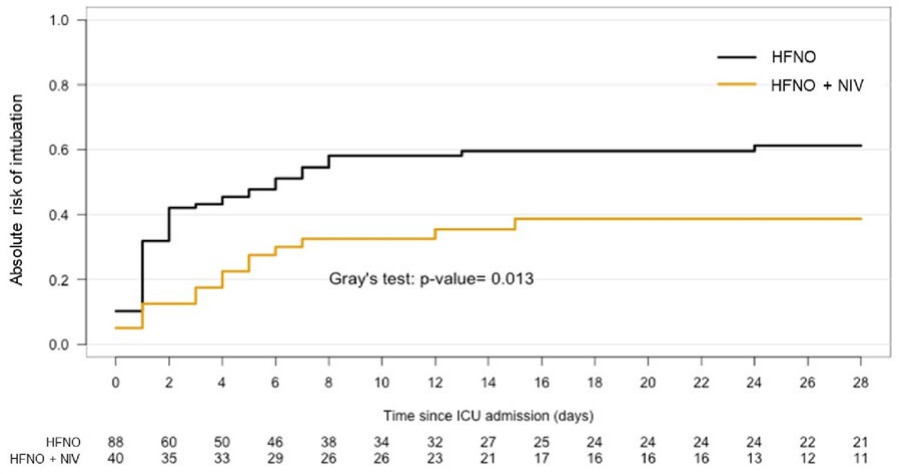


*Figure 1. Cumulative incidence of intubation to day 28 in the high-flow nasal oxygen only and the additional face-mask noninvasive ventilation groups. Hazard ratio from a Cox model multivariate analysis in the whole population was 0.46 (95%CI, 0.23–0.95),*

### FC-156 Patient outcomes in a respiratory weaning unit after prolonged intensive care: a review of 2021

#### PERETOUT Jean-Baptiste^1^, NSSAIR Karim^1^, ABDALLAH Razach^1^, LEPECQ Raphael^1^, DONETTI Laurence^1^, LAISSI Mohamed^1^, CHOUKROUN Gérald^1^

##### ^1^Hôpital Forcilles - Fondation Cognacq-Jay, Férolles-Attilly, France

###### Correspondence: Jean-Baptiste PERETOUT (jperetout@cognacq-jay.fr)

*Annals of Intensive Care* 2022, **12(1):**FC-156

**Rationale:** Weaning units have shown their value in the management of tracheostomized patients after prolonged intensive care^1^. The global and multidisciplinary approach contributes to accelerate and improve weaning^2^. The objective of this study is to describe the results of weaning and decannulation in these patients.

**Patients and methods/Materials and methods:** This is a retrospective, descriptive study in a respiratory weaning unit over the year 2021. All patients hospitalized in the weaning unit were included. Demographic characteristics and weaning unit outcomes were collected. All inpatients with complete data were analyzed. The analysis was completed by a subgroup analysis of patients on 24-h spontaneous ventilation at admission and patients still with mechanical ventilation (MV) at admission (intermittent or continuous).

**Results:** In 2021, 144 patients were admitted. A total of 134 patients were analyzed with male 70%, age 63 ± 12 years, COVID 70%. The main diagnosis of admission on intensive care unit was acute respiratory failure in 84%. The goal of admission on weaning unit was: ventilatory and tracheostomy weaning in 63% and tracheostomy weaning in 37%. On admission, patients were on 24-h spontaneous ventilation in 34% (n = 46) and on mechanical ventilation in 66% (n = 88): 66% intermittent MV (n = 58); 34% continuous MV (n = 30). In overall patients, 24-h spontaneous ventilation was achieved in 8.4 ± 12.8 days, decanulation in 20.4 ± 15 days and the length of stay was 30.7 ± 18.3 days. The overall decanulation success rate was 88%. Subgroup analyses are presented in Table 1.

**Conclusion:** Patients with prolonged intensive care and complex medical needs treated at a specialized weaning unit had high quality weaning and decannulation rates. This was achieved with a multidisciplinary team approach to continued intensive care and simultaneous rehabilitation with physiotherapists, speech therapists, nutritionists… Cares in a weaning unit remains relevant despite a prolonged stay in ICU during this COVID period.

**Reference 1:** Boles JM, Bion J, Connors A, Herridge M, Marsh B, Melot C, Pearl R, Silverman H, Stanchina M, Vieillard-Baron A, Welte T. Weaning from mechanical ventilation. Eur Respir J. 2007 May;29(5):1033–56.

**Reference 2:** Nelson JE, Cox CE, Hope AA, Carson SS. Chronic critical illness. Am J Respir Crit Care Med. 2010 Aug 15;182(4):446–54.

**Compliance with ethics regulations:** Yes in clinical research.
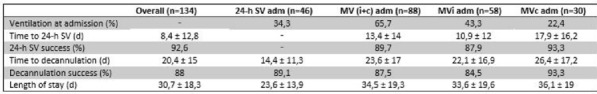


Table I* Subgroup analysis. 24-h SV adm: 24-h spontaneous ventilation on admission; VM (i* + *c) adm: mechanical ventilation (intermittent* + *continuous) on admission; MVi adm: intermittent mechanical ventilation on admission; MVc adm: continous mech.*

### FC-157 Pulmonary consequences at 12 months of a severe COVID-19 infection

#### SAINT JOUAN Mélanie^1^, MORICHAU-BEAUCHANT Tristan^1^, MAILLET Jean-Michel^1^

##### ^1^Centre Cardiologique du Nord, Saint Denis, France

###### Correspondence: Mélanie SAINT JOUAN (Melaniesaintjouan@Gmail.com)

*Annals of Intensive Care* 2022, **12(1):**FC-157

**Rationale:** Two years after the first wave of the SARS-CoV-2 epidemic in France, the pandemic is still active, but data about the long-term consequences of the pulmonary function are limited.

In this context, we have carried out an evaluation of the pulmonary function of patients who survived severe ARDS secondary to SARS-CoV-2 infection, 1 year after their hospitalization.

**Patients and methods/Materials and methods:** We have carried out a single-center prospective and descriptive cohort study with patients who survived severe ARDS during the first wave of the pandemic between the months of March to April 2020. This study took place from April 2021 to July 2021. The patients included in this study had required a prolonged stay (more than seven days) in intensive care unit and were under mecanical invasive ventilation for more than three days during the first wave of the pandemic. The main goal was to assess the lung function. The assessment included a chest CT-scan, pulmonary function tests, a 6-min walk test, and an evaluation of patient's quality of life depending on their respiratory symptoms using the St George’s respiratory survey. The patients were hospitalized for a day in the center for these exams.

**Results:** 30 patients were included in the study, of which 8 patients were under veno-venous ECMO during their hospitalization.

The chest CT-scans revealed few ground-glass opacities, with a median scanographic severity score of 1 out of 25 [IQR 0–2.5]; 43% of the CT-scans were normal. On the pulmonary function tests 32% of patients had restrictive ventilatory defects, and 45% of patients had diffusing capacity of the lungs for carbon monoxide impairment. The impact on functional abilities assessed by the six-minute walk test resulted in a distance traveled less than normal in 33% of patients. The results of the St George's Hospital survey showed that quality of life was altered, with results similar to those of ARDS survivors.

**Conclusion:** This is the first cohort study assessing long-term respiratory sequelae only patients who suffered from severe COVID-19 infection, 1 year after ICU discharge. The majority had abnormal lung function tests and residual parenchymal abnormalities on CT scans. Diffusing capacity of the lungs for carbon monoxide and total lung capacity were impaired in one-third of patients. Limitation of physical activity has been documented. Despite, they were autonomous and living at home, which is encouraging for the functional prognosis of patients with severe forms of COVID-19.

**Compliance with ethics regulations:** Yes in clinical research.

### FC-158 Interest of non-invasive ventilation in the prevention of post-extubation respiratory distress in medical intensive care

#### OUALI Mourad^1^, HAMIDI Redha Malek^1^

##### ^1^Faculté de médecine; Centre hospitalo-universitaire Béni Messous, Alger, Algérie

###### Correspondence: Mourad OUALI (moudoc2002@yahoo.fr)

*Annals of Intensive Care* 2022, **12(1):**FC-158

**Rationale:** The failure of extubation defined by the need for reintubation of the patient less than 72 h later is correlated with excess mortality as well as an increase in the length of stay in intensive care, this failure is often due to the occurrence acute respiratory failure after extubation.

**Patients and methods/Materials and methods:** In order to assess the impact of non-invasive prophylactic intermittent ventilation on the frequency of occurrence of post-extubation respiratory distress, a comparative, descriptive, single-center and randomized study was conducted in a medical intensive care unit over a period of 2 years (2018–2020). 73 patients hospitalized in intensive care, put on invasive mechanical ventilation for more than 24 h and extubated after having tolerated the spontaneous ventilation challenge well, were randomized to receive non-invasive intermittent ventilation for 24 h in a group then conventional oxygen therapy and in another group conventional oxygen therapy alone. The primary endpoint was the occurrence of respiratory distress after extubation.

**Results:** The results allowed us to observe a significant decrease in the occurrence of post-extubation respiratory distress in patients in the NIV group compared to patients in the conventional oxygen therapy group (7.9% VS 28.6%; P = 0.02), as well as a reduction in the reintubation rate (7.9% in the NIV group versus 20% in the oxygen only group; p = 0.13). The length of stay in the intensive care unit was longer in patients who presented with acute respiratory failure (25.77 ± 5.0 days in the respiratory distress group versus 15.37 ± 0.85 days in the group without respiratory distress; p = 0.001) but without significant difference between the NIV group and the conventional oxygen group, p = 0.64. Post-extubation respiratory distress was correlated with significant excess mortality with 46.2% mortality in the group of patients who presented with respiratory distress after extubation versus no death in the group of patients who did not develop this complication, p < 0.001. In contrast, we did not notice a significant difference in mortality between the two study groups.

**Conclusion:** Our study has made it possible to highlight all the interest that NIV can bring in the period following the planned extubation of patients in intensive care, by demonstrating a significant reduction in the frequency of occurrence of acute respiratory failure after extubation, the reduction in the rate of reintubation as well as the shortening of the length of stay in intensive care.

**Reference 1:** Xu Z, Li Y, Zhou J, Li X, Huang Y, Liu X, et al. High-flow nasal cannula in adults with acute respiratory failure and after extubation: a systematic review and meta-analysis. Respir Res. 2018;19(1):202.

**Reference 2:** Zhu Y, Yin H, Zhang R, Ye X, Wei J. High-flow nasal cannula oxygen therapy versus conventional oxygen therapy in patients after planned extubation: a systematic review and meta-analysis. Crit Care. 2019;23(1):180.

**Compliance with ethics regulations:** N/A.

### FC-159 One-year follow-up of patients and relatives after severe COVID-19 related ARDS treated in the intensive care unit

#### THIERY Guillaume^1^, LAURENT Raphael ^1^, CORREIA Patricia ^1^, EZINGEARD Eric^1^, ERIC Diconne^1^, LACHAND Raphael^1^, PERINEL-RAGEY Sophie^1^, BRUNA Franklin^1^, DIREZ Magali^1^, ROUSSET Elodie^1^, CURTO Nadège^1^, PINATEL Emilie^1^, CHARRA Céline^1^, PHILIBERT Gérald^1^, GUENIER Pierre Alban^1^

##### ^1^CHU de Saint-Etienne, St Priest En Jarez, France

###### Correspondence: Guillaume THIERY (thiery.icu@gmail.com)

*Annals of Intensive Care* 2022, **12(1):**FC-159

**Rationale:** Severe COVID-19 often leads to prolonged stay in the ICU. Survivors may experience physical and psychological sequelea. The impact on relatives is also considerable. The aim of this study is to evaluate physical and psychological condition of severe COVID-19 patients 1 year after ICU discharge and to assess the psychological impact on their relatives.

**Patients and methods/Materials and methods:** All consecutive patients admitted to the ICU for COVID-19 related ARDS from March 5th to November 21st 2020 were included in our post-ICU follow-up program, if their ICU stay was ≥ 7 days. They were invited to attend a post-ICU consultation, which consisted of a 2-h face to face meeting with a senior ICU physician and an ICU nurse. Before the consultation, they were asked to complete four self-evaluation questionnaires: Hospital Anxiety and Depression scale (HADS), Revised Impact of Event Scale (IES-R), Short-Form General Health Survey (SF-12) and Inconforts des Patients de REAnimation (IPREA). In addition, a CT-scan and basic laboratory tests were performed before the consultation. Patients were invited to be accompanied by a close relative, who was also invited to fill HADS and IES-R questionnaires.

**Results:** Among the 170 patients admitted to the ICU, 127 patients survived to the hospital and 81 met inclusion criteria. Twenty-five patients did not respond or declined. The remaining 56 patients attended the consultation. The median age was 66 (IQR 57–71), 79% had been mechanically ventilated. Duration of ICU and hospital (including rehabilitation) stays were 19 (IQR 12–26) and 49 (IQR 26–64) days respectively. Severe symptoms of anxiety, depression and PTSD are reported on table 1. The most frequently reported discomforts were limited visiting hours, sleep deprivation, anxiety, being tied down and noise. Fifty-nine percent of the patients resumed their hobbies at 1 year. Five (28%) of the 18 pre-ICU employed patients fully returned to work. Shortness of breath was reported by 64% of the patients, although among the 42 CT scan performed, only 7 (16%) showed persistent abnormalities. Symptoms of fatigue were reported by 63% of the patients, and physical functioning was reduced with a physical component of SF-12 score at 44.

**Conclusion:** One year after their ICU discharge, the psychological impact of severe COVID-19 for patients treated in the ICU is high, and is even higher for relatives. A majority of patients present still with persistent respiratory symptoms although most of them have a lung CT scan normal.

**Compliance with ethics regulations:** Yes in clinical research.
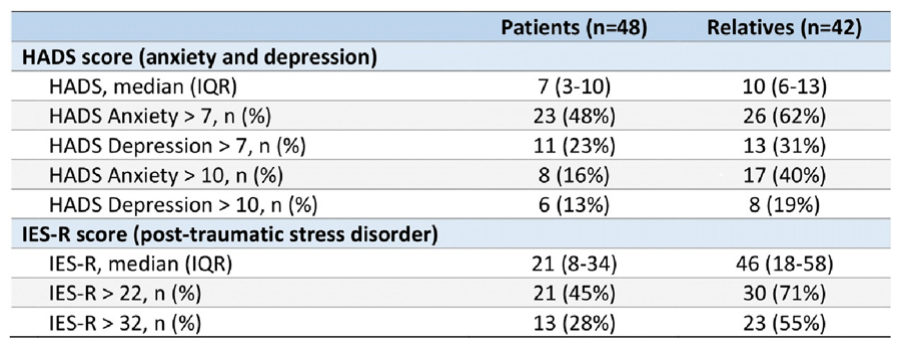


*Table 1*

### FC-160 Tracheostomy in patients admitted due to COVID-19

#### SANA Khedher^1^, ALI Rashed^1^, ELDHAWI Nawar^1^, HASSAN Majid Al Dossari^1^

##### ^1^AFH Wady addawassir, Riadh, Arabie Saoudite.

###### Correspondence: Khedher SANA (sanakhedher@hotmail.fr)

*Annals of Intensive Care* 2022, **12(1):**FC-160

**Rationale:** During the COVID-19 pandemic, the world has seen an increase in the number of critically ill patients who required invasive mechanical ventilation (IMV). Ventilation is certainly a challenge and tracheostomy was one of the management strategies.

**Patients and methods/Materials and methods:** We reviewed the medical records of all patients admitted to our intensive care, between April 01, 2020 and August 31, 2021, suffering from acute respiratory syndrome due to SARS COV2 and requiring tracheostomy after IMV. The objective was to determine the rate as well as the clinico-epidemiological profile of tracheotomized patients.

**Results:** A total of 96 patient admitted during this period. Fourteen 40 (41%) had acute respiratory failure and needed IMV support. Ten (25%) had a tracheostomy, they were predominantly male (55%) with mean of age 70+ − 15 years and all having at least one comorbidity. Mean SAPSII score on ICU admission was 34+ − 16. The average time from endotracheal intubation to tracheostomy was 20+ − 8 days. Failure to weaning (50%) was the most common indication, followed by respiratory failure (30%), hypersecretion (10%), and neuromyopathy (10%). 50% were liberated from ventilator, 20% have been decannulated and all patient have been discharged alive. Here we have opted open surgical technique. Two episodes of local bleeding were observed. No transmission has occurred among health workers.

**Discussion:** Several previous studies have shared their experiences and have shown that tracheostomy done in the correct manner is safe for the health care workers and provides good recovery for the severely ill with SARS-COV2 pneumonia.

**Conclusion:** Use of tracheostomy can be adopted as essential clinical strategy for managing critical ill patients with SARS COV 2 pneumonia who require IMV.

**Reference 1:** Tiffany N. Chao et al. Outcomes after tracheostomy in COVID-19 patients. Ann Surg 2020;272:e181–e186.

**Reference 2:** Zhou F, et al. Clinical course and risk factors for mortality of adult In patients with COVID-19 in Wuhan, China: a retrospective cohort study. Lancet. 2020;395:1054–1062.

**Compliance with ethics regulations:** Yes in clinical research.

### FC-161 Clinical features, ventilatory management and outcomes of COVID-19 related ARDS in a medical ICU

#### TOUMI Radhouane^1,2^, MEDDEB Khaoula^1,2^, BOUGUEZZI Nabil^1^, ZOUARI Hajer^1^, MAATOUK Iyed^1^, HAMDI Dhouha^1^, BEN SAIDA Imen^1,2^, BOUSSARSAR Mohamed^1,2^

##### ^1^Medical Intensive Care Unit, Farhat Hached University Hospital, Sousse, Tunisie; ^2^Research Laboratory N° LR12SP09. Heart Failure. Farhat Hached University Hospital, University of Sousse, Sousse, Tunisie

###### Correspondence: Radhouane TOUMI (Radhouane.toumi@gmail.com)

*Annals of Intensive Care* 2022, **12(1):**FC-161

**Rationale:** The COVID-19 pandemic took the world by storm defying physicians in many aspects; going from health systems responsiveness to diagnosis and treatment, especially with the introduction of concepts such as “happy hypoxemia” that modulated intensivists’ approach to ventilatory management. The aim of the present study is to describe clinical features, ventilatory management and outcomes of critically ill adults admitted for COVID-19 related ARDS.

**Patients and methods/Materials and methods:** This is a descriptive retrospective study carried out in a 12-bed medical intensive care unit (MICU) of a University Hospital from March 2020 to September 2021. All patients admitted for a critical COVID-19 related ARDS were included. Data on patients’ characteristics at ICU admission, clinical presentation, ARDS and COVID-19 severity, ventilatory management, ventilatory settings, airways pressures and outcomes were collected. The database served to inquire about several aspects of COVID-19 related ARDS that will be discussed through a series of studies.

**Results:** During the study period, 442 patients were admitted for COVID-19 pneumonia, 409 (92.6%) presenting severe to critical COVID-19. They were aged 64 [54–71] years; male, 279 (63.1%); had hypertension, 197 (44.6%); diabetes mellitus, 187 (42.3%), and obese 172 (38.9%). At admission, moderate to severe acute respiratory failure, 340 (76.9%); median SAPS II, 28 [22–35]; moderate to severe ARDS, 400 (90.5%); mean P/F ratio, 114 [84–157]. Chest CT-scan performed in 268 (60.6%) patients, 163 (60.8%) had more than 50% of pulmonary parenchyma lesions. Ventilatory support was required in 439 (99.3%); immediate IMV at admission, 33 (7.5%); HFNC, 334 (77.8%); median HFNC duration, 4 [2–7] days; NIV and CPAP as rescue therapy to avoid intubation, respectively, 52 (11.8%); 10 (2.2%), median NIV starting day, day 1 [0–2]; median NIV duration, 2 [1–33] days; CPAP starting day, day 3 [1–3]; median CPAP duration, 2 [1–4] days. Non invasive procedures failure rate, 41.2%; median delay of intubation, 3 [2–5] days; overall IMV, 215 (48.6%); median overall IMV starting day, day 2 [1–4]; median IMV duration, 8 [5–12] days. Overall mortality was at 211 (47.7%), 130 (37.8%) for patients on HFNC, 93 (72.7%) when patients were switched to NIV and 180 (83.72%) when on IMV.

**Conclusion:** The present study demonstrated a poor prognosis in mechanically ventilated patients with COVID-19 related ARDS. HFNC was prioritized for most patients. NIV was used as a rescue therapy in an attempt to avoid intubation.

**Compliance with ethics regulations:** Yes in clinical research.

### FC-162 Doses of dexamethasone in Covid-19 patients

#### SDIRI Ines^1,2^, FITOUHI Nizar^2^, DRIDI Amira^2^, LAROUI Rim^1^, OUERGHI Sonia^2^, MESTIRI Tahar^2^

##### ^1^Hôpital Circonstance menzah, Tunis, Tunisie; ^2^Abderahmen memi ariana, Ariana, Tunisie

###### Correspondence: Ines SDIRI (sdiri.ynes@gmail.com)

*Annals of Intensive Care* 2022, **12(1):**FC-162

**Rationale:** The SARS-CoV-2 induces both systemic and localized immune response leading to increased inflammation. Severe pulmonary lesions in SARS-Cov-2 ARDS have been found to be associated with severe inflammatory cell infiltration and elevated pro-inflammatory cytokines (1). Dexamethasone decreases mortality in patients with severe coronavirus disease 2019 (COVID-19) and has become the standard of care (2). The role of dexamethasone in the high incidence of ventilator-associated pneumonia observed in intensive care units (ICUs) deserves to be investigated.

**Patients and methods/Materials and methods:** We conducted a retrospective study in an intensive care unit including critically ill patients with confirmed SARS-COV2 infection between January 2021 and December 2021. We compared two different doses of dexamethasone (6 mg×3 per day versus 6 mg per day) given intravenously over 10 days. Secondary, our population was divided into two groups: those who received 6 mg * 3 (group 1) and those who received only 6 mg (group 2). These two groups were matched on age, gender, SAPSII and APACHE scores, severity of acute respiratory distress syndrome on admission and clinical severity.

**Results:** We evaluated 323 patients with COVID-19 admitted to our institution. The median age was 57 [25–85] years and 53.6% of the patients were male. Among these patients, 37.3% had hypertension, 33.1% had diabetes and 12.1% had chronic lung disease. On admission, all patients presented respiratory distress with CT injury superiors to 50% in 49.7% of patients. 57.7% patients were intubated during hospitalization. ICU length of stay was 9 ± 8 days. ICU mortality was 45%. All patients received dexamethasone. 77.2% were included in group 1 and 20.5% patients in group 2. There was no difference in acute kidney injury rate (17.6% vs. 3.4%; p = 0.275) or the need of mechanical ventilation (59.5% vs 45.4%; p = 0.11) between the two groups. We noticed a higher incidence of nosocomial infection without signification (61.1% vs 45.8%; p = 0.095). However, in the high dose dexamethasone group, we contented a higher rate of mortality not significant (48.3% vs. 29.1%; p = 0.059). Besides, we noted more hyperglycemia and hypokalemia in the group 1 ((53% vs. 34%; p = 0.04), (12% vs. 6%; p = 0.039)).

**Conclusion:** Different doses of corticosteroids seem to increase the rate of nosocomial infection and do not affect mortality. However, the high-dose group patients had more metabolic complications.

**Reference 1:** Maskin LP, Olarte GL, Palizas F, Velo AE, Lurbet MF, Bonelli I, et al. 26 août 2020;21.

**Reference 2:** J Biol Regul Homeost Agents. août 2020;34.

**Compliance with ethics regulations:** Yes in clinical research.

### FC-163 The impact of solumedrol in patients with COVID-19 related acute respiratory distress syndrome (ARDS)

#### SDIRI Ines^1^, FITOUHI Nizar^1^, DRIDI Amira^1^, DORGHAM Sana^1^, OUERGHI Sonia^1^, MESTIRI Tahar^1^

##### ^1^Hôpital ariana abderahmen memi, Sfax, Tunisie

###### Correspondence: Ines SDIRI (sdiri.ynes@gmail.com)

*Annals of Intensive Care* 2022, **12(1):**FC-163

**Rationale:** Coronavirus disease 2019 (Covid-19) is associated with diffuse lung damage. Glucocorticoids may modulate inflammation-mediated lung injury and thereby reduce progression to respiratory failure and death. The aim of our study was to evaluate the impact of methylprednisolone boli (1) on the prognosis of patients admitted to the ICU for covid 19 pneumonia.

**Patients and methods/Materials and methods:** A retrospective study in intensive care unit of circumstance hospital over the period from January to September 2021. All admitted patients were systematically put on dexamethasone intravenously for 10 days, some of them had a stagnant respiratory status during the hospitalization, received a bolus of solumedrol 5 days in a row, at a dose of 250 mg/day and followed by hydrocortisone hemisuccinate at a dose of 1 mg/kg/day of methylprednisolone equivalent with progressive digression over 15 days, according to the clinician's judgement, ensuring the absence of concomitant infection. Secondary, our population was divided into two groups: those who received solumedrol (group 1) and those who received only dexamethasone (control group). These two groups were matched on age, gender, SAPSII and APACHE scores, severity of acute respiratory distress syndrome on admission and clinical severity.

**Results:** Our study population included 82 patients, 41 in each group. The two groups were matched for age (p = 0.9), gender (p = 1), and different comorbidities (obesity, p = 0.19, cardiovascular history p = 0.009 and respiratory history p = 0.3). At admission, 35 patients (92%) in group 1 had moderate to severe ARDS (p = 0.9). However, the solumedrol group had a more severe clinical presentation at admission: oxygen saturation on admission (89.5% ± 9 versus 92% ± 5 in the control group, (p = 0.02); more signs of struggle (15 patients versus 8 in the control group, p = 0.07). Solumedrol administration was performed within a median of 4 days [2–6], with respiratory worsening occurring within a median of 4 ± 3 days (p = 0.05). The use of invasive mechanical ventilation was more frequent in the G1 (25 patients versus 20, p = 0.008). The occurrence of nosocomial infection was more frequent in the G1 (31 vs. 21 patients, p = 0.0001). The duration of invasive mechanical ventilation, length of stay were comparable between the two groups (p = 0.3 and p = 0.08 respectively). The prescription of bolus solumedrol was associated with higher rate of mortality (69% G1 vs. 45%; p = 0.005) and more shock (p = 0.004).

**Conclusion:** Patients who received solumedrol had a more severe clinical presentation, with more invasive mechanical ventilation, more nosocomial infection and higher rate of mortality (2). A randomized study is needed to assess the efficacy of solumedrol compared with dexamethasone.

**Reference 1:** Pinzón MA, Ortiz S, Holguín H, Betancur JF, Cardona Arango D, Laniado H, et al.

**Reference 2:** Edalatifard M, Akhtari M, Salehi M, Naderi Z, Jamshidi A, Mostafaei S, et al.

**Compliance with ethics regulations:** Yes in clinical research.

### FC-164 Therapeutic plasma exchange in patients with covid-19 pneumonia in intensive care unit: cases series

#### ZAID Ikram^1^, ESSAAD Ounci^1^, EL AIDOUNI Ghizlane^1^, AABDI Mohammed^1^, BERRICHI Samia^1^, TAOUIHAR Salma^1^, MARBOUH Manal^1^, BKIYER Houssam^1^, ABDA Naima^1^, HOUSNI Brahim^1^

##### ^1^Centre Hospitalier Universitaire Oujda, Oujda, Maroc

##### Correspondence: Ikram ZAID (ikramzaid1993@gmail.com)

*Annals of Intensive Care* 2022, **12(1):**FC-164

**Rationale:** COVID 19 pneumonia can lead to an inappropriate inflammatory response, and can be complicated by acute respiratory distress syndrome, multi organ failure with a high mortality rate. The objective of this study is to observe the effect of therapeutic plasma exchange on the excessive inflammatory response.

**Patients and methods/Materials and methods:** In this study, we included 7 confirmed cases of COVID-19 in the intensive care unit (ICU). COVID-19 cases were confirmed by RT PCR (reverse transcription polymerase chain) and CT (computerized tomography) imaging according to WHO guidelines. Therapeutic plasma exchange was performed to decrease cytokine storm-induced ARDS (Acute respiratory distress syndrome). Inflammation marker assays were performed before and after therapeutic plasma exchange to assess its efficacy.

**Results:** Levels of inflammatory cytokines (IL-6) and acute phase response proteins, including ferritin and CRP, were elevated before therapeutic plasma exchange. After therapeutic plasma exchange, levels of acute phase reactants and inflammatory mediators were significantly reduced (p < 0.05).

**Conclusion:** Our data suggest that therapeutic plasma exchange reduces the inflammatory response in patients with severe COVID-19 not undergoing mechanical ventilation. Further studies are needed to explore the efficacy of therapeutic plasma exchange in patients with COVID-19.

**Compliance with ethics regulations:** Yes in clinical research.

### FC-165 Impact of corticosteroid use in critically-ill COVID-19 patients on mortality, intubation, and adverse events: a French multicenter study

#### RAYMOND Matthieu^1^, LE THUAULT Aurélie^1^, ASFAR Pierre^2^, DARREAU Cédric^3^, REIZINE Florian ^4^, COLIN Gwenhaël^5^, DANO Charly^6^, LORBER Julien^7^, HOURMANT Baptiste^8^, DELBOVE Agathe^9^, FREROU Aurélien^10^, MORIN Jean^1^, ERGRETEAU Pierre Yves^11^, SEGUIN Philippe^4^, REIGNIER Jean^1^, LASCARROU Jean Baptiste^1^, CANET Emmanuel^1^

##### ^1^CHU de Nantes, Nantes, France; ^2^CHU d'Angers, Angers, France; ^3^CH du Mans, Le Mans, France; ^4^CHU de Rennes, Rennes, France; ^5^CHD La Roche sur Yon, La Roche Sur Yon, France; ^6^CH de Cholet, Cholet, France; ^7^CH de Saint Nazaire, Saint Nazaire, France; ^8^CHU de Brest, Brest, France; ^9^CH de Vannes, Vannes, France; ^10^CH de Saint Malo, Saint Malo, France; ^11^CH de Morlaix, Morlaix, France

###### Correspondence: Matthieu RAYMOND (matthieu.raymond@chu-nantes.fr).

*Annals of Intensive Care* 2022, **12(1):**FC-165

**Rationale:** The use of corticosteroids is recommended for the management of critically-ill COVID-19 patients based on the results of the RECOVERY trial [1], which reported a reduced day-28 mortality in patients requiring oxygen therapy or mechanical ventilation. However, the effectiveness and safety of corticosteroids still remain a matter of debate.

**Patients and methods/Materials and methods:** We conducted a multicenter observational study. All Covid-19 patients admitted in 13 ICUs from Pays-de-la-Loire and Bretagne regions between February 1st 2020 and December 31th 2020 were included. The main objective was to assess the impact of an early course of dexamethasone on day-28 mortality.

**Results:** Overall, 1058 patients were included of whom 644 (60.87%) were intubated. An early course of dexamethasone (dexamethasone group) was given to 611 (57.75%) patients while 447 (42.25%) patients did not receive an early course of dexamethasone (usual care group). Day-28 mortality was 15.4% in the whole population, 15.1% in the dexamethasone group, and 15.9% in the usual care group (p = 0.63). On multivariable analysis, age (aHR 1.06 (1.03–1.08), p < 0.001), SOFA score (aHR 1.15 (1.09–1.21), p < 0.001), and immunocompromised status (aHR 1.62 (1.06–2.47), p = 0.026) were associated with a higher risk of day-28 mortality while the use of dexamethasone had no impact (aHR 0.81 (0.57–1.15), p = 0.245). The risk of intubation was reduced in the dexamethasone group compared to the usual care group. This result was confirmed after adjustment for respiratory rate, PaO2/FiO2 ratio, and respiratory support at ICU admission (standard oxygen vs high-flow oxygen or non-invasive ventilation) (aHR 0.48 (0.40–0.58), p < 0.001). Ventilator-free days at day-28 were 22 [2;28] in the dexamethasone group and 15 [0;28] in the usual care group (p < 0.001). Patients in the dexamethasone group had a higher risk of ventilator associated pneumonia than those of the usual care group (HR 1.24 (1.00–1.54), p = 0.05), while there was no difference on the risk bloodstream infection (HR 1.23 (0.80–1.88), p = 0.35), fungal infection (OR 0.64 (0.37–1.10), p = 0.11) and gastrointestinal bleeding (OR 1.10 (0.58–2.09), p = 0.77) between the 2 groups.

**Conclusion:** In our study, an early course of dexamethasone in critically-ill Covid-19 patients was not associated with a reduced day-28 mortality, but increased the risk of ventilator associated pneumonia compared to standard of care management. However, an early course of dexamethasone reduced the risk of intubation, increased the number of ventilator-free days at day-28, and was not associated with a higher risk bloodstream infection, fungal infection or gastro-intestinal bleeding.

**Reference 1:** [1] RECOVERY Collaborative Group, Horby P, Lim WS, Emberson JR, Mafham M, Bell JL, et al. Dexamethasone in Hospitalized Patients with Covid-19—Preliminary Report. N Engl J Med. 17 juill 2020.

**Compliance with ethics regulations:** Yes in clinical research.

### FC-166 High dose steroids for persisting ARDS in critically ill COVID-19 patients treated with dexamethasone: a prospective observational multicentre study

#### LOPINTO Julien^1^, ARRESTIER Romain^1^, VOIRIOT Guillaume^3^, URBINA Tomas^2^, LUYT Charles-Edouard^4^, BELLAICHE Raphael^1^, PHAM Taï^6^, AIT-HAMOU Zakaria^7^, ROUX Damien^5^, CLERE-JEHL Raphael^9^, GAUDRY Stéphane^8^, MAYAUX Julien^4^, MEKONTSO DESSAP Armand^1^, DE PROST Nicolas^1^

##### ^1^Hôpital Henri Mondor AP-HP, Créteil, France; ^2^Hôpital Saint Antoine AP-HP, Paris, France; ^3^Hôpital Tenon AP-HP, Paris, France; ^4^Hôpital Pitié Salpétrière AP-HP, Paris, France; ^5^Hôpital Louis Mourier AP-HP, Colombes, France; ^6^Hôpital Bicêtre AP-HP, Le Kremlin-Bicêtre, France; ^7^Hôpital Cochin AP-HP, Paris, France; ^8^Hôpital Avicenne AP-HP, Bobigny, France; ^9^Hôpital Civil de Strasbourg, Starsbourg, France

###### Correspondence: Julien LOPINTO (julien.lopinto@aphp.fr)

*Annals of Intensive Care* 2022, **12(1):**FC-166

**Rationale:** Severe COVID-19 is responsible for acute respiratory distress syndrome (ARDS), requiring the use of invasive mechanical ventilation in 60% of the cases. Short-term corticosteroid therapy (CTC) initiated in the acute phase of the infection reduces the 28-day mortality of patients on oxygen therapy or mechanical ventilation. In some cases, the evolution in intensive care is marked by the persistence of ARDS; the mechanisms generating this unfavorable development involve inflammatory processes and pulmonary fibro-proliferation. The impact of high-dose corticosteroid therapy has already been assessed in the field of persistent ARDS unrelated to SARS-CoV-2 but to date there is not data to assess the impact of such a strategy during severe COVID-19.

**Patients and methods/Materials and methods:** We collected data from the prospective multicenter database ANTICOV involving 11 intensive care unit (ICU) of AP-HP. Eligible patients were admitted in ICU with pneumonia related to COVID 19 and were treated with initial CTC with dexamethasone. We compared patients receiving usual ICU care and patients receiving a secondarily high dose of CTC during ICU care (CTC group).

**Results:** Between September 2020 and March 2021, we enrolled 593 patients. 61 patients (12%) received secondarily high doses of CTC during ICU care. Median delay between dexamethasone and high dose of corticosteroids was 16 days [9–27.5]. There was no between-group difference for median SOFA admission. ICU mortality was higher in the CTC group (48%) as compared with usual care (28%) (p = 0.002). Invasive mechanical ventilation was performed in 359 (67%) patients with usual care and in 47 (77%) patients in CTC group (p = 0.13). The median duration in days of mechanical ventilation and ICU length of stay was higher in the CTC group: respectively 16 [9–28] and 13 [6–27] vs 26.5 [19–40] and 31 [21–45] (both p < 0.001). The proportion of bacterial ventilator associated pneumonia was higher in the CTC group (69%) as compared with usual care (49%) (p = 0.004). Fungal infection was documented in 40 (7.5%) with usual care and in 8 patients (13%) in CTC group (p = 0.132). ICU mortality in CTC group with fungal infection was 87.5%.

**Conclusion:** We found that 12% of patient admitted in ICU for severe COVID 19 received a secondarily high doses of corticosteroids during ICU care. High doses CTC was associated with a higher mortality rate in ICU and higher duration of invasive mechanical ventilation and ICU stay. Propensity score will be made to assess the probability of CTC assignment conditional on observed baselines.

**Compliance with ethics regulations:** Yes in clinical research.

### FC-167 Efficacy of corticosteroids as rescue therapy for prolonged mechanically ventilated-COVID-19 patients with suspected pulmonary fibroproliferation: an observational retrospective study

#### SIBLANI Dima^1^, STEFAN Roxana-Maria^1^, SROUR Claire^1^, PALPACUER Clément^1^, KUTEIFAN Khaldoun^1^, STIEL Laure^1^

##### ^1^GHRMSA - Hôpital Emile Muller, Mulhouse, France

###### Correspondence: Laure STIEL (lau57@hotmail.fr)

*Annals of Intensive Care* 2022, **12(1):**FC-167

**Rationale:** Pathophysiology of coronavirus disease 2019 (COVID-19) suggests a strong role of exaggerated immune response in the evolution to acute respiratory distress syndrome (ARDS). COVID-19 ARDS is characterized by its protracted course, lasting twice as long as ARDS of other origins. Persistent ARDS is characterized by ongoing inflammation, excessive pulmonary fibroproliferation (PFP), prolonged mechanical ventilation, and a substantial mortality. Corticosteroid treatment dampens the hyperinflammatory response and is prescribed as an adjunctive treatment for moderate and severe forms of COVID-19 pneumonia. Only rare data are available for patients with COVID-19 requiring prolonged ventilation. In this retrospective study, we aimed to assess the efficacy of corticosteroids as rescue therapy in patients with persistent COVID-ARDS.

**Patients and methods/Materials and methods:** This observational retrospective study was conducted in one intensive care unit of a French general hospital including mechanically ventilated COVID-ARDS patients admitted between March 2020 and January 2022 who received high-dose of methylprednisolone (1–2 mg/kg/day) for persistent COVID-ARDS after more than ten days of mechanical ventilation. Included patients exhibit at least one out of three criteria for suspicion of PFP changes: low lung compliance, fibrotic changes on Chest CT, or persistent hypoxemia. Endpoints were improvement of lung compliance and hypoxemia.

**Results:** Four hundred forty-three patients with severe COVID pneumonia were admitted to our ICU during the inclusion period. One hundred eighty-seven patients were mechanically ventilated for more than ten days. Nineteen patients (10%) had persistent ARDS and received high-dose methylprednisolone as rescue therapy. Mean age was 68.2 + 6.3 years, median SOFA score at initiation of corticotherapy was 8. Mean delay between intubation and methyprednisolone initiation was 19.1 + 5.6 days. Ten patients (53.7%) survived and were discharged from ICU, and all were still alive at day-90. Lung compliance upon initiation of corticotherapy was similar in patients who survived and those who died (20.1 + 12.5 and 19.9 + 5.6 ml/cmH_2_O respectively). PaO_2_/FiO_2_ ratio was also similar between both groups (152 + 42.6 and 124.2 + 31.7 respectively). Survivors showed significant improvement in both lung compliance (p = 0.0324, Fig. 1A) and PaO_2_/FiO_2_ ratio (p = 0.0352, Fig. 1B).

**Conclusion:** In this cohort of persistent COVID-19 ARDS patients with suspicion of PFP evolution, both lung compliance and PaO_2_/FiO_2_ ratio did significantly ameliorate in patients who survived. Whether this observed improvement in lung mechanics and hypoxemia is due to the late use of steroids, or not, has to be confirmed.

**Compliance with ethics regulations:** Yes in clinical research.
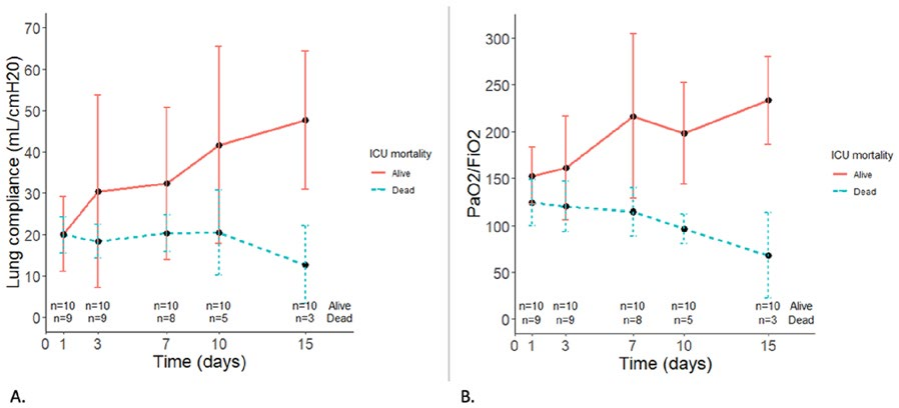


*Evolution of lung compliance and PaO*_*2*_ */FiO*_*2*_ *ratio after initiation of corticotherapy*

### FC-168 Comparison between different doses of dexamethasone and its outcome on critically ill COVID-19 patients

#### ALILA Ilef^1^, HADDED Amina^1^, JERBI Salma^1^, KHARRAT Sana^1^, BOUBTANE Rihab ^1^, BAHLOUL Mabrouk^1^, BOUAZIZ Mounir^1^

##### ^1^Hôpital Habib Bourguiba Sfax, Sfax, Tunisie

###### Correspondence: Ilef ALILA (ilefalila1323@gmail.com)

*Annals of Intensive Care* 2022, **12(1):**FC-168

**Rationale:** The coronavirus disease 2019 significantly impacted human society. Recently, the synthetic pure glucocorticoid dexamethasone was identified as an effective compound for treatment of severe COVID-19 (1). The aim of this study was to compare the different doses of dexamethasone prescribed and their impact on prediction of outcome in critically ill COVID-19 patients.

**Patients and methods/Materials and methods:** We conducted a retrospective study in a medical ICU over a period of 16 months (September 2020–December 2021) including patients with SARS-COV2 infection. Dexamethasone-based corticosteroid therapy was introduced from day one in patients with acute distress respiratory syndrome (ARDS) with different doses: ≤ 8 mg/day, 12 mg/day and 24 mg/day.

**Results:** During the study period, 430 patients were included with a mean age of 58.7 ± 15.9 and a gender ratio of 1.5. The median SAPSII and SOFA score were respectively of 28 ± 15 and 4 ± 2.6. Most patients had comorbidities, including hypertension (35.8%), obesity (31.6%) and diabetes (36.7%). At admission, 301 patients had severe ARDS (70%). Dexamethasone was prescribed with 12 mg/day in 342 patients (79.5%), with ≤ 8 mg/day in 51 patients (11.9%) and 24 mg/day in 37 patients (8.6%). The use of invasive mechanical ventilation was more frequent in patients who received 12 mg/day (46%; p = 0.035). The occurrence of infection during hospitalization was high in the same group of patients (46%; p = 0.014). There was no significant difference between the three doses of dexamethasone in terms of diabetic decompensation, hypokalemia or mortality (Table I).

**Conclusion:** This study showed that the use of different doses of dexamethasone was not associated with different outcome in critically ill COVID-19 patients admitted in ICU.

**Reference 1:** (1) Tomoshige Kino, Irina Burd, James H. Segars: Dexamethasone for severe COVID-19: how does it work at cellular and molecular levels?

**Compliance with ethics regulations:** Yes in clinical research.
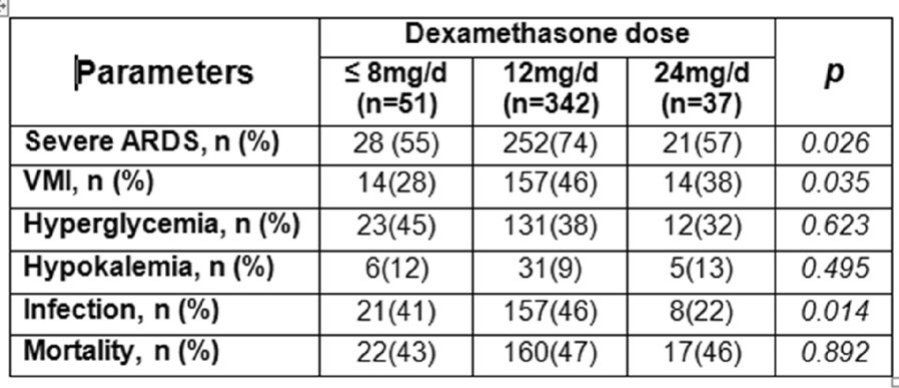


*Table 1: outcome comparison according to dexamethasone dose*

### FC-169 Delirium in critically ill patients: a prospective study

#### SANA Khedher^1^, HASSAN Majid Al Dossari^1^

##### ^1^AFH Wady addawassir, Riadh, Arabie Saoudite

###### Correspondence: Khedher SANA (sanakhedher@hotmail.fr)

*Annals of Intensive Care* 2022, **12(1):**FC-169

**Rationale:** Delirium is among the most common problem in the intensive care unit (ICU). It is often under diagnosed and it has a considerable impact on morbidity and mortality rate. We aimed to determine the incidence, risk factors and outcomes of delirium in the ICU.

**Patients and methods/Materials and methods:** We conducted a retrospective study including critically-ill patients fulfilling the criteria for admission to the ICU between January 2022 and September 2022. Delirium was assessed during each shift (morning and evening), by trained nurses, using the Confusion Assessment Method for the ICU (CAM-ICU). SOFA and RASS scores were calculated on admission. Demographics and clinical status were also collected based on the medical record.

**Results:** One hundred twenty patients were included. Median age was 75 ± 13 years with a sex ratio of 1.2. Fourty-two developed delirium (incidence: 35%); 20% were hypoactive, 5% mixed and 10% were hyperactive in type. They were older (70 versus 55) and had a significantly higher SOFA score at admission (8[8–10] versus 3 [2–4], p < 0.001), RASS (2.8 versus 1.3, p < 0.001). Cumulative doses of midazolam (80 mg versus 48 mg, p < 0.001). The presence of visual or auditory deficits were reported at baseline in 27.7% of patients with delirium. They had also longer ICU length-of-stay (6 vs 14, p < 0.001) and mortality was significantly higher in the delirium group (44.4% versus 28%).

**Conclusion:** The incidence of delirium in our ICU is 35%, it was associated with a twofold increase in the risk of death and prolongs the length of hospitalization. Our results were an opportunity to implement a strategy for assessment and surveillance and prevention of delirium to improve the care in ICU.

**Compliance with ethics regulations:** Yes in clinical research.

### FC-170 Mortality predictors of snake bite envenomation in southern Tunisia

#### BRADAI Sabrine^1^, GHORBEL Rezk^1^, TURKI Olfa^1^, CHTARA Kamilia^1^, BAHLOUL Mabrouk^1^, BEN HAMIDA Chokri^1^, BOUAZIZ Mounir^1^

##### ^1^Habib Bourguiba University Hospital, Sfax, Tunisia, Sfax, Tunisie

###### Correspondence: Sabrine BRADAI (sabrine.bradai2@gmail.com)

*Annals of Intensive Care* 2022, **12(1):**FC-170

**Rationale:** Snakebite envenoming is a neglected tropical disease that kills more than 100.000 people every year. In Tunisia, it is particularly frequent in the sub-Saharan regions with high mortality and morbidity. Therefore, we aim to identify the predictor factors of mortality in snakebite envenomation in our region.

**Patients and methods/Materials and methods:** It is a retrospective descriptive study, conducted at the intensive care unit (ICU) of Habib Bourguiba University Hospital, Sfax, Tunisia, over a period of 16 years (from January 01, 2006, to December 31, 2021). Snakebite envenomation was defined by a history of snakebite. Survivor and non-survivor subgroups were compared to identify predictors of mortality.

**Results:** Out of the 19 included patients, 4 patients died. The mortality rate was 21.1%. Univariate analysis revealed that at ICU admission: high SOFA (p = 0.017), high shock index (p = 0.024), high value of leucocytes (p = 0.001), high value of blood urea (p = 0.003) and the presence of clinical signs of shock (p = 0.037), are associated with mortality.

**Conclusion:** Early evaluation of severity at admission is crucial in the management of snakebite envenomation. Further multicentric studies are required to approve a severity scale proper to snakebite envenomation in Tunisia.

**Reference 1:** World Health Organization. Venomous snakes distribution and species risk categories. 2017. [Online] Available from: https://apps.who.int/bloodproducts/snakeantivenoms/database/default.html.

**Reference 2:** Chakroun-Walha O, Issaoui F, Nasri A, Bradai H, Farroukh A, Karray R, et al. Early severity predictors of snakebite envenomation in the southern region of Tunisia: a multivariate analysis. J Acute Dis. 2021;10(2):71.

**Compliance with ethics regulations:** Yes in clinical research.
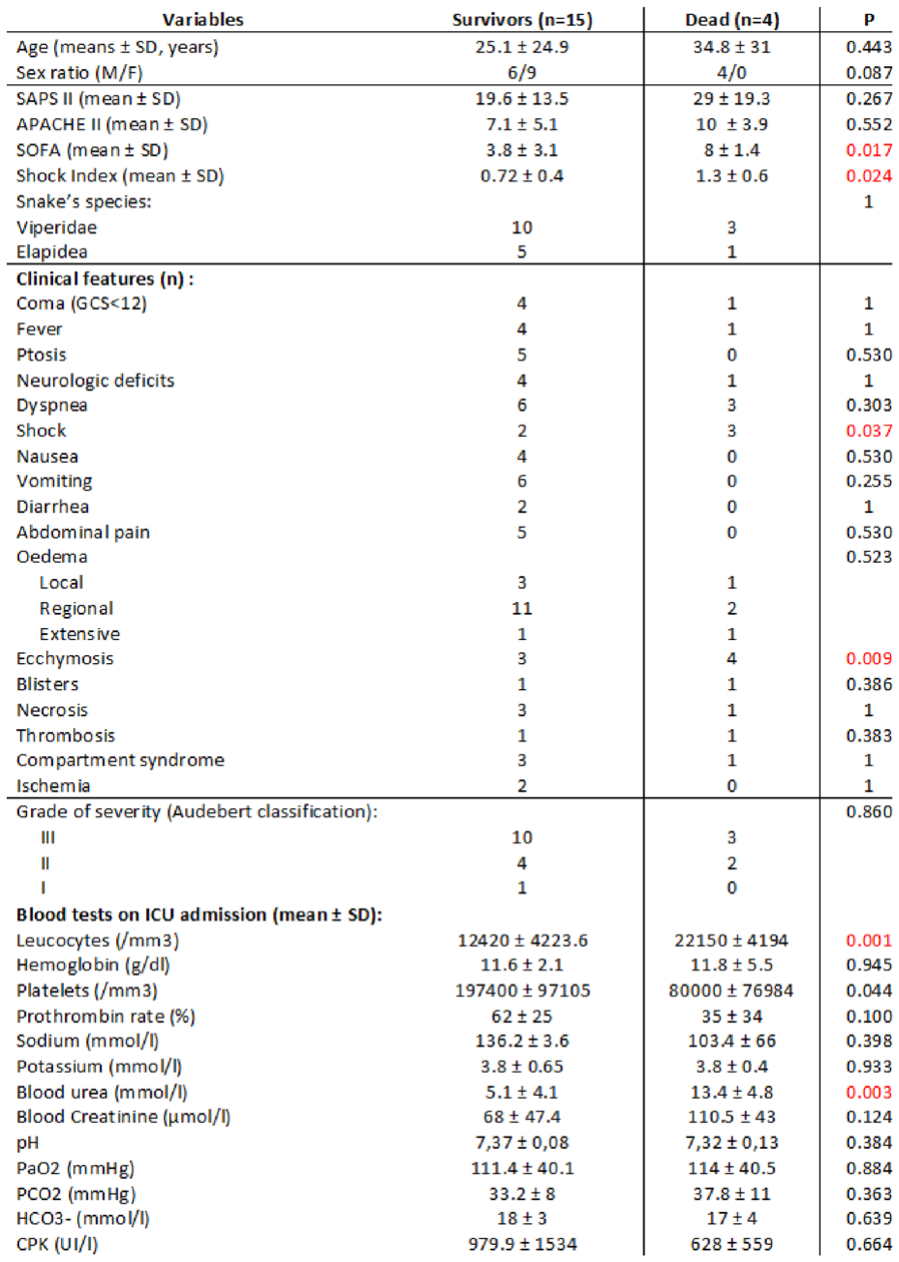


*Table 1: Univariate analysis of predictors of mortality in snake envenomation at admission in ICU.*

### FC-171 Acquired agitation in ARDS with Covid-19 compared to influenza patients: a propensity score matching observational study

#### MAAMAR Adel^1^, LIARD Clémence^1^, REIZINE Florian^1^, PAINVIN Benoit^1^, DELAMAIRE Flora^1^, COIRIER Valentin^1^, QUELVEN Quentin^1^, GUILLOT Pauline^1^, LESOUHAITIER Mathieu^1^, TADIÉ Jean-Mac^1^, GACOUIN Arnaud^1^

##### ^1^CHU de Rennes, Hôpital Pontchaillou, Rennes, France.

###### Correspondence: Adel MAAMAR (adel.maamar@chu-rennes.fr)

*Annals of Intensive Care* 2022, **12(1):**FC-171

**Rationale:** A growing body of evidence reports that agitation and encephalopathy are frequent in critically ill Covid-19 patients. Our aim was to assess the incidence and risk factors of agitation in critically-ill ARDS patients with Covid-19, and compare these findings to a population of patients with ARDS patients with influenza.

**Patients and methods/Materials and methods:** We included all patients with laboratory-confirmed Covid-19 infection and ARDS admitted to our medical intensive care unit (ICU) between March 10th, 2020 and April 16th, 2021, and all the patients with laboratory-confirmed influenza infection and mechanical ventilation (MV) admitted to our ICU between April 10th, 2006 and February 8th, 2020. Clinical and biological were retrospectively collected. We also recorded previously known factors associated with agitation (ICU length of stay, length of invasive ventilation, SOFA score and SAPS II at admission, sedative and opioids consumption, time to defecation). Agitation was defined as a day with Richmond Agitation Sedation Scale greater than 0 after exclusion of other causes of delirium and pain. We compared the prevalence of agitation among Covid-19 patients during their ICU stay and in those with influenza patients. To improve the balance of baseline characteristics and reduce the effects of selection bias and potential confounding factors in this observational study, a propensity score analysis was performed. Second, we used a Cox-proportional hazard model to determine whether agitation during the ICU stay was independently associated with mortality at day-28.

**Results:** We included 241 patients (median age 62 years [53–70], 158 males (65.5%)), including 146 patients with Covid-19 and 95 patients with Influenza. One hundred eleven (46.1%) patients had agitation during their ICU stay. Patients with Covid-19 had significantly more agitation than patients with influenza (respectively 80 patients (54.8%) and 31 patients (32.6%), p < 0.01). After matching with a propensity score, Covid-19 patients remained more agitated than influenza patients (49 (51.6% vs 32 (33.7%), p = 0.006). Agitation remained independently associated with mortality after adjustment for other factors (HR = 1.85, 95% CI 1.37–2.49, p < 0.001).

**Conclusion:** Agitation in ARDS Covid-19 patients is more frequent than in ARDS influenza patients. The agitation was not associated with common risk factors, such as severity of illness or sedation. These findings suggest that SARS-CoV-2 is directly and indirectly involved in agitation and should probably be acknowledged as a risk factor. As agitation can be one of the presentations of a delirium, its presence should alert us to the risk of encephalopathy.

**Compliance with ethics regulations:** Yes in clinical research.
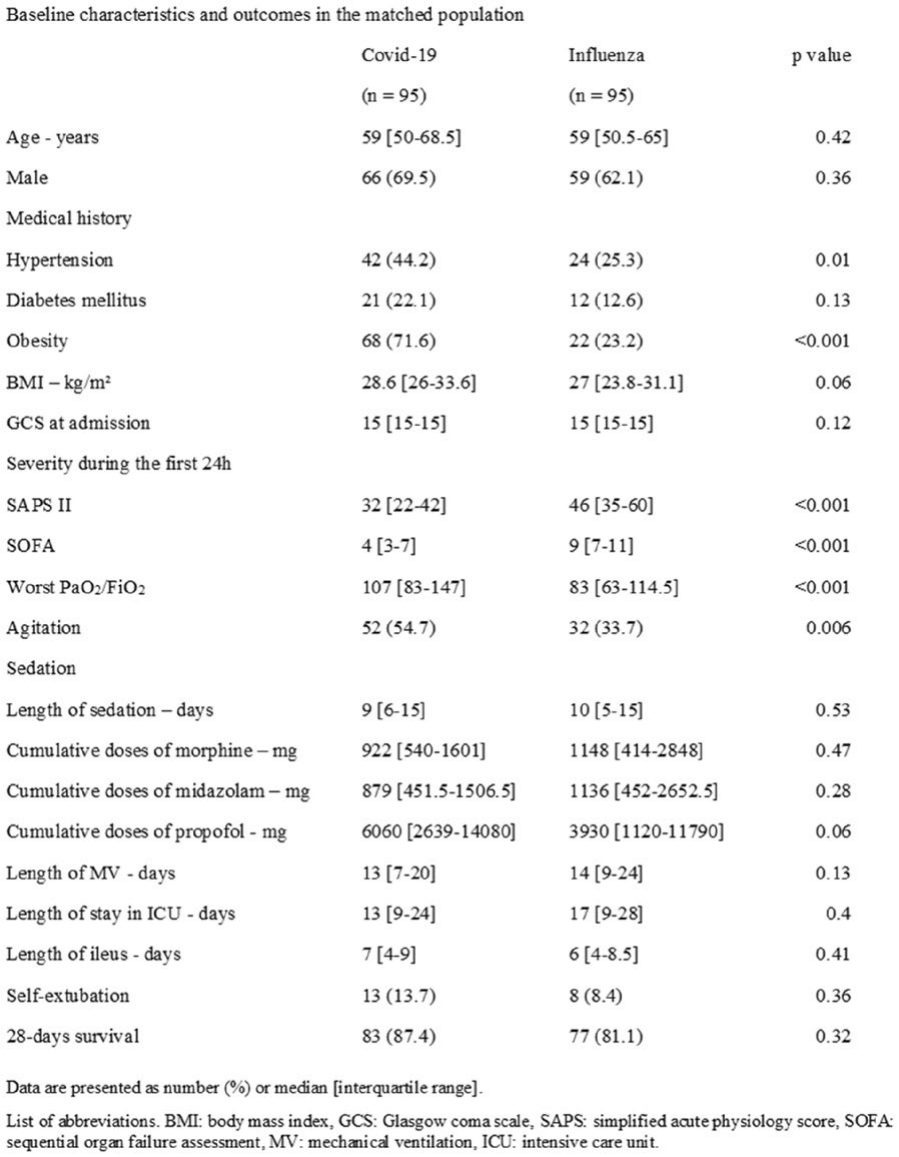


*Baseline characteristics and outcomes in the matched population*

### FC-172 Neuro-psychiatric complications of COVID-19 in intensive care unit

#### ESSAFI Fatma^1,2^, JAAFAR Nour Zayneb^1,2^, KHARRAT Malek^1,2^, BEN ISMAIL Khaoula^1,2^, TALIK Imen^1,2^, KADDOUR Moez^1,2^, MERHABENE Takoua^1,2^

##### ^1^Intensive Care Unit, Regional Zaghouan’s Hospital, Zaghouan, Tunisie; ^2^Faculty of Medicine of Tunis, Tunis El-Manar university, Tunis, Tunisie.

###### Correspondence: Fatma ESSAFI (fatma.essafi@fmt.utm.tn)

*Annals of Intensive Care* 2022, **12(1):**FC-172

**Rationale:** Several studies have reported association between COVID-19 infection and a plethory of neuropsychiatric complications whereas the neuropsychiatric damage is still contested. We aimed to describe the epidemiology of neuropsychiatric manifestations in patients hospitalized for confirmed severe COVID-19 pneumonia and determine its impact on patient’s management and outcome.

**Patients and methods/Materials and methods:** All data of confirmed COVID-19 patients hospitalized in Zaghouan’s Hospital ICU between March 2020 and January 2022 were retrospectively analyzed. We collected epidemiological, clinical, prescribed medications and outcomes. Factors associated with neuropsychiatric complications and its impact on mortality were evaluated through binary logistic regression models, by comparing two groups: patients with or without neuropsychiatric manifestations.

**Results:** Of the total of 365 patients included, 201 have developed neurologic and/or psychiatric manifestations during their hospital stay (55%). Median age was 58 [17–89] with sex ratio 1.3. Median SAPS II and APACHE II scores were 26 ± 8.3 and 7 ± 4.9 respectively. Fifteen patients had underlying psychiatric disease history and two had a history of ischemic stroke without sequelae. Neurologic disorders were described in 125 patients. It consisted on 118 episodes of headache, 6 ischemic strokes and 2 cases of encephalitis, one of which was symptomatic of status epilepticus; no peripheral nervous system involvement was reported. Psychiatric disturbance was seen in 121 patients. The most common manifestations were: an unexpected agitation in 79 patients, sleep disturbances in 25 patients, depressed mood in 20 patients, anxiety in 8 patients, delirium in 3 cases and confusion in 2 cases. Depression was manifested by reduced appetite and anorexia, tiredness and lack of energy and irritability. Management of patients consisted in majority of cases in long-acting benzodiazepine prescription alone or associated to neuroleptic agents. No differences were noted in demographic characteristics between patients who developed neuropsychiatric signs and the others. But active smoking was associated with the presence of neuropsychiatric signs (44.7% vs 31.7%, p = 0.034). At admission, the 2 groups had comparable respiratory exchange and laboratory findings. Presence of psychiatric signs was significantly associated with more use of invasive mechanical ventilation (49% vs 24.3%, p < 0.001), longer length of ICU stay (11 vs 7 days, p < 0.001) and higher ICU mortality (60% vs 33%, p < 0.001). Headache, the most common neurologic manifestation, alone was not associated with worse prognosis.

**Conclusion:** Neuroinflammation associated to SARS-CoV-2 infection could be responsible for a wide and still unknown spectrum of neuropsychiatric disorders which are associated with poor prognosis and high mortality. Long term follow up is recommended to detect disability consequences of neuro-COVID.

**Compliance with ethics regulations:** Yes in clinical research.

### FC-173 Incidence and severity of the post-intensive care syndrome at 1 year after a long hospitalization in ICU for a COVID-19-related ARDS

#### TCHOUBOU Tona^1^, MORICHAU-BEAUCHANT Tristan^2^, SAINT-JOUAN Mélanie ^2^, DAVIAUD Fabrice^2^, FICHET Jerôme^2^, THIERRY Stéphane^2^, NAHUM Julien^2^, MAILLET Jean-Michel^2^

##### ^1^Hôpital Delafontaine, Saint-Denis, France; ^2^Centre Cardiologique du Nord, Saint-Denis, France

###### Correspondence: Tona TCHOUBOU (ttchoubou@gmail.com)

*Annals of Intensive Care* 2022, **12(1):**FC-173

**Rationale:** Post-Intensive Care Syndrome (PICS) is a frequent condition in ICU-survivors including physical, cognitive and psychological disorders. The long-term consequences of a hospitalization in intensive care for severe COVID-19 are unknown. The aim of this study was to assess the incidence and severity of cognitive and psychological disorders, the sequelae of hospitalization and their impact on the health-related quality of life of patients who survived at one year after a prolonged hospitalization in intensive care for COVID-19-related ARDS.

**Patients and methods/Materials and methods:** Prospective, descriptive, monocentric cohort study, carried out in the Seine-Saint-Denis department in France between May and July 2021. Patients who received mechanical ventilation of at least 72 h during a stay in intensive care of at least 7 days for COVID-19-related ARDS were included.

**Results:** Thirty out of thirty-six eligible patients were assessed at a median time of 423 days since infection (IQR [404–437]). The patients’ quality of life was impaired mainly in the physical domain. Sixteen percent of patients complained of asthenia and 23% reported muscle weakness. Fifty-three percent of patients complained of cognitive impairment and 36% of patients had symptoms of post-traumatic stress disorder. Symptoms of anxiety were found in 40% and symptoms of depression in 16% of patients. Seventy percent of patients had at least one skin, neurological or joint sequelae of hospitalization. Twelve patients (40%) had persistent dyspnea.

**Discussion:** This study has several limitations. It is descriptive, monocentric and the study population is quite small. Yet, to this day, the data concerning the sequelae of severe COVID-19 especially in the critically ill remain scarce. Since the “first wave” of the pandemics, the management of COVID-19 ARDS changed and effective vaccines have been found. Further studies are mandatory to assess the effect of these findings on the prevalence and severity of the PICS.

**Conclusion:** The sequelae of a hospitalization in intensive care for a COVID-19-related ARDS are common and impair survivors' health-related quality of life at one year after infection. This study among others will help to know better the long-term consequences of the COVID-19 pandemics in terms of PICS.

**Compliance with ethics regulations:** Yes in clinical research.

### FC-174 Management of hemorrhagic stroke in neurocritical care ICU

#### ZEGHDOUD Dalila^1^, BOUGDAL Dalila^1^, SADAT Souhila^1^

##### ^1^EHS SALIM ZEMIRLI EL HARRACH, Alger, Algérie

###### Correspondence: Dalila ZEGHDOUD (dalila-z2011@hotmail.fr)

*Annals of Intensive Care* 2022, **12(1):**FC-174

**Rationale:** Hemorrhagic strokes represent 10 to 15% of all strokes. The incidence increases with age, especially beyond 55 years. Intracerebral hemorrhage is a serious pathology whose mortality is between 30 to 60% at 30 days. Half of the deaths occur within the first 48 h. The management of intracerebral hemorrhage in neurocritical care units has reduced this morbidity and mortality. The objective of our study is to study the impact of the management of hemorrhagic stroke in a neurocritical care unit.

**Patients and Methods/Materials and methods:** This is a prospective, monocentric study from January 2010 to October 2019. The study involved patients admitted for hemorrhagic stroke, confirmed by brain CT. Patients with traumatic brain hematomas, hemorrhagic changes in ischemic stroke, and cerebro-meningeal hemorrhage were excluded from the study. Demographic data, history, Glasgow score, CT data, initial medical care and short-term change were studied. The surgical indication was at the discretion of the surgeon.

**Results:** During the study period 120 patients with an average age of 60 years were included, with a sex ratio of 1.1. 55% of patients were hypertensive, 13% diabetic, of which 14% were on AVK and 26% on platelet aggregation inhibitors. No risk factors were found in 32% of patients. The average Glasgow admission score was 8. At imaging, 60% had a deep localization of the hematoma and 54% a hydrocephalus. Medical management was based on monitoring of blood pressure figures in 58%, the use of mechanical ventilation in all patients, osmotherapy in 22% and reversion of anticoagulant therapy in 6% of cases. Surgery was indicated in 60% of cases: An external ventricular shunt in 70% of cases and a decompression and evacuation of the cerebral hematoma in 40% of cases. Overall mortality was 70%.

**Conclusion:** In terms of public health, stroke is a medical, social and economic scourge. It has serious life-threatening and functional consequences: just under 75% of people with intracerebral hemorrhage die within a year, and more than half of stroke survivors have significant consequences.

**Compliance with ethics regulations:** N/A.

### FC-175 Scorpion envenomation in the emergency department: a prospective study of predictive factors of severity

#### KARRAY Rim^1^, MALLEK Mariem^1^, BEN ALI Hana^1^, ZOUARI Ala^1^, BEN SALEM Imen^1^, GORBEL Rizk^1^, CHAKROUN-WALHA Olfa^1^, REKIK Noureddine^1^

##### ^1^CHU Habib Bourguiba, Sfax, Tunisie

###### Correspondence: Hana BEN ALI (Dr.hanabenali@gmail.com)

*Annals of Intensive Care* 2022, **12(1):**FC-175

**Rationale:** Scorpion envenomation is common in the tropical and subtropical regions. It poses a major public health problem with some patients having serious clinical manifestations and severe complications including death. The aim of our study was to describe the predictive factors of severity in the management of scorpion envenomation patients admitted in the acute care unit.

**Patients and methods/Materials and methods:** A 6 months prospective analytic observational single-center study including patients admitted for stage II or III of scorpion envenomation. Stage I scorpion envenomation were excluded. Statistical analysis was performed using the statistic software SPSS 20.

**Results:** Sixty-six patients were included. Mean age was 29 ± 20 years (1 to 84 years old) with female predominance (sex-ratio = 0.8). The majority of patients (90%) were staged II according to severity. Among demographic factors, age (p = 0.007) and the mode of transport in the acute care unit (p = 0.001) were predictor factor of mortality. However, comorbidities and gender had no prognostic impact. Clinical predictor factor of mortality on admission were: agitation (p < 0.001), dyspnea (p = 0.02), abdominal pain (p = 0.03), rapid breathing (p = 0.01), oxygen saturation (p = 0.04), priapism (p = 0.01) and the presence of signs of shock (p < 0.001): marbling, cool skin. Leukocyte, glucose level and troponin level increased significantly with the severity of envenomation and are correlated with high mortality: p = 0.001. Acidosis (p = 0.026), hypoxemia (p = 0.04) and anemia (p = 0.008) were also predictive factors of mortality The presence of abnormalities on electrocardiogram (p = 0.005), chest X-ray (p = 0.03) and cardiac ultrasound (p < 0.001) were associated with higher mortality. Finally, a significant association was found between cardiogenic shock requiring the use of dobutamine and death rate (p < 0.001).

**Conclusion:** Scorpion envenomation is an accidental pathology that continues to be serious in our country specially in summer. The predictive factors of mortality seem similar to those described in the literature. However, new diagnostic tools may be helpful in the early detection of patients with significant risk of death.

**Compliance with ethics regulations:** Yes in clinical research.

### FC-176 Impact of intensive physical therapy on patients with neuromyopathy in a weaning unit after prolonged stay in ICU

#### PERETOUT Jean-Baptiste^1^, COTTIAS Josépine^1^, FRANTZ Camille^1^, DUARTE Amaury^1^, DIAZ-LOPEZ Carlos^1^, CHOUKROUN Gérald^1^

##### ^1^Hôpital Forcilles - Fondation Cognacq-Jay, Férolles-Attilly, France.

###### Correspondence: Jean-Baptiste PERETOUT (jperetout@cognacq-jay.fr)

*Annals of Intensive Care* 2022, **12(1):**FC-176

**Rationale:** Neuromyopathy is a severe but common complication in the ICU, particularly for patients with prolonged stays^1^. Intensive physical therapy with a muscle strengthening program and a daily schedule allows a faster recovery. The objective of this study is to determine the impact of intensive physical therapy on the functional recovery of these patients in a weaning unit.

**Patients and methods/Materials and methods:** This is a retrospective, descriptive study in a respiratory weaning unit over the year 2021. All patients hospitalized in the weaning unit were included. The MRC score at admission was used to determine the diagnosis and severity of neuromyopathy and was compared with the same score at discharge. The prevalence of neuromyopathy at admission was also investigated. Demographic and physical therapy characteristics were collected. All inpatients with complete data were analyzed.

**Results:** In 2021, 144 patients were admitted. A total of 134 patients were analyzed with male 70%, age 63 ± 12 years, COVID-19 70%. The MRC score was statistically higher at discharge than at admission: 52.3 ± 11.4 vs 39.5 ± 14 (p < 0.001). The prevalence of neuromyopathy at admission was 73% (n = 98). During the hospitalization, delivered rehabilitation treatment were: sitting on the edge of the bed in 93%, reverticalization in 82% and walking in 57%. The different techniques are presented in Table I.

**Conclusion:** Intensive physical therapy is associated with an improvement in the MRC score for patients during their stay in the weaning unit. Multidisciplinary management also contributes to improve the different functional aspects of the patients.

**Reference 1:** De Jonghe B, Lacherade JC, Durand MC, Sharshar T. Critical illness neuromuscular syndromes. Crit Care Clin. 2007 Jan;23(1):55–69.

**Compliance with ethics regulations:** Yes in clinical research.
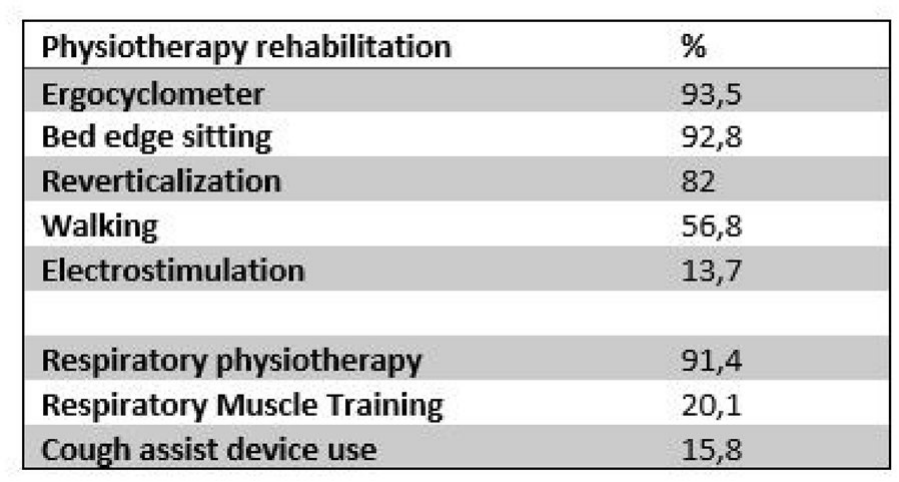


*Table I Physiotherapy rehabilitation techniques in a respiratory weaning unit.*

### FC-177 Breast cancer in the ICU: outcomes and prognosis factors

#### VIGNERON Clara^1^, CHARPENTIER Julien^1^, COUSSY Florence^2^, ALEXANDRE Jérôme^1^, PÈNE Frédéric^1^, JAMME Matthieu^3^

##### ^1^Hôpital Cochin, Paris, France; ^2^Institut Curie, Paris, France; ^3^Hôpital Privé de l’Ouest Parisien, Ramsay Générale de Santé, Trappes, France

###### Correspondence: Clara VIGNERON (claravigneron@hotmail.fr)

*Annals of Intensive Care* 2022, **12(1):**FC-177

**Rationale:** Breast cancer has surpassed lung cancer as the most frequently diagnosed cancer with an estimated 2.3 million new cases in 2020. One in eight to ten women will have breast cancer during their lifetime. Compared with other solid cancers, its prognosis is usually seen as encouraging even at a metastatic stage. Surprisingly, characteristics of breast cancer patients hospitalized in the ICU have seldom been described and especially whether this common pathology affects admission patterns and prognosis in critically-ill patients.

**Patients and methods/Materials and methods:** We conducted a retrospective monocenter study including patients with breast cancer requiring unplanned admission to a medical ICU over a 14-year period (2007–2020). Non-inclusion criteria were the following: admission to secure a procedure, planned admissions following elective surgery and patients with cancer cured for more than 5 years. Independent predictors of in-ICU and 1-year outcome (available for n = 170 (96%) patients) were addressed by a cause-specific multivariate Cox regression analysis.

**Results:** One hundred seventy-seven patients (median age = 65 [57–75] years) were analyzed. Breast cancer was at a metastatic stage for 122 (68.9%) patients and recently diagnosed or in progression under treatment in respectively 25 (14.1%) and 76 (42.9%) patients. Median SOFA score at admission was 5 [4–8] and patients required invasive mechanical ventilation, vasopressors/inotropic agents and renal replacement therapy in respectively 72 (40.7%), 57 (32.2%) and 26 (14.7%) cases. Admissions were related to sepsis in 56 (31.6%) patients, to iatrogenic/procedural complication in 19 (10.7%) patients and to specific (i.e. directly linked to the underlying malignant disease) complication in 47 (26.6%) patients. ICU, hospital and one-year mortality rates were respectively 20.9%, 30.5% and 57.1%. In multivariate analysis, factors associated with ICU-mortality were invasive mechanical ventilation (Cause specific hazard (CSH) 2.53 [1.11–5.75], p = 0.03) and poor performance status (at 3 or 4) (CSH 2.37 [1.18–4.80], p = 0.02). One-year mortality in ICU-survivors was independently associated with the occurrence of specific complications during ICU stay (CSH 2.20 [1.31–3.71], p = 0.003), a triple negative cancer status (CSH 1.97 [1.06–3.67], p = 0.03) and poor performance status (CSH 1.51 [1.19–1.92], p < 0.001).

**Conclusion:** One in four breast cancer patients are admitted to the ICU for life-threatening specific complications directly linked to their underlying malignant disease. The in-ICU prognosis is encouraging and not related to oncologic characteristics supporting broad-admission policy in this population. Long-term prognosis is poor, especially for those patients with advanced malignancy.

**Compliance with ethics regulations:** Yes in clinical research.

### FC-178 Outcome of patients with solid tumors considered for ICU admission: a single-center experience

#### BENGUERFI Soraya^1^, HIRSINGER Baptiste^1^, VALENTINE Colin De Verdière^1^, RAIMBOURG Judith^2,3^, SENELLART Hélène^2^, VIALA Caroline^4^, SEGUIN Amélie^1^, DECAMPS Paul^1^, DESMEDT Luc^1^, REIGNIER Jean^1^, CANET Emmanuel^1^

##### ^1^Médecine Intensive Réanimation, CHU de Nantes, Nantes, France; ^2^Département d'Oncologie médicale, Institut de cancérologie de l'Ouest, Saint-Herblain, France; ^3^Nantes Université, INSERM, CNRS, Université d’Angers, CRCI2NA, Nantes, France; ^4^Service d'Oncologie médicale, CHU de Nantes, Nantes, France

###### Correspondence: Soraya BENGUERFI (soraya.benguerfi@gmail.com)

*Annals of Intensive Care* 2022, **12(1):**FC-178.

**Rationale:** Patients with solid tumors are increasingly considered for ICU admission. Our purpose was to evaluate the outcome of patients with solid tumors considered for ICU admission and to assess the performance of physiological scores at the time of triage for predicting hospital mortality.

**Patients and methods/Materials and methods:** Prospective cohort study including all patients with solid tumors for whom ICU admission was requested between July 2019 and December 2021 in a French University-affiliated Hospital.

**Results:** Of the 6,262 patients considered for ICU admission, 410 patients (6.5%) had solid tumors. Median age was 64 and 137 patients (33.4%) were women. At the time of triage, 206 patients (50.8%) had sepsis. Of the 410 patients evaluated, 176 patients (42.9%) were ultimately admitted. Diagnoses of hemoptysis (odds ratio 10.35; 95% CI 2.80–66.89; p 0.006) and pneumothorax (odds ratio 17.94; 95% CI 3.48–328.64; p 0.002) were independently associated with ICU admission, whereas older age (odds ratio 0.98, 95% CI 0.96–0.99; p 0.004) was independently associated with ICU refusal. Of the 234 patients (57.1%) who were denied ICU admission, 61 patients (26.1%) were deemed too well to benefit from ICU, and 173 (73.9%) were deemed too sick to benefit from ICU. ICU and 6-months survival rates of patients admitted to the ICU were 80.1% and 58.0%, respectively. In patients not admitted in ICU because considered too well, hospital survival was 86.9% and overall survival at 6 months was 67.2%. In patients not admitted in ICU because considered too sick, hospital survival was 37.0% and overall survival at 6 months was 19.7%. qSOFA, NEWS score and Shock index at the time of triage were similar between patients admitted and not admitted to the ICU and demonstrated poor performances for predicting hospital mortality (AUC 0.52, 0.62 and 0.51, respectively).

**Conclusion:** In our experience, patients with solid tumors had a high rate of treatment limitations taken at the time of triage. Hospital survival of patients admitted to the ICU was encouraging. However, both the significant rates of hospital mortality in patients considered too well to benefit from ICU admission, and the survival rates of patients considered too sick underline the limits of clinical judgment at the time of triage. Unfortunately, physiological scores at the time of triage demonstrated poor performances for predicting hospital mortality.

**Compliance with ethics regulations:** Yes in clinical research.

### FC-179 Mechanisms, management and prognosis of severe gastrointestinal hemorrhage in patients with pancreatic cancer: a retrospective multicenter study

#### PICARD Benjamin^1^, WEISS Emmanuel^2,3^, CALIEZ Olivier^4^, GOURY Antoine^5^, BONNY Vincent^6^, KEMOUN Gabriel^1^, RUDLER Marika^4,7^, RHAIEM Rami^8^, BACHET Jean Baptiste^4,7^, RONOT Maxime^3,9^, MAYAUX Julien^1^, DEMOULE Alexandre^1,10^, DECAVÈLE Maxens^1,10^

##### ^1^AP-HP.Sorbonne Université - Service de Médecine Intensive et Réanimation - Département R3S - Hôpital de la Pitié Salpêtrière, Paris, France; ^2^AP-HP.Nord - Université de Paris - Département d’Anesthésie-Réanimation - Hôpital Beaujon, Clichy, France; ^3^Université de Paris - UMRS1149 - Centre de recherche sur l’inflammation, Paris, France; ^4^AP-HP.Sorbonne Université - Service d’Hépato-Gastro-Entérologie - Hôpital de la Pitié Salpêtrière, Paris, France; ^5^CHU de Reims - Unité de Médecine Intensive et Réanimation Polyvalente - Hôpital Robert Debré, Reims, France; ^6^APHP-6 Sorbonne Université - Service de Médecine Intensive et Réanimation - Hôpital Saint-Antoine,, Paris, France; ^7^Sorbonne Université - INSERM Centre de Recherche Saint-Antoine (CRSA) - Institute of Cardiometabolism and Nutrition (ICAN), Paris, France; ^8^CHU de Reims - Service de Chirurgie hépatobiliaire, pancréatique et oncologique digestive - Hôpital Robert Debré, Reims, France; ^9^AP-HP.Nord - Université de Paris - Département de Radiologie - Hôpital Beaujon, Clichy, France; ^10^Sorbonne Université - INSERM UMRS1158 Neurophysiologie Respiratoire Expérimentale et Clinique, Paris, France.

###### Correspondence: Benjamin PICARD (benjamin.picard@aphp.fr)

*Annals of Intensive Care* 2022, **12(1):**FC-179

**Rationale:** Gastrointestinal bleeding (GIB) is a frequent and potentially life-threatening complication in patients with pancreatic cancer. Although GIB represents the second reason for intensive care unit (ICU) admission in pancreatic cancer patients, only limited data are available regarding bleeding mechanisms, management and outcomes in these patients. Primary aim was to identify factors associated with a successful hemostatic intervention (i.e. gastrointestinal endoscopy and/or arterial embolization). Secondary aim was to identify factors associated with ICU mortality.

**Patients and methods/Materials and methods:** Retrospective multicenter cohort study in five French ICUs. All consecutive patients with pancreatic cancer, admitted to the ICU for GIB between January 2009 and January 2020 were screened for inclusion. Patients with a recent pancreatic surgery (< 4 weeks) were excluded.

**Results:** Seventy-eight patients were included (48 (62%) males, 67 [56–73] median [interquartile interval] years-old). Tumors were mainly adenocarcinoma (91%) in cephalic position (74%). Prior to ICU admission, 81% of patients received chemotherapy and 31% underwent pancreatic resection surgery. Cancer progression was identified in 51% of cases. GIB mechanisms were gastroduodenal tumor infiltration (35%), gastroesophageal varices (21%) mostly related to left-sided portal hypertension, arterial aneurysm (14%), non-tumoral ulcer (11%) and miscellaneous (19%). Arterial aneurysms were more frequently detected in patients who underwent pancreatic resection (41%) than their counterpart (2%), p < 0.001. During the ICU stay, 41 (53%) patients presented hemorrhagic shock, 60 (77%) received red blood cells transfusion (median number of red blood cells units 3 [2–5]), and 32 (41%) required mechanical ventilation. Sixty-two (80%) patients and 21 (27%) patients had at least one upper endoscopy and one arterial embolization, respectively. A successful hemostasis was achieved in 35 (45%) patients. By multivariate analysis, three independent variables were significantly associated with hemostatic interventions success: aneurysmal bleeding OR 32.76, p = 0.005; ongoing chemotherapy at ICU admission OR 0.13, p = 0.013 and prothrombin time ratio (PT) OR 1.05, p = 0.003. ICU mortality rate was 20%. By multivariate analysis, four independent variables were significantly associated with ICU mortality: performance status > 2 odds ratio (OR) 9.29, p = 0.045; need for mechanical ventilation OR 24.74, p = 0.006; success of therapeutic interventions OR 0.07, p = 0.020 and hemorrhagic shock OR 22.02, p = 0.011.

**Conclusion:** In patients with pancreatic cancer and GIB, the success of hemostatic interventions and thus ICU mortality is influenced by the mechanism of bleeding and the PT. Early identification of arterial aneurysm (abdominal CT-scan before endoscopy), especially in patients with previous pancreatic resection surgery history, and prompt PT correction should be proposed.

**Compliance with ethics regulations:** Yes in clinical research.

### FC-180 Clinical features and outcomes of patients with pancreatic cancer requiring medical ICU admission: a retrospective multicenter study

#### KEMOUN Gabriel^1^, WEISS Emmanuel^2,4^, EL HOUARI Lina^1^, CALIEZ Olivier^1^, GOURY Antoine^3^, BONNY Vincent^6^, PICARD Benjamin^1^, RUDLER Marika^1,5^, RHAIEM Rami^3^, REBOURS Vinciane^2,4^, MAYAUX Julien^1^, BELIN Lisa^1^, DEMOULE Alexandre^1,5^, DECAVÈLE Maxens^1,5^

##### ^1^APHP Sorbonne Université, site Pitié-Salpêtrière, Paris, France; ^2^APHP-Nord, Université de Paris, Hôpital Beaujon, Clichy, France; ^3^Hôpital Robert Debré, CHU de Reims, Reims, France; ^4^Université de Paris, Paris, France; ^5^Sorbonne Université, Paris, France; ^6^APHP-6 Sorbonne Université, site Saint-Antoine, Paris, France

###### Correspondence: Gabriel KEMOUN (gabriel.kemoun@gmail.com)

*Annals of Intensive Care* 2022, **12(1):**FC-180

**Rationale:** Pancreatic cancer is the sixth leading cause of solid malignancy in France, with increasing incidence and low survival probability. About 5–10% of cancer patients will require intensive care unit (ICU) admission in the 2-year following diagnosis. However, there is no data regarding clinical features and outcomes of pancreatic cancer patients admitted to the ICU. Primary aim was to describe the reasons for ICU admission. Secondary aim was to identify factors associated with ICU mortality.

**Patients and methods/Materials and methods:** Retrospective multicenter cohort study in five French ICUs from January 2009 to January 2020. All consecutive patients with proved pancreatic cancer admitted to the ICU were screened for inclusion. Patients with a recent surgery were excluded (< 4 weeks). Logistic regression multivariate analysis was performed on ICU mortality.

**Results:** Two hundred and sixty-nine patients were included (161 (60%) males, 65 [57 − 73] median [interquartile interval] years old, simplified acute physiology score 2 (SAPS 2) 39 [29–55]). Tumors were mainly adenocarcinoma (90%) in cephalic location (70%). Time between diagnosis and ICU admission was 8 [1–17] months. At ICU admission, most of the cancer were metastatic (57%) and the disease was responsive/stable, newly diagnosed or progressive in 32%, 25% and 43% of cases respectively. Prior to ICU admissions 74% of patients received chemotherapy, 24% surgery and 13% radiotherapy. By frequencies, reasons for ICU admission were sepsis/septic shock (32%) followed by gastrointestinal bleeding (28%), acute respiratory failure (16%), metabolic disorder (12%) and miscellaneous (12%). Among the 87 patients admitted with sepsis/septic shock, a biliary tract infection was identified in 45 (52%) patients. Biliary tract infection was more frequently observed in tumors with cephalic location than their counterparts (61% vs. 24%, p = 0.003). During the ICU stay, mechanical ventilation and vasopressors were required in 101 (38%) and 95 (35%) patients. ICU mortality rate was 26% 95% confidence interval [20%; 31%]. Using multivariate analysis, performance status 3–4 (OR 3.58), cancer status (responsive/stable -ref-, newly diagnosed OR 3.28, progressive OR 5.99), mechanical ventilation (OR 8.03), vasopressors (OR 4.19), SAPS 2 (OR 1.69) and pH (OR 0.02) were independently associated with ICU mortality.

**Conclusion:** The reasons for ICU admissions of pancreatic cancer patients seem to substantially differ from that observed in previous work on solid cancer (acute respiratory failure). ICU mortality seems roughly similar to that observed also in previous works on solid cancer and is strongly influenced by performance status, disease status and organs failure at ICU admission.

**Compliance with ethics regulations:** Yes in clinical research.

### FC-181 Quality of life of cancer patients at 3 months after ICU stay: a prospective case–control study

#### TOFFART Anne Claire^1^, MARNAS Wassila^1^, JERUSALEM Sophie^1^, GODON Alexandre^1^, BETTEGA Francois^1^, ROTH Gael^1^, PAVILLET Julien^1^, GIRARD Edouard^1^, GALERNEAU Louis Marie^1^, SCHWEBEL Carole^1^, PAYEN Jean Francois^1^

##### ^1^CHU GRENOBLE, La Tronche, France

###### Correspondence: Wassila MARNAS (wmsallaoui@chu-grenoble.fr)

*Annals of Intensive Care* 2022, **12(1):**FC-181

**Rationale:** Although short- and long-term survival in critically ill cancer patients was extensively studied, only limited data exist concerning their quality of life (QoL) following an intensive care unit (ICU) stay. We thus sought to determine the impact of an ICU stay on QoL at 3 months in solid cancer patients.

**Patients and methods/Materials and methods:** A prospective observational case–control study was conducted between February 2020 and February 2021 involving three French ICUs. Surviving ICU patients with lung, colorectal, or head and neck cancers at 3 months after ICU discharge were matched with non-ICU cancer patients in terms of their cancer type, anticancer treatment goal (curative/palliative), and treatment line. QoL was assessed using the Short Form 36 (SF-36) health survey (ranging 0–100, higher values indicating better QoL) including mental (MCS) and physical (PCS) components. Anticancer treatment at 3 months was additionally assessed.

**Results:** Overall, 23 surviving ICU cancer patients were matched with 46 non-ICU cancer patients. All patients answered the survey except four in the ICU group. MCS was significantly higher in ICU patients than in non-ICU patients, with a median of 54 (interquartile range [IQR]: 42–57) versus (vs.) 47 (IQR: 37–52), respectively (p = 0.01). PCS did not differ between both groups, with a median of 35 (IQR: 31–47) vs. 42 (IQR: 34–47) (p = 0.24). The multivariate analysis revealed a non-significant association between ICU/non-ICU patient status and patient quality-of-life scores (MCS or PCS) at 3 months post-discharge. The therapeutic strategy based on anticancer treatment at 3 months post-discharge was similar between both groups. A good performance status (PS) (coef. 1.46, confidence interval [CI] 95%: 1.11–1.90, p = 0.01) and non-metastatic cancer status (coef. 1.21, CI95%: 1.05–1.40, p = 0.01) were independently associated with a higher PCS.

**Conclusion:** At 3 months, cancer patients admitted into ICU displayed comparable QoL with matched patients not admitted into ICU. Thus, the challenging issue is to identify patients with good prognostic factors who would benefit from an ICU stay in terms of QoL and continue their anticancer treatments. These results require confirmation by a multicenter study.

**Compliance with ethics regulations:** Yes in clinical research.

### FC-182 Clinical features and outcomes of chimeric antigen receptor T-cell patients admitted to the intensive care unit: a single center experience

#### LE CACHEUX Corentin^1^, SORTAIS Clara^1^, GASTINNE Thomas^1^, SEGUIN Amélie^1^, BENGUERFI Soraya^1^, LACOU-AGBAKOU Maïté^1^, BLONZ Gauthier^1^, REIGNIER Jean^1^, CANET Emmanuel^1^

##### ^1^CHU de Nantes, Nantes, France

###### Correspondence: Corentin LE CACHEUX (lecacheuxcorentin@gmail.com).

*Annals of Intensive Care* 2022, **12(1):**FC-182

**Rationale:** Chimeric antigen receptor (CAR) T-cell therapy is a promising treatment for refractory hematological malignancies, but can induce severe adverse events. We aimed to report the clinical features and outcomes of CAR T-cell patients admitted to the intensive care unit (ICU).

**Patients and methods/Materials and methods:** We retrospectively included all adult patients admitted to a French University-affiliated ICU between August 27, 2018 and January 18, 2022, within 3 months after CAR T-cell therapy.

**Results:** We report 33 ICU stays for 30 patients, out of 87 (i.e., 34.5% ICU admission) treated by CAR-T cell therapy during the study period. Hematological malignancies were predominantly lymphoma (76.7%) and multiple myeloma (16.7%). ICU admission occurred 5 days (IQR 2.5–9.0) after CAR T-cell injection. Reasons for ICU admission were hemodynamic instability (42.4%), neurological failure (36.4%), and close monitoring (24.2%). Median SOFA andSAPS2 scores were 4 (IQR 3.0–6.5) and 45 (IQR 37–50), respectively. All but one patient (n = 29, 96.7%) met the criteria for cytokine release syndrome (CRS), of whom 5 (17.2%) developed grade 3–4 toxicity. Immune effector cell-associated neurotoxicity syndrome (ICANS) occurred in 20 (66.7%) patients, of whom 12 (60%) had grade 3–4 toxicity. ICANS was identified 4 days (IQR 2–4) after CRS, and 3 (10%) patients developed both CRS and ICANS grade 3–4 toxicities. Tocilizumab was used in 28 (93.3%) patients, with a median of 2 doses (IQR 1.0–3.0). Steroids were given in 24 (80%) patients, of whom 5 (20.8%) received high-dose pulse therapy. Broad-spectrum antimicrobial therapy was delivered during 32 (97%) ICU stays. Overall, 15 (50.0%) patients had a documented infection. Bacterial infections were reported in 13 (43.3%) patients and 2 (6.7%) patients had fungal infections (one pulmonary aspergillosis and one mucormycosis). Hemophagocytic syndrome was noted in 6 (20%) patients. Life-sustaining therapies were required for 12 (36.4%) patients: 11 (33.3%) received vasopressors, 7 (21.2%) were mechanically ventilated and 4 (12.1%) underwent dialysis. Overall, 3 (9.1%) patients died in the ICU (2 of them during a second ICU admission). At day-90, 11 (44.0%) patients were alive with complete remission, 5 (20%) were alive with hematological malignancy relapse, and 9 (36%) died.

**Conclusion:** ICU admission is common after CAR T-cell therapy, mostly for the management of specific toxicities. Our experience is encouraging with low ICU mortality despite a high rate of grade 3–4 toxicities, and almost half of the patients being alive in complete remission at day-90. Further studies are needed to improve the treatment of such toxicities which remains largely empirical.

**Compliance with ethics regulations:** Yes in clinical research.

### FC-183 Acute Kidney Injury in the setting of Tumor Lysis Syndrome: impact on long-term outcomes

#### BLOUET Anaise^3^, BOUD'HORS Charlotte^4^, LEMERLE Marie^5^, SCHMIDT Aline^6,7^, KOUATCHET Achille^5^, AUGUSTO Jean-François^4^, ORVAIN Corentin^6,7^, DEMISELLE Julien^1,2^

##### ^1^Hôpitaux Universitaires de Strasbourg, Nouvel Hôpital Civil, Strasbourg, France; ^2^INSERM UMR 1260 Regenerative Nanomedecine (RNM), FMTS, Strasbourg, France; ^3^Onco-hématologie, Strasbourg Oncologie libérale, Strasbourg, France; ^4^Service de Néphrologie, Dialyse, Transplantation, CHU Angers, Angers, France; ^5^Service de Médecine Intensive Réanimation, Médecine Hyperbare, CHU Angers, Angers, France; ^6^Service des Maladies du sang, CHU Angers, Angers, France; ^7^CRCINA, INSERM U1232, Université de Nantes, Université d'Angers, Angers, France

###### Correspondence: Julien DEMISELLE (julien.demiselle@chru-strasbourg.fr)

*Annals of Intensive Care* 2022, **12(1):**FC-183

**Rationale:** Acute kidney injury (AKI) is the main complication of tumor lysis syndrome, and is associated with high mortality and short term morbidity. However, the impact of AKI in this setting with long-term outcome is poorly investigated.

**Patients and methods/Materials and methods:** We collected and analyzed data of a retrospective monocentric cohort of adult patients, hospitalized between January 2007 and December 2017, who survived to an episode of tumor lysis syndrome. Tumor lysis syndrome was defined according to Cairo and Bishop criteria (1). For each patient, we recorded the occurrence of death, hematological disease progression, and modification in further chemotherapy regimen. In addition, we recorded renal function evolution, every three months during the first year, then annually. Chronic kidney disease was defined as an estimated glomerular filtration rate under 60 ml/min/1.73 m^2^ (eGFR CKD-EPI). We compared patient’s evolution (survival, progression free survival, modification of chemotherapy regiment and chronic kidney disease occurrence) according to AKI occurrence (AKI group) or not (no AKI group) during the initial tumor lysis syndrome episode.

**Results:** 115 patients were included in this analysis. Acute leukemia and non-Hodgkin lymphoma accounted for 84% of malignancies. Median duration of follow-up was 24.8 months (IQR 25–75 months [4.4–70.5]). During the initial tumor lysis syndrome episode, 57 patients experienced AKI. AKI and no AKI groups were similar at baseline, and in tumor lysis syndrome management. At the end of follow-up, mortality in the AKI group was 59.6% and 55,4% in the no AKI group (p = 0,644). Disease progression occurred in 44,4% and 39.2% in the AKI and no AKI groups, respectively (p = 0.587). We found a modification of chemotherapy regimen in 18 patients, only in the AKI group. Among these patients, persistent renal failure was considered as an additional comorbidity leading to palliative care in 8 of them. Renal function evolution was available in 81 patients, with a median follow-up of renal function of 39.9 months (IQR [25–75]: 13.2–78.7 months). CKD was observed in 21 patients (25.9%). CKD was mostly diagnosed during the first year after TLS (12/21 patients). No difference between AKI and no AKI groups was observed in CKD occurrence.

**Discussion:** AKI during tumor lysis syndrome was strongly associated with modifications in chemotherapy regimen. We found no impact of initial AKI on major patient’s centered outcomes, but our study may be underpowered.

**Conclusion:** AKI during tumor lysis syndrome impacts further chemotherapy regimen. Of note, more than one fourth of patients developed chronic kidney disease.

**Reference 1:** Cairo MS, Bishop M. Tumour lysis syndrome: new therapeutic strategies and classification. British Journal of Haematology. 2004;127(1):3–11.

**Compliance with ethics regulations:** Yes in clinical research.

### FC-184 A multicentric prospective observational study of diagnosis and prognosis features in ICU mesenteric ischemia: the DIAGOMI study

#### BOURCIER Simon^1,2^, ULMANN Guillaume^4^, JAMME Matthieu^5^, SAVARY Guillaume^1^, PAUL Marine^1^, BENGHANEM Sarah^1^, LAVILLEGRAND Jean-Rémi^3^, SCHMIDT Matthieu^2^, LUYT Charles-Edouard^2^, MAURY Eric^3^, COMBES Alain^2^, PÈNE Frédéric^1^, CYNOBER Luc^4^, NEVEUX Nathalie^4^, CARIOU Alain^1^

##### ^1^Medical intensive Care Unit, Cochin Hospital, AP-HP, Paris, France; ^2^Assistance Publique-Hôpitaux de Paris, AP-HP, Pitié-Salpêtrière Hospital, Médecine Intensive Réanimation, Paris, France; ^3^Assistance Publique-Hôpitaux de Paris, APHP, Saint-Antoine Hospital, Médecine Intensive Réanimation, Paris, France; ^4^Clinical Chemistry Department, Hôpital Cochin, AP-HP Centre, Université de Paris, Paris, France; ^5^Réanimation polyvalente, Hôpital Privé de l’Ouest Parisien, Ramsay Générale de santé, Trappes, France

###### Correspondence: Simon BOURCIER (simon_bourcier@hotmail.com)

*Annals of Intensive Care* 2022, **12(1):**FC-184

**Rationale:** Non-occlusive mesenteric ischemia (NOMI) is a challenging diagnosis and is associated with extremely high mortality in critically ill patients, particularly due to delayed diagnosis and when complicated by intestinal necrosis. Citrulline and intestinal-fatty acid binding protein (I-FABP) have been proposed as potential promising biomarkers but have never been studied prospectively in this setting.

**Patients and methods/Materials and methods:** We conducted a prospective observational study in 3 tertiary ICU centers. Diagnosis features and outcomes were compared according to NOMI, intestinal necrosis or ruled out diagnosis using stringent classification criteria.

**Results:** Diagnosis of NOMI was suspected in 61 patients and confirmed for 33 patients, with intestinal necrosis occurring in 27 patients. Clinical digestive signs, routine laboratory results and CT signs of mesenteric ischemia didn’t discriminate intestinal necrosis from ischemia without necrosis. Plasma I-FABP was significantly increased in presence of intestinal necrosis (AUC 0.83 [0.70–0.96]). A threshold of 3114 pg/mL showed a sensitivity of 70% [50–86], specificity of 85% [55–98], a negative predictive value of 58% [36–93] and a positive predictive value 90% [67–96]. Necrosis resection was significantly associated with ICU survival (38.5%), whereas no patient survived without intestinal resection (HR = 0.31 [0.12–0.75], p = 0.01).

**Conclusion:** In critically ill patients with suspected NOMI, intestinal necrosis was associated with extremely high mortality, and increased survival when intestinal resection was performed. Elevated plasma I-FABP was associated with the diagnosis of intestinal necrosis. Further studies are needed to set plasma I-FABP threshold valuable in clinical-decision making.

**Compliance with ethics regulations:** Yes in clinical research.

### FC-185 Relationship between the rate of full stomach and daily SOFA in the enteral-fed ICU patient

#### JOFFRÉDO Emilie^1^, DALSHEIMER Xavier^2^, BOUVET Lionel^3^, VACHERON Charles-Hervé^1^, LUKASZEWICZ Anne-Claire^2^, ALLAOUCHICHE Bernard^1^

##### ^1^Centre Hospitalier LYON Sud, Pierre-Bénite, France; ^2^Hôpital Edouard Herriot, Lyon, France; ^3^Hôpital Louis Pradel, Bron, France

###### Correspondence: Emilie JOFFRÉDO (emilie_joffredo@hotmail.com)

*Annals of Intensive Care* 2022, **12(1):**FC-185

**Rationale:** The provision of adequate nutrition is an important part of the management of intensive care patients. Enteral nutrition (EN) is the preferred mode of feeding in these patients. However, some patients have an ileus that causes gastric stasis, which can lead to reflux of nutritional fluid with risks of micro-inhalation. Ultrasonographic measurement of antral cross-sectional area (ACSA) is an easy and reliable bedside procedure to estimate the rate of full stomach in critically ill patients receiving EN in 30-degree head-of-bed elevation and supine position. This technique is already well recognized. The aim of this work was to analyze the relationship between the rate of full stomach and the SOFA score and its impact on the tolerance of enteral nutrition.

**Patients and methods/Materials and methods:** This prospective observational cohort study received approval (n°: L16-174) from the ethics committee (Comité pour la Protection des Personnes Sud-Est IV, Centre Léon Bérard, Lyon, France) and was registered in the public registry ClinicalTrials.gov, no. NCT03205592. In this prospective multicenter study, adult patients under mechanical ventilation were included, except supra-mesocolic surgery patients. Ultrasound examination of the gastric antrum was daily measured for five days long. Correlation was computed according to the method described by Bland and Altman specifically designed in order to compute correlation coefficient for repeated observation. Briefly, the coefficients of correlation (r) were estimated by modeling the effect of the gradient on the severity and introducing the patient as a dummy variable. Then, the coefficient was computed by the square root of the sum of square of the gradient /(sum of square of the gradient + residual sum of square), and the p value derived from the F test in the associated analysis of variance table of the model.

**Results:** Thirty-five patients were analyzed: age = 61 ± 14, 9 female, SAPS 2 = 40 ± 17, admission SOFA = 4 ± 2. The incidence of full stomach varied between 77 to 89% during the follow-up period. The repeated measures correlation was 0.087, p = 0.38 (Fig. 1). We found no relation between rate of full stomach and the incidence of ventilator-associated pneumonia or vomiting. The incidence of full stomach was not influenced by diabetes, Covid, sepsis, trauma, sedation, norepinephrine, renal replacement therapy or prone position.

**Conclusion:** Full stomach was frequently found but had no association with the SOFA score nor pneumonia or vomiting in enteral fed ICU patients.

**Compliance with ethics regulations:** Yes in clinical research.
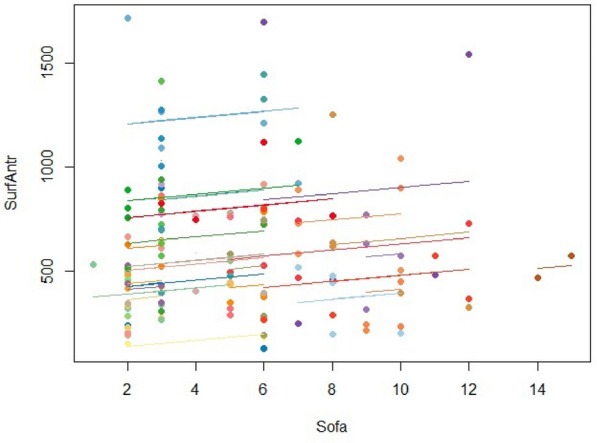


*Figure 1*

### FC-186 Extravascular lung water predicts progression to acute respiratory distress syndrome in critically ill cirrhotic patients

#### SCHOULMANN Alix^1^, PLATTIER Rémi^1^, ANTY Rodolphe^1^

##### ^1^Hopital Archet 2, Nice, France

###### Correspondence: Alix SCHOULMANN (alix.schoulmann@gmail.com)

*Annals of Intensive Care* 2022, **12(1):**FC-186

**Rationale:** Acute respiratory distress syndrome (ARDS) is a syndrome of acute respiratory failure caused by a protein-rich alveolar edema fluid. Its incidence is variable and often related to a poor outcome. Extravascular lung water (EVLW) estimated by transpulmonary thermodilution using the PiCCO device is a hallmark of lung edema, and can help to predict the progression to lung injury in patients with risk factors. It is also an independent prognostic factor, and can further be used to guide fluid management. Thereby, we aim to evaluate the reproducibility of EVLW’s usefulness in critically ill cirrhotic patients.

**Patients and methods/Materials and methods:** The medical records of 90 critically ill cirrhotic patients who benefited of haemodynamic monitoring using the PiCCO device from January 2013 to November 2019 were retrospectively reviewed. Independent risk factors and outcomes were then analyzed according to whether their EVLW value on ICU admission was greater than 10 mL/kg or not.

**Results:** An EVLW > 10 ml/kg at ICU admission is associated with ARDS (32% vs 8% p < 0,01), and with the onset of ARDS during hospital stay (66% vs 33% p < 0,01). Using a ROC curve, an EVLW cutoff of 10 ml/kg has been defined with the best sensitivity (74%) and specificity (62%) threshold. There is no difference between the groups regarding ICU mortality rate (51% vs 55%, p = 0,71), nor regarding the proportion of patients with cirrhotic ascites (61% vs 73%, p = 0,21 et 78%, p = 0,95) or with pleural fluid (39% vs 24%, p = 0,14 et 66% vs 47%, p = 0,072), respectively on ICU admission and during the stay. In multivariate analysis, TIPSS history and ARDS during hospital stay were both independent risk factors of EVLW > 10 ml/kg (OR = 7.7 [1.36 – 43]; p = 0.02 and OR = 4.9 [1.9 – 12.6]; p = 0.001 respectively).

**Conclusion:** An EVLW > 10 ml/kg at ICU admission is associated with the onset of ARDS in critically ill cirrhotic patients, without interference in the measure from cirrhotic ascites or pleural fluid.

**Reference 1:** ARDS Definition Task Force, Ranieri VM, Rubenfeld GD, et al. Acute respiratory distress syndrome: the Berlin Definition. JAMA. 2012;307(23):2526–2533.

**Reference 2:** Monnet X. Transpulmonary thermodilution: advantages and limits. Published online 2017:12.

**Compliance with ethics regulations:** Yes in clinical research.

### FC-187 Liver transplantation for critically ill cirrhotic patients: results from the French transplant registry

#### ARTZNER Thierry^1^, LEGEAI Camille^2^, ANTOINE Corinne^2^, JASSERON Carine^2^, MICHARD Baptiste^1^, FAITOT François^1^, SCHNEIDER Francis^1^, BACHELLIER Philippe^1^, CASTELAIN Vincent^1^

##### ^1^CHU Strasbourg, Strasbourg, France; ^2^Agence de la Biomédecine, Saint Denis, France

###### Correspondence: Baptiste MICHARD (baptiste.michard@chru-strasbourg.fr)

*Annals of Intensive Care* 2022, **12(1):**FC-187

**Rationale:** This study describes the population of cirrhotic patients who were transplanted from the ICU in France. It identifies pre-transplant risk factors of post-transplant mortality and describes geographic variations in ICU transplant activity.

**Patients and methods/Materials and methods:** Cirrhotic patients transplanted between 2008 and 2018 were included through the national transplant registry. The demographic, clinical and biological characteristics of the patients transplanted from the ICU were compared to cirrhotic patients who were transplanted from home or from the hospital. Risk factors of post-transplant one-year mortality were identified in uni- and multivariable analysis within the population transplanted from the ICU. Funnel plots were used to illustrate center-specific differences in ICU transplant activity.

**Results:** 1,047 cirrhotic patients were transplanted from the ICU during the study period. While the national rate of transplants performed from the ICU was 14.3%, the absolute number and the proportion of cirrhotic patients transplanted from the ICU varied significantly from one center to another, ranging from 6.6% to 22.8% (p < 0.05) (cf. figure). The one-year post-LT survival rate for cirrhotic patients transplanted from the ICU was significantly lower compared to cirrhotic patients transplanted from the hospital and from home (77.1% vs 88.0% vs 89.8% respectively, p < 0.0001). Three recipient associated independent risk factors of one-year post-LT mortality were identified in the population transplanted from the ICU: age > 50 years (HR 1.65, 95%CI 1.16–2.36), p = 0.005), diabetes (HR 1.46, 95%CI 1.07–1.98, p = 0.02) and intubation (HR2.12, 95%CI 1.62–2.78), p < 0.001). Donor age was also independently associated with mortality (HR 1.01, 95%CI 1.01–1.02, p < 0.001).

**Conclusion:** This study underlines the increased post-transplant mortality among cirrhotic patients transplanted from the ICU. It identifies four clinically pertinent independent risk factors associated with post-transplant mortality in this specific sub-group of transplant candidates. Finally, it illustrates how diverse the landscape of liver transplantation for critically ill cirrhotic patients is across a single country, despite a unified allocation algorithm.

**Compliance with ethics regulations:** Yes in clinical research.

### FC-188 Pretransplant intensive care unit management and selection of grade 3 acute-on-chronic liver failure transplant candidates

#### MICHARD Baptiste^1^, ARTZNER Thierry^1^, DERIDDER Mathilde^1^, BESCH Camille^1^, ADDEO Pietro^1^, CASTELAIN Vincent^1^, GUILLOT Max^1^, LEROUX Justine^1^, HERBRECHT Jean Etienne^1^, JANSSEN LANGENSTEIN Ralph^1^, SCHENCK Maleka^1^, BACHELLIER Philippe^1^, SCHNEIDER Francis^1^, FAITOT Francois^1^

##### ^1^CHU Strasbourg, Strasbourg, France

###### Correspondence: Baptiste MICHARD (baptiste.michard@chru-strasbourg.fr)

*Annals of Intensive Care* 2022, **12(1):**FC-188

**Rationale:** The aim of this study is to report on the liver transplant (LT) activity and post-LT outcome over time of grade 3 acute-on-chronic liver failure (ACLF3) patients in the largest single center cohort of patients transplanted with ACLF3 published in the literature. It aims at showing how pre-LT intensive care unit (ICU) management impacts post-LT outcome, in particular through monitoring the transplantation for ACLF3 model (TAM) score, which is based on the recipient’s age, arterial lactate level, white blood cell count and respiratory status.

**Patients and methods/Materials and methods:** 100 consecutive patients who had ACLF3 at the time of liver transplant between 2007 et 2019 were included retrospectively. The cohort was divided in 2 periods, with 50 patients in each period.

**Results:** There was a sharp increase in the number of ACLF3 patients transplanted over the course of the study period (from 4.2 to 12.4 patients transplanted with ACLF3 per year), and significantly higher one-year post-LT survival in the second period compared to the first period (respectively 86% vs 66%, p = 0.035). Interestingly, patients during both periods had similar severity profiles and scores, apart from a significantly lower number of patients with TAM scores > 2 at the time of LT in the second period compared to the first period. In addition, patients whose clinical condition improved in the ICU (with a TAM score being downstaged between admission and LT) had significantly higher post-LT survival rates than those whose TAM score stayed the same or increased: respectively 88% vs. 70% (p = 0.042). Finally, the predictive power of the TAM score increased as critically ill cirrhotic patients were closer to the time of LT (cf. figure). In particular, the TAM score was most predictive immediately prior to LT (at the time of organ proposal), with significantly higher survival rates for patients with TAM score 0–2 vs. TAM score > 2: respectively 85% vs. 8% (p < 0.0001).

**Conclusion:** This study shows a learning curve in LT for ACLF3 patients, with optimized ICU management and patient selection leading to increased numbers of LTs for ACLF3 patients and improved post-LT outcomes. It delineates how the TAM score can be used in practice to identify the optimal transplantability window for ACLF3 patients. It shows that the decision to transplant candidate with ACLF3 should not be made prematurely close to ICU admission but rather at the time of organ proposal and that patients whose clinical state improves in the ICU have better post-LT survival.

**Compliance with ethics regulations:** Yes in clinical research.

### FC-189 Predictors of early mortality after liver transplantation for acute liver failure

#### MOULIADE Charlotte^1,2,3^, SACLEUX Sophie-Caroline^1,2,3^, ORDAN Marie-Amélie^1^, BOUDON Marc^1,2,3^, COILLY Audrey^1,2,3^, CAILLEZ Valerie^1,2,3^, SAMUEL Didier^1,2,3^, SALIBA Faouzi^1,2,3^, ICHAI Philippe^1,2,3^

##### ^1^AP-HP Hôpital Paul-Brousse, Centre Hépato-Biliaire, Liver Intensive Care Unit, Villejuif, France; ^2^INSERM Unité 1193, Université Paris-Saclay, Villejuif, France; ^3^DHU Hepatinov, Villejuif, France

###### Correspondence: Charlotte MOULIADE (charlotte.mouliade@gmail.com)

*Annals of Intensive Care* 2022, **12(1):**FC-189

**Rationale:** After liver transplantation for acute liver failure (ALF), the majority of deaths occur early, in the days and weeks after surgery, due to organ dysfunctions or failures at the time of transplantation.

**Patients and methods/Materials and methods:** Between 2010 and 2021, 105 consecutive patients (62 women, 43 men, mean age 41.5 years) from a single center, with ALF registered for emergency liver transplantation were studied. Epidemiological, clinical, biological and radiological characteristics were collected upon admission, at registration on the transplant list and at the time of transplantation. The objective was to determine the predictive factors associated with 7-day mortality following the liver transplantation.

**Results:** The cause of ALF was paracetamol in 42 patients and non paracetamol in 63. Ninety-eight (93.3%) patients presented hepatic encephalopathy. Mean PT at admission was 17%. The mean SAPS 2 and SOFA scores were 45.4 and 9.8 respectively. All fulfilled the criteria for liver transplantation (King’s college and/or Clichy-Villejuif criteria). At registration for the liver transplant, 53 patients (50.5%) were treated with renal replacement therapy, 44 (41.9%) received vasopressor support, and 72 (68.6%) were on mechanical ventilation. Sixty-four patients were transplanted, 15 died prior to liver transplantation and 26 improved without liver transplantation. Eight patients died following liver transplantation. The main causes of death were: multi-organ failure (60.9%), brain death (26.1%), ARDS (8.7%) and ventricular fibrillation (4.3%). Among these patients, 6 died within 7 days of transplantation and 2 after 8 days. The predictors of early mortality (day 7) at the time of transplantation in univariate analysis were: anuria (p = 0.018), low pH (p = 0.038), low bicarbonate level (p = 0.0006) and high lactate level (p = 0.01). Three events occurring before liver transplantation were associated with an increased risk of early mortality: cardiopulmonary arrest, total hepatectomy with portocaval anastomosis for toxic liver and extensive digestive ischemia. An increase in catecholamine doses was also associated with early mortality, although this association was not statistically significant.

**Conclusion:** This single-center study made it possible to highlight several predictive factors of early mortality after liver transplantation for ALF. The sample size was the main limitation of this study, and a multicenter study is needed to determine independent risk factors.

**Compliance with ethics regulations:** Yes in clinical research.

### FC-190 Gut microbiota composition but not? diversity is linked to subsequent occurrence of ventilator-associated pneumonia in critically patients

#### ORIEUX Arthur^1^, ENAUD Raphaël^1^, IMBERT Sébastien^1^, BOYER Philippe^1^, BEGOT Erwan^1^, CAMINO Adrian^1^, BOYER Alexandre^1^, BERGER Patrick^1^, GRUSON Didier^1^, DELHAES Laurence^1^, PRÉVEL Renaud^1^

##### ^1^CHU Bordeaux, Bordeaux, France

###### Correspondence: Renaud PRÉVEL (renaud.prevel@hotmail.fr)

*Annals of Intensive Care* 2022, **12(1):**FC-190

**Rationale:** Ventilator-associated pneumonia (VAP) is the most frequent nosocomial infection in critically ill ventilated patients carrying high morbidity and an increase in healthcare costs. Oro-pharyngeal and lung microbiota have been demonstrated to be associated with VAP occurrence but gut microbiota involvement has not been investigated so far despite the relevance of the gut–lung axis in human health and diseases. The aim of this study is thus to compare the composition of the gut microbiota between patients who will subsequently develop VAP and those who will not.

**Patients and methods/Materials and methods:** A rectal swab was performed for routine faecal ESBL-E carriage screening at admission of every consecutive patient into intensive care unit (ICU) from October 2019 to March 2020. After DNA extraction, V3–V4 and ITS2 regions were amplified by PCR. Deep-sequencing was performed on MiSeq sequencer (Illumina^®^); data were analysed using DADA2 pipeline.

**Results:** Among 255 patients screened, 42 (16%) patients with invasive mechanical ventilation for more than 48 h were included, 18 (43%) with definite VAP and 24 without (57%). Patients with subsequent VAP had similar gut bacteriobiota and mycobiota α-diversities than those who will not develop VAP but gut mycobiota was dissimilar between those 2 groups. The presence of Megasphaera massiliensis was associated with the absence of VAP occurrence whereas the presence of *Bifidobacterium breve*, *Peptostreptococcus anaerobius*, *Enterococcus avium*, *Alternaria* sp. and *Saccharomyces kudriavzevii* was associated with subsequent VAP.

**Conclusion:** The composition of the gut microbiota, but not α diversity, differs between critically ill patients who will subsequently develop VAP and those who will not. This study encourages for large multicentre cohort study investigating the role of gut-lung axis and oro-pharyngeal colonization in the development of VAP in ICU patients and identify potential probiotic candidates for tailored prevention of VAP.

**Compliance with ethics regulations:** Yes in clinical research.

### FC-191 Decontamination regimen and MDR environmental contamination: a sink survey

#### MASSART Nicolas^1^, DUPIN Clarisse^1^, MARIE Veronique^1^, BARBAROT Nicolas^1^, LEGAY Francois^1^, FILLATRE Pierre^1^

##### ^1^CH Saint-Brieuc, Saint-Brieuc, France

###### Correspondence: Nicola MASSART (nicolasmassart@hotmail.fr)

*Annals of Intensive Care* 2022, **12(1):**FC-191

**Rationale:** Despite a large number of scientific data supporting clinical benefit of selective decontamination in ICU patients with a decreased risk of multi-drug resistant micro-organisms acquisition, data regarding environmental impact are scarce. We aim to provide a better knowledge on the relationship between decontamination regimen and environmental contamination.

**Patients and methods/Materials and methods:** We conducted an observational analysis in a 14 beds French polyvalent ICU in Saint-Brieuc, Bretagne, France. As of 05th of May 2021, patients with expected mechanical ventilation duration over 24 h received multiple site decontamination (MSD) regimen. MSD is a variant of selective digestive decontamination that included topical antibiotics (gentamycin, polymyxin and amphotericin B) 4 times daily in the oropharynx and the gastric tub, without systemic antibiotic, in addition with a daily 4% chlorhexidine bodywash and a 5-day intra nasal mupirocin course. Two-point prevalence study of sink contamination was performed on June 26th 2020 (pre-implementation) and October 20th 2021 (post-implementation) in the 24 sinks of the unit, at least48 hours after last disinfection procedure.

**Results:** Samples from the pre-implementation period showed a higher rate of ESBL-PE with 12 sinks (50%) positive for 13 extended spectrum betalactamase producing enterobateriaceae (ESBL-PE) specimens as compared with 4 sinks (17%) containing 4 ESBL-PE during post-implementation period (Macnemar paired test = 0.043). Conversely, the number of positive samples for imipenem resistant Pseudomonas aeruginosa (IRPA) increased 1 (4%) and 8 (33%) positive sinks in the pre and post-implementation periods respectively (Macnemar paired test = 0.046) (Figure). Of note, there were 11 Pseudomonas aeruginosa infections and 1 ESBL-PE infection during 6 months pre-implementation (3112 patients-days) as compared with 2 Pseudomonas aeruginosa and 1 ESBL-PE infections in the 6 months post-implementation (2196 patients-days) (p = 1 for ESBL-PE infections and p = 0.023 for PA, poisson regression test).

**Discussion:** Previous studies already reported a diminution of colonization with Gram-negative resistant bacteria in patients receiving decontamination regimens. Decrease in ESBL-PE sink colonization may be secondary to a decrease of patient-to-sink ESBL-PE transmission. Despite a diminution in the incidence of Pseudomonas aeruginosa infections between both periods, there are concerns regarding the increased in IRPA in the sinks. A study previously suggested that exposure to common disinfectant leads to expression of outer membrane protein OprR, giving potential resistance to carbapenems.

**Conclusion:** In conclusion, we described for the first time a diminution of ESBL-PE but an increased in IRPA sink contamination with MSD regimen including topical antibiotic and chlorhexidine body wash.

**Compliance with ethics regulations:** Yes in clinical research.
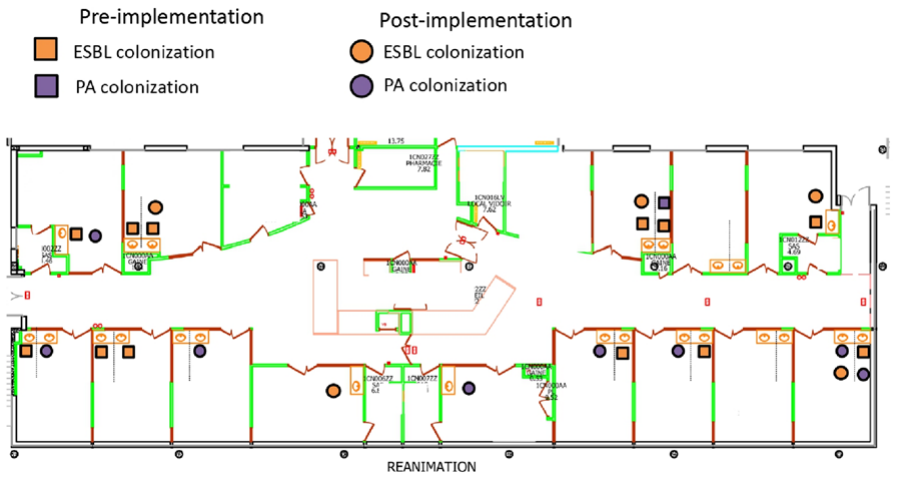


*Figure. ICU maps*

### FC-192 Echocardiography in the Time of COVID-19.

#### SDIRI Ines^1^, FITOUHI Nizar^1^, DRIDI Amira^1^, LAROUI Sana^1^, OUERGHI Sonia^1^, MESTIRI Tahar^1^

##### ^1H^Hôpital Ariana Abderahmen Memi, Sfax, Tunisie

###### Correspondence: Ines SDIRI (sdiri.ynes@gmail.com)

*Annals of Intensive Care* 2022, **12(1):**FC-192

**Rationale:** Many published studies during the Covid-19 pandemic have focused on the respiratory component and their management while the hemodynamic component has had a major impact (1). Despite the important incidence of cardiac complications, few studies have reported the place of echocardiography in Covid-19 patients (2). Our study aimed to report the echocardiographic findings in severe covid-19 patients.

**Patients and methods/Materials and methods:** This is a retrospective descriptive study conducted at the medical intensive care unit of circumstance Hospital, between January 1, 2021 and December 31, 2021. Were included the confirmed covid-19 patients of severe form and having benefited from a echocardiography at admission. An evaluation of the left ventricular function, the right one, the filling pressures, the valves and the pericardium.

**Results:** 300 results were collected. Mean age was 57 years, 53.6% male. The BMI was 30.84. Comorbidities were hypertension 37.3%, diabetes 33.1% and cardiovascular pathologies 14%. The mean PaO2/FiO2 ratio was 123 and the CT scan injury was 71.12%. The use of mechanical ventilation was 57.7% and catecholamines 62.8%. The average length of stay was 10 ± 8 days. The incidence of thrombi-embolic events was 22.1%. Mortality was 45%. Cardiac ultrasound was totally transthoracic. LV ejection function was impaired in 20.8% (< 50% in women and < 55% in men). Septal hyperkinesia was observed in 4% revealing ACS. 3% had acute pulmonary heart disease as evidenced by the presence of a paradoxical septum. 31.6% had relaxation disorders and 1% had compliance disorders. The evaluation of the filling pressures was in the gray zone for 33.7% and 2% elevated. The patients were mostly in hypovolemia 78.6% objectified by an inferior vena cava diameter lower than 15 cm. PAH was found in 10% of cases. Valvulopathies were present in 10.9%, dominated by mitral insufficiency 42% and tricuspid insufficiency 58% minimal justified by ARDS. Pericardial effusion was found in 13.9%, of minimal to moderate abundance.

**Conclusion:** Our study underlines the contribution of cardiac ultrasound in severe covid-19 to detect cardiac complications, the repercussion of ARDS in the hemodynamics and to guide the adequate management.

**Reference 1:** J Am Soc Echocardiogr. août 2020;33(8).

**Reference 2:** J Am Coll Cardiol. 19 janv 2021;77(2).

**Compliance with ethics regulations:** Yes in clinical research.

### FC-193 The contribution of Tocilizumab in treatment of patients with a SARS-COV2 infection

#### BOUCHECH Bouthaina^1^, CHTOUROU Ameni^1^, DAMMAK Rania^1^, KOLSI Hichem^2^, DERBEL Rahma^1^, BEN OMOR Oumaima^2^

##### ^1^Hôpital Habib Bourguiba, Sfax, Tunisie; ^2^Hôpital de campagne Slim Chaker, Sfax, Tunisie

###### Correspondence: Bouthaina BOUCHECH (bouthainasellami@gmail.com)

*Annals of Intensive Care* 2022, **12(1):**FC-193

**Rationale:** SARS-COV2 virus is responsible for hypoxemic pneumonia that can evolve into an acute respiratory distress syndrome The Tocilizumab is indicated in the treatment of severe syndromes caused by cytokine release induced by CAR-T lymphocytes and therefore the objective of this study is to determine the contribution of this molecule in the treatment of critical patients infected with SARS-COV2.

**Patients and methods/Materials and methods:** it is a descriptive retrospective study including 20 patients hospitalized in an intensive care unit for a SARS-COV2 pneumonia during the period extending from March 2021 to October 2021. These patients have received 2 doses of Tocilizumab equivalent to 8 mg per kg within an interval of 48 h between the two doses. The inclusion criteria are patients with an elevated level of IL6 (superior to 7 pg/ml), hospitalized in an intensive care unit, under high oxygen flows (superior to 10 l/min). The exclusion criteria are the presence of a bacterial infection, hepatocellular failure, thrombocytopenia, pregnancy and intubated patients.

**Results:** our study included 20 patients with an age average of 56 years and a sex ratio of 1.16. These patients are classified ASAII at minimum. All patients were under corticosteroid therapy .16 patients had a thoracic scan revealing a lung damage extended on 25% to 50%. During their stay in the ICU, all of these patients presented with a respiratory deterioration between the third and 22nd day of evolution 80% of patients were under either a high flow oxygen or high concentration mask and 20% were under a non-invasive ventilation. Before the administration of the cure, their level of IL6 was between 12 and 107 pg/ml, the level of AST was between 18 and 76 IU/L and the level of AlT was between 12 and 73 IU/L. The treatment was administered between day 5 and day 19 The complications induced by the treatment were a respiratory deterioration in 80% of the cases, a nosocomial infection in 15%, hepatic cytolysis in 10%, and anaphylactic choc in 5% After a week, 30% of patients were out of the ICU and put under oxygen with a flow between 6 and 15 l/min while 70% were still under mechanical ventilation.

**Conclusion:** In Tunisia the number of reported cases is still low. However, it is essential to conduct further clinical trials to evaluate the efficiency as well as the timing of administration of Tocilizumab in relation to the beginning of symptoms especially that this treatment is not risk free.

**Compliance with ethics regulations:** Yes in clinical research.

### FC-194 Dexamethasone or methylprednisolone therapy in covid-19 pneumonia: a retrospective and comparative study of 513 cases

#### EL MEZZEOUI Sanae^1^, BEN CHAIB Rajae^1^, HOUSNI Brahim^1^, BKIYER Houssam^1^.

##### ^1^centre hospitalier mohammed V oujda, Oujda, Maroc.

###### Correspondence: Sanae EL MEZZEOUI (sanae.elmezzeoui@gmail.com)

*Annals of Intensive Care* 2022, **12(1):**FC-194

**Rationale:** Corona virus disease 19 (Covid-19) affects especially the respiratory tract, and induces lung injury which may progress to the acute respiratory distress syndrome (ARDS). Various treatment options were tried all over the world, corticosteroids had showed beneficial effects. The Objective of this study, is to compare the safety and efficiency of two corticosteroids: dexamethasone and prednisolone in the treatment of Covid-19 infection.

**Patients and methods/Materials and methods:** This retrospective and comparative study included 513 patients diagnosed with Covid-19 infection and were admitted to intensive care unit of our university hospital center of MOHAMMED VI Oujda from March 1, 2020, to December 31st, 2020.

**Results:** In this study, 513 cases were included, 230 patients received methylprednisolone, and 283 were treated with dexamethasone. The median age in methylprednisolone group was 64 years, and 63 years in the second group treated with dexamethasone. Patients treated with dexamethasone had more critically lesions compared to patients treated with methylprednisolone (67.6%), these patients had a good evolution with a significant reduction of oxygen supplementation, lower use of invasive ventilation and a significant improvement in biological parameters. The difference in outcome between the two groups in terms of mortality was significantly reduced in the second group.

**Conclusion:** Both steroids are efficient in the management of mild, moderate and severe Covid-19 pneumonia with a clear superiority of dexamethasone especially in severe forms.

**Compliance with ethics regulations:** Yes in clinical research.

### FC-195 Prediction of ICU mortality in critically ill patients with COVID-19

#### TRIFI Ahlem^1^, CHERIF Hichem^2^, SEGHIR Eya^1^, JERIBI Badis^2^, ABDENNEBI Cyrine^1^, MASSEOUDI Yosri^2^, ABDELLATIF Sami^1^, AMMOUS Adel^2^, BEN LAKHAL Salah^1^

##### ^1^Medical ICU, la Rabta hospital, Faculty of Medicine of Tunis, Tunis, Tunisie; ^2^Deparetment of anesthesia, la Rabta hospital, Faculty of Medicine of Tunis, Tunis, Tunisie

###### Correspondence: Ahlem TRIFI (trifiahlem2@gmail.com)

*Annals of Intensive Care* 2022, **12(1):**FC-195

**Rationale:** COVID-19 pandemic has rapidly required an increased number of ICU admission and deaths. In view of this alarming situation, it became mandatory to develop prognostic models to evaluate critical COVID-19 patients. We aimed to study the characteristics and prognosis of adult patients with critical COVID-19 requiring hospitalization in ICU during 2nd, 3rd and 4th waves of the pandemic in order to identify the predictive factors of mortality.

**Patients and methods/Materials and methods:** We retrospectively evaluated a cohort of consecutive COVID-19 critically ill patients admitted to ICU with a confirmed diagnosis of SARS-CoV-2 pneumonia. A multivariable regression model including demographic, clinical and laboratory findings was developed to assess the predictive value mortality of these variables using the ROC curves. Our main outcome was ICU mortality.

**Results:** 358 were included (genre-ratio = 1.75, mean age = 60 + 13 years, median BMI = 27.7 kg/m^2^ and SOFA = 4 [3–6]). The most observed age class was 60–79 and 44% were obese. 72 (20%) had no comorbidities. Arterial hypertension (40.2%) and diabetes (39.1%) were the most reported co-morbidities. The median time of respiratory distress from the symptomatology onset was 11 days [8–15]. Clinical signs were: dyspnea (94%), fever (72.6%) and cough (70.4%). The median P/F ratio was 90 [67–129.5] and 58.5% of patients were in severe ARDS. In 45% of cases, ground-glass opacities exceeded 50% in the chest CT. 218 patients (61%) required mechanical ventilation (MV) and among them 12 (5.5%) were successfully weaned. The most complications were shock (49%), acute kidney injury (31%) and ICU related infection (34%). The evolution was fatal in 64% of cases (n = 229). In Multivariable Model: Tachycardia (HR > 99 bpm), an initial P/F ratio < 107 and MV were the factors independently associated to mortality with respectively OR [95% CI] at 39.9 [1.2–1307], 424 [2,23–80560] and 2235 [2,74–181842]. Other factors tended to the significance: SOFA > 3.5 (OR = 292 [0.9–9715], p = 0.055), a lymphocyte count < 555 el/mm3 (OR = 92,3 [0.84–1073], p = 0.059) and occurrence of shock (OR = 119 [0.99–15679], p = 0.055). The most predictive death factors were the use of MV (AUC/ROC = 0.904 [0.866–0.941], p < 10–3, Se = 90% and Sp = 90.7%) and shock (AUC/ROC = 0.854 [0.814–0.895], p < 10–3 and Sp = 96.2%). The other variables had significant AUC/ROC but a low predictivity (table).

**Conclusion:** in our large series, independent factors of mortality were a deep hypoxemia, tachycardia, mechanical ventilation, and with a trend to a signficance shock, presence of more than 3 organ dysfunctions and lymphopenia. The highly predictive death factors were MV and Shock.

**Compliance with ethics regulations:** Yes in clinical research.
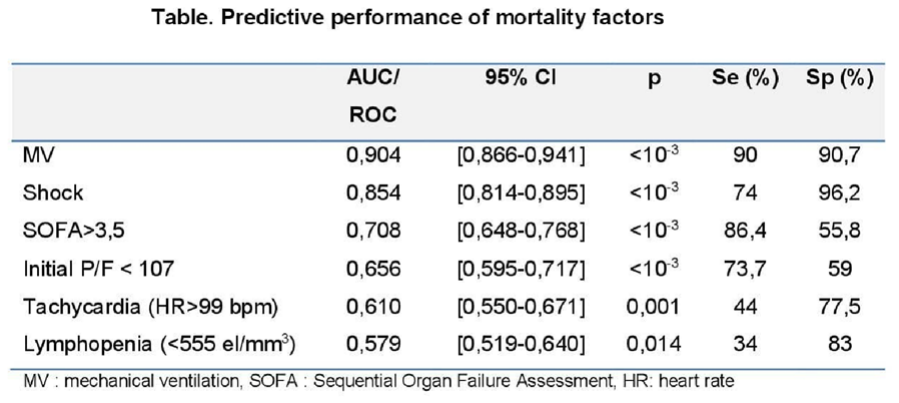


*Predictive performance of mortality factors*

### FC-196 Characterization of patients who return to Emergency department following discharge from hospitalization for COVID-19

#### JERBI Mouna^1^, GHORBEL Rezk^1^, GHARBI Emna^1^, SNOUSSI Haifa^1^, AFFES Lobna^1^, KESENTINI Bouthaina^1^, CHAKROUN Olfa^1^, REKIK Noureddine^1^

##### ^1^Centre Hospitalo-universitaire Habib Bourguiba sfax, Sfax, Tunisie

###### Correspondence: Rezk GHORBEL (rezkghorbel3@gmail.com)

*Annals of Intensive Care* 2022, **12(1):**FC-196

**Rationale:** To describe the specific epidemiological profile of post-COVID patients consulting in the emergency ward, the characteristics of the infectious episode, these repercussions on daily life as well as data concerning the symptoms, the problems and the reasons for consulting.

**Patients and methods/Materials and methods:** This is a descriptive prospective study collecting post-COVID patients, who presented to the medical emergency ward, regarding age and reason for consulting during the period from 1 June to 30 August 2021.

**Results:** 111 patients returned for emergency care after a median period of 8 ± 2 days, with 32.4% requiring readmission. Mean age was 54 ± 18 years, 42.9% men. Their chronic medical conditions included hypertension 25 (22.5%), diabetes mellitus 26 (23.4%) and coronary artery disease 4 (3.6%). The reasons for consulting the emergency ward, were mainly represented by exacerbation of dyspnea (39%). Main reasons for readmission were acute respiratory failure (25.2%), chest pain (25.2%). The rate of arterial or venous thromboembolic complications in COVID-19 patients was (21%). Among patients readmitted to the hospital 4% were hospitalized in intensive care unit.

**Conclusion:** The follow-up of post-COVID patients is very interesting. It makes it possible to identify different problems that can be classified as acute complication or a post-COVID syndrome, including respiratory failure. It requires monitoring and multidisciplinary care involving many specialties.

**Compliance with ethics regulations:** Yes in clinical research.

### FC-197 Vaccination against COVID-19: knowledge and perception of healthcare givers working in COVID sectors

#### TRIFI Ahlem^1^, MEHDI Asma^1^, MASSEOUD Lynda^1^, MEFTEH Amal^1^, SEGHIR Eya^1^, ABID Emna^1^, ABDENNEBI Cyrine^1^, ABDELLATIF Sami^1^, BEN LAKHAL Salah^1^

##### ^1^Medical ICU, la Rabta hospital, Faculty of Medicine of Tunis, Tunis, Tunisie

###### Correspondence: Ahlem TRIFI (trifiahlem2@gmail.com)

*Annals of Intensive Care* 2022, **12(1):**FC-197

**Rationale:** The generalization of vaccination against SARS-CoV-2 is one of the pillars of the strategy to fight against the virus spread and limit the morbidity and mortality. In this context, the role of caregivers is paramount by being a frequently requested source for information and influence. OBJECTIVE: To assess the knowledge and perception of healthcare givers (HCG) working in COVID sectors regarding the SARS-CoV-2 vaccine.

**Patients and methods/Materials and methods:** Online survey targeting HCG working in the COVID sectors of Tunisia. The questionnaire was created on the Google-Forms platform exploring 3 sections: personal data, knowledge and perceptions about vaccination.

**Results:** 282 HCG responded to the questionnaire. The majority were medical physicians (199 (70.5%) including seniors (n = 106). The age range of 18–39 years was predominant (68%) and the sex ratio (M/F) = 0.35. Medical HCG exercised in university hospitals in 230 cases, private (n = 15) and regional (n = 37) and intensive care accounted for 43% of the nature of COVID activity. Half (48%) contracted the virus SARS-CoV-2 and the clinical form was considered minor in 82% of cases and severe requiring resuscitation in 5 cases. 87% received their full dose and 45% were within 3–6 months of the 2nd dose. Pfizer-Biontech (48%), Sputnik (34%) and Chinese vaccines (13%) were the most received. As for knowledge and opinions, there was a discrepancy between the medical and paramedical HCG: the second category was less convinced by the vaccination, recommended the vaccine less to their advice seekers, believed less that immunity would be better acquired by the disease than by the vaccine and had less confidence in science in the development of the anti-SARS-CoV vaccine. Twenty-five (9%) of the responders considered themselves to be anti-vaccine (medical HCG: 4 and paramedical HCG: 21) and the main reason was the lack of information about vaccines.

**Conclusion:** The existing perception and attitude of frontline health workers differ depending on medical or paramedical status. Motivation and positive influence were felt less by the second population. Measures are essential to minimize these gaps: more awareness programs adapt communication strategies, obligations? …

**Compliance with ethics regulations:** N/A.

### FC-198 Mental health of intensive care professionals more than a year after the first peak of the health crisis

#### FOURNIER Alicia^1^, DELTOUR Victoire^1,2^, LHEUREUX Florent^3^, ECARNOT Fiona^4^, BINQUET Christine^5,6^, QUENOT Jean Pierre^5,7,8,9^, LAURENT Alexandra^1^

##### ^1^Laboratoire Psy-DREPI, Université de Bourgogne, Dijon, France; ^2^VCR EA 7403, Ecole de Psychologues Praticiens, ICP, Paris, France; ^3^Laboratoire de Psychologie, Université de Franche-Comté, Besançon, France; ^4^Département de cardiologie, Université de Franche-Comté, Besançon, France; ^5^INSERM, CIC1432, module Epidémiologie Clinique (CIC-EC), Dijon, France; ^6^CHU Dijon-Bourgogne, UFR des Sciences de Santé, Dijon, France; ^7^Service de Médecine Intensive-Réanimation, CHU Dijon-Bourgogne, Dijon, France; ^8^Equipe Lipness, centre de recherche INSERM UMR1231 et LabEx LipSTIC, Université de Bourgogne, Dijon, France; ^9^Espace de Réflexion Éthique Bourgogne Franche-Comté, Dijon, France

###### Correspondence: Alicia FOURNIER (alicia.fournier@u-bourgogne.fr)

*Annals of Intensive Care* 2022, **12(1):**FC-198

**Rationale:** The health crisis linked to COVID-19 has significantly changed the organization and the context of care in intensive care units (ICU). New and numerous stressors have been added to the stressors already present in the units (1). One of the specificities of this crisis is notably its duration and its uncertain outcome. In this context, acute and chronic stress have been combined, constituting the risk of developing stress-related disorders that become chronic in ICU professionals. The objective of this paper is to present the psychological impact of the health crisis on intensive care workers 1 year and 2 months after its first peak in France.

**Patients and methods/Materials and methods:** Between 21 June and 19 July 2021, 840 professionals (physicians, residents, nurses, nurses’ aides, students, and nursing managers) in 48 French hospitals took part in the PsyCOVID-ICU study. An online questionnaire was administered online via the Limesurvey platform. We assessed the severity of PTSD symptoms (IES-R), burnout (MBI-HSS), perceived stressors (PS-ICU) and the support used to cope with professional stress (colleagues, hierarchy, entourage, hotline, psychologist).

**Results:** The results show that 60.24% of the professionals had burnout. Of these, 16.38% also had possible PTSD. Only 2.4% of the professionals had possible PTSD without burnout. Professionals who suffered from both possible PTSD and burnout perceived significantly more stress than other professionals (*p*s < 0.002). They were particularly sensitive to the perception of a heavy workload and difficulties related to human resources management. Most of these professionals turned to support from their colleagues and relatives (*p*s < 0.025) and the least to the hotline (*p*s < 0.001).

**Conclusion:** This study illustrates the long-term effects of the health crisis on the mental health of intensive care professionals. It highlights a population of ICU professionals who are particularly sensitive to stress in the ICU one year after the crisis and who suffer from post-traumatic stress and burnout.

**Reference 1:** Laurent A., Fournier A., Lheureux F, …. Quenot JP. (2021). Mental health and stress among ICU caregivers in France according to intensity of the COVID-19 epidemic. Annals of intensive care. 4;11(1):90. https://doi.org/10.1186/s13613-021-00880-y.

**Compliance with ethics regulations:** Yes in clinical research.

### FC-199 Prediction of the location of infectious droplets and aerosols with computational fluid dynamics in the rooms of ICU patients

#### DE HARENNE Charlotte^1^, GAMBALE Alessandro^2^, BOSSE Maxime^3^, LISSON Maxime^4^, LAMTIRI Mouhsine^3^, PITTI Pauline^3^, OBANDO VEGA Pedro^2^, BERTRAND Axelle^1^, MAHIANE-BIDJA Anne^1^, KESSELER Sophie^1^, MEEX Cécile^3^, HAUBRUGE Eric^4^, SAEGERMAN Claude^5^, DESMECHT Daniel^6^, MISSET Benoît^1^

##### ^1^Department of Intensive Care Medicine - CHU Liege, Liege, Belgique; ^2^BUILDWIND SPRL, Brussels, Belgique; ^3^Department of Microbiology - CHU LIEGE, Liege, Belgique; ^4^Terra Research Center - Gembloux Agrobiotech - University of Liege, Gembloux, Belgique; ^5^Research Unit in Epidemiology and Risk Analysis - Faculty of Veterinary Medicine - University of Liege, Liege, Belgique; ^6^Department of Pathology - Faculty of Veterinary Medicine - University of Liege, Liege, Belgique

##### Correspondence: Benoît MISSET (benoit.misset@chuliege.be)

*Annals of Intensive Care* 2022, **12(1):**FC-199

**Rationale:** SARS-CoV 2 is likely transmitted between humans through direct contact and/or infectious droplets and aerosols. Droplets are defined as particles over 5 µm and aerosols as particles less than 5 µm diameter. Our aim was to estimate the location of the particles within a real intensive care room when hosting a patient with COVID-19 and assess the presence of SARS-CoV-2 in these particles.

**Patients and methods/Materials and methods:** We used the architectural features of several individual rooms of our Intensive Care department, the prescribed and measured heating, ventilation, and air-conditioning characteristics. We modeled the air flow and droplet dynamics using Computational Fluid Dynamics. Unsteady RANS, three-dimensional numerical simulations used a k-w SST turbulence model with heat transfer and buoyancy for natural convection. The computational domain included the room ventilation system with piping and vents, air leakage from the doors, and the main objects in the rooms. Air and surface samples for SARS-CoV-2 PCR were guided by the simulation.

**Results:** The measured HVAC characteristics in terms of air flow were different than the prescribed one. Simulations showed that droplets were mostly present on the workbenches and on the floor in front of the door, and that aerosols flew vertically towards the ceiling, stayed in suspension or followed the leakage around the doors. SARS-CoV-2 was searched in the rooms of 9 patients treated with high flow oxygen and nasal Ct between 17 and 31. PCR was positive in 10/25 (40%) surface and 3/15 (20%) air samples where it was expected by simulation, and in 1/5 (20%) surface and 0/12 air samples where it was not expected. Positive surfaces were air-extract units, the floor in front of the door and the nurse workbench. Positive air samples were over the patient’s head and in front of the door outside the room. Additional samples are underway to increase the prediction of the computational model.

**Conclusion:** In our institution, the observed HVAC characteristics are different from the prescribed ones. Taking the actual characteristics of the rooms into account and using a numeric 3-D simulation model, the use of a computational model accurately predicts locations where SARS-Cov-2 particles can be found.

**Compliance with ethics regulations:** Yes in clinical research.
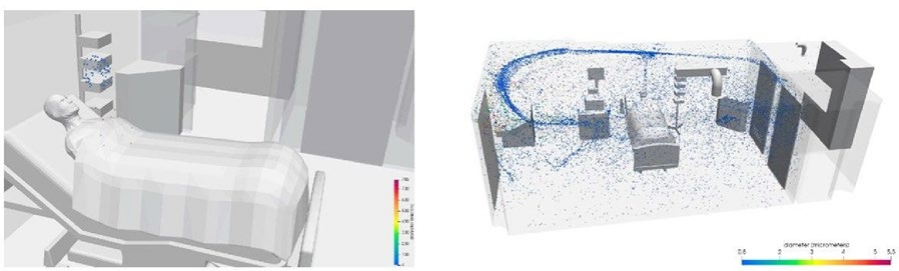


*Numeric 3-D simulation model*

### FC-200 Impact of emotional stimulations on thirst in critically ill mechanically ventilated patient

#### BUREAU Côme^1^, SIMILOWSKI Thomas^1^, DEMOULE Alexandre^1^

##### ^1^Hôpital Universitaire Pitié-Salpêtrière, Paris, France

###### Correspondence: Côme BUREAU (come.bureau@aphp.fr)

*Annals of Intensive Care* 2022, **12(1):**FC-200

**Rationale:** Up to 70% of intensive care unit patients report thirst. Thirst provokes the urge to drink fluids. This multidimensional symptom is described in terms of intensity and dry mouth. Few studies have investigated thirst relief in intensive care unit patients and none has focused on mechanically ventilated (MV) patients. Relieving thirst usually involves giving the patient something to drink; unfortunately, in this population, it is difficult to meet this need and alternative interventions are needed. Our objective was to evaluate and to compare the impact of auditory and a sensory stimulations on the intensity of thirst in critically ill patients undergoing invasive or non-invasive MV. This is an ancillary study of the “Sensopnea 2” study.

**Patients and methods/Materials and methods:** Patients MV for more than 48 h with a dyspnea intensity ≥ 40 mm on a visual analog dyspnea scale (Dyspnea-VAS) were included (inclusion criteria of the Sensopnea 2 study). Two visual analog scales (Thirst-VAS and Dry mouth-VAS) assessed thirst and dry mouth sensation. We studied the following interventions: 1) a relaxing standardized music piece vs. a "pink" noise as a control (auditory stimulation), and 2) fresh air directed toward the face vs. the thigh as a control (sensory stimulation). A washout period separated each condition.

**Results:** We included 46 patients, 19 tracheostomized (41%), 18 intubated (39%) and 9 under non-invasive ventilation (20%). Median (interquartile range) age was 63 years (54–73) and duration of mechanical ventilation was 33 days (7–49). Figure 1 shows Thirst-VAS and Dry mouth-VAS during sensory stimulation and auditive stimulation with their control. Compared to their respective control group, the two interventions decreased Thirst-VAS: 1) auditory stimulation 20 [0–30] vs. 30 [0–40], p = 0.0004 and 2) sensory stimulation 30 [20–50] vs. 40 [30–50], p = 0.0025). Compared to their respective control group the two interventions decreased Dry mouth-VAS: 1) auditory stimulation 30 [10–40] vs. 40 [30–70], p = 0.0002 and 2) sensory stimulation 30 [0–60] vs. 40 [30–60], p = 0.0013).

**Conclusion:** In critically ill dyspneic MV patients, auditory and sensory stimulations decrease thirst and dry mouth sensation.

**Compliance with ethics regulations:** Yes in clinical research.
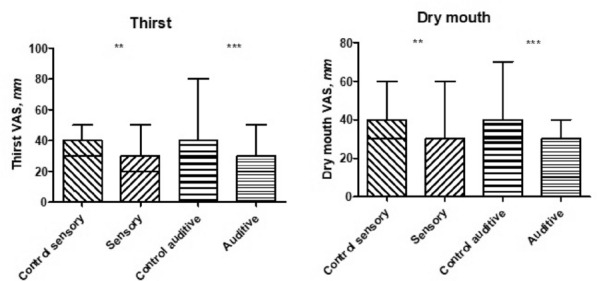


VAS Visual analogue scale **p < 0.01, ***p < 0.001

### FC-201 Prevalence of low vitamin D level and outcome impact in weaning unit after prolonged stay in ICU

#### PERETOUT Jean-Baptiste^1^, NSSAIR Karim^1^, ABDALLAH Razach^1^, LEPECQ Raphael^1^, DONETTI Laurence^1^, LAISSI Mohamed^1^, CHOUKROUN Gérald^1^

##### ^1^Hôpital Forcilles - Fondation Cognacq-Jay, Férolles-Attilly, France

###### Correspondence: Jean-Baptiste PERETOUT (jperetout@cognacq-jay.fr)

*Annals of Intensive Care* 2022, **12(1):**FC-201

**Rationale:** Recent studies suggest that low vitamin D level is associated with an increased risk of hospitalization, critical care use, enhanced systemic inflammation and mortality^1,2^. The objective of this study was to determine the prevalence of hypovitaminosis D and vitamin D deficiency in patients hospitalized in a respiratory weaning unit and the impact on weaning outcomes after a prolonged ICU stay.

**Patients and methods/Materials and methods:** This is a prospective, descriptive cohort study of patients hospitalized in respiratory weaning unit after a prolonged ICU stay over the year 2021. Plasma 25-hydroxyvitamin D concentrations ([25(OH)D]) was collected from the 48-h admission blood test. We defined hypovitaminosis D by [25(OH)D] < 30 ng/mL and vitamin D severe deficiency by [25(OH)D] < 10 ng/mL. We also compared the biological data and weaning outcomes of patients with hypovitaminosis D and patients with normal levels.

**Results:** We analyzed 136 patients (mean age 63 ± 12 years; male 70%; COVID 70%) hospitalized in a weaning unit for mechanical ventilation and/or tracheostomy after prolonged stay in the ICU. On admission, the prevalence of hypovitaminosis D was 88.3% (n = 120), the prevalence of severe deficiency was 3.7% (n = 5) and the mean [25(OH)D] was 20.7 ± 7.9 ng/mL. No statisticial difference on [25(OH)D] levels was found between patients with successful decannulation and those with failed decannulation: 20.7 ± 8.3 vs 20.7 ± 5.6 ng/mL (p = 0.98). In hypovitaminosis D group, phosphorus was statistically lower than in the other group: 1.08 ± 0,32 vs 1.25 ± 0,27 p = 0.035. No statistical difference was found on weaning outcomes (Table I).

**Conclusion:** The prevalence of hypovitaminosis D is major in weaning unit after a prolonged ICU stay. Among the other biomarkers, only phosphorus is lower in the deficient group probably related to its role in the metabolism of vitamin D. However, vitamin D deficiency does not appear to be a factor influencing weaning’s outcomes. Screening and treatment are still necessary in the treatment of long-term complications.

**Reference 1:** Bychinin MV, Klypa TV, Mandel IA, Andreichenko SA, Baklaushev VP, Yusubalieva GM, Kolyshkina NA, Troitsky AV. Low Circulating Vitamin D in Intensive Care Unit-Admitted COVID-19 Patients as a Predictor of Negative Outcomes. J Nutr. 2021 Aug 7;151(8):2199–2.

**Reference 2:** Pimentel GD, Dela Vega MCM, Pichard C. Low vitamin D levels and increased neutrophil in patients admitted at ICU with COVID-19. Clin Nutr ESPEN. 2021 Aug;44:466–468.

**Compliance with ethics regulations:** Yes in clinical research.
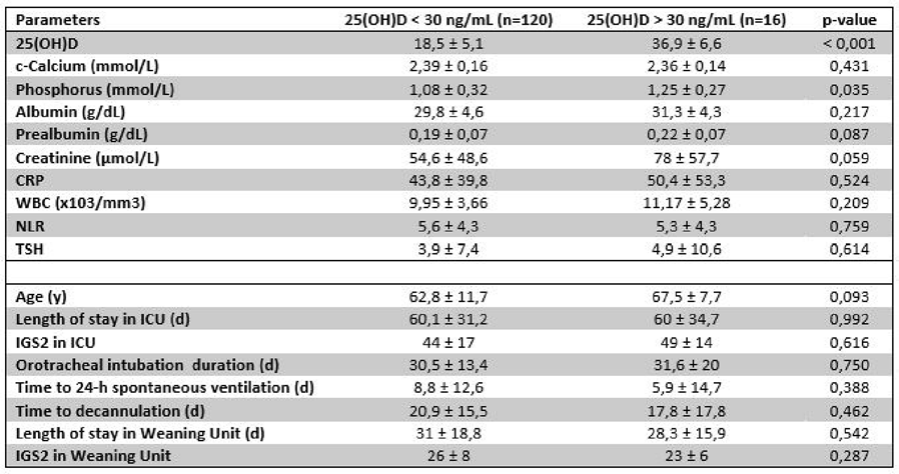


*Table I Biomarkers and weaning outcomes in hypovitaminosis D group and normal vitamin D group.*

### FC-202 Impact of time of administration and dose of desmopressin in brain-dead donors: a before-after study

#### MOLINA Laura^1^, GENEIX Mario^1^, LE PRINCE Marine^1^, MOSCHIETTO Sebastien^1^, MONTINI Florent^1^

##### ^1^Centre hospitalier d'Avignon, Avignon, France

###### Correspondence: Laura MOLINA (molina.laura@hotmail.fr)

*Annals of Intensive Care* 2022, **12(1):**FC-202

**Rationale:** Diabetes insipidus is one of the complications of brain death. Its management consists in the introduction of desmopressin. There are no studies focusing on the dosage and timing of desmopressin administration in brain dead organ donors. It's possible that the earliest administration of desmopressin in relation to the diagnosis of central diabetes insipidus and at high doses may influence the hemodynamics of donors, their renal function and recipient in the short and long term. The objective of our study is to compare, after introducing a new protocol in 2018, the modalities of desmopressin administration in terms of time of introduction and dosage and then the impact on hemodynamics, donor renal function, and the impact on renal function of renal transplant recipients.

**Patients and methods/Materials and methods:** We conducted a single center, retrospective study. A total of 196 patients were included, with a diagnosis of brain death, during a period of 6 years. The protocol used during the first period between 2015 and 2017 was, after the diagnosis of diabetes insipidus, the infusion of a bolus of 1 µg of desmopressin, then 1ug every 6 h according to evolution. The new protocol was infusion of a bolus of 2 µg of desmopressin then continuous infusion of 4 µg/24 h, with the possibility of additional boluses. The data on the recipients was sent to us by the biomedicine agency.

**Results:** We found a statistically significant difference in the delay of desmopressin introduction (4H group 2015–2017 vs. 1H group 2018–2020), as well as in the total dosage administered (1 µg 2015–2017 vs. 4 µg 2018–2020). We found a significant difference in the administration of glucose solution (4L group 2015–2017 vs 2.5L group 2018–2020), as well as a significant difference in the dosages of noradrenaline administered (0.2 gamma/kg/min group 2015–2017 vs 0.1 gamma/kg/min group 2018- 2020 p = 0.04). We noted higher natremia in the first group, not significant (148 mmol/l group 2015–2017 vs 146 mmol/l group 2018–2020). However, we did not find any differences in the renal functions of the donors or recipients. The number of functional grafts at 1 year was higher in the 2018–2020 group.

**Conclusion:** This study, the first to compare 2 methods of desmopressin administration in brain-dead donors. It shows that increasing dosages and reducing the time of administration is safe, allows better haemodynamic management of donors and may improve long-term renal transplant outcomes.

**Compliance with ethics regulations:** Yes in clinical research.
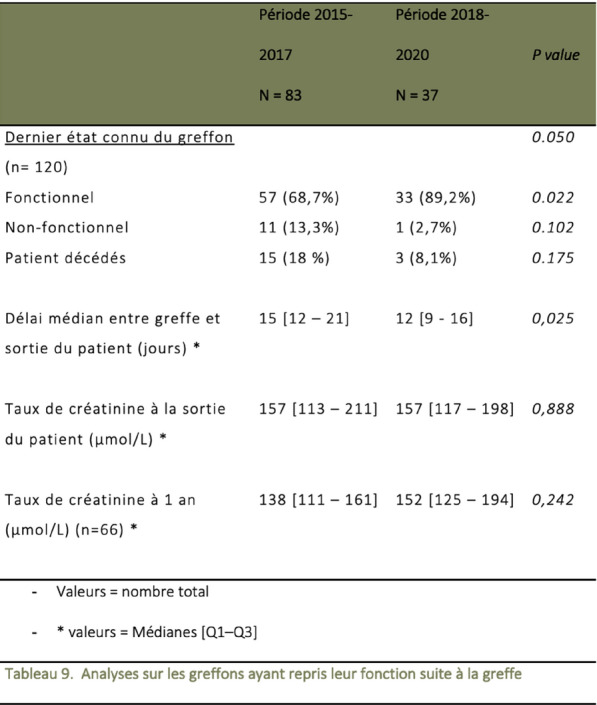


*Analysis of the grafts that have regained their functions*

### FC-203 Right atrial strain, renal doppler and worsening renal function in acute decompensated pulmonary hypertension

#### PICHON Jérémie^1^, ROCHE Anne^1^, EBSTEIN Nathan^1^, BOUCLY Athenais^1^, CORTESE Jonathan^1^, JEVNIKAR Mitja^1^, JAIS Xavier^1^, SITBON Olivier^1^, MONTANI David^1^, HUMBERT Marc^1^, SAVALE Laurent^1^

##### ^1^Hôpital Bicêtre APHP, Kremlin Bicêtre, France

###### Correspondence: Jérémie PICHON (jeremie.pichon1@gmail.com)

*Annals of Intensive Care* 2022, **12(1):**FC-203

**Rationale:** Although congestion has been identified as a main contributing element to the cardio-renal syndrome in acute right heart failure (RHF), right atrial (RA) mechanical performance has never been evaluated to predict worsening renal failure.

**Patients and methods/Materials and methods:** The aim of our study was to analyze the prognostic value of RA Strain in precapillary pulmonary hypertension (PH) patients admitted in intensive care unit (ICU) for acute RHF at admission and at day three. Congestion was assessed by alteration of the intrarenal Doppler, sushepatic and portal vein flow. The primary endpoint was the occurrence of acute kidney injury (AKI) KDIGO I–III.

**Results:** Among the 64 patients analyzed (66% female, age 60 ± 16 years), all patients received intravenous diuretics and 36 (56%) required inotropic and/or vasopressor support. At ICU admission, the median values of peak, conduit, boost RA Strain and RA ejection fraction (EF) were respectively 12% [8–20], − 5% [7–4], − 7% [10–4] and 31% [20–41]. There is a good interobserver correlation (Rho = 0.94; p < 0.01). Peak RA strain higher than median value was significantly associated with higher eGFR (p = 0.02), lower renal resistive index (RRI; p = 0.01), lower renal venous stasis index (RVSI; p < 0.01) and lower volume excess ultrasound (VExUS; p < 0.01). A primary endpoint event occurred in 17 patients (26%). Event rate was higher in low RA EF (p = 0.02), in high RRI (p = 0.01) and high RVSI (p < 0.01) groups. At day 3, an increase in peak RA Strain and RA EF was both associated with fewer worsening of renal function. Patients with peak RA Strain > 20% at admission does not develop AKI during the stay.

**Conclusion:** RA Strain seems to be an easy-to-use bedside and relevant tool for evaluation of cardio-renal syndrome. It could help to predict worsening renal function in acute RHF.

**Compliance with ethics regulations:** Yes in clinical research.

### FC-204 Permissive hypotension during sepsis: impact on fluid balance and renal prognosis

#### BLUM Laurene^1^, LAVILLEGRAND Jean-Rémi^1^, HARIRI Geoffroy^1^, URBINA Tomas^1^, BIGE Naike^1^, BAUDEL Jean Luc^1^, GUIDET Bertrand^1^, MAURY Eric^1^, AIT OUFELLA Hafid^1^

##### ^1^APHP-CHU Saint Antoine, Paris, France

###### Correspondence: Jean-Rémi LAVILLEGRAND (jrlavillegrand@gmail.com)

*Annals of Intensive Care* 2022, **12(1):**FC-204

**Rationale:** The therapeutic management of sepsis includes control of the infectious source, fluid resuscitation associated with the introduction of catecholamines, primarily norepinephrine to maintain a mean arterial pressure (MAP > 65 mmHg). However, this MAP target remains debated, especially since MAP is not a direct reflection of tissue perfusion. Some authors suggest that MAP should be adapted individually to tissue perfusion. The objective of our work is to evaluate if a strategy called permissive hypotension, which consists in tolerating a moderate arterial hypotension when peripheral perfusion is preserved, has an impact on the fluid balance and on the renal function.

**Patients and methods/Materials and methods:** This was a single-center observational study. Patients with sepsis and MAP < 65 mmHg after 30 mL/kg fluid resuscitation were included. We compared 2 groups of patients, a so-called Septic Shock group that received norepinephrine to maintain MAP > 65 mmHg and a Permissive Hypotension group in whom moderate hypotension was tolerated. The volume of fluids administered and the renal outcome (diuresis and change in creatinine) during the first days of management were studied.

**Results:** Over a period of 1 year, 94 patients were included, 79 in the septic shock group and 15 in the permissive hypotension group. Severity, assessed by SAPS 2 (60 [45–86] vs 45 [27–46], P = 0.0001) and SOFA score (7 [5–10] vs 4 [1–6], P < 0.0001), was greater in the Septic Shock group, as was organ support: invasive mechanical ventilation (71% vs 0%, P < 0.0001) and extra-renal replacement therapy (20% vs 0%, P = 0.06). In univariate analysis, mortality was lower in the Permissive Hypotension group (0% vs. 38%, P = 0.004) as was length of stay among survivors (3 [2–5] vs. 7 [4–14] days, P = 0.0004). The volume of fluid administration before admission to the ICU did not differ between the 2 groups (26 [20–32] vs 24 [18–33] mL/kg, p = 0.8. In contrast, during the first 6 h in the ICU, the volume administered was significantly lower in the Hypotensive Permissive group (500 [0–1000] versus 1000 [500–1500], p = 0.03). Renal function improved faster in the Permissive Hypotension group with a significantly lower KDIGO score at 48 h (0 [0–1] vs. 1 [0–2] (p < 0.05). Blood creatinine levels were not different at discharge between the 2 patient groups.

**Conclusion:** Permissive hypotension in sepsis, in the absence of clinical peripheral perfusion abnormalities, is very well tolerated. It is not accompanied by excess vascular filling or negative impact on renal function.

**Compliance with ethics regulations:** Yes in clinical research.

### FC-205 Soluble TREM-1 plasma levels are associated with acute kidney injury, acute atrial fibrillation and prolonged ICU stay after cardiac surgery

#### VANDESTIENNE Marie^2^, BRAIK Rayan^1,2^, LAVILLEGRAND Jean-Rémi^1,2^, EL RIFAI Rida^2^, HARIRI Geoffroy^1^, RAIA Lisa^1^, DEMAILLY Zoe^3^, TARTOUR Eric^1,2^, BEN HAMOUDA Nadine^1,2^, TEDGUI Alain^2^, BOUGLÉ Adrien^1^, TAMION Fabienne^3^, CLAVIER Thomas^3^, AIT-OUFELLA Hafid^2^

##### ^1^APHP, Paris, France; ^2^PARCC INSERM, Paris, France; ^3^CHU Rouen, Rouen, France

###### Correspondence: Rayan BRAIK (rayan.braik@aphp.fr)

*Annals of Intensive Care* 2022, **12(1):**FC-205

**Rationale:** Cardiopulmonary bypass (CPB) during cardiac surgery leads to deleterious systemic inflammation. We hypothesized that TREM-1, a myeloid receptor, is engaged during CPB and shed in plasma.

**Patients and methods/Materials and methods:** During non-urgent cardiac surgery, we assessed 1/the kinetics of myeloid TREM-1 expression (flow cytometry), 2/the relationship between soluble (s)TREM-1 and pro-inflammatory cytokine plasma levels (ELISA) and 3/the association between sTREM-1 levels and postoperative outcome.

**Results:** In a first pilot study (N = 11), TREM-1 expression on neutrophils decreased between H0 and H2 while sTREM-1 plasma levels increased. In a second prospective cohort (N = 46), sTREM-1 levels increased at H2 and at H24 (p < 0.001). IL-6, IL-8, G-CSF and TNF-a, but not IL-1b, significantly increased at H2 compared to H0 (p < 0.001), but dropped at H24. Principal component analysis showed a close relationship between sTREM-1 and IL-8. Three patterns of patients were identified: Profile 1 with high baseline sTREM-1 levels and high increase and profile 2/3 with low/moderate baseline sTREM-1 levels and no/moderate increase overtime. Profile 1 patients developed more severe organ failure after CPB, with higher norepinephrine dose, higher SOFA score and more frequently acute kidney injury at both H24 and H48. Acute atrial fibrillation was also more frequent in profile 1 patients at H24 (80% vs 19.4%, p = 0.001). After adjustment on age and duration of CPB, sTREM-1 levels at H2 remained associated with ICU and hospital length of stay.

**Conclusion:** Early changes in sTREM-1 levels after cardiac surgery identified patients at high risk of post-operative complications and prolonged length of stay.

**Compliance with ethics regulations:** Yes in clinical research.

### FC-206 Evaluation of balanced crystalloids on early renal recovery in patients with septic shock and continuous veno-venous hemofiltration: a retrospective observational study

#### TETE Xavier^1^, GRIFFIER Romain^2^, PEARSON Marie-Fleur^2^, TRAN VAN David^1^

##### ^1^HIA Robert Picqué, Villenave D'ornon, France; ^2^CHU de Bordeaux, Bordeaux, France

###### Correspondence: Xavier TETE (xavier.tete@hotmail.fr)

*Annals of Intensive Care* 2022, **12(1):**FC-206

**Rationale:** The impact on renal function of balanced crystalloids versus normal saline has been extensively studied in recent randomised trials (1). However, few data are available regarding their impact on renal recovery, particularly in patients admitted to the ICU and receiving renal replacement therapy at admission. The objective of our study was to evaluate the impact of balanced crystalloids versus normal saline on renal recovery in patients admitted to the ICU for septic shock complicated by acute renal failure requiring continuous hemofiltration.

**Patients and methods/Materials and methods:** We conducted a single-center retrospective study including patients hospitalized in the intensive care unit between 2014 and 2019 who presented a septic shock with acute kidney injury requiring renal replacement therapy (RRT) within the first 48 h. The patients included were classified into two groups according to their exposure to balanced crystalloids. The primary endpoint was early renal recovery defined as definitive weaning from RRT by day 7.

**Results:** Of the 91 patients included in the study, 38 were classified in the balanced crystalloid (BCs) group and 53 in the unbalanced crystalloid (UBCs) group. The median volume of balanced crystalloids administered during the first 7 days was 0 ml (IQR [0;500]) in the UBCs group and 2000 ml (IQR [1312.5;2500]) in the BCs group (p < 0.001). The total volume of fluids administered was not statistically different between the BCs group and the UBCs group (p = 0.67). On day 7, 47.4% of patients in the BCs group recovered versus 58.5% in the UBCs group (p = 0.29). There was no difference after adjustment on confounding factors in multivariate analysis. The mean duration of RRT, maximum chloruremia, and ICU mortality were not significantly different between the two groups. In multivariate analysis, a positive fluid balance lower than 5% of baseline weight was associated with better renal recovery (OR 0.35; [0.13; 0.9]).

**Conclusion:** Our study did not find a benefit to the use of balanced crystalloids on renal recovery in our population of patients with septic shock complicated by AKI requiring early continuous hemofiltration. Further studies are needed to determine whether using balanced cristalloids improves renal recovery when renal replacement therapy is required.

**Reference 1:** Semler MW, Self WH, Wanderer JP, Ehrenfeld JM, Wang L, Byrne DW, et al. Balanced Crystalloids versus Saline in Critically Ill Adults. N Engl J Med. mars 2018;378(9):829–39.

**Compliance with ethics regulations:** Yes in clinical research.

### FC-207 Risk factors for mortality in anemic patients in the intensive care unit

#### BENHAMZA Sabah^1^, LAZRAQ Mohamed^1^, ATAK Badreddine^1^, BENSAID Abdelhak^1^, MILOUDI Youssef^1^, EL HARRAR Najib^1^

##### ^1^Hôpital 20 Août 1953, CHUIR, Casablanca, Maroc

###### Correspondence: Sabah BENHAMZA (benhamzasabah5@gmail.com)

*Annals of Intensive Care* 2022, **12(1):**FC-207

**Rationale:** Anemia affects about two thirds of patients at admission to the ICU (1), and may lead to transfusions that may cause complications. Our objective was to specify the risk factors of mortality of anemic patients in our department.

**Patients and methods/Materials and methods:** Retrospective cohort over 1 year, in the intensive care unit of the 20 August hospital of Casablanca. All patients with anemia below 13 g/dl for men and 12 g/dl for women were included and excluded those coming from the hematology department or followed for a hematopathy.

**Results:** Out of 442 hospitalizations, 160 were included. The incidence of anemia was 36%. The mean age was 45 years, sex ratio M/F was 1.58. 26% were admitted for postoperative cephalic surgery and 12% for diabetic ketoacidosis. 82% of the patients were anemic at admission. Clinical signs were dominated by tachycardia (59%) and neurological disorders in (41%). Ferritinemia was collapsed in 39%, the infectious workup was positive with CRP and PCT elevated in 72% and 78% of cases respectively. 49% were transfused with red blood cells. 73% stayed less than 7 days and 15% more than 14 days. At discharge, 40% of the patients exceeded the threshold of 13 g/dl. 24% of our patients died. In multivariate analysis, the degree of anemia was not a risk factor for mortality, unlike SOFA score > 5 (OR 6.9 IC95% [2.7–17.4]) and blood transfusion which was a protective factor (OR = 0.3 IC 95% [0.2–0.7]).

**Conclusion:** The degree of anemia was not associated with mortality in our study, contrary to the SOFA score, because it is probably the comorbidities and the causal pathology that cause death.

**Reference 1:** 1- Zittoun J. Anémies macrocytaires carentielles. EMC 13–001-10 n.d.

**Compliance with ethics regulations:** Yes in clinical research.

### FC-208 Thombotic microangiopathy in ICU: a retrospective study of 20 years

#### KHARRAT Sana^1^, TURKI Olfa^1^, CHTARA Kamilia^1^, BRADAII Sabrine^1^, BAHLOUL Mabrouk^1^, BOUAZIZ Mounir^1^

##### ^1^CHU Habib bourguiba Sfax, Sfax, Tunisie

###### Correspondence: Olfa TURKI (olfa.turki.rea@gmail.com)

*Annals of Intensive Care* 2022, **12(1):**FC-208

**Rationale:** Thrombotic microangiopathy (TMA) is a rare condition. It covers a group of related disorders characterized by thrombosis in the micro-vascularization and by consequence organ dysfunction. It is responsible for significant morbidity and mortality. However, only a few reports have been published so far. The aim of the study was to describe our cohort of TMA in terms of incidence, clinical and-biological presentation, management and outcome. We also aimed to identify factors associated with poor prognosis.

**Patients and methods/Materials and methods:** Our study was retrospective, conducted in an intensive care unit. The study period was over 20 years: from January 1, 2001 to December 31, 2020. Our study included a descriptive part observing the different epidemiological, clinical, biological and characteristics of patients admitted for TMA and their evolution. The second part consisted on an analytical study highlighting the poor prognostic factors in our patients.

**Results:** A total of 37 patients were included. The incidence of TMA in the ICU was 0.18% with a female predominance and an average age of 34.8 years. Hemolytic anemia and thrombocytopenia were present in all patients. Neurological manifestations were the most frequent clinical signs (81.1%). Thrombotic thrombocytopenic purpura occupied ¾ of TMA’s forms in our cohort. TMA was associated with pregnancy in 45.9% of cases. Medical support was based on plasma exchange (70.3%) in association with corticosteroids and immunoglobulins. Follow-up of the patient showed an exacerbation rate of 18.9% and complications such as infections in 45.9% of cases. Mortality rate was 29.7%. Factors correlated with poor prognosis in our cohort were age > 36 years, Glasgow score < 9 and platelet count < 14,000 elts/mm^3^ at admission.

**Conclusion:** TMA is still a cause of high mortality. A better knowledge of this disease and the awareness of physicians as well as the development of diagnostic methods could improve the prognosis.

**Compliance with ethics regulations:** Yes in clinical research.

### FC-209 Impact of French lyophilized plasma transfusion in Trauma Center for severe civilian trauma patients

#### DELAMAIRE Flora^1^, LEBOUVIER Thomas^1^

##### ^1^CHU Rennes, Rennes, France

###### Correspondence: Flora DELAMAIRE (flora_d7@hotmail.com)

*Annals of Intensive Care* 2022, **12(1):**FC-209

**Rationale:** The initial management of severe trauma patients with hemorrhagic shock requires early blood products transfusion. Administration of high plasma: Red Blood Cell (RBC) ratio is associated with a reduction in mortality. French Lyophilized Plasma (FLYP) has been used for years for military prehospital care. We hypothesized that the use of FLYP for civilian severe trauma patients would reduce time for first plasma administration, reduce number of RBC transfused before first plasma and achieve a plasma:RBC transfusion ratio greater than 1:2.

**Patients and methods/Materials and methods:** This retrospective study was performed in a level 1 trauma center from January 2015 to June 2020. FLYP was available in trauma center since June 2017. Severe trauma patients who received massive transfusion (four RBC in the first hour and/or 10 RBC in the first 24 h) were included and divided in 2 groups according to the period of inclusion: Fresh Frozen Plasma (FFP) group before June 2017 and FLYP group after.

**Results:** Twenty-eight severe trauma patients were included in the FLYP group and 12 in the FFP group. The time until the first transfusion of plasma was shorter in the FLYP group, 35 min (IQR 20, 45) vs. 59 min (IQR 36.88) in the FFP group (p = 0.007). The number of RBC transfused before the first plasma was lower in the FLYP group than in the FFP group, respectively 2 vs. 3.67 (p = 0.002). The ratio plasma:RBC greater than 1:2 was obtained in 26 (93%) patients of the FLYP group vs 8 (67%) in the FFP group (p = 0.05).

**Conclusion:** FLYP permitted an early transfusion of plasma during the massive transfusion of severe trauma patients. It significantly reduced time for in hospital plasma delivery and improved the order of blood products transfusion. It could permit to obtain a greater than 1:2 plasma:RBC ratio in the trauma center. These results should be confirmed by a randomized trial.

**Compliance with ethics regulations:** N/A.
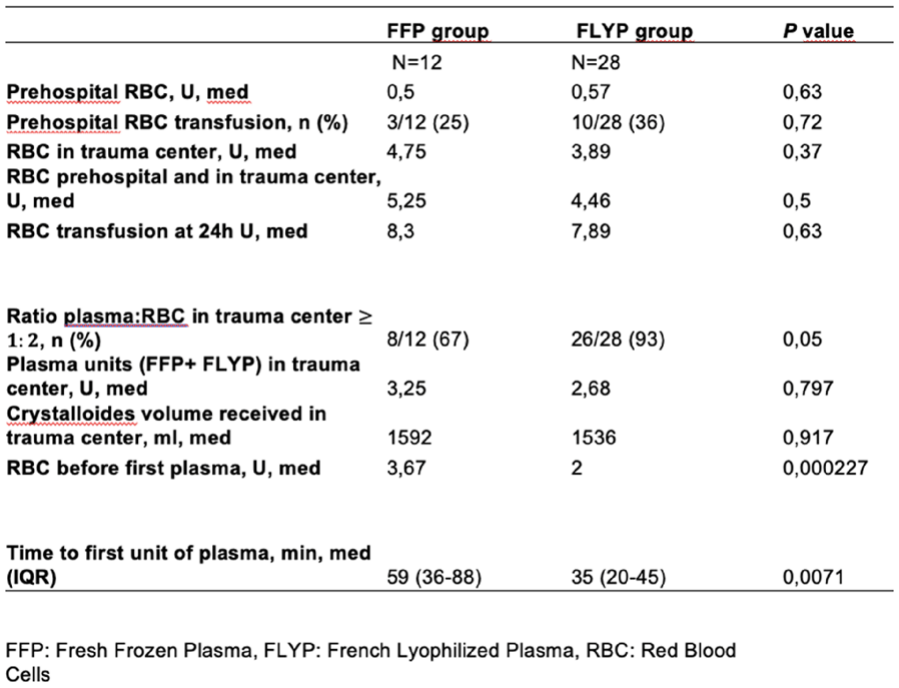


*Transfusion data: comparison of the FFP group and the FLYP group*

### FC-210 Storage time of transfused packed red blood cells and mortality in critically ill patients: a retrospective study

#### BEN DABEBISS Rafla^1^, MAHMOUD Jihène^1^, CHOUCHÈNE Salma^1^, ZOUARI Hajer^1^, BOUATAY Amina^1^, HMOUDA Houssem^1^

##### ^1^Sahloul University Hospital, Sousse, Tunisie

###### Correspondence: Rafla BEN DABEBISS (rafla_bendabebiss@hotmail.com)

*Annals of Intensive Care* 2022, **12(1):**FC-210

**Rationale:** Anemia is frequently diagnosed in intensive care. It may be present at admission or may develop during ICU stay due to blood loss secondary to frequent blood sampling in most of the cases. Red blood cell (RBC) transfusions may be lifesaving in some circumstances. However, several studies have reported increased morbidity and mortality in transfused patients due to “storage lesions” of older blood. This study focuses on outcome of transfused patients according to storage time of RBCs, and compares transfusion yield between older and newer blood.

**Patients and methods/Materials and methods:** This is a retrospective descriptive and analytical study of patients admitted between January 2014 and January 2021, who received RBC transfusion in the ICU. Epidemiological, clinical, and laboratory data were collected, as well as the number of transfused packed RBCs, storage time (age) of each unit, and estimation of blood spoliation secondary to blood sampling during ICU stay.

**Results:** During the study period, 80 patients were concerned by 150 transfusion episodes, including 236 packed RBCs. The mean age of patients was 57.1 ± 18.5 years. All patients had co-morbid conditions. Acute respiratory failure was the most frequent diagnosis present in 65% of cases at admission. The APACHE II score was 20.4 ± 9.4, and SAPS II score was 48.5 ± 21.1. The mean hemoglobin level at admission was 9 ± 2.7 g/dl. The mean pre-transfusion hemoglobin level was 6.9 ± 1.1 g/dl. The mean time to first ICU transfusion was 7.7 ± 8.7 days. Age of RBCs was 1 to 5 days in 21 transfusion episodes (14%), 6 to 10 days in 46 episodes (30.7%), 11 to 19 days in 59 episodes (39.3%), and ≥ 20 days in 24 transfusion episodes (16%). Mortality rate among transfused patients was 47.5%, and was related to severity of illness. Patients who received older blood did not have a significantly higher mortality (p = 0.839), however the increment in hemoglobin per episode was significantly higher with newer blood (P = 0.001). The mean value of blood spoliation was 426.7 ± 387.9 ml, and the number of transfusion episodes increased with blood spoliation and length of ICU stay. There was no statistically significant link between blood spoliation and mortality.

**Conclusion:** Newer transfused blood resulted in significantly higher hemoglobin increment. However, RBCs storage time did not affect mortality according to univariate and multivariate analysis.

**Compliance with ethics regulations:** Yes in clinical research.

### FC-211 Survey on static physiologic parameters (single measure) that could guide red blood cell (RBC) transfusion practice in pediatric intensive care units (PICU), on behalf of the Pediatric Interest Group of the Canadian Critical Care Trials Group (CCCTG)

#### LACROIX Jacques^1^, GALLAND Anne^1^, TUCCI Marisa^1^, LETEURTRE Stéphane^2^, SARFATTI Avishay^3^, RAY Samiran^4^, STANWORTH Simon^5^, FONTELA Patricia^6^, KAWAGUCHI Atsushi^7^, DEMARET Pierre^8^, DUCRUET Thierry^9^, DU PONT-THIBODEAU Geneviève^1^

##### ^1^CHU Sainte-Justine, Montreal, Canada; ^2^Réanimation et Surveillance Continue Pédiatriques, Hôpital Jeanne de Flandre, CHRU Lille, Lille, Lille, France; ^3^Paediatric Intensivist and research lead for paediatric critical care, Department of Pediatrics, Oxford University Hospitals NHS Foundation Trust, Oxford, UK, Oxford, Royaume-Uni; ^4^Paediatric Intensivist, Department of Pediatrics, Great Ormond Street Hospital, London, Royaume-Uni; ^5^Transfusion Medicine, Department: Haematology, NHS Blood & Transplant/Oxford Radcliffe Hospitals, Oxford, Royaume-Uni; ^6^Pediatric Critical Care, Departments of Pediatrics and of Epidemiology, Biostatistics, and Occupational Health, The Montreal Children's Hospital, McGill University, Montreal, Canada; ^7^Pediatric Intensive Care Unit, Tokyo Women's Medical University, Department of Intensive Care Medicine, Tokyo, Japon; ^8^Pediatric intensive care unit, Department of Pediatrics, CHC Liège, Liège, Belgique; ^9^Unité de recherches cliniques appliquées, Research Centre, CHU Sainte-Justine, Montreal, Canada

###### Correspondence: Jacques LACROIX (jlacroix052@gmail.com)

*Annals of Intensive Care* 2022, **12(1):**FC-211

**Rationale:** To determine what single value of physiological parameter threshold would guide the RBC transfusion practice of pediatric intensivists, in addition to hemoglobin (Hb) concentration.

**Patients and methods/Materials and methods:** Scenario-based self-administered questionnaire e-mailed to pediatric intensivists in 2021. Five scenarios were considered: 1) a previously healthy 4-year-old boy mechanically-ventilated after a severe multiple traumas, 2) idem, but with a toxic shock syndrome and ARDS, 3) idem, but after ablation of hepatoblastoma; 4) 5-month-old boy admitted to PICU following corrective surgery for tetralogy of Fallot; 5) 3-week-old boy with hypoplastic left heart syndrome admitted to PICU after Norwood + Sano procedure. Patients were hemodynamically stable in all scenarios. Respondents addressed three questions: 1. What baseline Hb concentration would prompt you to prescribe an RBC transfusion in this scenario? 2. Do you use the physiologic parameter to guide your RBC transfusion practice? What threshold of the parameters would prompt you to give an RBC transfusion if Hb is 1 g/dL above the baseline Hb that you chose in 1st question?

**Results:** 132 among 235 eligible respondents (56%) from France (50), United Kingdom (36), Canada (16), Japan (15) and Belgium (11) filled the questionnaire. The mean baseline Hb thresholds chosen are detailed in Table 1. Two out of 7 physiologic parameters (ScvO_2_ and blood lactate) were used by more than 75% of respondents to guide their RBC transfusion practice (Table 1). The proportion of respondents who stated that they do not use some parameters was higher in the trauma case (range: 21% to 75%); it was lower for the other scenarios (range: 0% to 39%). The difference in use between the 5 scenarios was statistically significant for all parameters (P < 0.001). Table 1 also reports the threshold of 7 physiologic parameters used by respondents that would prompt them to prescribe an RBC transfusion if Hb is 1 g/dL above the baseline Hb concentration they chose. A ScvO_2_ threshold of 58% emerged in four scenarios, but not for the post-operative cyanotic cardiac patient. Respondents agreed on a blood lactate threshold of 4 mmol/L in all scenarios.

**Conclusion:** The proportion of respondents who do not use some parameters changed with different basic disease. Only ScvO2 and blood lactate were used by more than 75% of respondents to guide their RBC transfusion practice in PICU patients. Respondents seem to consider a ScvO_2_ threshold of 58% and blood lactate threshold of 4 mmol/L.

**Compliance with ethics regulations:** Yes in clinical research.
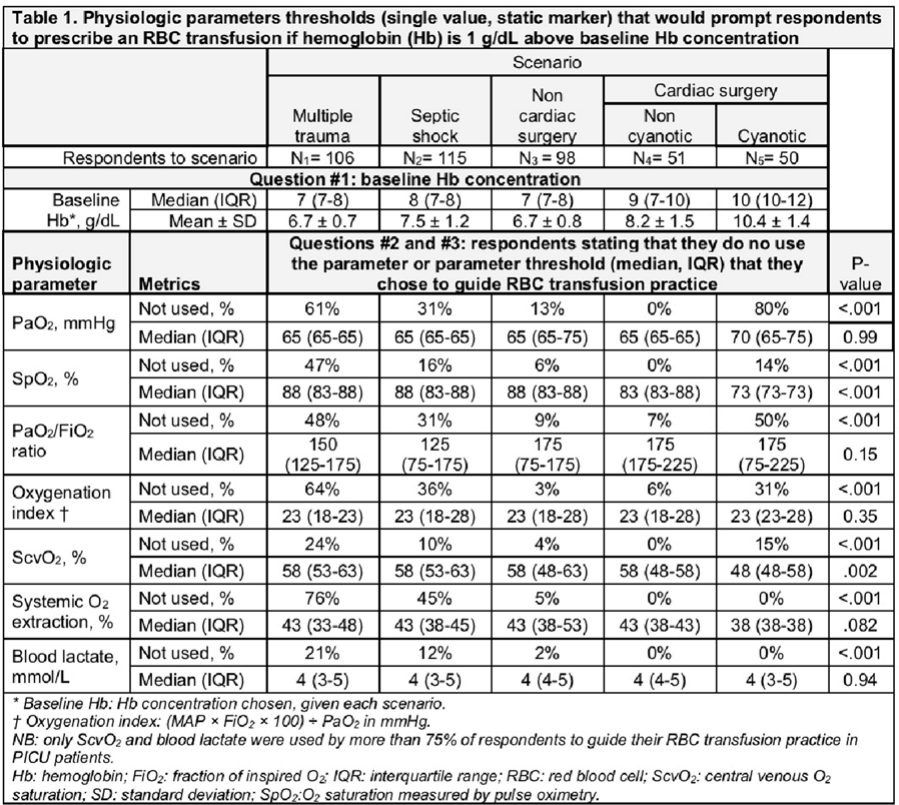


*Baseline Hb: Hb concentration chosen, given each scenario

### FC-212 Survey on how dynamic physiologic markers can modulate the hemoglobin threshold and guide red blood cell (RBC) transfusion practice in pediatric intensive care units (PICU), on behalf of the CCCTG, PCCS, JICRG and GFRUP

#### GALLAND Anne^1^, TUCCI Marisa^1^, LETEURTRE Stéphane^2^, SARFATTI Avishay^3^, RAY Samiran^4^, STANWORTH Simon^5^, FONTELA Patricia^6^, KAWAGUCHI Atsushi^7^, DEMARET Pierre^8^, DUCRUET Thierry^9^, LACROIX Jacques^1^, DU PONT-THIBODEAU Geneviève^1^

##### ^1^Division of Pediatric Critical Care Medicine, Department of Pediatrics, CHU Sainte-Justine, Université de Montréal, Montreal, Canada; ^2^Réanimation et Surveillance Continue Pédiatriques, Hôpital Jeanne de Flandre, CHRU Lille, Lille, France, Lille, France; ^3^Department of Pediatrics, Oxford University Hospitals NHS Foundation Trust, Oxford, Royaume-Uni; ^4^Department of Pediatrics, Great Ormond Street Hospital, London, Royaume-Uni; ^5^Transfusion Medicine, Department: Haematology, NHS Blood & Transplant/Oxford Radcliffe Hospitals, Oxford, Royaume-Uni; ^6^Pediatric Critical Care, Departments of Pediatrics and of Epidemiology, Biostatistics, and Occupational Health, The Montreal Children's Hospital, McGill University, Montreal, Canada; ^7^Pediatric Intensive Care Unit, Tokyo Women's Medical University, Department of Intensive Care Medicine, Tokyo, Japon; ^8^Pediatric intensive care unit, Department of Pediatrics, CHC Liège, Liège, Belgique; ^9^Unité de recherches cliniques appliquées, Research Centre, CHU Sainte-Justine, Université de Montréal, Montréal, Canada

###### Correspondence: Jacques LACROIX (jlacroix052@gmail.com)

*Annals of Intensive Care* 2022, **12(1):**FC-212

**Rationale:** To determine if the trajectory (2 sequential measures) of dynamic physiologic parameters modulates the Hb threshold and guides the stated RBC transfusion practice of pediatric intensivists, in addition to hemoglobin (Hb) concentration.

**Patients and methods/Materials and methods:** Scenario-based self-administered questionnaire e-mailed to pediatric intensivists in 2021. There were five scenarios: 1) previously healthy 4-year-old boy mechanically-ventilated after a severe multiple trauma; 2) idem with toxic shock syndrome and ARDS; 3) idem after ablation of hepatoblastoma; 4) 5-month-old boy admitted to PICU following corrective surgery for tetralogy of Fallot; 5) 3-week-old boy with hypoplastic left heart syndrome admitted to PICU after a Norwood + Sano procedure. Respondents addressed two questions: 1. What Hb concentration would prompt you to prescribe an RBC transfusion to the patient in the scenario? 2. What would be the lowest acceptable Hb value that would prompt you to transfuse the patients described in the scenario given the Hb concentration that you chose (question #1) and the sequential results of the following physiologic parameters?

**Results:** 132 participants among 235 eligible respondents (56%) from France (50), United Kingdom (36), Canada (16), Japan (15) and Belgium (11) filled the questionnaire. Mean and median baseline Hb threshold are reported in Table 1. Two parameters (ScvO_2_ and blood lactate) improved over time: the Hb threshold decrease was small in five scenarios for blood lactate and three scenarios for ScvO_2_ (range: − 0.2 ± 0.1 to 0.1 ± 0.9 g/dL), but it reached − 0.4 ± 1.1 and − 0.6 ± 0.7 g/dL for blood lactate in two cardiac scenarios. We did not find a statistically significant difference between scenarios for blood lactate (p = 0.87), but the difference was significant when ScvO_2_ increases from 50 to 70% (p < 0.001). Four parameters worsened over time. Compared to mean baseline Hb threshold, the revised mean Hb threshold for transfusion decreased by ≤ 0.6 g/dL after worsening of PaO_2_/FiO_2_, DO2, and VO2 (p > 0.05 between scenarios). However, the mean Hb threshold increased by ≥ 1 g/dL after ScvO_2_ dropped from 70 to 50% (p < 0.001 between scenarios).

**Conclusion:** Improvement of ScvO_2_ or blood lactate did not prompt pediatric intensivists to decrease significantly their Hb threshold (< 0.2 g/dL), unless the ScvO_2_ increased from 50 to 70% after a cardiac surgery. A clinically significant drop in ScvO_2_ (from 70 to 50%) caused a clinically significant increase in the revised Hb threshold in all scenarios (≥ 1.0 g/dL), but worsening PaO_2_/FiO_2_, DO2 and VO2 were associated with a lower Hb threshold (≤ − 0.6 g/dL).

**Compliance with ethics regulations:** Yes in clinical research.
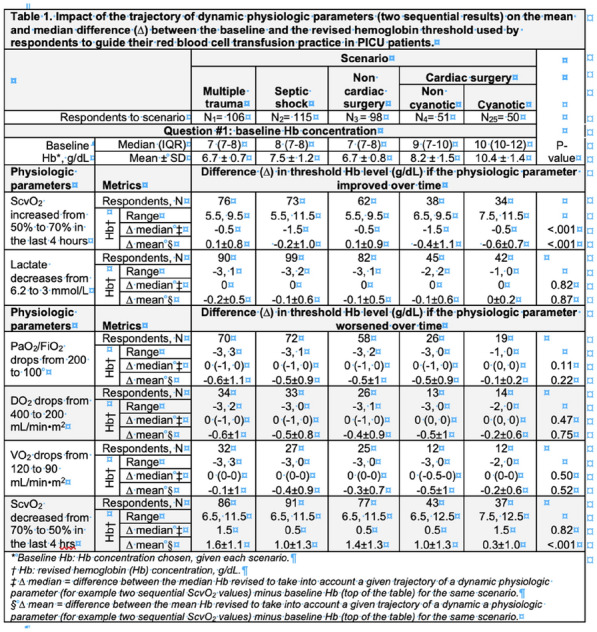


*Impact of the trajectory of dynamic physiologic parameters (2 sequential results) on mean and median difference between baseline and revised hemoglobin threshold used by respondents to guide their red blood cell transfusion practice in PICU patients.*

### FC-213 Thrombocytopenia and COVID-19: what prognostic value?

#### BOUCHECH Bouthaina^1^, BEN AMOR Oumaima^2^, CHTOUROU Ameni^2^, KOLSI Hichem^2^, KTATA Salma^1^, KESKES Mariem^1^, LOUATI Bilel^2^, KAROUI Abdelhamid^1,2^

##### ^1^hopital hbib bourguiba, Sfax, Tunisie; ^2^hopital de campagne Slim Chaker, Sfax, Tunisie

###### Correspondence: Bouthaina BOUCHECH (bouthainasellami@gmail.com)

*Annals of Intensive Care* 2022, **12(1):**FC-213

**Rationale:** Since December 2019, a severe acute respiratory syndrome coronavirus 2 is spreading rapidly around the world and became a pandemic. Among the confirmed cases, 15% developed severe forms and the mortality in intensive care units ranged from 30 to 70%. Indicators are needed to evaluate and predict the severity of the disease. We aimed to determine if thrombocytopenia is independently associated wtih poor outcomes in critically patients with COVID-19.

**Patients and methods/Materials and methods:** This was a retrospective study extended over a period from February to July 2021 including 240 infected patients by SARS-CoV-2. The exclusion criteria for this study were patients with progressive hematological pathology, patients with chronic renal failure and patients under anticoagulation or antiplatelet therapy before their infection with SARS-CoV-2. Biological monitoring of platelet levels was carried out in all patients admitted to intensive care on Day 1, Day 5, Day 12 and beyond 12 days every 72 h. All these patients had a preventive or curative anticoagulation by Low Molecular Weight Heparin. Thrombocytopenia was defined by platelet count < 150 × 10^9^/L. For each patient, the nadir platelet count was identified and categorized into 3 groups: G1 [150–100]; G2 [100–50] and G3 [< 50] (× 10^9^/L).

**Results:** A total of 240 patients were included in this study, 60.7% of whom were male. The median age was 62.4 years.The diagnosis of the infection was made in 37% by a rapid test, 25.4% by RT-PCR and 82.6% by chest CT. 94% of patients received enoxaparin anticoagulation, 44.2% of whom had curative anticoagulation during their stay. We found that 26.7% had thrombocytopenia and for patients categorized into [150–100]; [100–50] and [< 50] groups, the thrombocytopenia was 5.1%, 12.3%, and 7.2% respectively. Patients developed thrombocytopenia between D1 and D27 with an average of 9.9 days (11%). Patients with thrombocytopenia died with significant difference (p = 0.3%). The G2 presented a statistically significant difference with (P = 0.07%) in terms of death as well as the early onset of thrombocytopenia (p = 0.065).

**Conclusion:** Although, it was a retrospective descriptive study and the tests on platelet count for each patient had different time intervals, this study confirms that thrombocytopenia upon admission is a strongly and independently associated with poor outcomes and mortality in patients hospitalized for SARS-CoV-2 pneumonia.

**Compliance with ethics regulations:** Yes in clinical research.

### FC-214 Respiratory variations of Central Venous Pressure during a standardized inspiration maneuver to predict fluid responsiveness in spontaneously breathing patients: a prospective, monocentric, diagnostic test evaluation

#### BOUREL Claire^1^, DURAND Arthur^1^, TERSCHIPHORST Benoit^1^, ONIMUS Thierry^1^, FAVORY Raphael^1^, PREAU Sebastien^1^

##### ^1^CHU de Lille, Lille, France

###### Correspondence: Claire BOUREL (clairebourel@gmail.com)

*Annals of Intensive Care* 2022, **12(1):**FC-214

**Rationale:** Delayed or excessive volume expansion increases morbidity/mortality in acute circulatory failure patients. Therefore, prediction and assessment of fluid responsiveness (FR) is necessary. Hemodynamic parameters used to guide fluid therapy are regularly limited by spontaneous breathing (SB) or technicity. Central Venous Pressure (CVP) as a static parameter fails to accurately predict FR. The ideal marker to predict FR has to be accessible, fast, usable in spontaneous breathing and accurate. CVP respiratory variations (CVPv) could meet these criteria. We analyzed the accuracy of CVPv to predict FR in spontaneously breathing patients, and its optimization by a standardized inspiratory maneuver (SIM).

**Patients and methods/Materials and methods:** We carried out a monocentric, prospective, diagnostic test evaluation. Patients admitted to our intensive care units that were spontaneously breathing and had a central venous catheter were prospectively included. CVPv was measured while the patient was spontaneously breathing, with (CVPv-sim) and without SIM (CVPv-nsim), before and after a passive leg raising test (PLR). The SIM was validated by a buccal cavity pressure < − 3 mmH_2_O. CVPv accuracy to predict FR was evaluated. FR was defined as a PLR-induced increase in the velocity time integral of aortic blood flow during a passive leg raising maneuver (self-fluid challenge) ≥ 10%.

**Results:** Among 63 patients, 38 (60%) presented FR. CVPv-nsim was not significantly different between fluid responsive and fluid unresponsive patients (− 4.9 mmHg [− 7.5; − 3.1] vs. − 4.1 mmHg [− 5.4; − 2.8], respectively; p = 0.15). CVPv-sim was higher in fluid responsive than fluid unresponsive patients (− 3.6 mmHg [− 10.6; − 1.6] vs − 9.7 mmHg [− 13.9; − 6.2], respectively; p < 0.05). CVPv-sim < − 4,5 mmHg predicted FR with a sensitivity of 89%, a specificity of 56%, and an area under ROC curve of 0.72 [0.56;0.86]. There were 4 false negative and 11 false positive patients. The overall median CVPv-sim increased from − 8.6 mmHg [-12.5;-3.6] to − 4.1 mmHg [− 9.75; − 0.25] during PLR (p < 0.05). Among the 11 false positive results before PLR, 4 patients could not be analyzed during PLR, 4 patients moved from false positive to true negative and 3 increased their CVPv-sim but remained false positive during PLR.

**Conclusion:** In presence of a central venous catheter, CVPv may be useful to identify spontaneously breathing patients unresponsive to volume expansion.

**Compliance with ethics regulations:** Yes in clinical research.

### FC-215 Prediction of fluid responsiveness by the corrected carotid flow time changes during a passive leg rising maneuver

#### WATTECAMPS Guilhem^1^, MORICONI Mickaël^1^, TONNELIER Alexandre^1^

##### ^1^CHIC Quimper, Quimper, France

###### Correspondence: Guilhem WATTECAMPS (guilhem.wattecamps.29@gmail.com)

*Annals of Intensive Care* 2022, **12(1):**FC-215

**Rationale:** The physician must rely on trustworthy and reliable tools to predict fluid responsiveness in order to provide an efficient and safe fluid expansion. We aimed to evaluate the ability of the measurement of corrected carotid flow time changes during a passive leg rising maneuver to predict fluid responsiveness among intensive care unit patients.

**Patients and methods/Materials and methods:** We performed a prospective and non-interventional study among patients in whom the physician in charge has decided a fluid expansion prior to the inclusion in the study. We performed the carotid flow time measurements before and after a passive leg rising maneuver. We used a linear Doppler probe. Fluid responsiveness was defined as an at least 15% rise in cardiac output following a fluid expansion of 500 mL of isotonic saline solution. Cardiac output was assessed after the PLR maneuver, thanks to a transpulmonay thermodillution PICCO2^®^ device. A second blinded investigator read the images recorded by the first one. We evaluated the accuracy of the cCFT changes through a ROC analysis. We evaluated the inter observers' agreement with a Bland Altman analysis.

**Results:** From January to July 2021, we included 21 patients, 13 responders and 8 non responders. The ROC analysis showed that a 4, 3% rise in cCFT predicted fluid responsiveness with a 76,9% sensitivity, a 87,5% specificity, a positive predictive value of 90,9% and a negative predictive value of 70%. The area under the curve was 0,85 CI 95% [0,68; 1]. In the post hoc blinded assessment, we showed a poor agreement between the two operators.

**Conclusion:** Correct Carotid Flow Time changes during a PLR maneuver may be a good tool to predict fluid responsiveness. However, the agreement between two observers is weak and could be an obstacle to its widespread use.

**Compliance with ethics regulations:** Yes in clinical research.
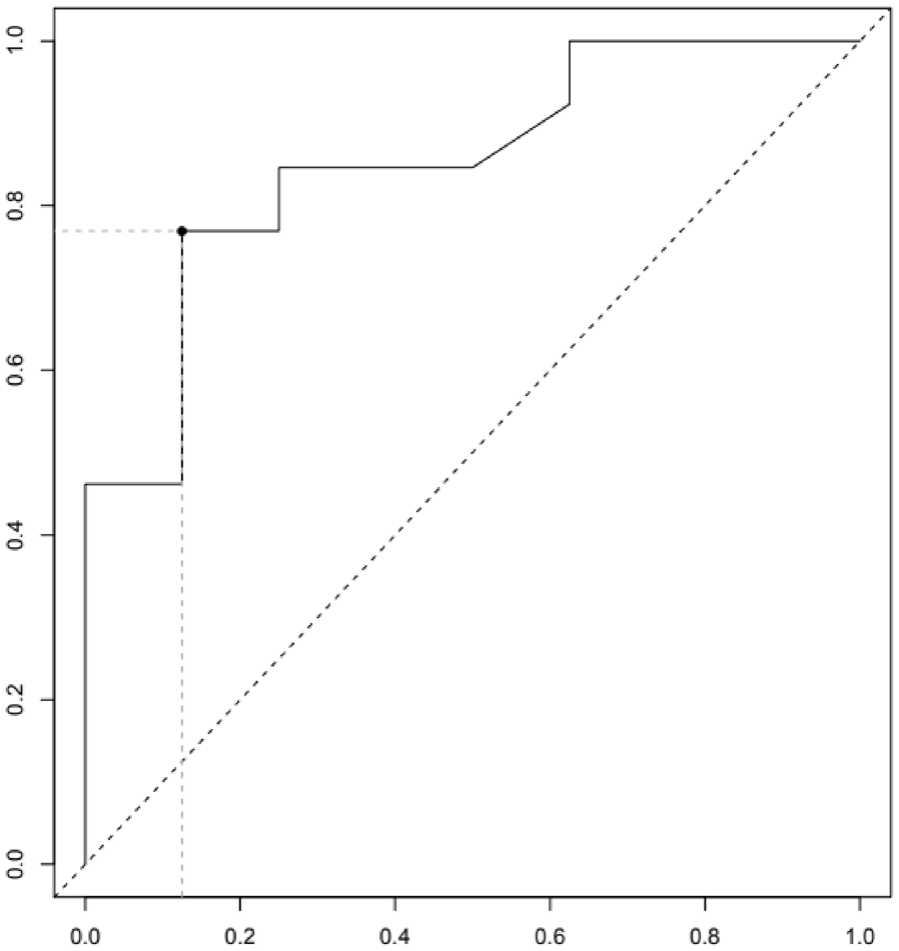


*ROC curve of the corrected carotid flow time changes during a passive leg rising maneuver*

### FC-216 Acute circulatory failure: comparative study between a population with or without echocardiography in the emergency department of Oran university hospital

#### ALACHAHER Djamel^1^, GOULMANE Mourad^1^, OUALI DADA Sofiane^1^, SIDI AISSA Nabil^1^, TABET AOUL Nabil^1^

##### ^1^Centre hospitalo-universitaire d'Oran/Faculté de Médecine d'Oran. Université Oran 1 Ahmed Ben Bella, Oran, Algerie

###### Correspondence: Djamel ALACHAHER (jalachaher@yahoo.fr)

*Annals of Intensive Care* 2022, **12(1):**FC-216

**Rationale:** Acute circulatory failures represent a frequent reason for hospitalization in the emergency department of the University hospital of Oran (CHUO), they are considered as serious states because in the absence of an adapted treatment, they can be rapidly life-threatening. The main objective of our study was to assess the contribution of echocardiography in our patients admitted in shock from a diagnostic, therapeutic and prognostic perspective.

**Patients and methods/Materials and methods:** A clinical and epidemiological, descriptive, prospective and mono-centric study was carried out  in the medical emergency department of the CHU Oran concerning any patient aged over 15 years admitted for a non traumatic acute circulatory failure. The group of patients benefiting from trans- thoracic echocardiography (TTE) evaluated during a period of 2 years from 01-01-2016 to 31-12-2017 and designated the “echo yes” group was compared with a second group of patients who did not benefit from the TTE collected from 01-01-2015 to 31-12-2015 and named group “echo no”.

**Results:** Seventy-seven (77) patients from the general population (n = 156) presenting a acute circulatory failure collected in our study benefited from TTE, i.e. a frequency of 49.35% with an average age of 64, 40 ± 17.70 years [17–106] and a sex ratio of 1.13, comorbidities were dominated by hypertension (59.70%) and diabetes (39%) and the reasons for consultation were represented by deterioration in general condition (74.30%) and disturbance of consciousness (50.64%). The ETT by two-dimensional exploration and Doppler allowed to determine the etiological mechanism in 94.8% of cases [38.96% (n = 30) hypovolemic shock, 36.36% (n = 28) cardiogenic shock, 88% (n = 13) septic shock and 2, 59% (n = 2) anaphylactic shock]. The rate of cases of undetermined mechanism decreased very significantly (“echo yes”: 5.20% vs. “echo no”: 36.70%), decrease by more than 31%, p < 0.001. The correction of the initial diagnostic hypothesis was noted in 29/77 patients, ie a frequency of 37.66%.

**Conclusion:** Our work has clearly illustrated and shown the contribution of TTE in improving the overall care of patients with shock by modifying the initial diagnostic hypothesis with, as corollary to the therapeutic management, to specify the etiological mechanism and to improve the prognosis of the patients.

**Compliance with ethics regulations:** Yes in clinical research.

### FC-217 Assessment of right ventricular mechanics by 3D transoesophageal echocardiography in the early phase of ARDS

#### EVRARD Bruno^1^, LAKATOS Bálint Károly ^2^, GOUDELIN Marine^1^, VIGNON Philippe^1^, KOVÁCS Attila^2^

##### ^1^CHU Limoges, Limoges, France; ^2^Heart and Cardiovascular Center, Semmelweis University, Budapest, Hongrie

###### Correspondence: Bruno EVRARD (bruno.evrard@chu-limoges.fr)

*Annals of Intensive Care* 2022, **12(1):**FC-217

**Rationale:** There are growing evidence distinguishing SARS-CoV-2-induced Acute Respiratory Distress Syndrome (ARDS) and those of other causes. This raises the possibility that severe COVID-19 may result in specific alterations of Right Ventricular (RV) mechanics. The objective was to compare global and axial right ventricular ejection fraction in ventilated patients for moderate-to-severe ARDS secondary to early SARS-CoV-2 pneumonia or to other causes, and in ventilated patients without ARDS used as reference.

**Patients and methods/Materials and methods:** Retrospective single-center cross-sectional study including 64 ventilated patients: 21 with ARDS related to SARS-CoV-2 (group 1), 22 with ARDS unrelated to SARS-CoV-2 (group 2), and 21 without ARDS (control group). Real-time three-dimensional transesophageal echocardiography was performed for hemodynamic assessment within 24 h after admission. Contraction pattern of the right ventricle was decomposed along the three anatomically relevant axes. Relative contribution of each spatial axis was evaluated by calculating ejection fraction along each axis divided by the global right ventricular ejection fraction.

**Results:** Global right ventricular ejection fraction was significantly lower in group 2 than in both group 1 and controls (median: 43% [25th–75th percentiles: 40–57] vs. 58% [55–62] and 65% [56–68], respectively: p < 0.001). Longitudinal shortening had a similar relative contribution to global right ventricular ejection fraction in all groups (group 1: 32% [28–39], group 2: 29% [24–40], control group: 31% [28–38], p = 0.6). Radial shortening was lower in group 2 when compared to both group 1 and controls (45% [40–53] vs. 57% [51–62] and 56% [50–60] respectively: p = 0.005). The relative contribution of right ventricular shortening along the anteroposterior axis was not statistically different between groups (group 1: 51% [41–55], group 2: 56% [46–63], control group; 56% [50–64], p = 0.076).

**Conclusion:** During early hemodynamic assessment, the right ventricular systolic function appears more impaired in ARDS unrelated to SARS-CoV-2 when compared to early stage SARS-CoV-2 ARDS. Radial shortening appears more involved than longitudinal and anteroposterior shortening in patients with ARDS unrelated to SARS-CoV-2 and decreased right ventricular ejection fraction.

**Compliance with ethics regulations:** Yes in clinical research.

### FC-218 Visualization of the superior vena cava using trans-thoracic echocardiography: learning curves using the cusum method

#### SOLTANI Joséphine^1^, PETIT Matthieu^1,2^, GODEMENT Mathieu^1^, JULLIEN Edouard^1,2^, VIEILLARD-BARON Antoine^1,2^, CHARRON Cyril^1^, GERI Guillaume^1^

##### ^1^Hôpital Ambroise Paré, Boulogne Billancourt, France; ^2^Université Paris Saclay, Saclay, France

###### Correspondence: Guillaume GERI (dr.guillaume.geri@gmail.com)

*Annals of Intensive Care* 2022, **12(1):**FC-218

**Rationale:** Echocardiography is routinely used in the hemodynamic monitoring of ICU patients. Trans-esophageal echo (TEE) has been described to provide additional evaluation, especially on the superior vena cava (SVC). While trans-thoracic echo (TTE) remains a marginal tool in SVC evaluation, learning curve of such a skill is unknown.

**Patients and methods/Materials and methods:** Monocentric observational study performed in a tertiary medical ICU used to use TEE in hemodynamic monitoring. A theoretical training was provided to all the medical staff (i.e. the trainees) and a structured data collection was performed including history of echo training of each trainee as well as echo data (including 2-dimensional [2D] and time-movements [TM] screenshots). These screenshots were reviewed by an external blinded expert using a Likert-derived scale (0: incorrect, 1: not optimal and 2: correct) for 2D and TM views. The cumulative sum (CUSUM) method was used to evaluate the evolution of trainees’ skills over time; an unacceptable failure rate (h1) and acceptable failure rate (h0) were a priori determined as 50 and 20%, respectively. Trainees who crossed h0 were considered successful while those crossing h1 had a unacceptable failure rate. Comparison of characteristics of trainees who succeeded and who failed were compared.

**Results:** 13 trainees were observed for a 2-months period. The median success rate was 77.8 [65.2, 80] and 33.3 [23.8, 42.9] for obtaining a correct 2D and a TM view, respectively. Three trainees crossed h0 for getting a correct 2D view while none did for a correct TM view. Crossing h0 occurred after 5, 12 and 17 procedures. Furthermore, all the trainees crossed h1 for the TM view, meaning the whole group crossed an unacceptable failure rate for this endpoint (Figure). The comparison of trainees who succeeded and who failed did not reveal any difference.

**Conclusion:** A one-shot theoretical training to get a correct visualization of the superior vena cava using TTE was not enough as most of them did not reach an acceptable failure rate.

**Compliance with ethics regulations:** Yes in clinical research.
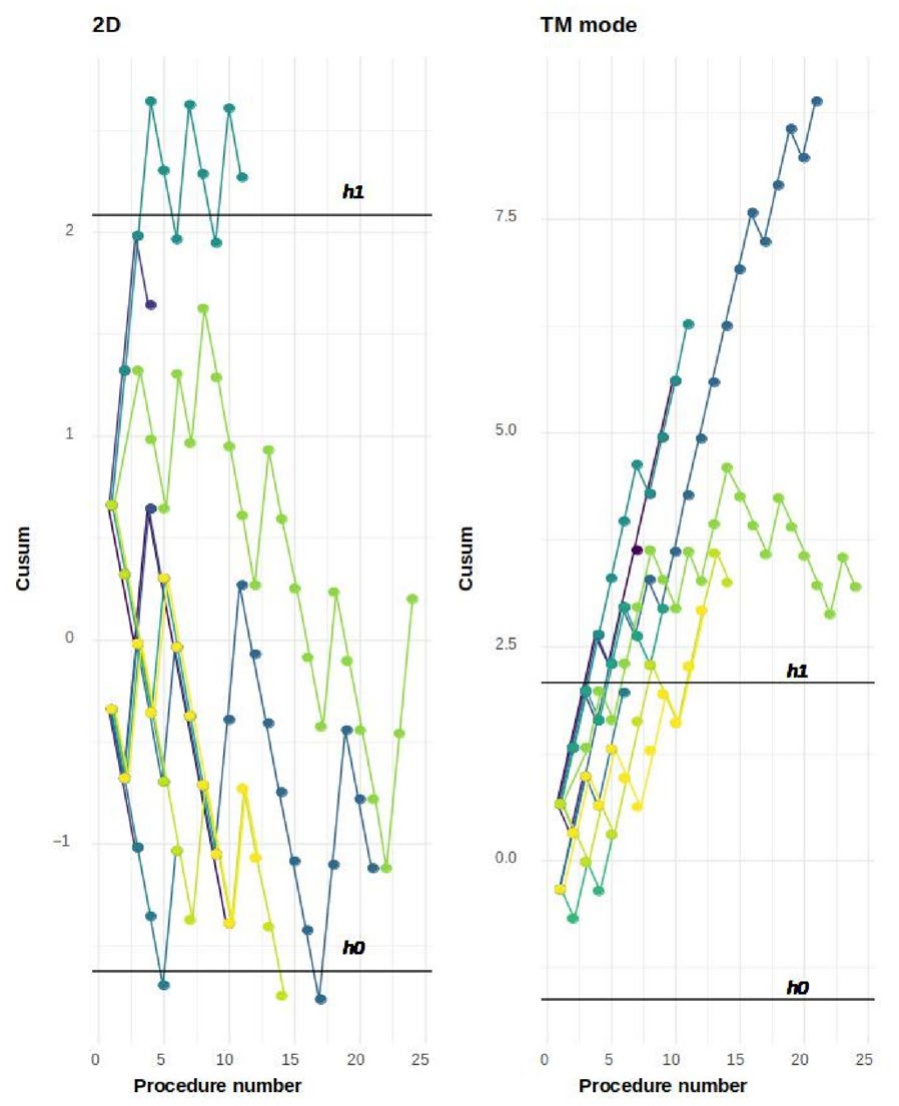


*CUSUM charts for 2D and TM views*

### FC-219 Cardiac manifestations in adult hemophagocytic lymphohistiocytosis: clinical and echocardiographic study

#### CERVEAUX Yohan^1^, SLAMA Michel^2^, MAIZEL Julien^2^, BRAULT Clément^2^

##### ^1^Centre hospitalier ouest Réunion, Saint Paul, Reunion; ^2^Centre hospitalo-universitaire Amiens Picardie, Amiens, France

###### Correspondence: Yohan CERVEAUX (yohan.cerveaux@gmail.com)

*Annals of Intensive Care* 2022, **12(1):**FC-219

**Rationale:** Only a few cases of cardiac dysfunction in hemophagocytic lymphohistiocytosis (HLH) have been reported. However, the prognosis for these patients is poor. A complete echocardiographic evaluation may provide an important clue for a better understanding of the pathogenesis.

**Patients and methods/Materials and methods:** We conducted a retrospective study of all patients diagnosed with HLH and hospitalized in intensive care unit (ICU) between 2008 and 2020. All patients had to get a baseline echocardiography in the 2 years before ICU admission. We compared SALH cases versus patients with septic shock.

**Results:** We included 28 patients; all of them had haematological malignancies. Compared with baseline echocardiography, left ventricular ejection fraction (LVEF) was decreased during SALH (55% vs. 65%; p = 0.02), with no significant difference in cardiac output. Likewise, the right ventricle function, assessed by tricuspid annular plane systolic excursion (TAPSE), was significantly impaired (p < 0.01). Only 36% of SALH patients had a LVEF lower than 50% versus 63% of septic shock patients (p = 0.03).

**Conclusion:** Cardiac involvement in HLH patients is characterized by a left and right ventricular dysfunction, however, it is less frequent and severe than in septic cardiomyopathy. Cardiac involvement in HLH and in septic shock could share similar pathophysiological mechanisms or it might be the same clinical affection. A prospective study comparing septic myocarditis and HLH heart disease is needed to conclude.

**Compliance with ethics regulations:** Yes in clinical research.

### FC-220 How central venous oxygen saturation can be used as a prognostic tool in cardiac surgery?

#### KOLSI Hichem^1^, JAWADI Wael^1^, KESKES Mariem^1^, SELLAMI Bouthaina^1^, KETATA Salma^1^, AMOURI Salim^1^, DERBEL Rahma^1^, CHEIKHROUHOU Hichem^1^, TRIKI Zied^1^

##### ^1^Centre hospitalo-universitaire Habib Bourguiba, Sfax, Sfax, Tunisie.

###### Correspondence: Hichem KOLSI (hichem.kolsi17@gmail.com)

*Annals of Intensive Care* 2022, **12(1):**FC-220

**Rationale:** Cardiac surgery with cardiopulmonary bypass (CPB) is known to be a lifesaving procedure. Nevertheless, CPB induces systemic inflammatory response and oxygenation impairment. Central venous oxygen saturation (ScvO_2_) is a classic marker of dysoxia. Low and high ScvO_2_ are associated with adverse outcome. However, there are no studies defining clearly abnormal values of ScvO_2_ and assessing the prognostic interest of ScvO_2_ in cardiac surgery. The aim of this study was to investigate the impact of increase and decrease of values of ScvO_2_ on mortality and morbidity after cardiac surgery, and to define optimal thresholds of high and low ScvO_2_ to predict complications.

**Patients and Methods/Materials and methods:** This was a prospective, observational, monocentric clinical study. We included 156 patients over 18 years undergoing cardiac surgery with CPB from September 2018 to January 2020. We have excluded patients who have died during surgery, patients who leave the operating room with a circulatory assistance technique and patients with misplacement of central venous catheter (CVC). Venous blood gas was collected from middle line of CVC. Population was divided into 2 groups: group H with ScvO_2_ > 75% and group L with ScvO_2_ ≤ 75%. In group H, we analyzed receiving operating curve (ROC) of ScvO_2_ for occurring of postoperative complications. In group L, we analyzed ROC of—ScvO_2_ for occurring of postoperative complications. We tested then the correlation of the variables with prognosis interest to ScvO_2_ by Spearman test.

**Results:** Median age was 62 (53; 68). Complications rate was 37.8%. Mortality rate was 18.6%. In group H, we have found that ScvO_2_ predicts complications with area under ROC 0.776. The best threshold was 80.6%. Moreover, we found that ScvO_2_ is significantly correlated with CPB time, mechanical ventilation time and majority of postoperative prognosis scores. In group L, the area under ROC of—ScvO_2_ concerning complications was 0.774. The best threshold was 69.7%. We found a significant negative correlation of ScvO_2_ to CPB time, mechanical ventilation time and majority of postoperative prognosis scores.

**Conclusion:** We conclude that both low and high ScvO_2_ are associated with adverse outcome after cardiac surgery. For patients with supranormal or low ScvO_2_, we should use additional hemodynamic monitoring to facilitate adequate therapy in the postoperative course. Nevertheless, to place as a target for postoperative resuscitation algorithm, a randomized trial comparing a conventional resuscitation strategy with a strategy based on defined levels of ScvO_2_ is essential.

**Compliance with ethics regulations:** Yes in clinical research.

### FC-221 Inappropriate intensive care unit stays: Perception and experience of ICU caregivers

#### MATHEY Lucas^1^, JACQUIER Marine^1,2^, MEUNIER-BEILLARD Nicolas^3,4^, ECARNOT Fiona^5^, ANDREU Pascal^1^, ROUDAUT Jean-Baptiste^1^, LABRUYERE Marie^1,3^, RIGAUD Jean-Philippe^6,7^, QUENOT Jean-Pierre^1,2,3,8^

##### ^1^CHU Dijon, Dijon, France; ^2^Equipe Lipness, centre de recherche INSERM UMR1231 et LabEx LipSTIC, université de Bourgogne-Franche Comté, Dijon, France; ^3^INSERM, CIC 1432, Module Épidémiologie Clinique, université de Bourgogne-Franche Comté, Dijon, France; ^4^DRCI, USMR, CHU Dijon Bourgogne, Dijon, France; ^5^EA3920, Department of Cardiology, University Hospital Besancon, Besancon, France; ^6^Centre Hospitalier de Dieppe - Department of Intensive Care, Dieppe, France; ^7^Espace de Réflexion Éthique de Normandie, University Hospital Caen, Caen, France; ^8^Espace de Réflexion Éthique Bourgogne Franche-Comté (EREBFC), Dijon, France

###### Correspondence: Jean-Pierre QUENOT (jean-pierre.quenot@chu-dijon.fr)

*Annals of Intensive Care* 2022, **12(1):**FC-221

**Rationale:** The question of futile or disproportionate care in the intensive care unit (ICU) has long been a subject of debate. It can happen that ICU stays come to be deemed inappropriate, after the fact, even by the ICU physicians themselves^1^. This study aimed to describe and understand the perceptions of ICU caregivers about ICU stays that the ICU physicians themselves judge to be inappropriate.

**Patients and methods/Materials and methods:** We performed a qualitative study using semi-structured interviews with caregivers from 3 ICUs in the region of Burgundy-Franche-Comté. An interview guide was developed in advance and focused on 3 questions, namely: (1) what is your experience and perception of ICU stays that are judged after the fact to have been inappropriate? (2) What are the consequences of an inappropriate ICU stay, in your experience? (3) In your view, how could such stays be avoided? Interviews were recorded, transcribed in full and analyzed (in French) using thematic analysis. Participants were interviewed until saturation was reached.

**Results:** A total of 17 nurses and 4 nurses’ aides were interviewed. Age, medical history, poor general status, deteriorating quality of life, and the lack of clear healthcare goals were the main criteria cited by respondents as possible reasons why an ICU stay would be deemed inappropriate after the fact by ICU physicians. The respondents also reported that certain specific circumstances may also give rise to ICU stays that could be inappropriate, e.g. emergency clinical situations, lack of knowledge about case, availability and/or choice of physicians, pressure from the patient’s family or from other physicians to admit the patient, and refusal of death. Possible consequences of inappropriate ICU stays cited by our respondents included stress, anxiety, a feeling of incomprehension and inequality, deprivation of liberty, and loss of the feeling that their work is meaningful. Conversely, possible avenues to preventing inappropriate ICU stays cited by the respondents, included anticipation of acute care needs, and careful evaluation of the patient prior to admission.

**Conclusion:** In daily practice, paramedical caregivers understand how some ICU stays may come to be considered after the fact as having been inappropriate. They could undoubtedly provide valuable input into admission decisions, to avoid inappropriate ICU stays and to ensure equitable distribution of healthcare resources and the respect of patients’ wishes.

**Reference 1:** Quenot JP, Large A, Meunier-Beillard N, et al. PlosOne 2019 Sep 6;14(9):e0222039.

**Compliance with ethics regulations:** Yes in clinical research.

### FC-222 Non-beneficial admission to the Intensive Care Unit: A Nationwide Survey of Practices

#### QUENOT Jean-Pierre^1,2,3,4^, JACQUIER Marine^1,3^, FOURNEL Isabelle^2^, MEUNIER-BEILLARD Nicolas^2,5^, GRANGÉ Clotilde^1^, ECARNOT Fiona^6^, LABRUYERE Marie^1^, RIGAUD Jean-Philippe^7,8^

##### ^1^CHU Dijon, Service de Médecine Intensive & Réanimation, Dijon, France; ^2^INSERM, CIC 1432, Module Epidémiologie Clinique, Dijon, France; CHU Dijon-Bourgogne, Centre d’Investigation Clinique, Module Epidémiologie Clinique/Essais Cliniques, Dijon, France; ^3^Equipe Lipness, centre de recherche INSERM UMR1231 et LabEx LipSTIC, université de Bourgogne-Franche Comté, Dijon, France; ^4^Espace de Réflexion Éthique Bourgogne Franche-Comté (EREBFC), Dijon, France; ^5^DRCI, USMR, CHU Dijon Bourgogne, Dijon, France; ^6^EA3920, Department of Cardiology, University Hospital Besancon, Besancon, France; ^7^Centre Hospitalier de Dieppe, Department of Intensive Care, Dieppe, France; ^8^Espace de Réflexion Éthique de Normandie, University Hospital Caen, Caen, France

###### Correspondence: Jean-Pierre QUENOT (jean-pierre.quenot@chu-dijon.fr)

*Annals of Intensive Care* 2022, **12(1):**FC-222

**Rationale:** Although the question of futile or disproportionate care in the intensive care unit (ICU) has been widely discussed in the literature, there is a paucity of studies about non-beneficial admissions to the ICU^1^. Labelling ICU stays as non-beneficial may give a false perception of the utility of ICU management. Using a national survey of practices, we sought to define criteria for non-beneficial admissions to ICU, the circumstances in which they occur, and the possible consequences, with a view to identifying aspects that could be targeted for change.

**Patients and methods/Materials and methods:** An online survey was made available from 22 June to 4 October 2021 via the LimeSurvey platform for ICU physicians. For each question, the physician was asked to answer on a 5-point Likert scale (strongly disagree/disagree/neutral/agree/strongly agree). The survey used the Network for Research in Ethics in Critical Care to ensure representativeness of the participating ICUs and forms of practice of the respondents.

**Results:** In total, 154/164 ICU physicians contacted (94%) completed the online survey. The main results highlight that an ICU admission must take into account the expected survival and quality of life after discharge, in view of the current clinical status and prior medical history of the patient. The urgency of the clinical situation, a lack of knowledge of the medical file, and a failure to anticipate potential ICU requirement in the patient’s healthcare trajectory are all factors that may lead to non-beneficial ICU admission. Stress, anxiety, incomprehension and even conflict with the patient’s family or between healthcare providers are possible consequences of ICU admissions occurring in such circumstances. Finally, one of the key measures that could be implemented to reduce or avoid non-beneficial admissions is pluridisciplinary meetings prior to ICU, involving the patient, the family, referring and treating physicians, as well as an ICU physician when the possibility of ICU admission is part of the patient’s healthcare goals.

**Conclusion:** This survey of practice suggests that there is a compelling need for shared reflection and decision-making, likely with greater involvement of ICU physicians, prior to ICU admission. This would take closer account of the preoccupations of non-ICU physicians, and could help to identify in advance the patients likely to benefit from ICU care, in coherence with their healthcare goals.

**Reference 1:** Quenot JP, Large A, Meunier-Beillard N, PlosOne 2019 Sep 6;14(9):e0222039.

**Compliance with ethics regulations:** Yes in clinical research.

### FC-223 How is the COVID crisis a challenge for caregivers regarding the place of relatives in intensive care unit ?

#### DE VILLIERS DE LA NOUE Valentin^1,2^, VIALLARD Marcel-Louis^1^

##### ^1^Université de Paris, Paris, France; ^2^CHU Robert Debré, Reims, France

###### Correspondence: Valentin DE VILLIERS DE LA NOUE (valentindelanoue@gmail.com)

*Annals of Intensive Care* 2022, **12(1):**FC-223

**Rationale:** Intensive care unit is a technical environment in which patients’ lives are at stake. The COVID pandemic has led to a restriction of the place given to the entourage of patients hospitalized in the intensive care unit. How is the COVID crisis a challenge for caregivers in terms of the place of the family and friends in the ICU?

**Patients and methods/Materials and methods:** Qualitative study including 10 semi-directive interviews with caregivers working in the ICU.

**Results:** The COVID crisis disrupted the place of patients’ relatives in ICU. This inclusion had been established by various means before the crisis: 24-h visits, participation to the mortuary toilet, pets’ visits, logbook, family lounges, presence of volunteers. These means lead to a better inclusion of relatives in ICU, even if there were still difficulties in the relationship with the family and friends: management of the acute phase, difficulties in communicating with the family and friends, waiting for care, choice of the person of trust, non-opening 24 h a day. The COVID crisis added barriers: initial closure of all the services in which the caregivers interviewed worked, then partial reopening, fear of the virus, constraints related to equipment, exacerbated communication difficulties, lack of medical time. The caregivers tried to implement new means to include relatives in this context: videoconferencing, virtual logbook, more regular telephone interviews. These difficulties in making room for the family and friends have deteriorated the relationship with the relatives, and the caregivers’ experience regarding these changes is mostly negative.

**Conclusion:** COVID crisis was a challenge for caregivers to make room for the entourage of hospitalized patients. Despite the means implemented to maintain this link between caregivers and family members, the face-to-face relationship seems to be necessary for caregivers and family members to live serenely in the resuscitation context.

**Compliance with ethics regulations:** Yes in clinical research.

### FC-224 Withholding or withdrawing therapy in intensive care unit: an analysis of collaboration among intensivists and general practitioners

#### ETCHEVERRY Jean-Baptiste^1^, RAVRY Céline^1^, GODARD Pierre^1,2^, MAYET Thierry^1^, AUVET Adrien^1^

##### ^1^CH de Dax, Dax, France; ^2^CHU Bordeaux, Bordeaux, France

###### Correspondence: Adrien AUVET (auveta@ch-dax.fr)

*Annals of Intensive Care* 2022, **12(1):**FC-224

**Rationale:** French law and practice guidelines (1) impose collegial procedures and interdisciplinary collaboration in end-of-life decision making. This procedure involves the intensive care unit (ICU) team and an external consultant. General practitioner (GP) could be a relevant consultant, through his understanding of the patient history and his relation with the family. The purpose of this study was to explore GP and intensivist point of views regarding the GP involvement in the collegial decision of withholding or withdrawing therapy.

**Patients and methods/Materials and methods:** We conducted a retrospective observational study over the year 2020. An online survey was sent by e-mail to the GPs and the intensivists of the 4 ICUs of a French mainland regional administrative county (“department”).

**Results:** 132 GPs and 22 intensivists answered. Almost all of the GPs (118/132; 89%) had at least 1 patient in ICU over the year. (55/118; 47%) have been aware that end-of-life decision making concerned at least one of their patients. When such a process started, intensivists requested (26/55; 47%) of them to participate in the procedure. Intensivists requested an external consultant in 77% situations. Most of the time, they asked a specialist (91%) and rarely discussed with the GP (36%). 61% of GPs felt having a strong impact on the procedure, whereas 46% of intensivists considered GP impact this way. 71% of GPs felt involved by the ICU team in the collegial decision, whereas 37% of intensivists felt the same way about it. Those professionals acknowledge a similar GP influence regarding the patient management (38% GP, 40% intensivists). 85% of GPs would like to be more solicited, whereas 36% of intensivists gave a major place to GP interventions. 79% of GPs were requested by a family member to help them understand the situation.

**Conclusion:** Our study shows that intensivists involved the GPs partially in the collegial decision. This lack of participation seems to be in inadequacy with GPs wishes. A systematic involvement of the GP in the decision making of withholding or withdrawing treatment could be beneficial and could improve interaction with the family. Moreover, GP could be directly involved in the collegial decision making and not just as consultants.

**Reference 1:** 1- Société de réanimation de langue. Limitation et arrêt des traitements en réanimation adulte. Actualisation des recommandations de la Société de réanimation de langue française. Réanimation. déc 2010;19(8):679–98.

**Compliance with ethics regulations:** Yes in clinical research.

### FC-225 Anticipating need for intensive care unit admission in case of acute deterioration of chronic disease: a qualitative study among non-ICU physicians

#### TAHA Alicia^1^, JACQUIER Marine^1,2^, MEUNIER-BEILLARD Nicolas^3,8^, ECARNOT F^4^, ANDREU Pascal^1^, ROUDAUT Jean-Baptiste^1^, BRUYERE Marie^1,3^, RIGAUD Jean-Philippe^5,6^, QUENOT Jean-Pierre^1,2,3,7^

##### ^1^CHU Dijon, Dijon, France; ^2^Centre de recherche INSERM UMR1231 et LabEx LipSTIC, Université de Bourgogne-Franche Comté, Dijon, France; ^3^INSERM, CIC 1432, Module Épidémiologie Clinique, université de Bourgogne-Franche Comté, Dijon, France; ^4^EA3920, Department of Cardiology, University Hospital Besancon, Besancon, France; ^5^Department of Intensive Care, Centre Hospitalier de Dieppe, Dieppe, France; ^6^Espace de Réflexion Éthique de Normandie, University Hospital Caen, Caen, France; ^7^Espace de Réflexion Éthique Bourgogne Franche-Comté (EREBFC), Dijon, France; ^8^DRCI, USMR, CHU Dijon Bourgogne, Dijon, France

###### Correspondence: Jean-Pierre QUENOT (jean-pierre.quenot@chu-dijon.fr)

*Annals of Intensive Care* 2022, **12(1):**FC-225

**Rationale:** Almost 70% of patients who are admitted to the intensive care unit (ICU) suffer from at least one chronic disease (1). Chronic disease in ICU patients is an additional risk factor for death and is associated with significant post-ICU disability. This study aimed to explore reflections and perceptions of non-ICU physicians for the anticipation of need for ICU admission in case of acute decompensation in patients with chronic disease.

**Patients and methods/Materials and methods:** We performed a qualitative multicentre study using semi-structured interviews among non-ICU specialist physicians. The interview guide, developed in advance, focused on 3 questions: (1) What is your perception of ICU care? (2) How do you think advance directives can be integrated into the patient’s healthcare goals? and (3) How can the possibility of a need for ICU admission be integrated into the patient’s healthcare goals? Interviews were recorded, transcribed and analyzed by thematic analysis. Interviews were performed until theoretical saturation was reached.

**Results:** In total, 16 physicians (8 women, 8 men) were interviewed. The main themes related to intensive care being viewed as a distinct specialty, dispensing very technical care, and with major human and ethical challenges, especially regarding end-of-life issues. The participants also mentioned the difficulty in anticipating an acute decompensation, and the choices that might have to be made in such a situation, for patient that they have known and followed for a long time. The timing of discussions for potential decompensation of the patient, the medical culture and the presence of advance directives are issues that arise when attempting to anticipate the question of ICU admission in the patient’s healthcare goals or wishes. To facilitate this process, it was proposed that dedicated consultations bringing together the patient’s referring physicians and ICU physicians could help to raise awareness and knowledge about ICU, and promote a palliative culture.

**Conclusion:** This study highlights the difficulties encountered by treating physicians of anticipating potential acute decompensation with ICU requirement in patients with chronic disease. It also opens perspectives for actions that could promote a pluridisciplinary approach to anticipating acute decompensation and ICU requirements in patients with chronic disease.

**Reference 1:** Quenot JP, Helms J, Labro G, et al. Ann Intensive Care 2020;10:20.

**Compliance with ethics regulations:** Yes in clinical research.

### FC-226 Anticipating need for intensive care unit admission in a patient’s healthcare trajectory: a qualitative study among general practitioners

#### JACQUIER Marine^1,2^, FOUQUET Bérénice^3^, MEUNIER-BEILLARD Nicolas^4,5^, ECARNOT Fiona^6^, ANDREU Pascal^1^, ROUDAUT Jean-Baptiste^1^, LABRUYERE Marie^1,4^, RIGAUD Jean-Philippe^7,8^, QUENOT Jean-Pierre^1,2,4,9^

##### ^1^CHU Dijon - Service de Médecine Intensive & Réanimation, Dijon, France; ^2^Equipe Lipness, centre de recherche INSERM UMR1231 et LabEx LipSTIC, université de Bourgogne-Franche Comté, Dijon, France; ^3^Ordre des médecins Rhône-Alpes, Lyon, France; ^4^INSERM, CIC 1432, Module Épidémiologie Clinique, université de Bourgogne-Franche Comté, Dijon, France; ^5^DRCI, USMR, CHU Dijon Bourgogne, Dijon, France; ^6^EA3920, Department of Cardiology, University Hospital Besancon, Besançon, France; ^7^Centre Hospitalier de Dieppe, Department of Intensive Care, Dieppe, France; ^8^Espace de Réflexion Éthique de Normandie, University Hospital Caen, Caen, France; ^9^Espace de Réflexion Éthique Bourgogne Franche-Comté (EREBFC), Dijon, France

###### Correspondence: Jean-Pierre QUENOT (jean-pierre.quenot@chu-dijon.fr)

*Annals of Intensive Care* 2022, **12(1):**FC-226

**Rationale:** The questions raised by Intensive care unit (ICU) physicians with non-ICU physicians and particularly general practitioners (GPs) usually aim to anticipate possible acute decompensation and need for ICU admission in the patient. Intensivists also question their correspondents about the patient’s wishes, with a view to avoiding unreasonable therapeutic obstinacy, or an ICU admission that might later be judged to have been inappropriate. We sought to explore GPs’ perception and experience of ICU, and their manner of anticipating possible future ICU requirements for their patients.

**Patients and methods/Materials and methods:** We performed a qualitative study using semi-structured interviews with GPs practising in the Bourgogne and Pays-de-Loire regions of France. An interview guide was developed in advance, and focused on the following questions: (1) what is your perception of ICU? (2) what is your role in anticipating ICU requirements for your patients? (3) how do you feel about ICU stays that may later be judged to have been unjustified, or inappropriate. Interviews were recorded, transcribed and analysed by thematic analysis. Interviews were performed until theoretical saturation was reached.

**Results:** A total of 20 GPs were interviewed between February and August 2021. The main themes to emerge from the analysis were: (1) The GPs perceive ICU care as being that is very far removed from their ordinary daily practice. Their usually rare contacts with ICUs became more common during the COVID pandemic, strengthening relations between GPs and ICUs. (2) Improved knowledge of patients’ wishes by GPs is desirable. This would make it possible, together with specialists during pluridisciplinary meetings, to better guide the patient’s healthcare trajectory. (3) GPs are generally not familiar with the notion of unjustified or inappropriate ICU stays, and do not give it much thought. (4) Advance directives and end-of-life issues should be detailed in the patient’s electronic medical record. However, due to a lack of time and training, GPs do not feel comfortable addressing these questions with their patients.

**Conclusion:** Anticipating ICU requirements in a patient’s healthcare trajectory is difficult for GPs due to a lack of time, and a lack of training or education about ethical issues, which are not a major preoccupation in their daily practice. There is undoubtedly a compelling need to improve communication between hospital-based specialists, and physicians working in primary care, and to incite the population to think about preparing advance directives.

**Compliance with ethics regulations:** Yes in clinical research.

### FC-227 Catastrophic COVID-19 delta variant surge in French West Indies—report of an ICU triage policy

#### POMMIER Jean-David^1^, DELAMARE Floran^1^, MARTINO Frederic^1^, VALETTE Marc^1^, DEMOULE Alexandre^2^, CAMOUS Laurent^1^

##### ^1^CHU de guadeloupe, Les Abymes, Guadeloupe; ^2^CHU Pitié-Salpétrière, Paris, France

###### Correspondence: Laurent CAMOUS (laurent.camous@chu-guadeloupe.fr)

*Annals of Intensive Care* 2022, **12(1):**FC-227

**Rationale:** Evaluate the results of crisis standards of care based on a multi-approach protocol in” real life conditions and the final outcome of all COVID-19 patients that have been proposed for admission to the intensive care unit (ICU) during a catastrophic delta variant SARS COV 2 surge overloading Intensive Care Unit (ICU) initial capacity.

**Patients and methods/Materials and methods:** All patients hospitalized in the University Hospital of Guadeloupe for COVID-19 pneumonia who were proposed for ICU admission during the surge peak from August 11th to September 10th were included. Retrospective analysis of population characteristics, outcome and death risk factors among patients admitted in ICU was performed.

**Results:** For this crisis, an ICU prioritization algorithm based on a multi-approach protocol was employed. According to day-by-day admission policy, three groups of patients were defined: “ICU ASAP”, “ICU maybe” and”No ICU”. During the 1-month study period, 328 patients were considered for ICU admission, of whom 132 (40%) were finally admitted. No “ICU ASAP “patients had to be refused of ICU admission due to Full ICU bed occupancy and 16 of the “ICU maybe” group patients had a secondary withdrawal of care”. In the ICU admitted patients (n = 152), 80 patients were from the “ICU ASAP” group and 52 from the “ICU maybe” group. Hospital mortality was 24% in the “ICU ASAP” patients and 37% in the “ICU maybe” patients. Unsurpringly, the “No ICU” group had a 77% mortality, reflecting severity of the patients. In ICU admitted patients, multivariate analysis found that late admission (> 48 H after first ICU referral) and hypertension were independent risk factor of death.

**Conclusion:** Multi approach protocol overcome a catastrophic COVID 19 Surge in Guadeloupe Island. Late admission (> 48 H after first referral) was grieved by raised ICU mortality in ICU eligible patients.

**Compliance with ethics regulations:** Yes in clinical research.

### FC-228 Sepsis in the emergency departement: is procalcitonin predictive of septic shock?

#### BAHRI Badra^1^, KHIARI Saoussen^1^, SEDGHIANI Ines^1^, GHABARA Racha^1^, DOGHRI Hamdi^1^, TOUJ Hajer^1^, CHOUAIEB Sonia^1^, BORSALI Nebiha^1^

##### ^1^hopital habib thameur tunis, tunisie, Tunis, Tunisie

###### Correspondence: Saoussen KHIARI (saoussen.khiari@gmail.com)

*Annals of Intensive Care* 2022, **12(1):**FC-228

**Rationale:** Sepsis is frequent in the emergency department and a common cause of intensive care unit admission. Early recognition of patients at risk of septic shock is essential. The purpose of our study was to evaluate the prognostic value of procalcitonin (PCT) in patients admitted to the emergency department for sepsis.

**Patients and methods/Materials and methods:** Prospective monocentric observational study, conducted between July and December 2021. We included patients aged 18 and over, admitted to the emergency department for sepsis with a SOFA score greater than or equal to 2 and a suspected infection. The procalcitonin assay was performed on admission. The primary endpoint was the onset of septic shock. The secondary endpoints were deaths in the emergency department, admission in intensive care and the use of mechanical ventilation.

**Results:** We included 137 patients aged on average 63.4 ± 14 years with a sex ratio of 1.49. The sites of infection sepsis were n (%): pulmonary 79 (58) including 60 (43) covid, urinary 38 (27), cutaneous 7 (5), digestive 8 (6), central nervous system 2 (1), and infective endocarditis 3 (2). The average PCT value was 9.16 ± 24 ng/ml and ranged from 0.03 to 100 ng/ml. The occurrence rate of a septic shock was 17.6% (n = 24) requiring the use of norepinephrine. Overall mortality was 35% (n = 48). The use of mechanical ventilation was indicated in 16.8% of cases. The intensive care admission rate was 20% (n = 27). The average emergency room stay was 5 ± 0.5 days. The mean PCT in the shock group was 3.1 ng/ml versus 0.64 ng/ml in the non-shock group (p = 0.006); the threshold value with the best sensitivity (79%) and specificity (43%) was 0.5 (Odds ratio = 3.6; AUC = 0.67). The mean PCT was 2.38 ng/ml in the deceased versus 0.43 ng/ml in the survivors (p = 0.004). The PCT threshold value for predicting mortality with the best sensitivity (68%) and specificity (91%) was 0.7 ng/ml (odds ratio = 2.1; AUC = 0.64). We did not findd significant difference in the use of mechanical ventilation (p = 0.06).

**Conclusion:** Procalcitonin is a predictive factor for the onset of septic shock and mortality in patients admitted to the emergency department for sepsis.

**Compliance with ethics regulations:** Yes in clinical research.

### FC-229 Histoplasmosis in the ICU: characteristics and outcome a retrospective study (2014–2021) in the French west indies

#### CAMOUS Laurent^1^, POMMIER Jean-David^1^, SUREL Arthur^1^, MARTINO Frederic^1^, NICOLAS Muriel^1^, CARLES Michel^2^

##### ^1^CHU de guadeloupe, Les Abymes, Guadeloupe; ^2^CHU de Nice, Nice, France

###### Correspondence: Laurent CAMOUS (laurent.camous@chu-guadeloupe.fr)

*Annals of Intensive Care* 2022, **12(1):**FC-229

**Rationale:** We aim to retrospectively identify the prevalence of severe histoplasmosis requiring intensive care management in a putative histoplasmosis endemic area without precise epidemiological data.

**Patients and methods/Materials and methods:** We conducted a retrospective review study of all adult patients (> 18 years old) who developed culture-confirmed histoplasmosis and admitted for a life-threatening event in the ICU of Guadeloupe University Hospital (GUH) from January 2014 toJune 2021.

**Results:** 21 patients were included with a median age of 61 (50.0;67.0), mostly men (n = 12, 57%). HIV was the most frequent underlying disease (n = 8, 38%). All HIV patients, whether treated or not, were severely immunocompromised with a CD4 cell count below 50 per mm3. Other underlying conditions were ongoing immunosuppressive treatments (n = 7, 33%), solid organ transplanted patients (N = 3, 14%) and cancer patients (n = 2, 10%)) (see Table 1). Three out of the 21 patients had no known immunosuppressive disorders but diabetes. The median IGS II at ICU admission was 63 (43.0;81.5). Median of symptoms duration before ICU admission was 23 days. The main clinical conditions requiring ICU admission were acute respiratory failure (n = 15, 71%), and/or shock then coma (n = 10 50%).

**Conclusion:** Histoplamosis is a rare but life-threatening infection with pleomorphic organ involvement in immunocompromised host. Physicians must be aware of diagnosis tools to propose fast antifungal therapy.

**Compliance with ethics regulations:** Yes in clinical research.

### FC-230 Severe community-acquired pneumonia due to *Acinetobacter baumannii* in Reunion Island: a retrospective observational study

#### ROTINI Giacomo^1^, DEMANGOU Axel ^1^, BARON Marie^1^, NATIVEL Mathilde^1^, PUECH Bérénice^1^, JABOT Julien^1^, ALLOU Nicolas^1^, VIDAL Charles^1^

##### ^1^CHU Félix Gyuon, Saint Denis, Reunion

###### Correspondence: Charles VIDAL (charlesvidal@orange.fr)

*Annals of Intensive Care* 2022, **12(1):**FC-230

**Rationale:** *Acinetobacter baumannii (Ab)* is usually involved in nosocomial infection especially in intensive care units (ICU). Few studies report series of severe community-acquired pneumonia (CAP) due to this germ, particularly in tropical or sub-tropical regions. The objective of this work is to perform an epidemiological analysis of CAP caused by *Acinetobacter baumannii (Ab)* in Reunion Island.

**Patients and methods/Materials and methods:** It was a retrospective study including all patients hospitalized for CAP in ICUs at the Reunion University Hospital in the period 2014–2020. CAP to *Ab* was confirmed by microbiological respiratory samples or blood cultures realized at the admission in ICUs. All patients presented nosocomial infection or pneumonia to *Ab* were excluded.

**Results:** In the inclusion period, 572 patients were hospitalized in the ICUs for severe CAP, 6 of which were attributable to *Acinetobacter baumannii* (CAP-*Ab*). Patients with CAP-*Ab* had more alcohol disorders (67% vs 28%, OR = 5, p = 0.05) and denutrition state (50% vs 16%, OR = 6.4, p = 0.02) than patient with “standard CAP”, 5/6 of patients with CAP-*Ab* were smokers. There was no statistically significant difference with diabetes, gender, immunosuppression and chronic respiratory diseases. Regarding the clinical presentation, patients with CAP-*Ab* presented septic shock (100%) and ARDS (67%). SOFA score at admission and mortality were higher (respectively 8 vs 3 and 83% vs 27% OR = 13, p < 0.01). All patients with CAP-Ab had initially received a first line inappropriate antibiotic therapy against *Ab* associating ceftriaxone/cefotaxime and spiramycine.

**Conclusion:** In Reunion Island, severe CAP due to *Acinetobacter baumannii* represent 1% of CAP hospitalized in ICU and are associated with high mortality. The severity of the initial clinical presentation as well as the presence of comorbidities such as alcohol disorders and denutrition should suggest this pathogen prompting a larger spectrum antibiotic therapy.

**Reference 1:** Leung, W.-S. et al. Fulminant Community-Acquired Acinetobacter baumannii Pneumonia as a Distinct Clinical Syndrome. Chest (2006).

**Reference 2:** Davis, J. S. et al. A 16-Year Prospective Study of Community-Onset Bacteremic Acinetobacter Pneumonia. Chest (2014).

**Compliance with ethics regulations:** Yes in clinical research.

### FC-231 Safety of early discontinuation of an empiric combination antibiotic regimen in severe community-acquired pneumonia: the STOP study

#### GUILLOT Pauline^1^, DELAMAIRE Flora^1^, PAINVIN Benoit^1^, PIAU Caroline^1^, REIZINE Florian^1^, LESOUHAITIER Mathieu^1^, GACOUIN Arnaud^1^, TADIE Jean-Marc^1^, MAAMAR Adel^1^

##### ^1^CHU de Rennes, Rennes, France

###### Correspondence: Pauline GUILLOT (pauline.guillot@chu-rennes.fr)

*Annals of Intensive Care* 2022, **12(1):**FC-231

**Rationale:** Severe community-acquired pneumonia (SCAP) is a leading cause of infectious mortality and a frequent reason for admission to intensive care unit (ICU). The recommended empirical antibiotic treatment is an association of a β-lactam plus a macrolide or a respiratory fluoroquinolone. Treatment de-escalation strategies are not uniform. Our objective was to assess the safety of discontinuing atypical micro-organism coverage in ICU.

**Patients and methods/Materials and methods:** We conducted a single-centre observational study over 6 years. Clinical data were collected retrospectively, microbiological data were collected prospectively. All consecutive patients over 18 years admitted to ICU with documented SCAP were included. All patients received an empirical combination therapy with a β-lactam plus a macrolide or quinolone. Legionella urine antigen test (UAT) was performed in all patients at admission. Macrolide or quinolone were discontinued if the UAT was negative. We examined the clinical and epidemiological characteristics of SCAP and analyzed the independent factors associated with ICU mortality.

**Results:** On 856 SCAP, 26 patients had atypical pneumonia: 18 Legionella pneumophila (LP) serogroup 1, 3 Mycoplasma pneumoniae (MP), and 5 Chlamydia psittaci (CP). There was no other Legionella serogroup or specie, and no Chlamydophila pneumoniae. UAT diagnosed 16 Legionella pneumonia. Combination therapy was discontinued for 5 patients with an atypical pneumonia: 1 with LP, 1 with MP and 2 with CP. Although the duration of mechanical ventilation was higher in atypical SCAP in univariate analysis, there was no significant difference in length of stay and ICU mortality compared with typical SCAP. The multivariate analysis showed SAPS II score (Odd-Ratio (OR) = 1.04 by 1-point increment; 95% confidence interval (CI) 1.03–1.05, p < 0.001) and the need for mechanical ventilation (OR = 5.4; 95% CI 2.5–13.3, p < 0.001) as independent factors associated with a higher ICU mortality. Type of micro-organism was not associated with a higher ICU mortality.

**Conclusion:** In the absence of a specific history, it might be safe to discontinue macrolides or quinolones when Legionella UAT is negative. This “early” discontinuation strategy reduces antibiotic consumption, which has an economic and ecological impact on the human microbiota and antibiotic resistance. These results need to be confirmed in a multicentre study with a larger cohort.

**Compliance with ethics regulations:** Yes in clinical research.
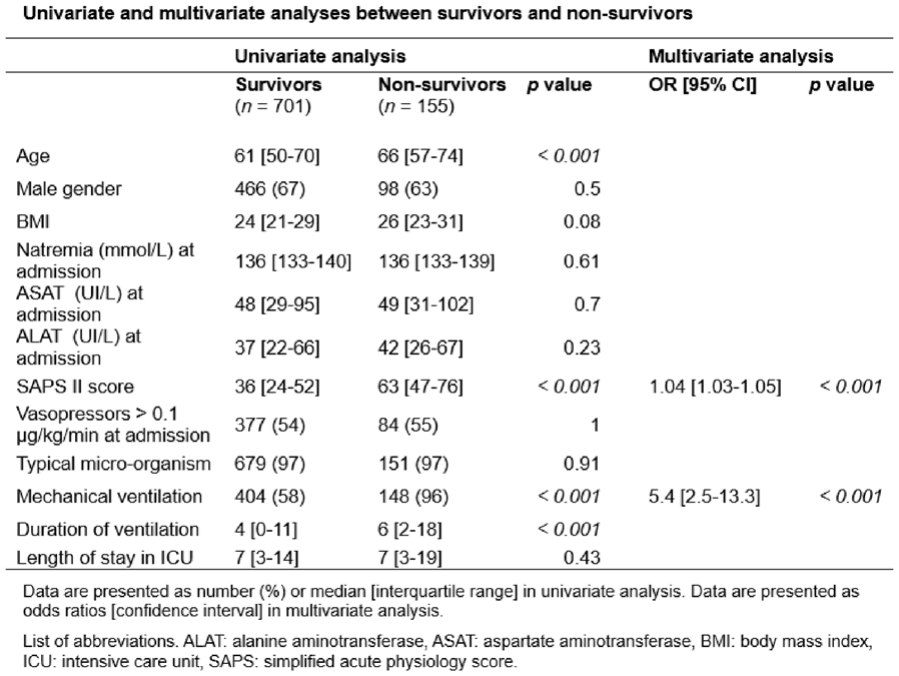


*Univariate and multivariate analyses between survivors and non-survivors*

### FC-232 Clinical features and outcome of influenza pneumonia in critically-ill immunocompromised patients

#### RAYMOND Matthieu^1^, MARTIN Maelle^1^, LAMOUCHE-WILQUIN Pauline^1^, BLONZ Gauthier^1^, DESCAMPS Paul^1^, AGBAKOU Maité^1^, DESMEDT Luc^1^, REIGNIER Jean^1^, LASCARROU Jean Baptiste^1^, CANET Emmanuel^1^

##### ^1^CHU Nantes, Nantes, France

###### Correspondence: Matthieu RAYMOND (matthieu.raymond@chu-nantes.fr)

*Annals of Intensive Care* 2022, **12(1):**FC-232

**Rationale:** Immunocompromised patients are at risk of severe viral infections which may require ICU admission. Flu is responsible of yearly epidemic waves and influenza pneumonia may deteriorate into acute respiratory syndrome (ARDS). However, data on the outcome of influenza pneumonia in critically-ill immunocompromised patients are limited.

**Patients and methods/Materials and methods:** We conducted a single-center observational study. All patients admitted to the ICU of a French university-affiliated hospital for influenza pneumonia between December 1st 2016 and February 28th 2020 were included. The main objective was to compare the clinical features and outcome of critically-ill patients with flu according to their immunocompromised status.

**Results:** Overall, 137 patients (age 60 (± 2.77) years-old, 58.4% male) were included, of whom 58 (42.34%) were intubated. Forty-three (31.4%) patients were immunocompromised and 94 (68.6%) had no underlying immunosuppression. The main causes of immunosuppression were solid tumor (39.53%), hematological malignancies (37.21%) and solid-organ-transplantation (13.95%). ICU mortality was 13.97%, with mortality of immunocompromised patients being three-times that of patients without immunosuppression (25.58% versus 8.6%, p = 0.015). Immunocompromised patients were younger (58 vs 64 years-old, p = 0.02), had a higher Charlson comorbidity index (5.77 vs 3.12, p < 0.0001) and a worse Performance Status (1.72 vs 1.43, p = 0.02) compared to non-immunocompromised patients. In contrast, severity scores (SAPS II), hypoxemia (PaO_2_/FiO_2_ ratio), and ventilatory support at ICU admission were similar between the 2 groups. During the ICU stay, mechanical ventilation (MV) was implemented in 41.86% of immunocompromised patients and 42.55% of non-immunocompromised patients (p = 1). There was no difference in the rate of ventilator-associated pneumonia between the 2 groups (22.2 vs 22.5%, p = 1). Among MV patients, 10 (55.5%) immunocompromised patients developed severe ARDS compared to 13 (32.5%) non-immunocompromised patients (p = 0.02). On univariate analysis, factors associated with ICU mortality were older age, higher Charlson comorbidity index, worse Performance status, higher SAPS II, lower PaO2/FiO2 ratio, immunocompromised status, acute kidney injury, and the use of MV and vasopressors. On multivariable analysis, immunocompromised status was the only factor associated with increased ICU mortality (aOR 2.87 (1.07–7.69), p = 0.03).

**Conclusion:** Immunocompromised patients with severe influenza pneumonia were more likely to develop severe ARDS and had a threefold increase in ICU mortality compared to non-immunocompromised patients. Such difference was not explained by an increased rate of ICU-acquired infections, suggesting that influenza virus was by itself responsible of a more severe form of pulmonary disease in immunocompromised patients. Further studies are needed to assess whether new antiviral drugs would translate into better patients’ outcome.

**Compliance with ethics regulations:** Yes in clinical research.

### FC-233 Relationship between prehospital modified Charlson Comorbidity Index and septic shock 30-day mortality

#### PARFAIT Pierre-Arnaud^1^, JOUFFROY Romain^1,2^, GILBERT Basile^3^, TOURTIER Jean Pierre^4^, BLOCH-LAINE Emmanuel^5^, ECOLLAN Patrick^6^, BOULARAN Josiane^7^, BOUNES Vincent^3^, VIVIEN Benoit^2^, GUEYE Papa^8^

##### ^1^APHP - CHRU Ambroise Paré, Boulogne-Billancourt, France; ^2^Intensive Care Unit, Anaesthesiology, SAMU, Necker Enfants Malades Hospital - Assistance Publique - Hôpitaux Paris, Paris, France; ^3^Department of Emergency Medicine, SAMU 31, University Hospital of Toulouse, Toulouse, France; ^4^Paris Fire Brigade, Paris, France; ^5^Emergency Department, Cochin Hospital, Paris, France & Emergency Department, SMUR, Hôtel Dieu Hospital - Assistance Publique - Hôpitaux Paris, Paris, France; ^6^Intensive Care Unit, SMUR, Pitie Salpêtriere Hospital - Assistance Publique - Hôpitaux Paris, Paris, France; ^7^SAMU 31, Castres Hospital, Castres, France; ^8^SAMU 972 University Hospital of Martinique, Fort-De-France Martinique, France

###### Correspondence: Romain JOUFFROY (romain.jouffroy@gmail.com)

*Annals of Intensive Care* 2022, **12(1):**FC-233

**Rationale:** In the prehospital setting, early identification of septic shock (SS) at risk of poor outcome is mainly based on clinical vital signs alteration evaluation. The Charlson Comorbidity Index (CCI) is an in-hospital tool used for burden of co-morbidity assessment. We report the relationship between the modified prehospital CCI, and 30-day mortality of SS patients initially cared for in the prehospital setting by a mobile ICU (MICU).

**Patients and methods/Materials and methods:** SS patients defined according to the 2012 sepsis-2 conference cared for by MICU between February 2017 and December 2021 were retrospectively analyzed. The modified prehospital CCI) calculation was based on the available comorbid conditions collected in the prehospital setting. A threshold of ≥ 6, was chosen according to previous results.

**Results:** Five-hundred and twenty-nine patients with a mean age of 70 ± 15 years old were analyzed. Presumed origin of septic shock was mainly pulmonary (44%), digestive (25%) or urinary (16%). 30 day-mortality reached 31%. Propensity score analysis using a multivariate logistic regression of Inverse Probability Treatment Weighting, found a significant association between the 30-day mortality in the modified prehospital CCI ≥ 6: aOR = 1.11 [1.02–1.21], p < 10^−3^.

**Conclusion:** Among septic shock patients initially managed by a MICU in the prehospital setting, a significant association between 30-day mortality and the elevation of modified prehospital CCI of at least 6 is useful to early identify a poorer outcome.

**Compliance with ethics regulations:** Yes in clinical research.

### FC-234 Adequacy of probabilistic prehospital antibiotic therapy for septic shock

#### HASSAN Anna^1^, JOUFFROY Romain^1,2^, GILBERT Basile^3^, TOURTIER Jean Pierre^4^, BLOCH-LAINE Emmanuel^5^, ECOLLAN Patrick^6^, BOULARAN Josiane^7^, BOUNES Vincent^3^, VIVIEN Benoit^2^, GUEYE Papa^8^

##### ^1^APHP - CHRU Ambroise Paré, Boulogne-Billancourt, France; ^2^Intensive Care Unit, Anaesthesiology, SAMU, Necker Enfants Malades Hospital, Assistance Publique - Hôpitaux de Paris, Université de Paris, Paris, France; ^3^Department of Emergency Medicine, SAMU 31, University Hospital of Toulouse, Toulouse, France; ^4^Paris Fire Brigade, Paris, France; ^5^Emergency Department, Cochin Hospital, Paris, France & Emergency Department, SMUR, Hôtel Dieu Hospital - Assistance Publique - Hôpitaux de Paris, Paris, France; ^6^Intensive Care Unit, SMUR, Pitie Salpêtriere Hospital - Assistance Publique - Hôpitaux de Paris, Paris, France; ^7^SAMU 31, Castres Hospital, Castres, France; ^8^SAMU 972 University Hospital of Martinique, Fort-De-France Martinique, France

###### Correspondence: Romain JOUFFROY (romain.jouffroy@gmail.com)

*Annals of Intensive Care* 2022, **12(1):**FC-234

**Rationale:** Guidelines on sepsis management recommend early recognition, diagnosis and treatment, especially early antibiotic therapy (ABT) administration in order to reduce septic shock (SS) mortality. However, the adequacy of probabilistic prehospital ABT remains unknown.

**Patients and methods/Materials and methods:** From May 2016 to March 2021, all consecutive patients with SS managed by a prehospital mICU intervention were retrospectively analyzed.

**Results:** Among 386 patients retrospectively analyzed, 119 (33%) received probabilistic prehospital ABT, among which 74% received a 3rd generation cephalosporin: 31% cefotaxime and 42% ceftriaxone. No patient had a serious adverse effect related to ABT administration. Overall mortality rate on day-30 was 29%. Among the 119 patients with prehospital ABT, bacteriological identification was obtained for 81 (68%) patients with adequate prehospital ABT for 65 patients (80%) of which 10 (15%) deceased on day-30. Conversely, among the 16 (20%) patients with inadequate prehospital ABT, 9 patients (56%) deceased on day-30. Prehospital adequate ABT was significantly different between alive and deceased patients on day-30 (p = 4.10^–3^). After propensity score matching, a significant association between adequate prehospital ABT administration and day-30 mortality was observed (aOR = 0.09 [0.01–0.47]). Inverse probability treatment weighting with multivariable logistic regression reported a day-30 mortality decrease in the adequate prehospital ABT group: aOR = 0.70 [0.53–0.93].

**Conclusion:** Among SS managed by a mICU, probabilistic prehospital ABT is most of the time adequate and associated with a day-30 mortality decrease. Further prospective studies are needed to confirm these results and the weight of prehospital ABT in the prehospital bundle of care for SS.

**Compliance with ethics regulations:** Yes in clinical research.
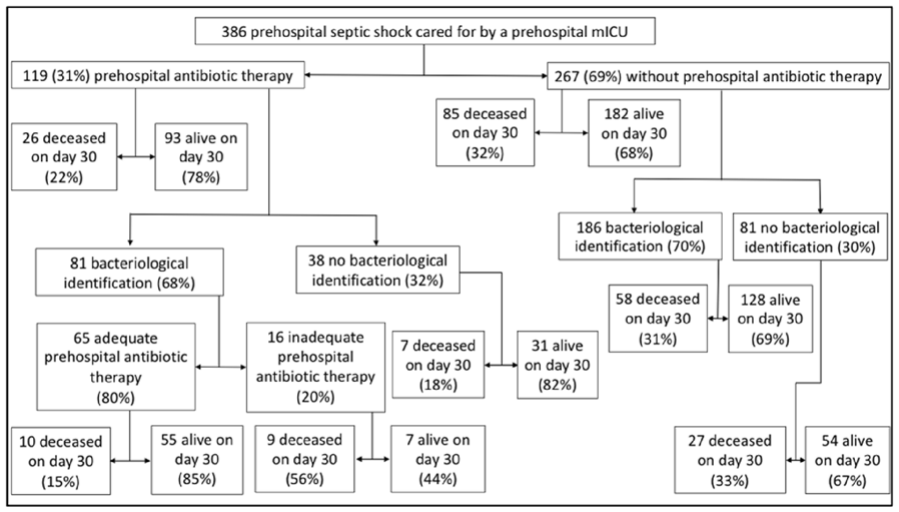


*Study flow chart*

### FC-235 Usefulness of Procalcitonin for cervicofacial cellulitis prognosis in intensive care

#### BENHAMZA Sabah^1,2^, LAZRAQ Mohamed^1,2^, MILOUDI Youssef^1,2^, BENSAID Abdelhak^1,2^, EL HARRAR Najib^1,2^

##### ^1^Hôpital 20 Août 1953, CHUIR, Casablanca, Maroc; ^2^Université Hassan II, faculté de médecine et de pharmacie, Casablanca, Maroc

###### Correspondence: Sabah BENHAMZA (benhamzasabah5@gmail.com)

*Annals of Intensive Care* 2022, **12(1):**FC-235

**Rationale:** Cervicofacial cellulitis (CFC) is a severe infection of the subcutaneous cellular tissue, and is one of the most serious head and neck infectious emergencies [1]. Procalcitonin (PCT) is a “specific marker of bacterial infection” [2]. Our objective was to evaluate the prognostic value of PCT in CFC in intensive care unit.

**Patients and methods/Materials and methods:** retrospective cohort over 6 years which covered all patients treated for CFC in intensive care unit at the August 20 hospital in Casablanca. All patients with less than two PCT assays were excluded.

**Results:** 47 patients were admitted for CFC during the duration of our study, 30 of whom had at least 2 PCT values. The annual incidence of CFC was 8 cases/year. The average age was 41 years old. The sex ratio was 1. The main medical history of admitted patients was diabetes (33.33%), hypertension (22.33%) and poor oral health (22.33%). The infectious gateway was dental in 53.33%. The average consultation time was 12 days. The main complications were upper aerodigestive tract obstruction (43.3%), mediastinitis (40%) and meningeal extension (13.3%). Positive PCT was found in 60% of cases. In bivariate analysis, positive PCT at admission was associated with mortality (p = 0.04), but not with length of stay (p = 0.49) or septic shock (p = 0.52) neither other clinical or biological parameters. The ascending kinetics of PCT was associated with the duration of hospitalization (0.017) but not with death.

**Conclusion:** Elevated PCT at admission seems to have prognostic rather than diagnostic value, multicenter studies should confirm this result.

**Reference 1:** 1) Bennani-Baïti, A A et al. “Cervicofacial cellulitis: The impact of non-steroidal anti-inflammatory drugs. A study of 70 cases.” European annals of otorhinolaryngology, head and neck diseases vol. 132,4 (2015): 181–4. https://doi.org/10.1016/j.anorl.2015.06.004

**Reference 2:** 2) Venet, C., et al. “Marqueurs biologiques de l’infection en réanimation chez l’adulte?: place de la procalcitonine Biological markers of infection in critically ill adult patients: role of procalcitonin.” Réanimation, vol. 11, no. 3, May 2002, pp. 156–

**Compliance with ethics regulations:** Yes in clinical research.

### FC-236 Characterization of endotracheal tube biofilm in critically ill patients, lessons from a novel microbiological compartment

#### MALDINEY Thomas^1^, PINEAU Valentin^2^, NEUWIRTH Catherine^2^, OUZEN Linda^2^, EBERL Isabelle^2^, JEUDY Géraldine^2^, DALAC Sophie^2^, PIROTH Lionel^2^, BLOT Mathieu^2^, SAUTOUR Marc^2^, DALLE Frédéric^2^, ABDULMALAK Caroline^1^, TER SCHIPHORST Romain^1^, PUGLIESI Paul-Simon^1^, POUSSANT Thomas^1^, OGIER-DESSERREY Agathe^1^, FOURNEL Isabelle^2^, DE GIRAUD D'AGAY Melchior^2^, JACQUIER Marine^2^, LABRUYÈRE Marie^2^, APTEL François^2^, ROUDAUT Jean-Baptiste^2^, VIEILLE Thibault^2^, ANDREU Pascal^2^, PRIN Sébastien^2^, CHARLES Pierre-Emmanuel^2^, HAMET Maël^1^, QUENOT Jean-Pierre^2^

##### ^1^William Morey Hospital, Chalon-Sur-Saône, France; ^2^Dijon University Hospital, Dijon, France

###### Correspondence: Thomas MALDINEY (thomas.maldiney@gmx.com)

*Annals of Intensive Care* 2022, **12(1):**FC-236

**Rationale:** Biofilm (BF) is considered to have a major impact on the development of ventilator-associated pneumonia (VAP) in intensive care unit (ICU) [1]. Despite an increasing amount of relevant data regarding the formation of BF [2], significant results are still lacking to understand the potential implication of endotracheal tube (ETT)-BF dispersal and better characterize the impact of its mesostructure and microbiological singularity on the occurrence of VAP.

**Patients and methods/Materials and methods:** We conducted a multicenter retrospective observational study in both William Morey general hospital and the University Hospital of Dijon between March and May 2021. Patients admitted in the ICU who required for more than 48 h invasive mechanical ventilation (MV) were screened for subsequent ETT characterization. Following tracheal extubation, we collected ETT, studied the mesostructural morphology of ETT-deposited BF with confocal microscopy (CM) and compared its microbiological characteristics to other colonization/infection sites.

**Results:** A total of 64 ETT were included in the study. Confocal microscopy acquisitions of ETT sections revealed a significant correlation between BF thickness and MV duration as well as two main morphological aspects of ETT-deposited BF (Fig. 1A to 1D): a thinner continuous ribbon-shaped (rs) aspect, less likely monobacterial and predominantly associated with Enterobacter spp., *Streptococcus pneumoniae* or Viridans streptococci, and a thicker discontinuous mushroom-shaped (ms) appearance more likely correlated with the association of bacterial and fungal species in respiratory samples (Fig. 1E). The microbiological characterization of ETT-deposited BF and subsequent cartography allowed the identification of additional species only in BF (Fig. 1F and 1G) with higher acquired resistance in more than 80% of analyzed BF phenotypes when compared to other colonization sites from the patient’s environment.

**Conclusion:** The present study reveals novel morphological and microbiological data regarding ETT-deposited BF characterization and formation in ICU patients. BF sampling as well as microbiological phenotypic cartography returned precious information regarding the acquisition of BF-exclusive species with preeminent antibiotic resistance and would therefore constitute a clinical value added in the perspective of future ETT-deposited BF-based antimicrobial stewardship in critically ill patients at increased risk for VAP.

**Reference 1:** Fernández-Barat L, Torres A. Biofilms in ventilator-associated pneumonia. Future Microbiology. déc 2016;11(12):1599–610.

**Reference 2:** Thorarinsdottir HR, Kander T, Holmberg A, Petronis S, Klarin B. Biofilm formation on three different endotracheal tubes: a prospective clinical trial. Crit Care. déc 2020;24(1):382.

**Compliance with ethics regulations:** Yes in clinical research.
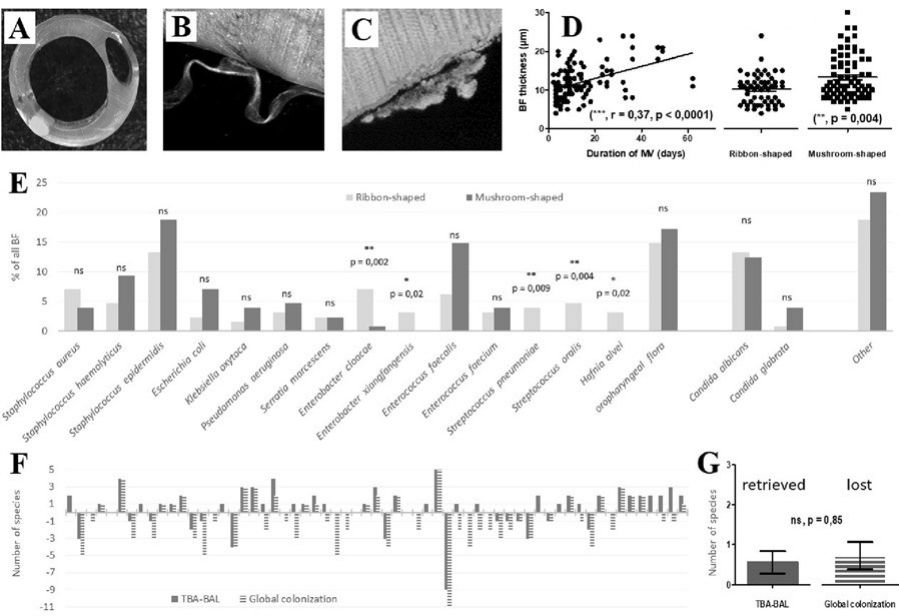


*ETT-deposited BF characterization. A) ETT section. B) CM of rs BF (bar, 50 µm). C) CM of ms BF (bar, 25 µm). D) MV duration-BF thickness correlation. E) BF bacterial-fungal distribution. F–G) BF lost-found differential species/patient (F) or median (G).*

### FC-237 Inhaled bacteriophage therapy in a porcine model of pneumonia caused by *Pseudomonas aeruginosa* during mechanical ventilation

#### GUILLON Antoine^1,2^, PARDESSUS Jeoffrey^2^, L’HOSTIS Guillaume^3^, FEVRE Cindy ^3^, BARC Céline^4^, DALLONEAU Emilie^2^, JOUAN Youenn^1,2^, BODIER Elsa^1,2^, PEREZ Yonatan^1,2^, THOREY Camille^1,2^, MEREGHETTI Laurent ^1^, CABRERA Maria^2^, RIOU Mickael^4^, VECELLIO Laurent^2^, LEGUELLEC Sandrine^5^, VOURC'H Nathalie ^2^.

##### ^1^CHRU Tours, Tours, France; ^2^INSERM, Centre d'Etude des Pathologies Respiratoires, U1100, Tours, France; ^3^Pherecydes Pharma, Research and Development, Romainville, France; ^4^INRAE, UE-1277 Plateforme d'infectiologie expérimentale (PFIE), Nouzilly, France; ^5^DTF-Aerodrug, Faculté de Médecine, Tours, France

###### Correspondence: Antoine GUILLON (antoine.guillon@univ-tours.fr)

*Annals of Intensive Care* 2022, **12(1):**FC-237

**Rationale:** *Pseudomonas aeruginosa* is a main cause of ventilator-associated pneumonia (VAP) with drug-resistant bacteria. Bacteriophage therapy has experienced resurgence to compensate for the limited development of novel antibiotics. However, phage therapy is limited to a compassionate use so far, resulting from lack of adequate studies in relevant pharmacological models. We used a pig model of pneumonia caused by *P. aeruginosa* that recapitulates essential features of human disease to study the antimicrobial efficacy of nebulized-phage therapy.

**Patients and methods/Materials and methods:** (i) Lysis kinetic assays were performed to evaluate in vitro phage antibacterial efficacy against *P. aeruginosa* and select relevant combinations of lytic phages. (ii) The efficacy of the phage combinations was investigated in vivo (murine model of *P. aeruginosa* lung infection). (iii) We determined the optimal conditions to ensure efficient phage delivery by aerosol during mechanical ventilation. (iv) Lung antimicrobial efficacy of inhaled-phage therapy was evaluated in pigs, which were anaesthetized, mechanically ventilated and infected with *P. aeruginosa.*

**Results:** By selecting an active phage cocktail and optimizing aerosol delivery conditions, we were able to deliver high phage concentrations in the lungs, which resulted in a rapid and marked reduction in *P. aeruginosa* density (1.5-log reduction, p < 0.001). No infective phage was detected in the sera and urines throughout the experiment.

**Conclusion:** Our findings demonstrated (i) the feasibility of delivering large amounts of active phages by nebulization during mechanical ventilation and (ii) rapid control of in situ infection by inhaled bacteriophage in an experimental model of P. *aeruginosa* pneumonia with high translational value.

**Compliance with ethics regulations:** Yes in animal testing.

### FC-238 Risk factors for a first episode of ventilator-associated pneumonia caused by Stenotrophomonas Maltophilia

#### CANIVET Clemence^1^, TEYSSEYRE Laura^1^, AUJOULAT Thomas^1^, CARON Margot^1^, NATIVEL Mathilde^1^, MILTGEN Guillaume^1^, VIDAL Charles^1^, ALLOU Nicolas^1^, PUECH Berenice^1^

##### ^1^CHU Felix Guyon, Saint Denis, Reunion

###### Correspondence: Bérénice PUECH (berenice.puech@gmail.com)

*Annals of Intensive Care* 2022, **12(1):**FC-238

**Rationale:** The incidence of ventilator-associated pneumonia caused by Stenotrophomonas maltophilia (SM-VAP) is on the rise. This disease is associated with increased morbidity and mortality in intensive care unit (ICU), notably due to intrinsic resistance and ineffective probabilistic antibiotic therapy. Our study aimed to determine the risk factors for a first episode of SM-VAP in ICU.

**Patients and methods/Materials and methods:** This single center retrospective study was conducted from 2010 to 2018 in the polyvalent ICU of University Hospital. All patients who developed ventilator-associated pneumonia (VAP) during their ICU stay were consecutively evaluated. Patients with a first episode of SM-VAP were compared to those with a first episode of VAP caused by another microorganism.

**Results:** A total of 89 patients developed a first episode of SM-VAP over the study period. In the group of patients with SM-VAP, infection was polymicrobial in 43.8% of cases and ICU mortality was 49.4%. After multivariate logistic regression analysis, the risk factors for a first episode of SM-VAP were: chronic respiratory failure (Odds Ratio (OR): 4.212; 95% Confidence Interval (CI): 1.776–9.989; p = 0.001), chronic renal failure (OR: 2.693; 95% CI: 1.356–5.352; p = 0.05), use of third-generation cephalosporins active against *Pseudomonas aeruginosa* (OR 2.862; 95% CI: 1.505–5.442; p = 0.001), and female sex (OR: 2.646; 95% CI: 1.458– .808; p = 0.001).

**Conclusion:** In our study, chronic respiratory failure, chronic renal failure, use of third-generation cephalosporins active against *P. aeruginosa*, and female sex were identified as risk factors for a first episode of SM-VAP.

**Compliance with ethics regulations:** Yes in clinical research.

### FC-239 Ventilator associated pneumonia among severe Covid-19 related ARDS patients

#### ZGHIDI Marwa^1^, BEN SAIDA Imen^1,2^, MAATOUK Iyed^1^, HAMDI Dhouha^1^, BOUBTANE Rihab^1^, BOUSSARSAR Mohamed^1^

##### ^1^Farhat Hached University Hospital, Medical Intensive Care Unit, Sousse,TUNISIA, Sousse, Tunisie; ^2^Research Laboratory N° LR12SP09. Heart Failure. Farhat Hached University Hospital, Sousse, Tunisia, Sousse, Tunisia, Tunisie

###### Correspondence: Marwa ZGHIDI (marwa_zghidi@outlook.fr)

*Annals of Intensive Care* 2022, **12(1):**FC-239

**Rationale:** The pandemic of coronavirus disease 2019 (covid19) has resulted in high rates of hospitalization in intensive care units (ICU) with widespread use of invasive mechanical ventilation (IMV) which exposes patients to the risk of ventilator-associated pneumonia (VAP). The objectives were: to determine the trends in frequencies and to analyze the characteristics, prognosis and risk factors of ventilator-associated pneumonia, in severe Covid-19 related ARDS patients.

**Patients and methods/Materials and methods:** This was a retrospective study of patients hospitalized with severe COVID-19 from March 2020 to October 2021, in a medical intensive care unit who required mechanical ventilation for more than 48 h. The study period was divided in three transition stages corresponding to each new adjustment. VAP was identified by using a combination of clinical, imaging and laboratory criteria. Univariate analysis and multivariable logistic regression were used to analyze the associated factors with VAP.

**Results:** During the study period, 442 patients with positive RT-PCR COVID-19 were hospitalized in the ICU. Two hundred fifteen (48.6%) were mechanically ventilated and met the inclusion criteria. Patients' characteristics were: mean age, 65 ± 12 years; male, 142(66%); median SAPSII on admission, 32 [26–38]; median SOFA on admission, 3 [1–4]; median IMV duration, 8 [4–12] days. The median length-of-stay was 11 [7–17] days. The overall mortality rate was 83%. Overall, 27(12.5%) patients developed VAP with density incidence rate estimated at 10.5 VAP/1000 ventilator days. The three identified periods were respectively, February–October 2020 (9 months) November 2020–April 2021 (6 months); and May–September 2021 (5 months).VAP frequencies were respectively, 6(28.5%), 12(11.3%) and 9(10.2%) at stages 1, 2 and 3. Patients with VAP had significantly longer duration of IMV (9[5–13] vs 7[4–11] days, p = 0.007), longer length of ICU stay (12[8–17] vs 10[7–16] days, p = 0.007), higher mortality rate (98.5% vs 79.5%, p = 0.027) and lower extubation rate (1.5% vs 31%, p = 0.001). The most frequently isolated microorganism was *Acinetobacter* *baumannii* 12(44%), followed by *Pseudomonas aeruginosa*, 7(26%); *Klebsiella pneumonia*, 4(14.8%) and *Candida albicans*, 2(7.4%). The only risk factor independently associated with VAP was the duration of IMV (RR, 1.15; 95% CI, [1.04–1.27]; p = 0.006).

**Conclusion:** Mechanically ventilated COVID-19 patients had a high frequency of ventilator-associated pneumonia, varying according to protection measures. IMV duration was identified as independent risk factor for VAP.

**Compliance with ethics regulations:** Yes in clinical research.

### FC-240 Impact of early steroids for COVID-19 related ARDS on ventilator-associated pneumonia

#### LAMOUCHE-WILQUIN Pauline^2^, SOUCHARD Jérôme^1^, PERE Morgane^2^, RAYMOND Matthieu^2^, ASFAR Pierre^3^, DARREAU Cédric^6^, REIZINE Florian^4^, HOURMANT Baptiste^7^, COLIN Gwenhaël^5^, RIEUL Guillaume^1^, KERGOAT Pierre^8^, FRÉROU Aurélien^9^, LORBER Julien^13^, AUCHABIE Johann^10^, LACOMBE Béatrice^13^, SEGUIN Philippe^4^, EGRETEAU Pierre-Yves^12^, MORIN Jean^2^, FEDUN Yannick^1^, CANET Emmanuel^2^, LASCARROU Jean-Baptiste^2^, DELBOVE Agathe^1^

##### ^1^CHBA Vannes, Vannes, France; ^2^CHU Nantes, Nantes, France; ^3^CHU Angers, Angers, France; ^4^CHU Rennes, Rennes, France; ^5^CHD Vendée, La Roche Sur Yon, France; ^6^Centre hospitalier du Mans, Le Mans, France; ^7^CHU Brest, Brest, France; ^8^Centre hospitalier de Cornouaille, Quimper, France; ^9^Centre hospitalier de St Malo, St Malo, France; ^10^Centre hospitalier de Cholet, Cholet, France; ^11^CH Morlaix, Morlaix, France; ^12^CH Saint Nazaire, St Nazaire, France; ^13^Centre hospitalier Bretagne Sud, Lorient, France

###### Correspondence: Agathe DELBOVE (agathe.delbove@ch-bretagne-atlantique.fr)

*Annals of Intensive Care* 2022, **12(1):**FC-240

**Rationale:** Few data of long-term outcome (including infectious complications) of early steroids course for COVID-19 related acute respiratory distress syndrome (ARDS) are available. This multicentric retrospective cohort study aims to determine the influence of an early course of steroids on ventilator-associated pneumonia (VAP) incidence in COVID-19 patients under mechanical ventilation (MV).

**Patients and methods/Materials and methods:** We included patients over 18 years old admitted in ICU for SARS-CoV 2 related ARDS under MV for at least 48 h in 15 ICUs. Inclusion period was from 1st January to 31st$ December of 2020. Early steroid group (ESG) was defined by administration of corticosteroids within 24 h of admission whereas patients in control group (CG) did not receive corticosteroids within 24 h of admission. The main study outcome was VAP incidence. Secondary outcomes included day 90 mortality, MV duration, other organ dysfunctions and VAP characteristics.

**Results:** We included 670 patients of mean age 67 years-old, of whom 369 in ESG and 301 in CG. Sex ratio (73.9% male), rate of hypertension (55.2%) and diabetes (31.2%) was similar between groups. ESG was slightly older (mean age 66.7 ± 10.2 vs 63.5 ± 11.3 years-old, p = 0.0001), had greater BMI (30.4 ± 6.0 vs 28.9 ± 5.4 kg/m^2^, p = 0.0007), more immunosuppression (20.3% vs 10.6%, p = 0.0007) and a greater Charlson score (4.3 ± 2.5 vs 3.1 ± 2.2, p < 0.0001). Mean PaO_2_/FiO_2_ ratio upon admission was 143 ± 67. The cumulative incidence of VAP with death and extubation as competing events was higher in ESG, with a Hazard Ratio (HR) of 1.29 (CI95% [1.05; 1.58], p = 0.016). Antibiotic therapy was active on future identified bacteria on 80.8% vs 74.6% patients in ESG vs CG (p = 0.18). Bacterial antibiotic resistance of VAP was not different (OR 0.94, CI95% [0.58; 1.53], p = 0.81). In univariate analysis, there was no difference in day-90 mortality between groups with 30.9% in ESG vs 24.3% in CG (p = 0.111). In a multivariate analysis, there was no difference in day-90 mortality in both group (HR 1.15, CI95% [0.83; 1.60], p = 0.411). VAP was associated with a higher day-90 mortality (HR 1.63; CI95% [1.17; 2.28], p = 0.0003). Each increase of 1 years-old (HR 1.05, CI95% [1.03; 1.07], p < 0.001) and increase of 1 point of SOFA score (HR 1.15, [1.09;1.22], p < 0.0001) were also associated with an increase of mortality.

**Conclusion:** In this retrospective study, early use of steroids was associated with an increased risk of developing VAP in mechanically ventilated COVID-19 patients. Risk of death was higher in presence of VAP, but day-90 mortality did not reach statistical difference between groups. These results suggest VAP associated mortality could counterbalance steroids benefits in this population.

**Compliance with ethics regulations:** Yes in clinical research.

### FC-241 Prolonged antimicrobial treatment over than 7 days is associated with lower recurrence of VAP in patients with COVID-19 related ARDS

#### GAVAUD Ariane^1^, TERRACOL Laura^1^, BOUGHDIRI Ahmed^2^, CANOUI Etienne^1^, CHARPENTIER Julien^1^, POUPET Hélène^1^, POYART Claire^1^, MIRA Jean-Paul^1^, GASTLI Nabil^1^

##### ^1^Hôpital Cochin, Paris, France; ^2^Ecole des Ponts, Champs-Sur-Marne, France

###### Correspondence: Ariane GAVAUD (ariane.gavaud@gmail.com)

*Annals of Intensive Care* 2022, **12(1):**FC-241

**Rationale:** Patients hospitalized in the intensive care unit (ICU) for COVID-19 related acute respiratory distress syndrome (ARDS) are at high risk for recurring ventilator-acquired pneumonia (VAP), that are associated with poor outcome. The aim of this study was to describe characteristics of first episode and recurrences of VAP along the 3 first epidemic waves, and to identify determinants involved in the recurrence, including host, microbiological and treatment factors.

**Patients and methods/Materials and methods:** We conducted a retrospective observational study in a 24-bed ICU between March 2020 and May 2021. All adult patients admitted for severe COVID-19 pneumonia requiring > 48 h of mechanical ventilation were consecutively included. VAP episodes were diagnosed using clinical, radiological and quantitative microbiological criteria. VAP recurrence was defined as reappearance of VAP criteria after antibiotic discontinuation, with at least one bacterial species growing at a significant concentration from a new respiratory sample. Recurrence was considered a “relapse” if at least one of the initial causative bacterial strains grew at a significant concentration from a second distal sample; otherwise, it was considered a “superinfection”. Among patients with first episode of VAP, we investigated determinants associated with recurrence using logistic regression analysis with Cox-model. Primary outcome measurement was ICU discharge without VAP recurrence.

**Results:** Of 154 included patients, 115 (75%) presented a first episode of VAP. At least one recurrence occurred in 64/97 (66%) patients who survived from the first episode, with same pathogen in 49/64 (76%) patients (relapse). Enterobacteriaceae (56%) and *Staphylococcus aureus* (37%) were the most frequently isolated in the first episode, while recurrences recovered mainly Enterobacteriaceae (67%) and non-fermenting Gram-negative bacteria (53%). Infection with Methicillin-susceptible *S.aureus* was associated with an increased risk of recurrence within 7 days after antimicrobial treatment interruption (HR for absence of recurrence: 0.3 [0.1–0.9]) compared with other pathogens. The ICU mortality was 32% in our study population. Among patients who survived the first episode of VAP, recurrence was correlated with poorer outcome (mortality of 34% vs 9% if no recurrence; P = 0.006) Prolonged duration of antimicrobial treatment (> 7 days), as well as appropriate empiric antimicrobial treatment were associated with an increased probability of ICU discharge without VAP recurrence: HR 7.3 [2.2–24.1] and HR 2.7 [1.0–7.0].

**Conclusion:** In COVID-19 related ARDS patients treated for first VAP episode, prolonged duration of antimicrobial treatment may be a possible key to reduce VAP recurrences and have a beneficial impact on prognosis in ICU.

**Compliance with ethics regulations:** Yes in clinical research.
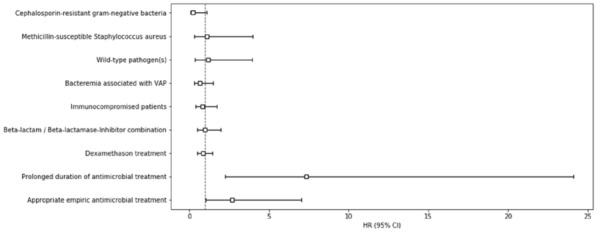


*Factors associated with discharge of ICU without recurrence of VAP*

### FC-242 Procalcitonin kinetics as a prognostic marker of ventilator-associated pneumonia

#### AQUIN Camille^1^, VIGNERON Clara^2^, CHARBONNEAU Philippe^3^, TRINH Vincent Quoc Huy^4^, QUENOT Jean-Pierre^1^, CHARLES Pierre-Emmanuel^1^

##### ^1^Centre Hospitalier Universitaire, Dijon, France; ^2^Hôpital Cochin, Paris, France; ^3^Centre Hospitalier de l'Université de Montréal, Montréal, Canada; ^4^Vanderbilt University Medical Center, Nashville, Etats-Unis

###### Correspondence: Camille AQUIN (camiaquin@yahoo.fr)

*Annals of Intensive Care* 2022, **12(1):**FC-242

**Rationale:** Ventilator-associated pneumonia (VAP) is a common and serious infection that is often difficult to diagnose. Biomarkers have been proposed as a diagnostic tool. The assay of procalcitonin (PCT) is not recommended for diagnostic purposes, but numerous studies have shown its prognostic value. The use of the kinetics, rather than an absolute value, seems more relevant.

**Patients and methods/Materials and methods:** Patients with an episode of VAP between December 2005 and October 2017 were identified. After excluding the missing data, 518 patients were included and divided into three groups according to their procalcitonin kinetics between D2 and D3 (Group 1: ΔPCTD2-D3 ≥ 30%; group 2: ΔPCTD2-D3 = [1–29%]; group 3: ΔPCTD2-D3 ≤ 0%).

**Results:** The 1st group (148 patients) had a 28-day mortality of 29,7%, the 2nd group (212 patients) of 42,9%, and the 3rd group (158 patients) of 50,6%. Mortality was greater in group 3, after univariate (p = 0.001) and multivariate (p = 0.014) analysis. Multivariate analysis identified age, cirrhosis, chronic renal failure, and belonging to group 3, as independent factors associated with mortality.

**Conclusion:** Our study suggests that the kinetics of procalcitonin between the following day and two days after the diagnosis of VAP (D2–D3) may be a prognostic marker of mortality. The identification of factors associated with mortality could allow the early identification of patients at risk in order to optimize their management.

**Compliance with ethics regulations:** Yes in clinical research.

### FC-243 Volume variation of endotracheal aspirates to predict onset of ventilator-associated pneumonia

#### DANCHE Estelle^1^, MEYER Sylvain^1^, GUICHARD Elie^2^, HERNANDEZ PADILLA Ana Catalina^1^, FEDOU Anne-Laure^1^, VIGNON Philippe^1^, BARRAUD Olivier^1^, FRANÇOIS Bruno^1^

##### ^1^CHU Dupuytren, Limoges, France; ^2^CHRU de Tours, Tours, France

###### Correspondence: Estelle DANCHE (estelle.danche@gmail.com)

*Annals of Intensive Care* 2022, **12(1):**FC-243

**Rationale:** Ventilator-associated pneumonia (VAP) is the most common cause of infection in the ICU but its diagnosis remains challenging. In this context, the CPIS, one of the most universal VAP score may help. Most of its component may be modified by “external factors” and therefore CPIS is in fact neither very specific neither very sensitive. Endotracheal secretions are one of the clinical criteria used for the diagnosis of VAP in CPIS and European and American guidelines. However, few studies analyzed the properties of those secretions, and their dynamics during mechanical ventilation. We hypothesized that endotracheal secretions volume modifications could help to early identify patients developing VAP.

**Patients and methods/Materials and methods:** We conducted a prospective study in adult patients requiring mechanical ventilation (MV) for at least 7 days. Included patients had to be free from acute or chronic pulmonary disease and from any acute infectious disease or antibiotic treatment before inclusion. Mostly neurological patients, including trauma were targeted to have a “very clean” population. Patients were included from intubation and all endotracheal secretions collected as part of routine care by bedside nurses were collected until D7. Daily secretions volume and appearance were measured as well as all CPIS components. All VAP events were retrospectively validated by a blinded adjudication committee. We analyzed the progression of CPIS during the first week of ventilation, and the variations in volume aspirates to identify different group pattern. Descriptive statistics were used to describe the study cohort. Spaghetti plot was used to visualize individual trajectories according to the VAP events.

**Results:** Forty-eight patients were analyzed [28 men; 58 (± 15) years old, BMI 26.4 (± 6.6) kg/m^2^, Apache II score 14.3 (± 5.3), Charlson Comorbidity Index of 2.8 (± 1.9)]. Ten (21%) patients developed VAP and 1556 ETA samples were analyzed. Although the volume aspirate tends to increase during mechanical ventilation in all patients (figure), we did not find differences between patients with or without VAP, and no pattern could be identified. The mean and standard deviation of volume aspirate for patients without VAP being high, it covered the value of patient with VAP. Concerning the CPIS, our study shows the absence of relevance of the CPIS, independently from the infectious status.

**Conclusion:** Our study did not permit to identify pattern for patients at risk of VAP, using the daily volume aspirate or the CPIS.

**Compliance with ethics regulations:** Yes in clinical research.
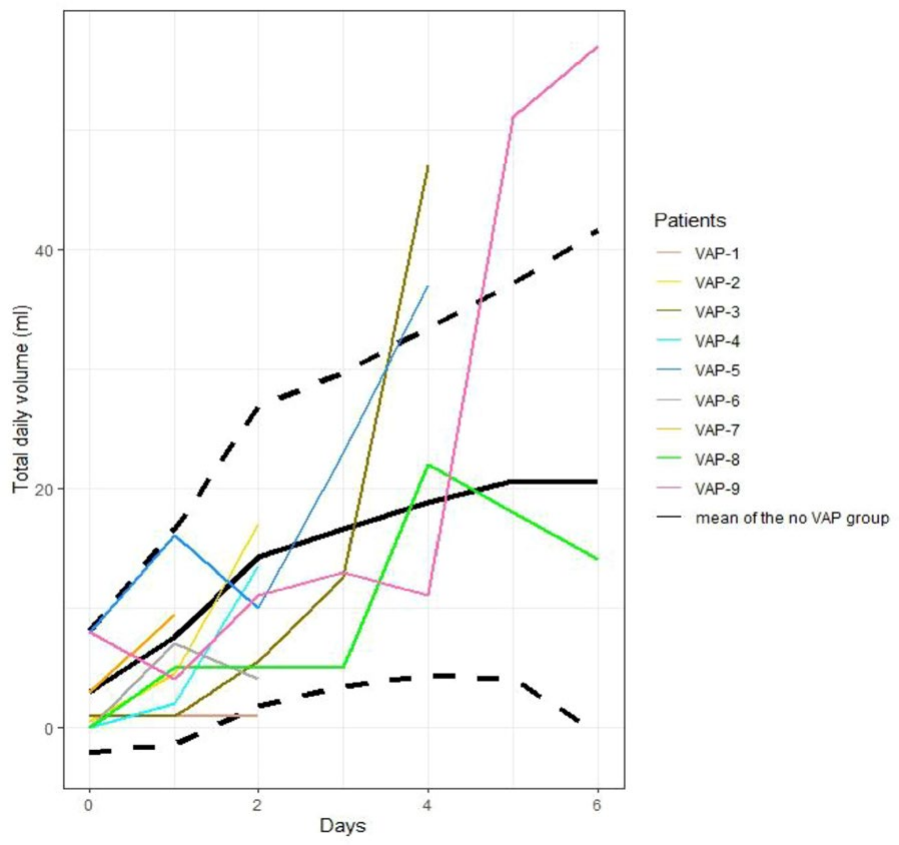


*Kinetics of daily volume aspirate during the first 7 days of ventilation. Coloured lines: patients who developed VAP (stops the day before VAP) Dashed and black lines: the mean* ± *the standard deviation for the patients without VAP.*

### FC-244 Serial measurements of serum procollagen III and hyaluronic acid correlate with lung fibroproliferation during COVID-19 associated acute respiratory distress syndrome

#### ALLARDET-SERVENT Jerome^1^, DEHAENE Aurelie^1^, PLAUZOLLES Anne^4^, SOMMA Claude^2^, BOURLARD Donatienne^6^, SECQ Veronique^3^, BOUAMRA Yannis^5^, KHIRI Hacene^4^, FOREL Jean-Marie^3^, D'JOURNO Xavier Benoit^3^

##### ^1^HOPITAL EUROPEEN MARSEILLE, Marseille, France; ^2^HOPITAL DE LA TIMONE, Marseille, France; ^3^HOPITAL NORD, Marseille, France; ^4^LABORATOIRE ALPHABIO, Marseille, France; ^5^LABORATOIRE EUROFINS BIOMNIS, Ivry Sur Seine, France; ^6^LABORATOIRE EUROFINS PATHOLOGIE, Marseille, France

###### Correspondence: Jerome ALLARDET-SERVENT (j.allardetservent@hopital-europeen.fr)

*Annals of Intensive Care* 2022, **12(1):**FC-244

**Rationale:** Fibroblastic proliferation with organizing fibrosis occurred in the late stage of COVID-19 related acute respiratory distress syndrome (CARDS)^1^. Monitoring lung fibroproliferation would help to timely introduce rescue corticosteroids therapy (CST). This study investigates the value of serial measurements in serum of three extracellular matrix (ECM) biomarkers to detect lung fibroproliferation during CARDS^2^.

**Patients and methods/Materials and methods:** Five patients with lung fibrosis following CARDS were studied. All patients had reticulations and bronchiectasis on CT scans and three had lung biopsy with confirmed histologic fibrosis. Three patients who underwent elective thoracic surgery and had normal preoperative lung function served as controls. The concentrations of amino-terminal propeptide of type I and type III procollagens (PINP, PIIINP) and hyaluronic acid (HA) were measured weekly using radioimmunoassay (UniQ^®^ RIA, Aidian Oy, Espoo, Finland) and immunoturbidimetric method (HA LT, FUJIFILM Wako Chemicals, Neuss, Germany). A PIIINP level above 16 µg/L indicated high probability of lung fibrosis^2^. Cut-offs for PINP and HA have not yet been established. Comparisons were performed using Mann–Whitney U test and correlation with the Rho Spearman’s test. Data are presented as median and interquartile.

**Results:** The five fibrotic patients were mostly male (80%), aged 58 [52,70] years, and had normal respiratory function prior admission. All received mechanical ventilation with a median duration of 37 [32,71] days and three required veno-venous extracorporeal membrane oxygenation during 20 [13,21] days. Only one patient survived (Case F-5). The average concentrations of PIIINP and HA over the stay were higher in fibrotic patients compared to controls, respectively 14 [9.2, 22] vs. 2.5 [2.2, 4] µg/L (p = 0.005) and 311 [143, 699] vs. 37 [22, 80] µg/L (p = 0.007). In contrast, PINP did not differ between cases (37.5 [21, 53] µg/L) and controls (40 [19.8, 44.5] µg/L; p = 0.64). Figure 1 displayed the time course of biomarkers in fibrotic individuals. The time-weighted average concentrations of PIIINP and HA correlated with the amount of histologic lung fibrosis (respectively ρ = 0.749, p = 0.032 and ρ = 0.687, p = 0.059). Serum PIIINP levels overcrossed the predefined fibrosis threshold in all cases within a median delay of 16 (14,25) days.

**Discussion:** This proof-of-concept study is the first to describe the course of three ECM biomarkers in the serum of patients with CARDS related lung fibrosis. Only PIIINP and HA exhibited dynamic variations, increased average concentrations and correlated with histologic fibrosis. PIIINP levels exceeded the predefined fibrosis threshold in all fibrotic patients.

**Conclusion:** The non-invasive monitoring of lung fibroproliferation using serial measurement of PIIINP in serum represents a promising strategy and warrants larger investigations.

**Reference 1:** Carsana, L et al. Pulmonary Post-Mortem Findings in a Series of COVID-19 Cases from Northern Italy: A Two-Centre Descriptive Study. The Lancet Infectious Diseases 2020, 20 (10), 1135–1140.

**Reference 2:** Forel, J.-M et al. Type III Procollagen Is a Reliable Marker of ARDS-Associated Lung Fibroproliferation. Intensive Care Med 2015, 41 (1), 1–11.

**Compliance with ethics regulations:** Yes in clinical research.
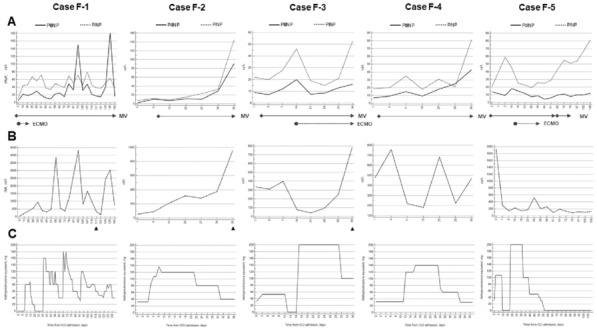


*Time course of serum biomarkers concentration among the five CARDS related fibrotic patients. A: PINP (dashed line) and PIIINP (solid line). B: Hyaluronic acid (HA). C: Daily dose of corticosteroid therapy (CST) expressed as methylprednisolone equivalent.*

### FC-245 Impact of extent parenchymal lesions on critically ill COVID-19 patient outcomes

#### ALILA Ilef^1^, KHARRAT Sana^1^, BOUBTANE Rihab^1^, TURKI Olfa^1^, HAFDHI Malek^1^, BAHLOUL Mabrouk^1^, BOUAZIZ Mounir^1^

##### ^1^hopital Habib Bourguiba Sfax, Sfax, Tunisie

###### Correspondence: Ilef ALILA (ilefalila1323@gmail.com)

*Annals of Intensive Care* 2022, **12(1):**FC-245

**Rationale:** During the COVID-19 pandemic, thoracic computed tomography (CT) has proven to be a useful tool not only for diagnosis but also for assessing the extent of SARS-COV2 lesions and detecting possible thoracic complications. The aim of our study was to assess the risk of death or use of invasive mechanical ventilation (IMV) in patients hospitalized with SARS-COV2 pneumonia according to the percentage of lung parenchymal injury.

**Patients and methods/Materials and methods:** This was a retrospective study conducted in a medical intensive care unit for 16-month period from September 2020 to December 2021. Patients hospitalized with SARS-COV2 pneumonia who received a chest CT scan within the first 48 h of their stay in the ICU were collected.

**Results:** This study included 440 COVID-19 patients, with a mean age of 59.7 ± 14.3 years and a male predominance (sex ratio = 1.75). On admission, 328 patients had severe acute respiratory distress syndrome (ARDS) (74.5%) Invasive mechanical ventilation was required in 196 (44.5%) patients and overall mortality was of 48% (210 patients). Severe lung damage ranging among 75% of the lung parenchyma was estimated in 117 patients (26.6%), of which 99 patients had severe ARDS on admission (85%), 62 patients had required mechanical ventilation (53%; p = 0.036), and 64 had developed infections during hospitalization (55%, p = 0.036). Comparison between patient outcomes according to the extent of parenchymal lesions on CT is shown in Table I.

**Conclusion:** Initial quantitative assessment of COVID-19 lesions may predict severity and outcomes in patients admitted with SARS-COV2 pneumonia.

**Compliance with ethics regulations:** Yes in clinical research.
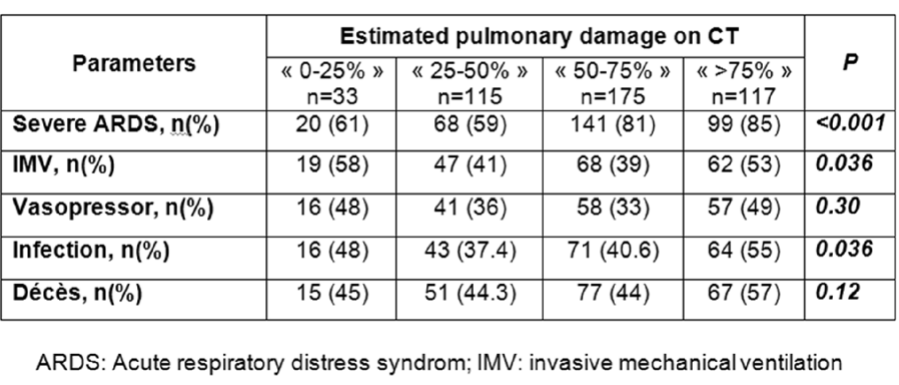


Table I: Patient outcomes according to CT extent of parenchymal lesions COVID-19

### FC-246 Are CT-scan severity scores accurate in predicting outcome in ICU COVID-19 patients: a cohort validation

#### MAATOUK Syrine^1^, LAHMAR Manel^1^, HAMMOUDA Zeineb^1^, SAAD Jamel^1^, BETBOUT M^1^, ZRIG Ahmed^1^, ABROUG Fekri^1^, BESBES Lamia^1^

##### ^1^CHU Fattouma Bourguiba Monastir, Monastir, Tunisie

###### Correspondence: Syrine MAATOUK (syrinemaatouk@gmail.com)

*Annals of Intensive Care* 2022, **12(1):**FC-246

**Rationale:** Several chest CT severity scores have been developed during the covid-19 pandemic. These scores were usually higher in patients with critical disease compared to those with less severe disease. The aim of this study is to compare the performance of two CT scoring methods in predicting the risk of intubation in patients initially managed with non-invasive ventilation (usually High Flow Nasal Oxygenation (HFNO)).

**Patients and methods/Materials and methods:** A single-center prospective analysis conducted during a 5-months period (from September 2020 to January 2021). All patients admitted to ICU with RT-PCR confirmed covid-19 infection and requiring HFNO as a respiratory support were included. All CT-scan images were analyzed according to two scores. The first one was widely used during the pandemic, gives precedence to the extension of the lesions, an extension expressed in a semi-quantitative way (< 25%, 25–50%, 50–75%, > 75%) and gives less importance to the nature of the lesions or their distribution, in particular in the antero-posterior dimension. The second score is an older one, first proposed during the H1N1 epidemic, and later on applied in several studies on ARDS. It divides each of the two lungs into 6 quadrants with 3 anteroposterior and longitudinal divisions. Weighing is assigned according to location and type of lung injury. This score ranges from zero to 72. We compared the performance of these two scores in predicting HFNO failure by calculating the area under the receiver operating characteristic (ROC) curves (AUC).

**Results:** During the study-period, a total of 137 patients were admitted for covid-19 ARDS. From this cohort, we included 106 patients with a positive RT-PCR test and available chest CT scan. This population included 76 (71.7%) male and 30 (28.3%) female with a median age of 62.5 years (IQR 53.75–69). All patients were put under HFNO at admission of whom 29 (27.4%) patients required invasive mechanical ventilation. The most common lesions of the disease included ground glass opacities (GGO) present in 103 (97.2%) patients, followed by consolidations (n = 83, 78.3%) and crazy paving pattern (n = 62, 58.5%). Both scores were well correlated (Pearson correlation coefficient of 0.75). The AUC for Score 1 was 0.73 (95% CI 0.623–0.843) with a p value and the CT score 2 had an AUC of 0.81 (95% CI 0.719–0.911) with a p value of 0.000 (Fig. 1).

**Conclusion:** These data suggest the potential role of CT scores for predicting the ventilatory outcome of SARS-CoV-2 patients. Both scores had a good discrimination for patients with higher risk of intubation.

**Compliance with ethics regulations:** N/A.
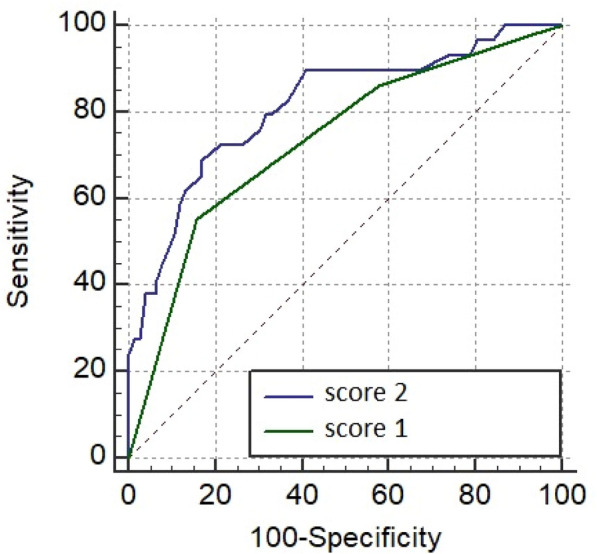


*ROC analysis of the score1 and score2 for prediction of requiring Invasive mechanical ventilation*

### FC-247 Is Chest Computed Tomography in COVID-19 inpatients at the ICU predictive of severity?

#### GHIGO Zoé^1^, SORIAL Didier^1^, EMERY Malo^1^, BABIN Matthias^1^, CARREIRA Serge^1^

##### ^1^Hopital Saint Camille, Bry-Sur-Marne, France

###### Correspondence: Zoé GHIGO (zoe.ghigo@gmail.com)

*Annals of Intensive Care* 2022, **12(1):**FC-247

**Rationale:** In December 2019, a new coronavirus, named SARS-CoV-2 (severe acute respiratory syndrome coronavirus 2), was discovered in the Hubei province of China, causing numerous cases of pneumonia in the World, to the point of getting the status of pandemic in March of 2020. Worldwide, the use of chest computed tomography (CT) to facilitate the management of the COVID-19 crisis has been uneven. Despite many scientific publications on COVID-19, very few of them have evaluated the prognostic use of CT at admission in the emergency room. Our study was conducted in order to determine a potential correlation between the percentage of lung damage on CT scans at admission in patients with positive COVID-19 RT-PCR and their prognosis.

**Patients and methods/Materials and methods:** This monocenter, observational, retrospective study aims to establish the prognostic value of the CT scan at admission in the emergency room based on the percentage of lung damage on CT scans at admission. Our secondary goals were to determine the clinical characteristics of patients admitted in the emergency room with a COVID-19 infection, and to report the non-clinical characteristics associated with a poor prognosis.

**Results:** From March 3rd to May 9th 2020, 293 patients admitted at the emergency room or the ICU for a COVID-19 infection and who underwent through a CT scan at admission were included in our study. After splitting our population between 3 groups, based on the percentage of lung damage on the CT scans (< 50%, > 50%, atypical), we found that 42% of patients presenting more than 50% of lung damage on the CT scan at admission were deceased or admitted at the ICU 7 days after admission (versus 20% and 12% in the 2 other groups, p < 0,01). In multivariable analysis, the sole percentage of lung damage on CT scans was associated with a poor prognosis (RR 3.37 [1.6–7.12], p = 0.02). The interpretation of the CT scan is bound to an inter-individual variation (Kappa-Cohen coefficient going from 0.19 to 0.53). The severity score of the CT scan was better associated with prognostic 7 days post admission when the CT was read by an expert radiologist (AUC = 0.714), versus automated severity score (AUC = 0.675) or by the on-call radiologist (AUC = 0.657).

**Conclusion:** CT scans at admission for patients with a COVID-19 infection are useful to predict admission in the ICU or mortality 7 days post admission.

**Compliance with ethics regulations:** N/A.

### FC-248 Role of echocardiography in the management and prognosis of patients admitted for acute respiratory failure in a Tunisian intensive care unit

#### MATOUK Iyed^1^, HAMMOUDA Zeineb^1^, BEDHIAFI Emir^1^, BEN AHMED Hedia^1^, HOURI Fadwa^1^, BOUGUEZZI Nabil^1^, LAHMAR Manel^1^, DACHRAOUI Fahmi^1^, ABROUG Fekri^1^, OUANES-BESBES Lamia^1^

##### ^1^University Hospital Fattouma Bourguiba of Monastir (Tunisia), Monastir, Tunisie

###### Correspondence: Iyed MATOUK (maatouk.yed@gmail.com)

*Annals of Intensive Care* 2022, **12(1):**FC-248

**Rationale:** Echocardiography is a crucial tool for the management of patients admitted in intensive care units (ICUs). Indeed, it is widely available and non-invasive. It offers vital data for diagnosis and treatment of the most common pathological conditions encountered in critically ill patients such as acute respiratory failure (ARF). We aimed to determine the role of echocardiography in the diagnosis, therapeutic changes and prognosis among patients admitted for ARF.

**Patients and methods/Materials and methods:** We conducted a retroprospective study in the ICU of a Tunisian University Hospital from January 2017 to June 2020. All patients aged over 18 years admitted for ARF and receiving at least one echocardiography in our department were included.

**Results:** A total of 346 patients received an ultrasound examination (476 echocardiography). Almost two thirds were male (72.5%). Median age was 65 [55, 72] years. The main medical history was respiratory diseases (61%). Fifty percent were under non-invasive ventilation and 48.3% under mechanical ventilation. Median duration of ventilation was 10 [5, 17]. Among the transthoracic echocardiography, 87.1% were performed within 48 h of admission. Main indication of echocardiography was suspicion of an increase in left ventricular filling pressures (38.7%) (mean E/E′ ratio was 10.4 ± 3.6, and mean E/A ratio was 1.4 ± 0.8). The main abnormalities of echocardiographic examinations were right heart dysfunction and left ventricular diastolic dysfunction (47.7% and 46% respectively). Echocardiography allowed hemodynamic monitoring in 25.7%. Echocardiography changed or clarified the initial diagnosis in 67.9%. In total, 67.9% of patients received a therapeutic change after an echocardiographic examination mainly furosemide (42.3%) and limitation of vascular filling (36%). Mortality rate was 30.9%. Almost 10% of the patients had RV/LV ratio > 1. The majority of patients who survived (90.9%) had RV/LV ratio < 1 (p = 0.6). Mean LVFE was 52.3 ± 13.1 in patient who died and 56.7 ± 9.2 in survivors (p = 0.002). Mean PAPS was 55.2 ± 16.5 in patient who died and 48.4 ± 13.2 in survivors (p = 0.05). The majority of patients who survived received a therapeutic change (64.4%) (p = 0.03).

**Conclusion:** Our study confirms the importance of echocardiographic examination in the diagnosis, hemodynamic monitoring, therapeutic changes, and prognosis in ICU, particularly in patients admitted for ARF. Thus, intensivists should have the necessary training for better patient management.

**Compliance with ethics regulations:** Yes in clinical research.

### FC-249 Radiographic Assessment of Lung Edema (RALE) score, oxygenation and compliances to predict End-Expiratory Lung Volume and Outcomes in mechanically ventilated patients

#### CAMPFORT Maëva^1^, OLIVIER Pierre-Yves^1^, STUDER Antoine^2^, PAVLOSKY Bertrand^1^, LEPROVOST Pierre^1^, DESPREZ Christophe^1^, YVIN Elise^1^, TAILLANTOU-CANDAU Mathilde^1^, CHEAN Dara^1^, COURTAIS Antonin^1^, MERDJI Hamid^2^, RICHARD Jean-Christophe^1^, MEZIANI Ferhat^2^, MERCAT Alain^1^, BELONCLE François^1^

##### ^1^CHU Angers, Angers, France; ^2^CHRU Strasbourg, Strasbourg, France

###### Correspondence: Maëva CAMPFORT (maevacampfort@gmail.com)

*Annals of Intensive Care* 2022, **12(1):**FC-249

**Rationale:** Beside oxygenation and respiratory mechanics, chest radiograph has been proposed to estimate the extent of lung injury and to predict the outcomes of mechanically ventilated patients at the bedside. The Radiographic Assessment of Lung Edema (RALE) score has thus been shown to correlate with edema in lung explants and with hypoxemia and survival in patients with acute respiratory distress syndrome (ARDS) (1). Its clinical value has however not yet been assessed in a general ICU population of mechanically ventilated patients with or without ARDS criteria. This study aimed to assess whether the RALE score can allow to predict the end-expiratory lung volume (EELV) and the outcomes in mechanically ventilated patients, differently from oxygenation or lung and respiratory system compliances.

**Patients and methods/Materials and methods:** All patients admitted to two ICUs and mechanically ventilated for any cause were prospectively enrolled. Gas exchange, respiratory mechanics (including esophageal pressure measurements), end-expiratory lung volume (EELV, using the nitrogen wash-out wash-in technique) and chest radiograph were assessed within 36 h after intubation when the patients were ventilated in volume-controlled ventilation (tidal volume = 6 mL/kg predicted body weight (PBW)) with a positive end-expiratory pressure of 5 cmH2O. The RALE score, in which the four quadrants of the chest are scored on a numerical scale for extent of consolidation and density of opacification, was independently assessed by two investigators.

**Results:** 217 patients were enrolled (124 men (83%), age 65 [56–77] years). 104 patients (48%) had ARDS criteria according to the Berlin definition. The RALE score was 19.9 [8.5–31.0]. The RALE score, respiratory system compliance, lung compliance and PaO_2_/FiO_2_ ratio were well correlated to EELV/PBW (r = − 0.39, p < 0.001; r = 0.51, p < 0.001; r = 0.60, p < 0.001 and r = 0.41, p < 0.001, respectively). The RALE score was lower in patients who survived at day 60 than in those who did not survive (16.5 [6.1–27.3] vs 22 [12.0–32.0], p < 0.05). The respiratory system compliance was not different between the survivors and the non-survivors at day 60 (48 [39–58] vs 46 [36–57] mL/cmH_2_O, p = 0.32), neither was the lung compliance (83 [60–101] vs 73 [49–94] mL/cmH_2_O respectively, p = 0.06). The PaO_2_/FiO_2_ ratio was lower in the survivors than in the non-survivors at day 60 (201 [147–263] vs 155, [103–257]), p < 0.05).

**Conclusion:** The RALE score measurement may allow to estimate the end-expiratory lung volume, similarly to the assessment of oxygenation or compliances and to predict mortality at day 60 in mechanically ventilated patients.

**Reference 1:** Warren MA, Zhao Z, Koyama T, Bastarache JA, Shaver CM, Semler MW, et al. Severity scoring of lung oedema on the chest radiograph is associated with clinical outcomes in ARDS. Thorax. 2018 Sep;73(9):840–6.

**Compliance with ethics regulations:** Yes in clinical research.

### FC-250 Early measurement of alveolar epithelial and endothelial biomarkers in serum predicts the need for mechanical ventilation in patients with COVID-19 pneumonia

#### ALLARDET-SERVENT Jerome^1^, HEZARD Nathalie^2^, PISSIER Christel^3^, BENAROUS Lucas^1^, HALFON Philippe^1^, MEGE Jean-Louis^4^, ALESSI Marie-Christine^2^, MORANGE Pierre^2^

##### ^1^HOPITAL EUROPEEN MARSEILLE, Marseille, France; ^2^HOPITAL DE LA TIMONE, Marseille, France; ^3^HOPITAL NORD, Marseille, France; ^4^INSTITUT HOSPITALO-UNIVERSITAIRE DE MALADIES INFECTIEUSES, Marseille, France

###### Correspondence: Jerome ALLARDET-SERVENT (j.allardetservent@hopital-europeen.fr)

*Annals of Intensive Care* 2022, **12(1):**FC-250

**Rationale:** Predicting early the need for mechanical ventilation (MV) during COVID-19 is highly relevant for triage. Biomarkers of alveolar epithelial and endothelial injuries might be helpful in this context. This study investigates the value of four biomarkers in patients hospitalized for moderate to severe COVID-19 pneumonia.

**Patients and methods/Materials and methods:** Fifty-four patients with laboratory confirmed COVID-19 pneumonia were prospectively included and compared to 20 healthy controls. The primary end-point was the need for MV during the stay. The concentrations of four biomarkers were measured in the serum within the first 48 h following hospital admission: Krebs von den Lungen 6 (KL-6), soluble receptor for advanced glycation end-products (sRAGE), angiopoietin-2 (ANG-2), and club cell protein 16 (CC-16). KL-6 was quantified by chemiluminescent enzyme immunoassay using the Lumipulse^®^ analyzer (Fujirebio, Tokyo, Japan) and the three other biomarkers using enzyme linked immunosorbent assays (Quantikine^®^ ELISA, R&D, Minneapolis, USA). All measurements by ELISA were duplicated and averaged. Biomarkers concentrations were compared between three groups (controls, COVID-No MV and COVID-MV) using the Kruskal–Wallis test with Conover post-hoc comparisons. Receiver operating characteristic (ROC) analysis was used to determine the area under the curve (AUC) of biomarkers to predict MV requirement and the best cut-off was determined using the Youden index method. Continuous data are presented as median and interquartile.

**Results:** COVID-19 patients were mostly male (76%), aged 63 [53,71] years, and 21 (39%) were obese. Eighteen COVID-19 patients (33%) received standard oxygen therapy in the ward while 36 (67%) required ICU admission. Among ICU patients, 13 (24%) received high flow oxygen therapy and 23 (43%) required MV. Four patients received extracorporeal membrane oxygenation. Seven patients (13%) deceased. Compared to healthy controls, the concentrations of KL-6 and sRAGE were higher in both group of COVID-19 patients (Fig. 1). ANG-2 was higher in COVID-MV than controls. CC-16 did not increase in COVID-19 patients. Among COVID-19 patients, those requiring MV had higher levels of KL-6, sRAGE and CC-16 compared to the others. Three biomarkers (sRAGE, CC-16 and KL-6) were predicting the need for MV but the sRAGE performed better with an AUC of 0.786 for a cut-off above 5449 pg/mL.

**Discussion:** This study is the first to compare the performance of four biomarkers. We observed similar range of KL-6 than others^1^ but higher sRAGE levels in MV patients^2^.

**Conclusion:** sRAGE, KL-6 and ANG-2 concentrations are increased in the serum of patients with COVID-19 pneumonia but sRAGE best predict the need for MV.

**Reference 1:** d’Alessandro, M et al. Serum KL-6 Concentrations as a Novel Biomarker of Severe COVID-19. J Med Virol 2020, 92 (10), 2216–2220.

**Reference 2:** Kapandji, N et al. Importance of Lung Epithelial Injury in COVID-19-Associated Acute Respiratory Distress Syndrome: Value of Plasma Soluble Receptor for Advanced Glycation End-Products. Am J Respir Crit Care Med 2021, 204 (3), 359–362.

**Compliance with ethics regulations:** Yes in clinical research.
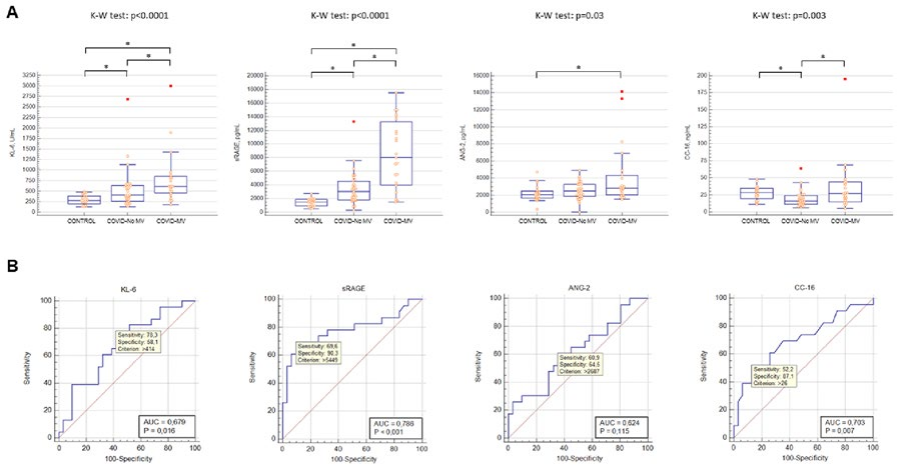


Biomarkers concentrations in the serum of healthy controls and COVID-19 patients. A: Box blot of biomarkers levels among controls, COVID-19 patients not requiring MV (COVID-No MV) and those requiring MV (COVID-MV). B: ROC curves to predict MV. * p < *0.05.*

### FC-251 Optimum time to initiate awake prone positioning in SARS-CoV-2 pneumonia to improve outcome

#### TRIFI Ahlem^1^, MASSEOUD Lynda^1^, OUHIBI Asma^1^, TLILI Bedis^1^, ABDENNEBI Cyrine^1^, DALY Foued^1^, TOUIL Yosr^1^, ABDELLATIF Sami^1^, BEN LAKHAL Salah^1^

##### ^1^Medical Intensive Care Unit, Teaching Hospital la Rabta, Faculty of Medicine of Tunis, Tunis, Tunisie

###### Correspondence: Ahlem TRIFI (trifiahlem2@gmail.com)

*Annals of Intensive Care* 2022, **12(1):**FC-251

**Rationale:** In patients with hypoxemic SARS-CoV-2 pneumonia, it becomes conventional that awake prone positioning (APP) under high nasal flow cannula (HNFC) improves oxygenation, reduces the need for mechanical ventilation (MV) and improve survival. And the earlier it was, the better outcome would be. But what about the optimum time to initiate APP in this kind of patients? Our aim was to determine the best time to initiate APP in hypoxemic SARS-CoV-2 pneumonia.

**Patients and methods/Materials and methods:** Prospective and analytical study including adult patients with acute hypoxemic confirmed SARS-CoV-2 pneumonia who received APP for at least one hour from HNFC administration. APP initiation times and all outcome parameters (primary: survival and secondary: MV use and ROX index) were recorded and compared. The optimum delay to initiate APP was determined by the ROC curve.

**Results:** We included 40 patients (31 male) with, a median age of 57 years, median SOFA of 4, and median signs onset of 11 days. Of them, 32.5% were obese, 37.5% have hypertension, 32.5% were diabetic and 5% were fully vaccinated. The intubation and death rates were respectively 45% and 50%. The median time to initiate APP from HNFC was 7,5 h [0–48] with extremes at 0 and 192 H. The number of APP sessions per day was at 2 [1–3] for a median duration at 4 h [2–6]. The time of APP from HNFC was significantly lower in survivors (n = 20) versus non survivors (n = 20) (14 [6–18] versus 48 [20–72], p = 0,033 respectively). Similarly, it was significantly lower in patients who not required MV (n = 22) (12.5 [4–14.75] versus 33 [18–72], p = 0.044 respectively). The best cutoff of APP from HNFC we showed predictor of survival was at 15 h with AUC/ROC = 0.725 ([0.563–0.887], p = 0.015), sensitivity at 65% and specificity at 85%. Initiating APP from HNFC within 15 h has less performance to predict the use of MV (AUC/ROC = 0.664 ([0.491–0.837], p = 0.07), sensitivity at 55.6% and specificity at 78%). Attached table displays the comparisons of the outcome’s variables according to the cutoff of APP from HNFC that we found (early: < 15H (n = 25) versus late: > 15H (n = 15)). In the early APP group, survival was significantly better and MV use was lower. MV duration and ICU stay tended to be lower in this same group with an average gain of 36 h.

**Conclusion:** In patients with hypoxemic SARS-CoV-2 pneumonia, the positioning on APP within 15 h of HNFC improved survival and reduced the need of MV.

**Compliance with ethics regulations:** Yes in clinical research.
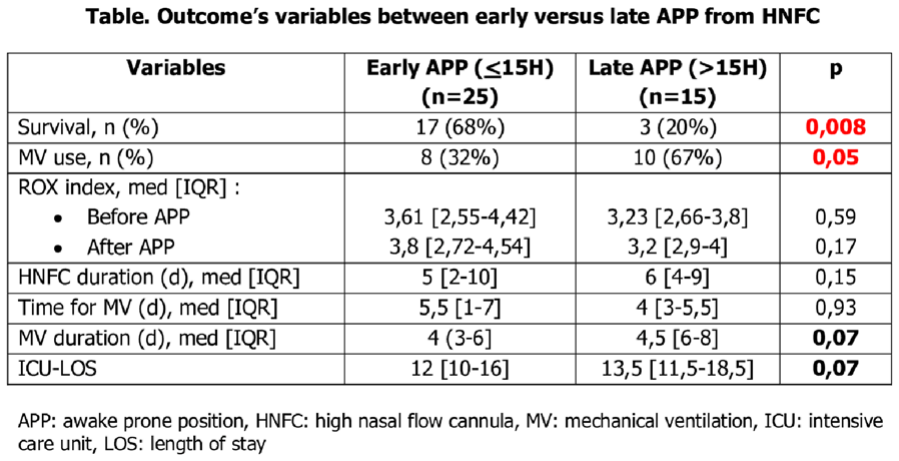


*Outcome’s variables between early versus late APP from HNFC*

### FC-252 Awake prone positioning for non-intubated patients with COVID-19 related acute hypoxemic respiratory failure: a systematic review and meta-analysis

#### PEREZ Yonatan^1^, LI Jie^2^, LUO Jian^3^, PAVLOV Ivan^4^, TAN Wei^6^, ROCA Oriol^7^, TAVERNIER Elsa^17^, KHARAT Aileen^8^, MACNICHOLAS Bairbre^9^, IBARRA-ESTRADA Miguel^10^, VINES David^2^, BOSCH Nicholas A.^11^, RAMPON Garrett^12^, SIMPSON Steven Q.^12^, WALKEY Allan J.^11^, FRALICK Michael^13^, VERMA Amol^14^, RAZAK Fahad^14^, HARRIS Tim^15^, LAFFEY John G.^9^, GUÉRIN Claude^16^, EHRMANN Stephan^5^

##### ^1^Hôpital de Hautepierre, Hôpitaux Universitaires de Strasbourg, Strasbourg, France; ^2^Department of Cardiopulmonary Sciences, Division of Respiratory Care, Rush University, Chicago, Etats-Unis; ^3^Respiratory Medicine Unit and Oxford NIHR Biomedical Research Centre, NDM Experimental Medicine, University of Oxford, Oxford, Royaume-Uni; ^4^Department of Emergency Medicine, Hôpital de Verdun, Montréal, Canada; ^5^CHRU de Tours, Médecine Intensive Réanimation, Tours, France; ^6^Department of respiratory and critical care medicine, the First Affiliated Hospital, China Medical University, Shenyang, Chine; ^7^Servei de Medicina Intensiva, Hospital Universitari Vall d’Hebron, Barcelone, Espagne; ^8^Department of respiratory medicine, Geneva University Hospital, Genève, Suisse; ^9^Department of Anesthesia and Intensive Care Medicine, Galway University Hospitals and School of Medicine, National University of Ireland, Galway, Irlande; ^10^Unidad de terapia Intensiva, Hospital Civil Fray Antonio Alcalde, Guadalajara, Mexique; ^11^Boston University School of Medicine and Boston Medical Center, Boston, Etats-Unis; ^12^University of Kansas Medical Center, Kansas City, Etats-Unis; ^13^Department of Medicine, Sinai Health System, and Department of Medicine, University of Toronto, Toronto, Canada; ^14^Li Ka Shing Knowledge Institute, St. Michael’s Hospital, Unity Health Toronto, and Department of Medicine, University of Toronto, and Institute of Health Policy, Management and Evaluation, University of Toronto, Toronto, Canada; ^15^Hamad Medical Corporation, Doha, Qatar; ^16^Médecine Intensive Réanimation, Hôpital Edouard Herriot, Lyon, France; ^17^Clinical Investigation Center, INSERM 1415, CHRU de Tours, Tours, France

###### Correspondence: Yonatan PEREZ (yonatperez@gmail.com)

*Annals of Intensive Care* 2022, **12(1):**FC-252

**Rationale:** Since the early phases of COVID-19 pandemic, prone positioning of non-intubated patients has been broadly utilized for patients with acute hypoxemic respiratory failure (AHRF) related to COVID-19. This intervention showed improvement of oxygenation. But the results of randomized controlled studies (RCTs) about intubation and mortality are contradictory. We aimed to assess the efficacy and safety of awake prone positioning (APP) including in relevant subpopulations.

**Patients and methods/Materials and methods:** We conducted a systematic review and meta-analysis according to the Preferred Reporting Items for Systematic Reviews and Meta-Analyses (PRISMA) recommendations. Two independent groups of investigators searched MEDLINE, Embase, PubMed, Web of Science, Scopus, MedRxiv, BioRxiv, Google Scholar, and ClinicalTrials.gov for eligible studies from January 1st, 2020 to November 8th, 2021. RCTs comparing APP (intervention group) and supine position (control group) for non-intubated patients hospitalized for COVID-19 related AHRF were included. Random-effects meta-analysis was used to pool individual studies. Observational studies of APP comprising a control group were also included as a sensitivity analysis. Primary outcome was reported need for intubation. Secondary outcomes included: reported all-cause mortality, need for escalating respiratory support, Intensive Care Unit (ICU) admission, ICU length of stay, hospital length of stay and safety outcomes.

**Results:** 29 studies were included, with 10 RCTs comprising 1,985 patients and 19 non-RCTs totalizing 2,669 patients. In 10 RCTs, compared to the supine position, APP significantly reduced the need for intubation in the overall population (risk ratio [RR] 0.84, 95% confidence interval [CI95] 0.72 to 0.97) (Fig. 2A). In the subgroup analysis, this significant reduction of intubation was observed in the studies with patients on advanced respiratory support (high-flow nasal cannula or noninvasive ventilation) at enrolment (RR 0.83, CI95 0.71 to 0.97), and those enrolled in ICU settings (RR 0.83, CI95 0.71 to 0.97) but not in patients receiving conventional oxygen therapy or enrolled in general wards. APP did not significantly reduce mortality (RR 1.00, CI95 0.70 to 1.44) (Fig. 2B). APP did not demonstrate a benefit on need for escalation of respiratory support, ICU admission, ICU and hospital length of stay. No serious adverse events associated with APP (in particular, cardiac arrest) were reported.

**Conclusion:** This systematic review and meta-analysis found that in patients with COVID-19 related AHRF, APP reduced the need for intubation, particularly in those patients requiring advanced respiratory support at enrollment and in those enrolled in the ICU but did not demonstrate a benefit on mortality and others secondary outcomes.

**Compliance with ethics regulations:** Yes in clinical research.
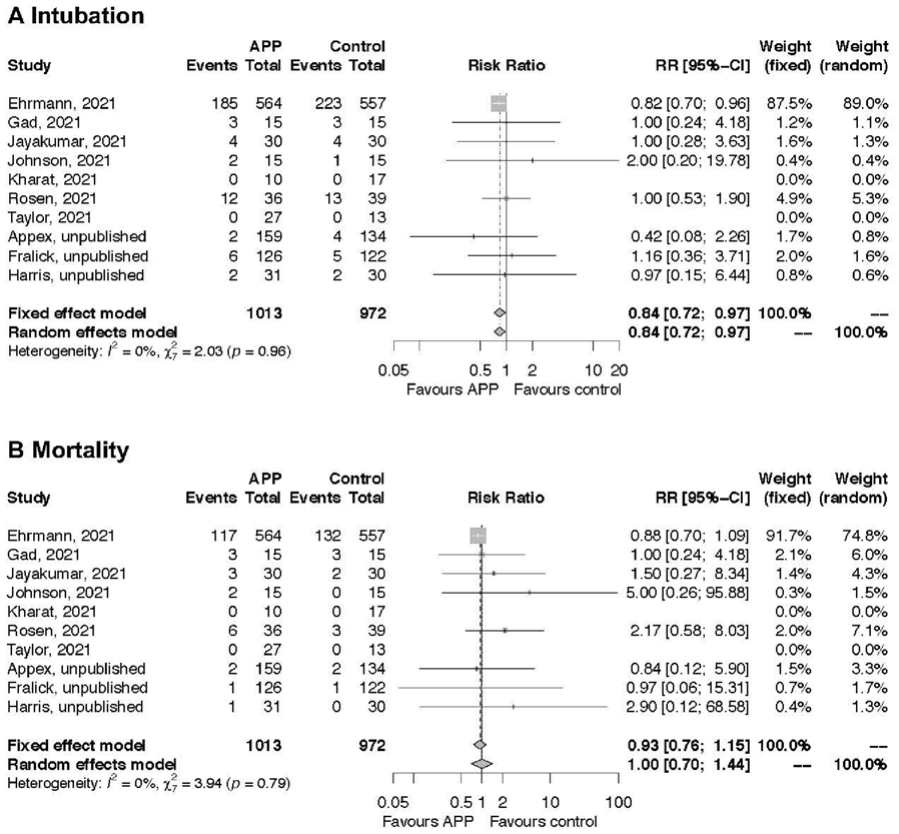


*Intubation and mortality for RCTs*

### FC-253 Extended prone positioning duration for COVID-related ARDS: a retrospective study

#### WALTER Thaïs^1^, ZUCMAN Noémie^1^, MULLAERT Jimmy^2,4^, THIRY Ingrid^1^, GERNEZ Coralie^1^, ROUX Damien^1,3^, RICARD Jean-Damien^1,4^

##### ^1^AP-HP, Hôpital Louis Mourier, DMU ESPRIT, Service de Médecine Intensive Réanimation, Colombes, France; ^2^Department of Epidemiology, Biostatistics and Clinical Research, AP-HP, Hôpital Bichat, Paris, France; ^3^Université Paris Cité, Institut Necker-Enfants Malades, INSERM U1151, CNRS UMR 8253, Paris, France; ^4^Université Paris Cité, UMR1137 IAME, INSERM, Paris, France

###### Correspondence: Thaïs WALTER (thais.walter10@gmail.com)

*Annals of Intensive Care* 2022, **12(1):**FC-253

**Rationale:** During the COVID-19 pandemic, many more patients were turned prone than before, resulting in a considerable increase in workload. Whether extending duration of prone position may be beneficial has received little attention. We report here benefits and detriments of a strategy of extended prone positioning duration for COVID-19 related acute respiratory distress syndrome (ARDS).

**Patients and methods/Materials and methods:** Retrospective, monocentric, study on intensive care unit patients with COVID-19 related ARDS who have been treated with at least one session of prone positioning session of duration greater or equal to 24 h. When prone positioning sessions were initiated, patients were kept prone for a period that covered two nights. They were put back in the supine position around 11 AM the morning following the second night. Demographic data, data regarding the incidence of pressure injury and ventilation parameters were collected retrospectively on medical and nurse files of charts.

**Results:** For the 81 patients included, the median duration of prone positioning sessions was 39 h (interquartile range (IQR) 34–42) and patients benefited from a median of 2 sessions (IQR 1–4). The cumulated incidence of grade ≥ II pressure injuries was 26% (95%CI 17–37) and 2.5% (95% CI 0.3–8.8) for stages III/IV pressure injuries. This increased duration was associated with additional increase in oxygenation after 16 h with the PaO2/FiO2 ratio increasing from 150 mmHg (IQR 121–196) at H + 16 to 162 mmHg (IQR 124–221) before being turned back to supine (p = 0.017) (Fig. 1). For 213 (94%) of prone positioning sessions, patients were turned over to supine position during daytime, i.e. between 9 AM and 6 PM. In comparison, had prone positioning sessions lasted strictly for 16 h, half of them (n = 117, 52%) would have required a turning back to supine between 6 PM and 9 AM, i.e. during night shifts.

**Discussion:** In an ancillary study of an international randomized trial evaluating prone positioning in ARDS, a cumulated incidence of 25% of patients presenting for the first time a pressure injury of stage ≥ II between day 1 and ICU discharge was reported (1). This is in line with our findings of a cumulated incidence of 26%.

**Conclusion:** Given the number of patients requiring prone position because of COVID-19-related ARDS, our results may have significant impact on both intensive care unit staffing and organizational issues, and could improve ventilatory parameters.

**Reference 1:** for the Proseva trial group, Girard R, Baboi L, Ayzac L, Richard J-C, Guérin C. The impact of patient positioning on pressure ulcers in patients with severe ARDS: results from a multicentre randomised controlled trial on prone positioning. Intensive Care.

**Compliance with ethics regulations:** Yes in clinical research.
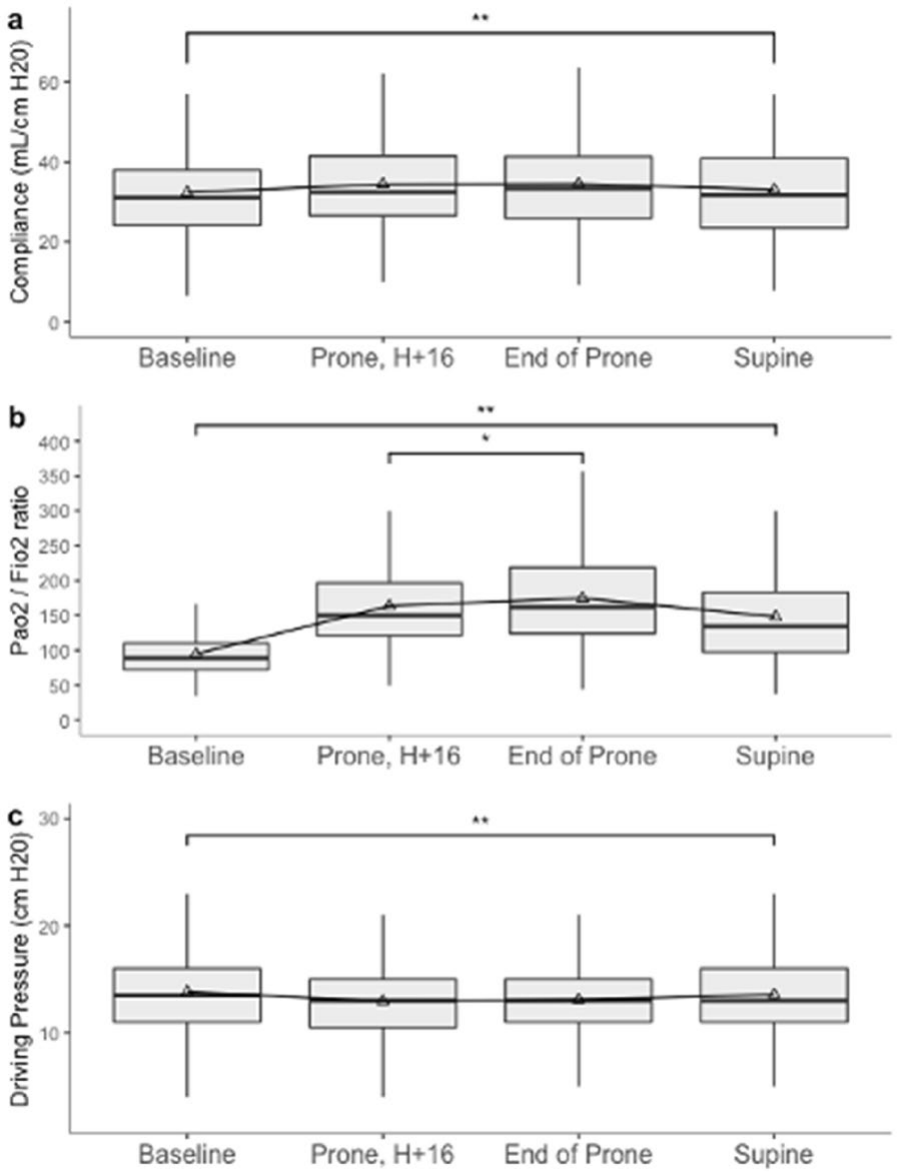


*Figure 1 Physiological parameter's changes during proning sessions.*

### FC-254 Impact of prone position on renal function in the critical covid19 patient

#### MOUNIR Anass^1^, NAANANI Karima^1^, CHABBAR Sara^1^, ELOUDGHIRI Ayman^1^, CHERKAB Rachid^1^, EL KETTANI ELHAMDI Chafik^1^, BARROU Lahoucine^1^

##### ^1^Réanimation Pavillon 17, CHU IBNROCHD, Casablanca, Maroc

###### Correspondence: Karima NAANANI (naananik@gmail.com)

*Annals of Intensive Care* 2022, **12(1):**FC-254

**Rationale:** Prone position [PP] is a method that has been widely used in the management of severe ARDS in COVID19. The increase in intra-abdominal pressure during the prone position can promote dysfunction of certain organs, particularly the kidney. The aim of our study was to assess the impact of prone position on renal function by analyzing the variations of intra-abdominal pressure.

**Patients and methods/Materials and methods:** A prospective study of 56 patients with critical COVID19 under mechanical ventilation over a period of 6 months [September 2020–March 2021]. PP was adopted in all our patients, with intravesical pressure [IVP] measurements before and during PP. In addition to the measurement of hourly diuresis, we performed the determination of renal aggression markers, urea and plasma creatinine levels before, during and after PP.

**Results:** The mean age was 61.8 years. A clear male predominance was observed. The prone position allowed an improvement of the patients` oxygenation after the first PP session and after 3 sessions. We detected an increase in IVP in the prone position, which exceeded 12 mmHg and rose even higher to 60 mmHg or 68 mmHg in some patients. Renal aggression was observed in 32 patients from the first session of PP. 26 patients progressed to AKI, which represents 46.4% of our study population, with recourse to extrarenal epuration in 12 out of 26 patients. Univariate analysis allowed us to show that obesity [OR: 1.24, Pvalue: 0.004], with a high abdominal perimeter [OR: 1.194, CI: 1.19–31.674, Pvalue: 0.009], and critical parenchymal damage > 75% [OR: 3.203, CI: 1.049–9.811, Pvalue: 0.038] represent risk factors for progression to renal insufficiency. It was also shown that high intravesical pressure is significantly associated with renal failure [OR: 6.4, CI: 1.446–44.64, Pvalue: 0.002].

**Discussion:** The kidney is the most sensitive organ to increased IAP and is the first organ affected in case of intra-abdominal pressure. However, to date, no study in the literature has concretely established predictive factors of the occurrence of acute renal failure in the patient placed in prone position, after a severe COVID19.

**Conclusion:** Our study allowed us to emphasize the value of prone positioning in the severe or critical Covid19 patient. However, the use of PP requires an understanding of the risks, including intra-abdominal pressure and the risk of renal failure.

**Compliance with ethics regulations:** Yes in clinical research.

### FC-255 Impact of Time-Controlled Adaptive Ventilation (TCAV) on hemodynamics in a swine ARDS model

#### PEQUIGNOT Benjamin^1^, LESCROART Mickael^1^, BITKER Laurent^2^, PINA Héloise^1^, TRAN N’Guyen^1^, HEBERT Jean-Louis^3^, RICHARD Jean-Christophe^2^, LEVY Bruno^1^, KOSZUTSKI Matthieu^1^

##### ^1^CHRU de Nancy, VandœUvre-Lès-Nancy, France; ^2^Hopital de la Croix Rousse, Lyon, France; ^3^Institut de Cardiologie, Groupe Hospitalier Pitié-Salpêtrière, Paris, France

###### Correspondence: Benjamin PEQUIGNOT (b.pequignot@chru-nancy.fr)

*Annals of Intensive Care* 2022, **12(1):**FC-255

**Rationale:** The current standard of care of severe ARDS is based on low tidal volume (VT), at 6 mL/kg of predicted body weight, and a positive end expiratory pressure (PEEP). An alternative strategy could be the time-controlled adaptive ventilation (TCAV) strategy, i.e. a specific settings of airway pressure release ventilation (APRV) mode. Initially reported by Habashi et al., TCAV relies on delivering a CPAP phase, followed by a brief expiratory release phase (1). TCAV leads to an increase in intra thoracic pressures and could be associated with hemodynamic impairment. The objective of our study was to compare hemodynamics between TCAV and conventional protective ventilation in a porcine ARDS model.

**Patients and methods/Materials and methods:** In ten pigs (63–73 kg) lung injury was induced by repeated saline lavages followed by 2 h of injurious ventilation. The animals were then randomized into two groups: 1) Volume-controlled ventilation (VCV) with a VT of 6 mL/kg and PEEP adjusted to a plateau pressure of 28 to 30 cmH2O; 2) TCAV group with P-high set between 27–29 cmH_2_O, P-low at 0 cmH_2_O, T-low adjusted to terminate at 75% of the expiratory flow peak, and T-high at 3–4 s for I:E > 6:1.

**Results:** There was no difference between-group for arterial blood pressure, pulmonary blood pressure or cardiac output. No significant left ventricular inotropic or relaxation alteration was noticed under TCAV versus VCV. Both elastance and PaO_2_:FiO_2_ were consistent with a moderate to severe ARDS after a 2-h period of injuring ventilation. Levels of total PEEP were significantly higher in TCAV group. Driving pressure and elastance were significantly lower in the TCAV group than in VCV group in multivariate analysis.

**Discussion:** First, experimental ARDS induced significant hemodynamic changes mainly driven by hypoxemia-induced pulmonary vasoconstriction. Secondly, TCAV did not significantly impact systemic hemodynamics, despite of the increase in intrathoracic pressures. Thirdly, TCAV increased alveolar recruitment in this ARDS model. Concerning limitations: the study might have been underpowered to assess a clinically relevant effect of TCAV on hemodynamics. Improvement in elastance can be in relation with higher level of total PEEP and suggest alveolar recruitment but we did not perform any CT scan to verify this hypothesis. To finish, conventional protective group had a transpulmonary pressure above 15 cmH_2_O at all times of interest which may also explained effect observed on hemodynamics parameters.

**Conclusion:** No safety signal was observed for hemodynamic adverse events in the TCAV group in this double hit ARDS swine model.

**Reference 1:** Habashi NM. Other approaches to open-lung ventilation: airway pressure release ventilation. Crit Care Med. 2005;33:S228–S240. https://doi.org/10.1097/01.CCM.0000155920.11893.37

**Compliance with ethics regulations:** Yes in animal testing.
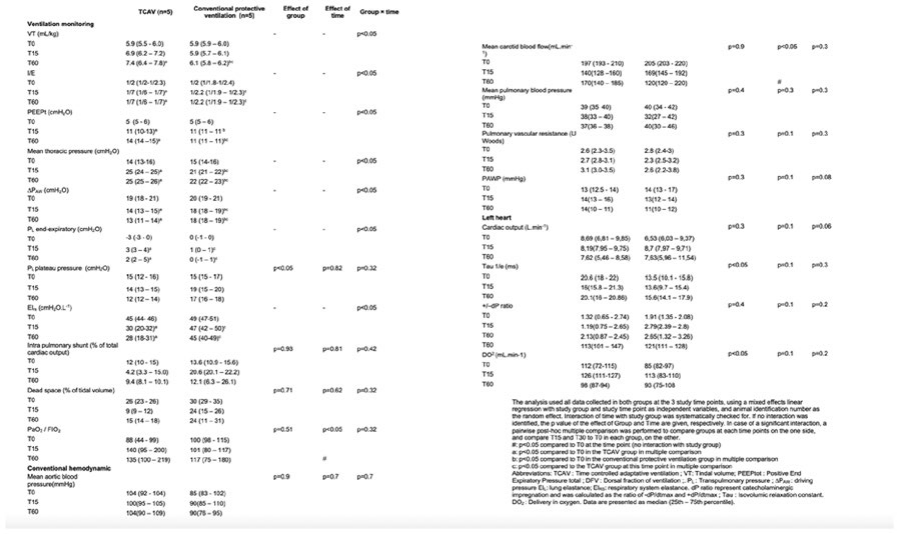


*Table of ventilatory and hemodynamics parameters.*

### FC-256 Potential for Alveolar recruitment in Covid-19 related ARDS: evaluation by the recruitment-to-inflation ratio (R/I) method

#### LAHMAR Manel^1^, BEN AHMED Hedia^1^, CHIHAOUI Abir^1^, BEDHIAF Amir^1^, HAMMOUDA Zeineb^1^, DACHRAOUI Fahmi^1^, ABROUG Fekri^1^, BESBES OUANNES Lamia^1^

##### ^1^CHU fattouma bourguiba Monastir, Monastir, Tunisie

###### Correspondence: Manel LAHMAR (firassmal4@gmail.com)

*Annals of Intensive Care* 2022, **12(1):**FC-256

**Rationale:** Optimizing alveolar recruitment by means of positive end-expiratory pressure (PEEP) is the cornerstone of ARDS ventilation. PEEP exposes nevertheless to alveolar distension, which is harmful in these patients. Hence, the actual effects of PEEP depend on the recruitment potential of the lung we should evaluate in every patient. Chen et al. recently validated in non-Covid-19 ARDS an index (R/I) allowing bedside assessment of the potential for alveolar recruitment (or overdistension).Objective: To characterize by R/I ratio the recruitment potential in an ARDS-Covid19 cohort.

**Patients and methods/Materials and methods:** a prospective study carried out between January and October 2021, including patients hospitalized consecutively for severe SDRA due to COVID-19 (PaO_2_/FiO_2_ ≤ 150 mmHg). All patients received a lung-protective ventilation. The R/I ratio was measured in the supine position and in the prone position. The threshold proposed by Chen et al. (0.5) was used to identify high recruiters and low recruiters.

**Results:** 23 patients were included. The median age was 62 years IQR [52–65], most patients were male (Sex ratio (H/F) = 1.87). The main chest computed tomography findings were ground-glass opacities only 13 (56.5%) or associated with consolidation 9 (39.1%). High Recruiters had isolated ground glass in 72.7% of cases. Whereas, low recruiters had more ground glass associated with condensations in 54.5%. All patients received a lung-protective ventilation. VT median was 380 ml [360–400], median PEEP and median Pplat were respectively 14 cmH_2_O [10–15], 30 cmH_2_O [29–30]. The median R/I index was 0.48 in supine and 0.65 in prone position. Eleven patients (47.8%) had recruitment potential (R/I ≥ 0.5) while 12 (52.2%) had non-recruiting lungs. Median plateau pressure was 30 (with extremes ranging from 25 to 32cmH2O) and static compliance was 27.

**Conclusion:** This study confirms the high frequency of ARDS with low recruitment potential in C-ARDS. This characteristic imposes a “personalized” ventilation based on the individual pulmonary mechanics properties.

**Compliance with ethics regulations:** Yes in clinical research.

### FC-257 Evaluation of alveolar recruitment in Covid-related ARDS: Multiple P–V curves vs Recruitment/Inflation (R/I) index

#### LAHMAR Manel^1^

##### ^1^CHU fattouma bourguiba Monastir, Monastir, Tunisie

###### Correspondence: Manel LAHMAR (firassmal4@gmail.com)

*Annals of Intensive Care* 2022, **12(1):**FC-257

**Rationale:** PV curves have allowed a better understanding of the pathophysiology of ARDS and remain a reference for evaluating the potential for alveolar recruitment. Chen and al recently validated an index (R/I) using the “Single breath maneuver” technique in ARDS. This index assesses the potential for alveolar recruitment (or inflation) at the patient's bedside. Both methods give an estimate of the magnitude of lung volume recruited. We conducted this study to compare estimated lung recruitment provided by these two methods.

**Patients and methods/Materials and methods:** Prospective study conducted in 26 patients with covid ARDS mechanically ventilated in prone position. Two PEEP levels were studied, and EELV was measured at both levels, with the difference (ΔEELV)) reflecting PEEP induced lung volume changes. Alveolar recruitment was measured using multiple pressure–volume (PV) curves (Extend respirator, Taema, France), and compared to the estimation made b the new index (R/I). High and Low recruiters were separated based on median estimated recruited lung volume (Rec_estim_). Estimated and measured recruitments were compared using Spearman correlation and Bland–Altman method.

**Results: T**he median age was 63 years IQR [52–66], with no difference between the groups. Median recruited lung volume (Rec mes) measured by the technique of P–V curves was 227 ml [IQR: 182–364]. By “Single breath maneuver” technique, the median volume recruited (Rec mes) was 410 ml [295–506]. The median index (R/I) in Low recruiters was 0.46 [0.33–0.71] vs 0.66 [0.57–0.89] in high recruiters. The ΔEELV and Recestim was higher in high recruiters than in low recruiters. Recruited volume measured by “Single breath maneuver” and the PV curves method were strongly correlated (biais = 79.95%CI: − 98.7; 211.8). The concordance of both methods is reported in the Fig. 1.

**Conclusion:** Bedside evaluation of lung recruitment can be performed by simple clinical tool such as single breath maneuver with acceptable bias.

**Compliance with ethics regulations:** Yes in clinical research.
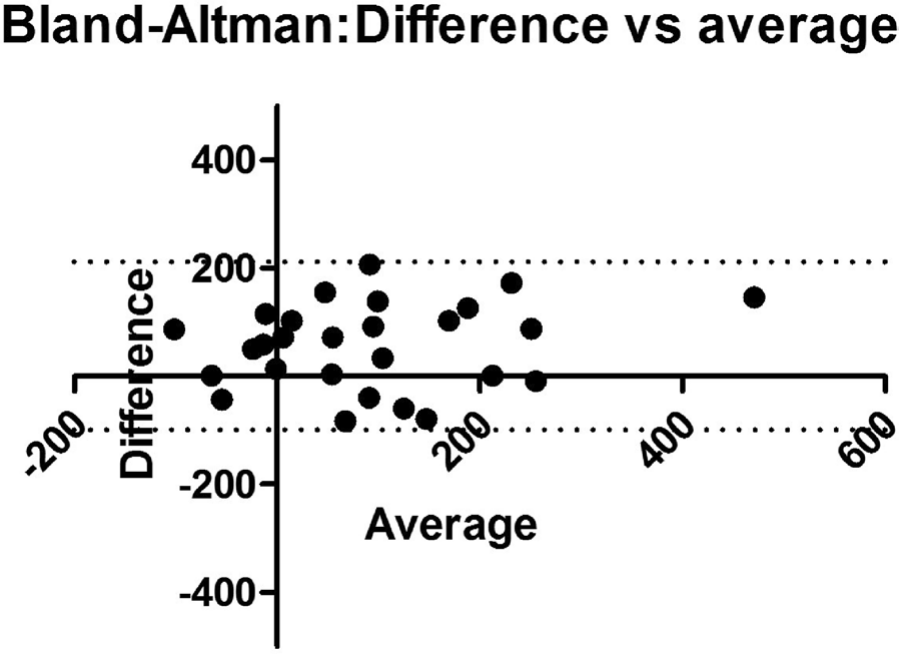


*Bland–Altman plot of Recruited volume measured by “Single breath maneuver” and the PV curves*

### FC-258 Prone position during veno-venous extracorporeal membrane oxygenation in COVID-19 ARDS

#### TEXTORIS Laura^1^, GRAGUEB-CHATTI Ines^1^, MICKAEL Bobot^1,2^, DAVIET Florence^1^, THEPENIER Mathieu^1^, PILARCZYK Estelle^1^, SALMI Saida^1^, PINGLIS Camille^1^, VALERA Sabine^1^, SANZ Celine^1^, AMALRIC Matthieu^1^, SYLVESTRE Aude^1^, PERES Noemie^1,3^, ARGENONE Fabien^1,4^, STEIN Claire^1^, BAILLEUL Clotilde^1^, FOREL Jean-Marie^1^, ROCH Antoine^1^, HRAIECH Sami^1^, PAPAZIAN Laurent^1^, GUERVILLY Christophe^1^

##### ^1^Médecine Intensive Réanimation, CHU Hôpital Nord, AP-HM, Marseille, France; ^2^Centre de Néphrologie et Transplantation Rénale, CHU de la Conception, AP-HM, Marseille, France; ^3^Réanimation, Hôpital Sainte Musse, Toulon, France; ^4^Réanimation, Centre Hospitalier de Digne-les-Bains, Digne-Les-Bains, France

###### Correspondence: Christophe GUERVILLY (christophe.guervilly@ap-hm.fr)

*Annals of Intensive Care* 2022, **12(1):**FC-258

**Rationale:** Veno-venous Extra-Corporeal Membrane Oxygenation (vv-ECMO) has been extensively used for severe Acute Respiratory Distress Syndrome (ARDS) related to COVID-19 in case of prone position (PP) failure. A recent meta-analysis on observational studies suggests to continue PP while on vv-ECMO both in classical ARDS and COVID-19 related ARDS (CARDS). PP could mitigate ventilator induced lung injuries even during low volume, low pressure ventilation facilitated by vv-ECMO. Objectives were to investigate respiratory mechanics and physiological effects of PP and to assess safety in severe CARDS patients during vv-ECMO.

**Patients and methods/Materials and methods:** We performed a prospective observational cohort study in COVID-19 patients supported by vv-ECMO in the ECMO center of a tertiary university hospital in Marseille. All patients were deeply sedated and received continuous infusion of neuromuscular blockers during PP. We compared respiratory mechanics, ECMO and ventilator settings, gas exchanges and hemodynamics in supine, start of PP, end of PP and back to supine with analysis of variance.

**Results:** We included 25 patients with a range of 2 to 17 PP totalizing 120 PP in the COVID-19 cohort. All patients had PP before ECMO consideration. ECMO was indicated after a median (IQR) of 5 (1–7) days of mechanical ventilation with PaO_2_: FiO_2_ of 68 (50–74) mmHg. ECMO weaning was achieved in 72%. ICU and hospital survival was 52%. First PP was performed after 2 (1–3) days of ECMO during 16 h. At baseline, very low tidal volume of 2.5 (2–3.5) ml/kg/PBW with a PEEP of 12 (10–15) cmH2O targeting a plateau pressure of 25 (21–26) cm H2O were used. We did not find significant changes in respiratory mechanics and gas exchanges during or after first PP. In particular, respiratory system compliance remains low at 11 (10–17) before and 11 (9–17) cm H2O after PP. PaO_2_ was respectively 75 (61–81), 78 (69–85), 77 (70–83) and 77 (67–89) mmHg at baseline supine, start of PP, end of PP and back to supine, (p = 0.33) at a constant ECMO blood flow (3.8 to 4 L/min) and membrane lung fraction of 1. Concerning safety, we did not observe severe adverse events during PP. Only 3 (2.5%) of PP were interrupted for respiratory or hemodynamics worsening.

**Conclusion:** PP is feasible and safe during vv-ECMO for severe CARDS. Potentials benefits could be explained by mechanism not identified in this study.

**Compliance with ethics regulations:** Yes in clinical research.

### FC-259 High-flow nasal oxygen therapy decrease the risk of mortality and the use of invasive mechanical ventilation in patients with severe SARS-CoV-2 pneumonia? A retrospective and comparative study of 265 cases

#### ALKOUH Rajae^1^, EL RHALETE Abdelilah^1^, MERBOUH Manal^1^, EL AIDOUNI Ghizlane^1^, BERRICHI Samia^1^, TAOUIHAR Salma^1^, AFTISS Fatima Zahra^1^, BKIYAR Houssam^1^, ABDA Naima^1^, HOUSNI Brahim^1^

##### ^1^CHU Mohammed VI OUJDA, Oujda, Maroc

###### Correspondence: Rajae ALKOUH (alkouhrajae1993@gmail.com)

*Annals of Intensive Care* 2022, **12(1):**FC-259

**Rationale:** To evaluate the efficacy of high-flow nasal oxygen (HFNO) versus non-invasive ventilation in COVID-19.

**Patients and methods/Materials and methods:** This is a retrospective and comparative study conducted over a period of 10 months from March 2020 to December 2020 and involving 600 patients hospitalized in the intensive care unit of the CHU Mohammed VI of Oujda for the management of acute respiratory failure caused by COVID-19.

**Results:** Out of 600 patients with acute respiratory failure, 265 patients were included in the analyses. 162 (61.10%) patients were treated with HFNO, the intubation rate was 49.7% (80 patients out of 162) of which 63 died intubated (78.8%). Concerning the 82 non-intubated patients, only 16 died (19.8%). The total number of patients who received NIV was 71 (26.8%), 33 (46.5%) required mechanical ventilation. In-hospital mortality in patients treated with NIV was 100%. The difference in mortality outcome between the two groups was significantly (P < 0.0001) reduced in HFNO.

**Conclusion:** Treatment with high-flow oxygen improved survival in patients with acute hypoxemic respiratory failure compared with noninvasive ventilation, although no difference was observed in intubation rate.

**Compliance with ethics regulations:** Yes in clinical research.

### FC-260 Predicting factors for the failure of high flow nasal cannula (HFNC) in COVID-19 related Acute Respiratory Distress Syndrome (ARDS)

#### BEDHIAFI Amir^1^, TOUMI Radhouane^1,2^, MATOUK Iyed^1^, BOUGUEZZI Nabil^1^, ZGHIDI Marwa^1,2^, MEDDEB Khaoula^1,2^, BEN SAIDA Imen^1,2^, BOUSSARSA Mohamed^1,2^

##### ^1^Medical Intensive Care Unit, Farhat Hached University Hospital, Sousse, Tunisie; ^2^Research Laboratory N° LR12SP09. Heart Failure. Farhat Hached University Hospital, University of Sousse, Sousse, Tunisie

###### Correspondence: Amir BEDHIAFI (bedhiafi.emir@gmail.com)

*Annals of Intensive Care* 2022, **12(1):**FC-260

**Rationale:** HFNC in widely used as a first line respiratory support in severe to critical COVID-19 pneumonia. We aimed to investigate factors associated with failure of HFNC in the management of COVID19 related ARDS (CARDS).

**Patients and methods/Materials and methods:** In this single-center study conducted in a 20-bed adult-ICU, consecutive admission for CARDS were screened for eligibility. Patients who underwent invasive mechanical ventilation (IMV) within 6 h upon admission were excluded. We collected clinical data and lab findings from admission until HFNC weaning or intubation which defined HFNC failure. ROX index was recorded at H0, H2, H6, H12 and H24. We also measured PF ratio evolution, monitored FiO_2_ and respiratory rate (RR). High work of breathing (HWOB) was defined as a respiratory rate (RR) above 30 cpm and/or excessive accessory muscle use.

**Results:** 230 patients were included until August 2021. 61.5% (n = 141) men, median aged 62 years [IQR 53–70] with SAPS-II at 28 ± 8, PF ratio 132 ± 63.8 under FiO_2_ 0.72 ± 0.23 on admission. 42% had hypertension, 41.3% diabetes and 32.2% obesity. 55.2% (n = 127) successfully weaned from HFNC with a survival rate at 18% among those ongoing IMV. HFNC failure was associated to: higher SAPS II (32 ± 7.4 vs 24.7 ± 7, p < 0.001) obesity (47% vs 27%, p = 0.001) and compromised oxygenation parameters on admission: high FiO_2_ (0.84 ± 0.17 vs 0.6 ± 0.21), RR (29.7 ± 4.6 vs 25.6 ± 3.7, p = 0.001), presence of HWOB (67% versus 17%, p < 0.001) and lower PF-ratio (101 ± 48.6 vs 157.5 ± 64.2, p < 0.001). Sub-group analysis following PF-ratio showed higher success rate in mild-to-moderate compared to severe ARDS (85.7%, 65.2% and 27.6% respectively, p = 0.001). ROX-index was significant at H6, H12 and H24 to predict HFNC failure. ROC analysis showed a higher specificity and sensitivity for H12 and H24 (AUC 0.906 IC 95% [0,864–0.948] and 0.944 IC 95% [0,908–0.981] respectively, p < 0.001) [Fig. 1]. Thirty percent (n = 69) required non-invasive positive pressure ventilation at day 2.1 ± 1.3 alternating with HFNC and had higher failure rate (88.4% vs 26.1%, p < 0.001). Eighty seven percent (n = 200) performed prone position while under HFNC with a higher associated success rate (58% vs 33%, p = 0.01).

**Conclusion:** HFNC appears to be safer in mild-to-moderate CARDS especially when combined to prone position. ROX index, PF-ratio and WOB monitoring are reliable predictors for HFNC failure. More studies might be needed to confirm these results.

**Compliance with ethics regulations:** Yes in clinical research.
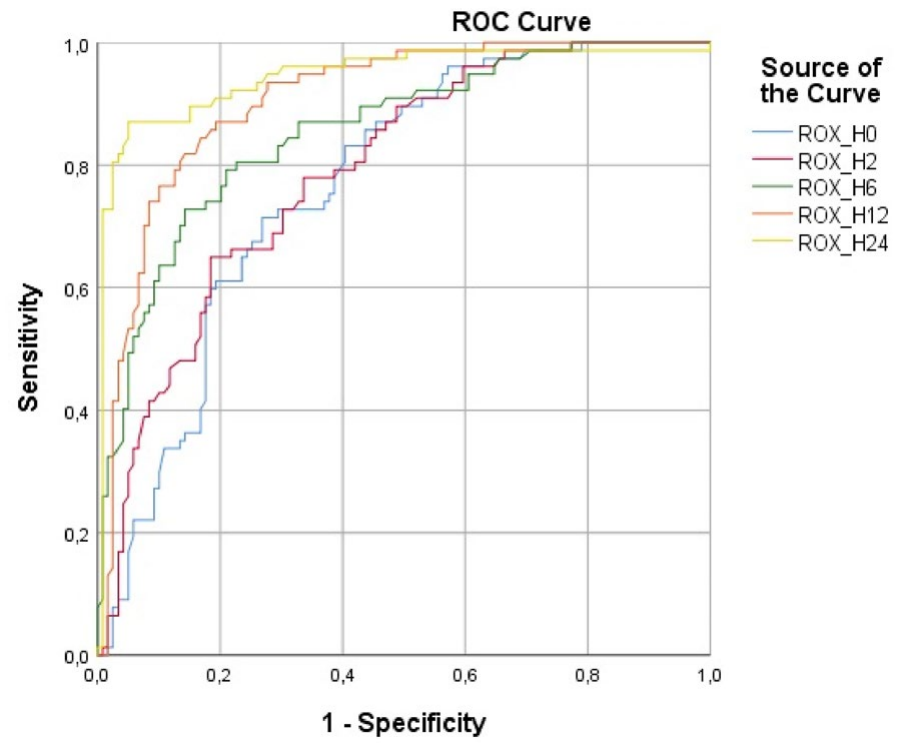


*ROC curves and AUC of ROC index measurement for prediction of HFNC failure*

### FC-261 Comparison of three noninvasive oxygenation strategies on outcomes in critically ill patients admitted for Covid-19 pneumonia: a retrospective multicenter cohort study

#### CHELLY Jonathan^1^, COUPRY Louis-Marie^2^, VONG Ly Van^3^, KAMEL Touffik^4^, MARZOUK Medhi^5^, TERZI Nicolas^6^, BRUEL Cedric^7^, AUTRET Aurélie^1^, GARNERO Aude^1^, ARNAL Jean-Michel^1^

##### ^1^Centre Hospitalier Intercommunal Toulon La Seyne sur Mer, Toulon, France; ^2^Groupe Hospitalier Sud Ile de France, Melun, France; ^3^Grand Hôpital de l'Est Francilien, Jossigny, France; ^4^Centre Hospitalier Régional d'Orléans, Orléans, France; ^5^Centre Hospitalier de Béthune, Béthune, France; ^6^Centre Hospitalier Universitaire de Grenoble, Grenoble, France; ^7^Hôpital Saint-Joseph, Paris, France.

###### Correspondence: Jonathan CHELLY (jonathan.chelly@ch-toulon.fr)

*Annals of Intensive Care* 2022, **12(1):**FC-261

**Rationale:** Noninvasive respiratory support (NIRS) is recommended as the first line to reduce the need of invasive mechanical ventilation (MV) and its associated complications and high mortality in patients affected by Covid-19 pneumonia admitted in the intensive care unit (ICU). However, the optimal first-line noninvasive strategy to support oxygenation and ventilation is still debated.

**Patients and methods/Materials and methods:** We conducted a retrospective multicenter study in 7 French ICUs, including all consecutive adults admitted between July and December 2020 with a documented SARS-CoV-2 acute respiratory failure (PaO_2_/FiO_2_ < 300 mmHg), and treated either with high-flow nasal therapy (HFNT) alone, or noninvasive ventilation (NIV) combined or not with HFNT or continuous positive airway pressure combined or not with HFNT (CPAP). Patients who received both NIV and CPAP and those who were intubated within the 12 first hours after ICU admission were excluded. The primary outcome was the NIRS failure, defined as the need of endotracheal intubation or death in the ICU without endotracheal intubation.

**Results:** Among the 355 included patients, 160 (45%) were treated with HFNT alone, 115 (32%) with NIV and 80 (22%) with CPAP. NIV and CPAP were combined with HFNT in 97 (84%), and 79 (99%) patients, respectively. Median age (67 [58–75] years), body mass index (29 [25–33] kg/m^2^), SAPS-2 (42 [32–57]) and PaO_2_/FiO_2_ (130 [93–167]) on admission were similar between groups whereas Charlson comorbidity index was lower when HFNT was used alone compared to NIV and CPAP. The primary outcome occurred in 158 (45%) patients with a statistical difference between HFNT alone, NIV, and CPAP groups (41%, 59%, and 31%, respectively; p < 0.001). Compared to patients who were treated with HFNT alone or NIV, those who were treated with CPAP had a lower incidence of the primary outcome on day 28 after ICU admission (p = 0.002) whereas ICU mortality was similar between groups (p = 0.20).

**Conclusion:** Among patients admitted for Covid-19 pneumonia in ICU and treated with NIRS, outcome seems to differ according to the strategy. More studies are warranted to compare such NIRS and identify the optimal treatment.

**Compliance with ethics regulations:** Yes in clinical research.

### FC-262 Using CPAP in patients with SARS-CoV-2 hypoxemic pneumonia outside intensive care unit

#### ESSAFI Fatma^1,2^, ESSAGHAYER Salma^1,2^, GLENZA Iheb^1,2^, TALIK Imen^1,2^, BEN ISMAIL Khaoula^1,2^, KADDOUR Moez^1,2^, BAYOUDH Aida^3^, FESSI Ilhem^3^, JERBI Tahani^4^, CHKIRBENE Mariem^4^, MERHABENE Takoua^1,2^

##### ^1^Intensive Care Unit, Regional Zaghouan’s Hospital, Zaghouan, Tunisie; ^2^Faculty of medicine of Tunis, Tunis El-Manar university, Tunis, Tunisie; ^3^Pneumology department, Regional Zaghouan’s Hospital, Zaghouan, Tunisie; ^4^Pharmacy department, Regional Zaghouan’s Hospital, Zaghouan, Tunisie

###### Correspondence: Fatma ESSAFI (fatma.essafi@fmt.utm.tn)

*Annals of Intensive Care* 2022, **12(1):**FC-262

**Rationale:** Use of continuous positive airway pressure (CPAP) has been recommended in the management of patients with acute respiratory failure related to COVID-19 infection. However, its impact on mortality, length of hospital stay and the occurrence of complications have not been elucidated especially when delivered outside of a traditional critical care environment. Through this study, we propose to investigate the role of CPAP in the management of hypoxemic pneumonia due to SARS-CoV-2 in a pneumology department.

**Patients and methods/Materials and methods:** We reviewed retrospectively all data of consecutive confirmed COVID-19 patients receiving CPAP for the treatment of respiratory failure secondary to COVID-19. Patients admitted from 1st June to 31th August 2021 on the pneumology department of the regional hospital of Zaghouan, Tunisia were enrolled. Epidemiological, clinical and paraclinical data, complications and outcome were collected.

**Results:** During the study period, 205 patients were hospitalized, 65 of them required CPAP (31.7%). Median age was 64 years [34–87]. A clear male predominance was noted with sex ratio1.5. Chronic heart disease, obesity, Diabetes mellitus, and chronic obstructive pulmonary disease were the most common comorbidities (55.4%; 38.5%, 27.7% and 10% respectively). Tobacco consumption was noted in 20% of patients. On admission, all patients had acute respiratory failure. Initial symptoms are essentially fever, nonproductive cough, dyspnea and gastrointestinal symptoms. The median time of symptoms at admission was 8.2 ± 3.6 days [1–20]. On initial examination, pulse air saturation ranged was 83.5 ± 8% [57–93] and oxygen requirements were 12 ± 6 L/min [6–30]. Four patients (6.2%) presented with acute hypertensive heart failure, requiring diuretic therapy. The predominant pattern of abnormality observed in chest CT was ground-glass opacification in 55 patients (84.6%), bilateral opacities in 27 patients (41.5%) and crazy paving in 17 patients (26.2%). Mean lung extension was estimated at 58 ± 20%. Mean duration of CPAP ventilation was 4 ± 3.2 days [1–18]. It was associated to high flow oxygen therapy in 43 patients and to conventional oxygen therapy in 22 patients. Awake prone position was applied in 39% of patients. Only one patient had developed care-related pneumonia. Mean length of hospital stay was 7.2 ± 4.8 days [1–21]. In hospital mortality was 32%. The outcome was favorable in 58 patients (66%).

**Conclusion:** CPAP represents a feasible and potentially efficient option for the treatment of respiratory failure in COVID-19. It can be delivered safely outside the traditional critical care, thus relieving pressure on critical care beds.

**Compliance with ethics regulations:** Yes in clinical research.

### FC-263 Predictive factors of high flow nasal cannula failure in critically ill COVID-19 patients admitted in a medical ICU for acute respiratory failure

#### ZORGATI Hend^1^, BEN BRAIEK Dhouha^1^, HIDRI Rania^1^, SAADAOUI Oussama^1^, BEN MANSOUR Safa^1^, MIGHRI Imen^1^, DOUZI Arsalene^1^, KAABI Chaima^1^, GHABI Oumaima^1^, AZAZA Asma^1^, BEN JAZIA Rahma^2^, KACEM Amani^2^, KHARRAT Imen^2^, AYACHI Jihene^1^

##### ^1^Service Réanimation Médicale, Hôpital Universitaire Ibn El Jazzar, Kairouan, Tunisie; ^2^Service Pneumologie, Hôpital Universitaire Ibn El Jazzar, Kairouan, Tunisie

###### Correspondence: Jihene AYACHI (ayachijihen@gmail.com)

*Annals of Intensive Care* 2022, **12(1):**FC-263

**Rationale:** High-Flow Nasal Cannula (HFNC) therapy appears to be effective to treat hypoxemic acute respiratory failure (ARF) patients during the outbreak of Coronavirus disease (COVID-19). However, there are few data about factors associated with HFNC failure in COVID-19 patients. Aim: To determine predictive factors associated with HNFC failure in COVID-19 patients admitted in medical intensive care unit (ICU) for ARF.

**Patients and methods/Materials and methods:** A 1 year observational, retrospective study performed from January 1st, 2021 to December 31st, 2021 in a 9-bed medical ICU at university hospital including adult patients admitted for hypoxemic ARF pneumonia secondary to SARS-CoV-2 and received HFNC on admission. Medical patients’ charts were reviewed to collect demographic characteristics, underling diseases, severity at admission, clinical course and outcomes. Univariate and multivariate analysis were used to identify predictive factors associated with HFNC failure. HFNC failure was defined as mechanical ventilation (invasive or noninvasive) use.

**Results:** From 146 patients admitted for severe COVID-19 pneumonia, 128 (87.7%) received HFNC. Main characteristics were mean age, 51.05 ± 14.8 years; male predominance, 75 (58.6%) and 100 (78.1%) had at least one comorbidity. Median BMI and P/F ratio were: 31 [28–32]kg/m^2^ and 92.75 [69–118.5]. Median SAPS II, SOFA and Charlson score were respectively: 23 [16–29]; 2 [2–4] and 1 [0–2]. HFNC parameters were median Flow at 52.5 [45–60]l/min and median FiO2 at 90 [70–100] %. The median ROX index at H1 and H24 were respectively at 4.26 [3.3–5.9] and 6.2 [4.5–8.4]. Awake prone position was performed in 105 (82%) of cases. HFNC failed in 43 (33.6%) patients, among them 32 (25%) were initially managed with noninvasive ventilation (NIV) with mortality rate at 35.2%. Univariate analysis identified the following factors for HNFC success and failure: elderly patient (7(8.2%) vs 17(39.5%), p = 0.000); cardiovascular comorbidity (7(8.2%) vs 9(20.9%), p = 0.04); Charlson index (0.5 [0–1] vs 2 [1–3], p = 0.000; SAPSII (19 [13–24] vs 29 [24–34.25], p = 0.000); SOFA (2 [1–4] vs 4 [2–8.5], p = 0.000); prone position (71(83.5%) vs 29(67.4%), p = 0.038); ROX index at H1(5 [3.8–6.04] vs 3.45 [3–4.57], p = 0.000); ROX index at H24 (7.73 [5.75–9.05] vs 4.2 [3.8–5.6], p = 0.000) and ROX index at day 6 (9.6 ± 3.5 vs 4.5 ± 1.4, p = 0.000). The multivariate logistic regression model identified ROX index at day 6 (OR, 0.3; 95% CI, [0.14–0.67]; p = 0.03) as a predictor factor of HFNC success.

**Conclusion:** ROX index at day 6 was the best predictor of HFNC failure in COVID-19 patients with hypoxemic AFR admitted in ICU.

**Compliance with ethics regulations:** Yes in clinical research.

### FC-264 Non-invasive ventilation in critically ill COVID-19 patients

#### ALILA Ilef^1^, KHARRAT Sana^1^, HAFDHI Malek^1^, BACCOUCH Najeh^1^, JERBI Salma^1^, BAHLOUL Mabrouk^1^, BOUAZIZ Mounir^1^

##### ^1^hopital Habib Bourguiba Sfax, Sfax, Tunisie

###### Correspondence: Ilef ALILA (ilefalila1323@gmail.com)

*Annals of Intensive Care* 2022, **12(1):**FC-264

**Rationale:** Non-invasive ventilation (NIV) was recommended for acute respiratory distress syndrome. A potential role for NIV to delay or avoid invasive mechanical ventilation (IMV) has emerged during the COVID-19 pandemic. The aim of this study was to explore the role of non-invasive ventilation in prediction of outcome in critically ill COVID-19 patients.

**Patients and methods/Materials and methods:** We conducted a retrospective study in a medical ICU over a period of 16 months [September 2020–December 2021] including patients with SARS-COV2 infection. The impact of non-invasive ventilation on complications and patient outcome was investigated.

**Results:** During the study period, 586 patients were included with a mean age of 59.5 ± 14.7 years and a gender ratio of 1.6. The median SAPSII and SOFA score were respectively of 29 ± 14.8 and 4 ± 2.6. Most patients had comorbidities, including hypertension (36%), obesity (32.1%) and diabetes (36.2%). 419 (71.5%) patients had severe acute respiratory distress syndrome (ARDS). Median ICU length of stay was 6 (IQR [3–10]) days and overall mortality was of 49% (287 patients). 378 patients (64.5%) needed NIV oxygenation. Of those who received NIV, 214 (57%) required IMV versus 50 (24%) of those not. Patients who received NIV developed more infections during their hospitalizations than those in the No-NIV group (220 (58%) vs 44 (21%); p < 0.001). Mortality was higher in patients receiving NIV (238 (63%) vs 49 (23%), P < 0.001). Length of stay was significantly longer in NIV group (Table I).

**Conclusion:** The use of NIV can predict morbidity and mortality in critically ill COVID-19 patients.

**Compliance with ethics regulations:** Yes in clinical research.
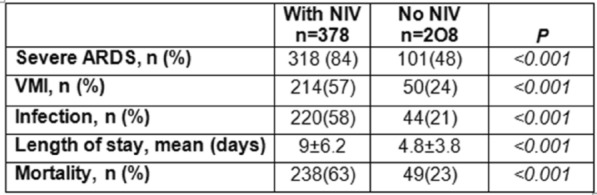


*Table 1: Outcome comparison according to non-invasive ventilation.*

### FC-265 Comparison of two respiratory support strategies in critically ill patients admitted for Covid-19: a retrospective monocentric cohort study

#### PAUL Timothé^1^, CHELLY Jonathan^1^, ARNAL Jean-Michel^1^, GARNERO Aude^1^

##### ^1^Centre Hospitalier Intercommunal Toulon La Seyne sur Mer - Hôpital Sainte Musse, Toulon, France., Toulon, France

###### Correspondence: Timothé PAUL (paultimothe2@gmail.com)

*Annals of Intensive Care* 2022, **12(1):**FC-265

**Rationale:** Respiratory support for patients suffering for COVID-19 pneumonia in intensive care unit (ICU) is mandatory. During the first months of the outbreak, clinical trials have demonstrated that many oxygen strategies could delay intubation. However the outcomes between early and late intubation were not established in this particular kind of pneumonia.

**Patients and methods/Materials and methods:** We conducted a retrospective observational monocentric study in a French ICU including all consecutive adults with a documented SARS-CoV-2 acute respiratory failure (PaO_2_/FiO_2_ < 300 mmHg). Two periods were compared: the first included all patient admitted between February 2020 and June 2020, and an early intubation strategy was applied; the second period included all patients admitted between August 2020 and December 2020, and a non-invasive respiratory support strategy, using High-flow nasal canula, continuous positive airway pressure (CPAP), awake prone positioning and late intubation was applied. The primary outcome was the ICU length of stay. The secondary outcomes were complications, rate and duration of invasive ventilation and mortality in ICU.

**Results:** 47 patients were included in the first period and 96 in the second period. First period was associated with a longer ICU stay compared to the second period (11.00 [8.25–19.50] days versus 8.00 [4.75–14.25] days; p = 0,028). First period was associated with longer duration of respiratory support and higher intubation rate compared to the second (9 [9–16] days versus 7 [3–14] days; p = 0.338; 68% versus 25%; p < 0,001, respectively). The first period was associated with more complications such as hypotension, tracheotomy, critical illness neuromyopathy, ventilator associated pneumonia and thromboembolic complications whereas barotrauma and venovenous extracorporeal membrane oxygenation were no different between periods. ICU mortality was similar between periods (p = 0.814).

**Conclusion:** In this study, non-invasive respiratory support strategy with late intubation is associated with a shorter length of stay for critically ill patient with COVID-19 compared to an early intubation strategy. A non-invasive strategy would allow a better resource management of the healthcare system during the outbreak. Other studies are warranted to evaluate the impact on the long-term mortality.

**Compliance with ethics regulations:** Yes in clinical research.

### FC-266 The effect on proning depends on the interface used: NRB vs HFNC vs NVI

#### SALLEMI Ilyes^1,2^, RACHDI Emna^1,2^, JARRYA Fatma^1,2^, JAMOUSSI Amira^1,2^, AYED Samia^1,2^, BESBES Mohamed^1,2^, BEN KHELIL Jalila^1,2^

##### ^1^Hopital Abderrahman Mami, Ariana, Tunisie; ^2^Faculté de Médecine de Tunis, Tunis, 0

###### Correspondence: Emna RACHDI (e.rachdi@yahoo.fr)

*Annals of Intensive Care* 2022, **12(1):**FC-266

**Rationale:** Since the PROSEVA study in 2013, prone positioning (PP) is a postural maneuver recommended early in the management of acute respiratory distress syndrome (ARDS) in patients who remain hypoxic on mechanical ventilation. With the increase in the incidence of ARDS over the last 2 years in relation to the COVID-19 pandemic, PP has been attempted in non-intubated patients to improve oxygenation. Several studies have been carried out in non-intubated patients admitted to an intensive care unit for the management of ARDS. We proposed to compare the effect of different oxygenation interfaces during PP wakefulness.

**Patients and methods/Materials and methods:** Between March and May 2021, 117 adult patients were admitted to the medical intensive care unit of MAMI hospital for severe to critical COVID-19. Among them, 56 patients underwent at least one PP session. During the first 24 h, PP sessions of at least 30 min were performed with change of oxygenation interface each time ranging from a non-rebreather mask (NRB) to a high flow nasal canula (HFNC) then to non-invasive ventilation (NIV) last. Blood gas was performed before and 20 min after each session under the same oxygenation parameters. The response to PP was defined by an increase in the PaO_2_/FiO_2_ ratio of 20%.

**Results:** Fifty-six patients had a PP session. They were male in 56.9% of cases with a mean age of 55 years and mean BMI of 28.06 kg/m^2^. At admission, they had severe ARDS in 46.4% and mild in 42.9%. PP session was performed in conventional oxygen therapy in fifty-one patients. The difference in PaO_2_/FiO_2_ ratio before and after the session was on average 14 mmHg [− 96, 138 mmHg] with no statistically significant difference (p = 0.09). The response to PP was heterogeneous, with 25.5% (n = 13) responders. Under HFNC, the mean delta PaO_2_/FiO_2_ was 5.6 mmHg (− 67, 125 mmHg) for all 32 patients, 21 were responders (65%). PP under NIV was tolerated by only 41 patients who had a mean PaO_2_/FiO_2_ response of 17.6 mmHg (− 120, 197 mmHg) with no statistically significant difference (p = 0.07). Eighteen of them (43.9%) were responders. In contrast to NIV, the PP response under NRB decreased the need for oro-tracheal intubation (p = 0.03). PP significantly improved survival of patients under NRB and HFNC (p = 0.04 for NRB and p = 0.03 for HFNC).

**Conclusion:** Although the benefice of PP in intubated patient is demonstrated, awake PP needs more evidence by conducting more prospective multicentric studies with prolonged proning sessions.

**Compliance with ethics regulations:** Yes in clinical research.

### FC-267 Factors associated with mortality in hospitalized Covid-19 patients

#### ALLOUCHE Hend^1^, GUISSOUMA Jihene^1^, TRABELSI Insaf^1^, BACHA Ichrak^1^, SAMET Mohamed^1^, BRAHMI Habib^1^, GHADHOUNE Hatem^1^

##### ^1^Hôpital universitaire Habib Bougatfa de Bizerte, Bizerte, Tunisie

###### Correspondence: Hend ALLOUCHE (hend.allouche@fmt.utm.tn)

*Annals of Intensive Care* 2022, **12(1):**FC-267

**Rationale:** The emergence of the novel Covid-19 has caused millions of deaths worldwide. This study aimed to identify factors that are associated with mortality in hospitalized Covid-19 patients.

**Patients and methods/Materials and methods:** This was a retrospective, observational cohort study conducted on adult patients hospitalized in intensive care unit with confirmed Covid-19 infection, between January 2021 and September 2021. Demographics, clinical pattern, laboratory and radiological investigations associated with increased rate of mortality were analyzed.

**Results:** Of the 140 patients included, mortality rate was 70.3%. Main factors associated with mortality among our cohort were age over 60 years (p < 0.001), history of hypertension (p = 0.001) and chronic kidney failure (p = 0.04). Also, elevated inflammatory markers and severity scores were associated with higher mortality. Patients placed on mechanical ventilation and those who had nosocomial infections had an increased rate of mortality (p < 0.001 and p = 0.01 respectively). Greater lung involvement and obesity were not associated with higher mortality in our study.

**Conclusion:** In our study several factors were associated with mortality as advanced age, hypertension, chronic kidney failure, elevated inflammatory markers and severity scores, mechanical ventilation and nosocomial infections.

**Compliance with ethics regulations:** Yes in clinical research.

### FC-268 Is procalcitonin useful to diagnose early bacterial pulmonary co-infection among intubated patients with SARS-CoV-2 or influenza pneumonia?

#### SAURA Ouriel^1^, ROUZÉ Anahita^1^, MARTIN-LOECHES Ignacio^2^, POVOA Pedro^3^, DEMOSTHENES Makris^4^, ARTIGAS Antonio^5^, LABREUCHE Julien^1^, NSEIR Saad^1^

##### ^1^CHU Lille, Lille, France; ^2^Multidisciplinary Intensive Care Research Organization (MICRO), St James Hospital, Dublin, Irlande; ^3^Hospital de Sao Francisco Xavier, Lisbone, Portugal; ^4^University Hospital of Larissa, Larissa, Grece; ^5^Corporacion Sanitaria Universitaria Parc Tauli, CIBER Enferme dades Respiratorias, Sabadell, Espagne

###### Correspondence: Ouriel SAURA (ouriel.saura@gmail.com)

*Annals of Intensive Care* 2022, **12(1):**FC-268

**Rationale:** A large proportion of critically ill patients admitted for severe coronavirus disease 2019 (COVID-19) receive empirical antimicrobial treatment upon admission. Recent studies have reported a low rate (< 10%) of early bacterial co-infection among patients with severe acute respiratory syndrome coronavirus 2 (SARS-CoV-2) pneumonia, and significantly lower compared to patients with influenza pneumonia (1). We aimed to determine the performance of procalcitonin (PCT) to diagnose early bacterial pulmonary co-infection within 48 h after intubation in critically ill patients with SARS-CoV-2 or influenza pneumonia.

**Patients and methods/Materials and methods:** We performed a post hoc analysis of data from the multicenter retrospective coVAPid cohort study. Adult patients receiving invasive mechanical ventilation for at least 48 h for SARS-CoV-2 (first wave in Europe) or influenza pneumonia (2016–2020 admissions), and with at least one PCT measurement within 48 h after intubation were considered for the present analysis. Bacterial pulmonary co-infection was defined by bacterial isolation, within 48 h after intubation, in endotracheal aspirates, bronchoalveolar lavage, or blood cultures, or a positive pneumococcal or legionella urinary antigen test. We assessed the performance of PCT to diagnose co-infection using a receiver operating characteristic (ROC) curve analysis, and the association of PCT with patient’s co-infection status and 28-day mortality after pre-specified adjustment (age, sex, IGS2, and disease group) using a multivariable logistic regression model.

**Results:** 615 patients were included (349 in SARS-CoV-2, and 266 in influenza groups). The area under the ROC curve (AUC) was 0.60 (95%CI 0.48 to 0.71) and 0.66 (95%CI 0.58 to 0.73) for the diagnosis of co-infection in SARS-CoV-2 and influenza groups, respectively. PCT was significantly associated with co-infection in patients with influenza pneumonia (adjusted odds ratio (OR) per log increase in level of PCT 1.29 (95%CI 1.13 to 1.48)), but not in patients with SARS-CoV-2 pneumonia (adjusted OR 1.26 (95%CI 0.98 to 1.61)) (Fig. 1). Conversely, adjusted OR for 28-day mortality per log increase of PCT was 1.03 (95%CI 0.89 to 1.19) and 1.41 (95%CI 1.16 to 1.71) in influenza and SARS-CoV-2 groups respectively, suggesting that PCT was a better indicator of disease severity than co-infection among severe COVID-19 patients.

**Conclusion:** We reported a low diagnostic value of PCT to detect bacterial co-infection within 48 h after intubation in patients with severe SARS-CoV-2 or influenza pneumonia. PCT level was significantly associated with 28-day mortality among severe COVID-19 patients.

**Reference 1:** Rouze A, Martin-Loeches I, Povoa P et al. coVAPid study group. Early bacterial identification among intubated patients with COVID-19 or influenza pneumonia: a European multicenter comparative cohort study. Am J Respir Crit Care Med. 2021 May 26;204(5):546.

**Compliance with ethics regulations:** Yes in clinical research.
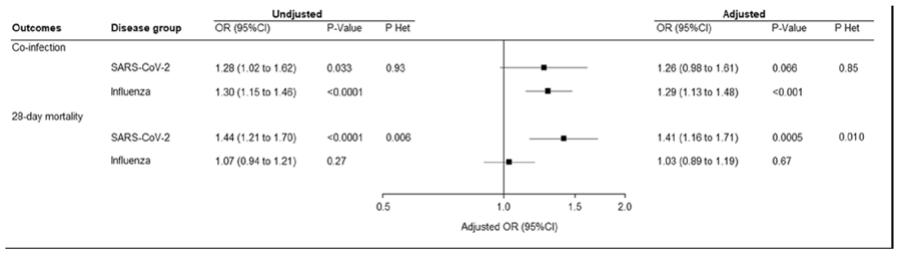


*Figure 1 Unadjusted and Pre-specified Adjusted Odds Ratio of Co-infection and 28-day mortality per log increase in highest level of procalcitonin*

### FC-269 Retrospective comparison of COVID-19 and seasonal influenza mortality and outcomes in the intensive care units of a French university hospital

#### DE MARIGNAN Donatien^1^, VACHERON Charles-Hervé^1^, ADER Florence^2^, LECOCQ Maxime^1^, RICHARD Jean Christophe^2^, ARGAUD Laurent^3^, BOHE Julien^1^, PIRIOU Vincent^1^, ALLAOUCHICHE Bernard^1^, FRIGGERI Arnaud^1^, WALLET Florent^1^

##### ^1^CHU LYON SUD - PIERRE BENITE (RHONE), Pierre Benite, France; ^2^CHU de la CROIX ROUSSE - LYON, Lyon, France; ^3^CHU EDOUARD HERRIOT - LYON, Lyon, France

###### Correspondence: Donatien DE MARIGNAN (donatien.de-seissan-de-marignan@chu-lyon.fr)

*Annals of Intensive Care* 2022, **12(1):**FC-269

**Rationale:** Severe COVID-19 has become a major worldwide health concern since its appearance at the end of 2019 in China. Commonalities have been suggested with seasonal influenza. Our objective was to evaluate the intrinsic mortality and burden of SARS-CoV-2 and seasonal influenza pneumonia in intensive care units (ICU) in a university hospital in a major city in France.

**Patients and methods/Materials and methods:** We conducted a retrospective study in six ICUs in a single institution. Consecutive patients admitted to an ICU with SARS-CoV-2 pneumonia from February 27 to April 4, 2020 (COVID-19 group) and seasonal influenza pneumonia from November 1, 2015 to April 30, 2019 (INFLUENZA group) were screened. A total of 350 patients were included in the COVID-19 group (18 refused to consent) and 325 in the INFLUENZA group (1 refused to consent). Diagnosis was confirmed by RT-PCR. Follow-up was completed on April 1, 2021. We collected demographic characteristics, comorbidities, long-term medications at ICU admission, and evaluated difference in 90-day adjusted-mortality between the COVID-19 and INFLUENZA group using a multivariable Cox proportional hazards model.

**Results:** COVID-19 patients were younger, mostly men, and had a higher median BMI, and comorbidities, including immunosuppressive condition or respiratory history were less frequent. In univariate analysis, no significant differences were observed between the 2 groups regarding in-ICU mortality, 30, 60 and 90-day mortality. After Cox modelling adjusted on age, sex, BMI, cancer, sepsis-related organ failure assessment (SOFA) score, simplified acute physiology (SAPS II) score, chronic obstructive pulmonary disease and myocardial infarction, the probability of death associated with COVID-19 was significantly higher in comparison to seasonal influenza (HR 1.57, CI95% [1.14; 2.17]; p = 0.006). The clinical course and morbidity profile of both groups was markedly different; COVID-19 patients had less severe illness at admission (SAPS II score, 37 [28–48] vs. 48 [39–61], p < 0.001, and SOFA score, 4 [2–8] vs. 8 [5–11], p < 0.001), but the disease was more severe considering ICU length of stay, duration of mechanical ventilation, PEEP level, and prone positioning requirement.

**Conclusion:** After ICU admission, COVID-19 was associated with an increased risk of death compared to seasonal influenza. Patient characteristics, clinical course, and morbidity profile of these diseases is markedly different. Patients with seasonal influenza die because of their comorbidities while COVID-19 patients die because of COVID-19 pneumonia.

**Compliance with ethics regulations:** Yes in clinical research.

### FC-270 Covid-19 and pregnancy: what we should know?

#### SDIRI Ines^1^, FITOUHI Nizar^1^, DRIDI Amira^1^, DORGHAM Sana^1^, OUERGHI Sonia^1^, MESTIRI Tahar^1^

##### ^1^hopital ariana abderahmen memi, Sfax, Tunisie

###### Correspondence: Ines SDIRI (sdiri.ynes@gmail.com)

*Annals of Intensive Care* 2022, **12(1):**FC-270

**Rationale:** Physiological changes during pregnancy have a significant impact on the immune system, respiratory system, cardiovascular function, and coagulation. The impact of SARS-CoV-2 in pregnancy remains to be determined. Our study aim to describe the characteristics of COVID19 infections in per partum patients before vaccine authorization.

**Patients and methods/Materials and methods:** A retrospective and descriptive study of 9 months duration carried out in the COVID intensive care unit including all per partum patients with COVID 19 pneumonia between March 2021 and September, 2021. All pregnant or immediate postpartum women hospitalized for SARS-CoV2 infection were included. Demographic, clinical, biologic, radiographic, therapeutic and outcomes data were collected. The primary endpoint was maternal mortality. Two groups were identified: Group 1 = Survivors and Group 2 = Deceased. The differences in data of the patients were evaluated.

**Results:** During the study period, we collected 17 patients. The incidence of COVID19 infection in per partum patients was 5% in our series. All patients were admitted postpartum (75% by caesarean section). The median age was 31(25–38) years. The comorbidities were chronic respiratory deficient (3 patients) and hypothyroidism (3). The mean BMI was 28 ± 3 kg/m^2^. All patients were unvaccinated. Seven were multiparous. The mean gestational age was 27 ± 6 weeks of amenorrhea. The mean PaO2/FiO2 ratio at admission was 98 ± 35 mmHg. All patients admitted to the ICU were in respiratory distress. Severe ARDS was observed in 11 patients (64%). 47% patients received high-flow nasal oxygen therapy and one patient was under mechanical ventilation at admission. Chest CT scans were performed in 15 patients (88.2%). An involvement highest of 50% was observed in 13 patients (76.4%). All patients received anticoagulation and corticosteroid therapy. Invasive mechanical ventilation (IMV) was required in 5 cases. 5 patients (29.4%) needed the use of vasoactive amines. The occurrence of nosocomial infection was found in 7 patients, 2 patients were developed acute kidney injury and one required hemodialysis, 6 patients presented a thromboembolic event. Tocilizumab was prescribed for 2 patients and one patient was discharged for ECMO. The median length of hospitalization was 9 ± 7 days and the mortality rate was 46.3%. Univariate analysis of the 2 groups showed that use of IMV (G1: 5 (100%) vs. G2: 0 (0%); p = 0.001) and nosocomial infection (G1: 4 (80%) vs. G2: 0 (0%); p = 0.01) were associated with excess mortality.

**Conclusion:** Currently data didn't show significant relationship between COVID-19 severity and pregnancy and there is no strong evidence that covid-19 lead to adverse pregnancy outcome, but further studies are needed.

**Compliance with ethics regulations:** Yes in clinical research.

### FC-271 Pregnant women and COVID 19 in intensive care: series of 18 cases

#### BOUSBIA Soulef^1^, DJEBBARI Abdelkader^1^, KRABA Rachid^1^, BENHAMED Mohamed Amine^1^

##### ^1^CHU D'ORAN, Oran, Algerie

###### Correspondence: Soulef BOUSBIA (bsoulef90@gmail.com)

*Annals of Intensive Care* 2022, **12(1):**FC-271

**Rationale:** In pregnant women, the symptoms of COVID-19 are similar to those of the general population in most cases. That said, like any population at risk, more serious symptoms may appear such as pneumonia or ARDS. The objective is to define the clinical, biological and therapeutic characteristics as well as the management of a series of pregnant women with COVID-19.

**Patients and methods/Materials and methods:** This is a retrospective and descriptive study which collected 18 pregnant patients affected by the Coronavirus SARS-CoV-2 in the intensive care unit between July 20 and September 15, 2021 We included parturient requiring a high flow of polypneic oxygen at 30 cycles/min with an SPO_2_ < 92% and the parturient without sign of respiratory distress were excluded.

**Results:** The average age is 31 years old. Six patients were followed for a chronic disease. Gestational diabetes was the most common comorbidity. Contact with a suspected or confirmed case of COVID-19 was identified in 10 patients. The average incubation period for the virus was 6.89 days. The average term of pregnancy was 33.36 weeks of amenorrhea. The preferred way of delivery was caesarean section. The most frequently reported symptoms were fever in all patients, dyspnea in all patients. 16 patients were taken care of in medical intensive care for an array of acute respiratory distress syndrome (ARDS) after cesarean section. Four patients were put on nasal high-flow oxygen therapywith recourse to mechanical ventilation after 48 h. In the 14 other cases, mechanical ventilation was indicated after the caesarean section. Symptomatic treatment and antibiotic therapy had been started upon admission in all the patients. Five patients were intubated 12 days, 5 patients died of massive pulmonary embolism, 5 of the patients died of pulmonary superinfection, 4 patients died of severe hypoxia.

**Conclusion:** This is a global pandemic with high mortality worldwide. Published studies on SARS-COV-19 infection during pregnancy are few; they are no more at risk of being infected with the coronavirus, but the disease can have significant consequences on the course of their pregnancy. They are also more prone to severe forms of Covid-19.

**Compliance with ethics regulations:** N/A.

### FC-272 Pregnant women and COVID19: hospitalization in intensive care unit predictive factors

#### JAAFAR Nour Zayneb^1^, FATHALLAH Ines ^1^, KOURAICHI Nadia^1^

##### ^1^Université de Tunis - El Manar, Hôpital régional de Ben Arous, Tunis, Tunisie

###### Correspondence: Nour Zayneb JAAFAR (jaafar.nour.zayneb@gmail.com)

*Annals of Intensive Care* 2022, **12(1):**FC-272

**Rationale:** Pregnant women, a vulnerable population, have not been spared from the COVID-19 pandemic. In the absence of vaccination, severe forms and acute respiratory distress syndromes (ARDS) have been reported.

**Patients and methods/Materials and methods:** We reviewed all data of confirmed COVID-19 pregnant patients hospitalized in our Hospital ICU and in gynecology’s department between September 2020 and September 2021 for the management of acute pneumopathy due to Sars-Cov-2. Infection was confirmed by RT-PCR, rapid test, or chest CT scan. Epidemiological, clinical, biologic and radiographicl data and outcomes were collected.

**Results:** Thirty-six patients were included with a mean age of 33 ± 5 years and a mean term of pregnancy of 34 ± 4 weeks. The median consultation time after the onset of symptoms was 3.5 days [1;7] and the median hospital stay was 4 days [1;7]. Among 36 pregnant women, no one of the patients was vaccinated against SARS-Cov-2. Eleven patients (34%) were hospitalized in the ICU. Symptoms were dominated by respiratory signs, cough and dyspnea, respectively 21 (65%) and 15 (47%) patients. The different ventilatory supports were: single oxygen therapy (50%), high concentration mask (34%), high flow oxygen therapy (12.5%), and invasive ventilation (16%). Predictive factors for hospitalization in ICU were: dyspnea [p < 10–3 RR = 5.25 95% CI [2.174; 12.679]], oxygen uptake [p = 0.01 RR = 3.81 95% CI [1.737; 8.393]], delay before consultation > 4.5 days [p = 0.021 RR = 3.897 95% CI [1.267; 11,991]] and a period of hospitalization before admission in ICU > 5.5 days [p = 0.002 RR = 6.33 95% CI [1.608; 24.939]]. Among the six patients who had invasive ventilation, tracheotomy was practiced at three patients (27%). The ICU stay was complicated by septic shock in 4 patients (36%) and a thromboembolic disease in 4 patients (36%). The median length of ICU stay was 11 [5.54] days. The ICU mortality was high around 18% against an overall mortality of 6%.

**Conclusion:** Mortality was significant in our study: 34% of patients required hospitalization in ICU with an overall mortality of 6%.

**Compliance with ethics regulations:** Yes in clinical research.

### FC-273 Prevalence and outcome of SARS-COV2 bacterial coinfection

#### GHABARA Racha^1^, AYED Samia^1^, DEBBICHE Lilya^1^, RACHDI Emna^1^, JARRAYA Fatma^1^, JAMOUSSI Amira^1^, BEN KHELIL Jalila^1^

##### ^1^HOPITAL ABDERRAHMEN MAMI, Ariana, Tunisie

###### Correspondence: Samia AYED (samia.ayed@yahoo.fr)

*Annals of Intensive Care* 2022, **12(1):**FC-273

**Rationale:** The rate of SARS-CoV2 bacterial coinfection is poorly defined. The decision to administer antibiotics early in the course of SARS-CoV2 infection depends on the likelihood of bacterial coinfection. The purpose of this study was to examine the prevalence of SARS-COV2 bacterial co-infection on admission and to investigate the impact on outcome.

**Patients and methods/Materials and methods:** This was a retrospective study carried within a medical intensive care unit in Abderrahmen Mami Hospital between March 2020 and August 2021. All consecutive patients admitted for severe COVID-19 were enrolled. Bacterial co-infection was systematically investigated on intensive care unit (ICU) admission. A comparative study was conducted between the coinfection group and a matched non-coinfection group based on age, body mass index, severity scores and PaO2/FiO2 ratio on admission.

**Results:** During the study period, 544 patients were hospitalized in the intensive care unit for severe COVID-19. Fifty-four per cent of patients received antibiotics before ICU admission (n = 292) including amoxicillin–clavulanic acid association (n = 85), azithromycine (n = 129) and levofloxacine (n = 29). Only 49 patients showed bacterial coinfection on admission (9%). Most involved pathogens were: enterobacteriaceae (Klebsiella n = 15, *Enterobacter cloacae* n = 2, *Serratia marcescens* n = 2), *Staphylococcus aureus methicilline sensitive* (n = 5) and non-fermentative Gram-negative bacilli such as *Pseudomonas aeruginosa* (n = 6). Twenty-one patients with bacterial coinfection received antibiotics prior to the ICU admission (43%). No statistical difference was found in the univariate analysis comparing the coinfection group and the matched non-coinfection group. Only SGOT and SGPT levels were significatively higher in the coinfection group (p = 0,05 and p = 0,03 respectively). Need for mechanical ventilation and mortality did not differ significatively between the two groups (p = 0,371 and p = 0,469 respectively).

**Conclusion:** Bacterial coinfection rate in patients admitted with severe SARS-CoV2 infection is low. No significative difference was found between coinfected and non-coinfected group. These findings support unrecommended systematic antibiotics association to treat severe viral infection.

**Compliance with ethics regulations:** Yes in clinical research.

## Supplementary Information

**Additional file 1.** Disclosures of links of interest.

